# Assessment of Conjugation Pathways in *N*-Methylporphyrins that Are Fused to Acenaphthylene, Phenanthrene, or Pyrene: Evidence for the Presence of Alternative Aromatic Circuits

**DOI:** 10.1021/acs.joc.4c01760

**Published:** 2024-10-25

**Authors:** Jared S. Salrin, Brian G. Carpenter, Deyaa I. AbuSalim, Timothy D. Lash

**Affiliations:** † Department of Chemistry and Biochemistry, Illinois State University, Normal, Illinois 61790-4160, United States; ‡ Department of Chemistry, Rowan University, Glassboro 08028, New Jersey; § STEM Department, Rowan College of South Jersey, Vineland 08360, New Jersey

## Abstract

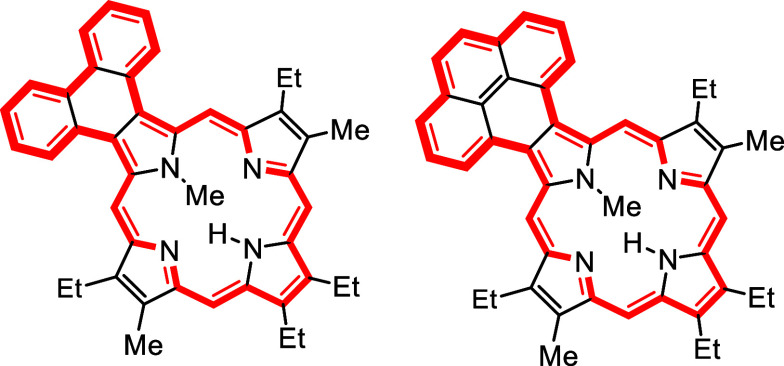

Acenaphtho-, phenanthro-, and pyrenopyrrole esters, readily available from Barton-Zard reactions of ethyl isocyanoacetate with nitroarenes, were reacted with methyl iodide and KOH in DMSO to give *N*-methylpyrroles and subsequent cleavage of the ester moieties was accomplished with KOH in ethylene glycol at 200 °C. Condensation with two equiv of an acetoxymethylpyrrole in refluxing acetic acid-2-propanol afforded a series of annulated tripyrranes. Cleavage of the terminal *tert*-butyl ester groups with trifluoroacetic acid, followed by condensation with a diformylpyrrole and oxidation with FeCl_3_, gave *N*-methyl acenaphtho-, phenanthro-, and pyrenoporphyrins. The *N*-methyl substituent effectively freezes the tautomeric equilibria to maximize interactions between the porphyrin nucleus and the fused aromatic substructures. Analysis of the proton NMR spectra provides evidence of the presence of extended aromatic circuits within these structures. Anisotropy of induced ring current (AICD) plots clearly shows the presence of 30π electron pathways in phenanthro- and pyrenoporphyrins that run around the exterior of the benzenoid fragments. These results demonstrate that *N*-alkylation can be used to relocate aromatic pathways in porphyrinoid systems.

## Introduction

**Scheme 1:**
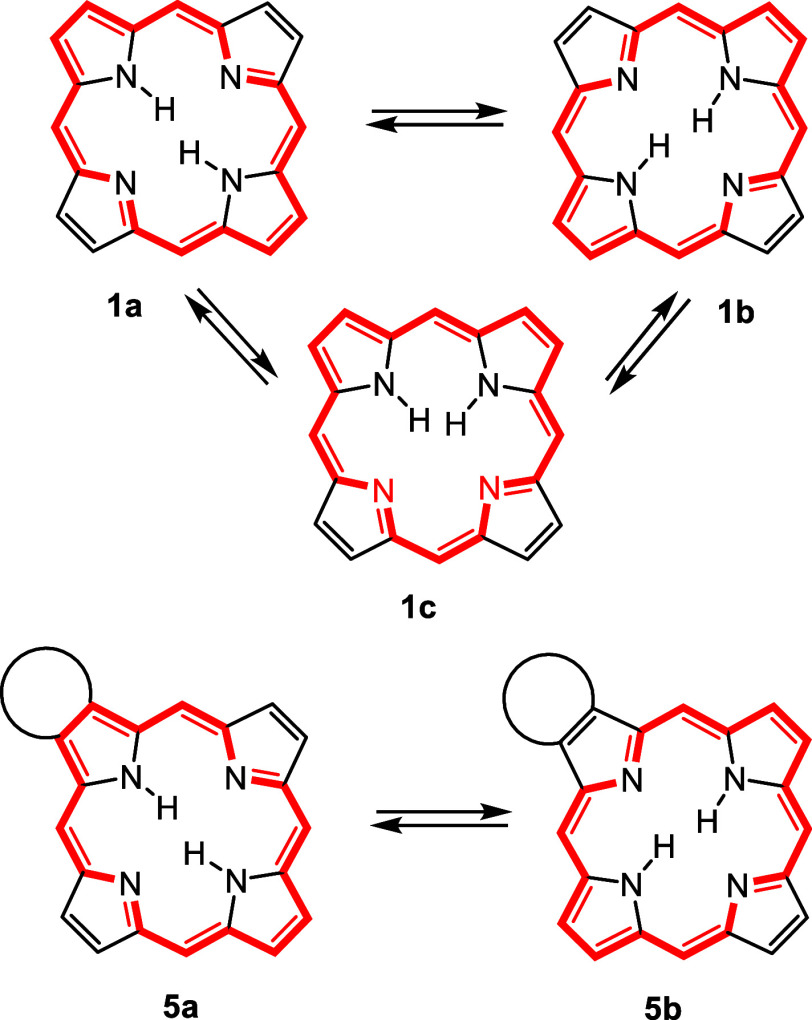
Porphyrin Tautomers

**Figure 1:**
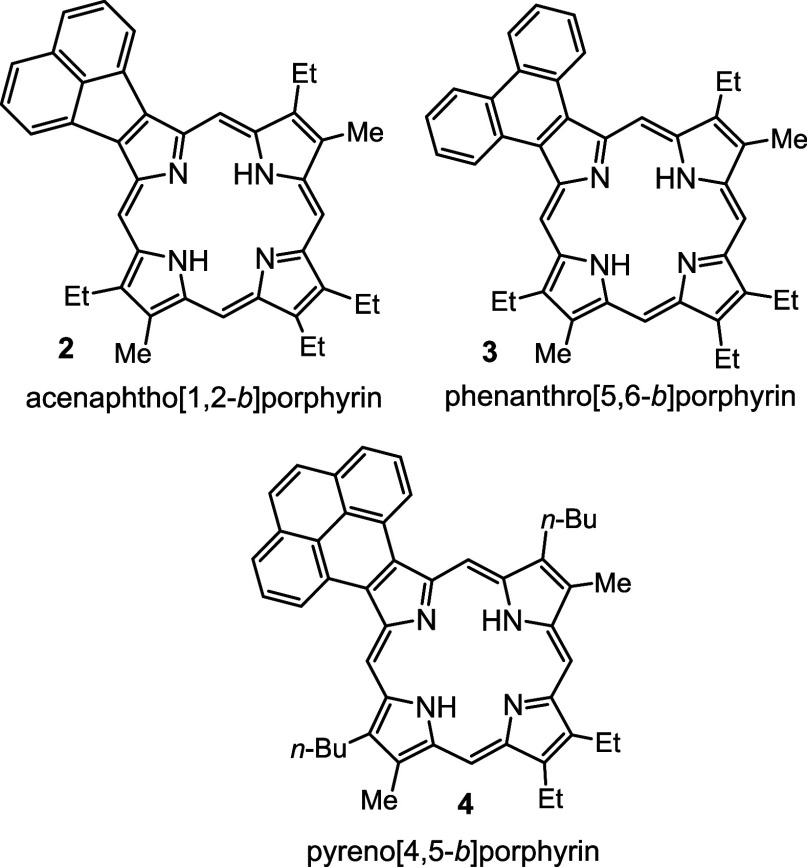
Examples of β,β’-annulated porphyrins.

Porphyrins and their derivatives fulfill a plenitude of biological functions that range from electron transport and oxygen transportation to photosynthesis.^1,2^ The free base forms of symmetrical porphyrins exist as two major interconverting tautomers **1a** and **1b** (Scheme 1) with N–H protons arranged opposite to one another and adjacent NH tautomers **1c** are significantly less stable due to steric crowding and less effective hydrogen bonding interactions.^3^ As compounds **1a** and **1b** are equivalent, they represent identical chromophores. However, when the structures are asymmetrically substituted, one form may be favored over the other.^4^ Porphyrins with β,β’-fused aromatic rings have been widely investigated as these can extend the π-system and modify the electronic structures of these pigments.^5−7^ This in turn can lead to properties that may be suitable for medicinal applications^8^ (e.g., as photosensitizers for photodynamic therapy^9−11^ or sensor development.^12,13^ In the case of monoannulated porphyrins such as acenaphthoporphyrins **2**,^14^ phenanthroporphyrins **3**,^15,16^ pyrenoporphyrins **4**,^17^ and related polycyclic aromatic hydrocarbon-fused porphyrins^18,19^ (Figure 1), the two *opp*-tautomeric forms, represented in the general case as **5a** and **5b** (Scheme 1), are distinctly different chromophores. Furthermore, the aromatic characteristics and preferred aromatic delocalization pathways could potentially be profoundly altered as the usual porphyrin-type 18π electron circuit in **5b** bypasses the fused unit while in principle **5a** can enable the introduction of extended conjugation routes. Tautomerization processes occur rapidly at room temperature, and the poor solubility of annulated porphyrins makes low temperature studies inaccessible. In order to assess these issues, a series of *N*-methyl annulated porphyrins were synthesized that essentially freeze the tautomeric equilibria so that type **5a** is the only available structure with an NH at the opposite position. By considering the proton NMR spectra for these porphyrins and carrying out DFT calculations, nucleus independent chemical shift (NICS) analyses, and assessing anisotropy of induced current density (AICD) plots, evidence for extended aromatic conjugation pathways in β,β’-annulated porphyrins was obtained.

## Results and Discussion

**Figure 2:**
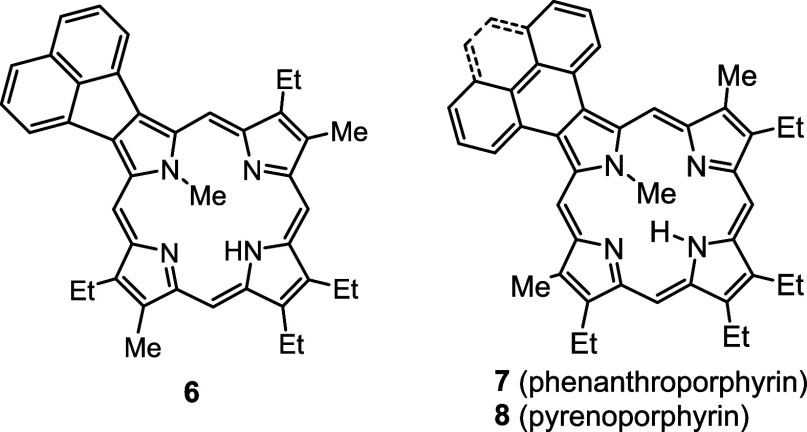
*N*-methyl annulated porphyrins.

**Scheme 2:**
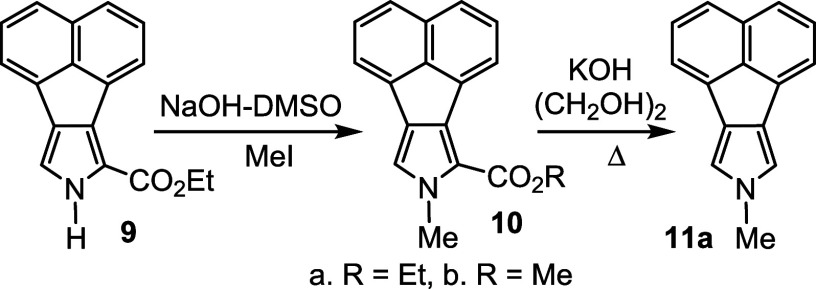
Synthesis of an *N*-Methylacenaphthopyrrole

**Scheme 3:**
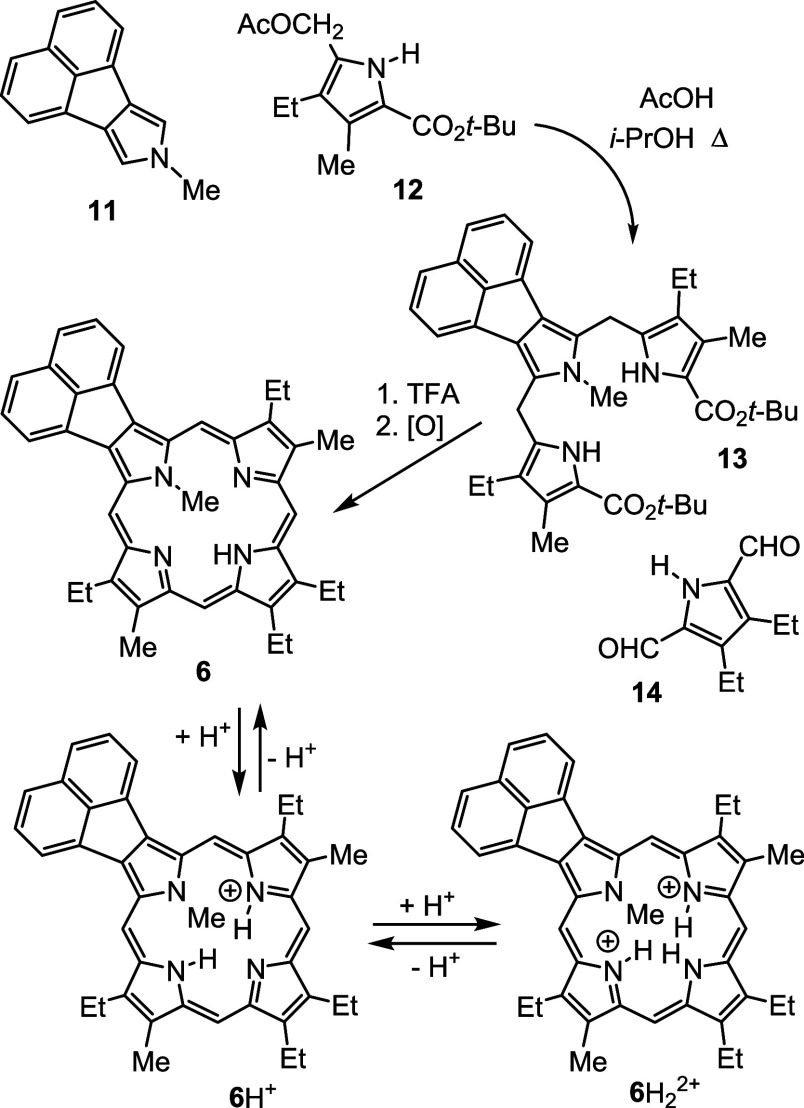
Synthesis of a 21-Methylacenaphthoporphyrin

*N*-methyl porphyrins **6**–**8** (Figure 2) with fused acenaphthylene, phenanthrene, and pyrene rings were targeted for synthesis. The *c*-annelated pyrrolic precursors were easily obtained from nitroaromatic compounds,^20,21^ using a modification of the Barton-Zard reaction.^22^ Acenaphtho[1,2-*c*]pyrrole **9**(14) (Scheme 2) was reacted with methyl iodide and potassium hydroxide in dimethylsulfoxide (DMSO) to introduce the required *N*-methyl substituent. Unexpectedly, partial transesterification occurred to give a mixture of ethyl and methyl esters **10a** and **10b**. The formation of **10b** presumably results from partial saponification of the ethyl ester, followed by the S_N_2 attack of the resulting carboxylate ion onto methyl iodide. As the ester groups were cleaved in the next step, the mixture was used without any attempts being made to avoid this side reaction or separate the two products. Hence, the mixtures of **10a** and **10b** were heated with KOH in ethylene glycol at 200 °C to give unsubstituted acenaphthopyrrole **11**. Condensation of **11** with acetoxymethylpyrrole **12** to give *N*-methyl acenaphthotripyrrane **13** was accomplished in 90% yield by heating the reactants in 2-propanol containing 5% acetic acid. The product precipitated from solution in pure form and required no further purification, apart from suction filtration and drying in vacuo. Treatment of **13** with trifluoroacetic acid (TFA) cleaved the terminal *tert*-butyl ester protective groups and subsequent condensation with pyrrole dialdehyde **14**, followed by oxidation with aqueous ferric chloride solutions, gave acenaphthoporphyrin in 51% yield (Scheme 3).

**Scheme 4:**
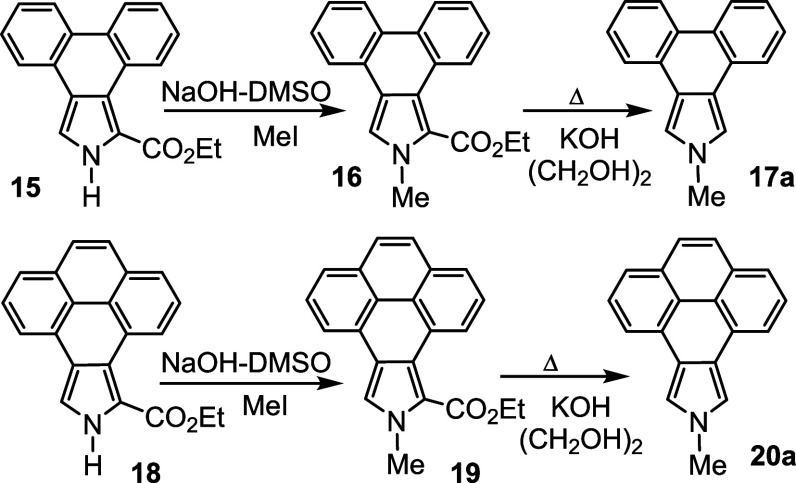
Alkylation of Phenanthro- and Pyrenopyrroles

**Scheme 5:**
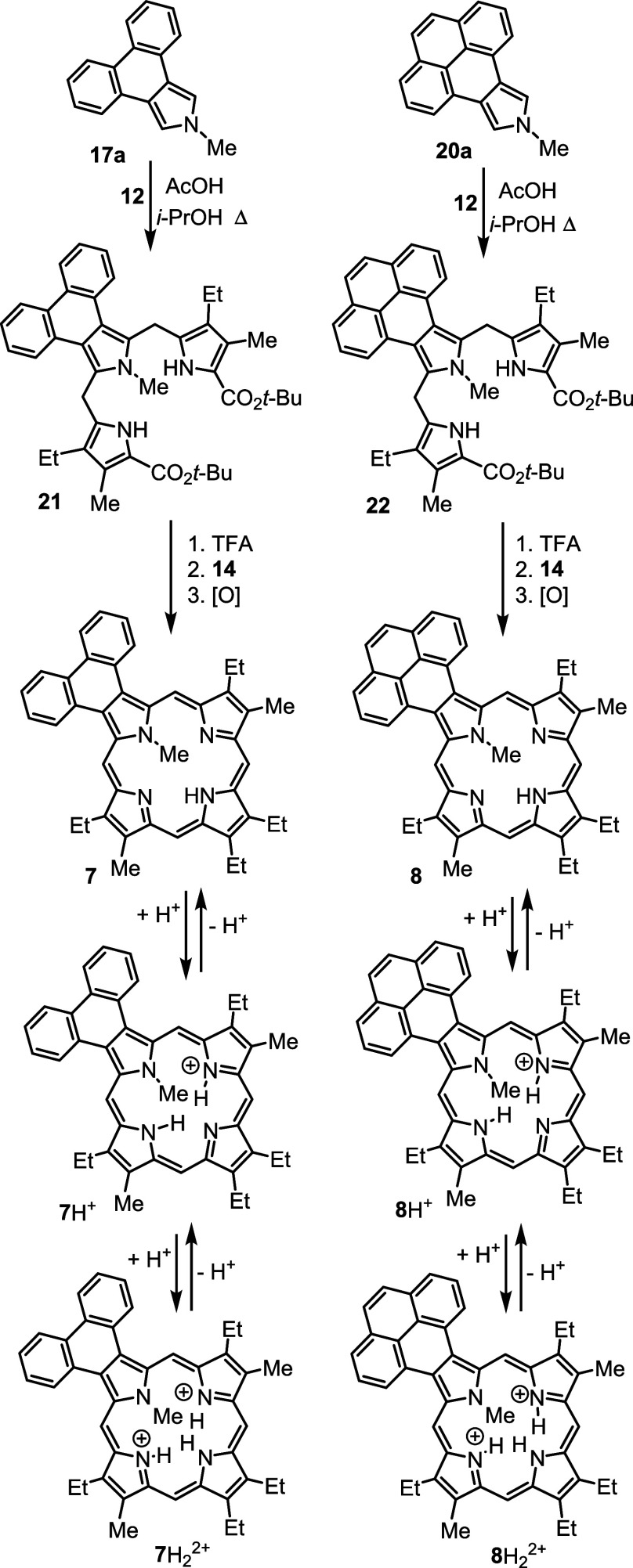
Synthesis of *N*-Methylporphyrins with Fused Phenanthrene and Pyrene Rings

A similar strategy was employed to synthesize *N*-methylporphyrins **7** and **8**. Reaction of phenanthropyrrole ethyl ester **15**(15) with methyl iodide and KOH in DMSO gave *N*-methyl derivative **16** (Scheme 4), although no transesterification was observed in this case. This difference is most likely due to the region around the carbonyl moiety being comparatively hindered due to the presence of a wider phenanthrene unit that sterically inhibits saponification of the ester group. Heating with KOH in ethylene glycol **16** afforded 2-methylphenanthro[5,6-*c*]pyrrole **17a** in a 61% yield. Alkylation of pyrenopyrrole ethyl ester **18**(17) with MeI-KOH in DMSO also gave *N*-methyl product **19** without any transesterification occurring and subsequent cleavage of the ester moiety with KOH in ethylene glycol at 200 °C generated 2-methylpyreno[4,5-*c*]pyrrole **20a** (Scheme 4). Reaction of **17a** or **20a** with two equivalents of acetoxymethylpyrrole **12**(23) in acetic acid-2-propanol gave phenanthrotripyrrane **21** and pyrenotripyrrane **22**, respectively, in 80–87% yield (Scheme 5). Tripyrrane **21** was stirred with TFA at room temperature for 10 min, and the solution diluted with dichloromethane and dialdehyde **14**(24) was added. After 2 h, the mixture was neutralized with triethylamine, and the products were oxidized with aqueous ferric chloride. Following column chromatography and recrystallization from chloroform–methanol, phenanthroporphyrin **7** was isolated in 13% yield. Following the same protocol, tripyrrane **22** was deprotected with TFA and condensed with pyrrole dialdehyde **14** to give, following purification, the related pyrenoporphyrin **8** in 16% yield (Scheme 5).

**Figure 3:**
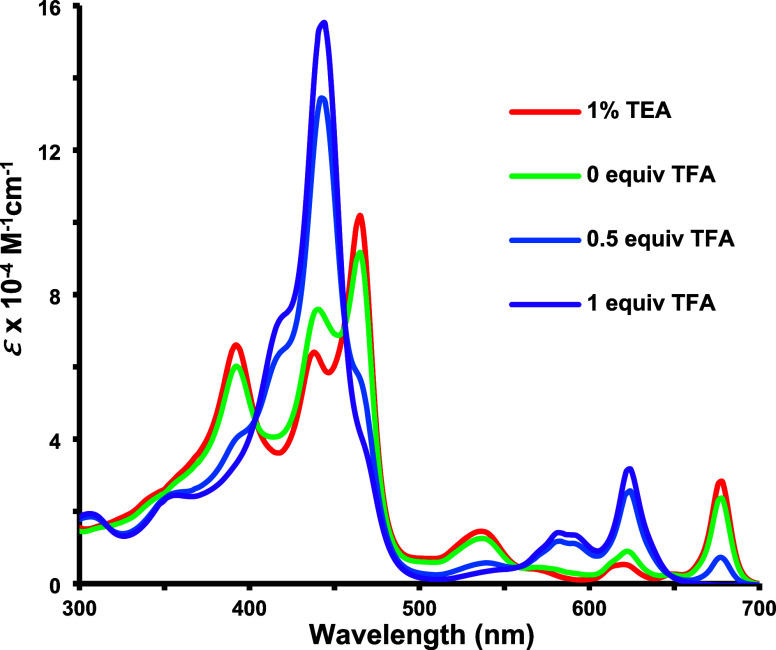
UV–vis spectra of acenaphthoporphyrin **6** in 1% Et_3_N–CH_2_Cl_2_ and in CH_2_Cl_2_ with 0–1 equiv of TFA.

**Figure 4:**
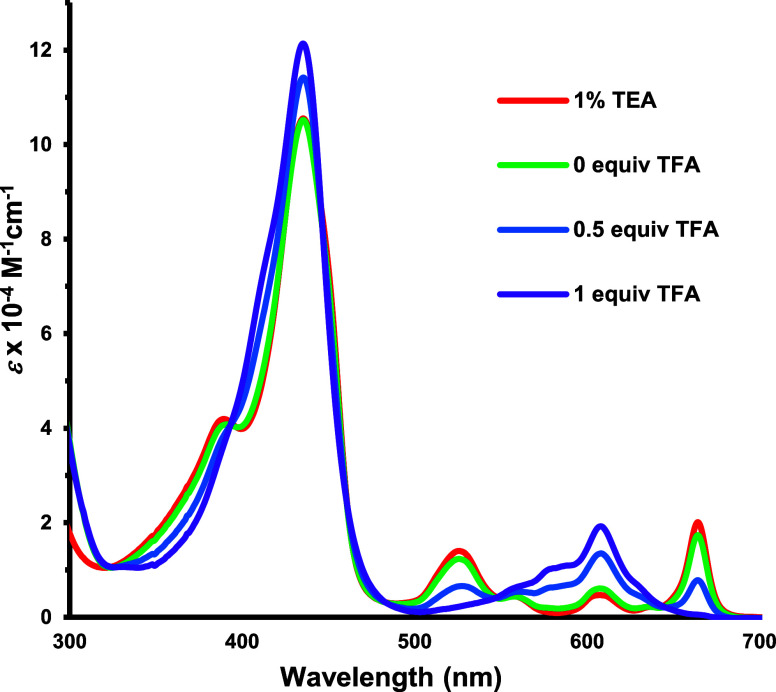
UV–vis spectra of phenanthroporphyrin **7** in 1% Et_3_N–CH_2_Cl_2_ and in CH_2_Cl_2_ with 0–1 equiv of TFA.

**Figure 5:**
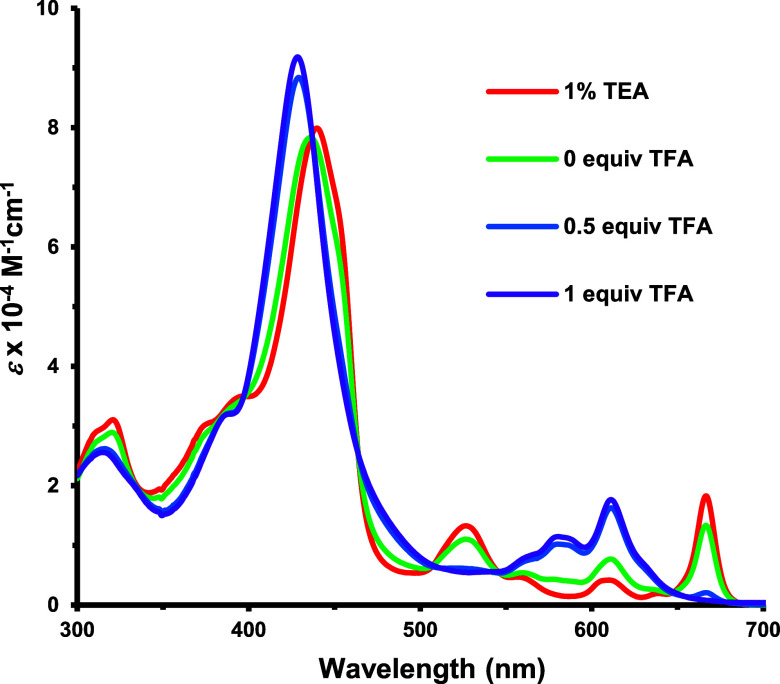
UV–vis spectra of pyrenoporphyrin **8** in 1% Et_3_N–CH_2_Cl_2_ and in CH_2_Cl_2_ with 0–1 equiv of TFA.

The UV–vis spectra of acenaphtho-, phenanthro- and pyrenoporphyrins show substantial differences from one another^18^ and introduction of *N*-methyl substituents leads to bathochromic shifts and peak broadening compared to the corresponding *N*-unsubstituted porphyrins.^25,26^ Acenaphthoporphyrin **6** in 1% Et_3_N–CH_2_Cl_2_ gave three Soret bands at 393, 438, and 465 nm and a long wavelength Q-band at 677 nm (Figure 3). Addition of trace amounts of TFA to solutions in CH_2_Cl_2_ gave rise to a monoprotonated species **6**H^+^ with a more intense Soret band at 443 nm (Figure 3) and further protonation occurs at higher TFA concentrations to generate dication **6**H_2_^2+^ (Scheme 3, Figures S3–S6). In 5% TFA, this species gave rise to a Soret band at 441 nm. Phenanthroporphyrin gave a strong Soret band at 435 nm and a long wavelength Q-band at 664 nm (Figure 4). Addition of 1 equiv of TFA afforded a monocationic species **7**H^+^ with a slightly intensified Soret band at 435 nm (Figure 4), but a dicationic species **7**H_2_^2+^ (Scheme 5) was generated at higher acid concentrations (Figures S9–S12). In 10% TFA-CH_2_Cl_2_, the Soret band shifted to 411 nm. Pyrenoporphyrin **8** also gave a single Soret band at 440 nm in 1% Et_3_N–CH_2_Cl_2_ and a distinctive long wavelength Q-band appeared at 667 nm (Figure 5). In the presence of 1 equiv of TFA, the corresponding monocation **8**H^+^ was formed, and this gave a Soret band at 428 nm. Once again, a second protonation occurred at higher acid concentrations to give dication **8**H_2_^2+^ (Figures S15–S17). In 5% TFA-CH_2_Cl_2_, this showed two Soret bands at 406 and 428 nm.

**Figure 6:**
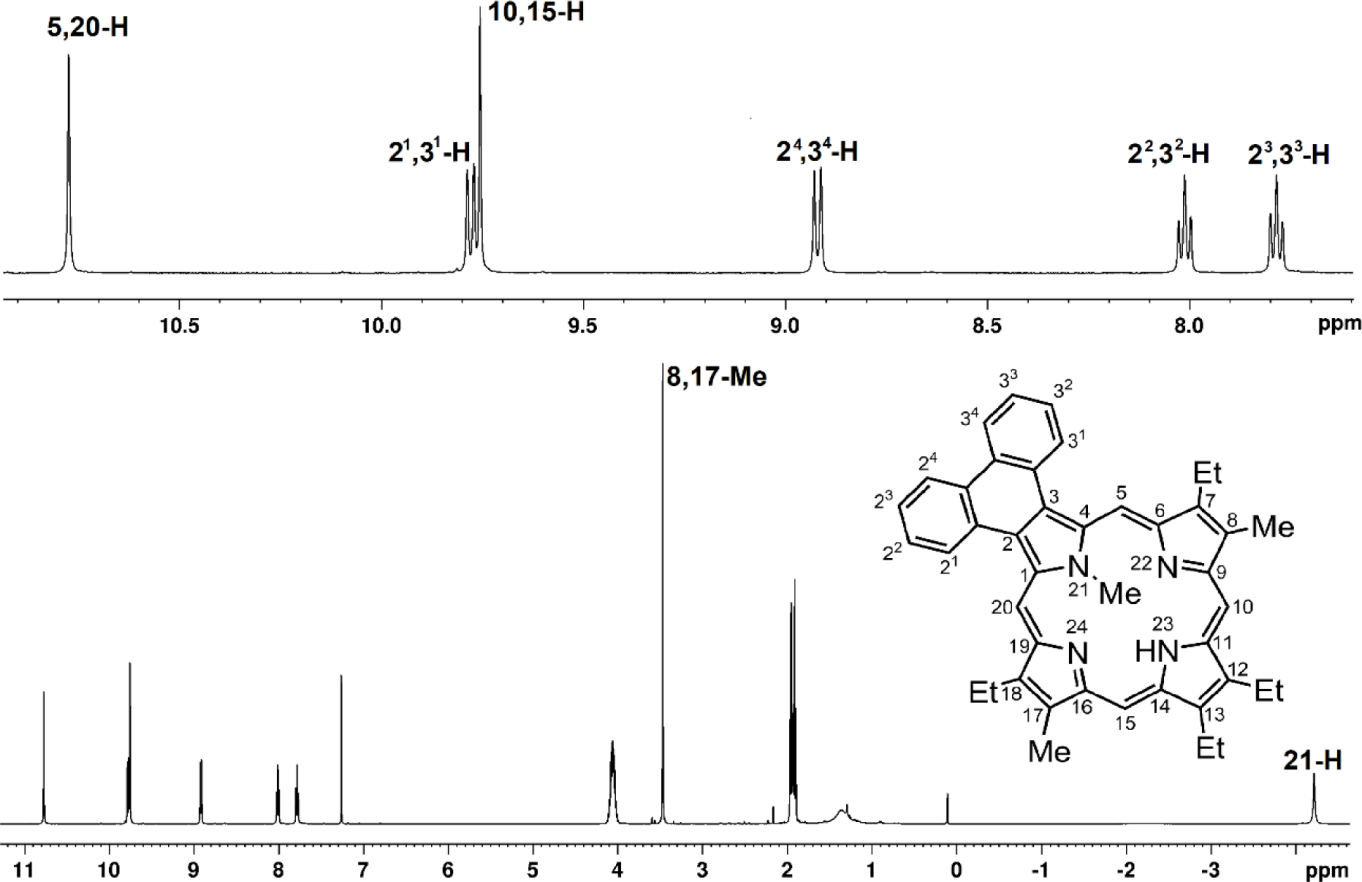
Proton NMR spectrum of phenanthroporphyrin **7** in CDCl_3_ at 50 °C.

Important insights can be obtained by examining the proton NMR spectra for porphyrins **6**, **7** and **8**. A typical proton NMR spectrum for phenanthroporphyrin **7** in CDCl_3_ is shown in Figure 6. The internal N-Me resonance appears upfield near −4 ppm, while the *meso*-protons gave rise to two strongly deshielded 2H singlets. While these results demonstrate that the system is fully aromatic, interesting trends can be discerned when comparing selected chemical shifts for the *N*-Me series with the corresponding *N*-unsubstituted porphyrins (Table 1). The *meso*-protons flanking the fused ring systems are further deshielded and provide a less reliable measure than the peaks for the further removed *meso*-protons at positions 10 and 15. The 8,17-methyl substituents also provide an independent measure as porphyrin methyls are greatly influenced by the aromatic ring current, commonly shifting downfield to ca. 3.6 ppm.^27^ A comparison of the resonances for the 10,15-H and 8,17-Me resonances for **2**, **3**, and **4**(14−17) show no significant differences in the shifts, indicating that all three systems have comparable diatropic ring currents. However, the corresponding *N*-methylporphyrins all exhibit reduced diatropicity. For acenaphthoporphyrins **2** and **6**, the *N*-methyl substituent causes the *meso*-protons to shift upfield by 0.20–0.32 ppm and the 8,17-methyl substituents similarly move upfield by 0.19 ppm (Table 1). Phenanthroporphyrin **7** is also affected compared to its *N*-unsubstituted congener **3** and the *meso*-proton resonances appear 0.27–0.29 ppm upfield in the *N*-methyl version. The 8,17-methyl resonance also moves 0.23 ppm upfield. Pyrenoporphyrin **8** shows the same shifts for the 10,15-H and 8,17-Me peaks but not for the 5,20-H resonances. As the 5,20-protons lie directly next to the pyrene nucleus, this effect can be attributed to conformational differences. Some caution must be applied in these analyses, as some shifts can be observed due to changes in concentration and temperature, but the trends appear to be consistent. The decreased diatropicity of the *N*-methyl series can be attributed to distortion of the porphyrin macrocycle due to steric crowding. DFT calculations (see below) show that the *N*-methyl porphyrins are severely distorted compared to the *N*-unsubstituted systems. Using the 10,15-H, 8,17-Me, and 21-Me resonances as a guide, *N*-methylacenaphthoporphyrin **6** has the smallest ring current with the least shielded internal methyl group and smaller downfield shifts for the external protons. Phenanthro- and pyrenoporphyrins **7** and **8** show similar values, although the upfield shifts for the 21-methyl group in **7** are slightly larger. Nevertheless, all of these structures retain a high degree of aromatic character. Further characterization was carried out on the protonated porphyrins, and the carbon-13 spectra were fully consistent with the proposed structures. Further support for these structures was obtained using high resolution mass spectrometry to confirm the molecular formulas.

**Table 1:**
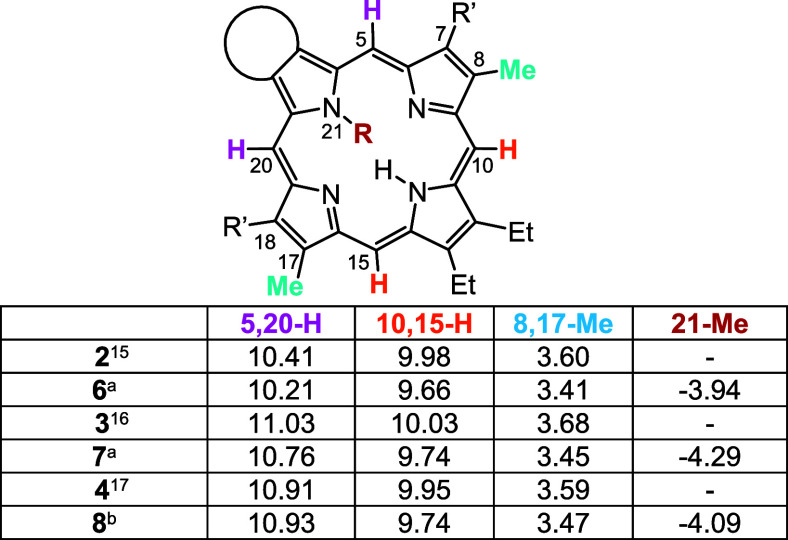
Proton NMR Chemical Shifts for Selected Resonances for Annulated Porphyrins in CDCl_3_

a The spectra were run at 50 °C.

b Spectrum obtained at 55 °C.

**Scheme 6:**
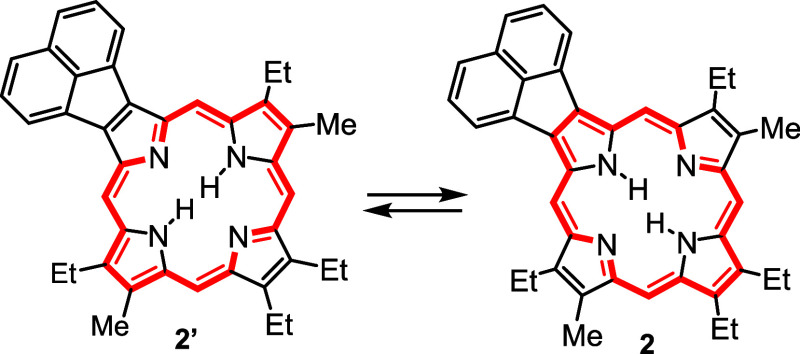
Major Tautomers for Acenaphtho[1,2-*b*]Porphyrin **2**

**Scheme 7:**
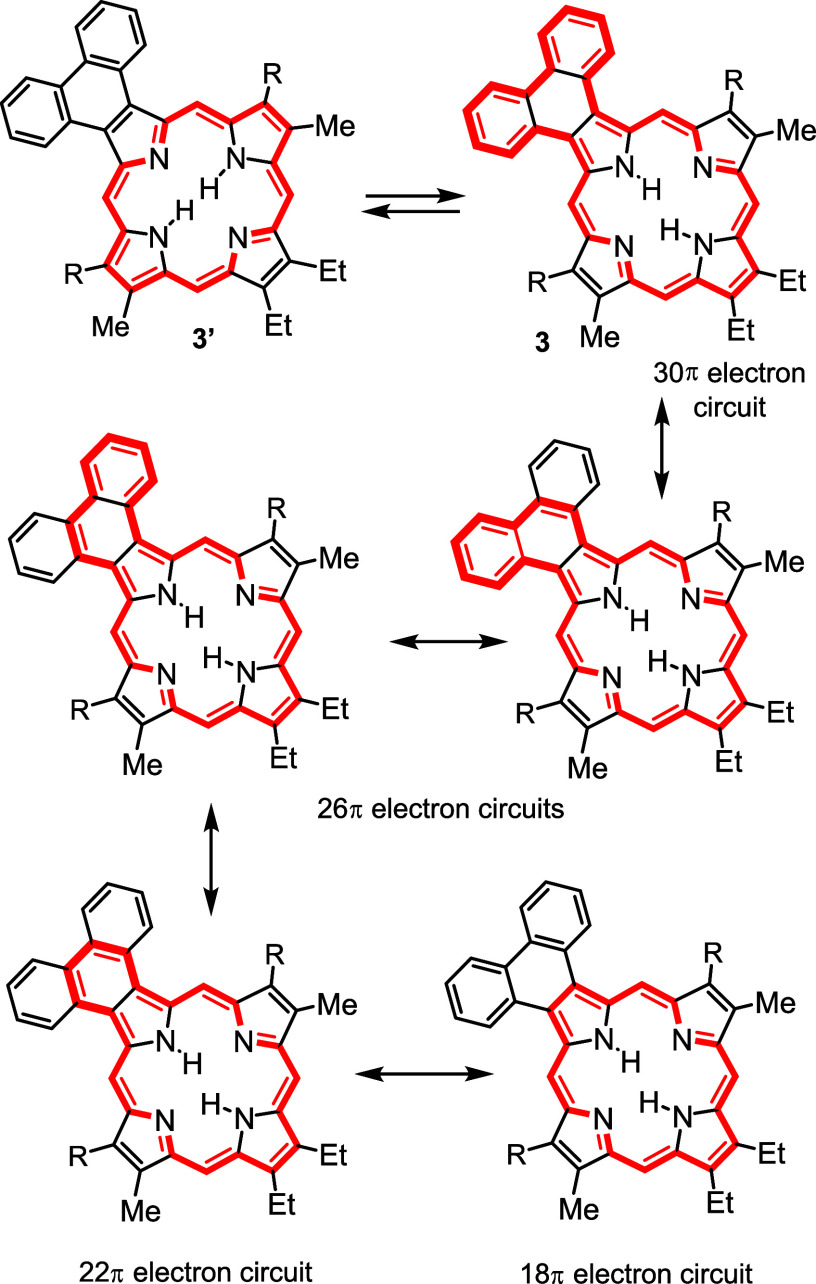
Tautomerism and Aromatic Conjugation Pathways in Phenanthro[5,6-*b*]porphyrins

**Scheme 8:**
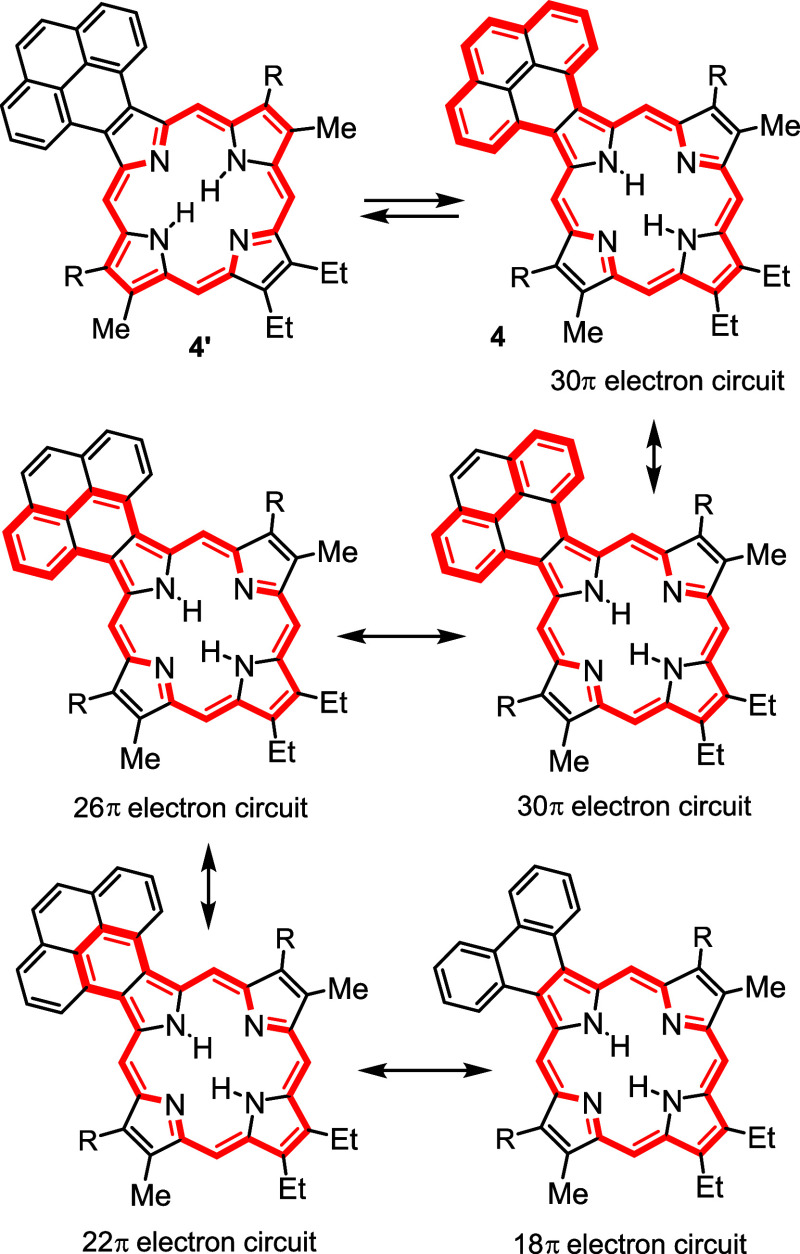
Tautomerism and Aromatic Conjugation Pathways in Pyreno[4,5-*b*]porphyrins

As noted in the introduction, the two major tautomers for porphyrins **2**–**4** may have significantly different properties.^14^ The tautomers for acenaphthoporphyrin, **2** and **2’** (Scheme 6) both seemingly isolate the acenaphthylene unit from the porphyrin core and may only exhibit minor differences. However, while the phenanthrene unit in phenanthroporphyrin tautomer **3′** might be expected to have only minor interactions with the porphyrin π-system, tautomer **3** can potentially merge with the macrocyclic π-system. Canonical forms with 30, 26, 22, and 18π electron circuits can be considered (Scheme 7). Similarly, pyrenoporphyrin would be expected to have minimal interactions in tautomer **4’**, but **4** can enable the emergence of 30, 26, and 22π electron pathways in addition to the usual 18π electron route (Scheme 8). The same extended pathways found in **3** and **4** would be expected to be present in the corresponding *N*-methyl porphyrins **7** and **8**, although the reduced planarity of these structures could potentially reduce these effects. The presence of extended aromatic pathways will not necessarily increase the diatropic ring currents for these structures and in fact we recently reported examples of porphyrinoids where extension of the π-system dramatically reduced the aromatic properties.^28^ Furthermore, ring currents can be modified by the presence of electron-withdrawing or electron-donating groups,^29−31^ and these factors may also complicate analyses.

**Table 2:**
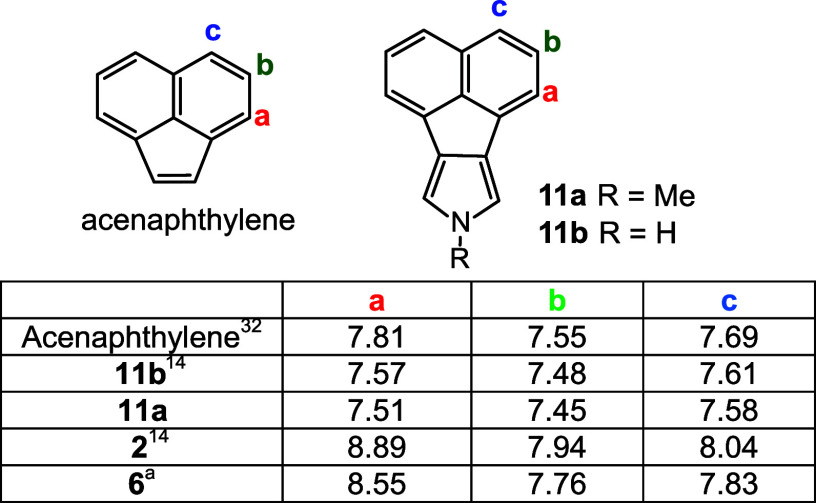
Chemical Shifts for Selected Proton NMR Resonances for Acenaphthylene, Acenaphthopyrroles, and Acenaphthoporphyrins in CDCl_3_

a The spectrum was obtained at 50 °C.

**Table 3:**
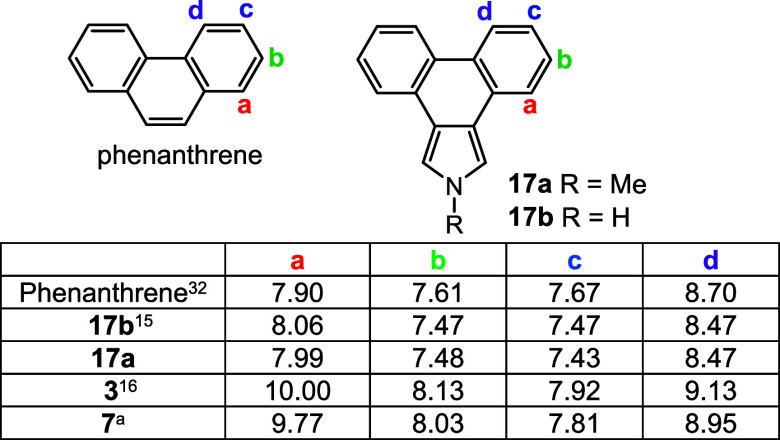
Chemical Shifts for Selected Proton NMR Resonances for Phenanthrene, Phenanthropyrroles, and Phenanthroporphyrins in CDCl_3_

a The spectrum was run at 50 °C.

If the π-system extends through the fused aromatic rings, then this should affect the chemical shifts for these subunits. Acenaphthylene, acenaphthopyrroles, and acenaphthoporphyrins were compared to assess this possibility. Protons directly next to the porphyrin system will give misleading results due to anisotropic deshielding and protons label **a** in Table 2 cannot be used to gauge the effects. However, proton resonances **b** and **c** give more meaningful insights. In acenaphthylene,^32^ protons **b** and **c** appear at 7.55 and 7.69 ppm, but these shift slightly upfield in acenaphthopyrroles **11b**(14) and **11a**. This effect can be attributed to the electron-donating effect of the electron-rich pyrrolic subunit. In acenaphthoporphyrin **2**,^14^ these peaks move downfield by 0.35–0.39 ppm, an effect that could be attributed to the presence of an extended π-system. Interestingly, these downfield shifts are essentially halved for *N*-methylporphyrin **6**, an effect that can be explained if the π-system is disrupted due to reduced planarity.

Analysis of phenanthrene, phenanthropyrroles, and phenanthroporphyrins showed similar trends (Table 3). Once again, anisotropic shielding of protons **a** directly next to the porphyrin system will overwhelm these effects. The chemical shifts for **b**, **c**, and **d** in phenanthropyrroles **17b**(15) and **17a** were shifted upfield by 0.13–0.24 ppm compared to phenanthrene due to the presence of the fused electron-rich pyrrole moiety. However, the same resonances are deshielded by 0.25–0.51 ppm in phenanthroporphyrin **3**,^16^ although the downfield shifts were reduced to 0.14–0.42 ppm in *N*-methylporphyrin **7** (Table 3). Pyrene shows a similar trend where protons **b**, **c**, and **d** are shifted upfield in pyrenopyrroles **20b**(17) and **20a** but significantly shifted downfield in porphyrins **4**(17) and **8** (Table 4). Once again, the shifts are slightly larger for *N*-unsubstituted pyrenoporphyrin **4** compared to *N*-methyl version **8**. Put together, the data suggest that the aromatic ring current in these porphyrins extends through the fused aromatic subunits. However, against expectations, the effects seem to apply to acenanthoporphyrins as well as phenanthroporphyrins and pyrenoporphyrins.

**Table 4:**
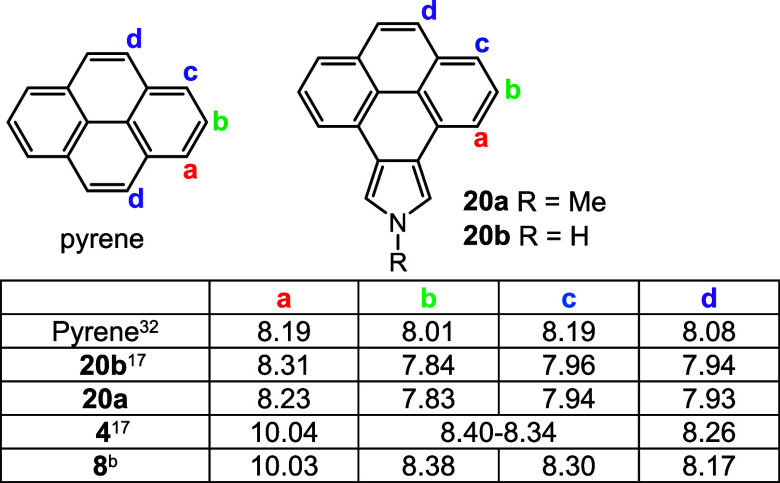
Chemical Shifts for Selected Proton NMR Resonances for Pyrene, Pyrenopyrroles, and Pyrenoporphyrins in CDCl_3_

b The spectrum was obtained at 55 °C.

**Figure 7:**
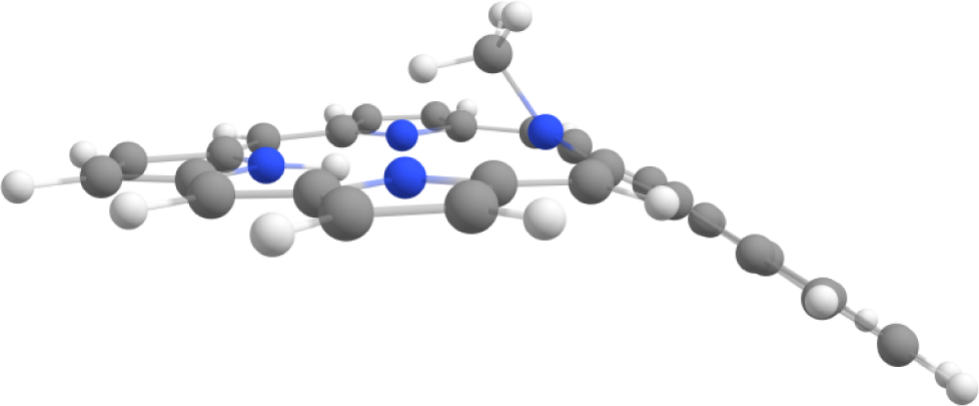
Calculated conformation (side view) for *N*-methylacenaphthoporphyrin **MeANPa**.

**Figure 8:**
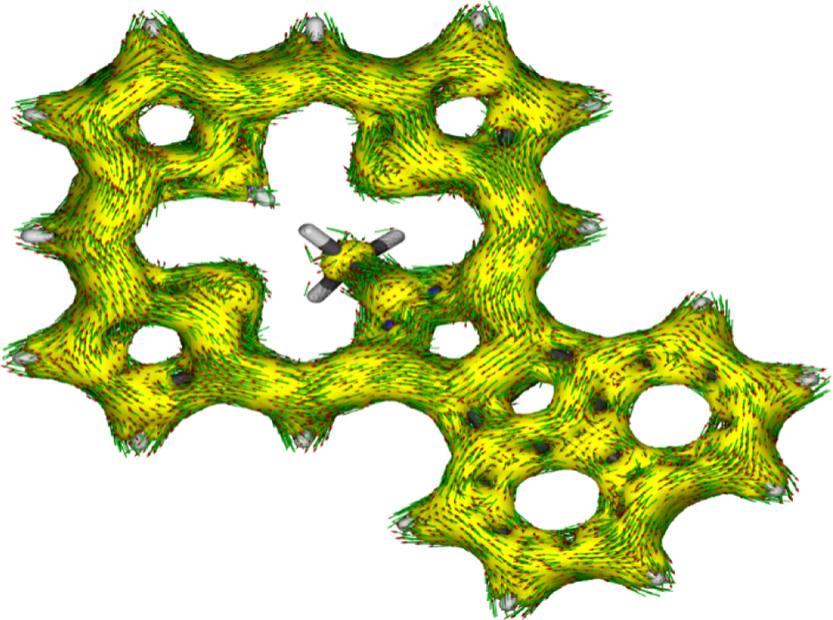
AICD plot (isovalues 0.05) of *N*-methylacenaphthoporphyrin **MeANPa**.

Density functional theory (DFT) calculations^33−37^ were carried out on a series of acenaphtho-, phenanthro-, and pyrenoporphyrin tautomers, their *N*-methyl derivatives, and selected protonated species. The structures were optimized by using M06-2X with the triple-ζ basis set 6–311++G(d,p). Four tautomers of unsubstituted acenaphthoporphyrin (**ANP**) were considered and as expected, the two forms with opposite N–H protons were shown to have the lowest energies (Table 5). Tautomers **ANPa** and **ANPb** have less than 1 kcal/mol differences in energy, and all four tautomers are highly diatropic as assessed by nucleus independent chemical shift calculations^38^ (Table 5). As standard NICS(0) calculations include effects due to σ and π electrons and are not always accurate, NICS(1)_*zz*_ calculations were also carried out. In NICS(1)*_zz_*, the calculations were performed 1 Å above the ring, and the results provide a more accurate measure of the diatropic ring currents. Negative values within a cyclic structure correspond to aromatic species, and while NICS(1)*_zz_* values are generally much larger than those for standard NICS calculations, the two methods showed similar trends. Positive values may indicate antiaromatic character but more often result from measurements of positions that are external to aromatic delocalization pathways. Two tautomers of *N*-methylacenaphthoporphyrin were considered (Table 6) and **MeANPa** with the inner N–H opposite the methyl group was shown to be favored. Although the ring system is significantly distorted due to steric congestion within the cavity (Table S1), the NICS calculations showed only a very slight reduction in the NICS(0) value. The *N*-methyl substituent is drastically pivoted away from the porphyrin core (Figure 7) and is essentially locked in place. When calculating NICS(1)*_zz_* values it was necessary to make the measurements on the face opposite to the *N*-methyl group as proximity to this group results in anomalous values. The porphyrin structures were also assessed using anisotropy of induced current density (AICD).^39^ The AICD plot for **MeANPa** (Figure 8) shows that the ring current associated with the naphthalene subunit is disconnected from the porphyrin aromatic circuit, and similar results have been seen for other acenaphthoporphyrin structures.

**Table 5:**
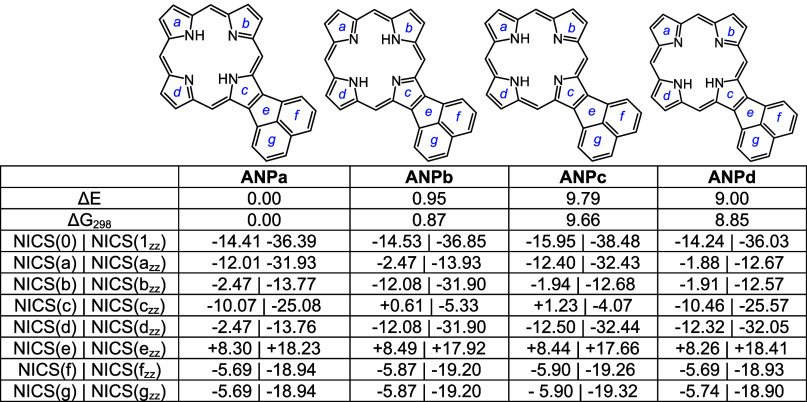
Calculated Relative Energies and NICS Values for Selected Acenaphthoporphyrin Tautomers

**Table 6:**
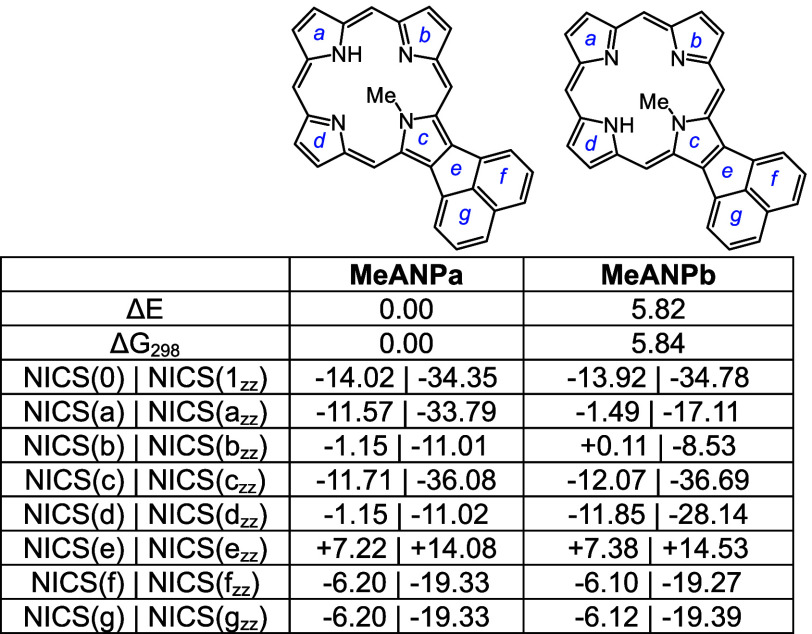
Calculated Relative Energies and NICS Values for *N*-Methylacenaphthoporphyrins

Three monoprotonated acenaphthoporphyrin tautomers were considered, and while **ANPb**H^+^ was the most favorable, the differences in energies were small (Table 7). NICS calculations showed that the monocations have comparable aromatic character to the free base structures. Two monocationic forms of *N*-methylacenaphthoporphyrin were calculated and tautomer **MeANP**H^+^ was significantly favored; both forms gave similar strongly aromatic NICS values (Table 8). Diprotonated **ANP** and **MeANP** gave strongly aromatic NICS values as well (Table 9) even though these are very distorted, particularly in the case of **MeANP**H_2_^2+^ (Table S1, Figures S105 and S107).

**Table 7:**
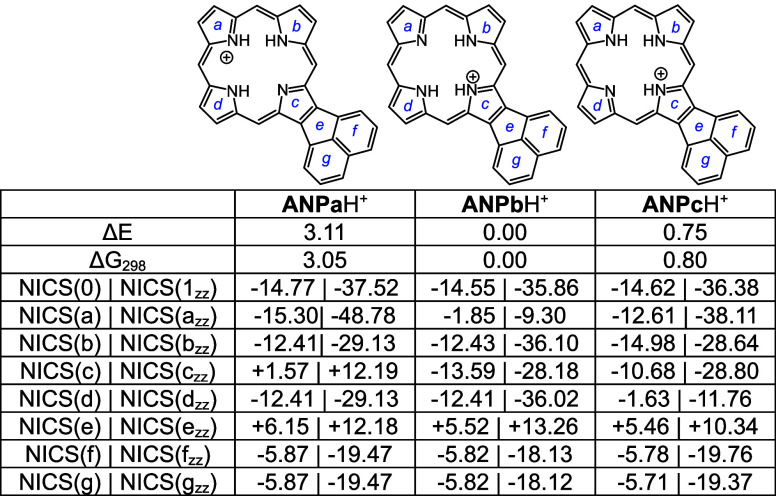
Calculated Relative Energies and NICS Values for Selected Acenaphthoporphyrin Monocations

**Table 8:**
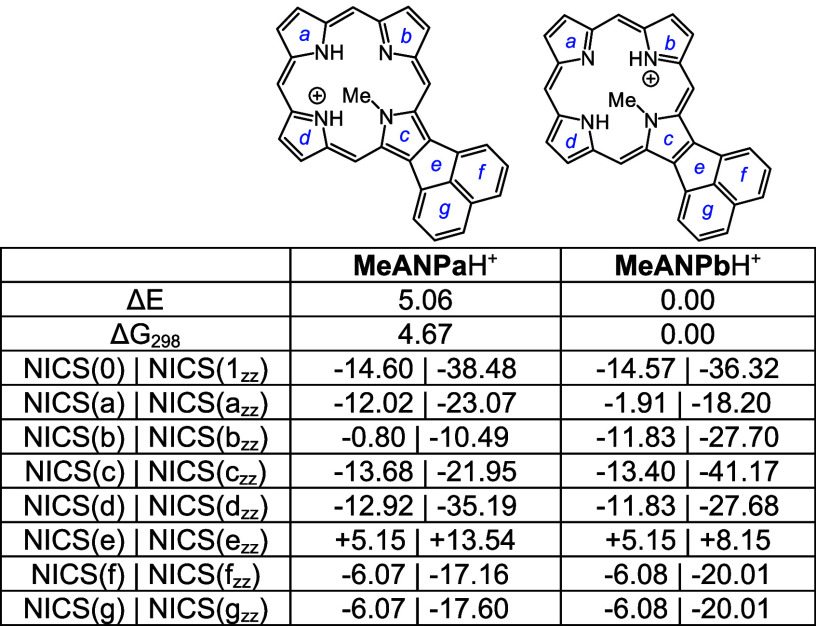
Calculated Relative Energies and NICS Values for *N*-Methylacenaphthoporphyrin Monocations

**Table 9:**
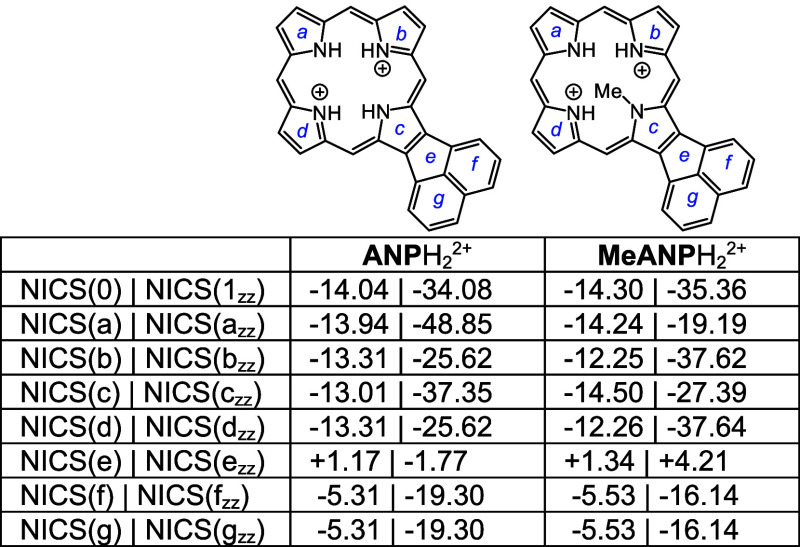
Calculated NICS Values for Acenaphthoporphyrin Dications

**Figure 9:**
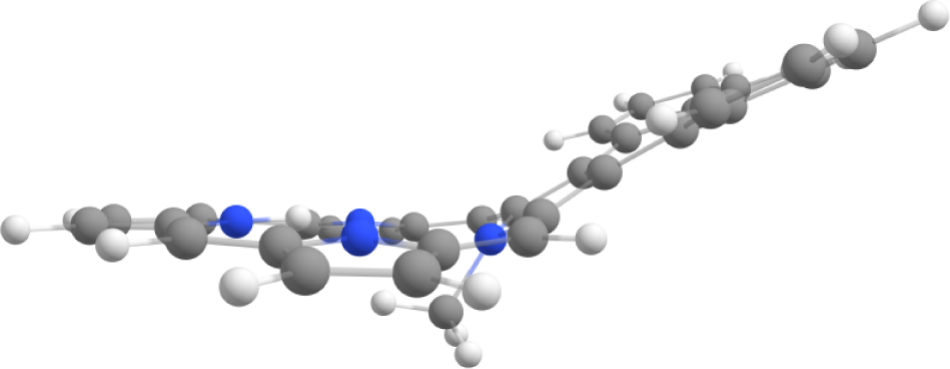
Calculated conformation (side view) for *N*-methylphenanthroporphyrin **MePhPa**.

**Figure 10:**
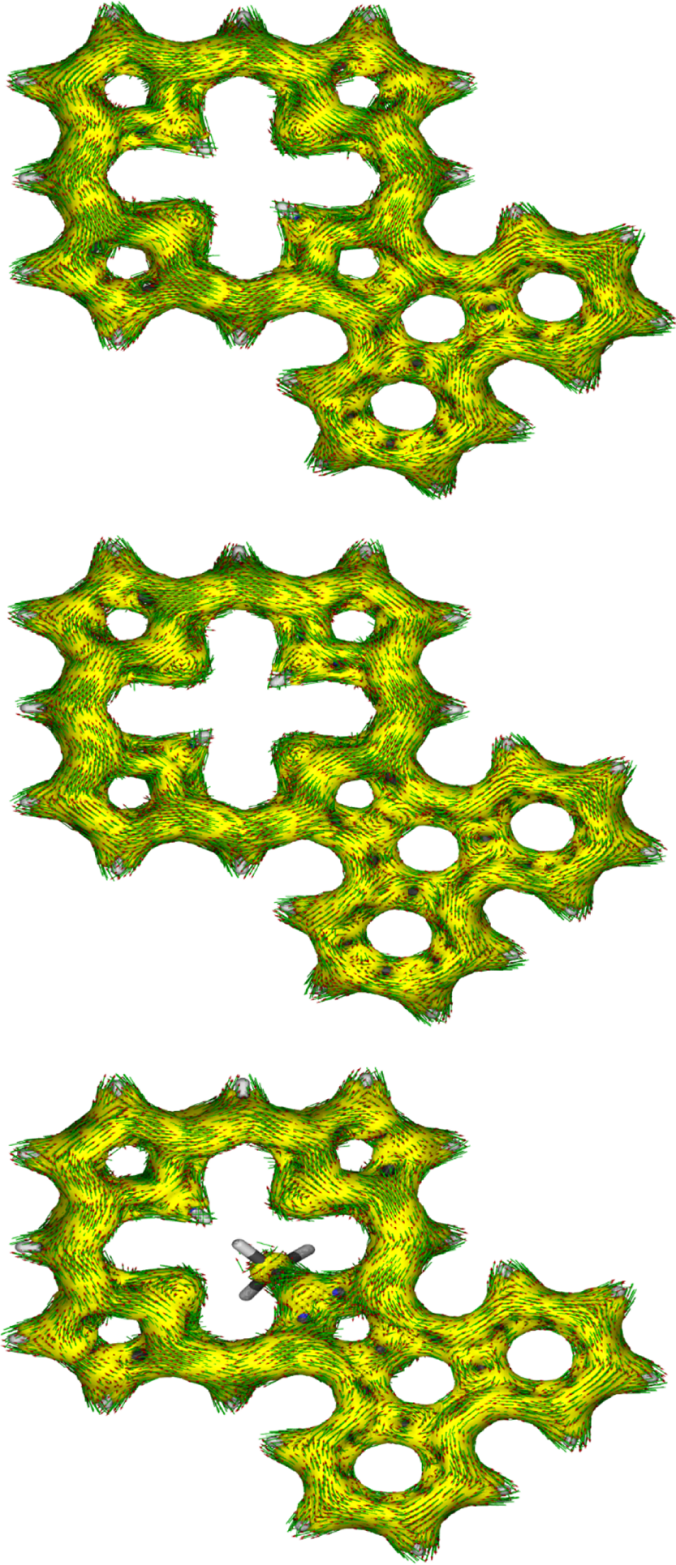
AICD plots (isovalues 0.05) of phenanthroporphyrin structures **PhPa** (top), **PhPb** (middle) and **MePhPa**. Extension of the aromatic circuits through fused phenanthrene rings is particularly evident in **PhPa** and **MePhPa**.

Four tautomers of phenanthroporphyrin (**PhP**) were considered and as before porphyrins with N–H protons opposite to one another are lower in energy by >8 kcal/mol. **PhPa** and **PhPb** have similar energies, although the latter is slightly favored over the former (Table 10). All four tautomers are reasonably planar (Table S2), but the *opp*-arrangement of N–H’s also facilitates hydrogen bonding and reduces steric crowding. Two *N*-methyl phenanthroporphyrins (**MePhP**s) were considered and the form with an N–H opposite to the *N*-methyl is lower in energy by ca. 6 kcal/mol. **MePhP**s are strongly distorted but show only slightly reduced negative NICS values (Table 11). Once again, the annulated pyrrole unit is twisted away from the macrocyclic core, and the *N*-methyl group is pivoted away from the system (Figure 9). AICD plots for these structures show significant interactions between the π-systems of the porphyrin nucleus and the fused phenanthrene rings (Figure 10). **PhPa** shows a significant 30π electron circuit that runs around the outside of the phenanthrene unit and this pathway can also be clearly seen in the analogous *N*-methyl derivative **MePhPa**. As expected, this pathway is less prominent for tautomer **PhPb**, although there is a degree of bifurcation and interactions with the fused arene unit are still present (Figure 10).

**Table 10:**
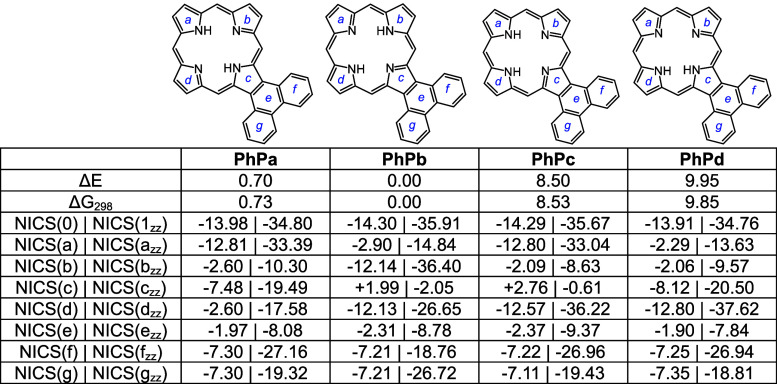
Calculated Relative Energies and NICS Values for *N*-Phenanthroporphyrin Tautomers

**Table 11:**
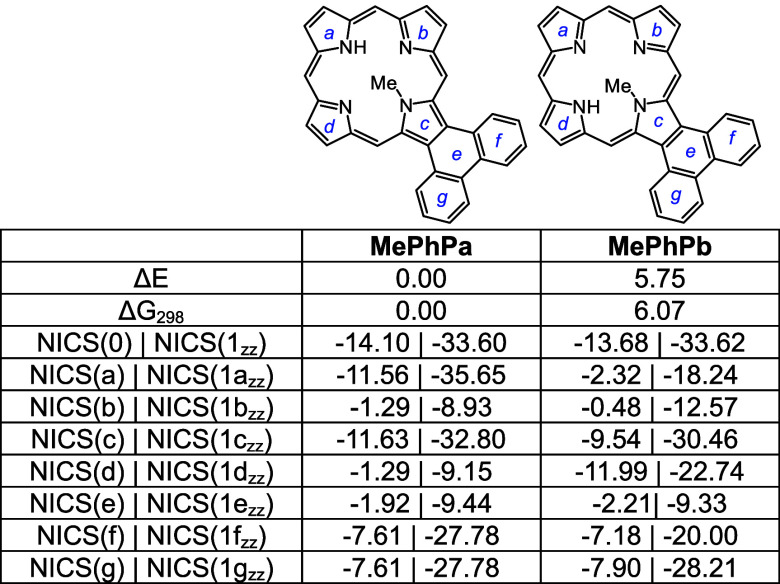
Calculated Relative Energies and NICS Values for *N*-Methylphenanthroporphyrins

**Table 12:**
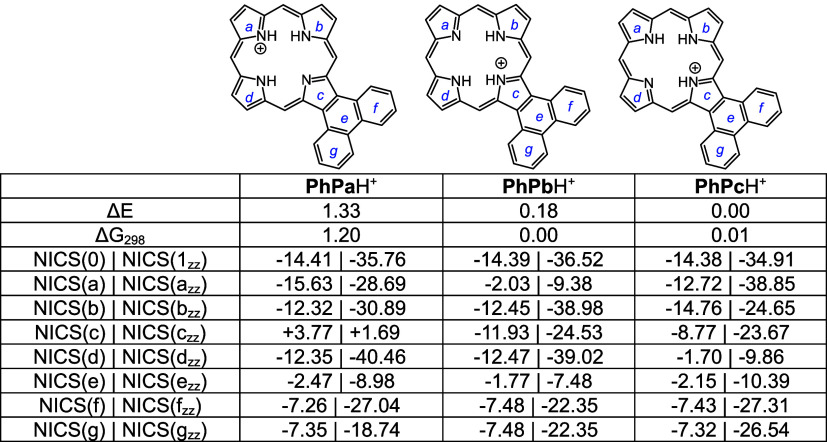
Calculated Relative Energies and NICS Values for Selected Phenanthroporphyrin Monocations

Monoprotonated phenanthroporphyrin cations **PhPa**H^+^, **PhPb**H^+^, and **PhPc**H^+^ have similar energies and retain strongly aromatic properties (Table 12). *N*-methyl monocations favor **MePhPb**H^+^ over **MePhPa**H^+^, primarily due to the arrangement better enabling hydrogen bonding interactions (Table 13). All of these monocations exhibit strongly aromatic properties (Tables 12 and 13). The related dications (Table 14) also exhibit strongly diatropic properties even though these structures have crowded cavities that result in substantial distortion (Table S2 and Figures S121 and S123).

**Table 13:**
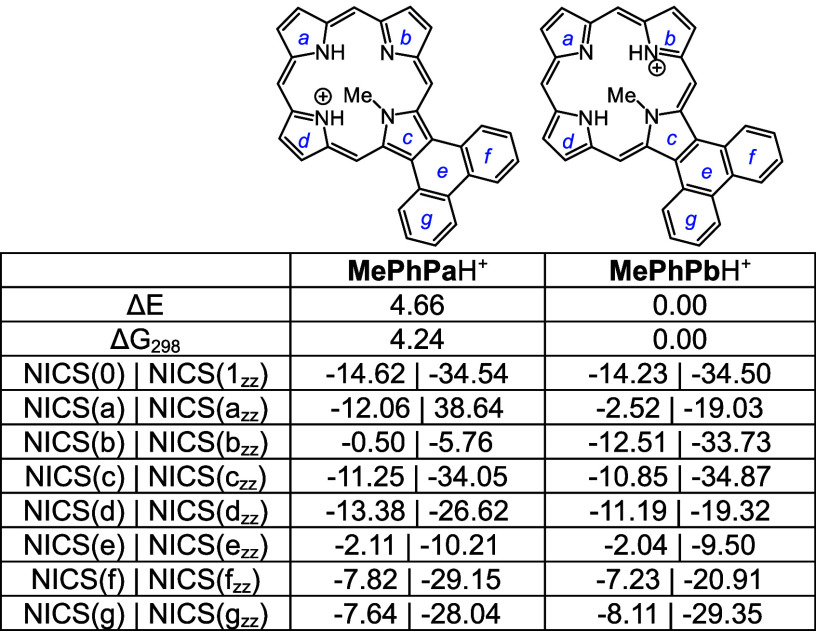
Calculated Relative Energies and NICS Values for *N*-Methylphenanthroporphyrin Monocations

**Table 14:**
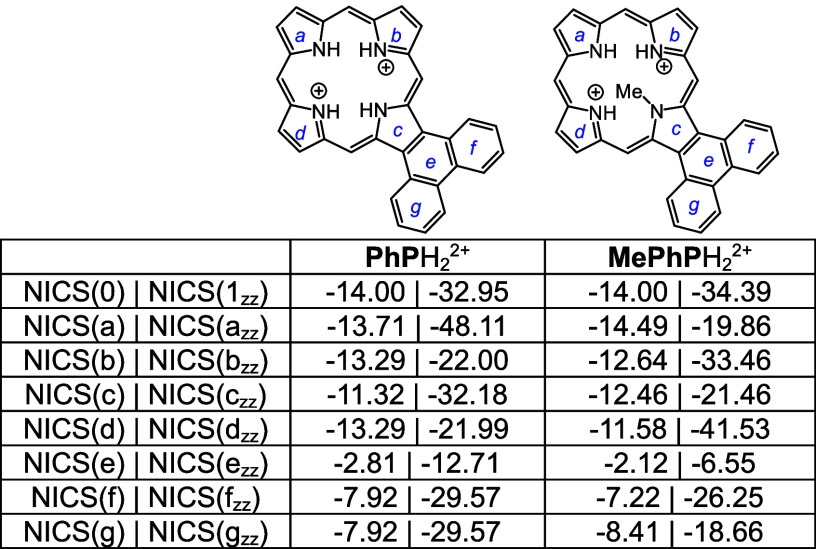
Calculated NICS values for Phenanthroporphyrin Dications

**Figure 11:**
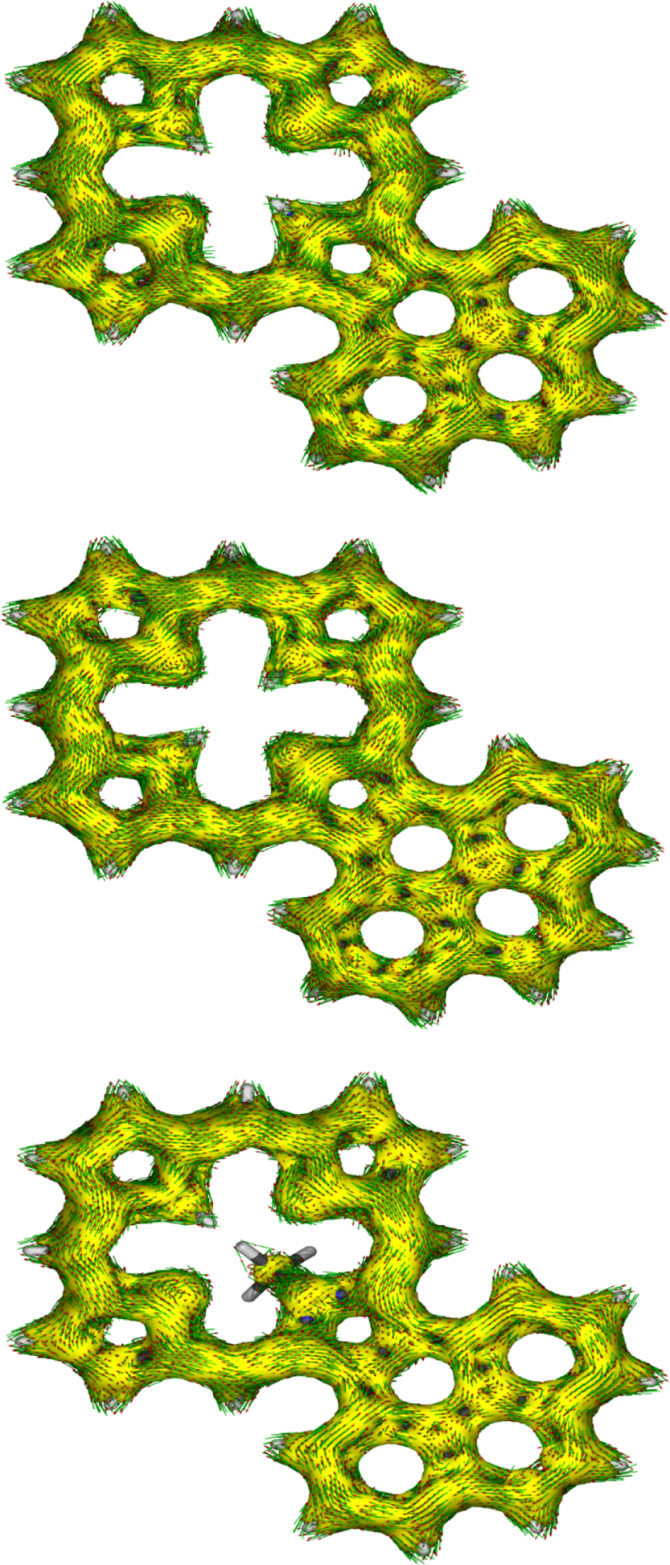
AICD plots (isovalues 0.05) of pyrenoporphyrin structures **PyPa** (top), **PyPb** (middle) and **MePyPa**. Extension of the aromatic circuits through the fused pyrene rings is particularly clear in **PhPa** and **MePhPa**.

Pyrenoporphyrins (**PyP**s) are also very aromatic and two tautomers **PyPa** and **PyPb** with opposite N-Hs are favored by >8 kcal/mol (Table 15) compared to adjacent N–H tautomers **PyPc** and **PyPd**. However, the two *opp*-isomers only differ in energy by <1 kcal/mol. *N*-methylpyrenoporphyrin **PyPa** is favored over **PyPb** due in part to more favorable hydrogen bonding interactions (Table 16). The presence of the internal methyl groups results in severe distortion to these porphyrin structures (Table S3 and Figure S129), but the NICS values indicate virtually no change in their diatropicity. AICD plots demonstrated that the aromatic ring current in **PyPa** favors a 30π electron circuit that passes around the outside of the fused pyrene subunit and a similar pathway can be seen in the related *N*-methyl structure **MePyPa** (Figure 11). The aromatic pathways in tautomer **PyPb** are mixed and show a significant porphyrin-type 18π electron pathway, together with some interactions with the pyrene moiety.

**Table 15:**
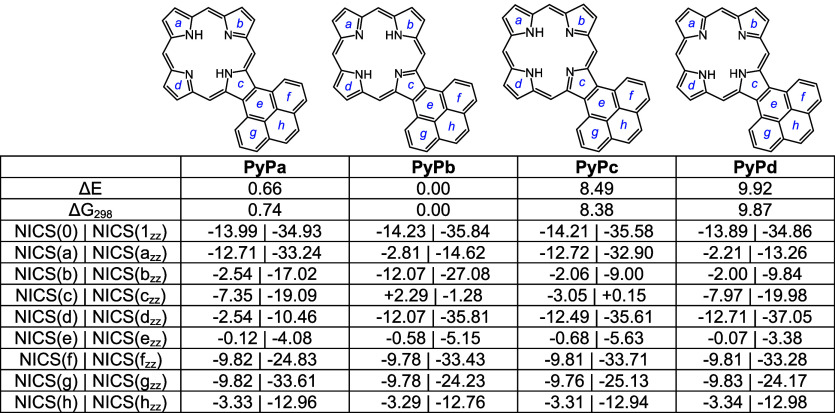
Calculated Relative Energies and NICS Values for Selected Pyrenoporphyrin Tautomers

**Table 16:**
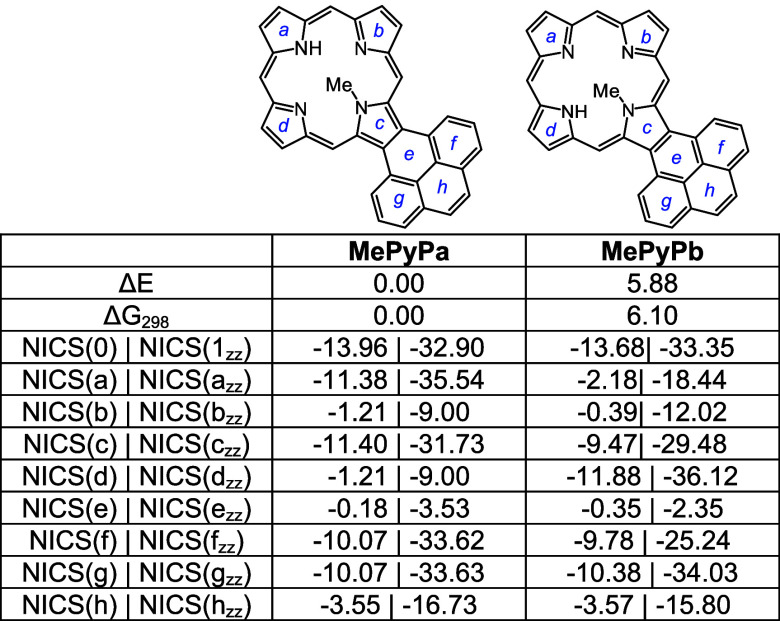
Calculated Relative Energies and NICS Values for *N*-Methylpyrenoporphyrins

Monoprotonation can give rise to three tautomers, **PyPa**H^+^, **PyPb**H^+^ and **PyPc**H^+^, that have similar energies; all three exhibit strongly aromatic properties (Table 17). Two *N*-methyl monocations were considered, and as had been seen for the phenanthrene series, tautomer **MePyPb**H^+^ is favored (Table 18). Finally, although highly distorted (Table S3, Figures S137 and S139), diprotonated species **PyP**H_2_^2+^ and **MePyP**H_2_^2+^ also show strongly aromatic properties, the loss of planarity may be balanced out by the ability of these conjugated structures to delocalize the positive charges (Table )19.

**Table 17:**
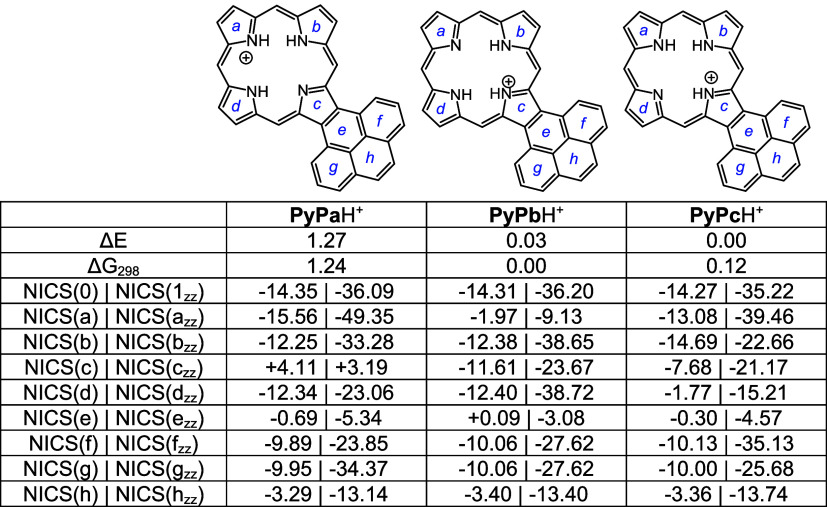
Calculated Relative Energies and NICS Values for Selected Pyrenoporphyrin Monocations

**Table 18:**
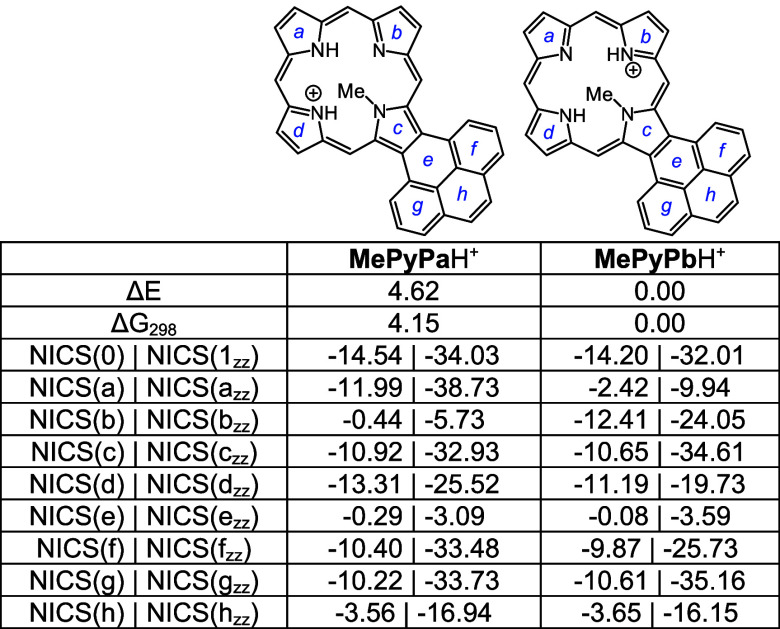
Calculated Relative Energies and NICS Values for Selected Pyrenoporphyrin Monocations

**Table 19:**
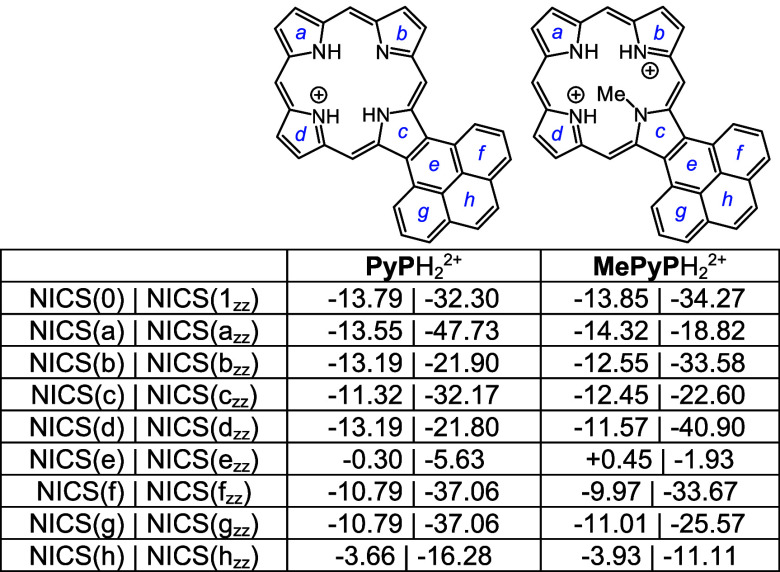
NICS Values for Pyrenoporphyrin Dications

The AICD plots for protonated phenanthro- and pyrenoporphyrins also demonstrate the presence of extended aromatic pathways, and this appears to be an important feature for these structures. Hence, the conjugation pathways present in PAH-fused porphyrins are determined by the individual tautomers and the degree of protonation.

## Conclusions

*N*-methyl *c*-annulated pyrroles were prepared and condensed with acetoxymethylpyrroles to generate tripyrranes with fused acenaphthylene, phenanthrene, and pyrene units. Following cleavage of the terminal ester protective groups, acid catalyzed MacDonald-type “3 + 1” condensations with a pyrrole dialdehyde gave *N*-methyl acenaphtho-, phenanthro-, and pyrenoporphyrins. The presence of internal alkyl substituents distorts the porphyrin macrocycles, but these structures retain porphyrin-type UV–vis spectra. Analysis of the proton NMR spectra shows that the diamagnetic ring currents are slightly reduced and downfield shifts to the protons attached to the benzenoid fragments indicate that the aromatic delocalization pathways extend around the periphery of these subunits. DFT studies demonstrated the loss of planarity in *N*-methyl annulated porphyrins, but the calculated NICS values are consistent with the retention of strongly aromatic characteristics. AICD plots show that in some cases 30π electron circuits are present that involve fused phenanthrene or pyrene rings, although fused acenaphthylene units give far smaller interactions. Overall, this study demonstrates that conjugation pathways vary significantly for different tautomers of *b*-annulated porphyrins and this effect persists in *N*-methyl fused ring porphyrins.

## Experimental Section

Spectrophotometric titrations were performed by adding 0.1% TFA-CH_2_Cl_2_ or 1% TFA-CH_2_Cl_2_ using a microliter syringe directly into a cuvette containing 2.00 mL of the porphyrin solution. NMR spectra were recorded using a 400 or 500 MHz NMR spectrometer and were run at 302 K unless otherwise indicated. ^1^H NMR values are reported as chemical shifts δ, relative integral, multiplicity (s, singlet; d, doublet; t, triplet; q, quartet; p, pentet; m, multiplet; br, broad peak), and coupling constant (*J*). Chemical shifts are reported in parts per million (ppm) relative to CDCl_3_ (^1^H residual CHCl_3_ singlet δ 7.26 ppm, ^13^C CDCl_3_ triplet δ 77.23 ppm), and coupling constants were taken directly from the spectra. Structural assignments were made with additional information from gCOSY, gHSQC, and NOE difference NMR experiments Mass spectral data were acquired using positive-mode electrospray ionization (ESI^+^) and a high-resolution time-of- flight mass spectrometer. ^1^H and ^13^C{^1^H} NMR spectra for all new compounds are reported in the Supporting Information.
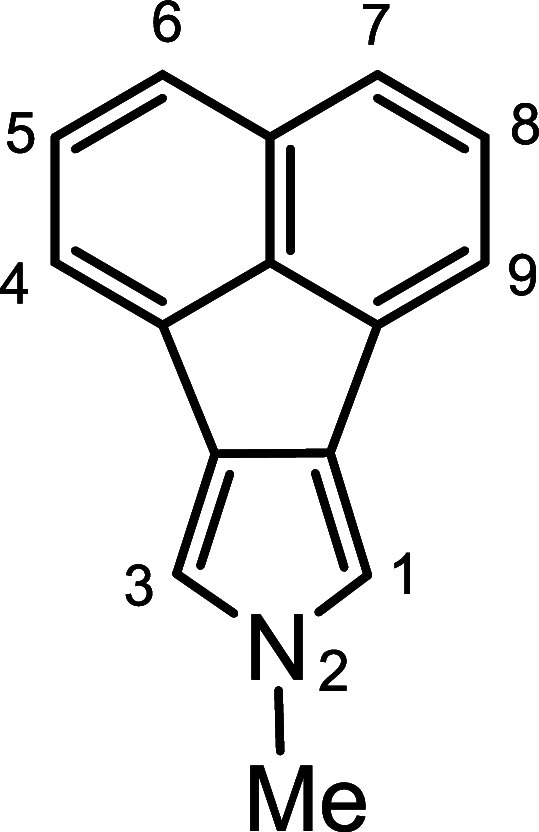


### 2-Methylacenaphtho[1,2-*c*]pyrrole (**11a**)

Potassium hydroxide pellets (4.0 g, 72 mmol) were added to DMSO (20 mL) in a 50 mL round-bottom flask, and the stirred mixture gently heated in an oil bath until the pellets had dissolved. After the mixture had cooled to room temperature, ethyl acenaphtho[1,2-*c*]pyrrole-1-carboxylate^14^ (2.00 g, 7.60 mmol) was added and stirring was continued for 1 h. Methyl iodide (4.0 g, 28 mmol) was added dropwise while maintaining the reaction temperature below 30 °C with the aid of an ice–water bath. The resulting mixture was stirred at room temperature for 1 h and then poured into 150 mL of ice–water. The product was extracted with dichloromethane (3 × 60 mL) and the combined organic layers were then washed with water (100 mL) and 5% sodium bicarbonate solution (100 mL). The organic solution was dried over sodium sulfate, and the solvent evaporated under reduced pressure to give a mixture of methyl and ethyl ester of 2-methylacenaphtho[1,2-*c*]pyrrole-2-carboxylate **10a/b** (1.89 g) in a ratio of approximately 77:23 ethyl ester: methyl ester. ^1^H NMR (CDCl_3_, 500 MHz, major ester product only): δ 8.12 (d, 1H, *J* = 6.9 Hz), 7.74 (d, 1H, *J* = 8.2 Hz), 7.68 (d, 1H, *J* = 8.1 Hz), 7.59–7.55 (m, 2H), 7.50 (dd, 1H, *J* = 6.9, 8.1 Hz) 6.97 (s, 1H), 4.51 (q, 2H, *J* = 7.1 Hz), 3.99 (s, 3H), 1.56 (t, 3H, *J* = 7.1 Hz). The foregoing product was mixed with ethylene glycol (80 mL), potassium hydroxide (4.5 g), 20 drops of hydrazine were added, and nitrogen was bubbled through the mixture for 5 min. The stirred mixture was heated at reflux for 1 h. The hot mixture was poured into ice–water (250 mL) and the resulting precipitate collected by suction filtration to give the acenaphthopyrrole **11a** (1.39 g, 6.8 mmol, 89%) as an off-white solid, mp 107.5–109 °C. ^1^H NMR (CDCl_3_, 500 MHz): δ 7.58 (d, 2H, *J* = 8.1 Hz, 6.7-H), 7.51 (d, 2H, *J* = 6.9 Hz, 4,9-H), 7.45 (dd, 2H, *J* = 6.9, 9.1 Hz, 5,8-H), 6.82 (2H, s, 1,3-H), 3.72 (s, 3H, N-Me). ^13^C{H} NMR (CDCl_3_, 125 MHz): δ 137.3, 134.2, 131.0, 129.0, 127.7 (5,8-CH), 124.0 (6,7-CH), 118.4 (4,9-CH), 114.9 (1,3-CH), 36.8 (N-Me). HRMS (ESI) *m*/*z*: [M + H]^+^ calcd for C_15_H_12_N 206.0964, found 206.0968.
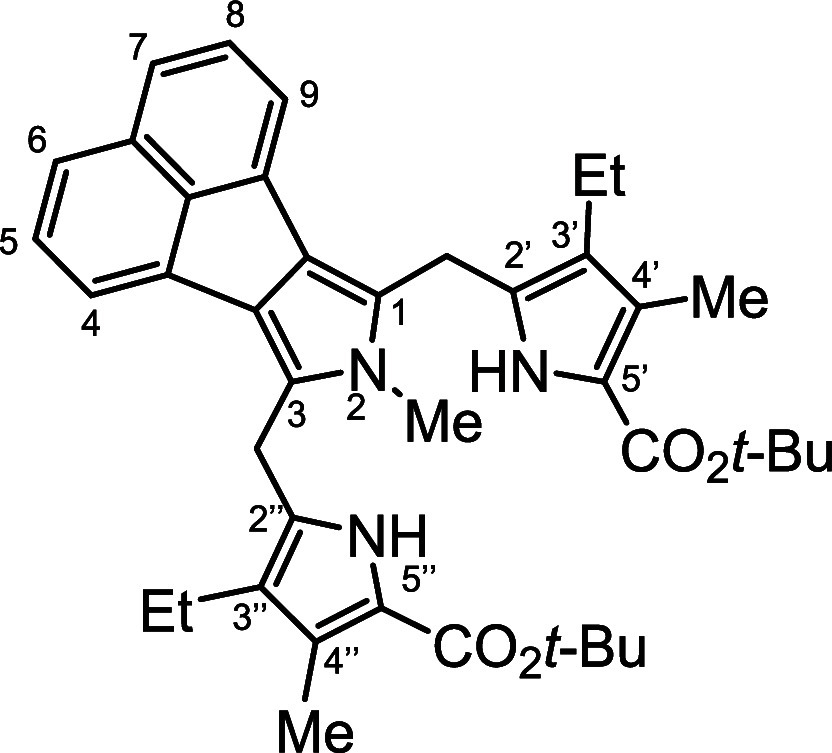


### 1,3-Bis(5-*tert*-butoxycarbonyl-3-ethyl-4-methyl-2-pyrrolylmethyl)acenaphtho[1,2-*c*]pyrrole (**13**)

Nitrogen was bubbled through a mixture of acetoxymethylpyrrole **12**(23) (2.88 g, 10.2 mmol) and *N*-methylacenaphthopyrrole **11a** (1.00 g, 4.88 mmol) in 2-propanol (30 mL) and acetic acid (1.5 mL) for 10 min. This stirred mixture was refluxed under nitrogen overnight using an oil bath as the heat source. After cooling to room temperature and then in an ice bath, the precipitate was suction filtered, washed with 2-propanol, and dried in vacuo to give the tripyrrane (2.85 g, 4.40 mmol, 90%) as an off-white solid, mp 228–229 °C. ^1^H NMR (CDCl_3_, 500 MHz): δ 8.74 (br s, 2H, 2 x NH), 7.55 (d, 2H, *J* = 8.2 Hz, 6,7-H), 7.39 (dd, 2H, *J* = 6.9, 8.2 Hz, 5,8-H), 7.15 (d, 2H, *J* = 6.9 Hz, 4,9-H), 4.16 (s, 4H, 2 x bridge-CH_2_), 3.27 (s, 3H, N-Me), 2.48 (q, 4H, *J* = 7.5 Hz, 2 x pyrrole-CH_2_), 2.28 (s, 6H, 2 x pyrrole-Me), 1.44 (s, 18H, 2 x O-*t*Bu), 1.07 (t, 6H, *J* = 7.5 Hz, 2 x CH_2_C*H*_3_). ^13^C{H} NMR (CDCl_3_, 125 MHz): δ 161.4, 137.7, 134.0, 131.2, 128.1, 127.8 (5,8-CH), 126.4, 126.2, 124.0, 123.8 (6,7-CH), 122.6, 119.3, 117.9 (4,9-CH), 80.4, 30.9 (N-Me), 28.6 (2(*t*-Bu), 24.0 (2 x bridge-CH_2_), 17.5 (2 x pyrrole-CH_2_), 15.7 (2 x CH_2_*C*H_3_), 10.6 (2 x pyrrole-Me). HRMS (ESI) *m*/*z*: M^+^ calcd for C_41_H_49_N_3_O_4_ 647.3723, found 647.3694.
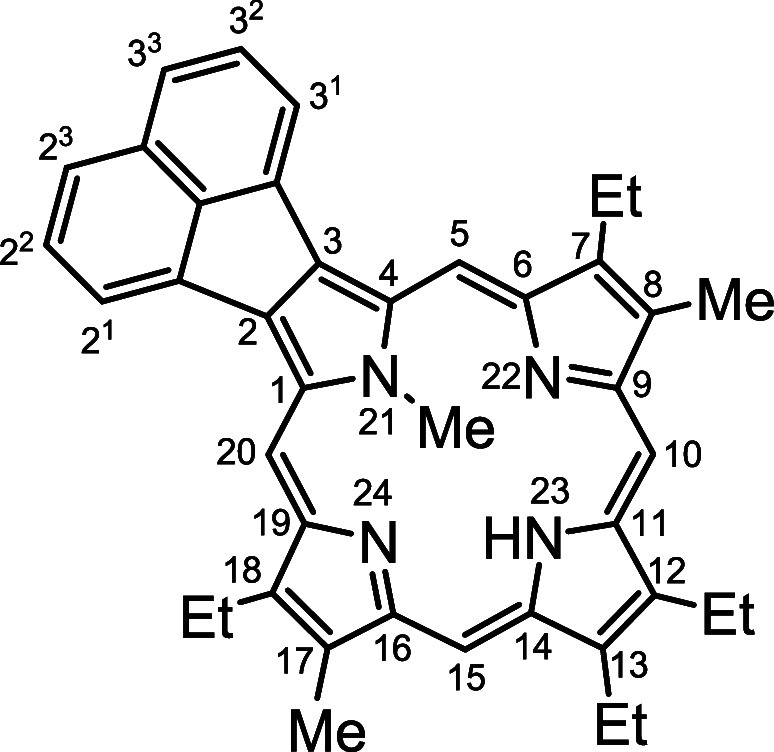


### 7,12,13,18-Tetraethyl-8,17,21-trimethylacenaphtho[1,2-*b*]porphyrin (**6**)

Acenaphthotripyrrane **13** (50.0 mg, 0.077 mmol) was stirred with TFA (1 mL) in a pear-shaped flask under nitrogen for 10 min. The mixture was diluted with dichloromethane (50 mL), pyrrole dialdehyde **14**(24) (13.8 mg, 0.077 mmol) was added, and stirring under nitrogen was continued for 16 h. The mixture was shaken vigorously with 0.15% w/v ferric chloride solution for 6–7 min. The organic layer was then washed with water, 5% aqueous sodium bicarbonate solution and water, and the solvent was removed under reduced pressure. The residue was purified by column chromatography on grade 3 alumina, eluting initially with dichloromethane and then chloroform. The product eluted as a red-brown solution that appeared to be green on the column. Evaporation of the solvent gave a residue that was recrystallized from chloroform–methanol to give the acenaphthoporphyrin (22.7 mg, 0.039 mmol, 51%) as dark crystals, mp >260 °C. UV–vis (1% Et_3_N–CH_2_Cl_2_) λ_max_/nm (log ε): 392 (4.82), 438 (4.81), 465 (5.01), 536 (4.16), 570 (sh, 3.70), 620 (3.73), 648 (sh, 3.42), 677 (4.46). UV–vis (2 equiv TFA-CH_2_Cl_2_) λ_max_/nm (log ε): 263 (4.22), 307 (4.29), 418 (sh, 4.86)443 (5.19), 582 (4.15), 623 (4.50). UV–vis (5% TFA-CH_2_Cl_2_) λ_max_/nm (log ε): 363 (sh, 4.54), 441 (5.02), 570 (sh, 4.01), 585 (4.12), 641 (4.47). ^1^H NMR (CDCl_3_, 50 °C, 500 MHz): δ 10.21 (s, 2H, 5,20-H), 9.66 (s, 2H, 10,15-H), 8.55 (d, 2H, *J* = 6.8 Hz, 2^1^,3^1^-H), 7.83 (d, 2H, *J* = 8.0 Hz, 2^3^,3^3^-H), 7.76 (t, 2H, *J* = 7.5 Hz, 2^2^,3^2^-H), 4.10–3.90 (m, 8H, 4 x C*H*_2_CH_3_), 3.42 (s, 6H, 8,17-Me), 1.93–1.88 (2 overlapping triplets, 12H, 4 x CH_2_C*H*_3_), −2.20 (br s, 1H, NH), −3.93 (s, 3H, 21-Me). ^13^C{H} NMR (CDCl_3_, 50 °C, 125 MHz): δ 157.5, 146.2, 143.5, 140.0, 138.6, 137.8, 137.5, 134.2, 131.8, 130.9, 128.5 (2^2^,3^2^-CH), 127.1 (2^3^,3^3^-CH), 123.8 (2^1^,3^1^-CH), 104.9 (5,20-CH), 96.5 (10,15-CH), 33.4 (N-Me), 20.3, 19.8 (4 x *C*H_2_CH_3_), 18.2, 17.9 (4 x CH_2_*C*H_3_), 11.6 (8,17-Me). ^1^H NMR (TFA-CDCl_3_, 500 MHz): δ 11.10 (s, 2H, 5,20-H), 10.72 (s, 2H, 10,15-H), 8.91 (d, 2H, *J* = 7.1 Hz, 2^1^,3^1^-H), 8.24 (d, 2H, *J* = 8.1 Hz, 2^3^,3^3^-H), 8.03 (dd, 2H, *J* = 7.1, 8.1 Hz, 2^2^,3^2^-H), 4.30–4.17 (m, 8H, 4 x C*H*_2_CH_3_), 3.77 (s, 6H, 8,17-Me), 1.91 (t, 6H, *J* = 7.7 Hz), 1.90 (t, 6H, *J* = 7.7 Hz) (4 x CH_2_C*H*_3_), −3.21 (br s, 2H, 2 x NH), −5.11 (s, 3H, 21-Me). ^13^C{H} NMR (TFA-CDCl_3_, 125 MHz): δ 149.1, 147.1, 146.5, 143.9, 142.6, 141.9, 140.6, 137.7, 137.1, 131.8 (2^3^,3^3^-CH), 131.1, 130.0, 129.6 (2^2^,3^2^-CH), 128.3 (2^1^,3^1^-CH), 103.0 (5,20-CH), 100.0 (10,15-H), 30.7 (N-Me), 20.7, 20.1 (4 x *C*H_2_CH_3_), 17.5, 16.7 (4 x CH_2_*C*H_3_), 12.0 (8,17-Me). HRMS (ESI) *m*/*z*: [M + H]^+^ calcd for C_41_H_41_N_4_ 589.3331, found 589.3320.
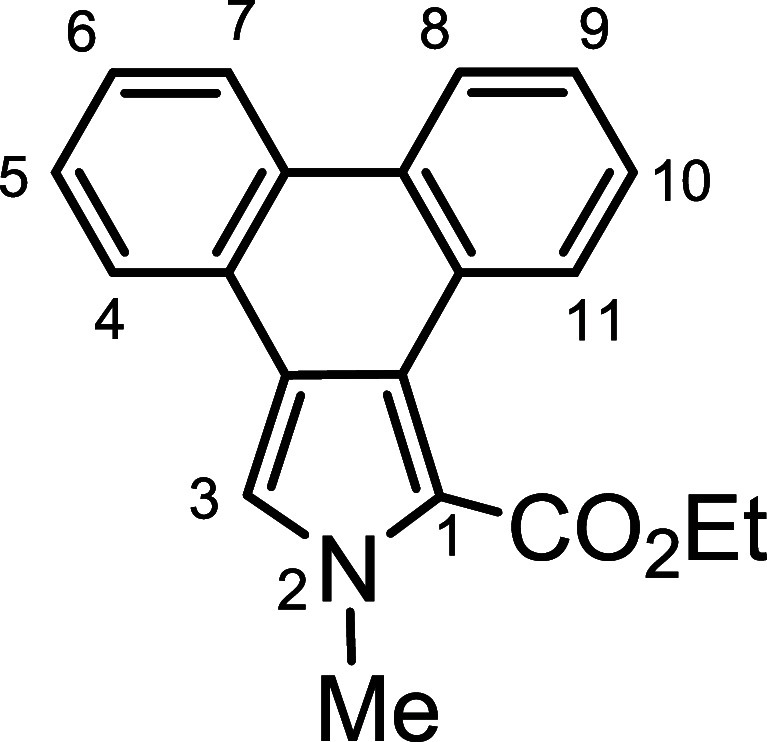


### Ethyl 2-Methylphenanthro[5,6-*c*]pyrrole-1-carboxylate (**16**)

Potassium hydroxide pellets (7.31 g, 130 mmol) were added to DMSO (32 mL) in a 100 mL round-bottom flask, and the stirred mixture was gently heated in an oil bath until the pellets had dissolved. After the mixture had cooled to room temperature, ethyl phenanthro[5,6-*c*]pyrrole-1-carboxylate^15^ (4.00 g, 13.8 mmol) was added and stirring was continued for 1 h. Methyl iodide (10.9 g, 77 mmol) was added dropwise while maintaining the reaction temperature below 30 °C with the aid of an ice–water bath. The resulting mixture was stirred at room temperature for 1 h and then poured into 200 mL of ice–water. The product was extracted with dichloromethane (3 × 100 mL) and the combined organic layers washed with water (100 mL) and 5% sodium bicarbonate solution (100 mL). The organic solution was dried over magnesium sulfate and the solvent evaporated under reduced pressure to give *N*-methylphenanthropyrrole **16** (3.62 g, 11.9 mmol, 86%) as a white solid, mp 124.4–125.6 °C. ^1^H NMR (CDCl_3_, 500 MHz): δ 9.09–9.06 (m, 1H, 11-H), 8.57–8.53 (m, 1H), 8.51–8.49 (m, 1H), 8.04–8.00 (m, 1H), 7.62 (s, 1H), 7.56–7.46 (m, 4H), 4.54 (q, 2H, *J* = 7.1 Hz, OCH_2_), 4.13 (s, 3H, N-Me), 1.50 (t, 3H, *J* = 7.1 Hz, CH_2_C*H*_3_). ^13^C{H} NMR (CDCl_3_, 125 MHz): δ 163.1, 130.7, 128.9, 127.6, 127.4, 127.2, 126.7, 126.4, 125.7, 123.9, 123.64, 123.60, 122.8, 121.4, 120.2, 118.0, 61.0, 39.4, 14.6. HRMS (ESI) *m*/*z*: [M + H]^+^ calcd for C_20_H_18_NO_2_ 304.1332, found 304.1323.
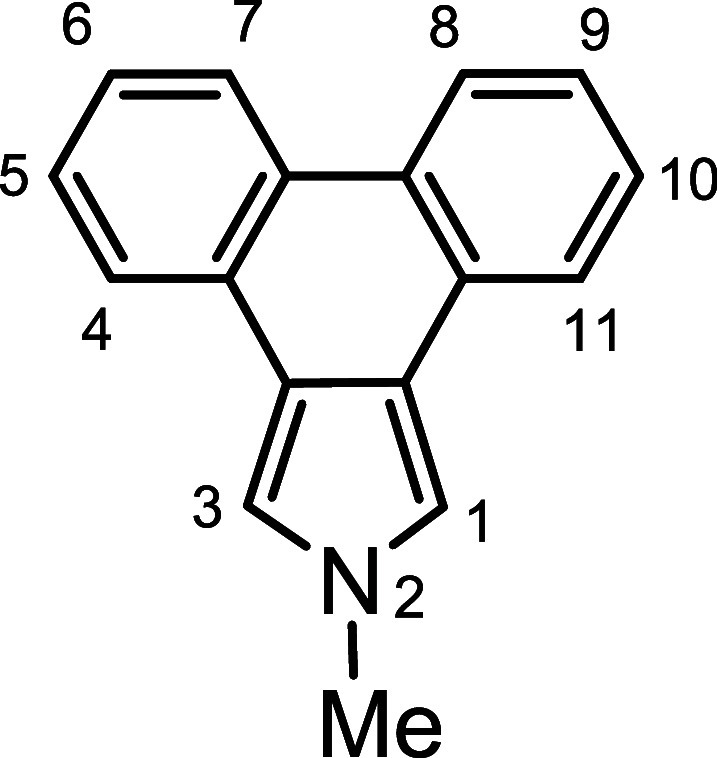


### 2-Methylphenanthro[5,6-*c*]pyrrole (**17a**)

Phenanthropyrrole ester **16** (4.90 g, 16.2 mmol) was taken up in ethylene glycol (200 mL), potassium hydroxide (10.0 g) and 50 drops of hydrazine were added, and nitrogen was bubbled through the mixture for 5 min. Using an oil bath, the stirred mixture was heated under reflux for 1 h. The hot mixture was poured into ice–water (1 L) and the resulting precipitate collected by suction filtration to give the title pyrrole (2.29 g, 9.9 mmol, 61%) as an off-white solid, mp 144.1–146.3 °C. ^1^H NMR (CDCl_3_, 500 MHz): δ 8.45 (d, 2H, *J* = 7.9 Hz, 7,8-H), 8.00–7.96 (m, 2H, 4,11-H), 7.48–7.41 (m, 4H, 5,6,9,10-H), 7.40 (s, 2H, 1,3-H), 3.98 (s, N-Me). ^13^C{H} NMR (CDCl_3_, 125 MHz): δ 128.8, 127.0 (5,10-CH), 124.9 (6,9-CH), 123.7 (7,8-CH), 123.3 (4,11-CH), 120.3, 114.4 (1,3-CH), 37.4 (N-Me). HRMS (ESI) *m*/*z*: [M + H]^+^ calcd for C_17_H_14_N 232.1121, found 232.1116.
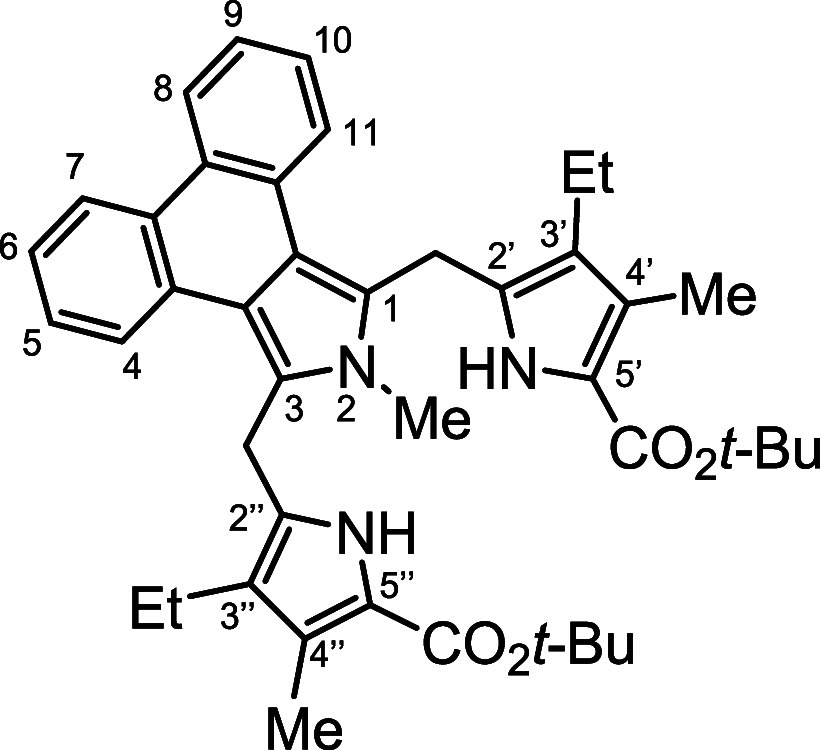


### 1,3-Bis(5-*tert*-butoxycarbonyl-3-ethyl-4-methyl-2-pyrrolylmethyl)phenanthro[5,6-*c*]pyrrole (**21**)

Nitrogen was bubbled through a mixture of acetoxymethylpyrrole **12**(23) (1.48 g, 5.26 mmol) and *N*-methylphenanthropyrrole **17a** (0.502 g, 2.17 mmol) in 2-propanol (40 mL) and acetic acid (2 mL) for 10 min. The stirred mixture was heated on an oil bath and refluxed under nitrogen overnight. After cooling to room temperature and then in an ice bath, the precipitate was suction filtered, washed with 2-propanol, and dried in vacuo to give the tripyrrane (1.296 g, 1.92 mmol, 88%) as an off-white solid, mp 261 °C, dec ^1^H NMR (CDCl_3_, 500 MHz): δ 8.52–8.49 (m, 2H, 7,8-H), 8.33 (br s, 2H, 2 x NH), 8.05–8.02 (m, 2H, 4,11-H), 7.44–7.38 (m, 4H), 4.54 (s, 4H, 2 x bridge-CH_2_), 3.58 (s, 3H, N-Me), 2.56 (q, 4H, *J* = 7.5 Hz, 2 x pyrrole-CH_2_), 2.29 (s, 6H, 2 x pyrrole-Me), 1.41 (s, 18H, 2 x O-*t*Bu), 1.18 (t, 6H, *J* = 7.5 Hz, 2 x CH_2_C*H*_3_). ^13^C{H} NMR (CDCl_3_, 125 MHz): δ 161.4, 129.9, 129.2, 128.0, 127.4, 126.3, 125.0, 124.1 (7,8-CH), 123.6, 123.2 (4,11-CH), 121.1, 119.6, 117.3, 80.4, 30.9 (N-Me), 28.7 (2 x *t*-Bu), 24.9 (2 x bridge-CH_2_), 17.6 (2 x *C*H_2_CH_3_), 15.5 (2 x CH_2_*C*H_3_), 10.7 (2 x pyrrole-Me). HRMS (ESI) *m*/*z*: M^+^ calcd for C_43_H_51_N_3_O_4_ 673.3880, found 673.3904.
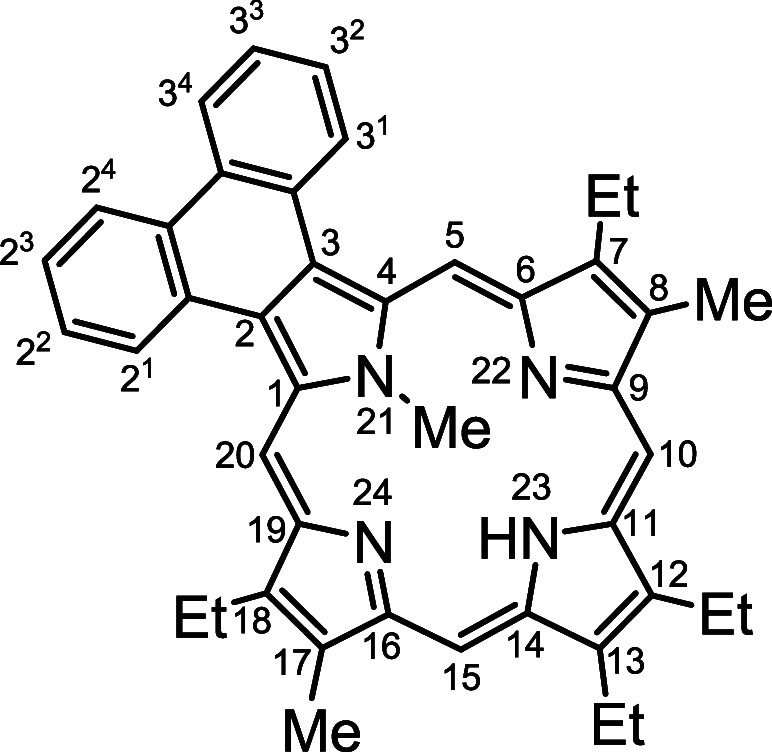


### 7,12,13,18-Tetraethyl-8,17,21-trimethylphenanthro[5.6-*b*]porphyrin (**7**)

Phenanthrotripyrrane **21** (100 mg, 0.148 mmol) was stirred with TFA (2 mL) in a pear shaped flask under nitrogen for 10 min. The mixture was diluted with dichloromethane (19 mL), pyrrole dialdehyde **14**(24) (27 mg, 0.15 mmol) was added, and stirring under nitrogen was continued for 2 h. The mixture was diluted with chloroform and shaken vigorously with 1% w/v ferric chloride solution for 6–7 min. The organic layer was then washed with water, 5% aqueous sodium bicarbonate solution and water, and the solvent was removed under reduced pressure. The residue was purified by column chromatography on grade 3 alumina, eluting with dichloromethane, and the product recrystallized from chloroform–methanol to give the phenanthroporphyrin (12.6 mg, 0.019 mmol, 13%) as dark crystals, mp 296.2–298.0 °C. UV–vis (1% Et_3_N–CH_2_Cl_2_) λ_max_/nm (log ε): 389 (4.62), 435 (5.02), 526 (4.14), 558 (3.65), 608 (3.67), 664 (4.30). UV–vis (2 equiv TFA-CH_2_Cl_2_) λ_max_/nm (log ε): 273 (4.91), 435 (5.08), 576 (sh, 4.02), 608 (4.28). UV–vis (10% TFA-CH_2_Cl_2_) λ_max_/nm (log ε): 341 (4.15), 411 (5.05), 576 (4.04), 632 (4.26). ^1^H NMR (CDCl_3_, 50 °C, 500 MHz): δ 10.77 (s, 2H, 5,20-H), 9.75 (s, 2H, 10,15-H), 9.78 (d, 2H, *J* = 8.2 Hz, 2^1^,3^1^-H), 8.92 (d, 2H, *J* = 8.2 Hz, 2^4^,3^4^-H), 8.01 (t, 2H, *J* = 7.6 Hz, 2^2^,3^2^-H), 7.78 (t, 2H, *J* = 7.5 Hz, 2^3^,3^3^-H), 4.12–4.01 (m, 8H, 4 x C*H*_2_CH_3_), 3.47 (s, 6H, 8,17-Me), 1.95 (t, 6H, *J* = 7.7 Hz), 1.91 (t, 6H, *J* = 7.7 Hz) (4 x CH_2_C*H*_3_), −2.23 (br s, 1H, NH), −4.22 (s, 3H, 21-Me). ^13^C{H} NMR (CDCl_3_, 50 °C, 125 MHz): δ 156.9, 152.3, 146.4, 144.5, 139.8, 138.3, 137.3, 132.1, 129.0, 128.1 (2^2^,3^2^-CH), 127.6 (2^1^,3^1^-CH), 126.4 (2^3^,3^3^-CH), 124.3 (2^4^,3^4^-CH), 119.2, 103.6 (5,20-CH), 96.7 (10,15-CH), 33.8 (N-Me), 20.5, 19.8 (*C*H_2_CH_3_), 18.2, 17.8 (CH_2_*C*H_3_), 11.7 (8,17-Me). ^1^H NMR (TFA-CDCl_3_, 500 MHz): δ 11.30 (s, 2H, 5,20-H), 10.55 (s, 2H, 10,15-H), 9.47 (d, 2H, *J* = 8.1 Hz, 2^1^,3^1^-H), 9.07 (d, 2H, *J* = 8.2 Hz, 2^4^,3^4^-H), 8.16 (t, 2H, *J* = 7.6 Hz, 2^2^,3^2^-H), 8.01 (t, 2H, *J* = 7.5 Hz, 2^3^,3^3^-H), 4.24–4.06 (m, 8H, 4 x C*H*_2_CH_3_), 3.67 (s, 6H, 8,17-Me), 1.86 (t, 6H, *J* = 7.7 Hz), 1.80 (t, 6H, *J* = 7.7 Hz) (4 x CH_2_C*H*_3_), −2.26 (br s, 2H, 2 x NH), −4.91 (s, 3H, 21-Me). ^13^C{H} NMR (TFA-CDCl_3_, 125 MHz): δ 151.5, 146.3, 145.5, 144.3, 142.6, 141.7, 139.6, 133.8, 129.6 (2^2^,3^2^-CH), 129.4 (2^3^,3^3^-CH), 127.8 (2^1^,3^1^-CH), 125.9, 124.9 (2^4^,3^4^-CH), 122.0, 101.5 (5,20-CH), 99.2 (10,15-CH), 30.9 (N-Me), 20.7, 20.0 (*C*H_2_CH_3_), 17.5, 16.6 (CH_2_*C*H_3_), 11.7 (8,17-Me). HRMS (ESI) *m*/*z*: [M + H]^+^ calcd for C_43_H_43_N_4_ 615.3488, found 615.3492.
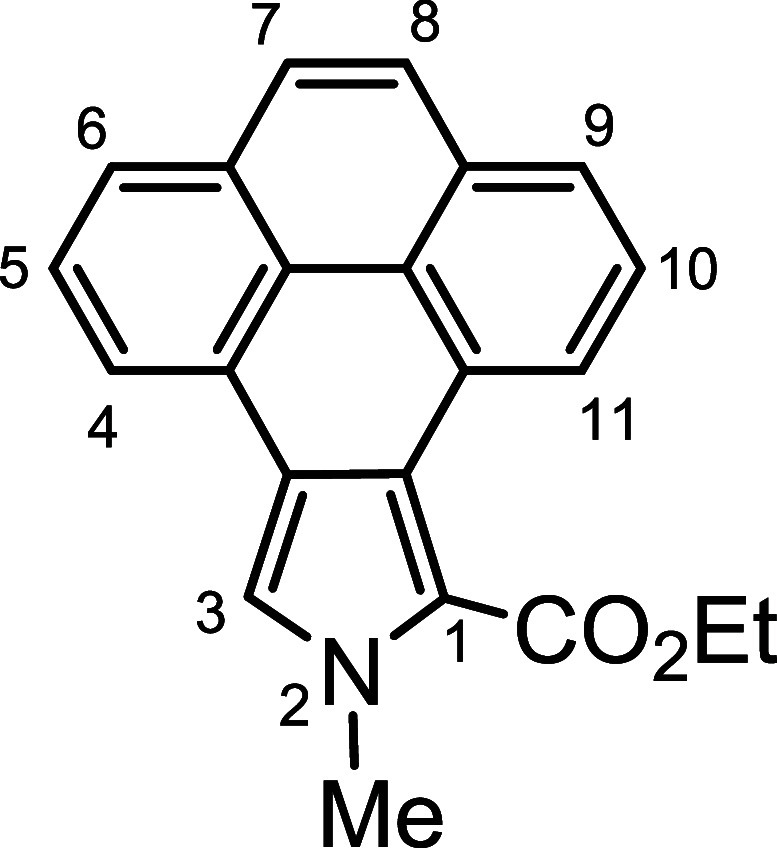


### Ethyl 2-Methylpyreno[4,5-*c*]pyrrole-1-carboxylate (**19**)

Potassium hydroxide pellets (1.8 g, 32 mmol) were added to DMSO (10 mL) in a 50 mL round-bottom flask and the stirred mixture gently heated on an oil bath until the pellets had dissolved. After the mixture had cooled to room temperature, ethyl pyreno[4,5-*c*]pyrrole-1-carboxylate^17^ (0.949 g, 3.03 mmol) was added and stirring was continued for 1 h. Methyl iodide (2.65 g, 18.6 mmol) was added dropwise while maintaining the reaction temperature below 30 °C with the aid of an ice–water bath. The resulting mixture was stirred at room temperature for 1 h and then poured into 200 mL of ice–water. The product was extracted with dichloromethane (3 × 100 mL) and the combined organic layers were washed with water (100 mL) and 5% sodium bicarbonate solution (100 mL). The organic solution was dried over magnesium sulfate and the solvent evaporated under reduced pressure to give *N*-methylpyrenopyrrole **19** (0.930 g, 2.84 mmol, 94%) as an off-white powder, mp 187.2–189.3 °C. ^1^H NMR (CDCl_3_, 50 °C, 500 MHz): δ 9.44 (dd, 1H, *J* = 1.2, 7.9 Hz, 11-H), 8.23 (dd, 1H, *J* = 1.2, 7.5 Hz, 4- or 6-H), 8.05 (dd, 1H, *J* = 1.1, 7.7 Hz, 9-H), 7.975 (dd, 1H, *J* = 1.1, 7.7 Hz, 4- or 6-H), 7.970 (d, 1H, *J* = 8.8 Hz), 7.93 (d, 1H, *J* = 8.8 Hz) (7,8-H), 7.90 (t, 1H, *J* = 7.8 Hz, 10-H), 7.84 (t, 1H, *J* = 7.7 Hz, 5-H), 7.74 (s, 1H, 3-H), 4.59 (q, 2H, *J* = 7.1 Hz, OCH_2_), 4.15 (s, 3H, N-Me), 1.55 (t, 3H, *J* = 7.1 Hz, CH_2_C*H*_3_). ^13^C{H} NMR (CDCl_3_, 50 °C, 125 MHz): δ 163.1, 132.3, 132.1, 128.2, 126.99, 126.97, 126.93, 126.14, 126.04, 125.8 (10-CH), 125.7, 124.8 (11-CH), 124.5, 124.4, 123.7, 122.0 (3-CH), 120.6, 119.2, 118.7, 61.0 (OCH_2_), 31.5 (N-Me), 14.6 (CH_2_*C*H_3_). HRMS (ESI) *m*/*z*: M^+^ calcd for C_22_H_17_NO_2_ 327.1259, found 327.1259.
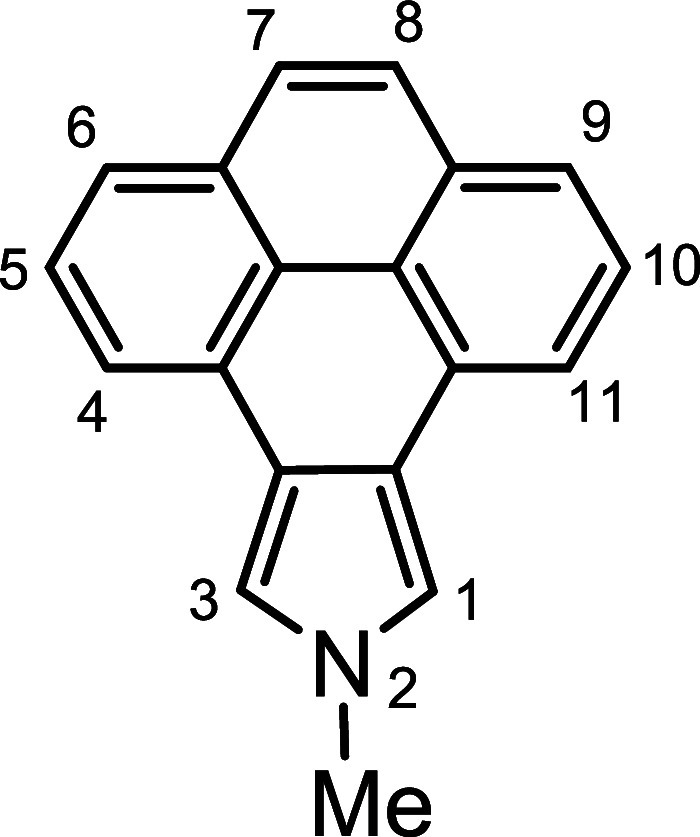


### 2-Methylpyreno[4,5-*c*]pyrrole (**20a**)

Pyrenopyrrole ester **19** (0.505 g, 1.54 mmol) was taken up in ethylene glycol (20 mL), potassium hydroxide (1.04 g), 20 drops of hydrazine were added, and nitrogen was bubbled through the mixture for 5 min. The stirred mixture was heated under reflux on an oil bath for 1 h. The hot mixture was poured into ice–water (1 L) and the resulting precipitate collected by suction filtration and dried in vacuo to give the *N*-methylpyrenopyrrole (0.370 g, 1.45 mmol, 94%) as a white powder, mp 190.0–191.1 °C. ^1^H NMR (CDCl_3_, 500 MHz): δ 8.23 (dd, 2H, *J* = 1.2, 7.5 Hz, 6,9-H), 7.94 (dd, 2H, *J* = 1.2, 7.7 Hz, 4,11-H), 7.93 (s, 2H, 7,8-H), 7.83 (t, 2H, *J* = 7.6 Hz, 5,10-H), 7.58 (s, 2H, 1,3-H), 3.99 (s, 3H, N-Me). ^13^C{H} NMR (CDCl_3_, 125 MHz): δ 132.2, 128.0, 127.6 (7,8-CH), 126.1 (5,10-CH), 124.1 (4,11-CH), 123.9, 120.5, 119.7 (6,9-CH), 115.1 (1,3-CH), 37.5 (N-Me). HRMS (ESI) *m*/*z*: [M + H]^+^ calcd for C_19_H_14_N 256.1121, found 256.1120.
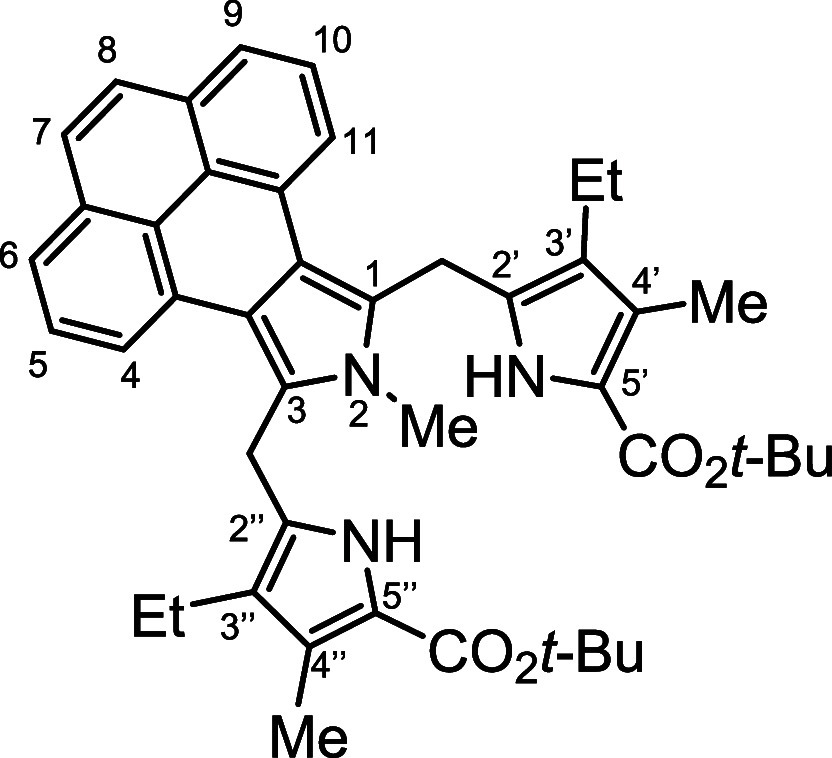


### 1,3-Bis(5-*tert*-butoxycarbonyl-3-ethyl-4-methyl-2-pyrrolylmethyl)pyreno[4,5-*c*]pyrrole (**22**)

Nitrogen was bubbled through a mixture of acetoxymethylpyrrole **12**(23) (1.10 g, 3.9 mmol) and *N*-methylpyrenopyrrole **20a** (0.509 g, 1.99 mmol) in 2-propanol (40 mL) and acetic acid (2 mL) for 10 min. The stirred mixture was refluxed under nitrogen overnight. After cooling to room temperature and then in an ice bath, the precipitate was suction filtered, washed with 2-propanol, and dried in vacuo to give the tripyrrane (1.12 g, 1.60 mmol, 82%) as a pale pink solid, mp >260 °C, dec^1^H NMR (CDCl_3_, 500 MHz): δ 8.33 (br s, 2H, 2 x NH), 8.25 (d, 2H, *J* = 7.7 Hz, 4,11-H), 7.937 (s, 2H, 7,8-H), 7.931 (dd, 2H, *J* = 1.1, 7.7 Hz, 6,9-H), 7.76 (t, 2H, *J* = 7.7 Hz, 5,10-H), 4.62 (s, 4H, 2 x bridge-CH_2_), 3.60 (s, 3H, N-Me), 2.59 (q, 4H, *J* = 7.5 Hz, 2 x pyrrole-CH_2_), 2.29 (s, 6H, 2 x pyrrole-Me), 1.35 (s, 18H, 2 x O-*t*Bu), 1.21 (t, 6H, *J* = 7.5 Hz, 2 x CH_2_C*H*_3_). ^13^C{H} NMR (CDCl_3_, 125 MHz): δ 161.3, 132.3, 128.5, 127.9, 127.7 (7,8-CH), 126.3 (5,10-CH), 126.2, 124.8, 124.4 (6,9-CH), 123.6, 121.7, 119.9 (4,11-CH), 119.4, 117.4, 80.3, 30.8 (N-Me), 28.5 (*t*-Bu), 24.8 (2 x bridge-CH_2_), 17.6 (2 x *C*H_2_CH_3_), 15.7 (2 x CH_2_*C*H_3_), 10.7 (2 x pyrrole-Me). HRMS (ESI) *m*/*z*: M^+^ calcd for C_45_H_51_N_3_O_4_ 697.3880, found 697.3860.
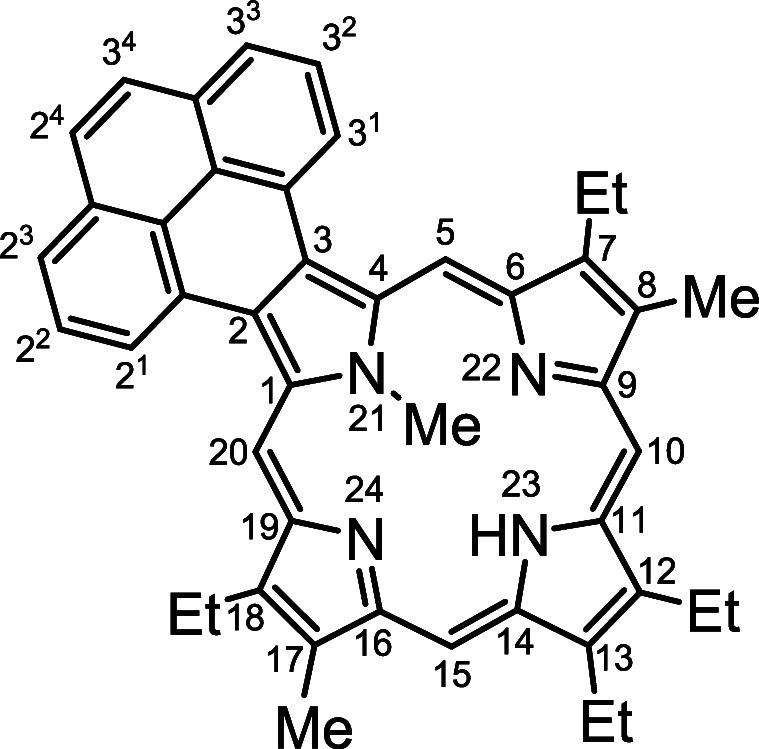


### 7,12,13,18-Tetraethyl-8,17,21-trimethylpyreno[4,5-*b*]porphyrin (**8**)

Pyrenotripyrrane **22** (102.5 mg, 0.147 mmol) was stirred with TFA (2 mL) in a pear-shaped flask under nitrogen for 10 min. The mixture was diluted with dichloromethane (20 mL), pyrrole dialdehyde **14**(24) (25.9 mg, 0.145 mmol) was added, and stirring under nitrogen was continued for 2 h. The mixture was diluted with chloroform and shaken vigorously with 0.1% w/v ferric chloride solution for 6–7 min. The organic layer was then washed with water, 5% aqueous sodium bicarbonate solution and water, and the solvent was removed under reduced pressure. The residue was purified by column chromatography on grade 3 alumina, eluting initially with dichloromethane and then with 1% ethanol-dichloromethane. Recrystallization from chloroform–methanol gave the pyrenoporphyrin (14.7 mg, 0.023 mmol, 16%) as a dark solid, mp >260 °C. UV–vis (1% Et_3_N–CH_2_Cl_2_) λ_max_/nm (log ε): 321 (4.49), 376 (sh, 4.48), 396 (sh, 4.54), 440 (4.90), 527 (4.12), 558 (sh, 3.67), 610 (3.62), 638 (sh, 3.28), 667 (4.26). UV–vis (2 equiv TFA-CH_2_Cl_2_) λ_max_/nm (log ε): 315 (4.41), 390 (sh, 4.51), 428 (4.96), 565 (sh, 3.94), 581 (4.07), 611 (4.25). UV–vis (5% TFA-CH_2_Cl_2_) λ_max_/nm (log ε): 317 (4.42), 406 (4.86), 428 (4.95), 528 (4.05), 586 (4.10), 636 (4.16). ^1^H NMR (CDCl_3_, 55 °C, 500 MHz): δ 10.91 (s, 2H, 5,20-H), 10.01 (d, 2H, *J* = 7.8 Hz, 2^1^,3^1^-H), 9.73 (s, 2H, 10,15-H), 8.37 (t, 2H, *J* = 7.8 Hz, 2^2^,3^2^-H), 8.30 (d, 2H, *J* = 7.6 Hz, 2^3^,3^3^-H), 8.16 (s, 2H, 2^4^,3^4^-H), 4.14–4.00 (m, 8H, 4 x C*H*_2_CH_3_), 3.46 (s, 6H, 8,17-Me), 1.98 (t, 6H, *J* = 7.7 Hz), 1.90 (t, 6H, *J* = 7.7 Hz) (4 x CH_2_C*H*_3_), −4.14 (s, 3H, 21-Me). ^1^H NMR (TFA-CDCl_3_, 500 MHz): δ 11.52 (s, 2H, 5,20-H), 10.62 (s, 2H, 10,15-H), 9.77 (dd, 2H, *J* = 1.5, 7.4 Hz, 2^1^,3^1^-H), 8.58 (dd, 2H, *J* = 1.5, 7.7 Hz, 2^3^,3^3^-H), 8.55 (t, 2H, J = 7.5 Hz, 2^2^,3^2^-H), 8.32 (s, 2H, 2^4^,3^4^-H), 4.34–4.24 (m, 4H), 4.22–4.10 (m, 4H) (4 x C*H*_2_CH_3_), 3.72 (s, 6H, 8,17-Me), 1.90 (t, 6H, *J* = 7.8 Hz), 1.84 (t, 6H, *J* = 7.8 Hz) (4 x CH_2_C*H*_3_), −2.44 (br s, 2H), −4.87 (s, 3H, 21-Me). ^13^C{H} NMR (TFA-CDCl_3_, 125 MHz): δ 152.1, 146.8, 146.1, 144.4, 142.8, 141.8, 140.0, 132.9, 129.0 (2^3^,3^3^-CH), 128.7 (2^4^,3^4^-CH), 128.2 (2^2^,3^2^-CH), 127.5, 125.7 (2^1^,3^1^-CH), 125.1, 123.0, 101.8 (5,20-CH), 99.3 (10,15-CH), 31.0 (N-Me), 20.8, 20.1 (*C*H_2_CH_3_), 17.5, 16.6 (CH_2_*C*H_3_), 12.0 (8,17-Me). HRMS (ESI) *m*/*z*: [M + H]^+^ calcd for C_45_H_43_N_4_ 639.3488, found 639.3459.

### Pyreno[4,5-*c*]pyrrole (**20b**)^17^

NMR data for **20b** was previously reported in DMSO-*d*_6_. In order to make comparisons, spectra of this compound were obtained in CDCl_3_ for the first time. ^1^H NMR

(CDCl_3_, 500 MHz): δ 8.96 (br s, 1H), 8.30 (dd, 2H, *J* = 1.2, 7.5 Hz, 6,9-H), 7.97 (dd, 2H, *J* = 1.2, 7.7 Hz, 4,11-H), 7.95 (s, 2H, 7,8-H), 7.85 (t, 2H, *J* = 7.6 Hz, 5,10-H), 7.74 (d, 2H, *J* = 2.8 Hz, 1,3-H). ^13^C{H} NMR (CDCl_3_, 125 MHz): δ 132.3, 128.2, 127.6, 126.1, 124.4, 124.2, 120.3, 120.0, 111.1.

### Computational Studies

All calculations were performed using the Gaussian 16 revision C.01.^40^ Geometry optimizations were performed using the M06-2X functional and the 6–311++G(d,p) basis set.^41^ Vibrational frequencies were computed to confirm the absence of imaginary frequencies and derive zero-point energy and vibrational entropy corrections from unscaled frequencies. Single point energy calculations were performed on the optimized minima using M06-2X/cc-PVTZ.^42^ NICS values were calculated using the GIAO method^43^ using CAM-B3LYP/6-31+G(d,p) and AICD plots were obtained from CGST calculations using B3LYP/6-31+G(d).^44^ NICS(0) was calculated at the mean position of all four heavy atoms in the middle of the macrocycle. NICS(a), NICS(b), NICS(c), NICS(d), NICS(e), NICS(f), NICS(g), and NICS(h) values were obtained by applying the same method to the mean position of the heavy atoms that comprise the individual rings of each macrocycle. In addition, NICS(1)*_zz_*, NICS(1a)*_zz_*, NICS(1b)*_zz_*, NICS(1c)*_zz_*, NICS(1d)*_zz_*, NICS(1e)*_zz_*, NICS(1f)*_zz_*, NICS(1g)*_zz_*, and NICS(1h)*_zz_* were obtained by applying the same method to ghost atoms placed 1 Å above each of the corresponding NICS(0) points and extracting the zz contribution of the magnetic tensor. The resulting energies, Cartesian coordinates, and AICD plots can be found in the Supporting Information.

## Data Availability

The data underlying this study are available in the published article and its online Supporting Information.

## Supporting Information Available

Selected UV–vis (Figures S1–S17), ^1^H NMR, ^1^H–^1^H COSY, HSQC, DEPT-135, ^13^C{^1^H} NMR (Figures S18–S81), mass spectra (Figures S82–S92), calculated conformations and bond lengths for selected tautomers (Figure S93–S140), calculated dihedral angles (Tables S1–S3), calculated Gibbs free energies for optimized structures (Tables S4–S6), AICD plots (Figures S141–S179) and Cartesian coordinates (Table S7) are provided (PDF)
