# Supplementary material for: Assessment of Conjugation Pathways in N-Methylporphyrins that Are Fused to Acenaphthylene, Phenanthrene, or Pyrene: Evidence for the Presence of Alternative Aromatic Circuits
Source: J Org Chem. 2024 Oct 25;89(22):16493–509. doi: 10.1021/acs.joc.4c01760 (PMC11574857; doi:10.1021/acs.joc.4c01760)
Supplement: Supplementary file 1 — jo4c01760_si_001.pdf [file jo4c01760_si_001.pdf]

Supporting information for

Assessment of Conjugation Pathways in *N*-Methylporphyrins that are Fused to Acenaphthylene, Phenanthrene or Pyrene: Evidence for the Presence of Alternative Aromatic Circuits

Jared S. Salrin,[1] Brian G. Carpenter,[1] Deyaa I. AbuSalim,[1,2,3] and Timothy D. Lash[1]

[1]Department of Chemistry, Illinois State University, Normal, Illinois 61790-4160

[2]Department of Chemistry, Rowan University, Glassboro, New Jersey 08028

[3]STEM Department, Rowan College of South Jersey, Vineland, New Jersey 08360

* Email: tdlash@ilstu.edu

Table of Contents

Page

S2-S10	Selected UV-Vis spectra (Figures S1-S17)
S11-S58	Selected proton, DEPT-135, ^{1}H-^{1}H COSY, HSQC and carbon-13 NMR spectra (Figures S18-S81)
S59-S64	Selected mass spectra (Figures S82-S92)
S65-S112	Calculated conformations and bond lengths of selected structures (Figures S93-S140)
S113-S115	Calculated dihedral angles for annulated porphyrin tautomers and selected monoprotonated and diprotonated species (Tables S1-S3)
S116-S118	Calculated Gibbs free energies for the optimized structures (Tables S4-S6)
S119-S157	AICD plots (Figures S142-S179)
S158-S190	Cartesian Coordinates (Table S7)

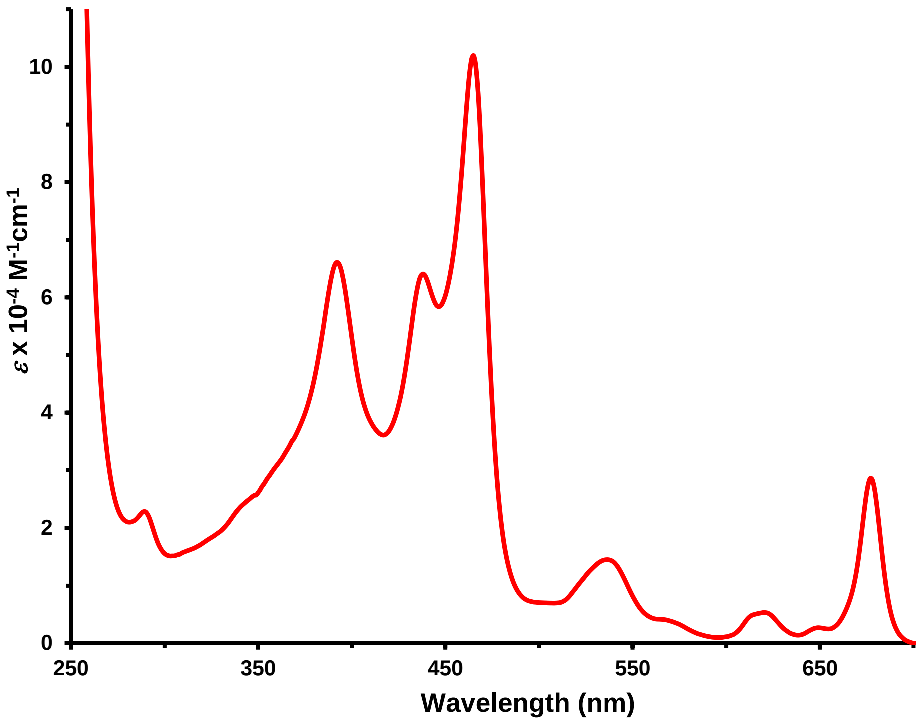

Figure S1. UV-vis spectrum of *N*-methyl acenaphthoporphyrin **6** in 1% Et_3N-CH_2Cl_2.

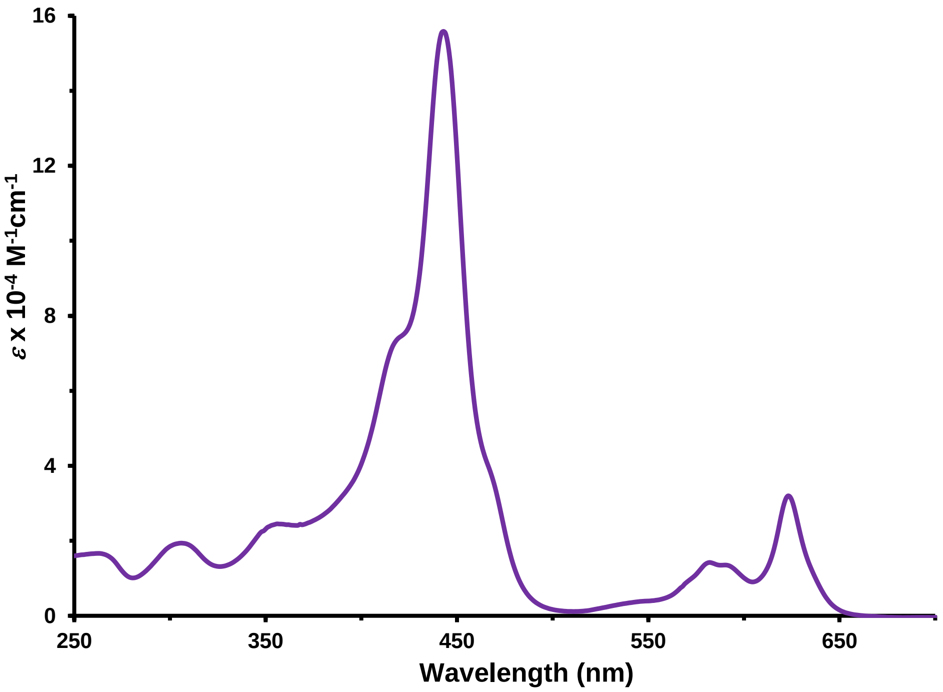

Figure S2. UV-vis spectrum of *N*-methyl acenaphthoporphyrin monocation **6**H^+ in CH_2Cl_2 with 1 equivalent of TFA.

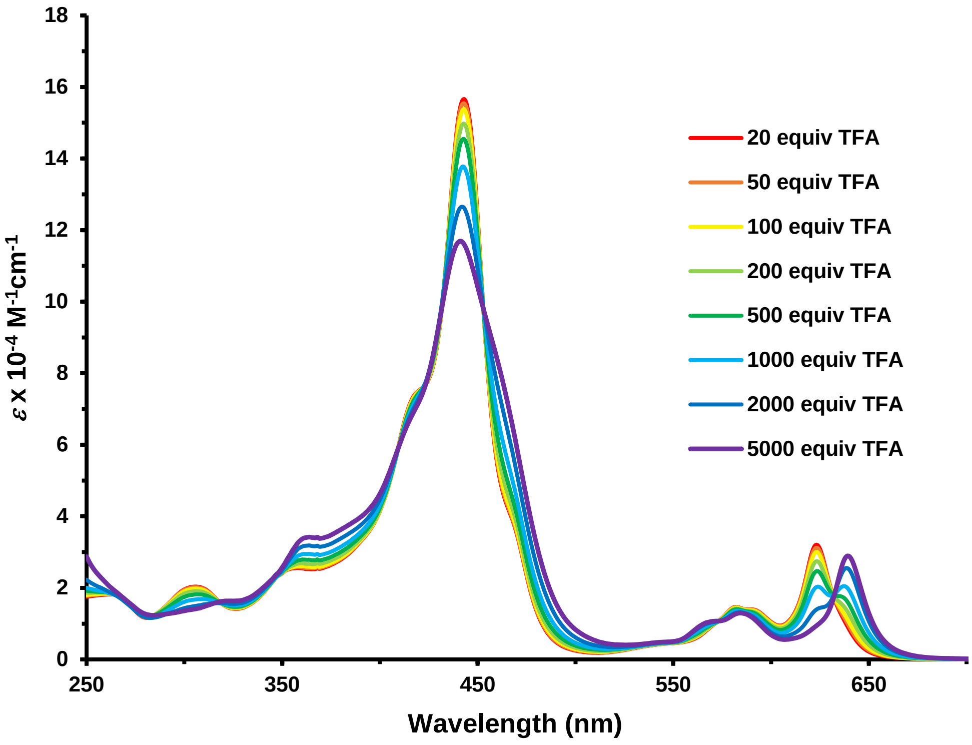

Figure S3. UV-vis spectra of *N*-methyl acenaphthoporphyrin **6** in CH_2Cl_2 with 20-5000 equivalents of TFA.

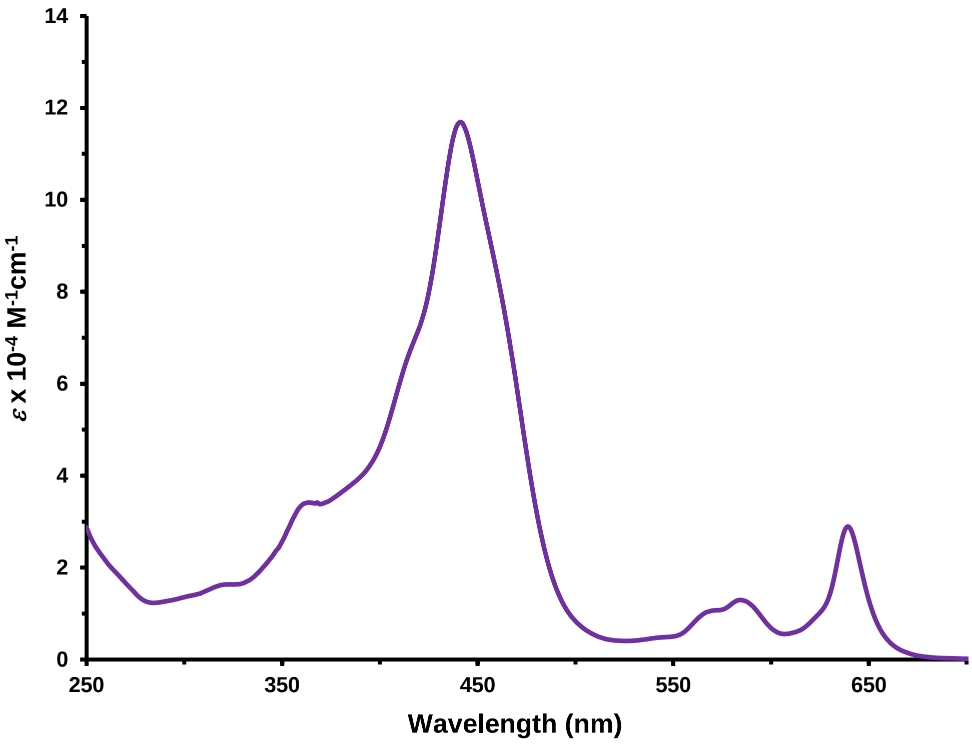

Figure S4. UV-vis spectrum of dication **6**H_2^{2+} in CH_2Cl_2 with 5000 equivalents of TFA.

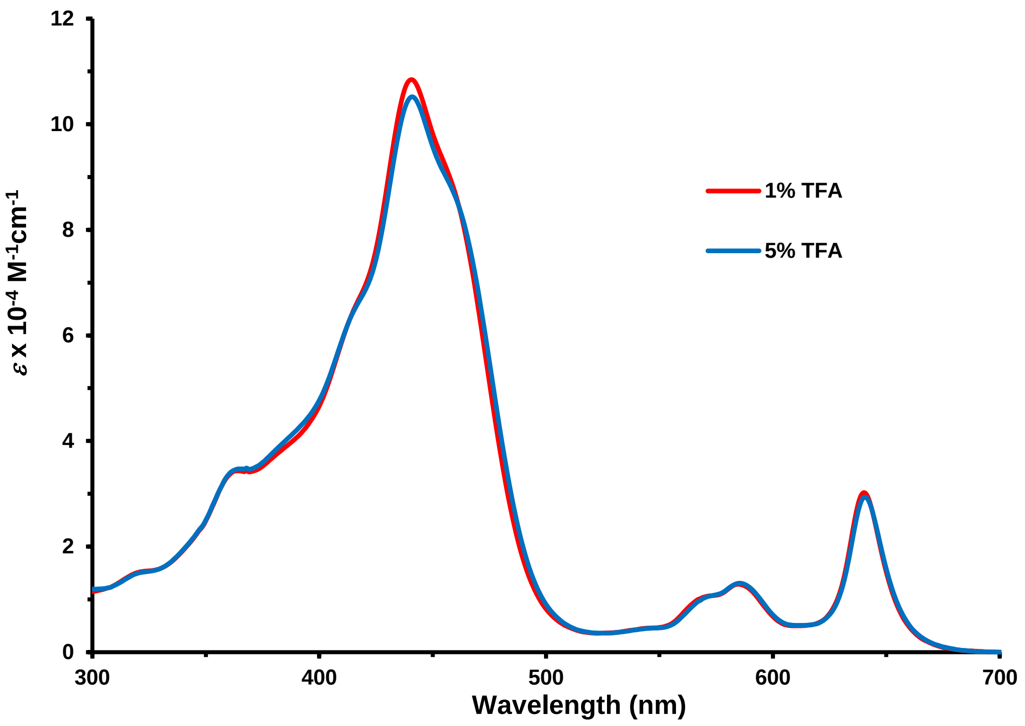

Figure S5. UV-vis spectra of **6**H_2^{2+} in 1% and 5% TFA-CH_2Cl_2.

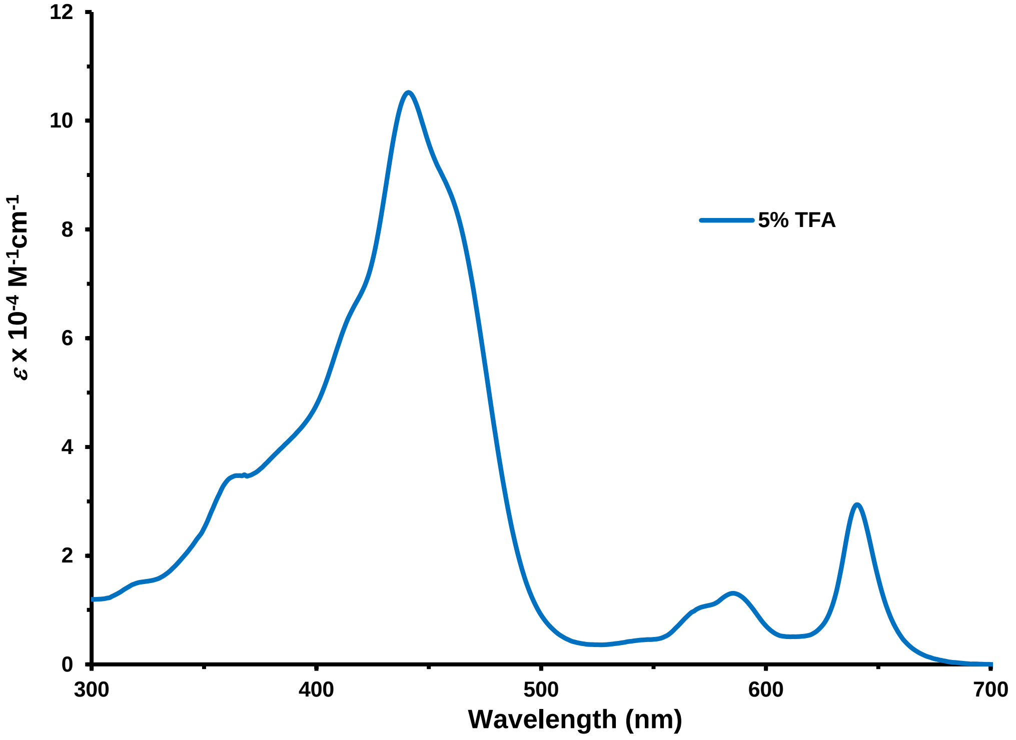

Figure S6. UV-vis spectrum of **6**H_2^{2+} in 5% TFA-CH_2Cl_2.

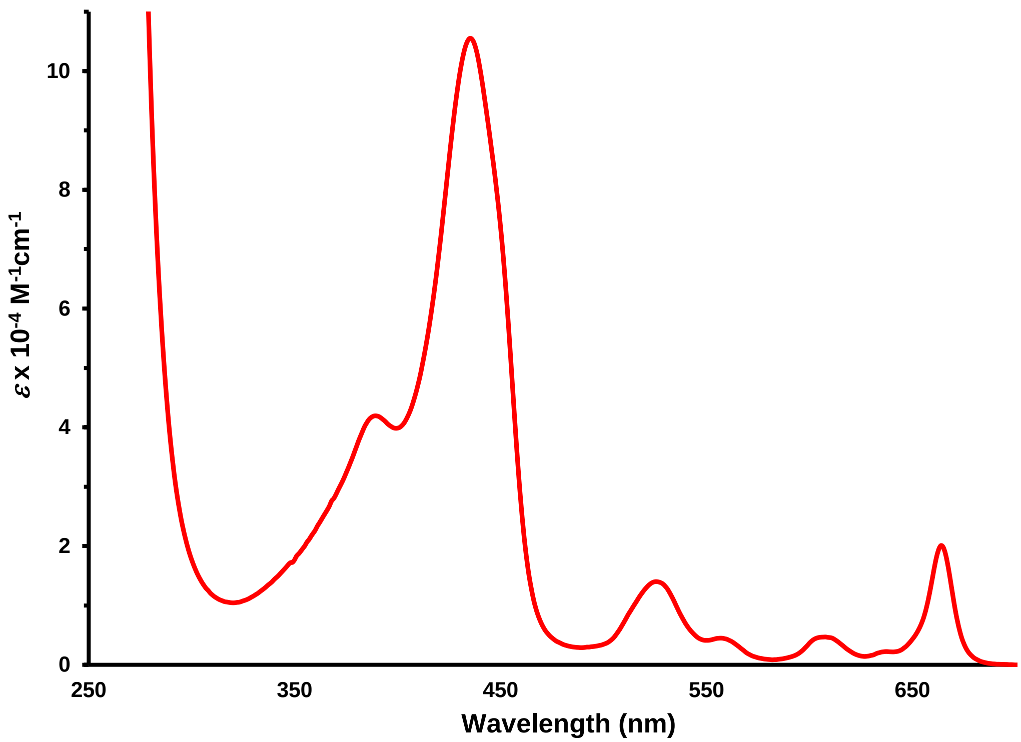

Figure S7. UV-vis spectrum of *N*-methyl phenanthroporphyrin **7** in 1% Et_3N-CH_2Cl_2.

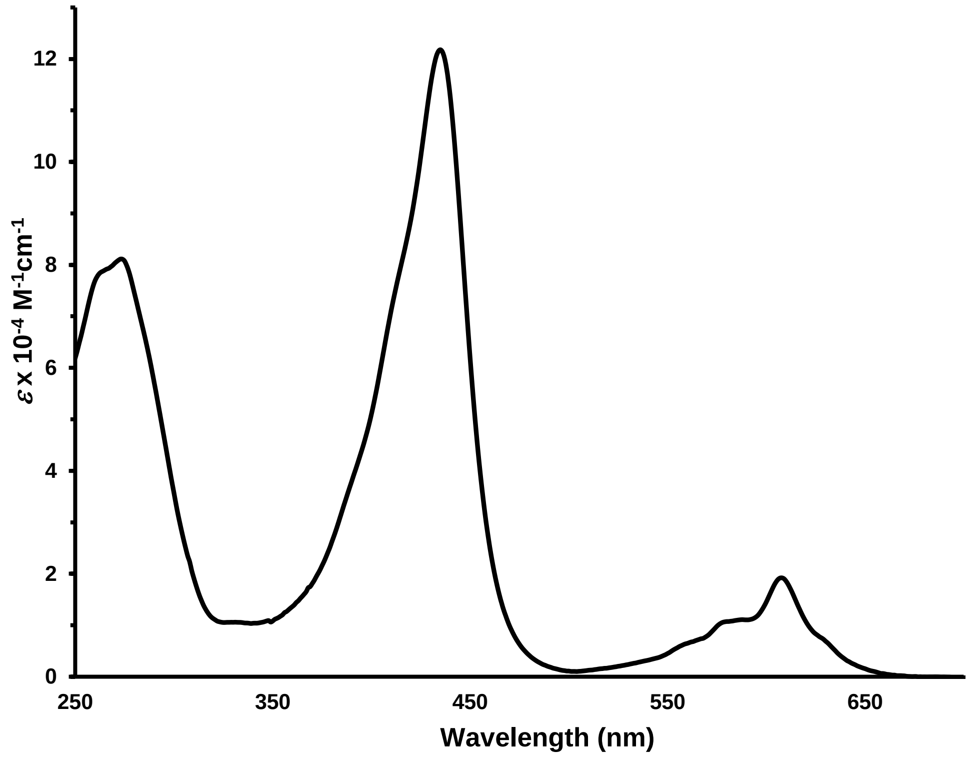

Figure S8. UV-vis spectrum of *N*-methyl phenanthroporphyrin monocation **7**H^+ in CH_2Cl_2 with 2 equivalents of TFA.

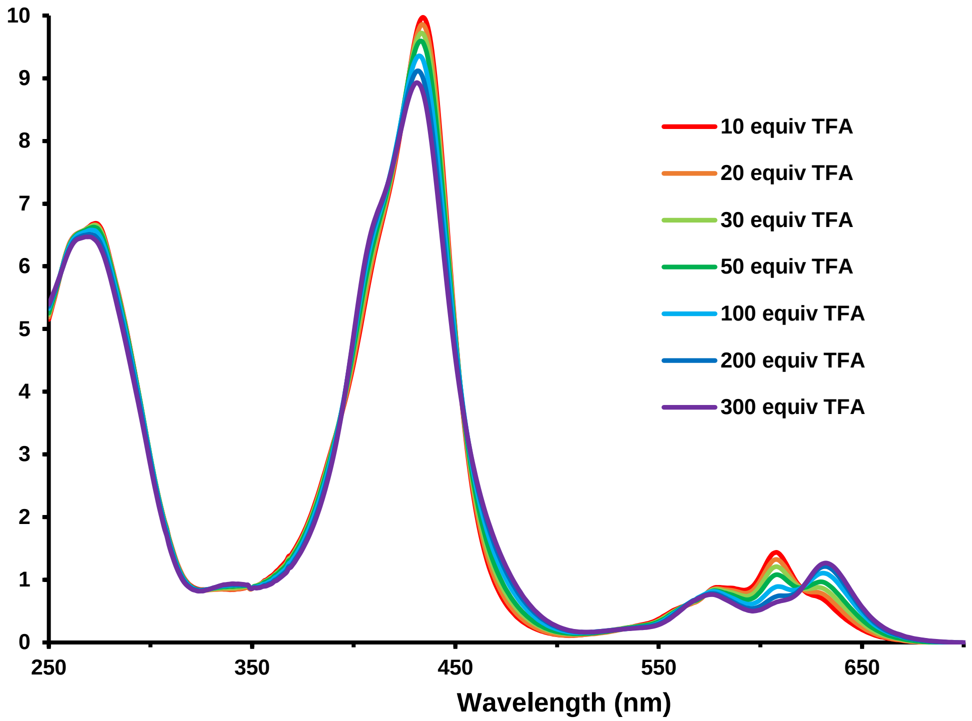

Figure S9. UV-vis spectra of **7** in CH_2Cl_2 with 10-300 equivalents of TFA.

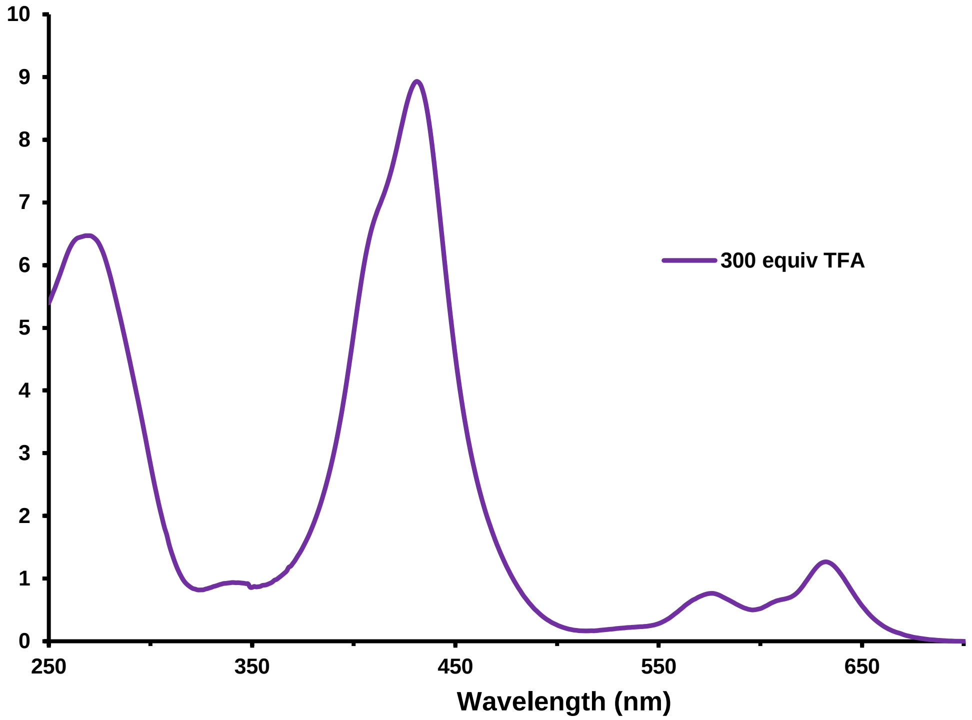

Figure S10. UV-vis spectrum of dication **7**H_2^{2+} in CH_2Cl_2 with 300 equivalents of TFA.

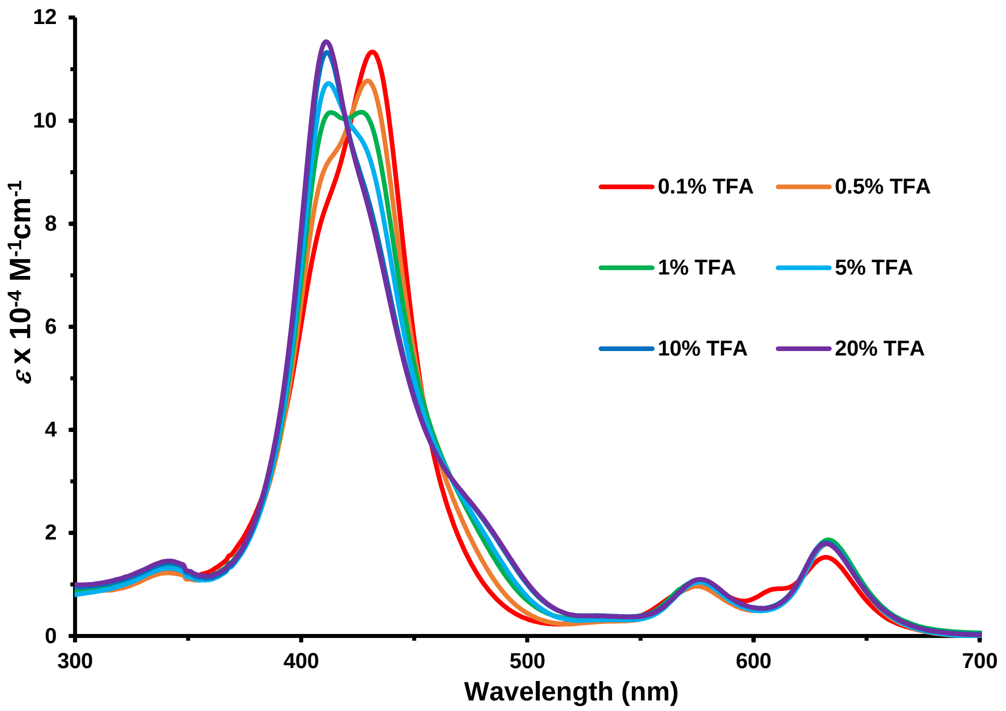

Figure S11. UV-vis spectra of **7** in CH_2Cl_2 with 0.1-20% TFA.

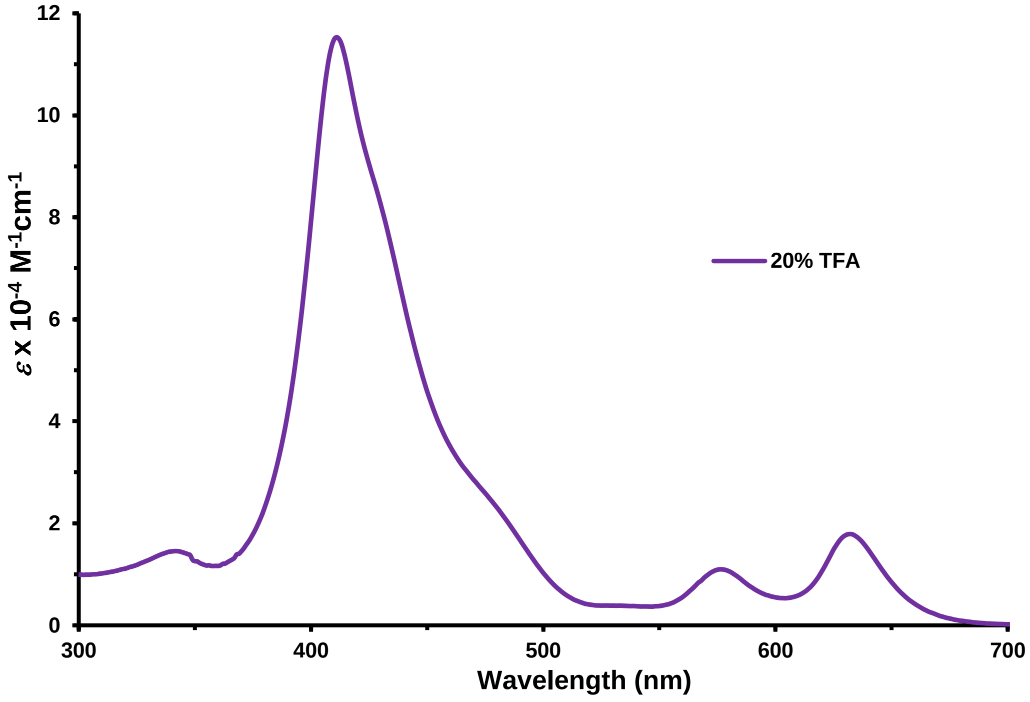

Figure S12. UV-vis spectrum of **7** in 20% TFA-CH_2Cl_2.

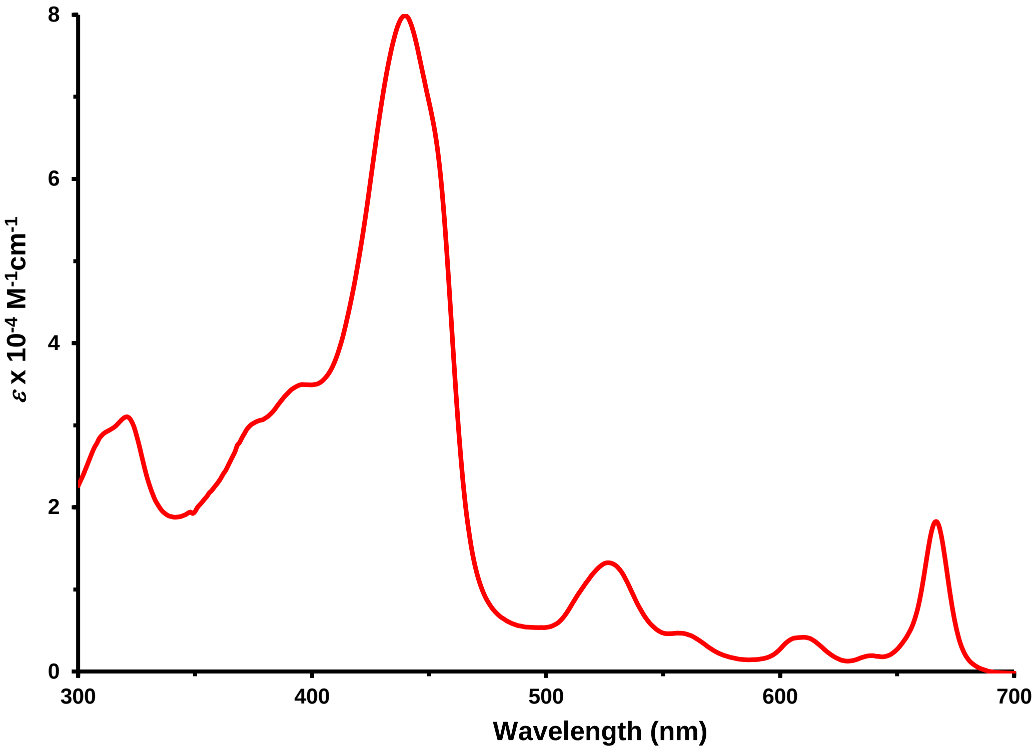

Figure S13. UV-vis spectrum of *N*-methyl pyrenoporphyrin **8** in 1% Et_3N-CH_2Cl_2.

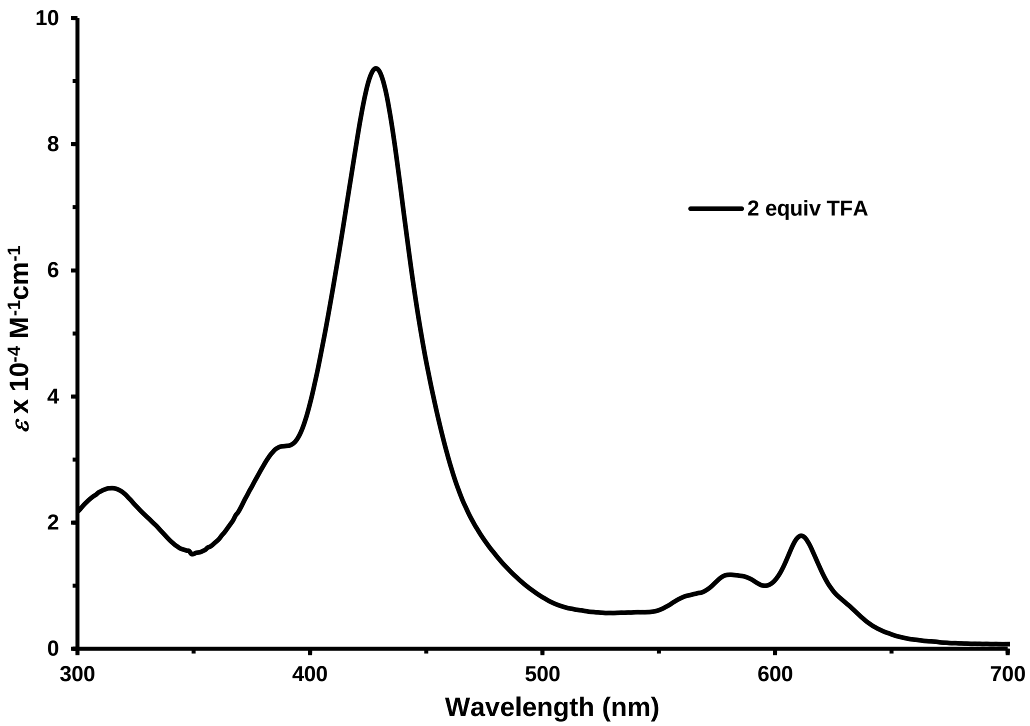

Figure S14. UV-vis spectrum of monocation **8**H^+ in CH_2Cl_2 with 2 equivalents of TFA.

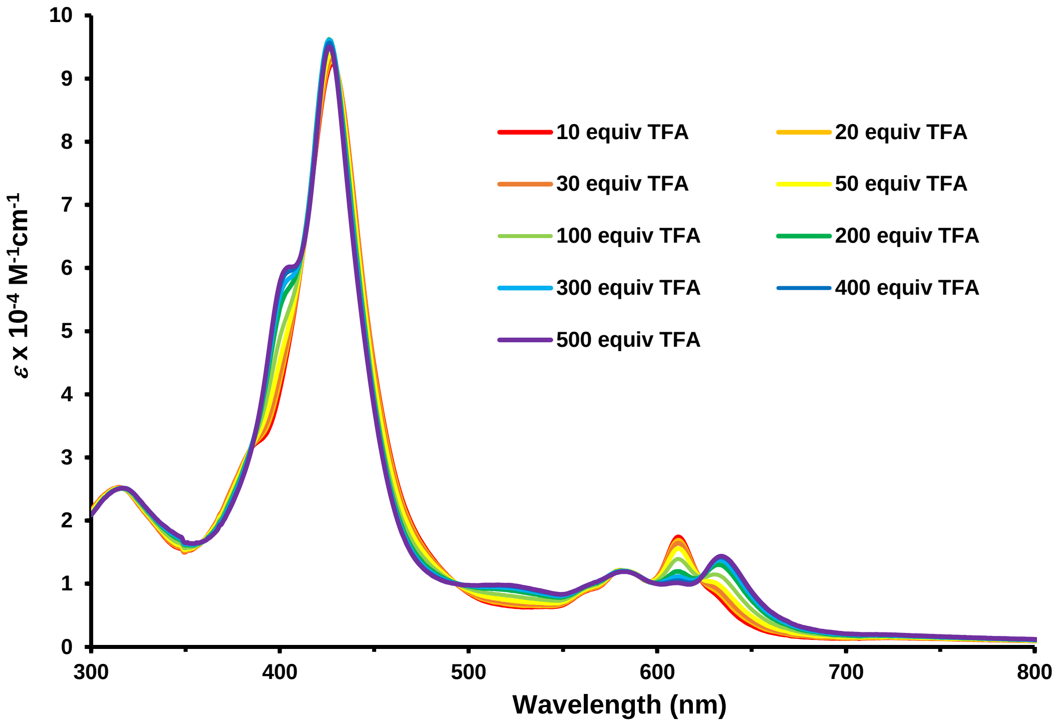

Figure S15. UV-vis spectra of *N*-methyl pyrenoporphyrin **8** in CH_2Cl_2 with 10-500 equivalents of TFA

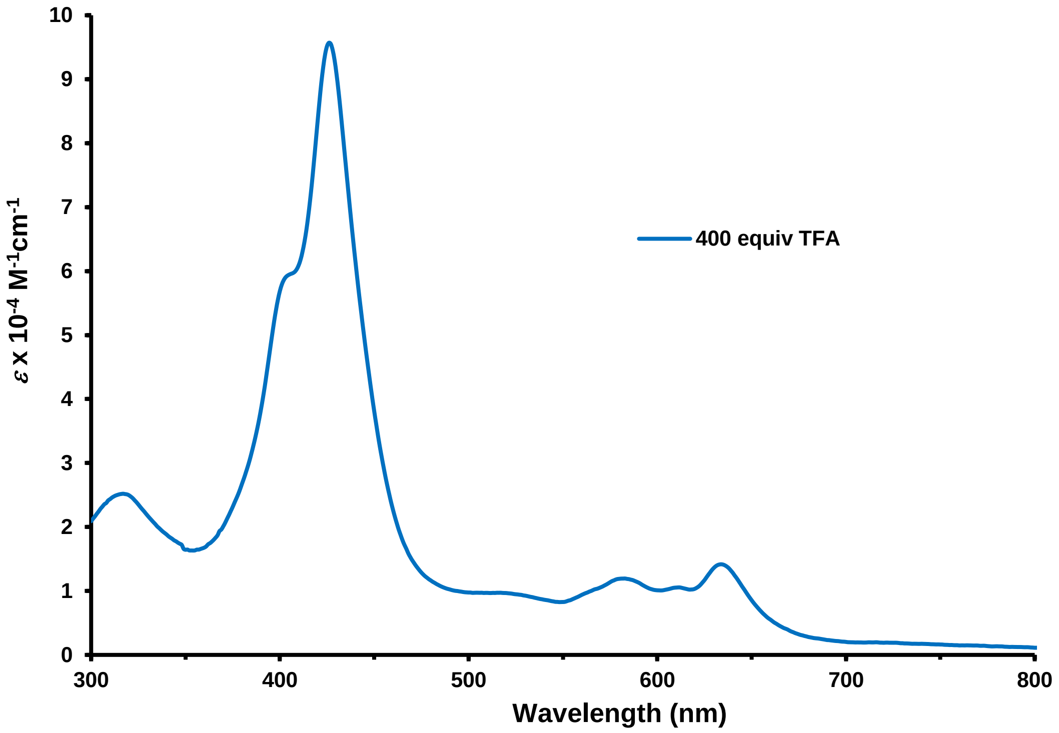

Figure S16. UV-vis spectrum of dication **8**H_2^{2+} in CH_2Cl_2 with 400 equivalents of TFA.

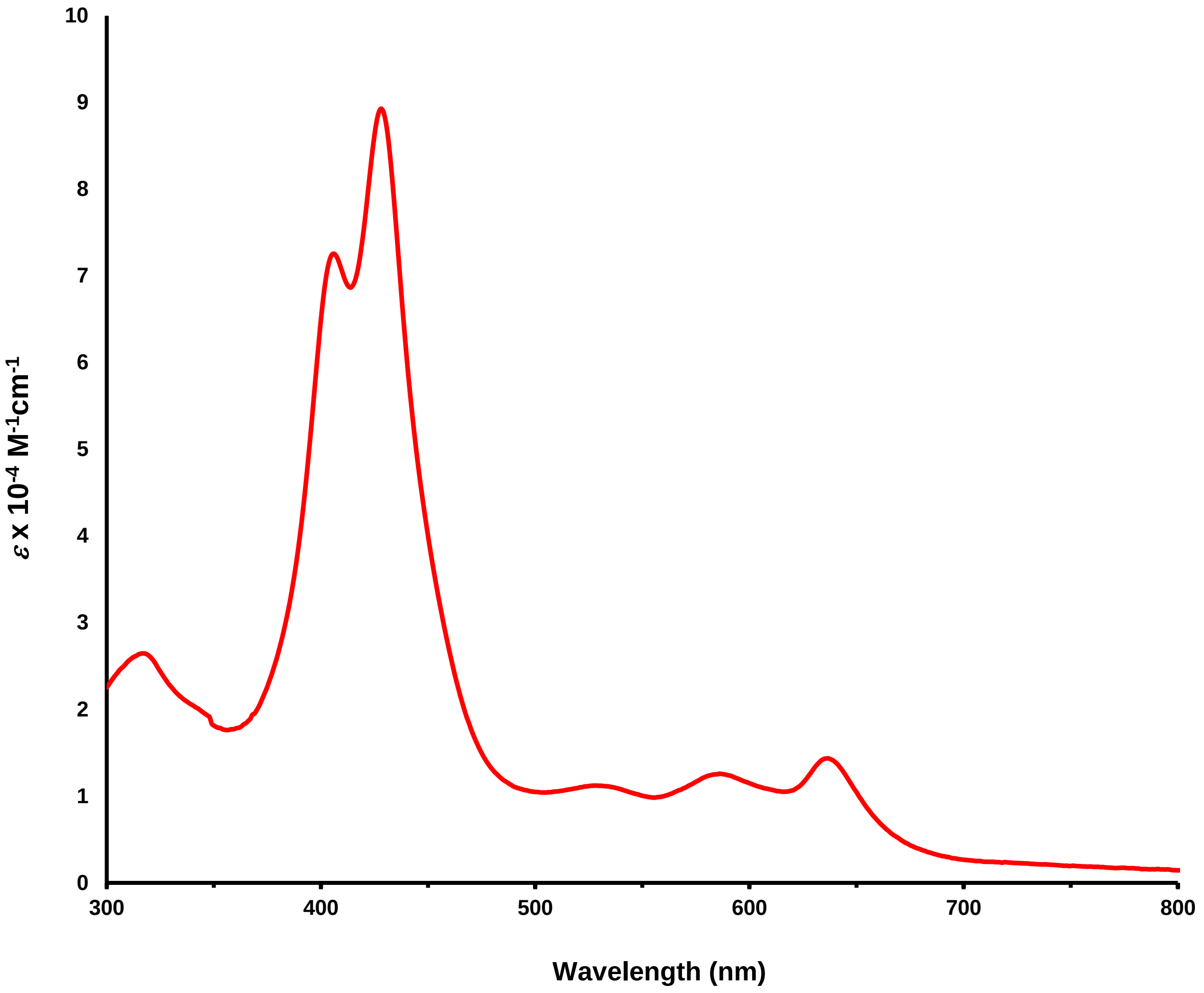

Figure S17. UV-vis spectrum of *N*-methyl pyrenoporphyrin **8** in 5% TFA-CH_2Cl_2.

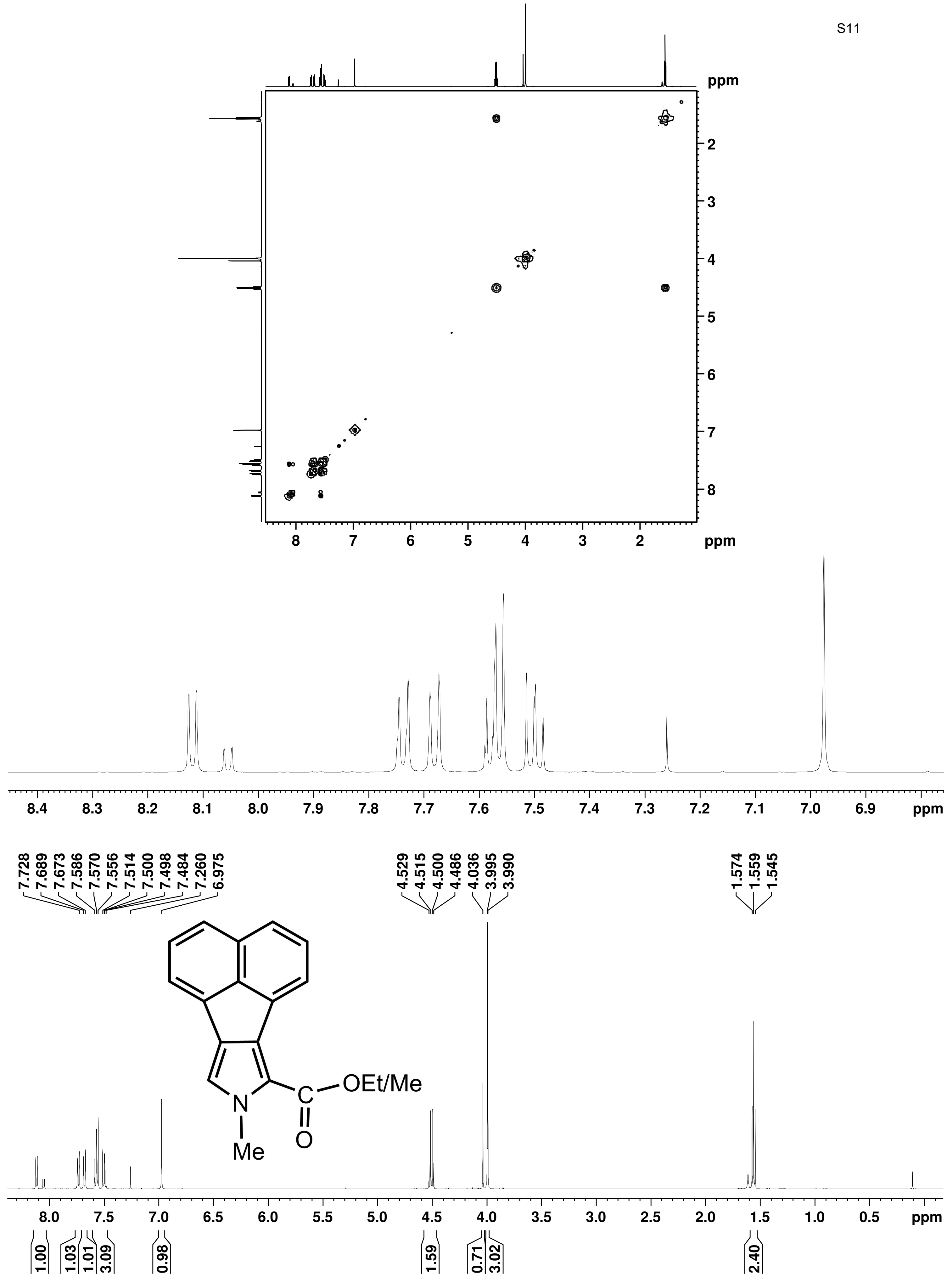

Figure S18. 500 MHz proton and ^{1}H-^{1}H COSY NMR spectra of a mixture of ethyl and methyl esters of 2-methyl acenaphtho[1,2-*c*]pyrrole-1-carboxylate **10a/b** in $CDCl_3$.

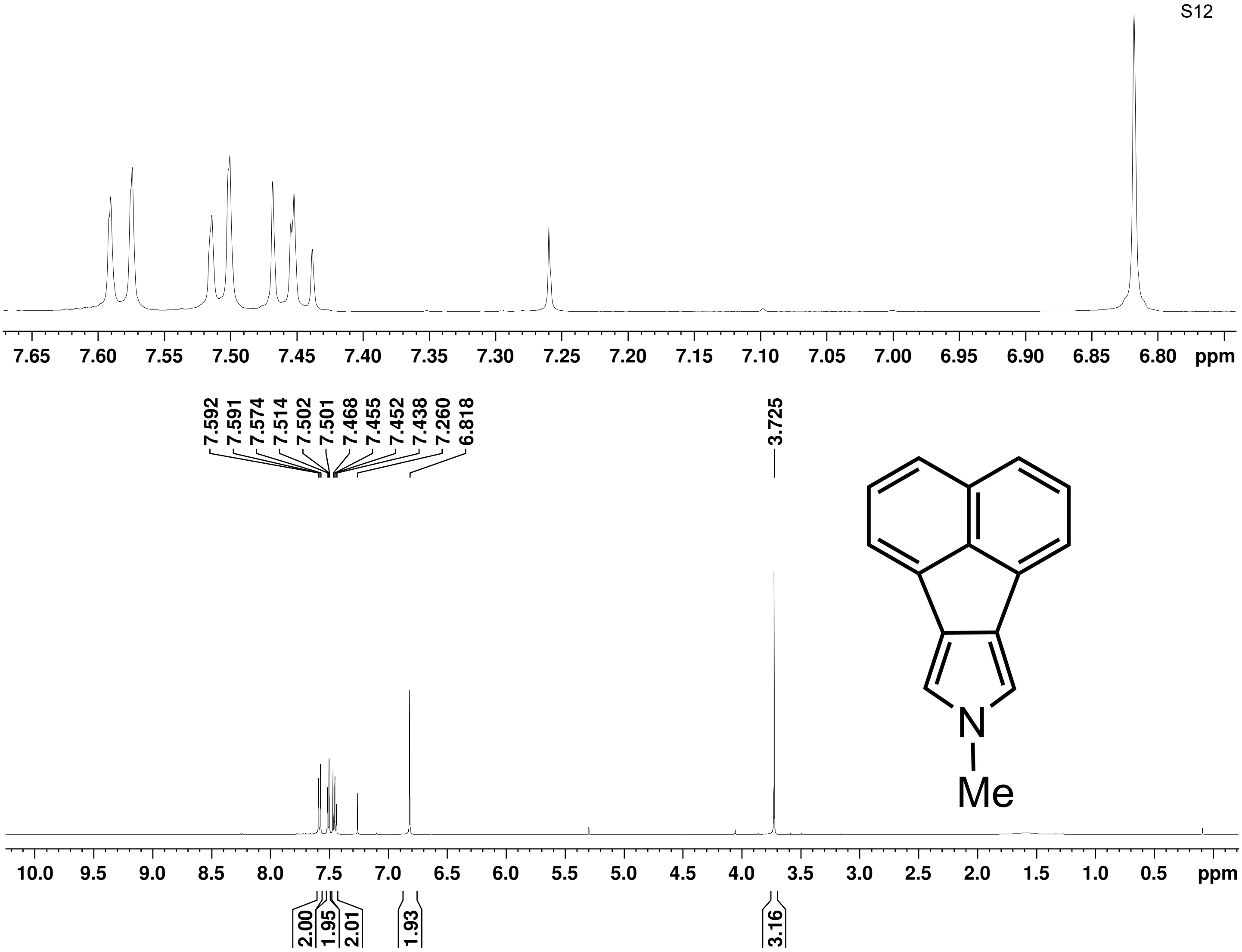

Figure S19. 500 MHz proton NMR spectrum of 2-methyl acenaphtho[1,2-*c*]pyrrole **11b** in $CDCl_3$.

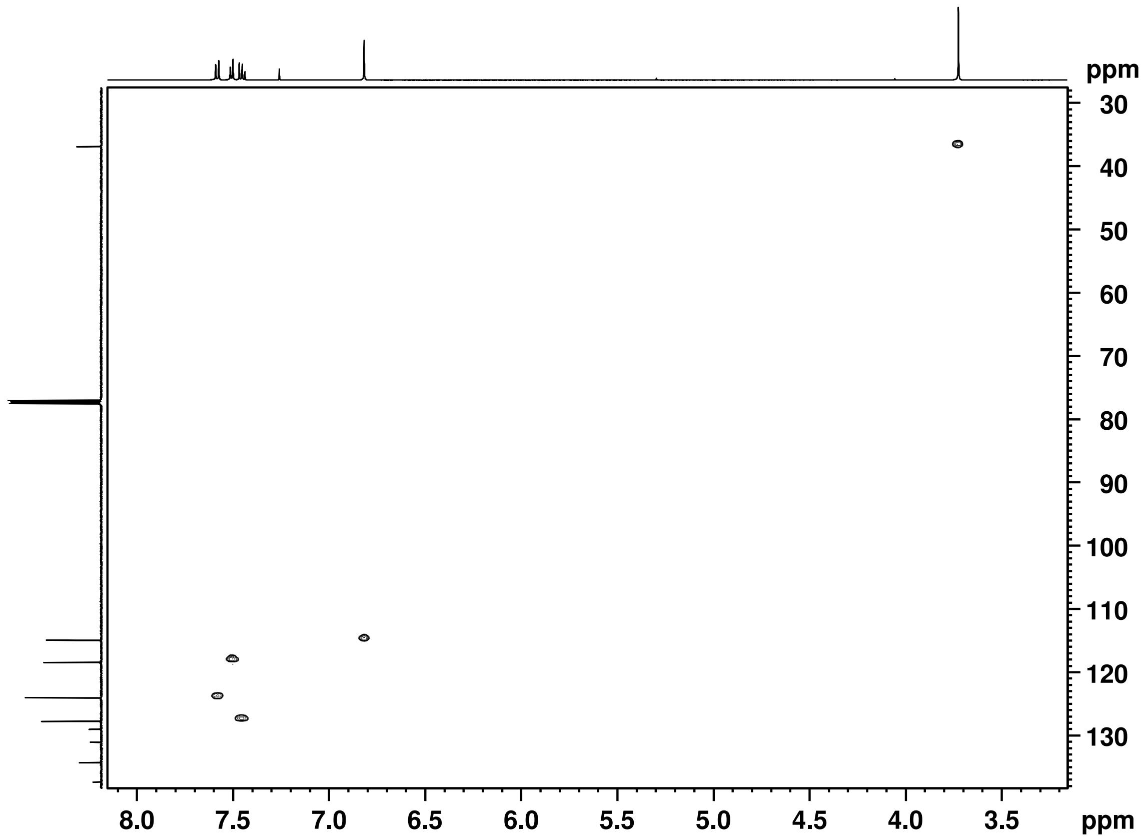

Figure S20. HSQC NMR spectrum of 2-methyl acenaphtho[1,2-*c*]pyrrole **11b** in $CDCl_3$.

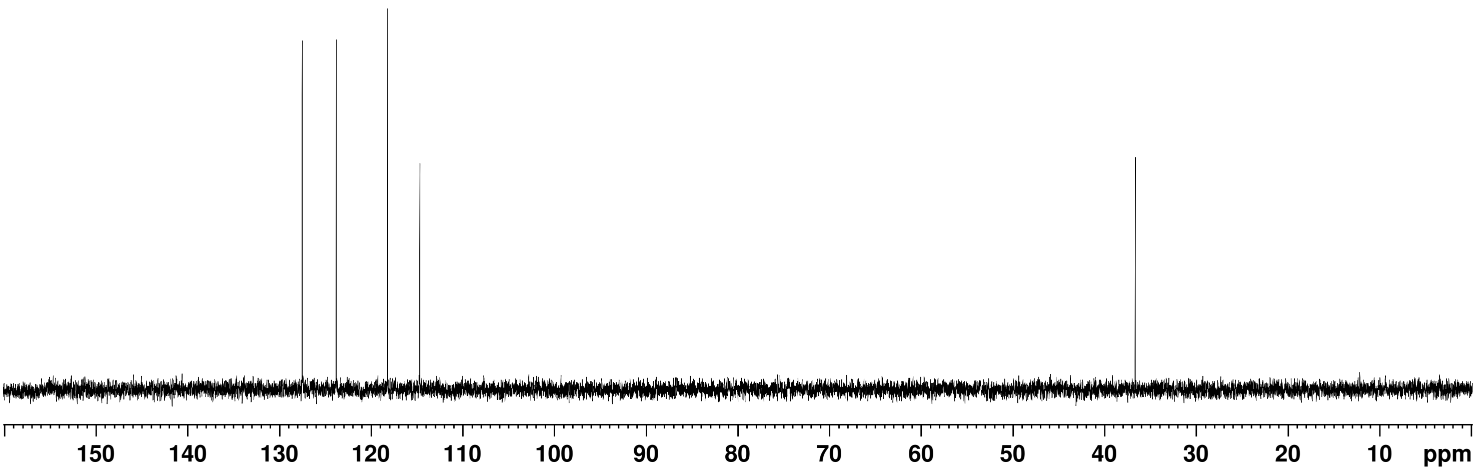

Figure S21. DEPT-135 NMR spectrum of 2-methyl acenaphtho[1,2-*c*]pyrrole **11b** in $CDCl_3$.

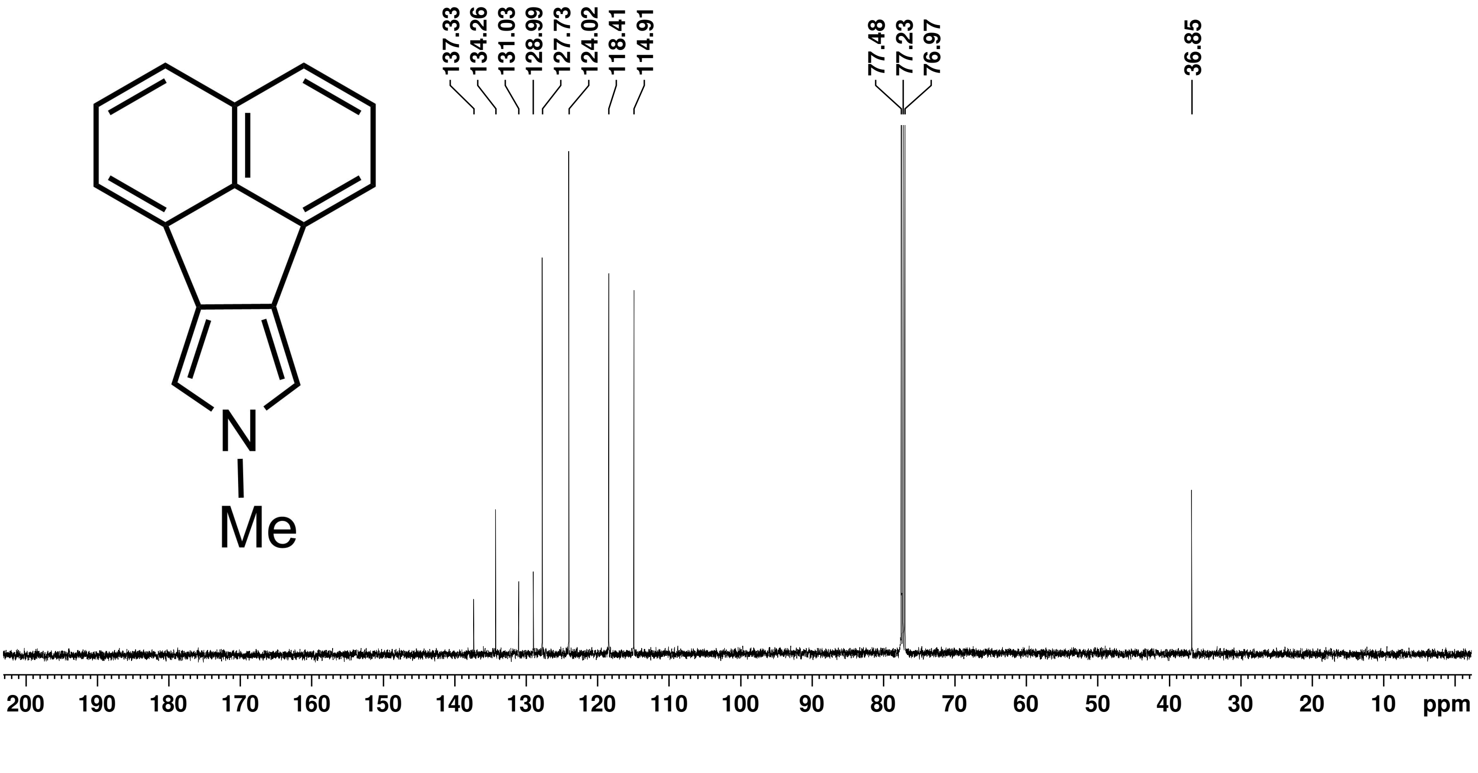

Figure S22. 500 MHz proton NMR spectrum of 2-methyl acenaphtho[1,2-*c*]pyrrole **11b** in $CDCl_3$.

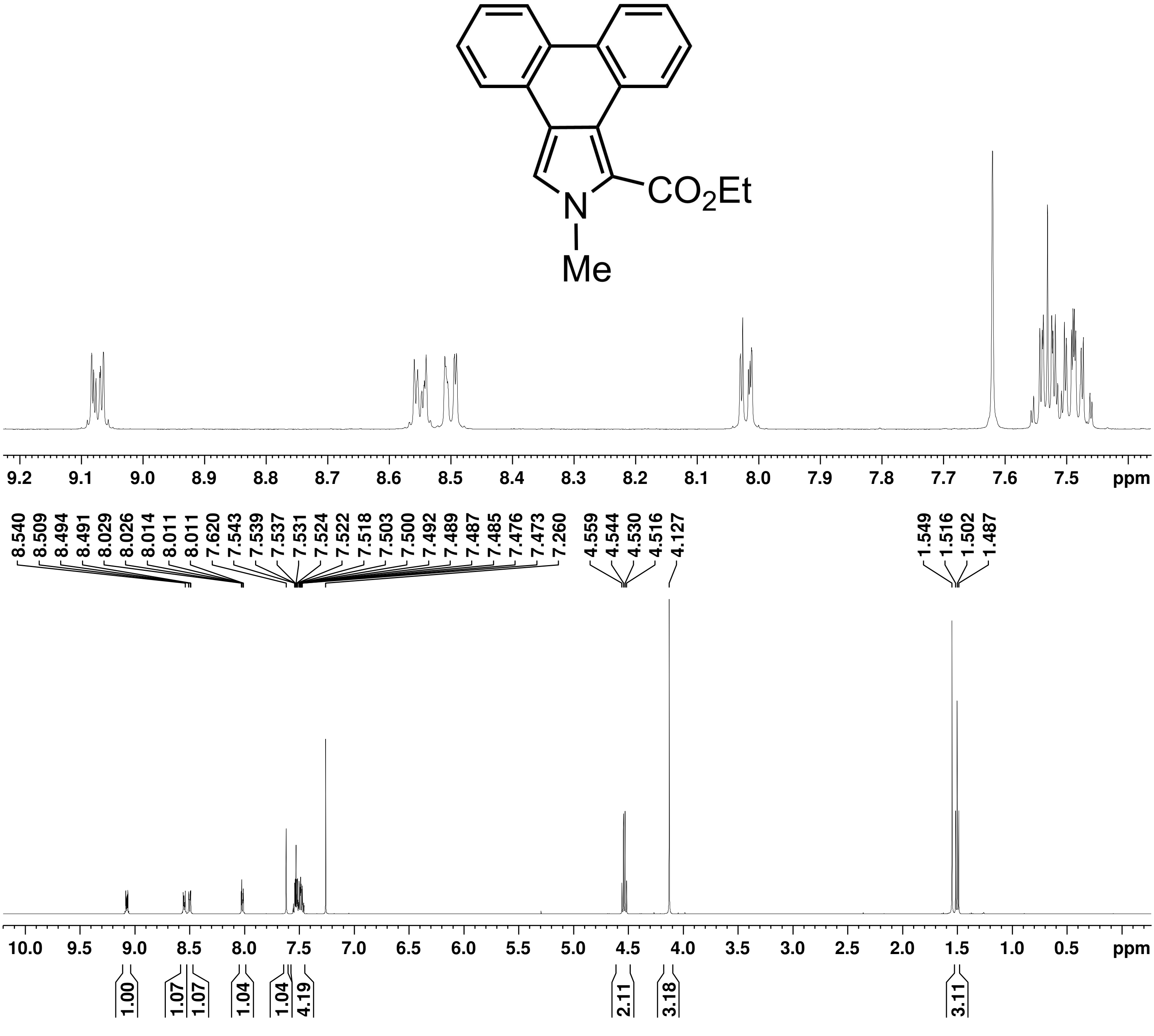

Figure S23. 500 MHz proton NMR spectrum of 2-methylphenanthro[1,2-*c*]pyrrole ethyl ester **16** in $CDCl_3$.

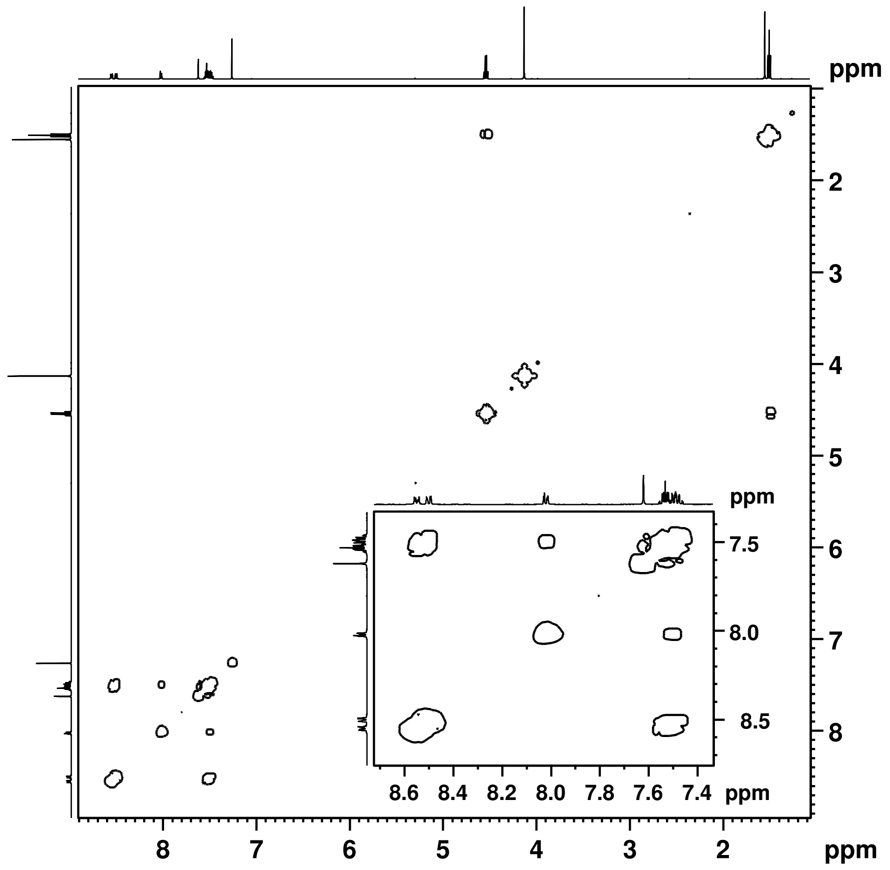

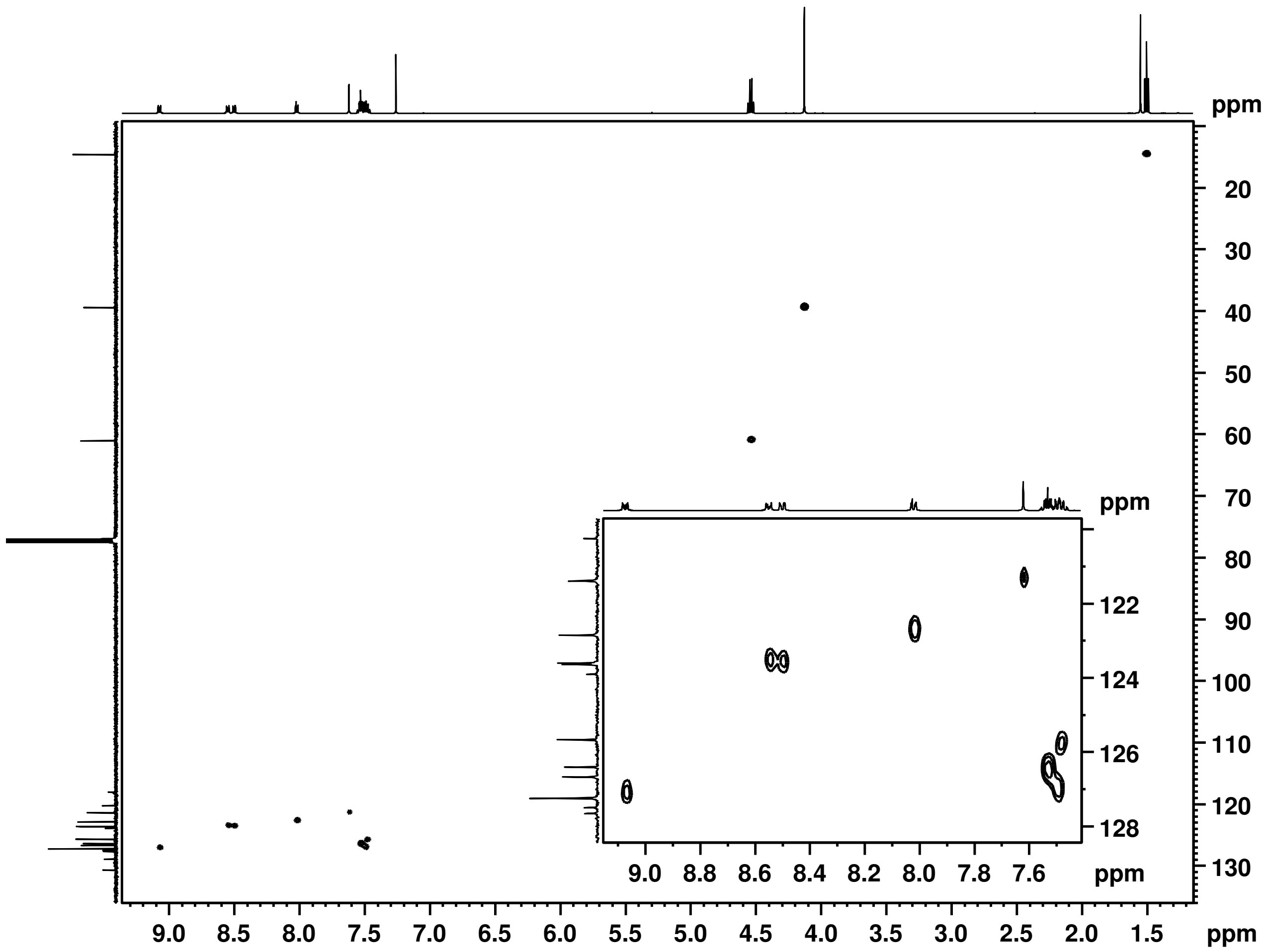

Figure S24. ^{1}H-^{1}H COSY (top) and HSQC (bottom) NMR spectra of **16** in $CDCl_3$.

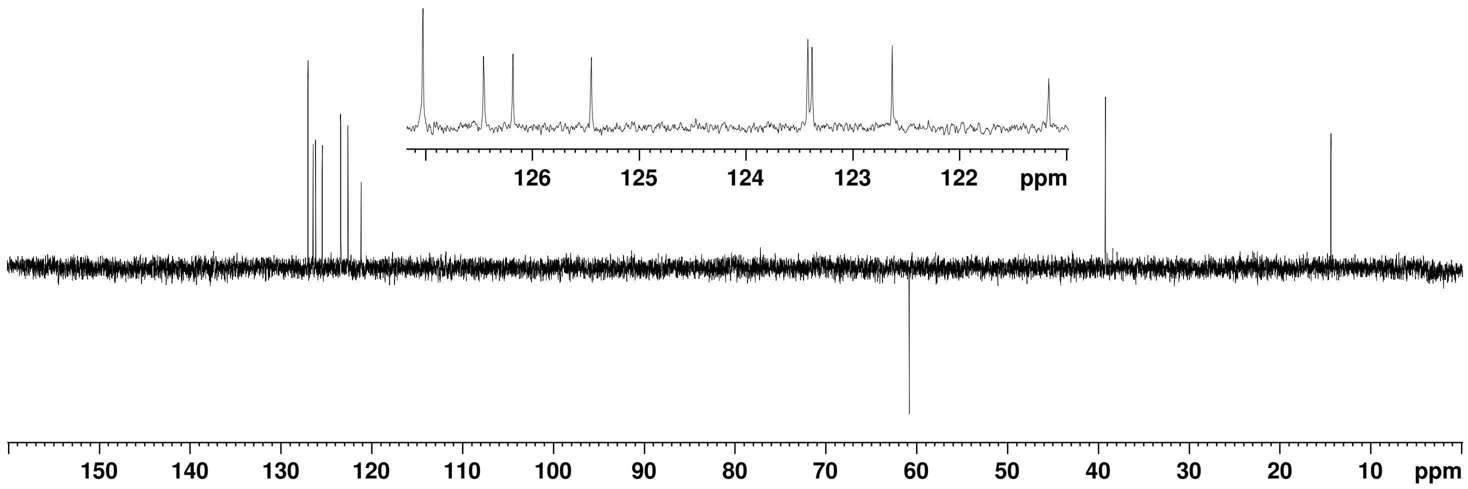

Figure S25. DEPT-135 NMR spectrum of phenanthropyrrole ethyl ester **16** in $CDCl_3$.

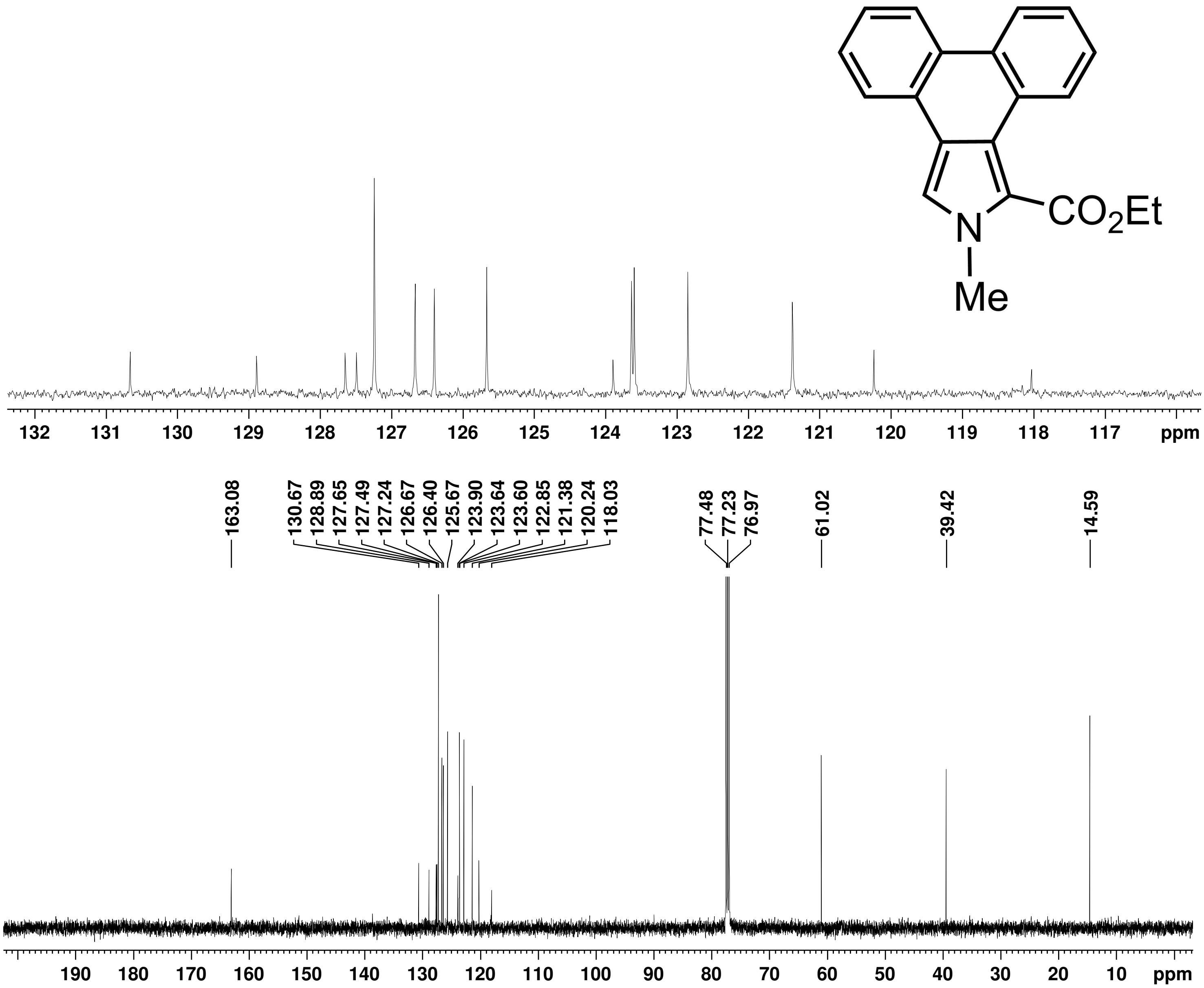

Figure S26. 125 MHz carbon-13 NMR spectrum of phenanthropyrrole ethyl ester **16** in $CDCl_3$.

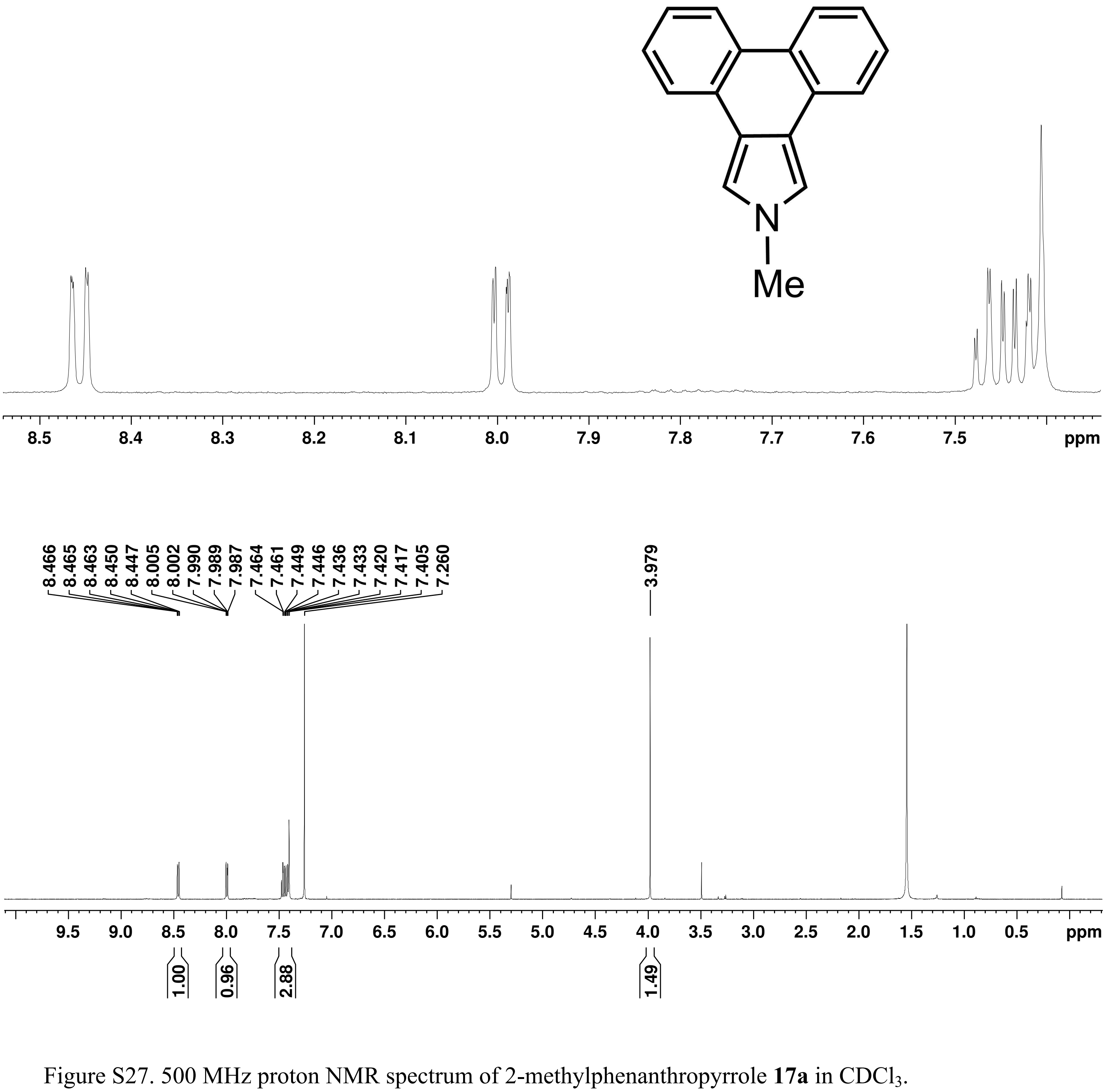

Figure S27. 500 MHz proton NMR spectrum of 2-methylphenanthropyrrole **17a** in $CDCl_3$.

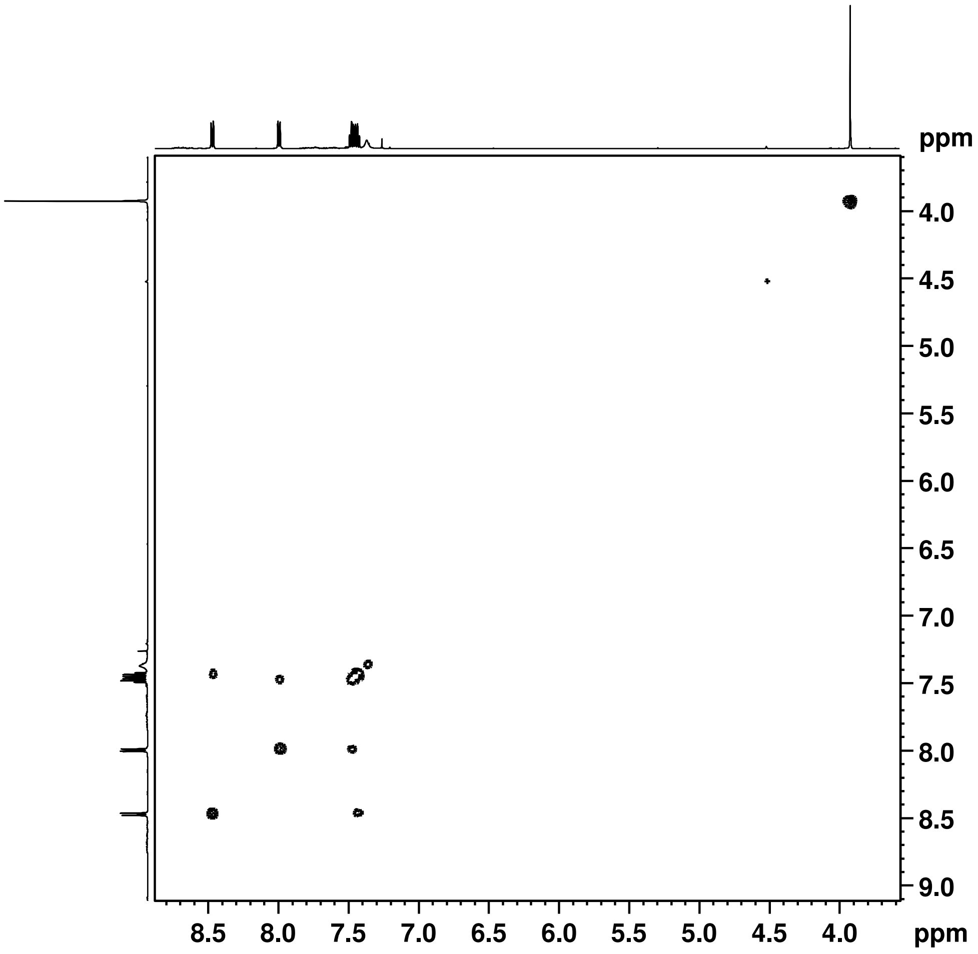

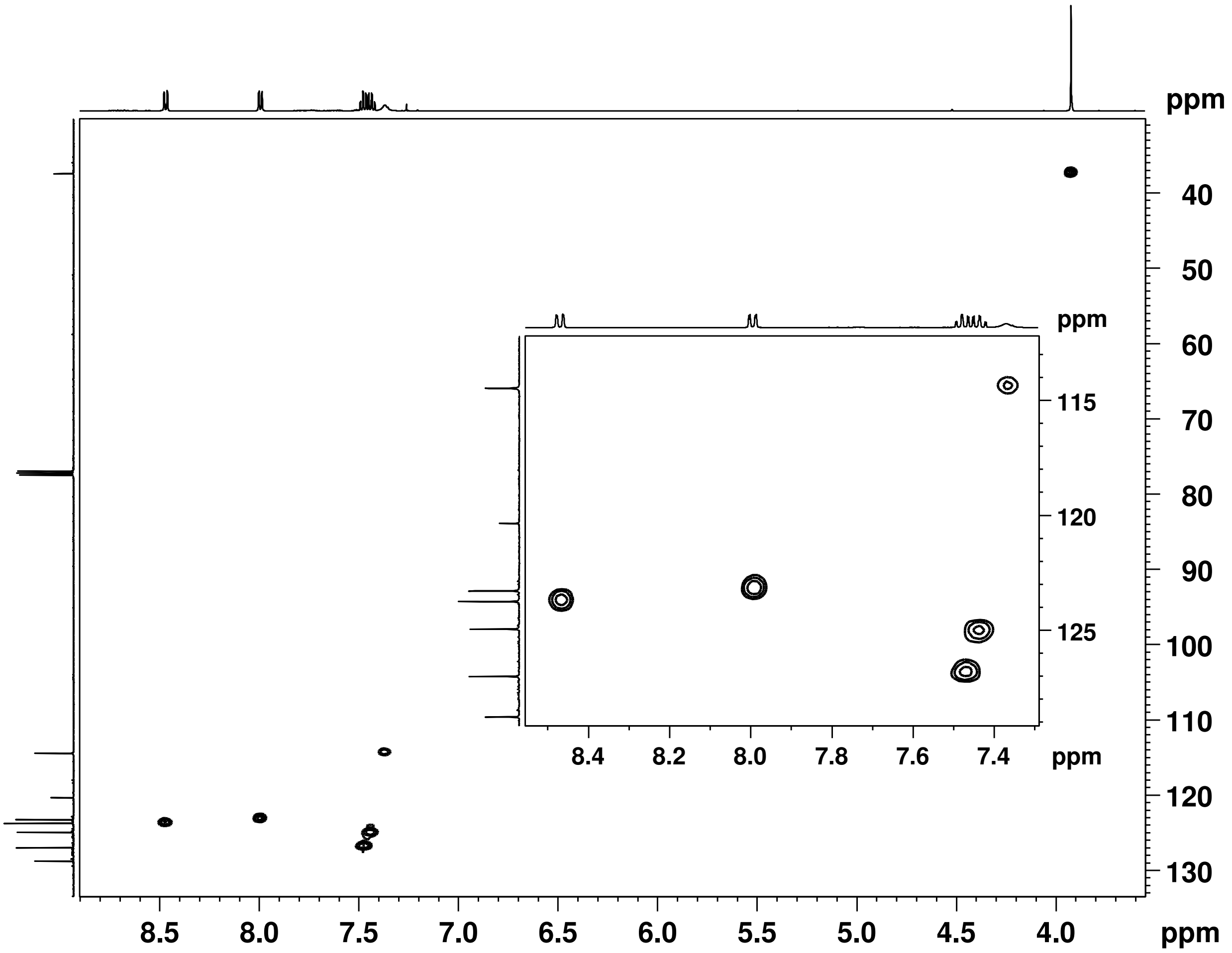

Figure S28. ^{1}H-^{1}H COSY (top) and HSQC (bottom) NMR spectra of **17a** in $CDCl_3$.

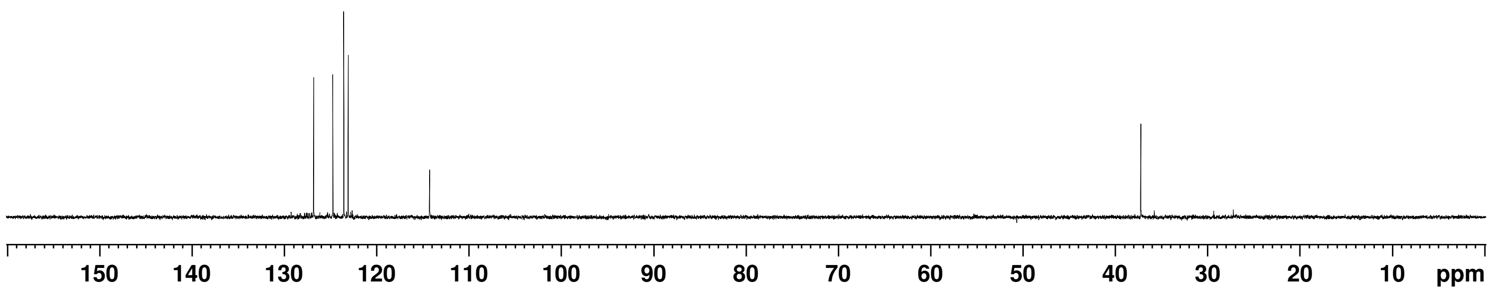

Figure S29. DEPT-135 NMR spectrum of 2-methylphenanthropyrrole **17a** in $CDCl_3$.

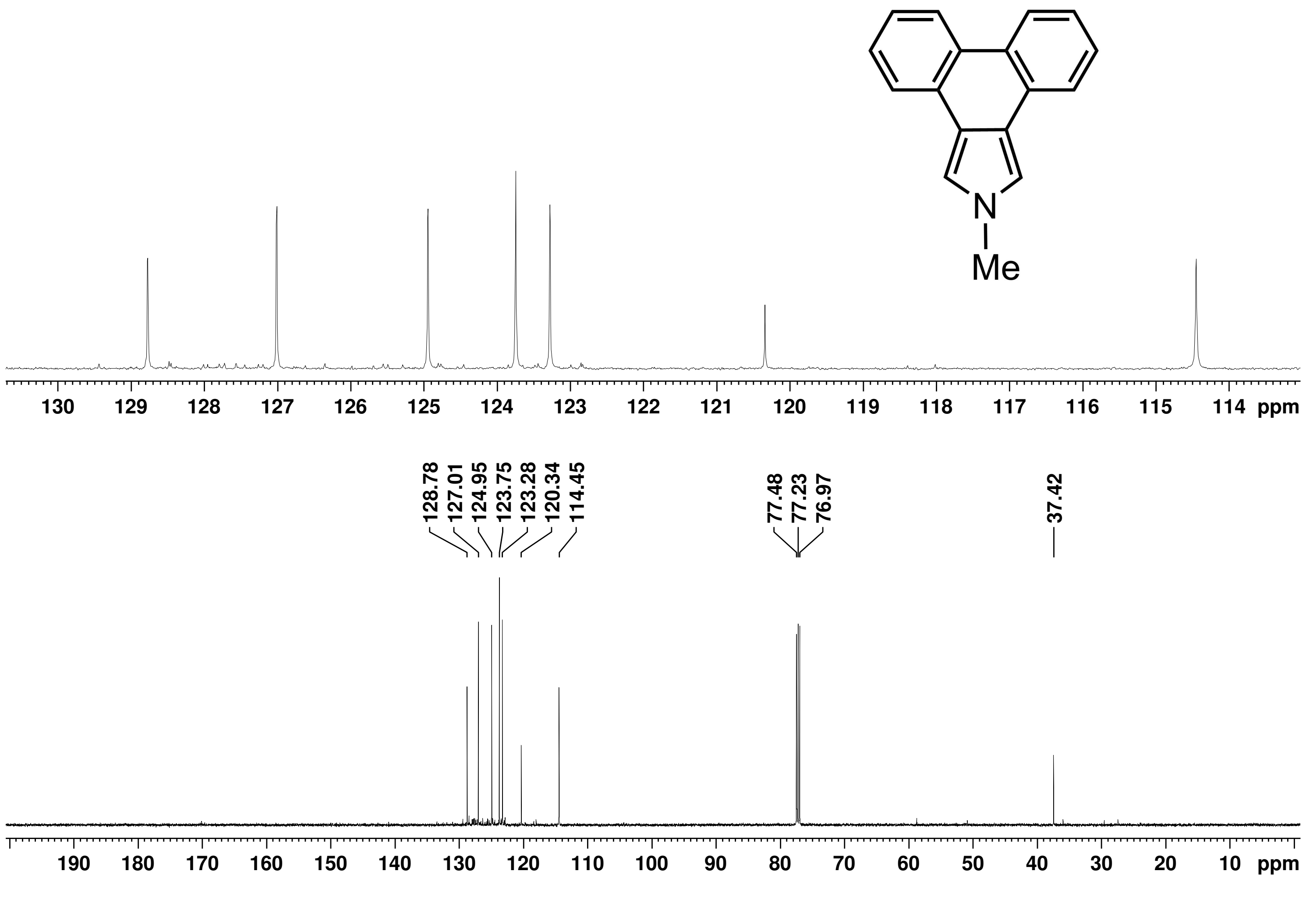

Figure S30. 125 MHz carbon-13 NMR spectrum of 2-methylphenanthropyrrole **17a** in $CDCl_3$.

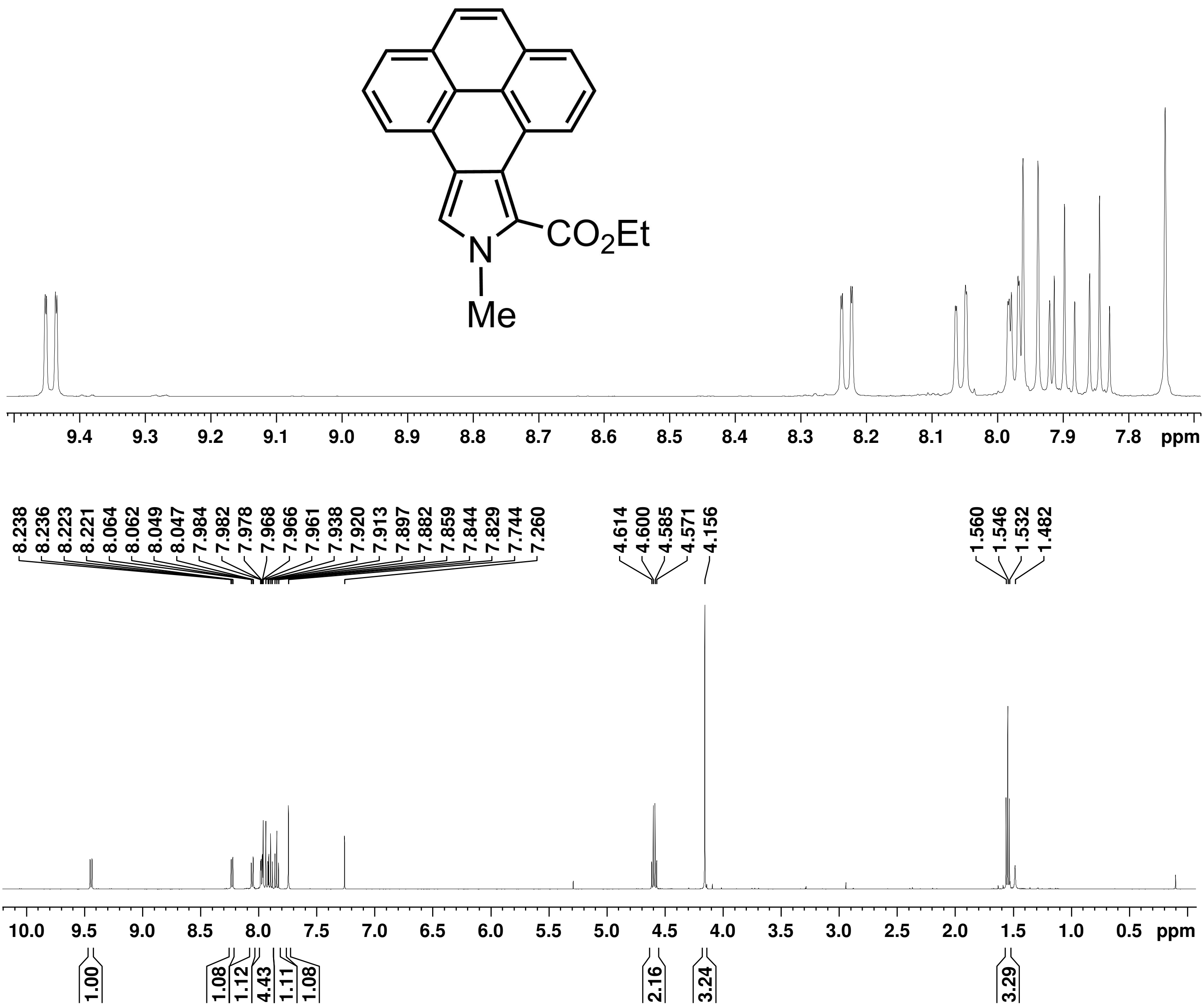

Figure S31. 500 MHz proton NMR spectrum of N-methylpyrenopyrrole ethyl ester **19** in $CDCl_3$.

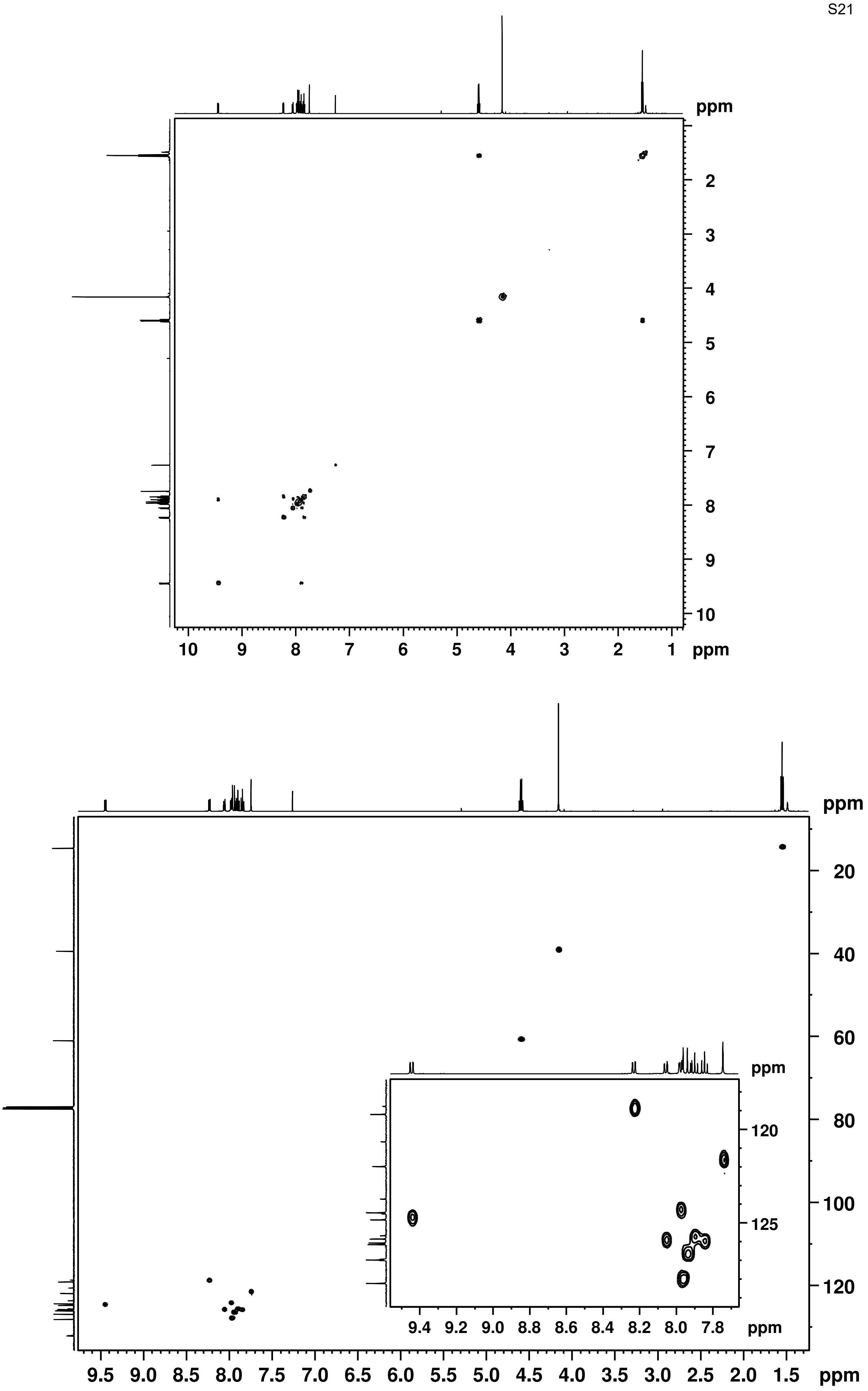

Figure S32. ^{1}H-^{1}H COSY (top) and HSQC (bottom) NMR spectra of pyrenopyrrole ester **19** in $CDCl_3$.

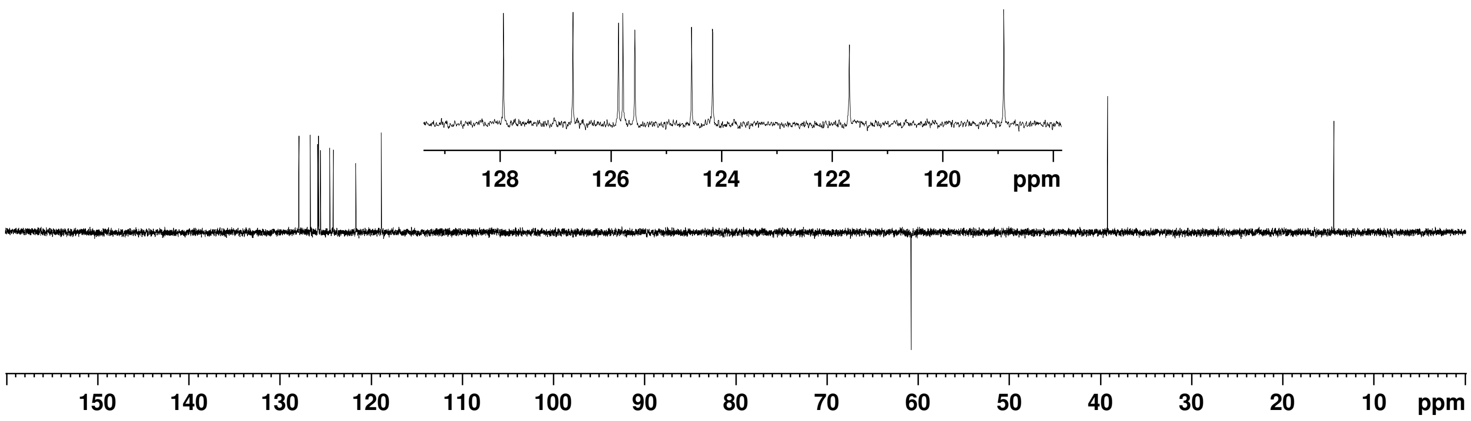

Figure S33. DEPT-135 NMR spectrum of pyrenopyrrole ester **19** in $CDCl_3$.

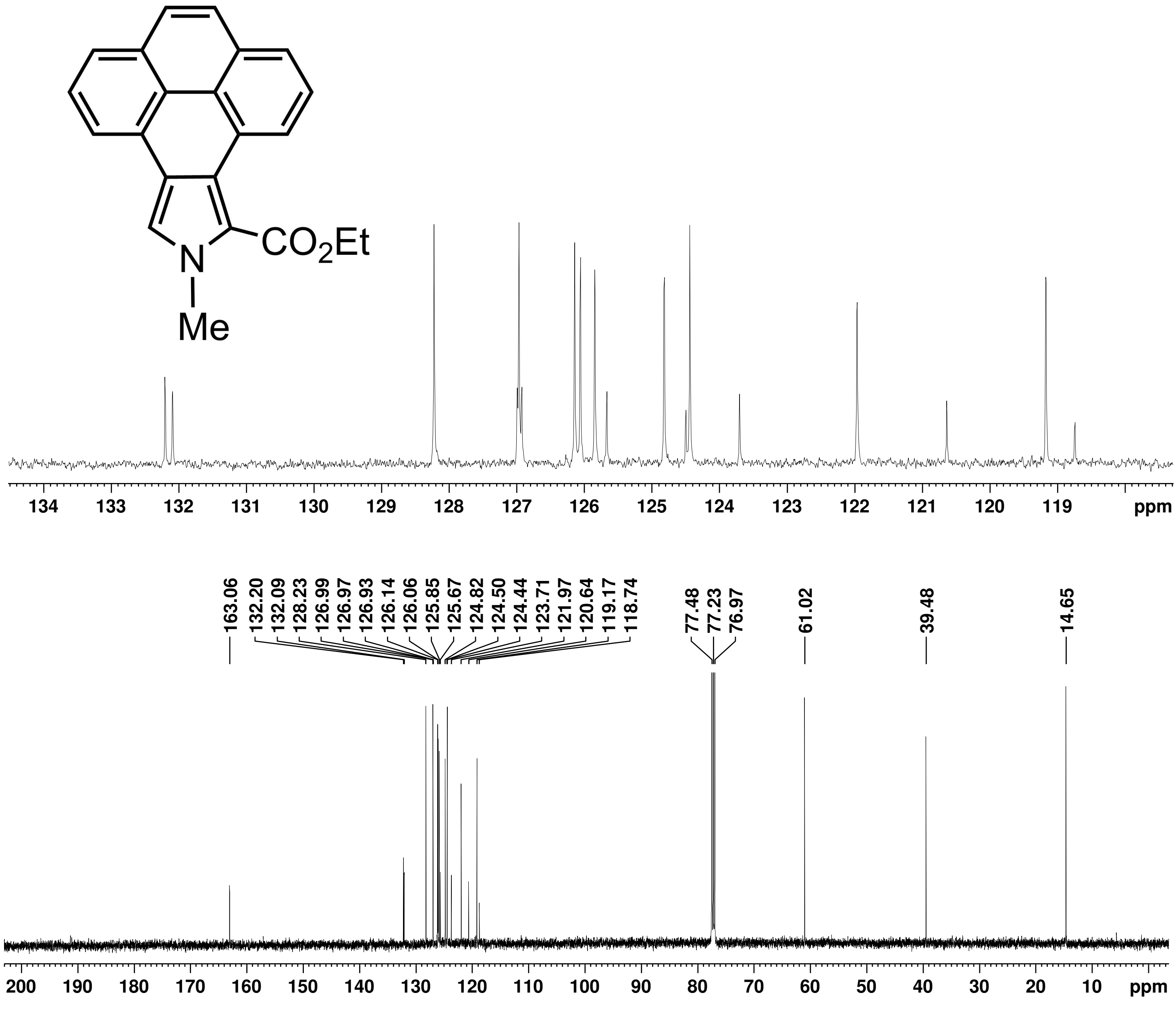

Figure S34. 125 MHz carbon-13 NMR spectrum of pyrenopyrrole ester **19** in $CDCl_3$.

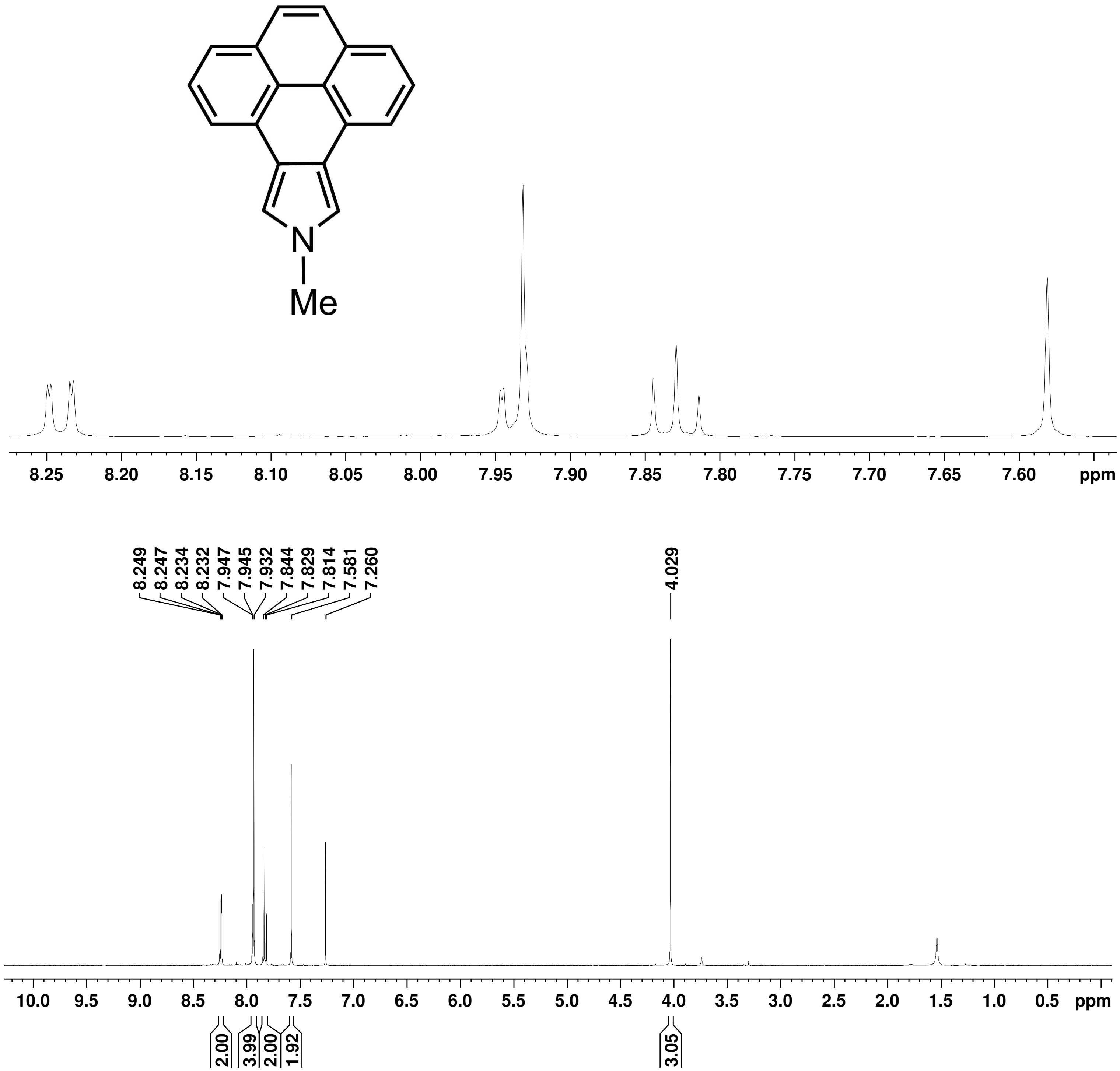

Figure S35. 500 MHz proton NMR spectrum of *N*-methylpyrenopyrrole **20a** in $CDCl_3$.

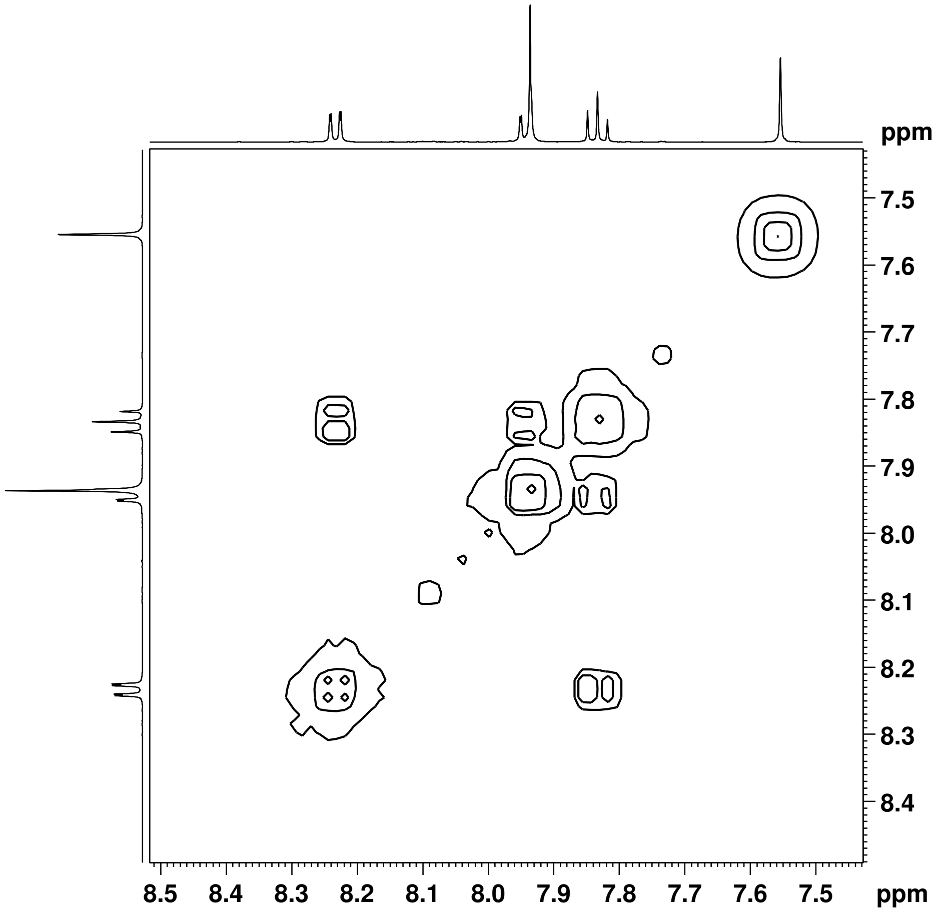

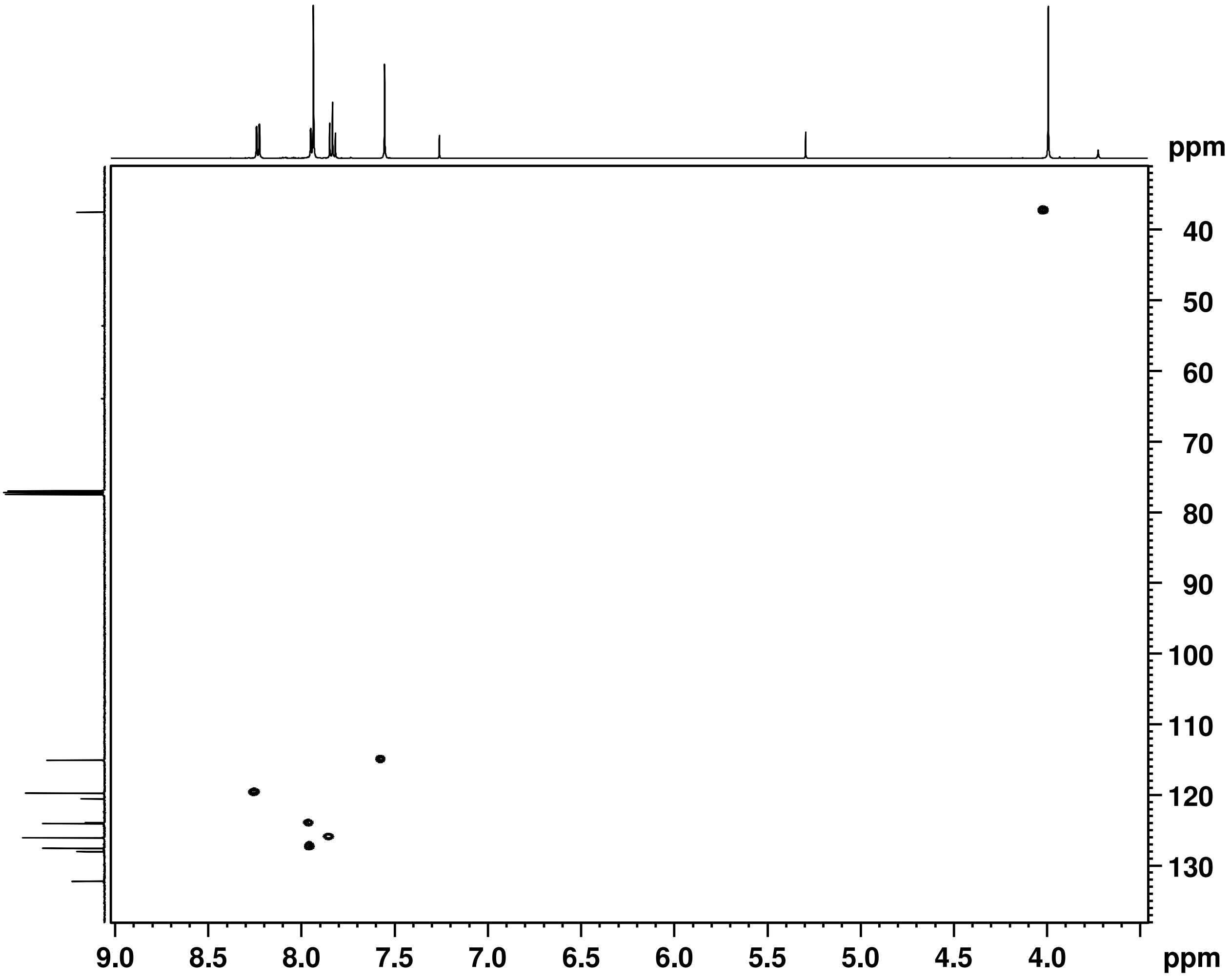

Figure S36. ^{1}H-^{1}H COSY (top) and HSQC (bottom) NMR spectra of **20a** in $CDCl_3$.

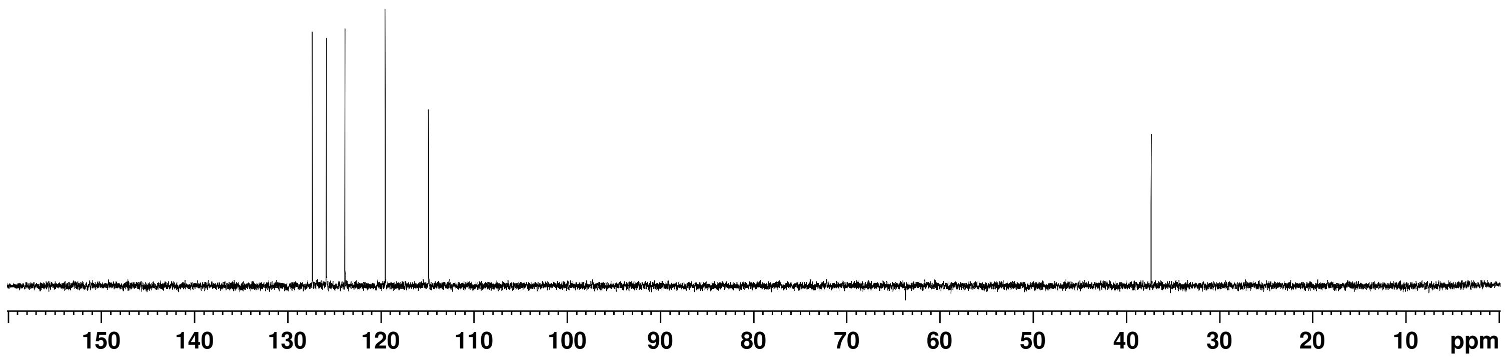

Figure S37. DEPT-135 NMR spectrum of N-methylpyrenopyrrole **20a** in $CDCl_3$.

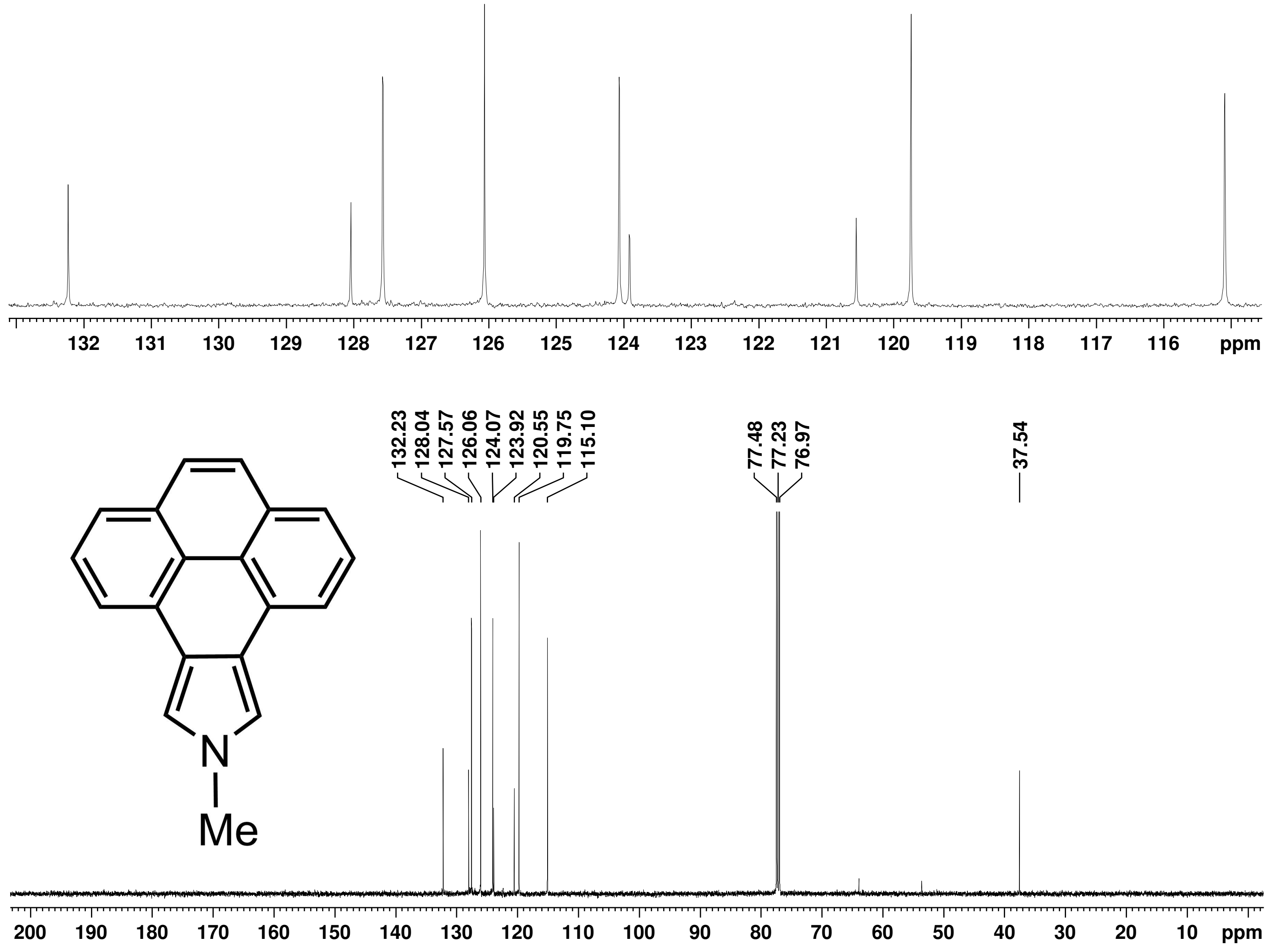

Figure S38. 125 MHz carbon-13 NMR spectrum of *N*-methylpyrenopyrrole **20a** in $CDCl_3$.

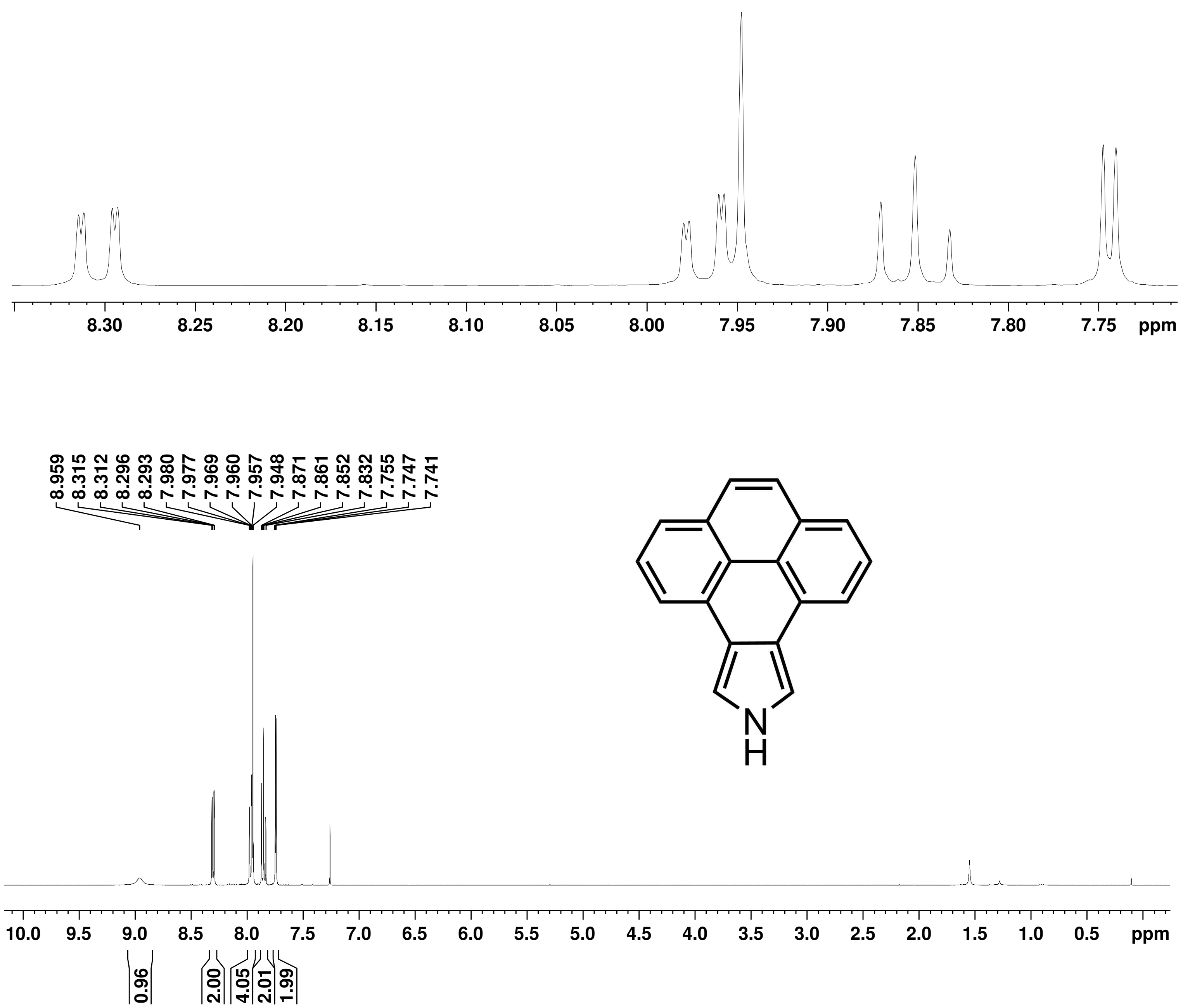

Figure S39. 500 MHz proton NMR spectrum of pyrenopyrrole **20b** in $CDCl_3$.

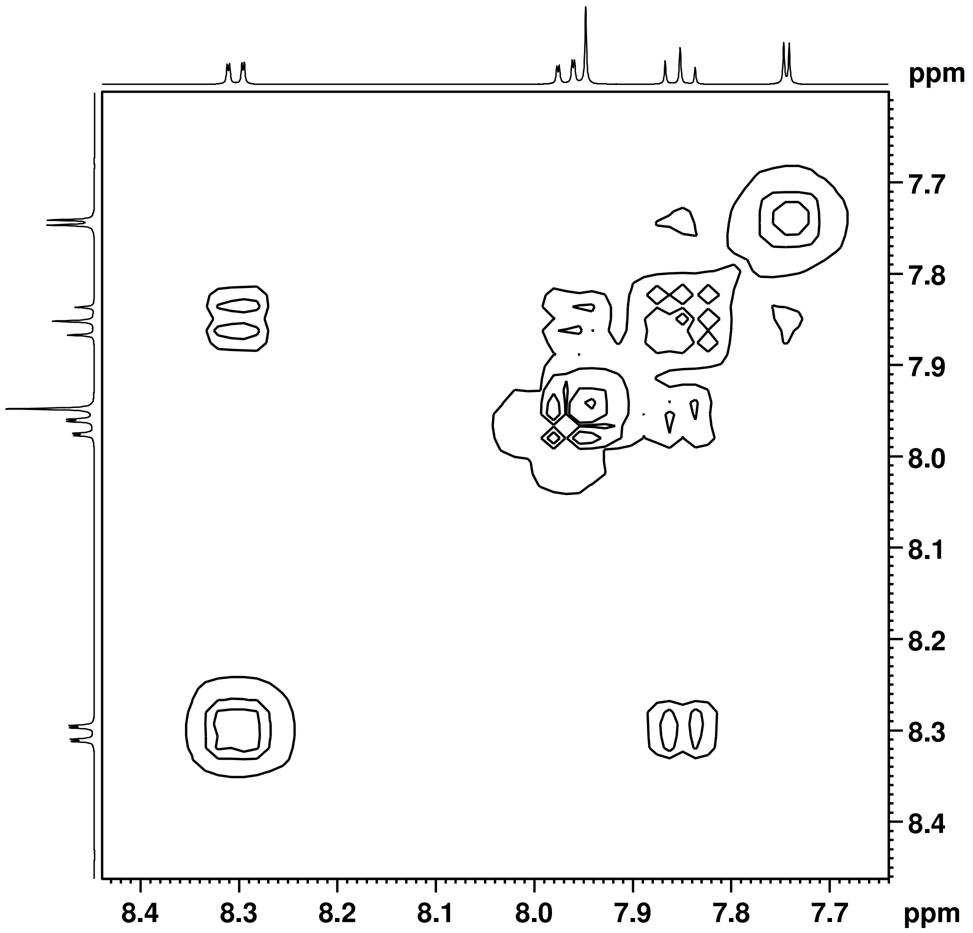

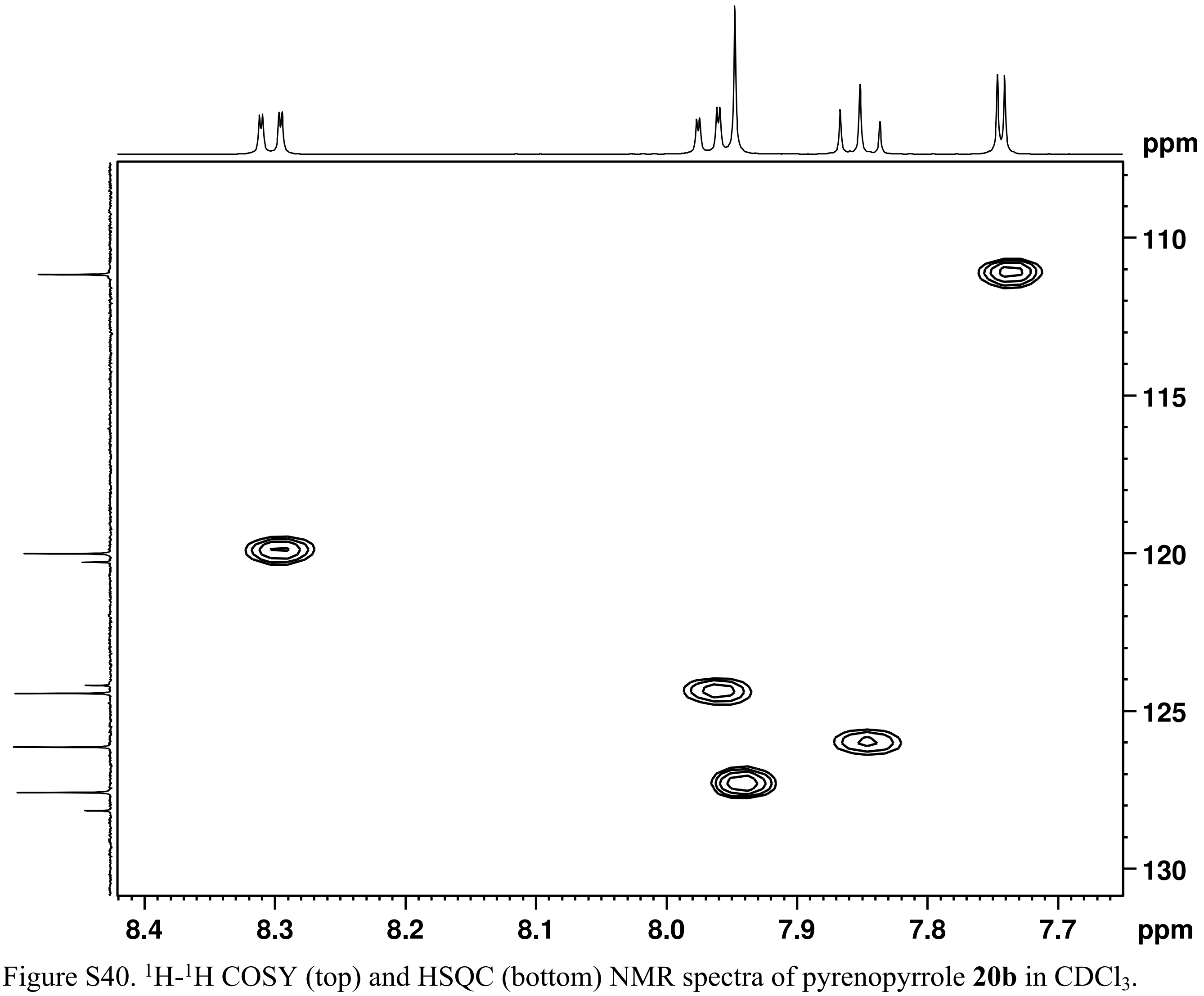

Figure S40. ^{1}H-^{1}H COSY (top) and HSQC (bottom) NMR spectra of pyrenopyrrole **20b** in $CDCl_3$.

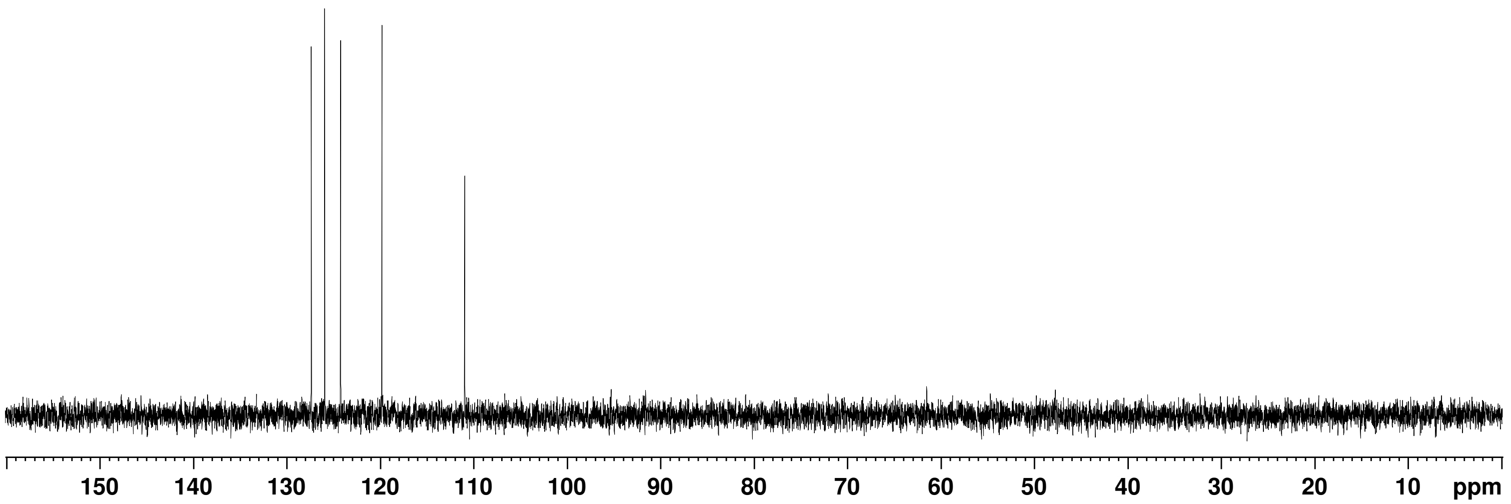

Figure S41. DEPT-135 NMR spectrum of pyrenopyrrole **20b** in $CDCl_3$.

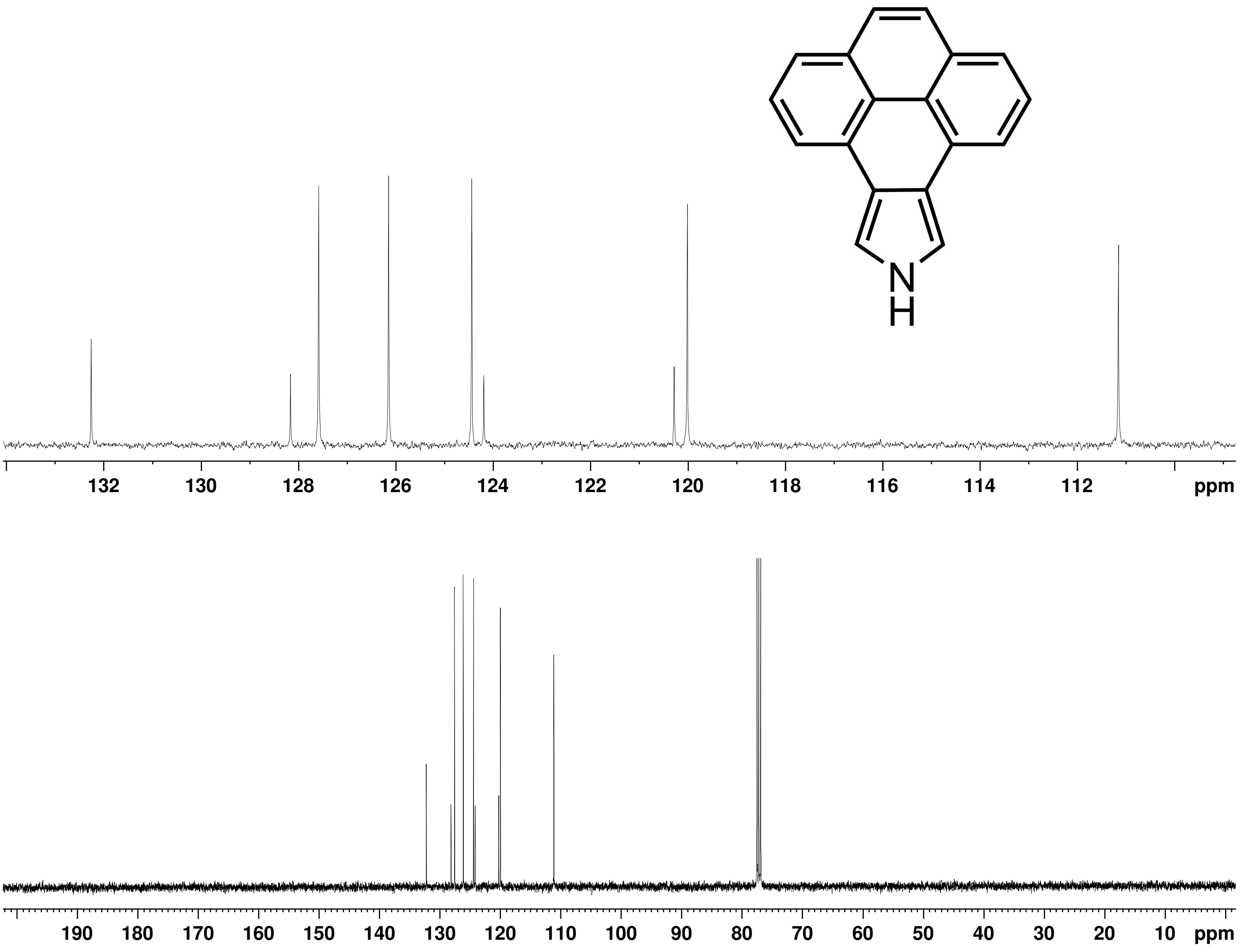

Figure S42. 125 MHz carbon-13 NMR spectrum of pyrenopyrrole **20b** in $CDCl_3$.

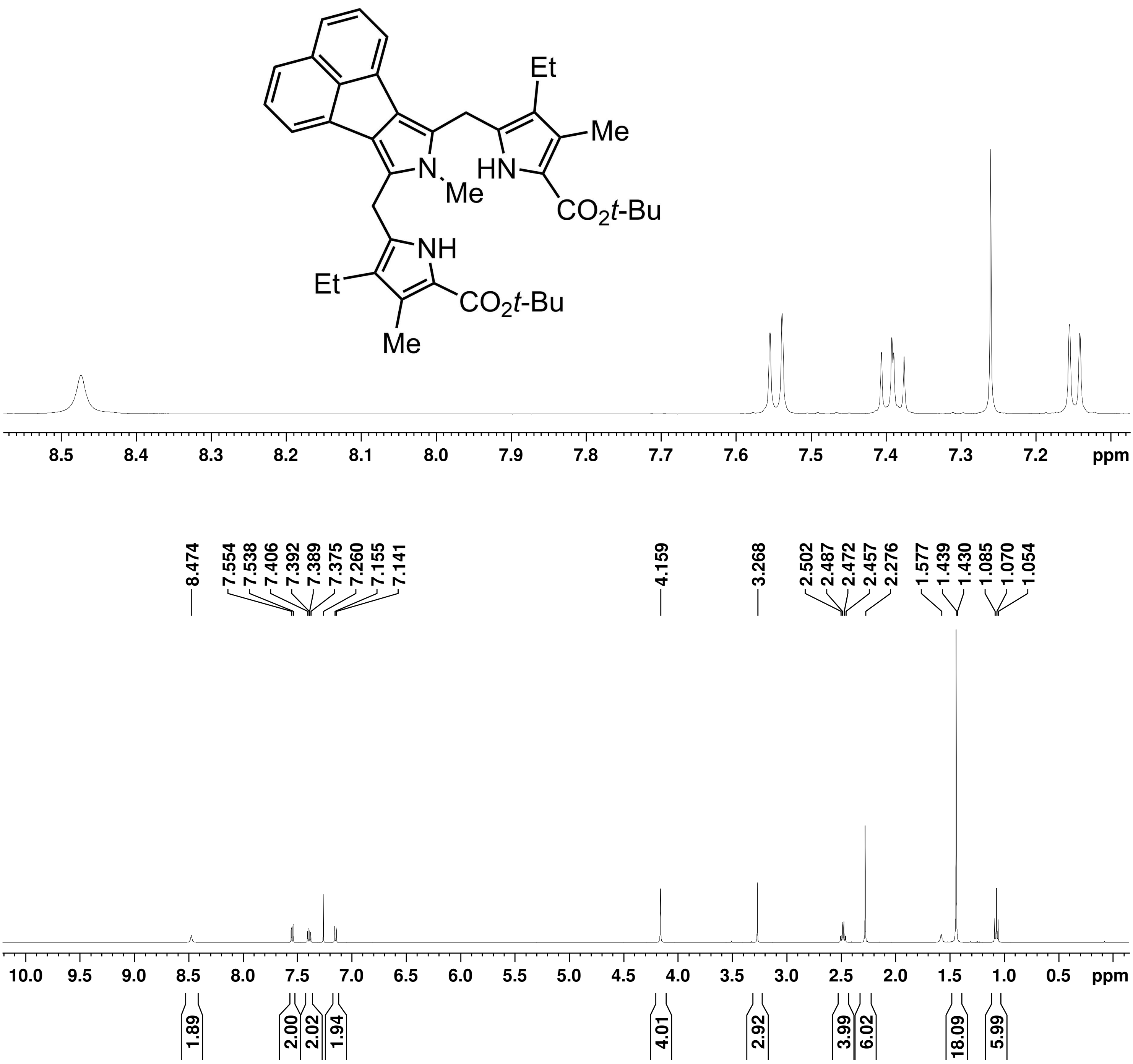

Figure S43. 500 MHz proton NMR spectrum of acenaphthotripyrrane **13** in $CDCl_3$.

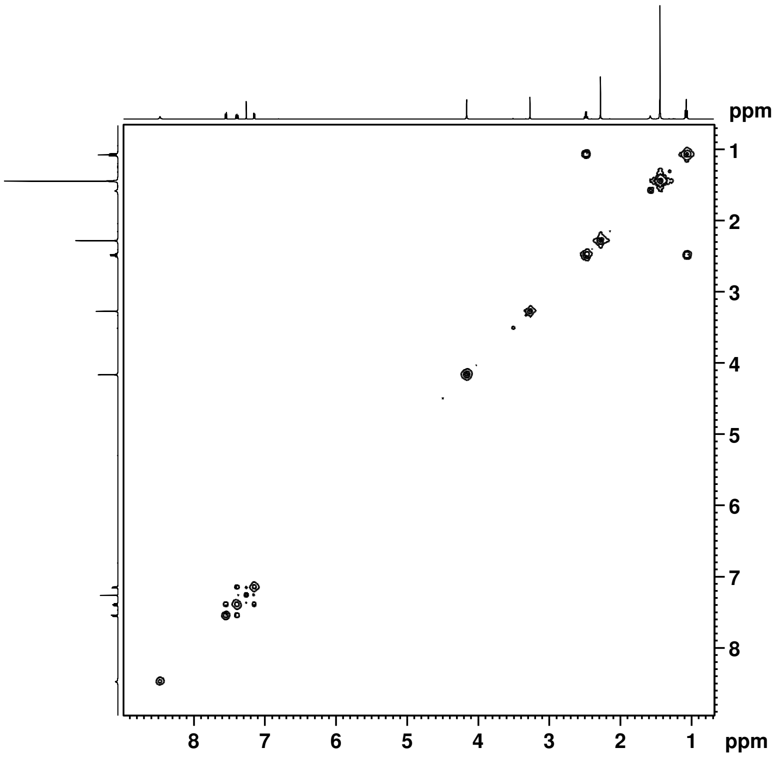

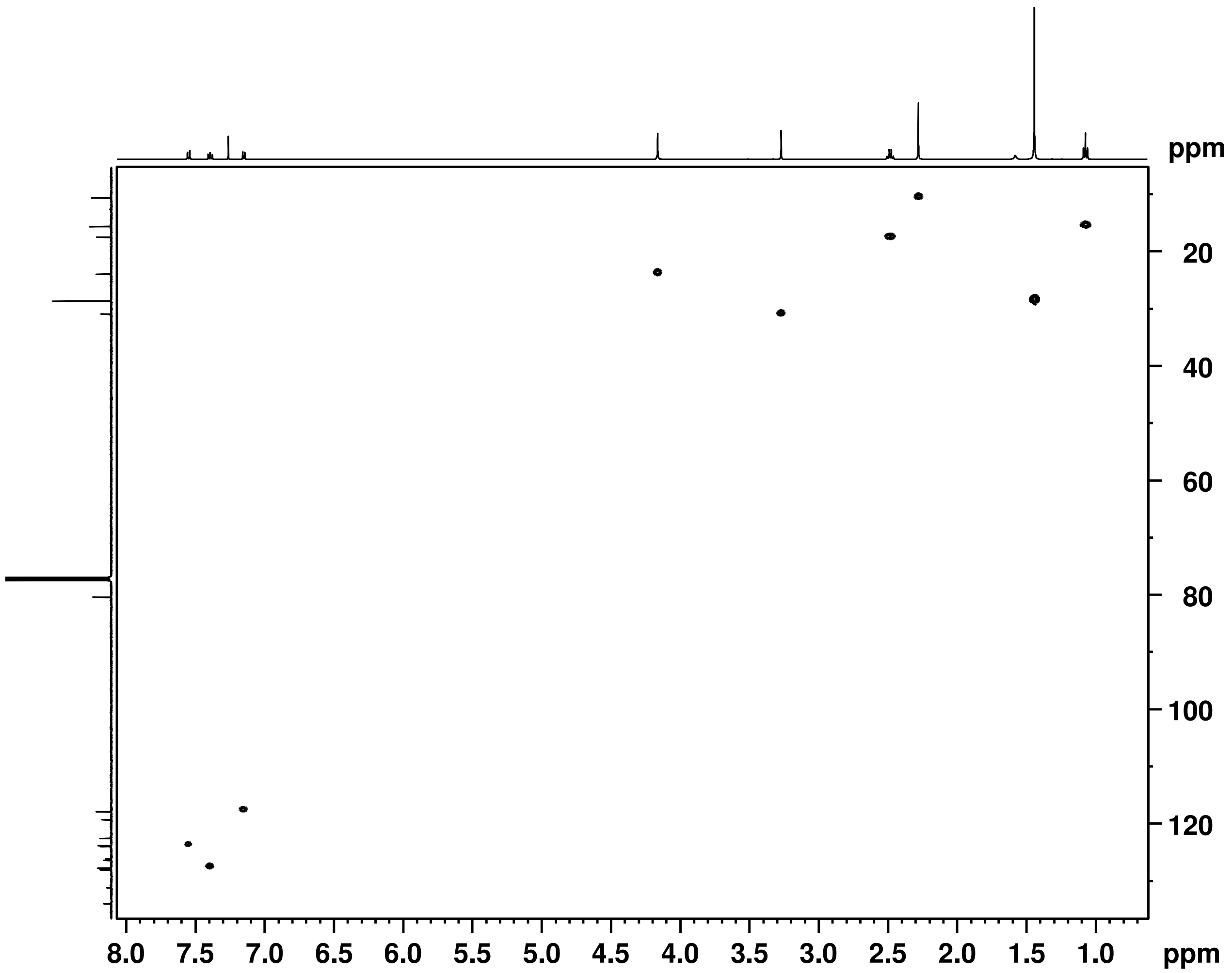

Figure S44. ^{1}H-^{1}H COSY (top) and HSQC (bottom) NMR spectra of acenaphthotripyrrane **13** in $CDCl_3$.

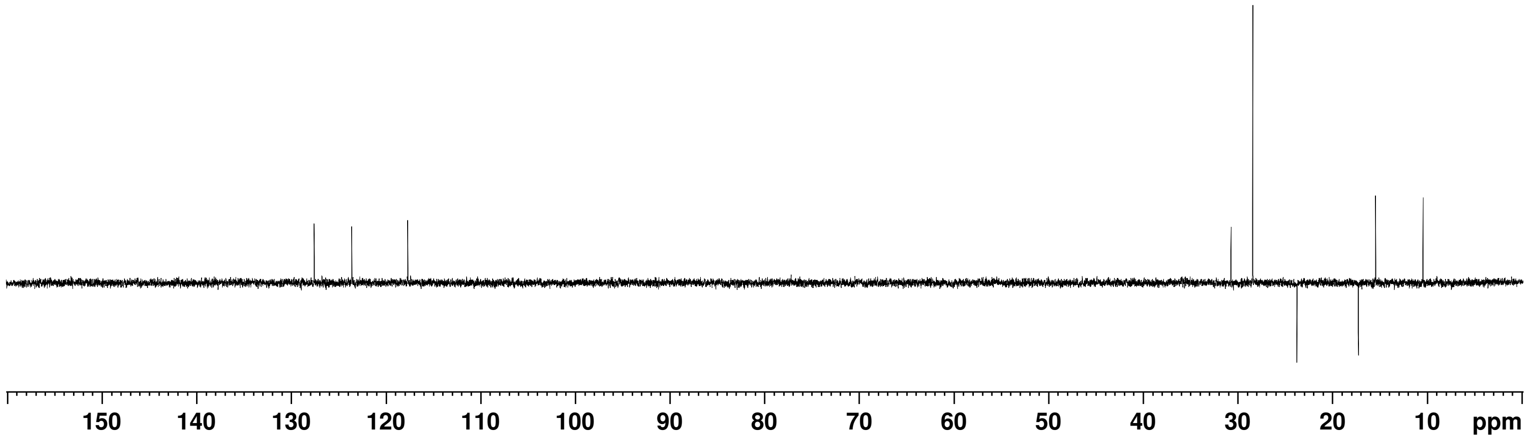

Figure S45. DEPT-135 NMR spectrum of acenaphthotripyrrane **13** in $CDCl_3$.

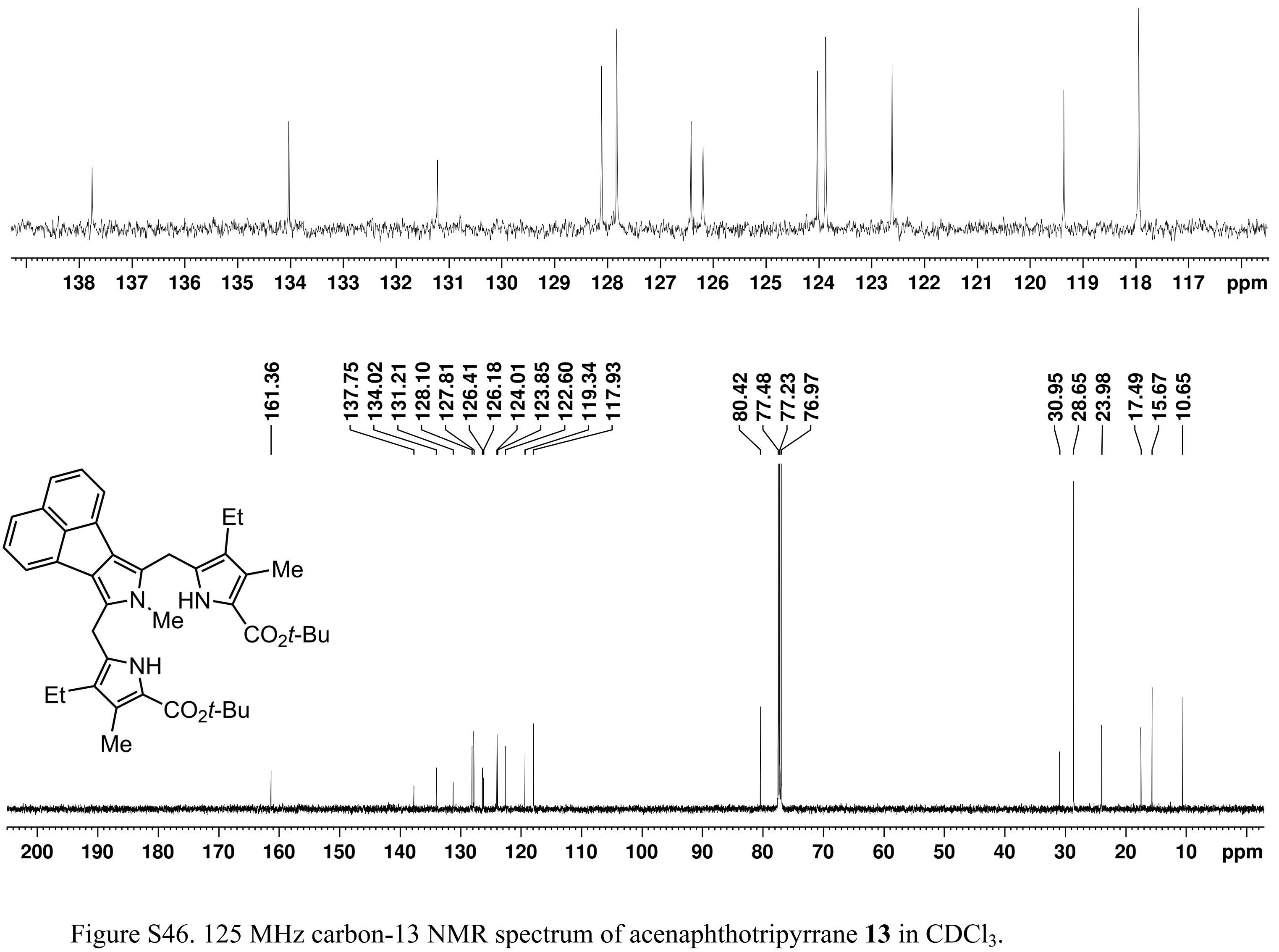

Figure S46. 125 MHz carbon-13 NMR spectrum of acenaphthotripyrrane **13** in $CDCl_3$.

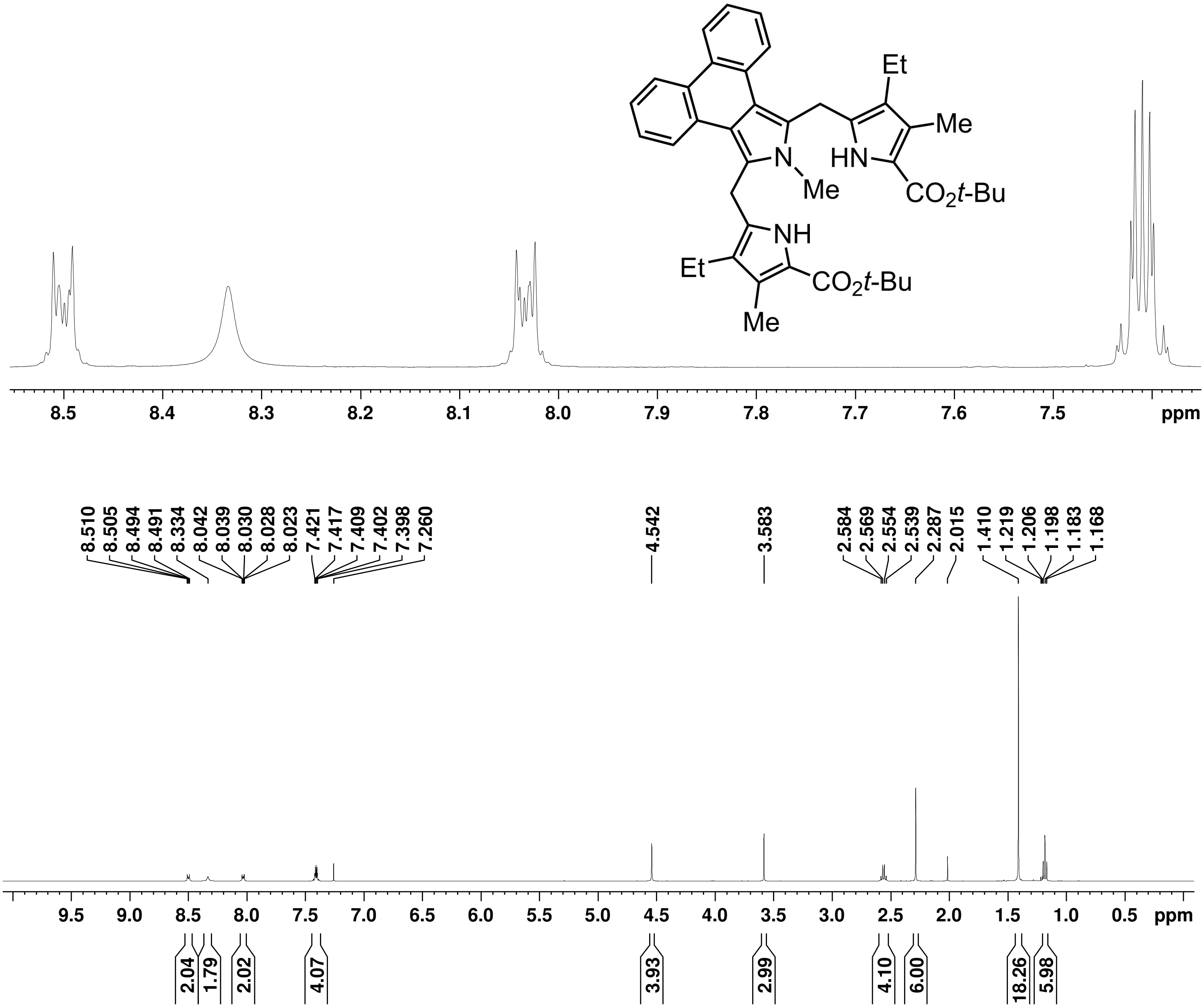

Figure S47. 500 MHz proton NMR spectrum of phenanthrotripyrrane **21** in $CDCl_3$.

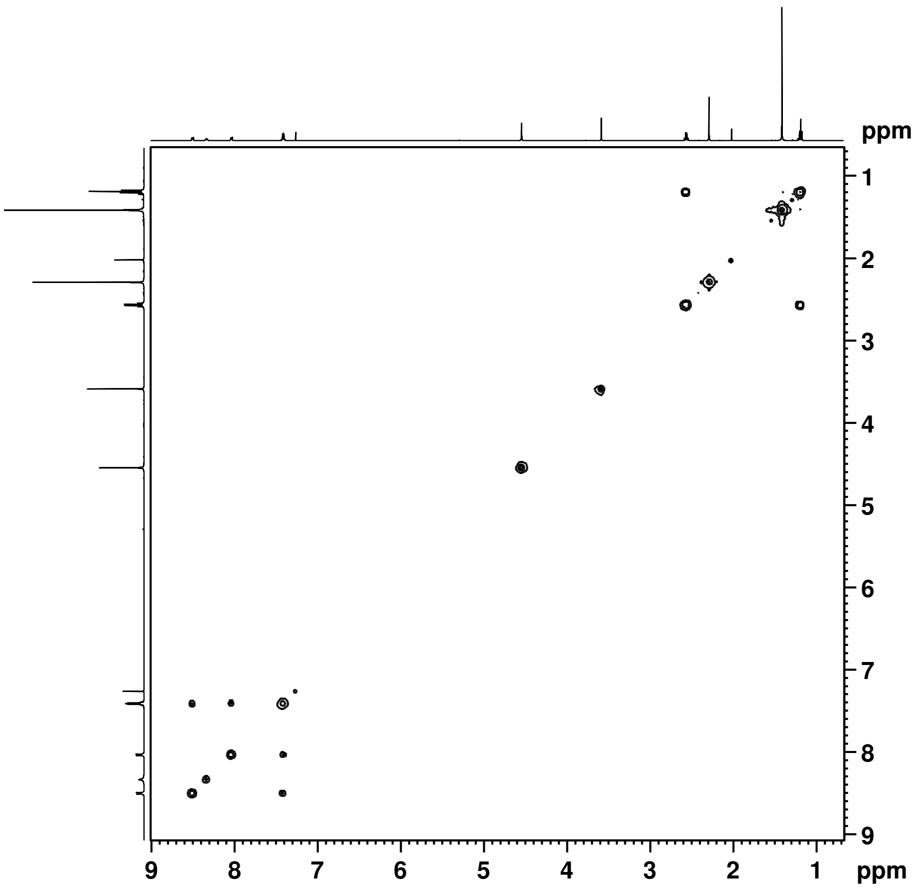

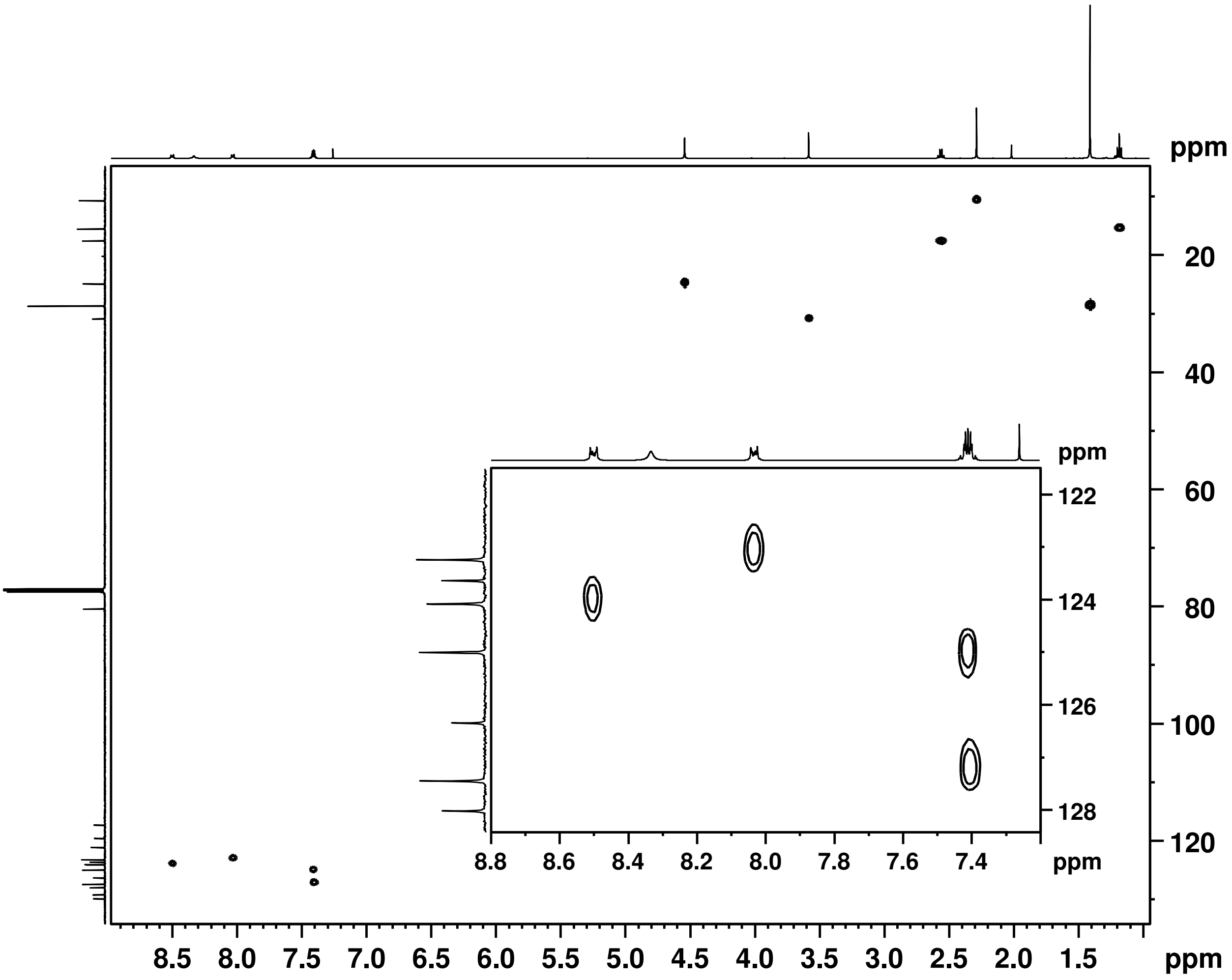

Figure S48. 1H-1H COSY (top) and HSQC (bottom) NMR spectra of phenanthrotripyrrane **21** in $CDCl_3$.

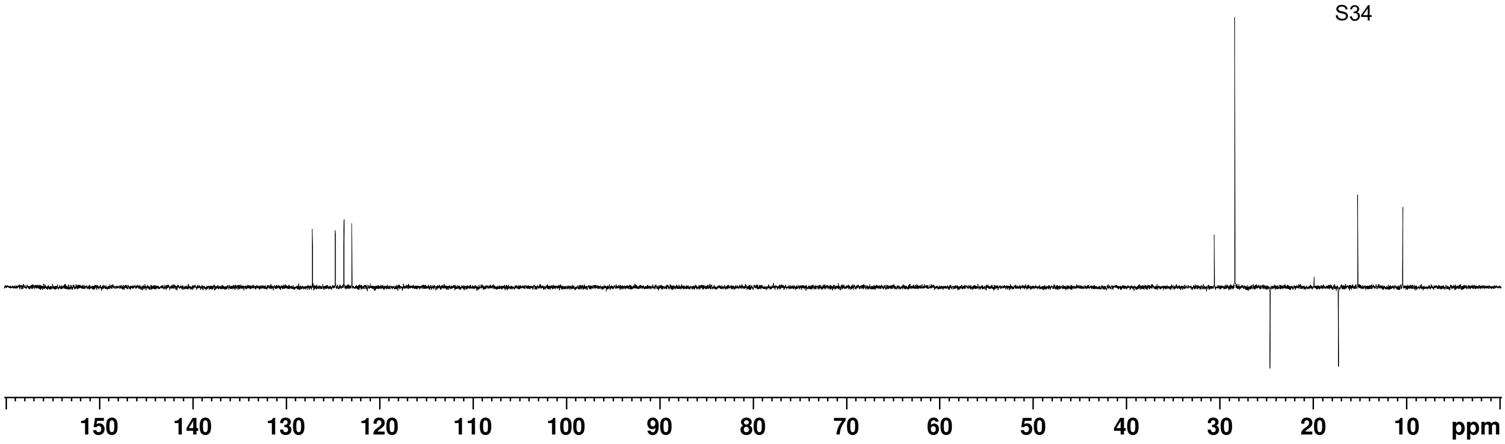

Figure S49. DEPT-135 NMR spectrum of phenanthrotripyrrane **21** in $CDCl_3$.

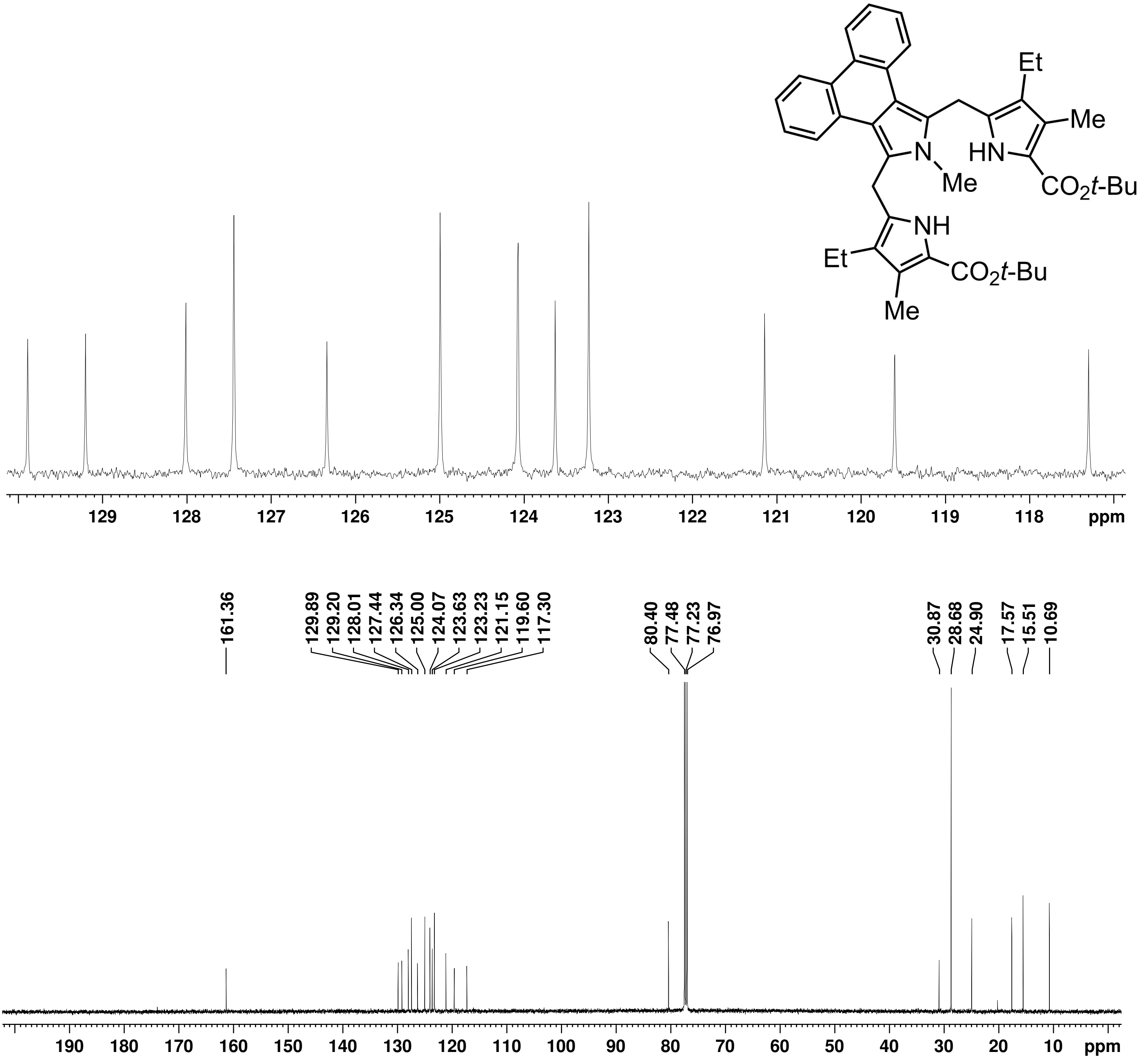

Figure S50. 125 MHz carbon-13 NMR spectrum of phenanthrotripyrrane **21** in $CDCl_3$.

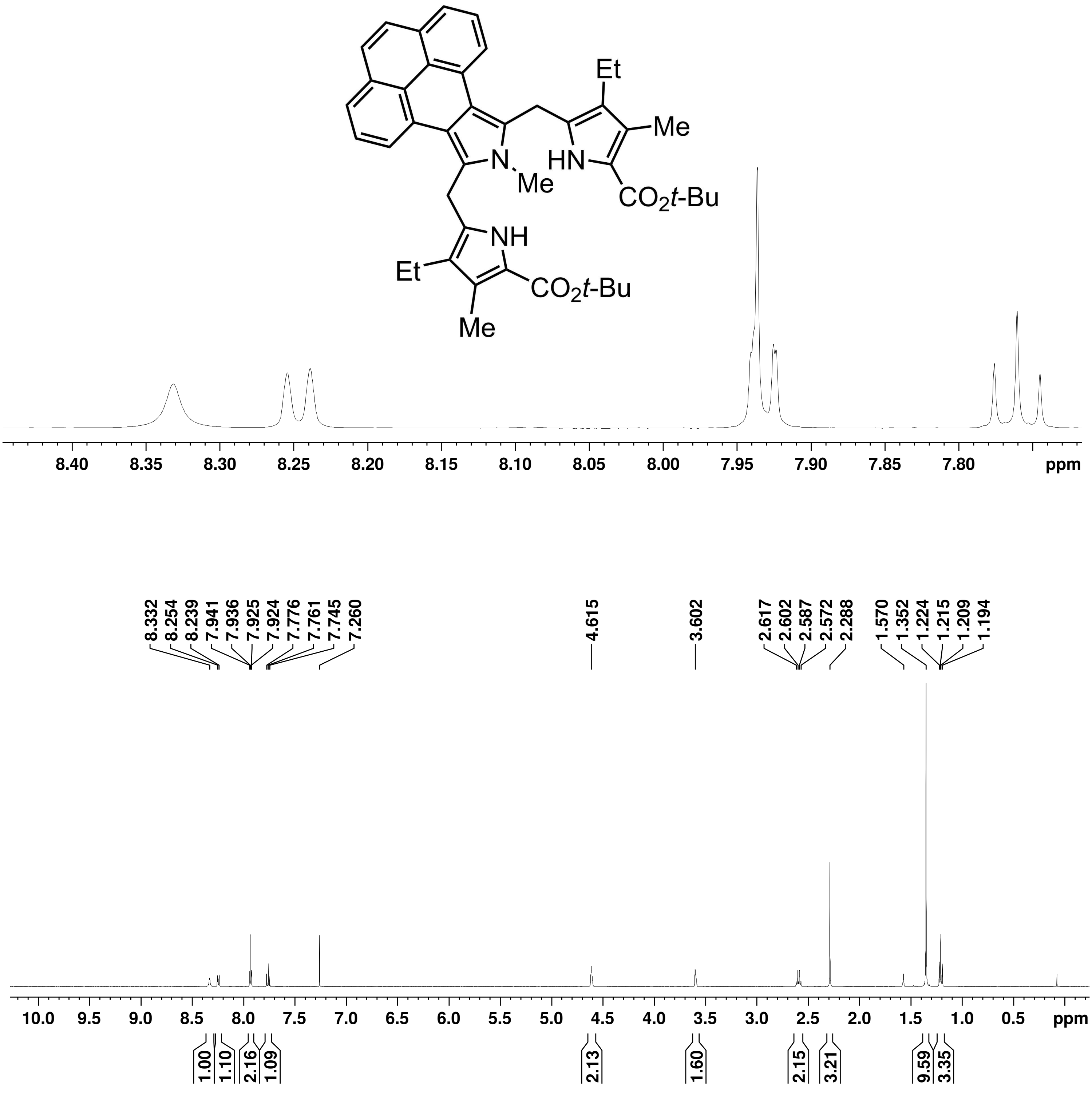

Figure S51. 500 MHz proton NMR spectrum of pyrenotripyrrane **22** in $CDCl_3$.

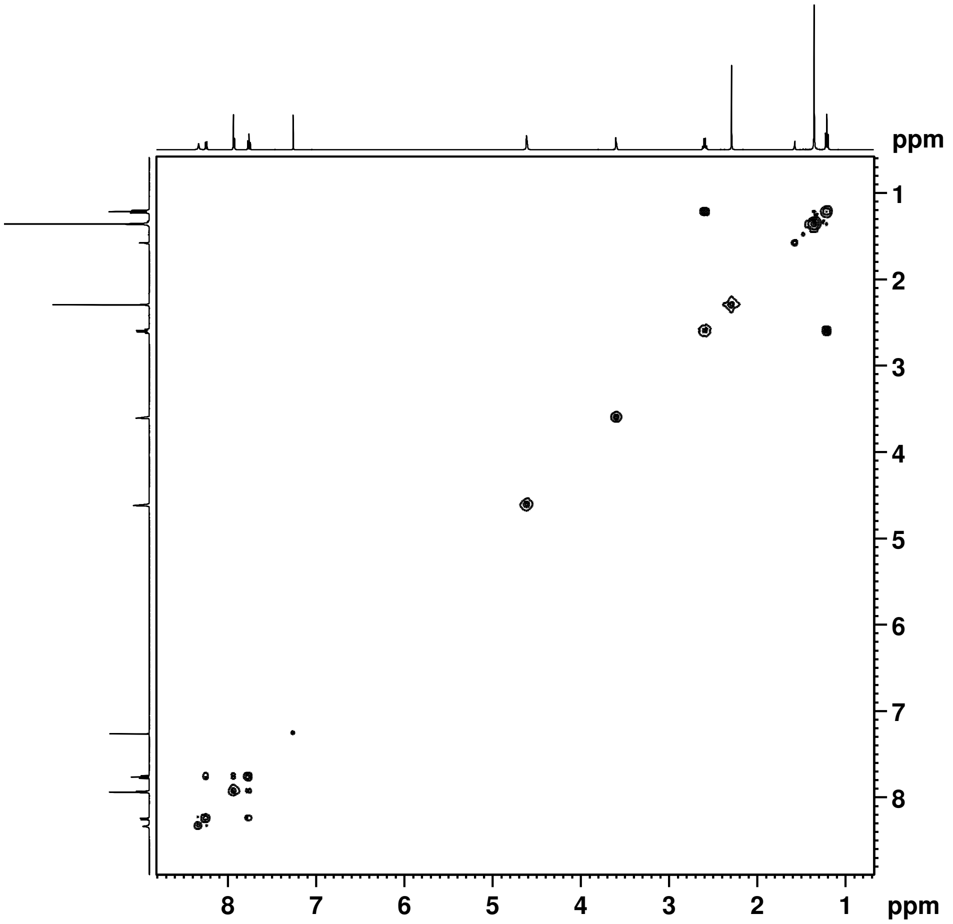

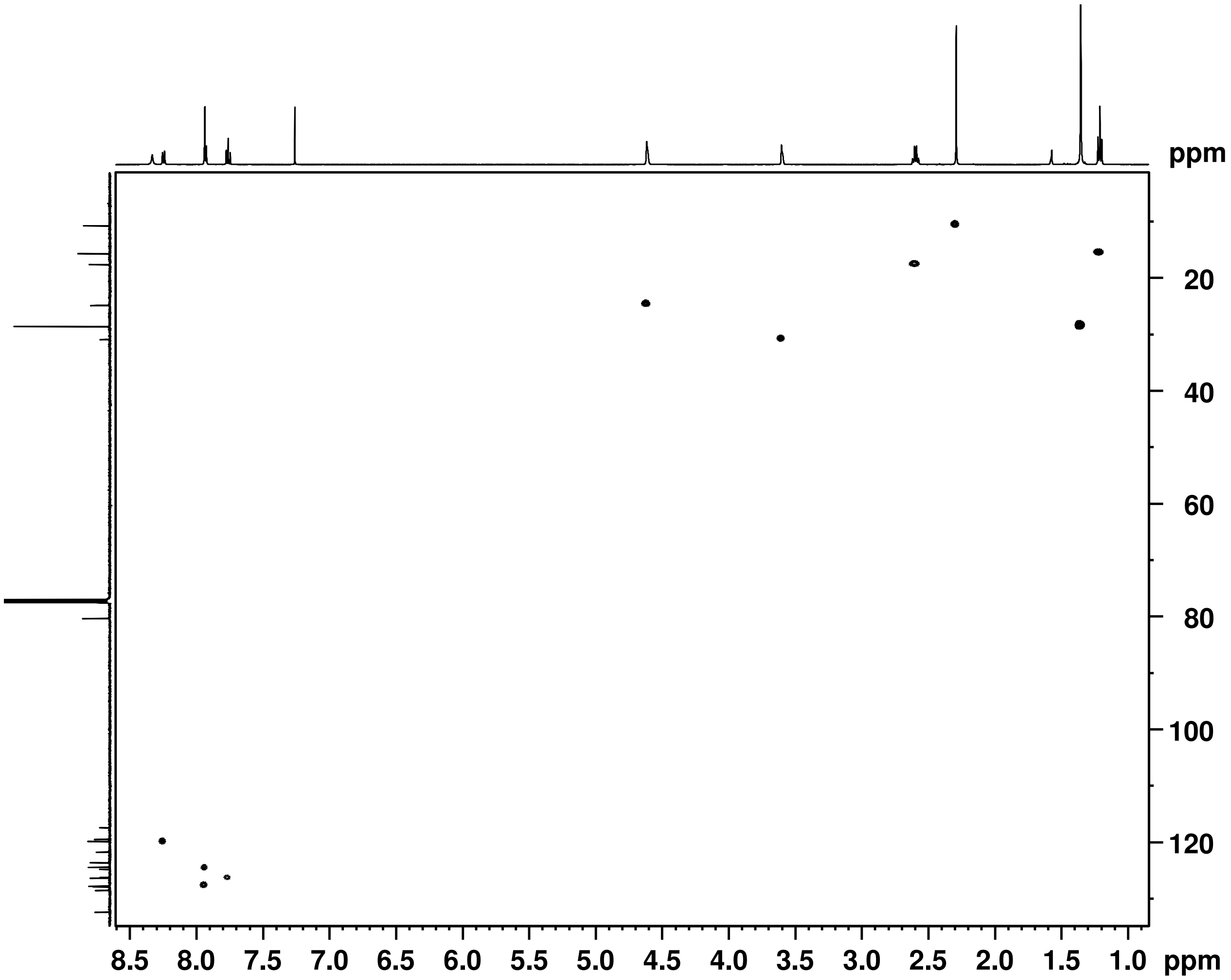

Figure S52. ^{1}H-^{1}H COSY (top) and HSQC (bottom) NMR spectra of pyrenotripyrrane **22** in $CDCl_3$.

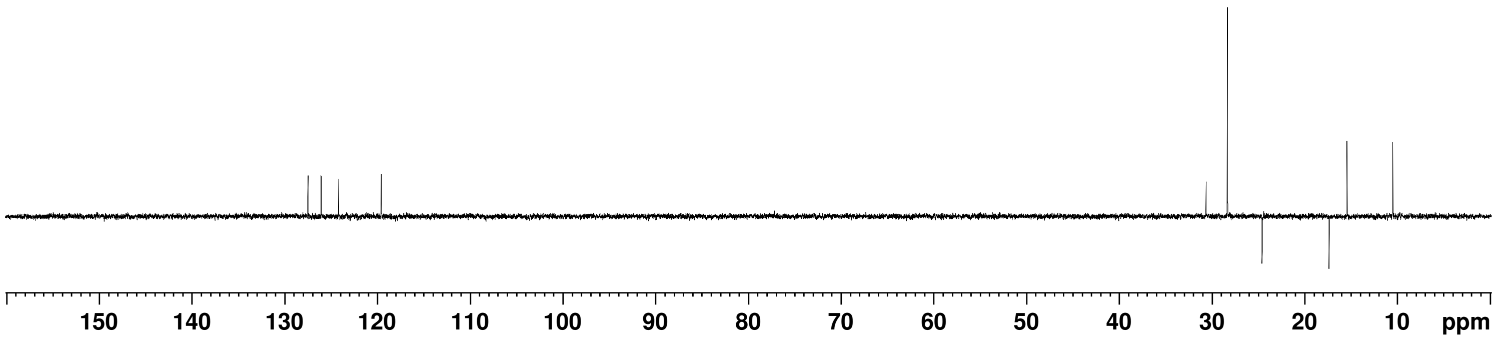

Figure S53. DEPT-135 NMR spectrum of pyrenotripyrrane **22** in $CDCl_3$.

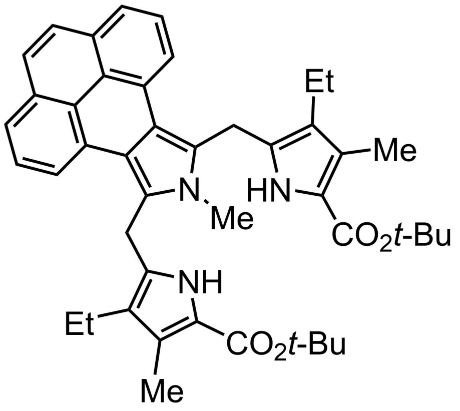

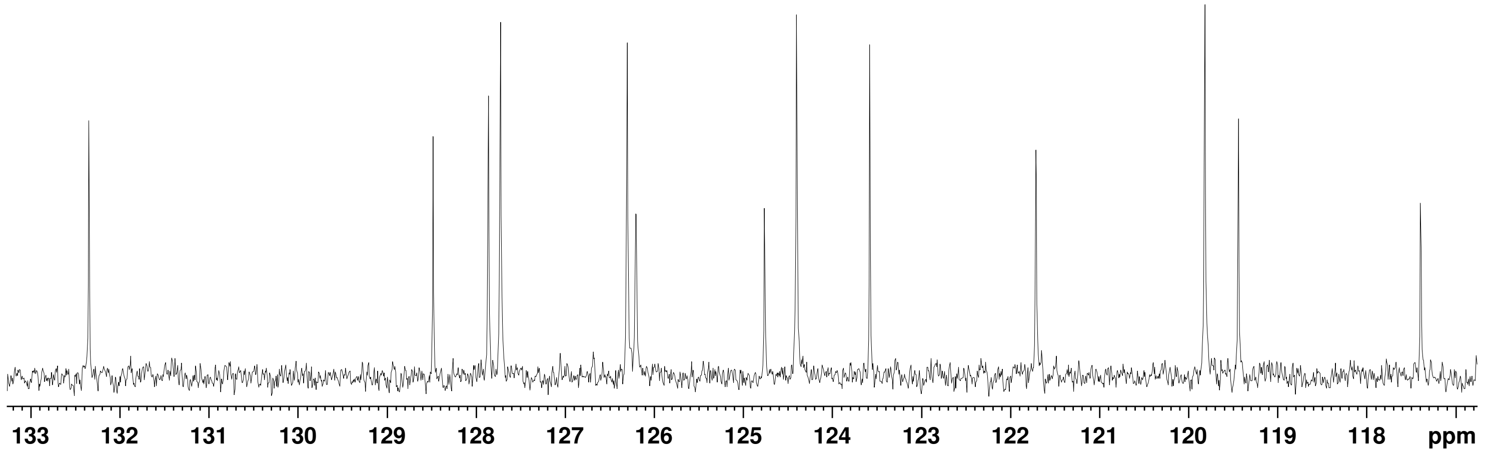

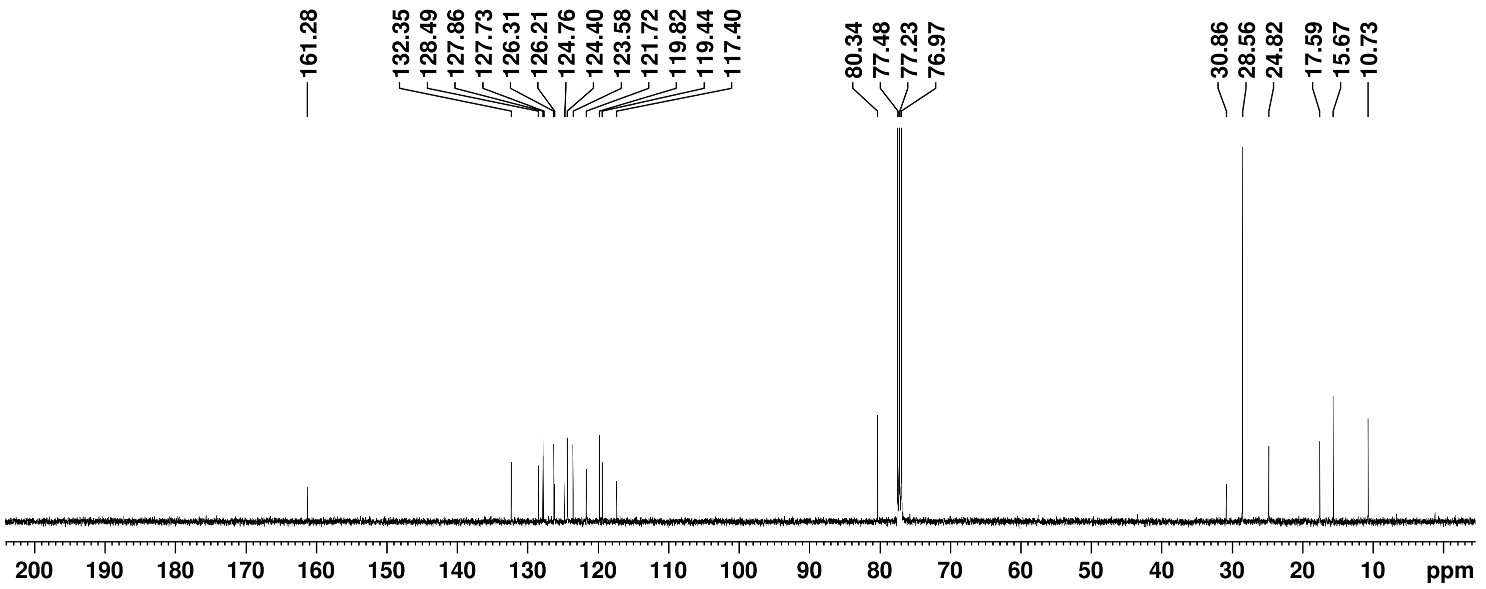

Figure S54. 125 MHz carbon-13 NMR spectrum of pyrenotripyrrane **22** in $CDCl_3$.

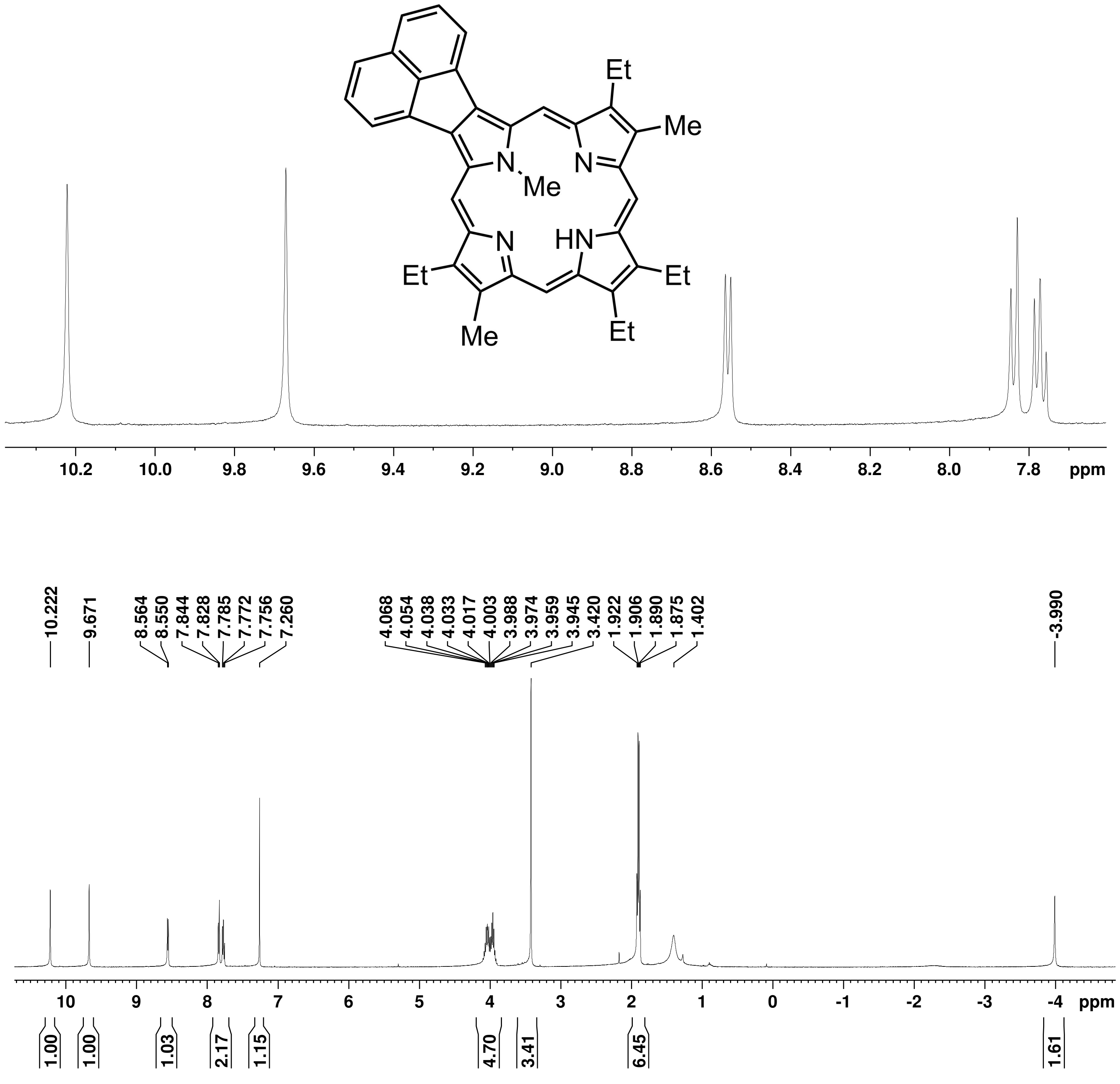

Figure S55. 500 MHz proton NMR spectrum of *N*-methylacenaphthoporphyrin **6** in $CDCl_3$ at 29 ^oC.

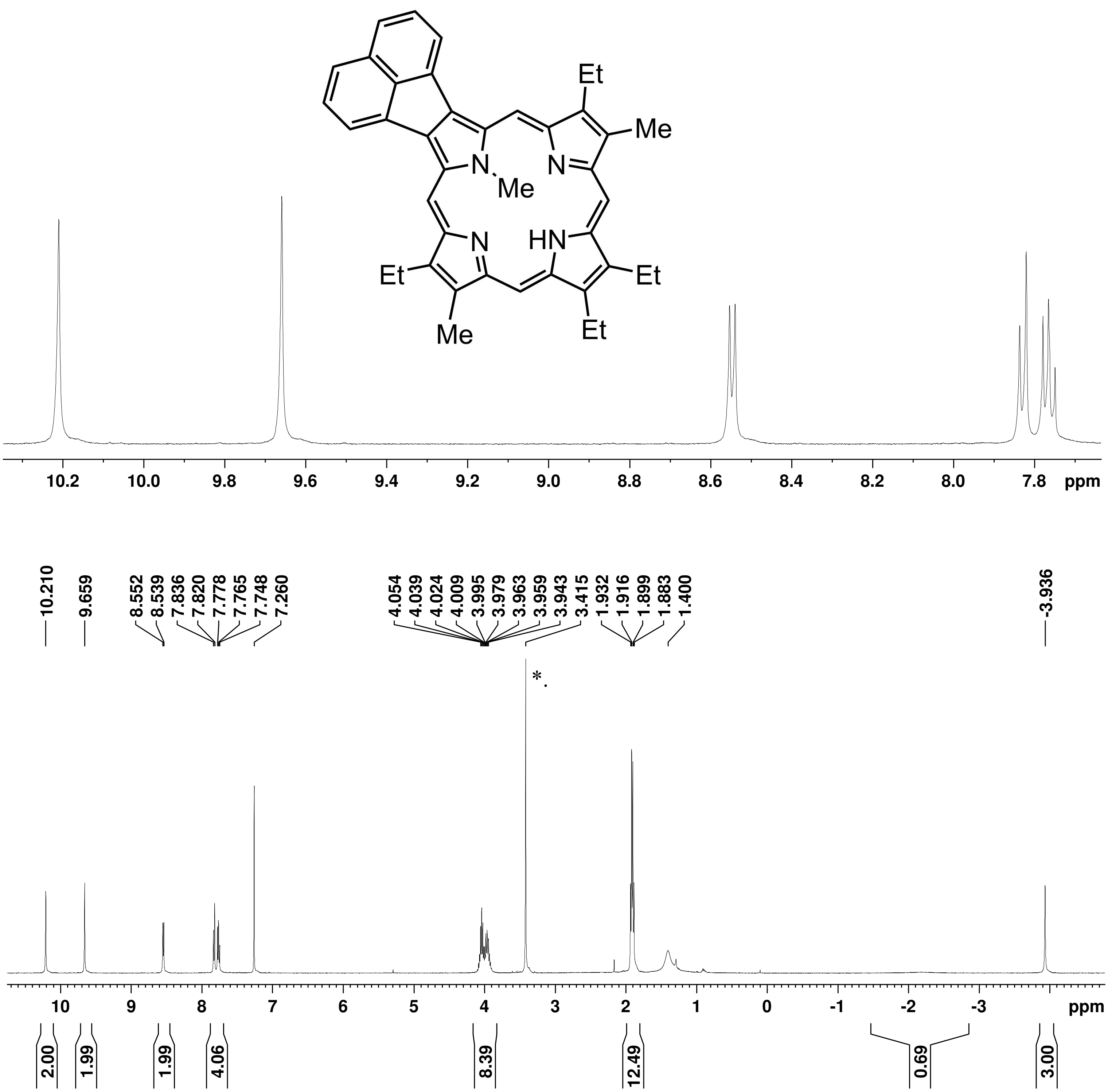

Figure S56. 500 MHz proton NMR spectrum of *N*-methylacenaphthoporphyrin **6** in $CDCl_3$ at 50 ^oC.

* = solvent impurity

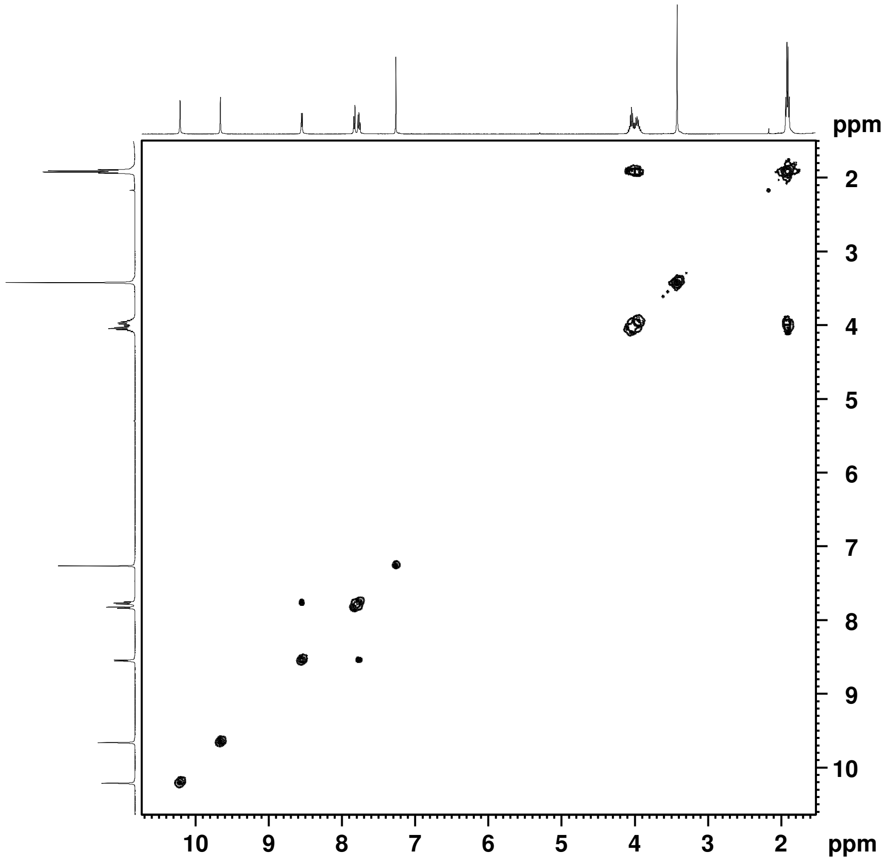

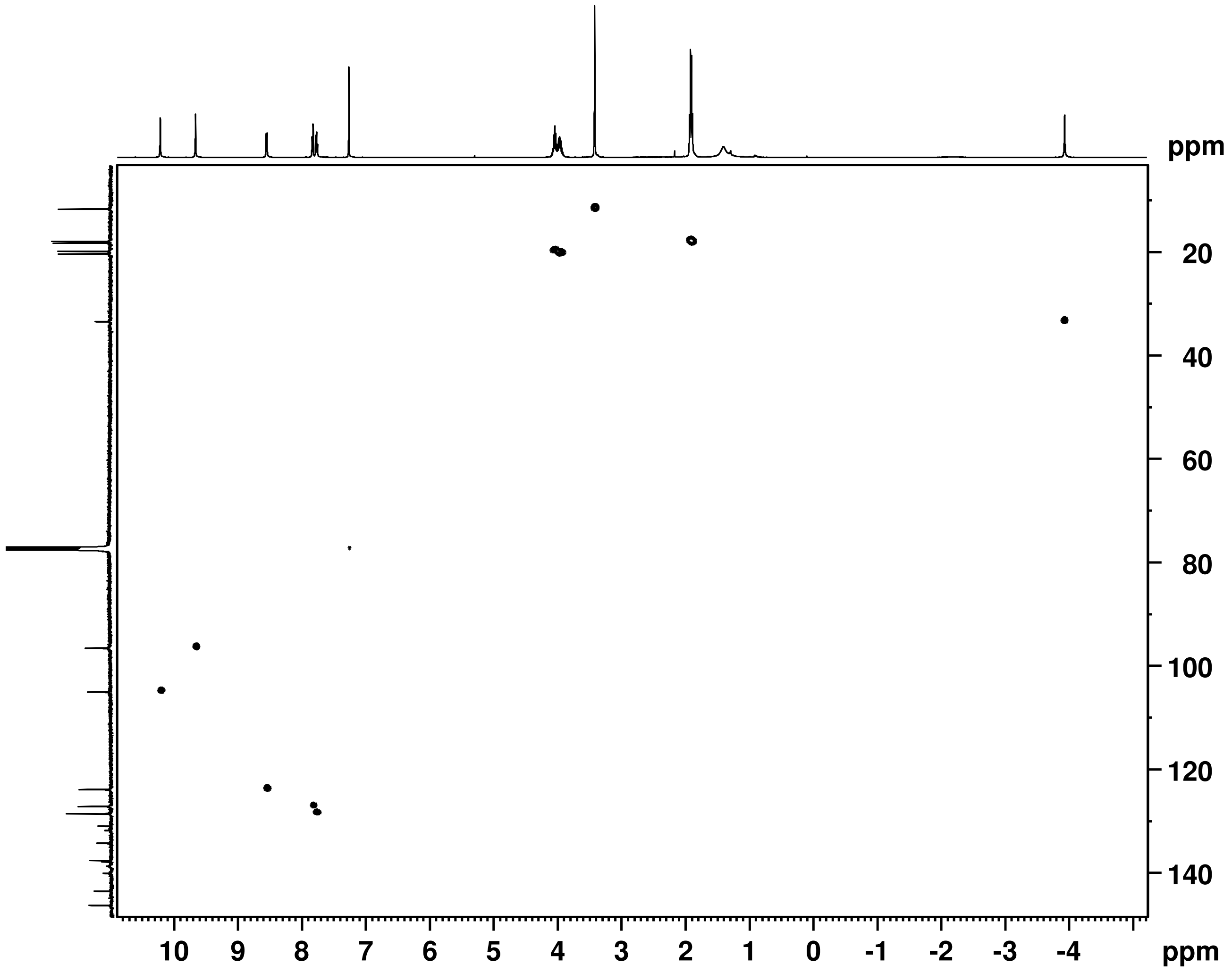

Figure S57. ^{1}H-^{1}H COSY (top) and HSQC (bottom) NMR spectra of **6** in $CDCl_3$ at 50 °C.

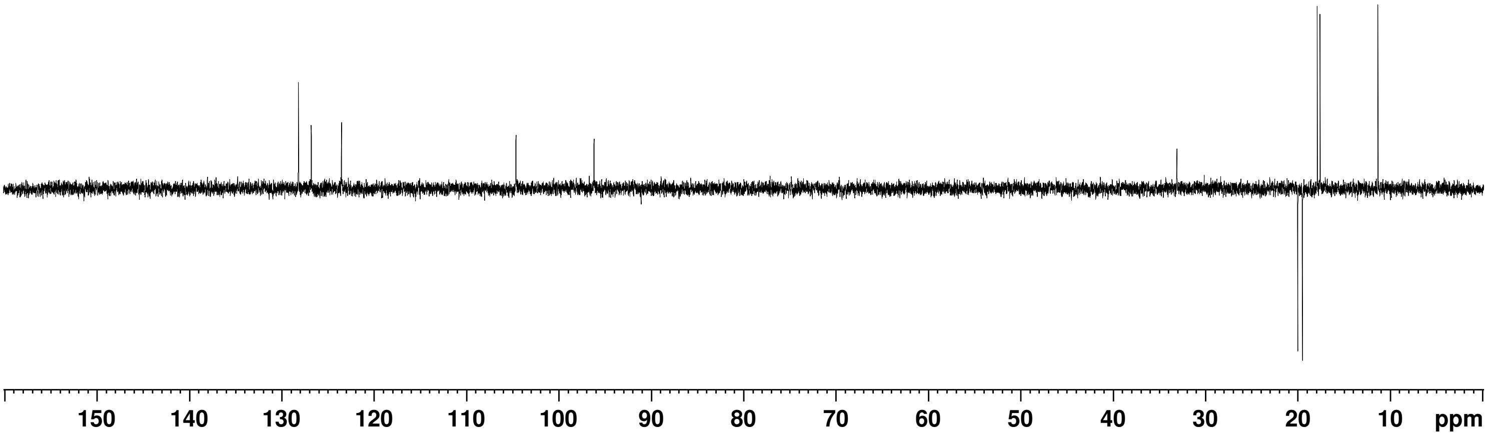

Figure S58. DEPT-135 NMR spectrum of *N*-methylacenaphthoporphyrin **6** in $CDCl_3$ at 50 °C.

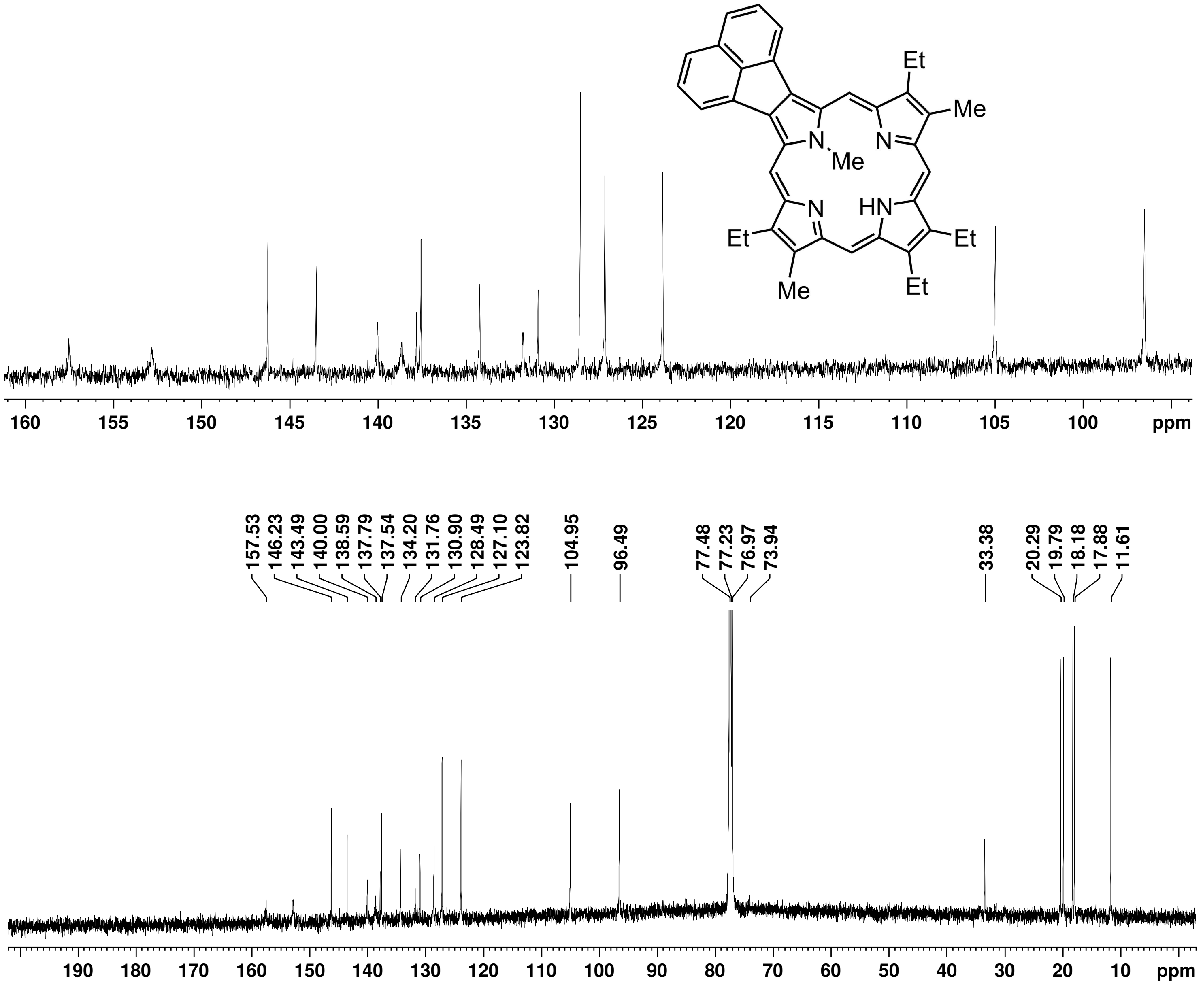

Figure S59. 125 MHz carbon-13 NMR spectrum of acenaphthoporphyrin **6** in $CDCl_3$ at 50 °C.

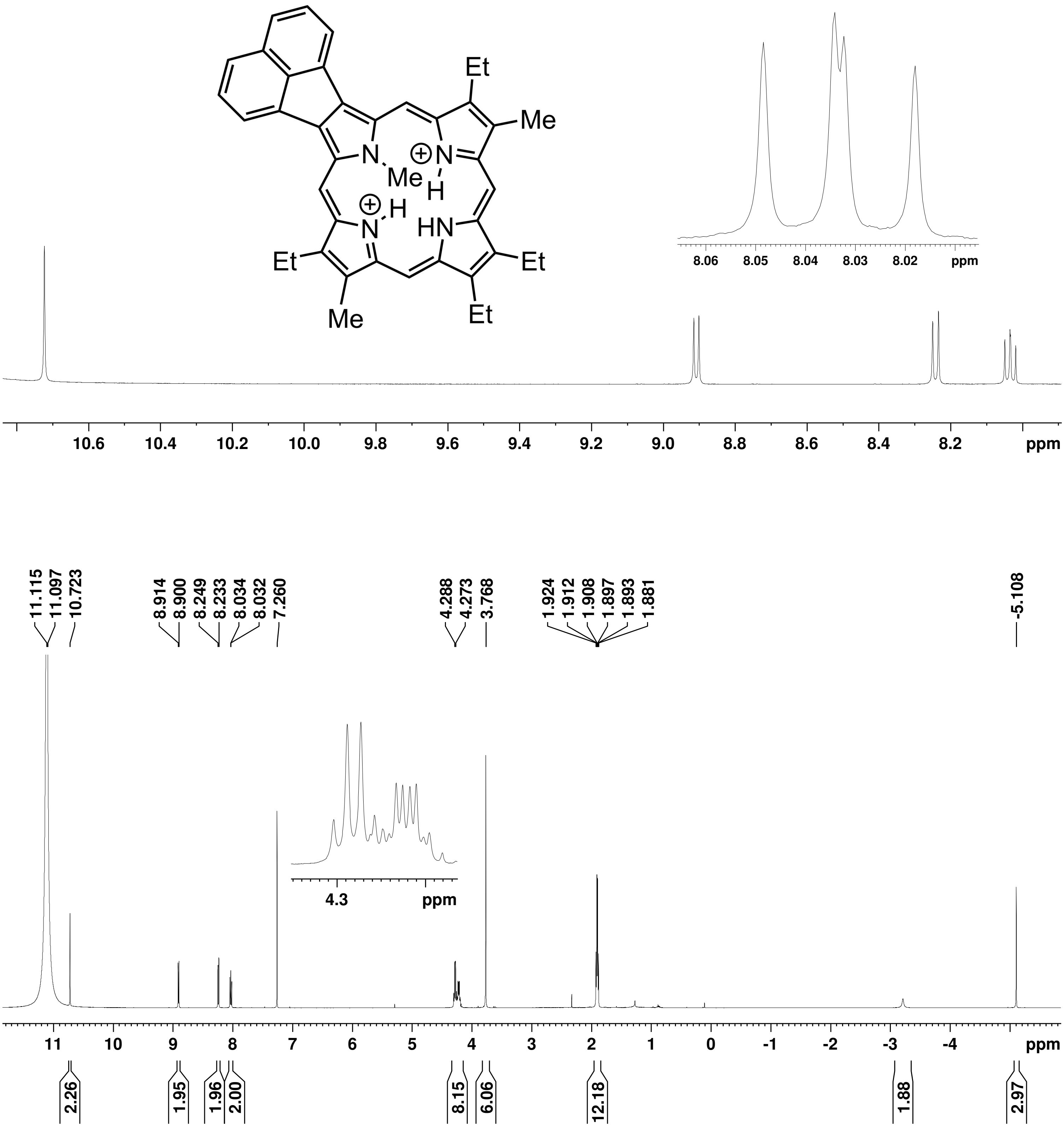

Figure S60. 500 MHz proton NMR spectrum of acenaphthoporphyrin dication $\mathbf{6}H_2^{2+}$ in TFA-CDCl$_3$.

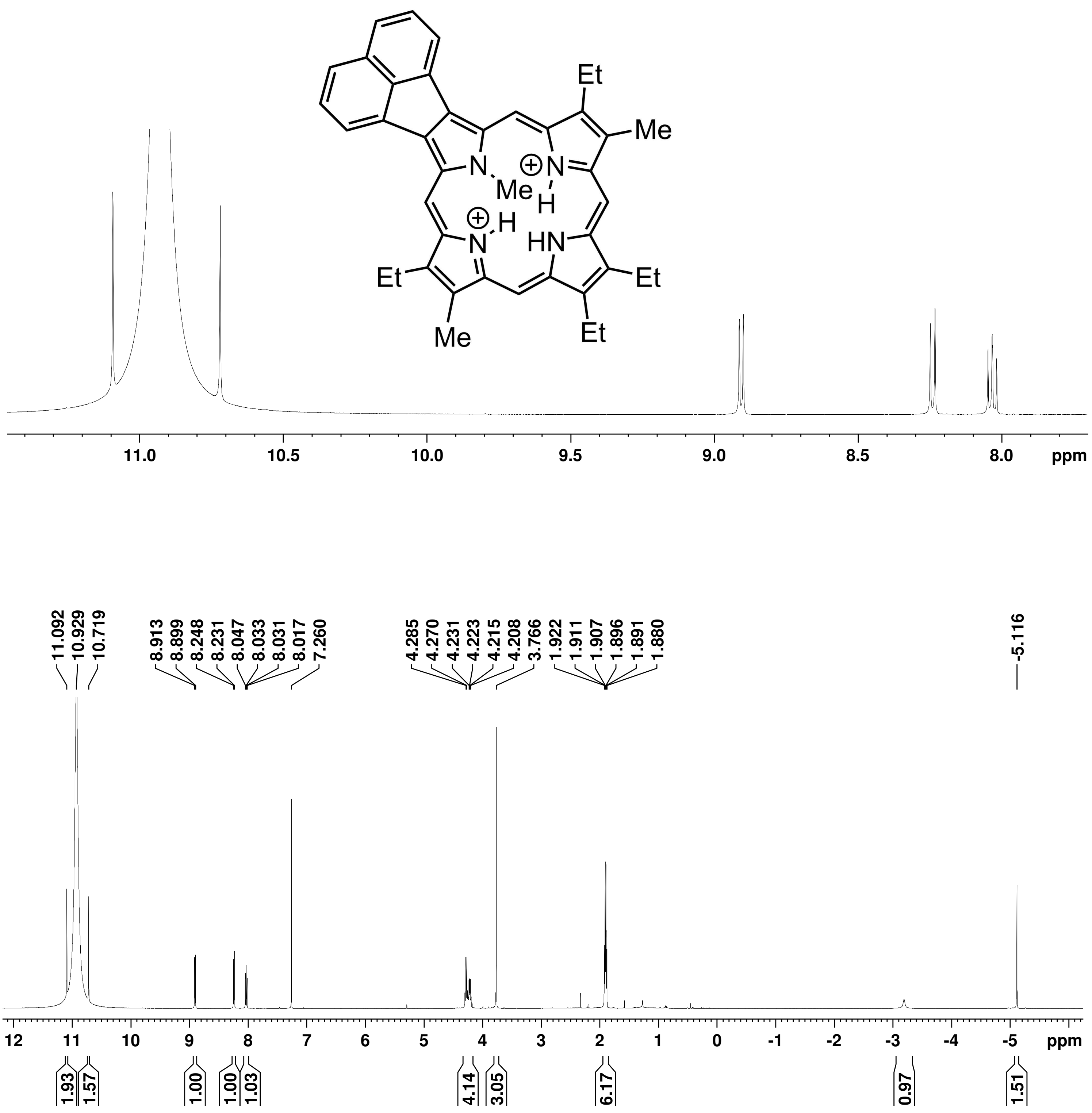

Figure S61. 500 MHz proton NMR spectrum of acenaphthoporphyrin dication **6**H_2^{2+} in TFA-$CDCl_3$. This spectrum differs from Figure S60 due to the further addition of TFA and this allows both of the *meso*-proton resonances to be identified.

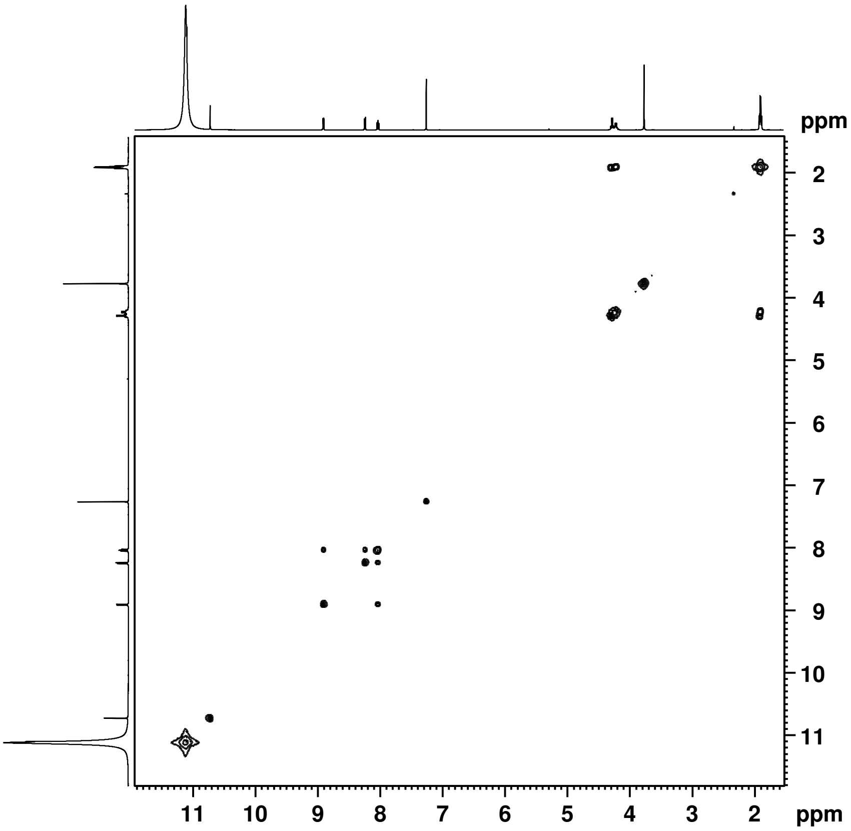

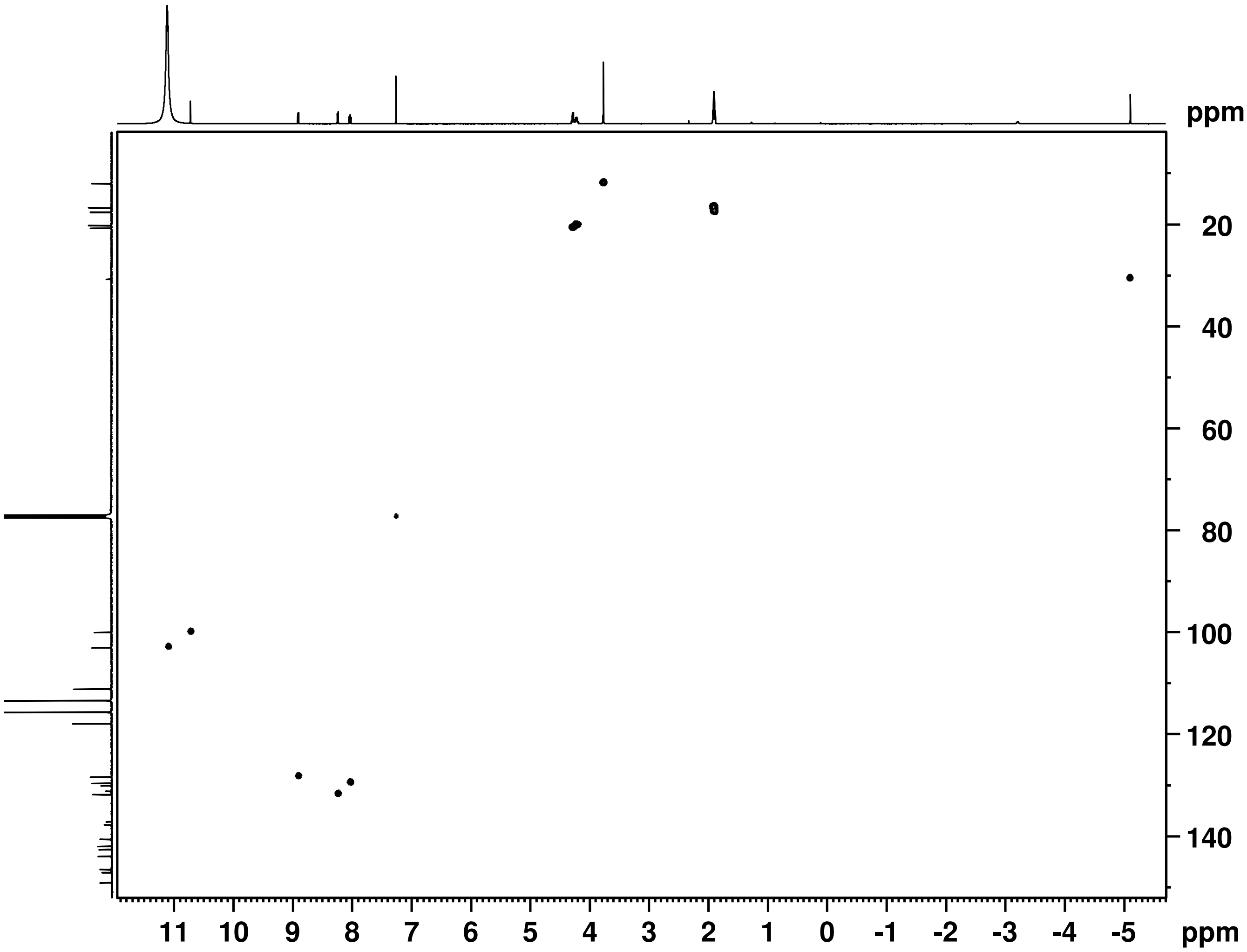

Figure S62. ^{1}H-^{1}H COSY (top) and HSQC (bottom) NMR spectra of **6**H_2^{2+} in TFA-$CDCl_3$.

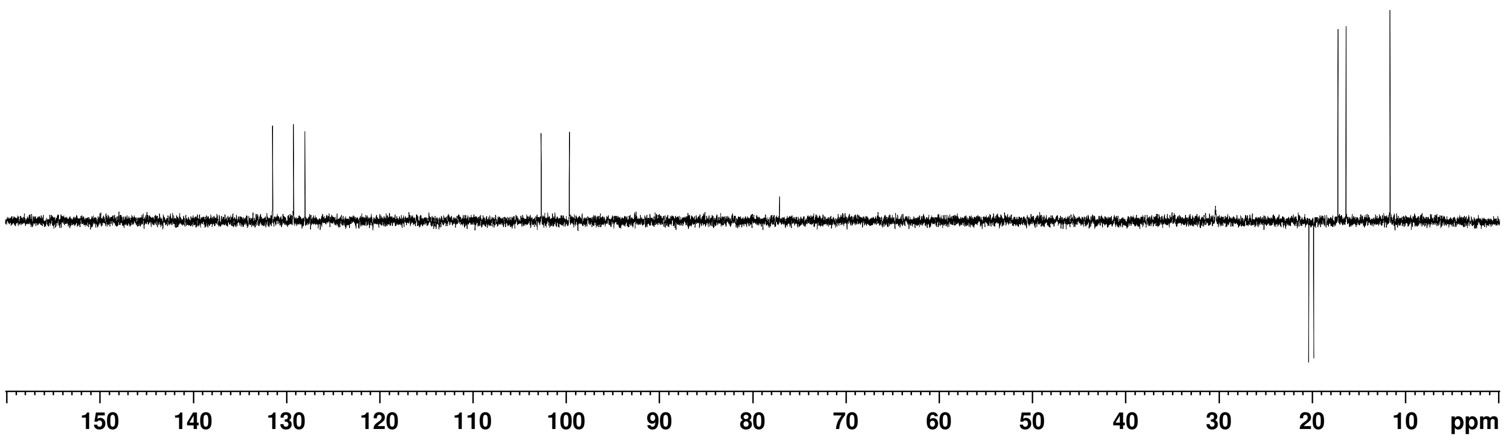

Figure S63. DEPT-135 NMR spectrum of acenaphthoporphyrin dication **6**H_2^{2+} in TFA-$CDCl_3$.

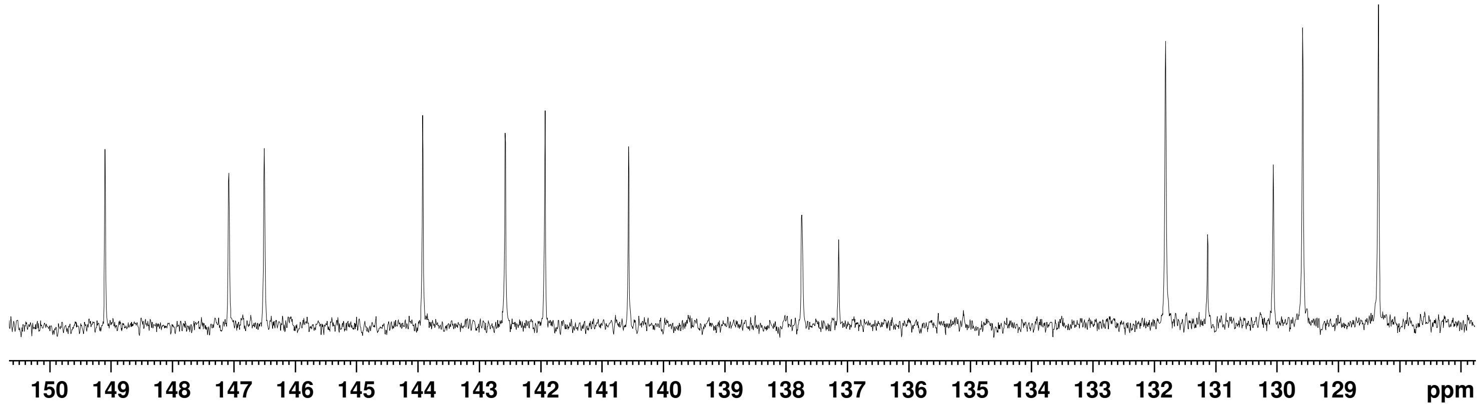

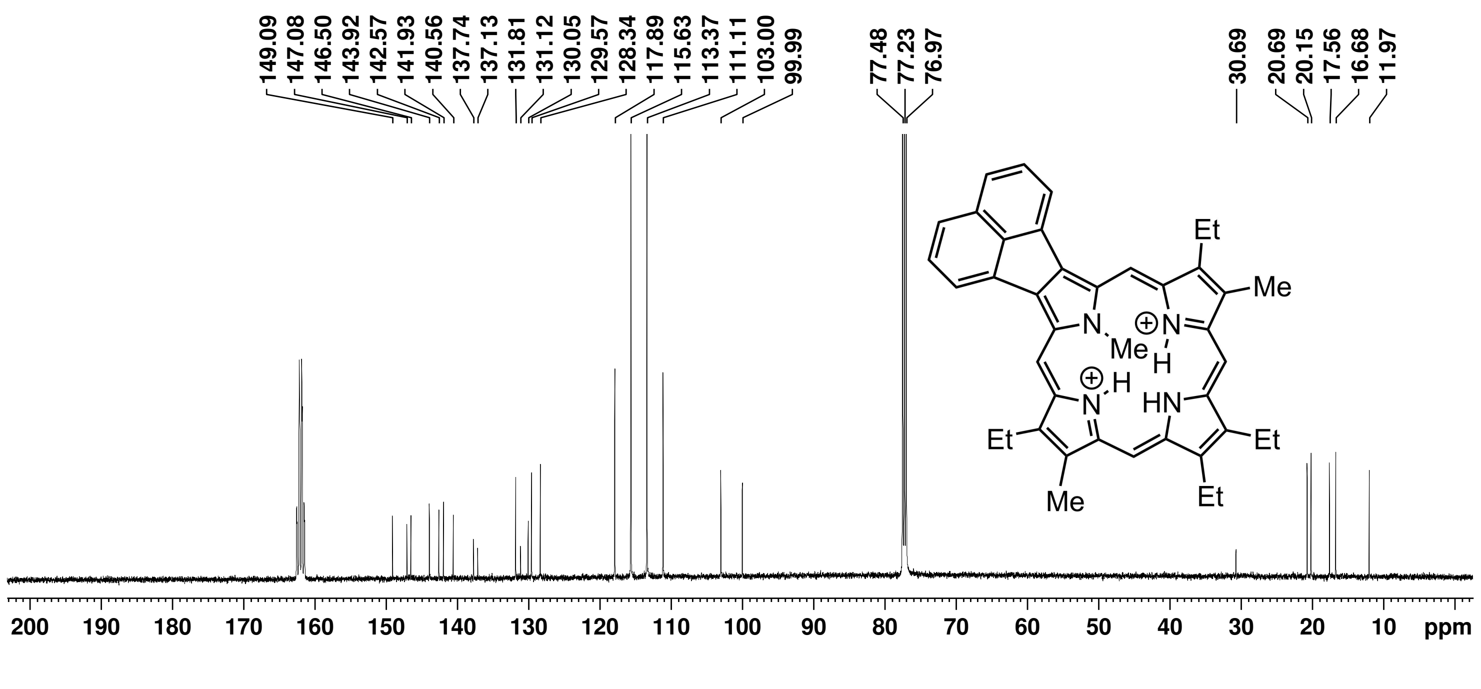

Figure S64. 125 MHz carbon-13 NMR spectrum of acenaphthoporphyrin dication **6**H_2^{2+} in TFA-$CDCl_3$.

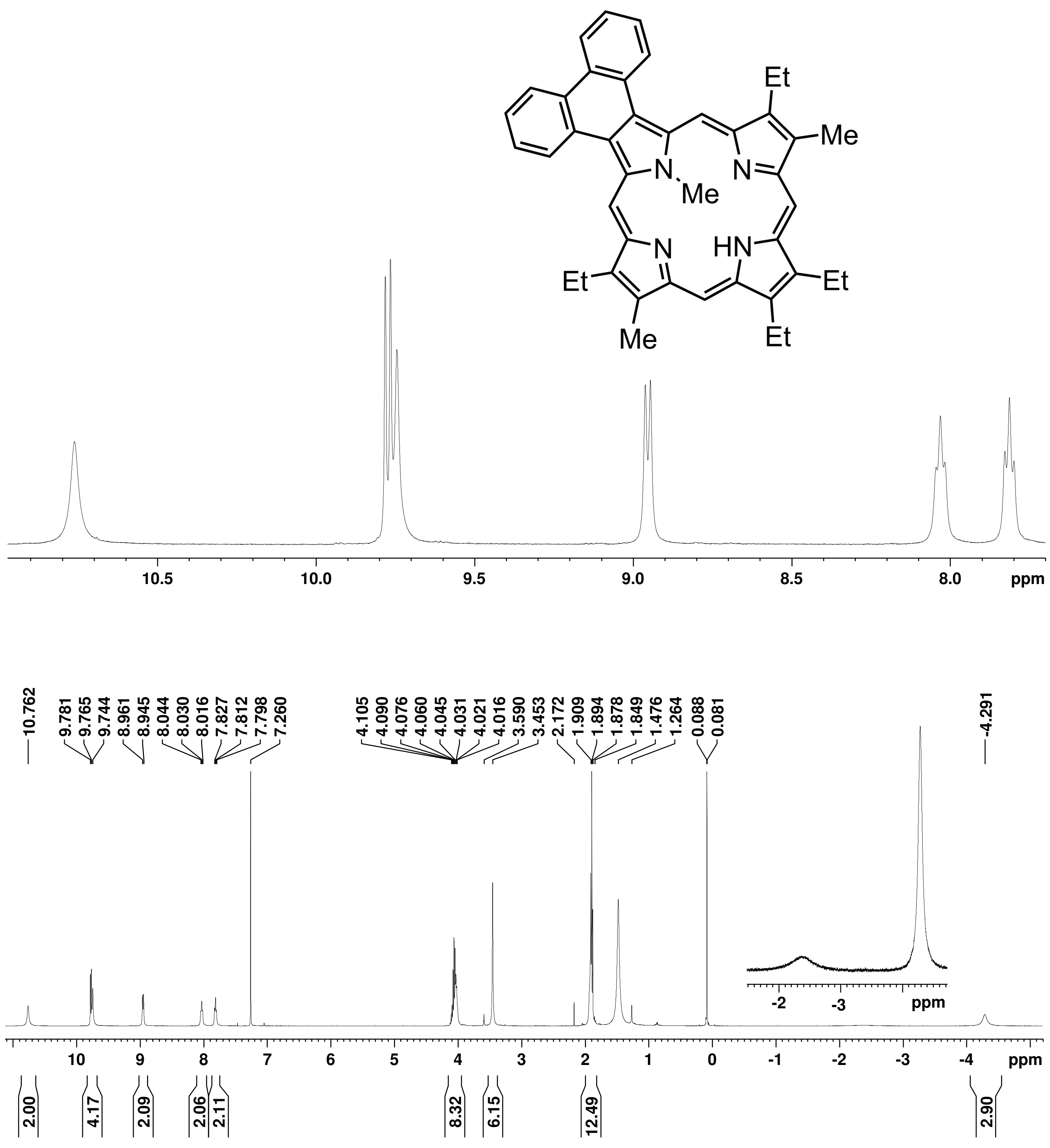

Figure S65. 500 MHz proton NMR spectrum of *N*-methylphenanthroporphyrin **7** in $CDCl_3$ at 29 °C.

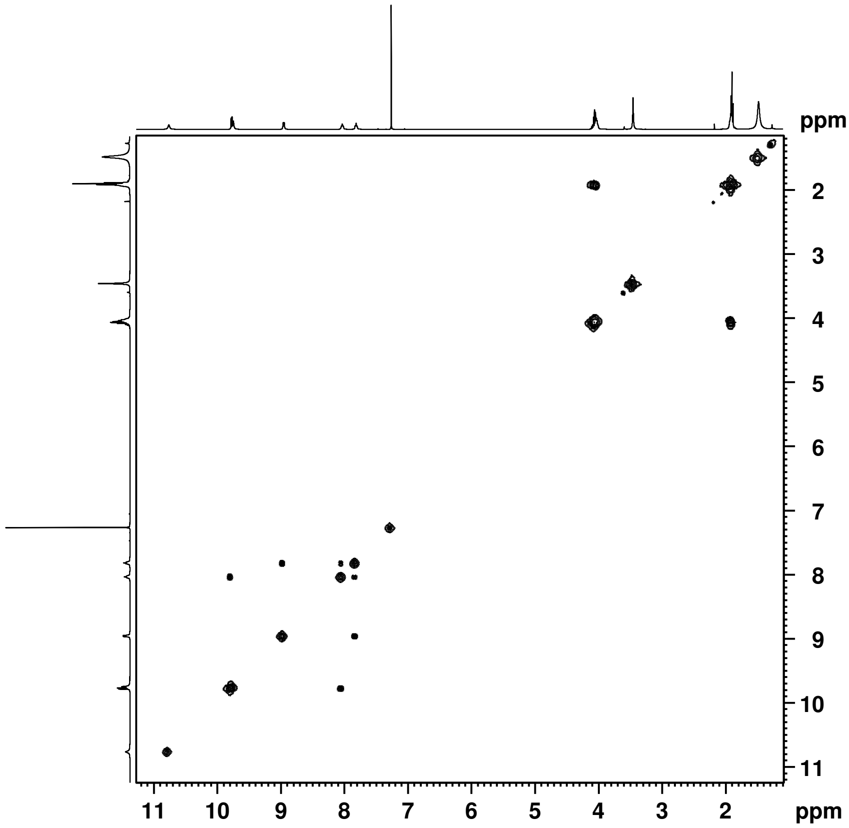

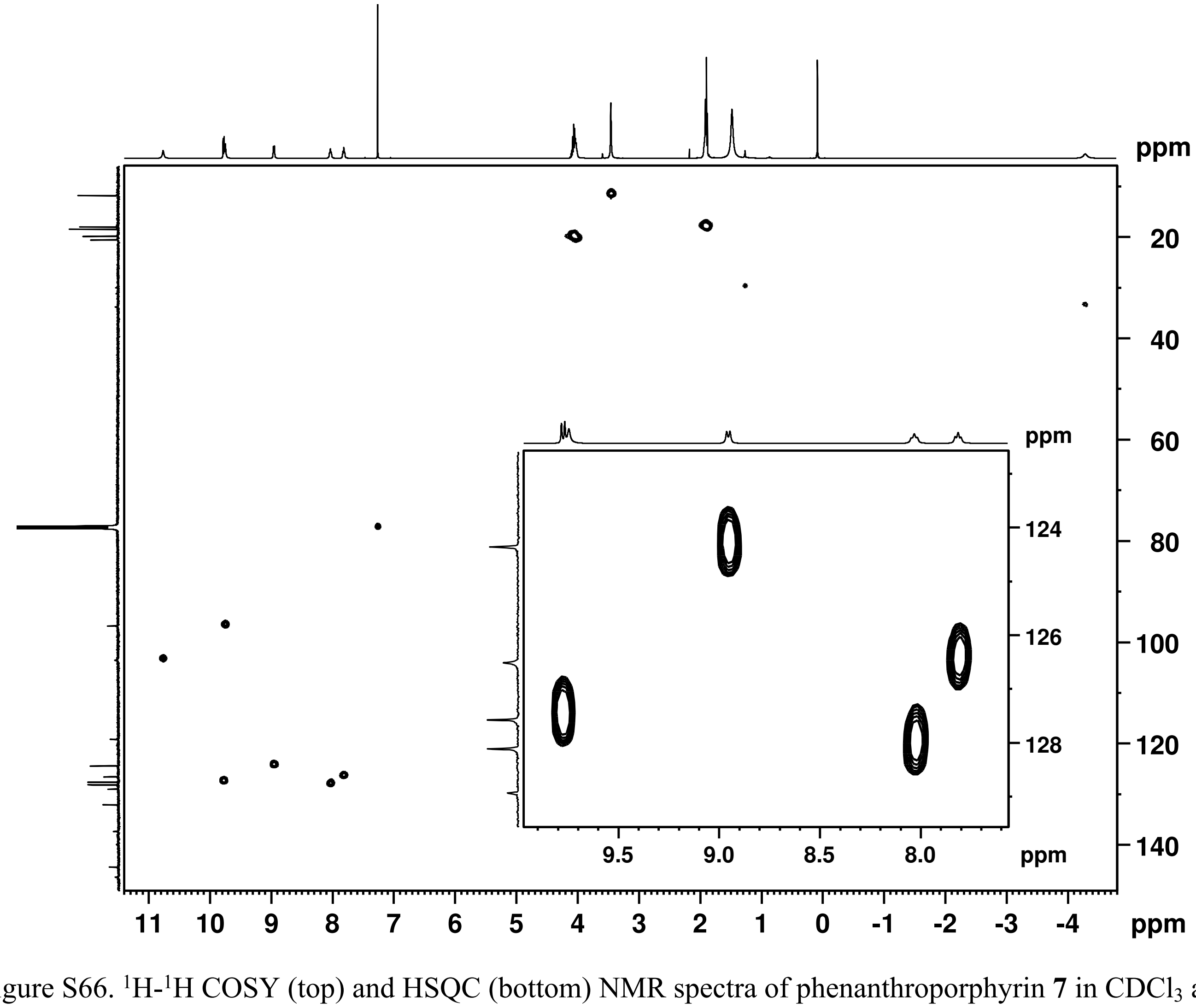

Figure S66. ^{1}H-^{1}H COSY (top) and HSQC (bottom) NMR spectra of phenanthroporphyrin **7** in $CDCl_3$ at 29 °C.

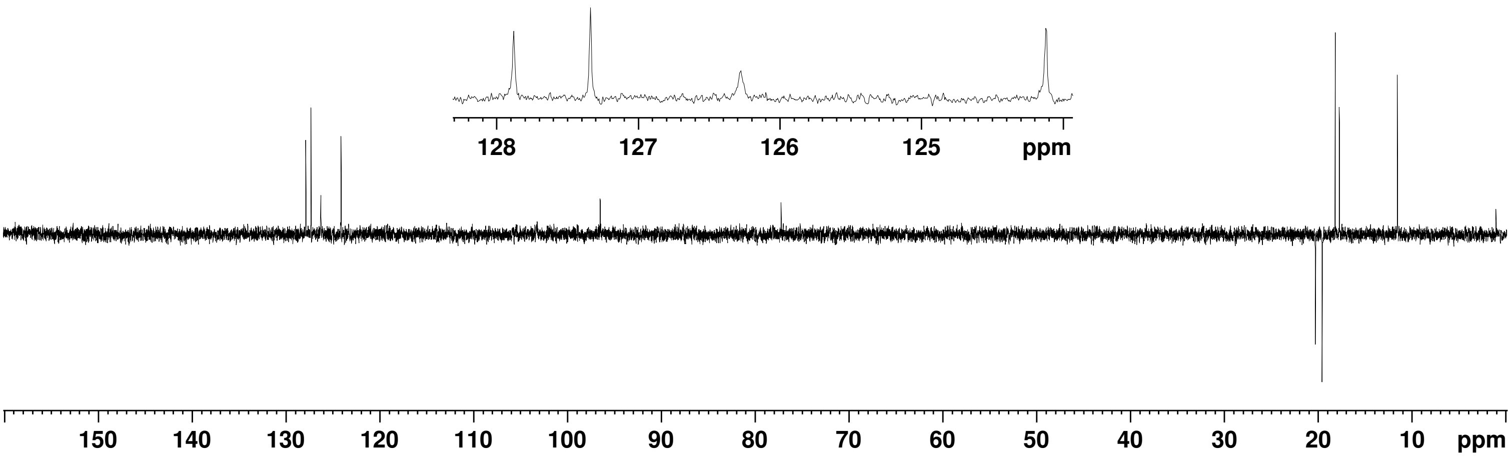

Figure S67. DEPT-135 NMR spectrum of phenanthroporphyrin **7** in $CDCl_3$ at 29 °C.

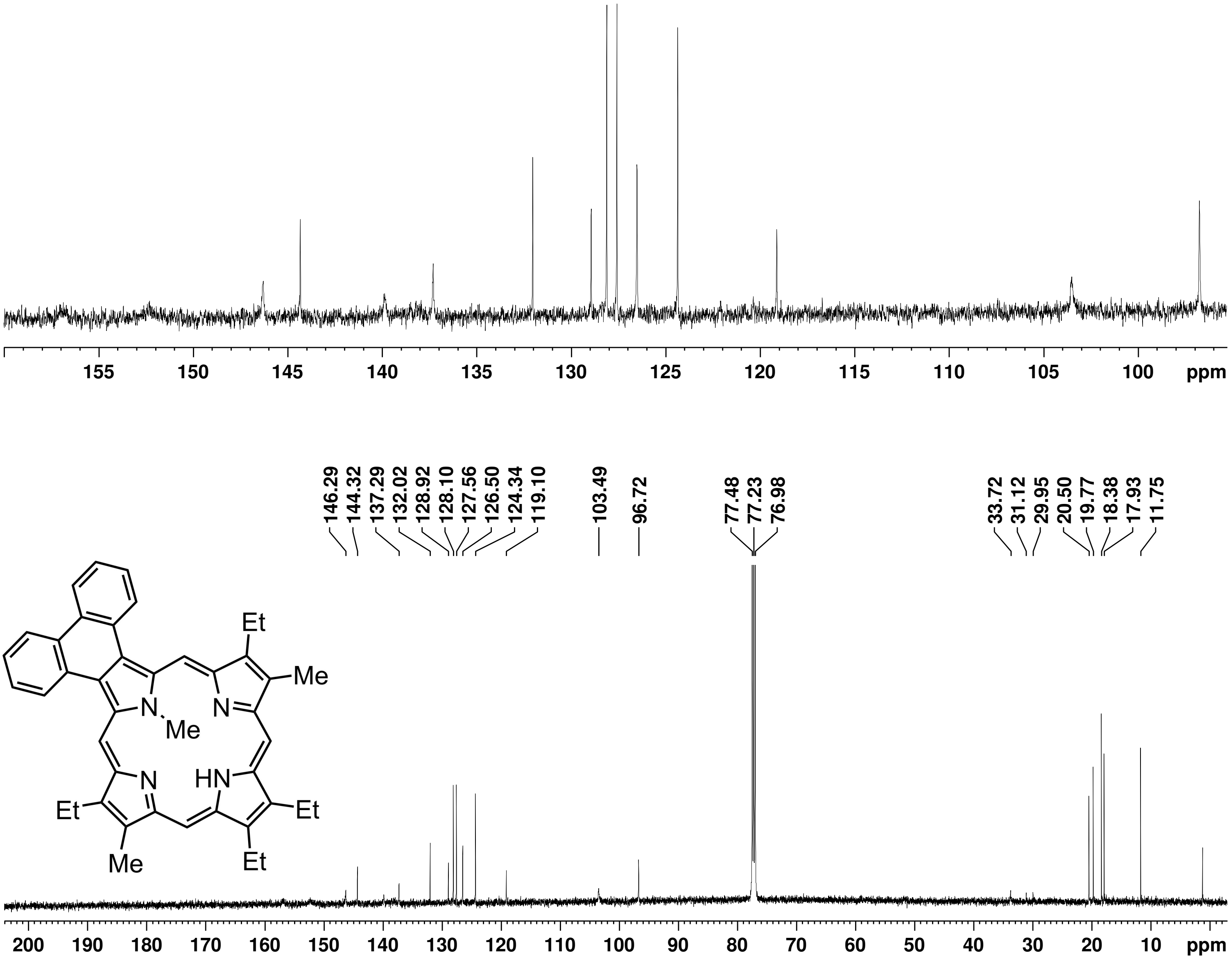

Figure S68. 125 MHz carbon-13 NMR spectrum of phenanthroporphyrin **7** in $CDCl_3$ at 29 °C.

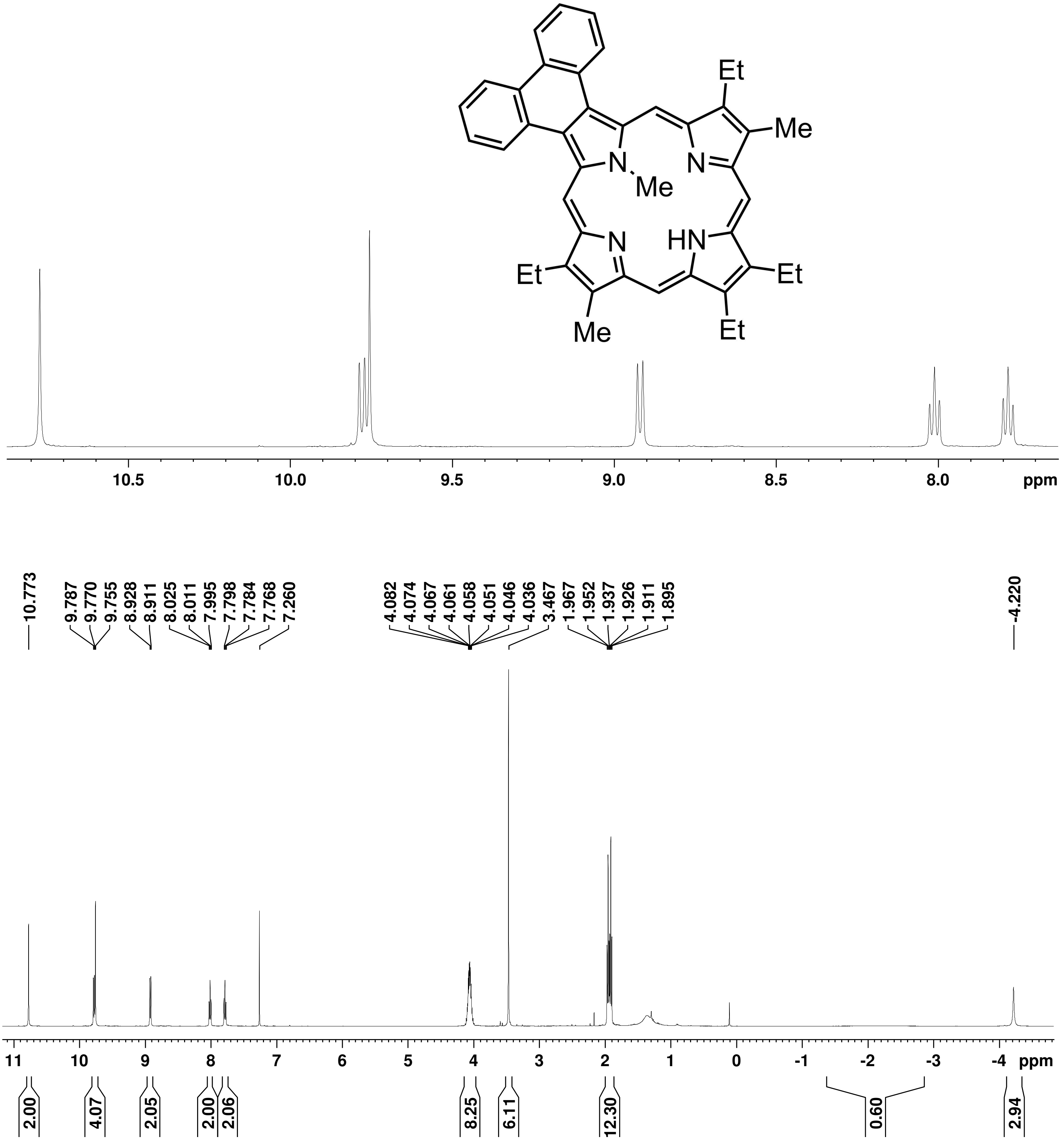

Figure S69. 500 MHz proton NMR spectrum of *N*-methylphenanthroporphyrin **7** in $CDCl_3$ at 50 °C.

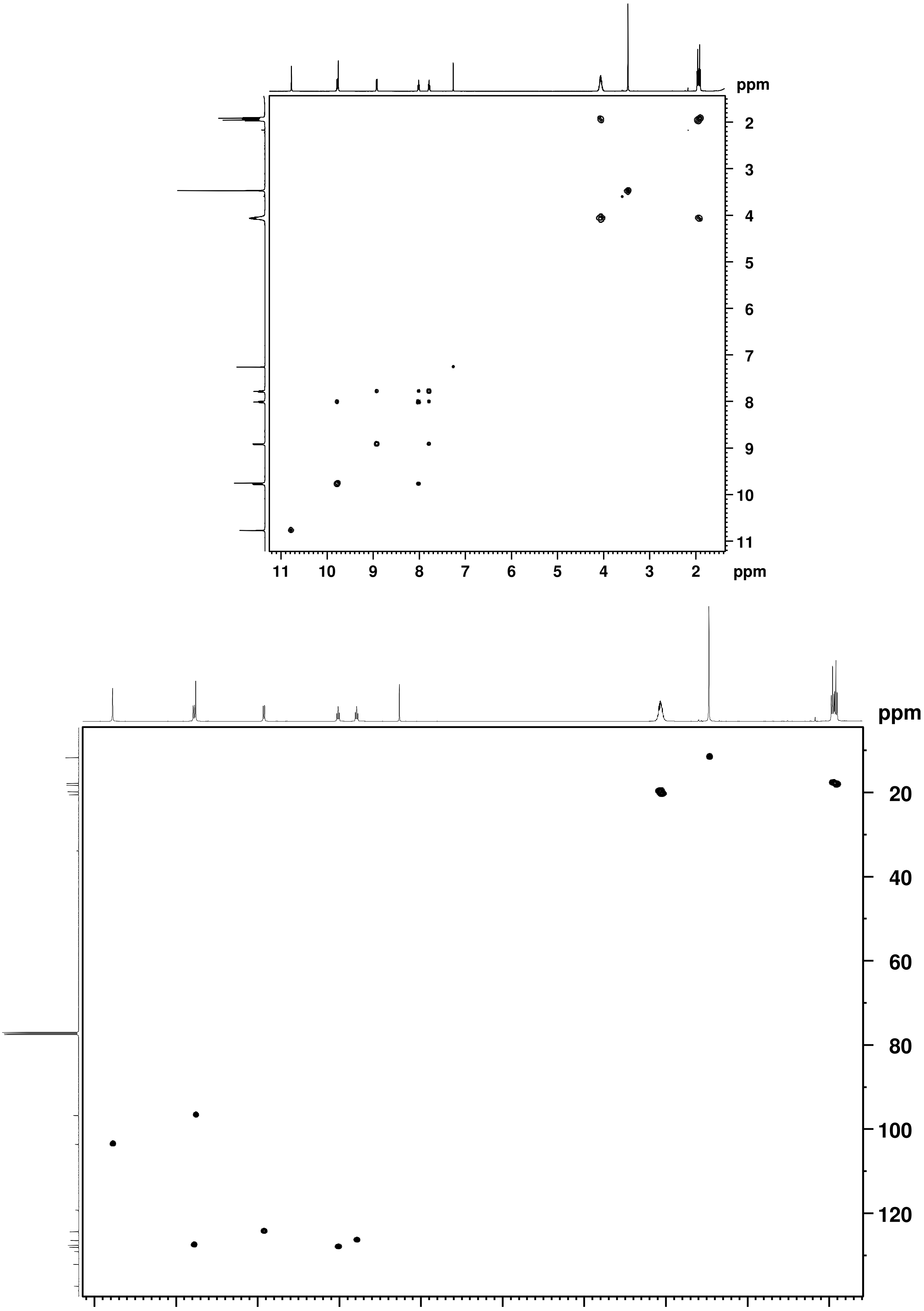

Figure S70. ^{1}H-^{1}H COSY (top) and HSQC (bottom) NMR spectra of **7** in $CDCl_3$ at 50 °C.

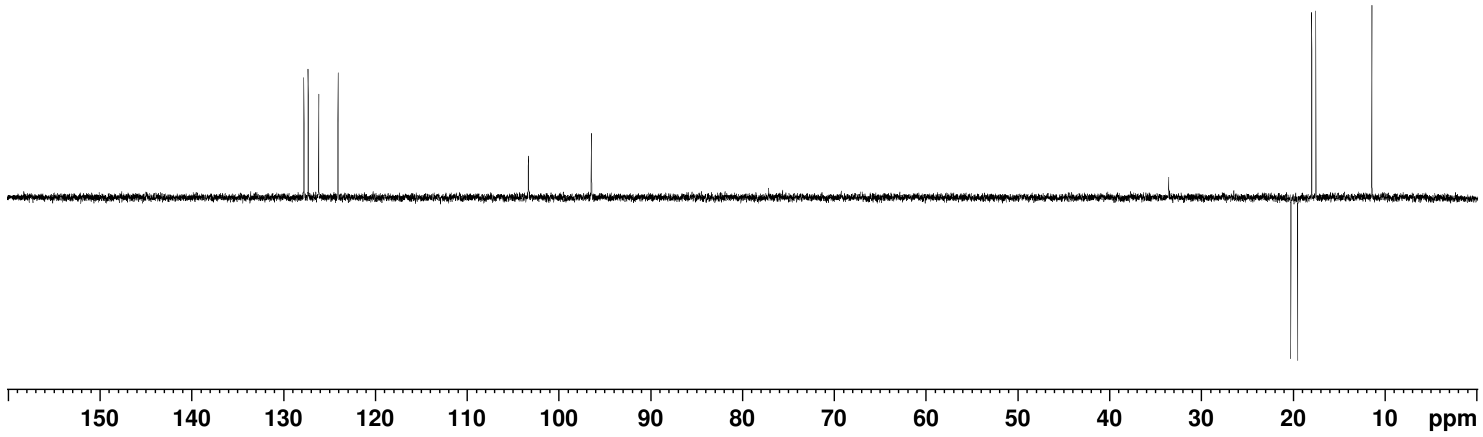

Figure S71. DEPT-135 NMR spectrum of phenanthroporphyrin **7** in $CDCl_3$ at 50 ^oC.

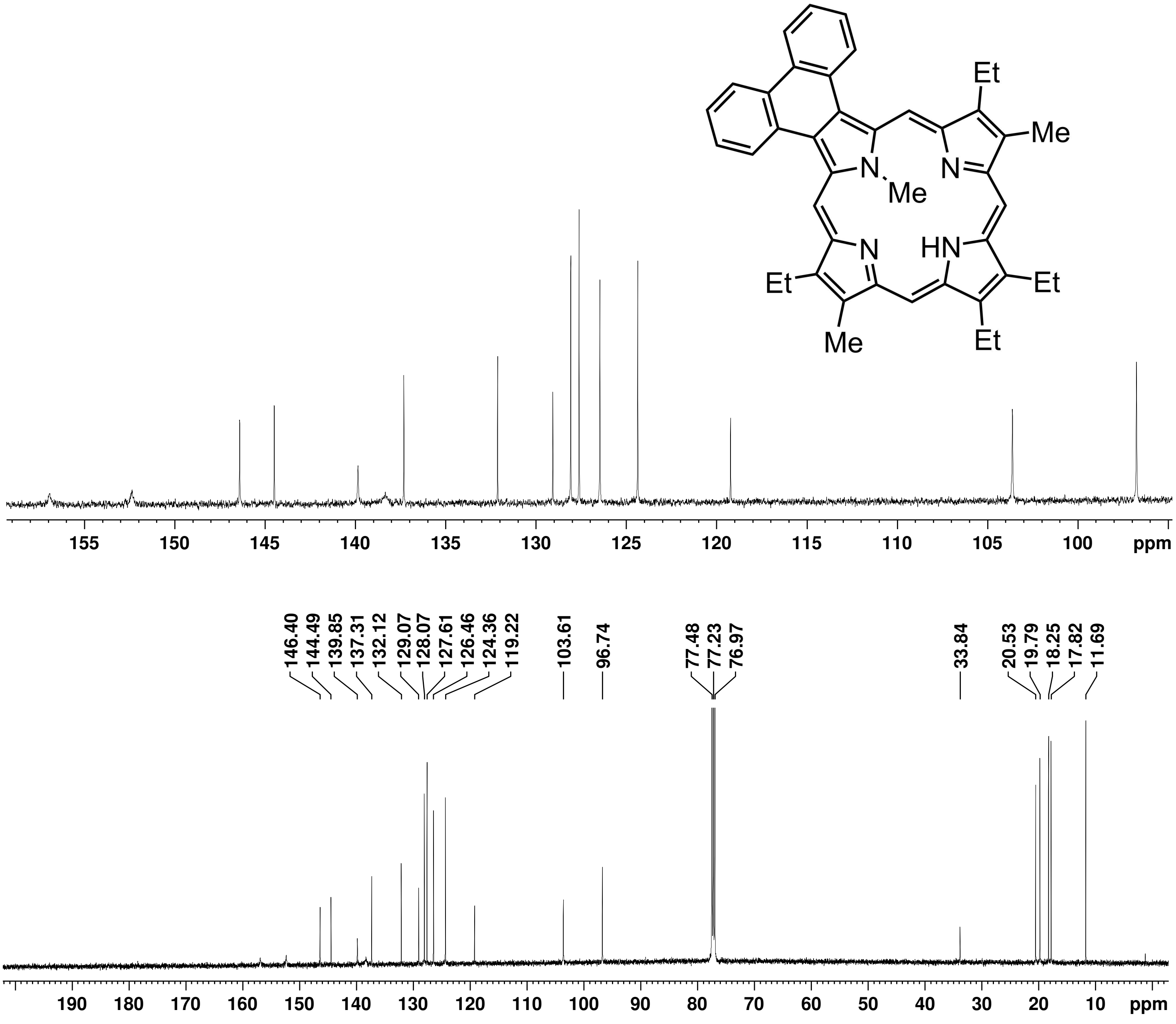

Figure S72. 125 MHz carbon-13 NMR spectrum of phenanthroporphyrin **7** in $CDCl_3$ at 50 ^oC.

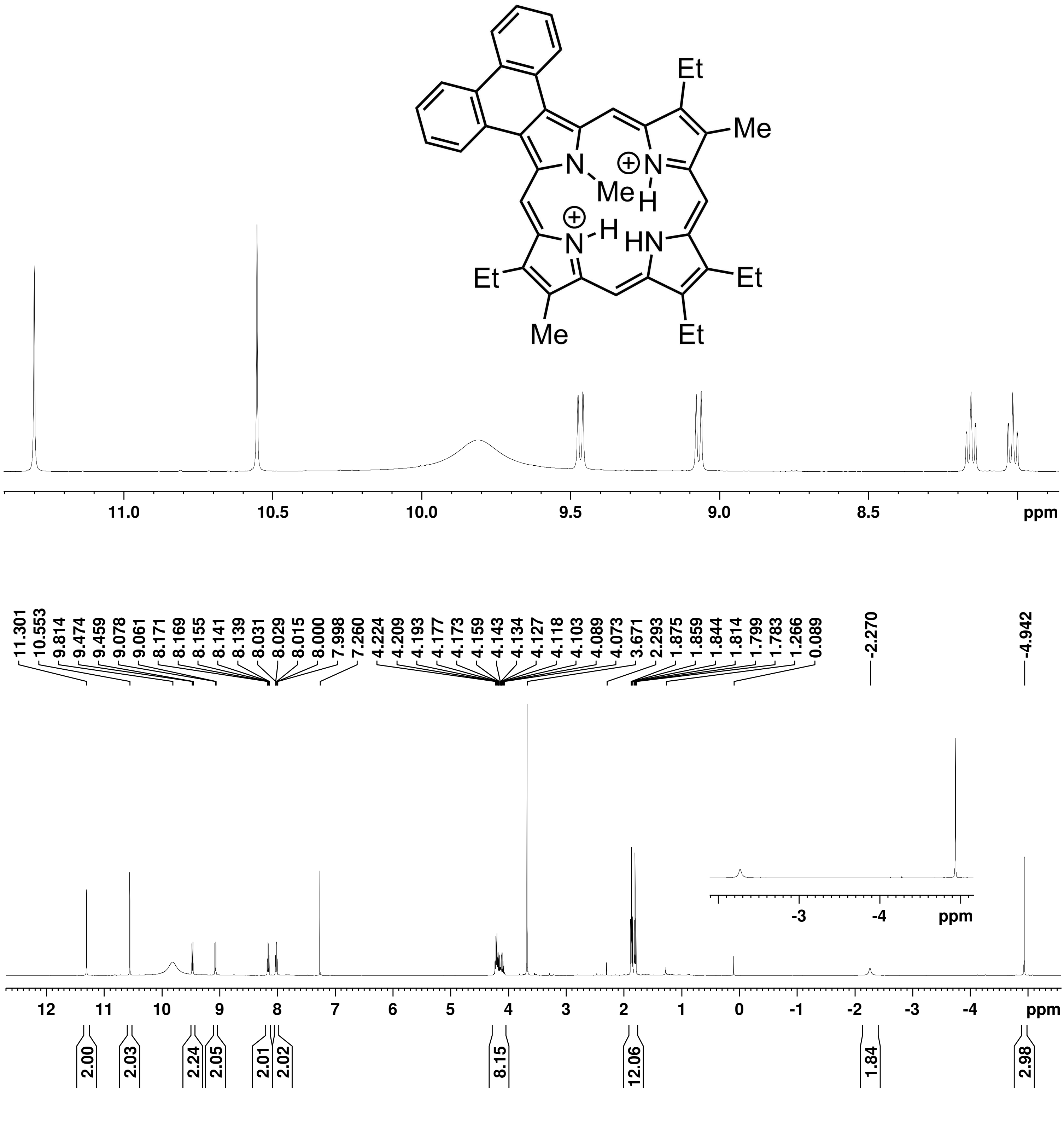

Figure S73. 500 MHz proton NMR spectrum of phenanthroporphyrin dication $\mathbf{7}H_2^{2+}$ in TFA-CDCl$_3$.

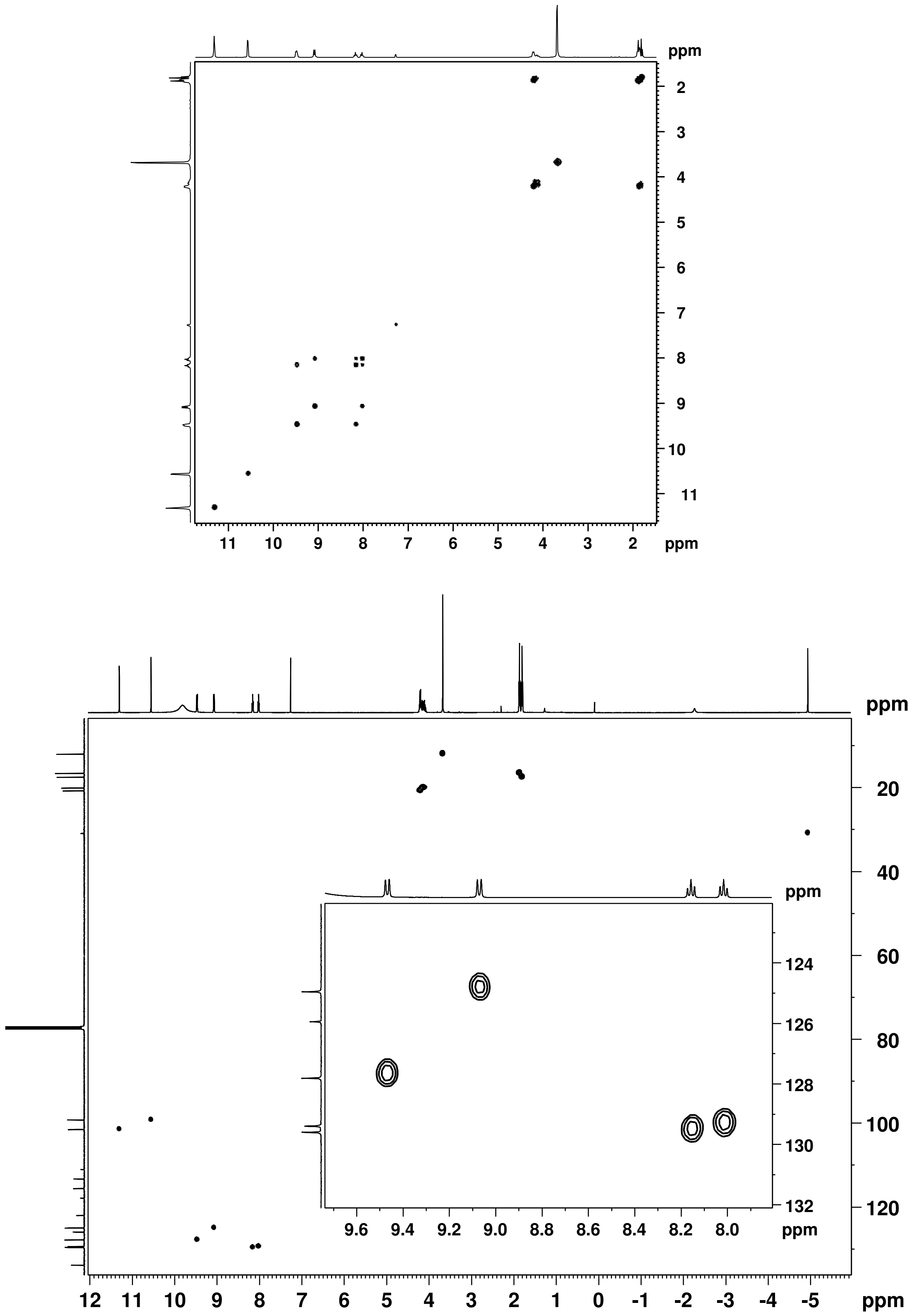

Figure S74. ^{1}H-^{1}H COSY (top) and HSQC (bottom) NMR spectra of dication **7**H_2^{2+} in TFA-$CDCl_3$.

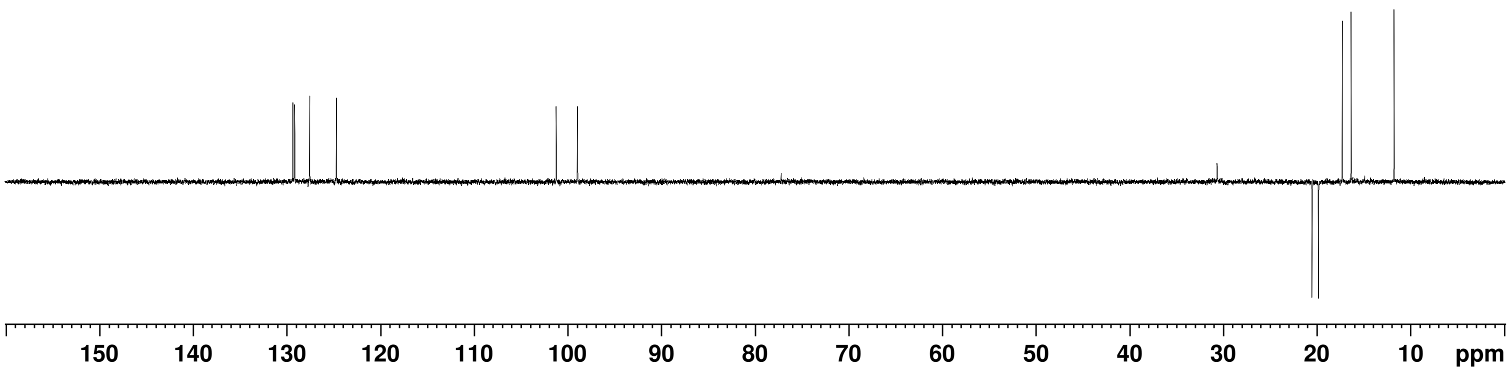

Figure S75. DEPT-135 NMR spectrum of phenanthroporphyrin dication $\mathbf{7}H_2^{2+}$ in TFA-CDCl$_3$.

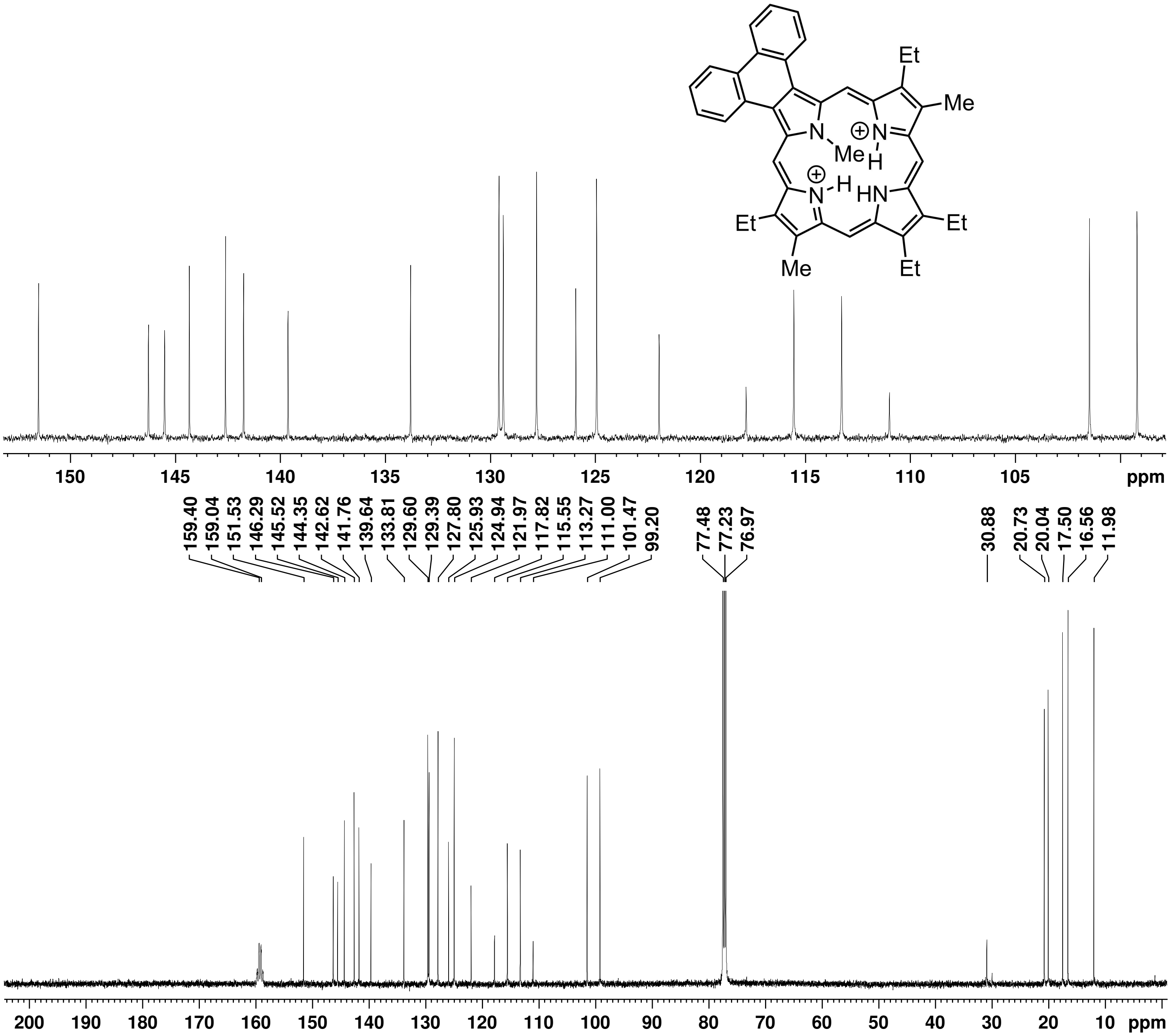

Figure S76. DEPT-135 NMR spectrum of phenanthroporphyrin dication $\mathbf{7}H_2^{2+}$ in TFA-CDCl$_3$.

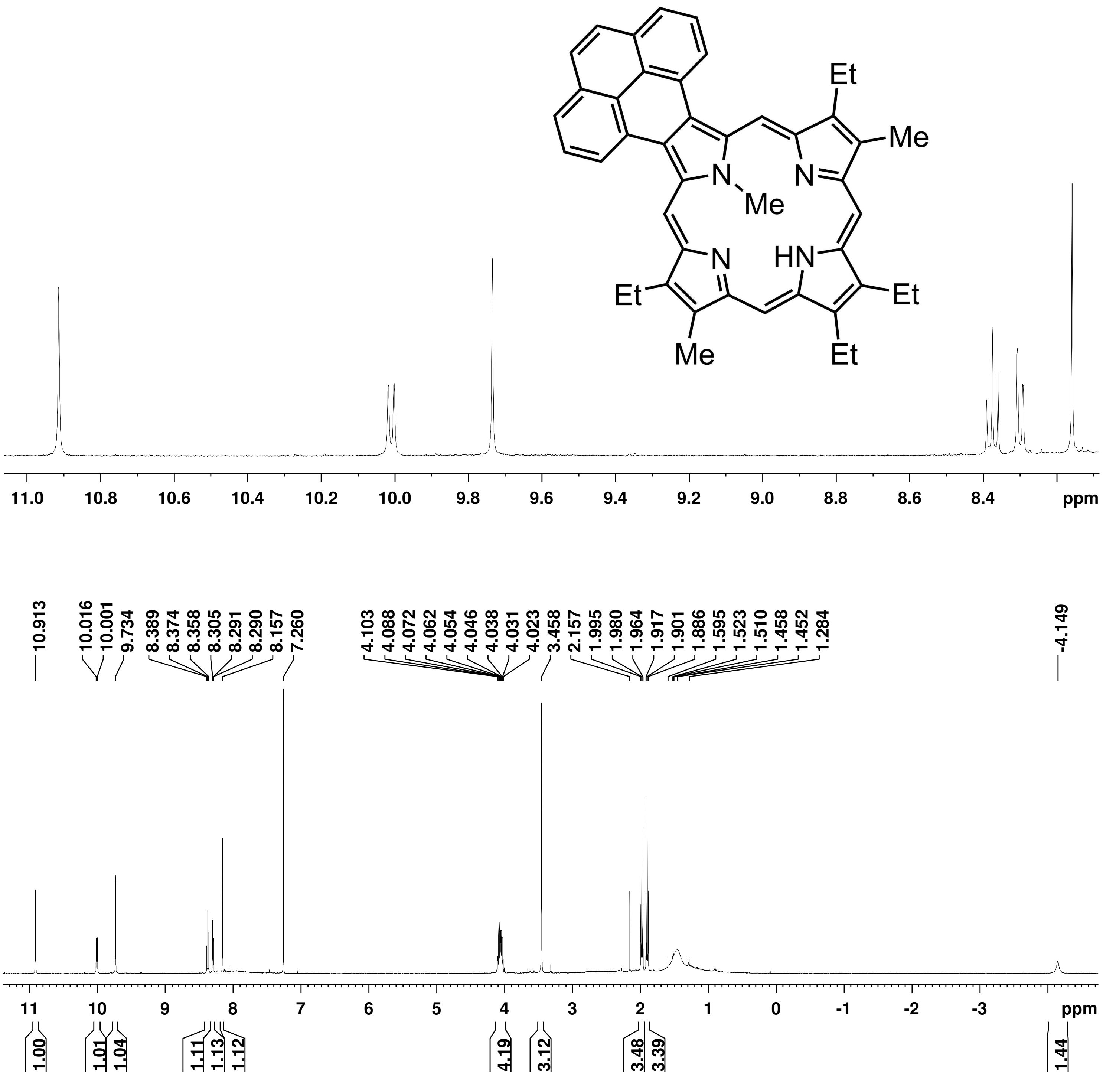

Figure S77. 500 MHz proton NMR spectrum of *N*-methylpyrenoporphyrin **8** in $CDCl_3$ at 55 °C.

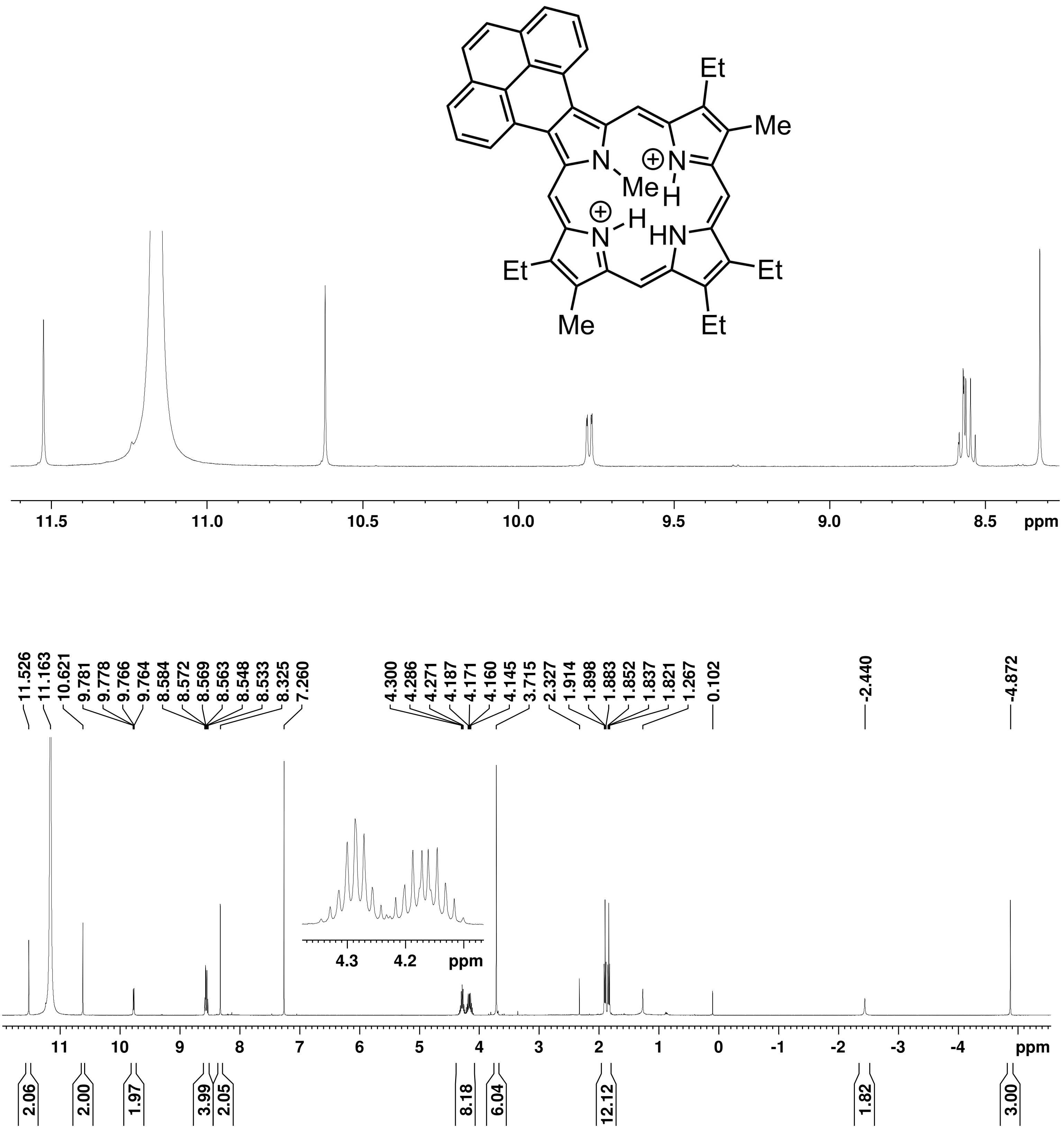

Figure S78. 500 MHz proton NMR spectrum of *N*-methylpyrenoporphyrin dication **8**H_2^{2+} in TFA-$CDCl_3$.

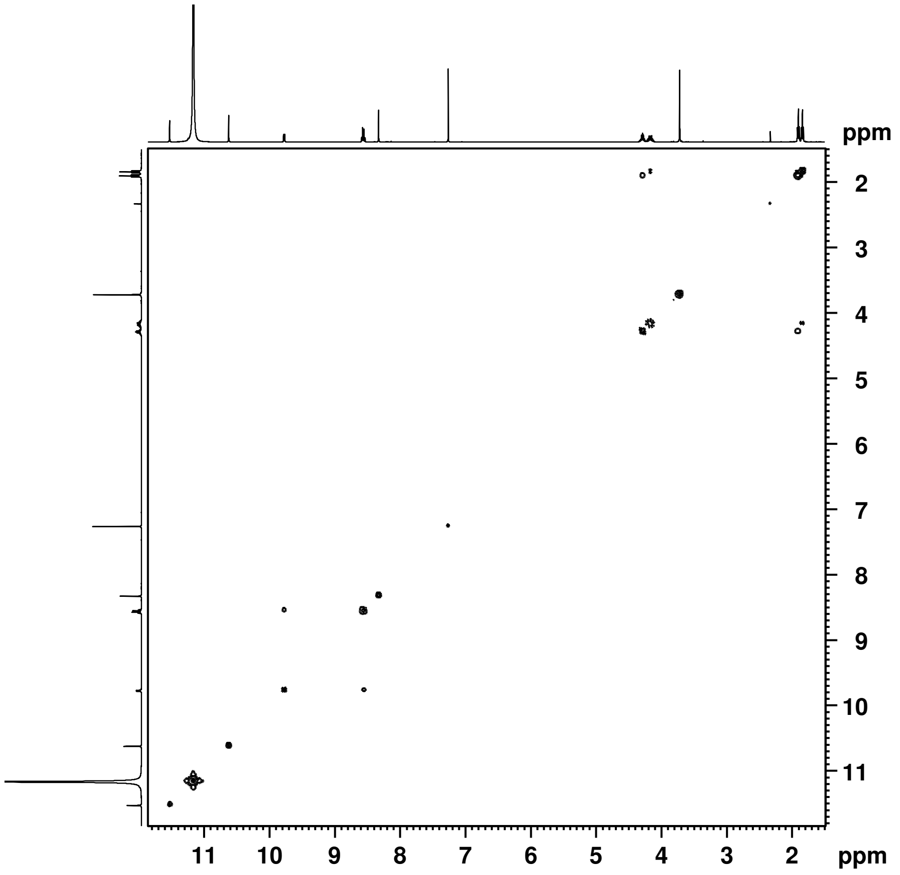

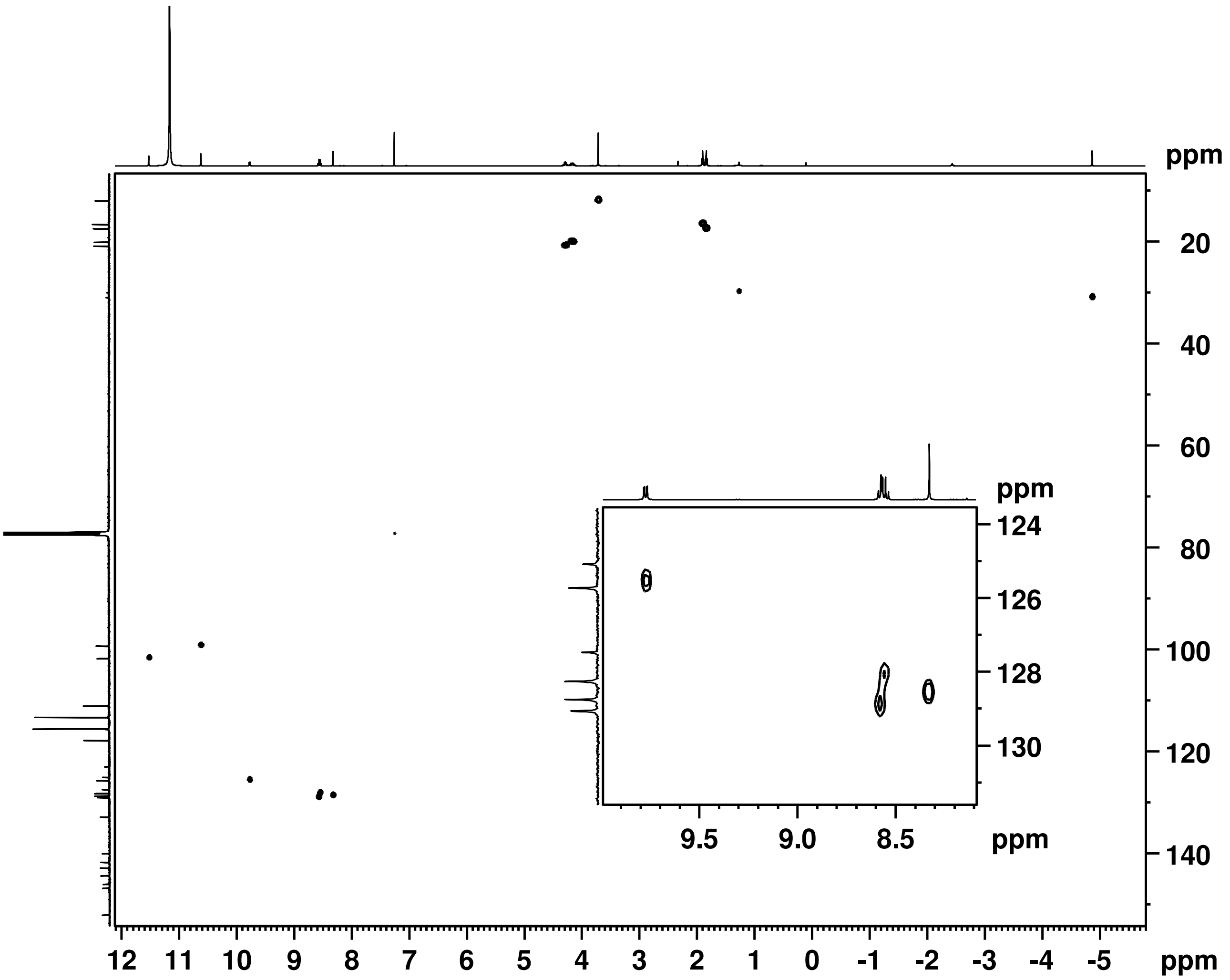

Figure S79. ^1H-^1H COSY (top) and HSQC NMR spectra of dication **8**H_2^{2+} in TFA-$CDCl_3$.

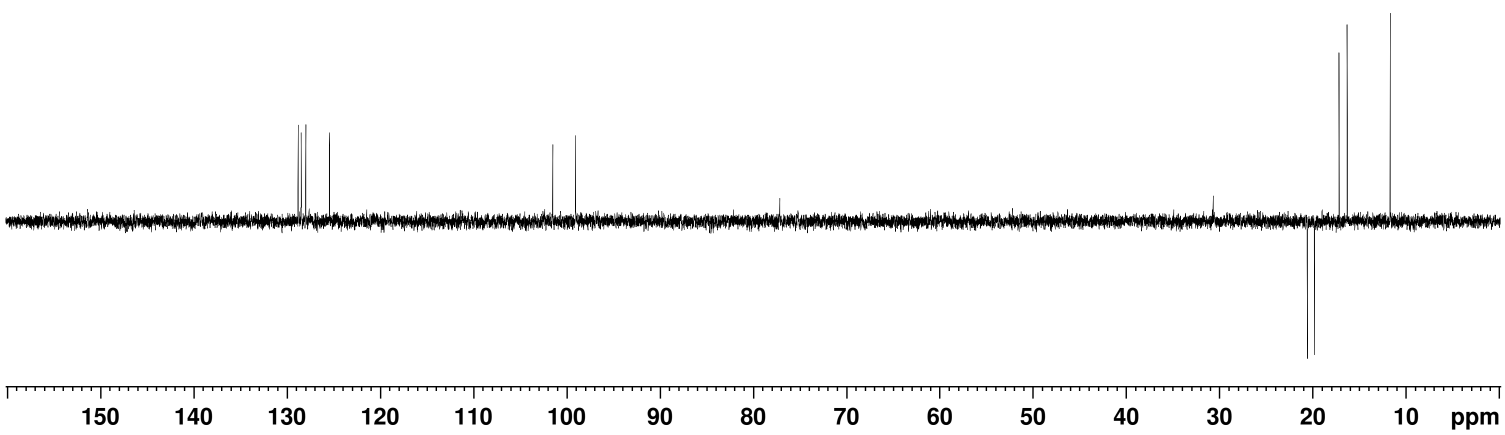

Figure S80. DEPT-135 NMR spectrum of pyrenoporphyrin dication **8**H_2^{2+} in TFA-CDCl$_3$.

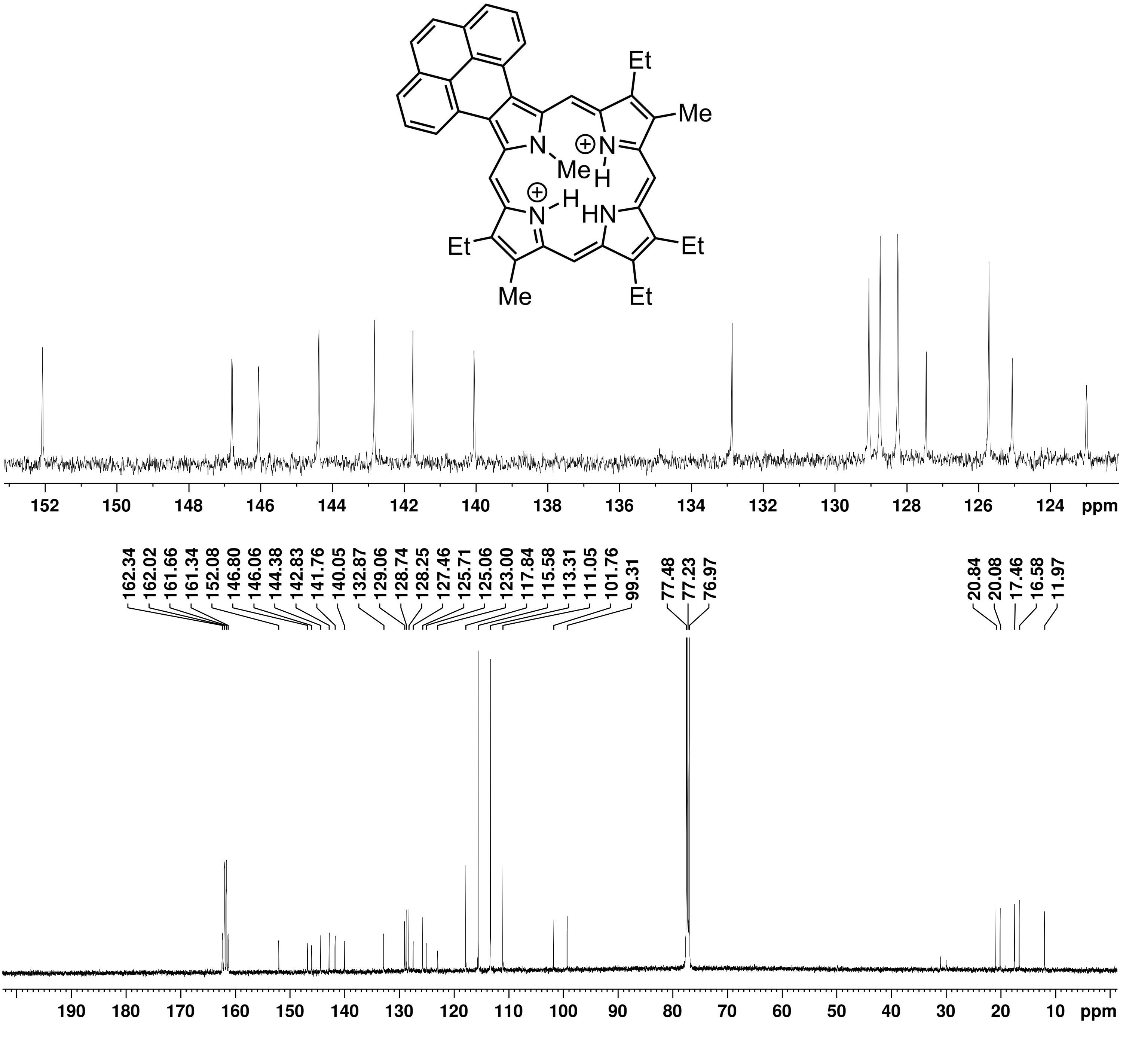

Figure S81. 125 MHz carbon-13 NMR spectrum of pyrenoporphyrin dication **8**H_2^{2+} in TFA-CDCl$_3$.

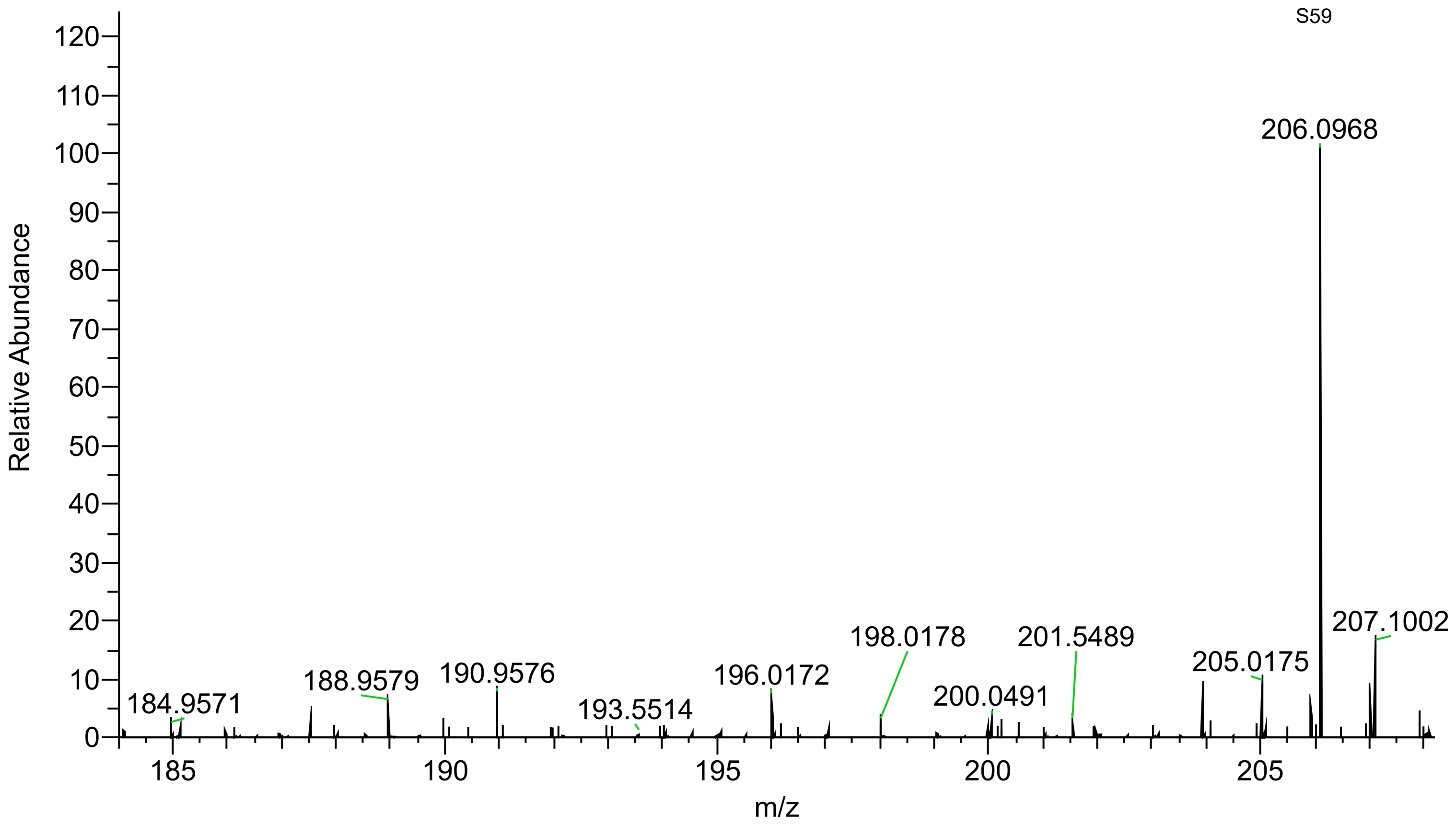

Figure S82. High resolution TOF ESI MS of *N*-methylacenaphthopyrrole **11a**.

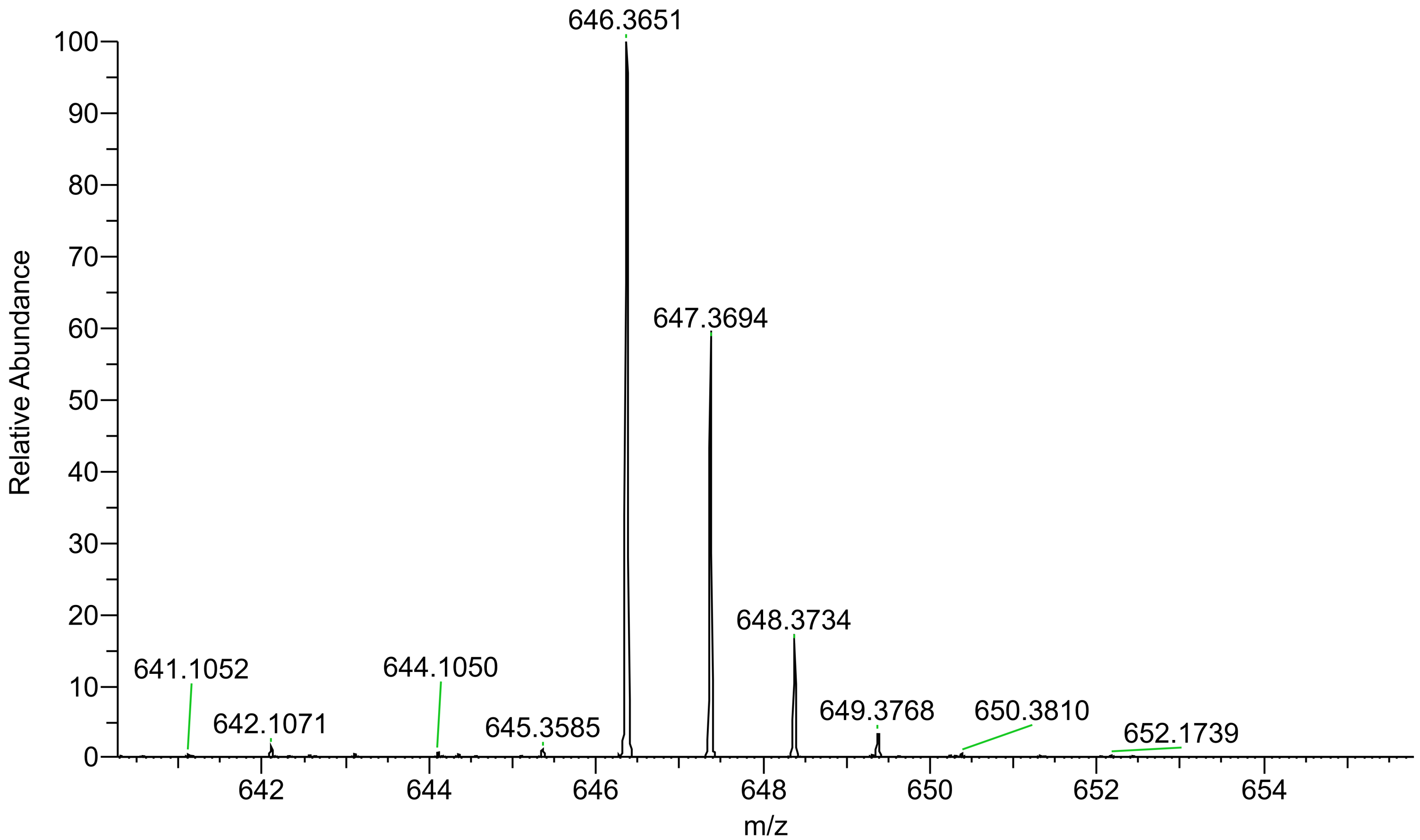

Figure S83. High resolution TOF ESI MS of acenaphthotripyrrane **13**.

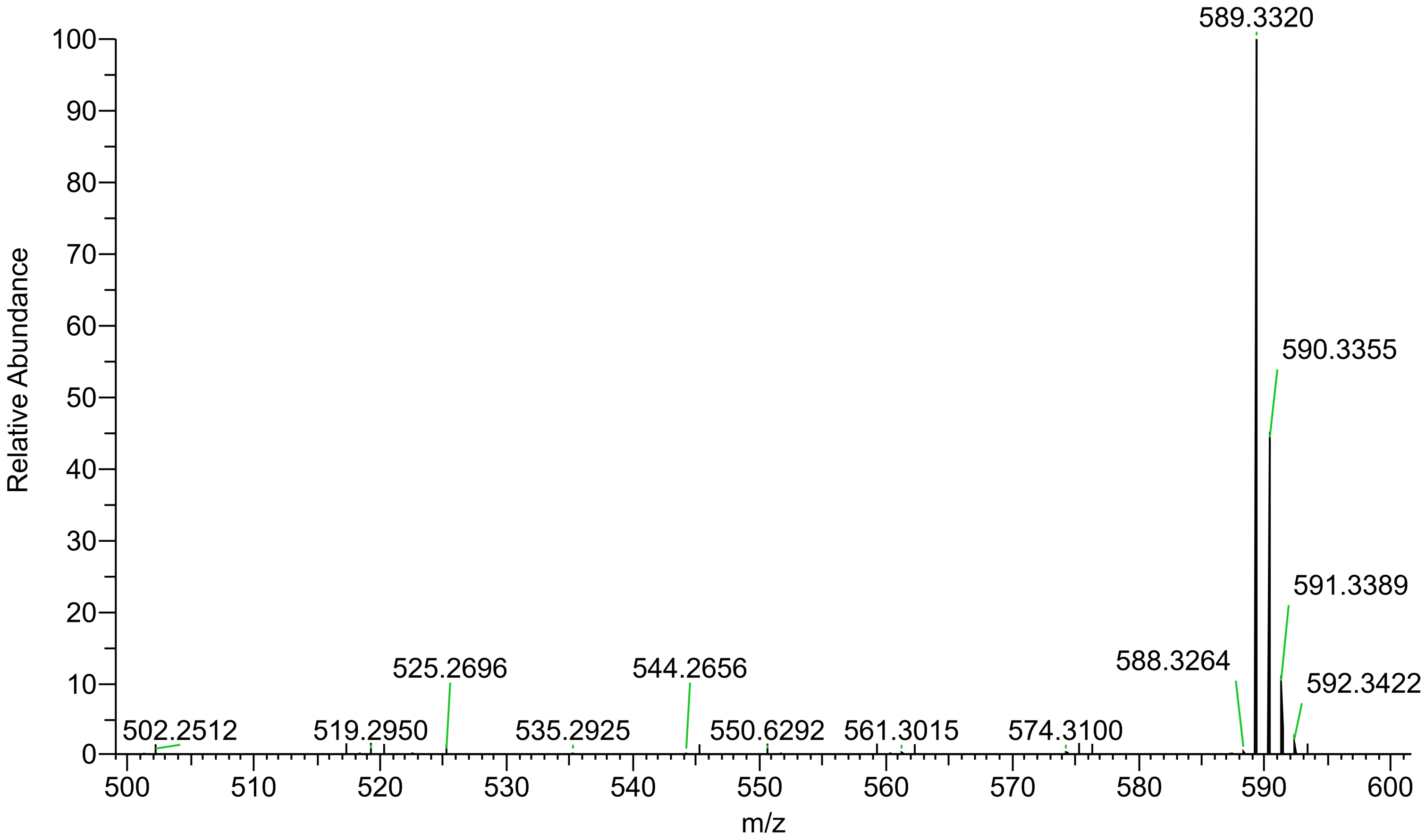

Figure S84. High resolution TOF ESI MS of *N*-methylacenaphthoporphyrin **6**.

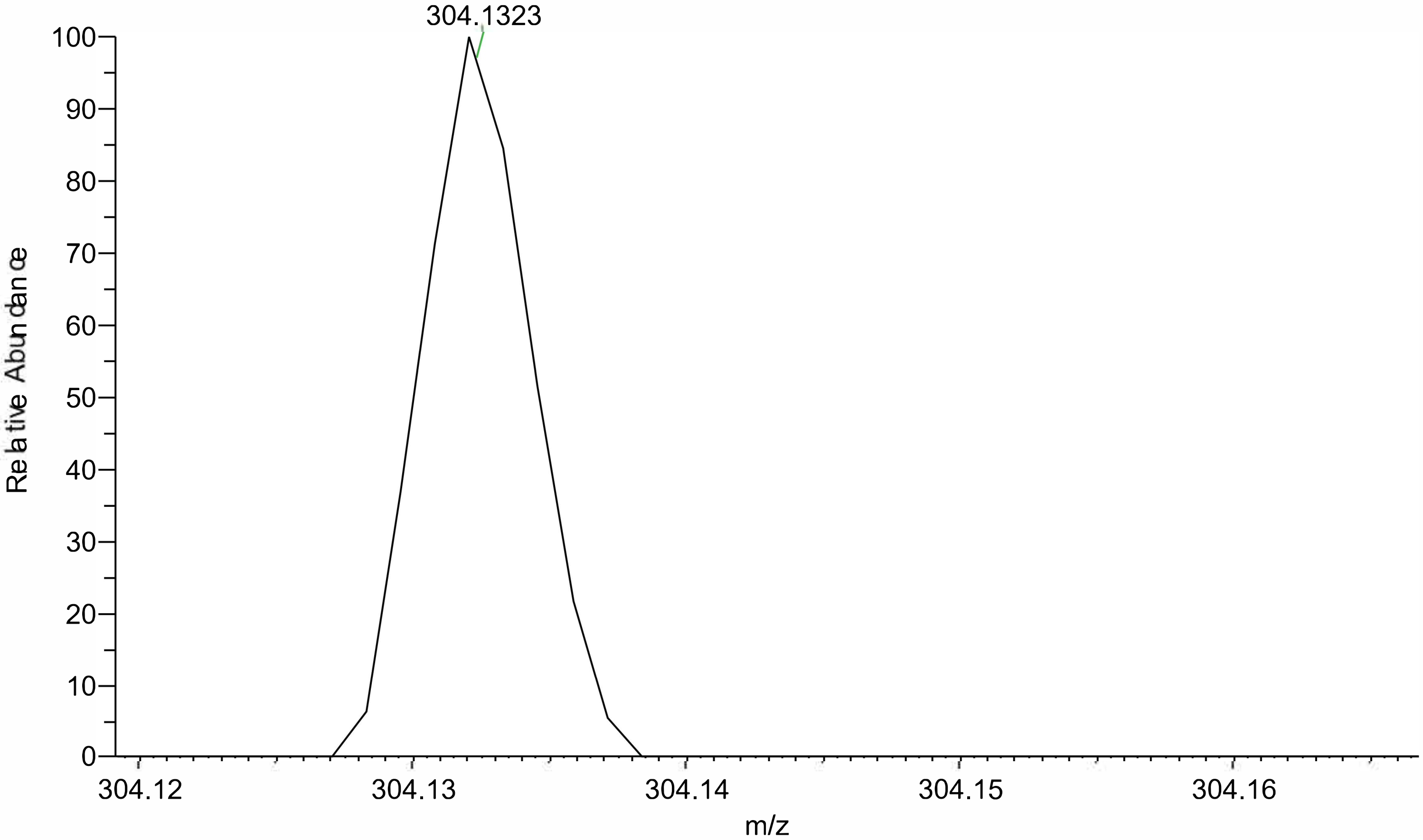

Figure S85. High resolution TOF ESI MS of *N*-methylphenanthropyrrole ethyl ester **16**.

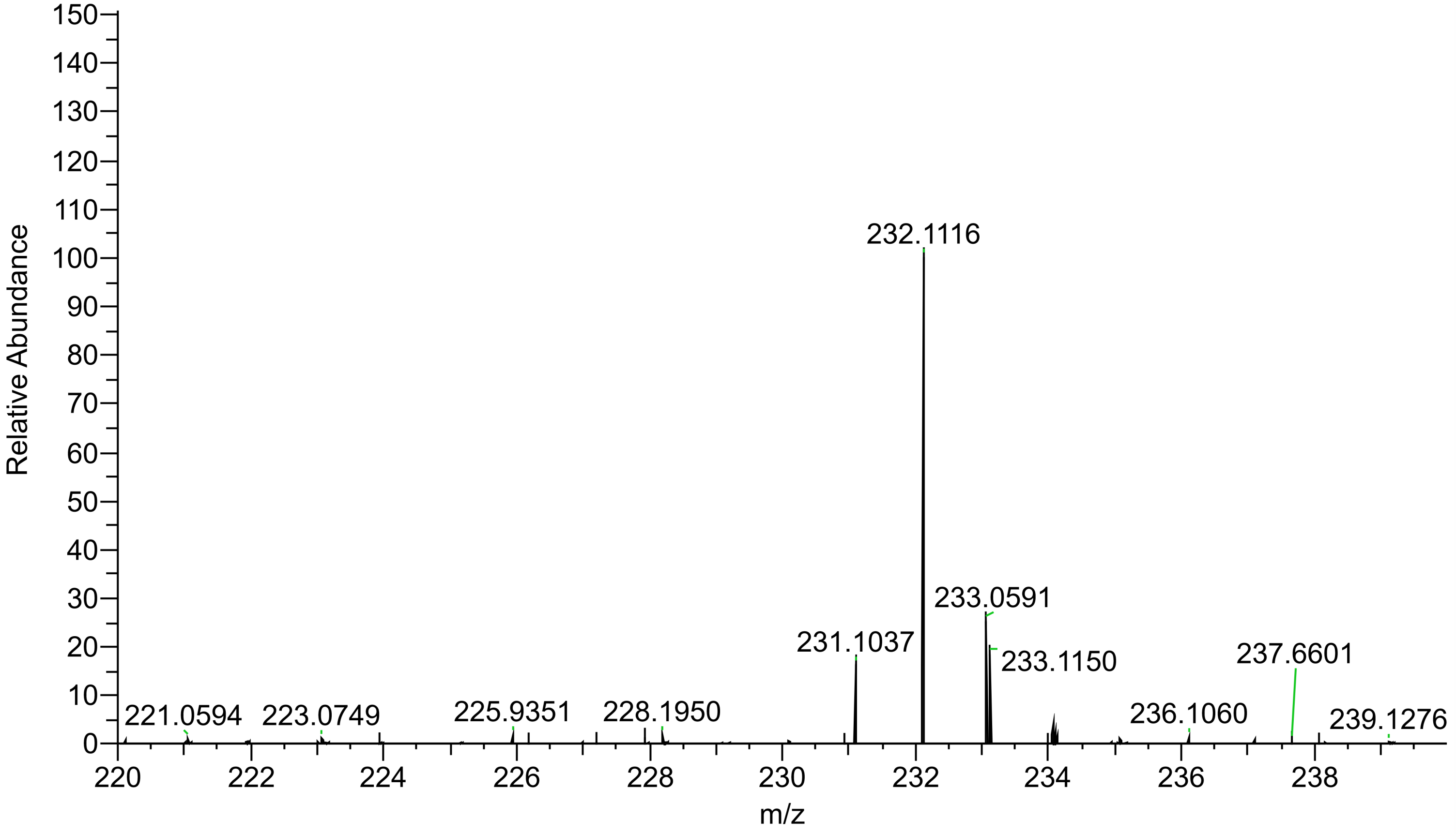

Figure S86. High resolution TOF ESI MS of *N*-methylphenanthropyrrole **17a**.

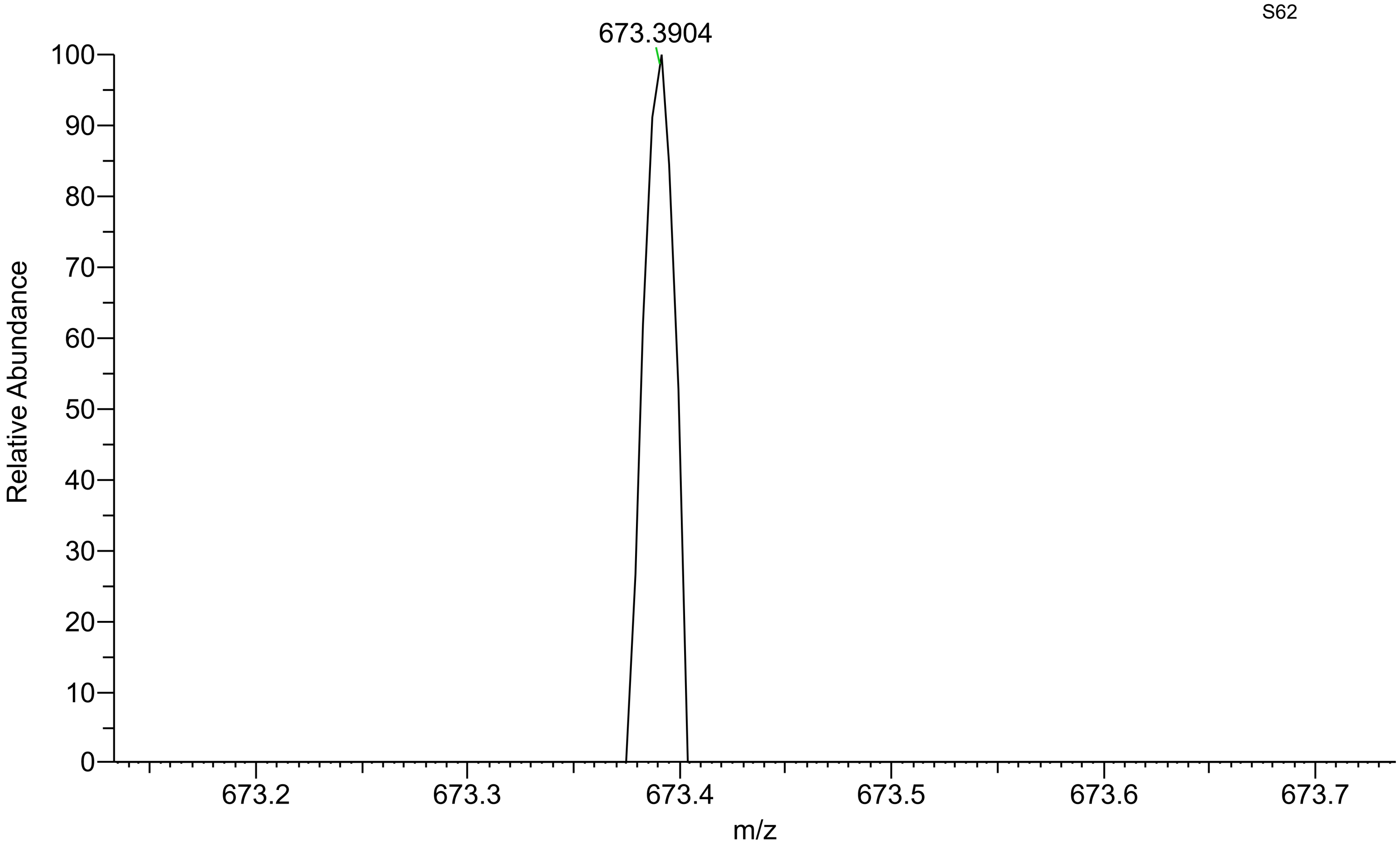

Figure S87. High resolution TOF ESI MS of phenanthrotripyrrane **21**.

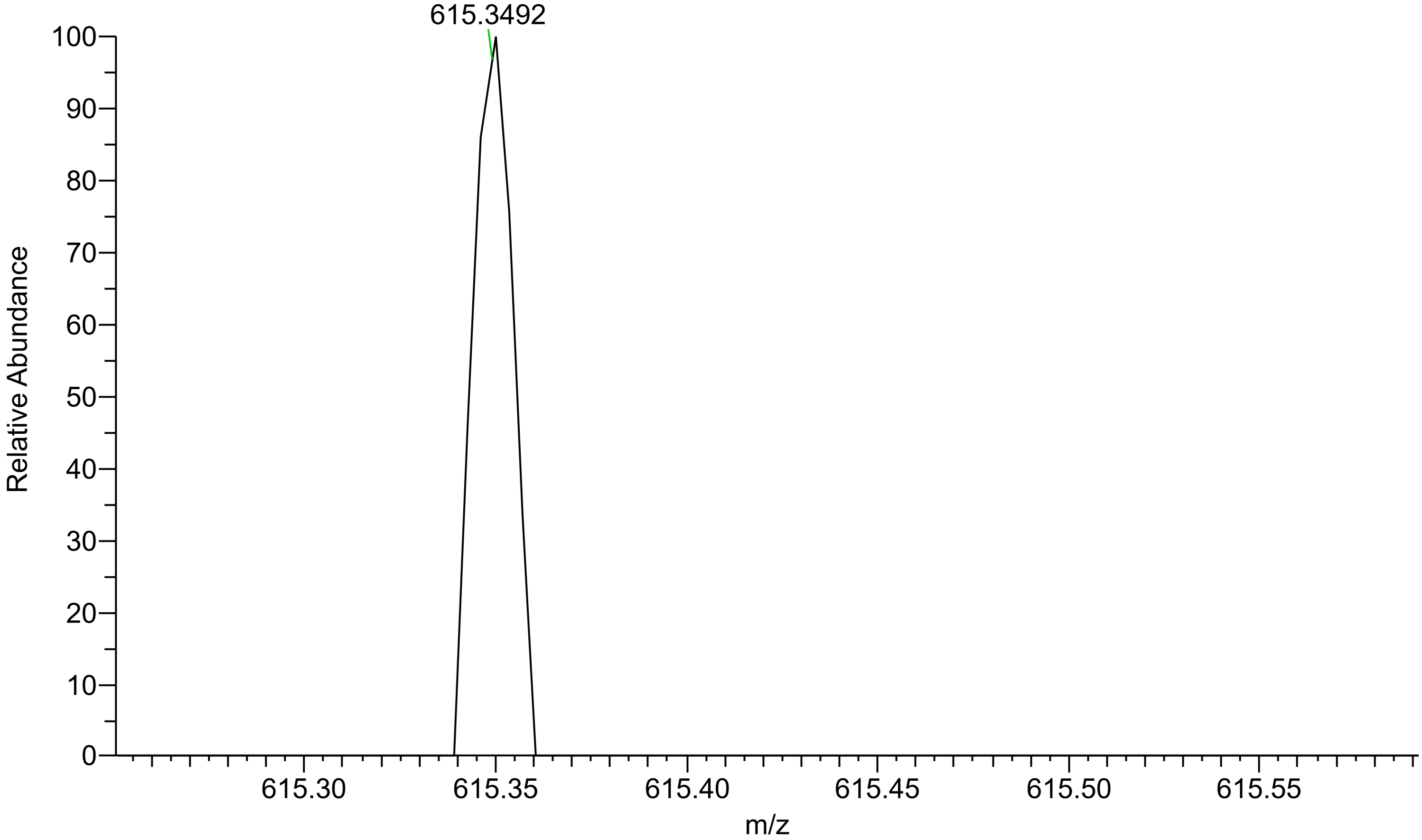

Figure S88. High resolution TOF ESI MS of *N*-methylphenanthroporphyrin **7**.

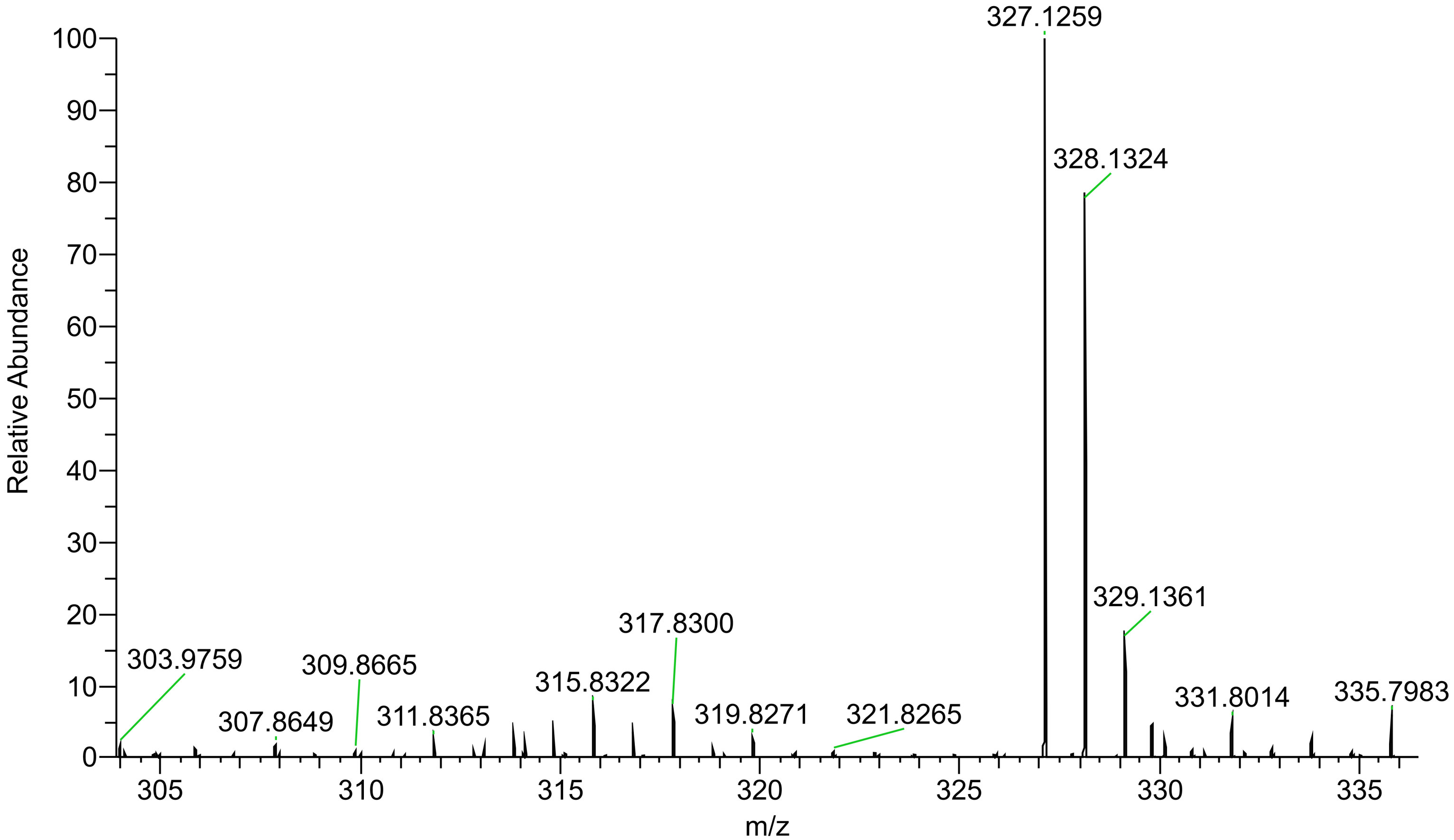

Figure S89. High resolution TOF ESI MS of *N*-methylpyrenopyrrole ethyl ester **19**.

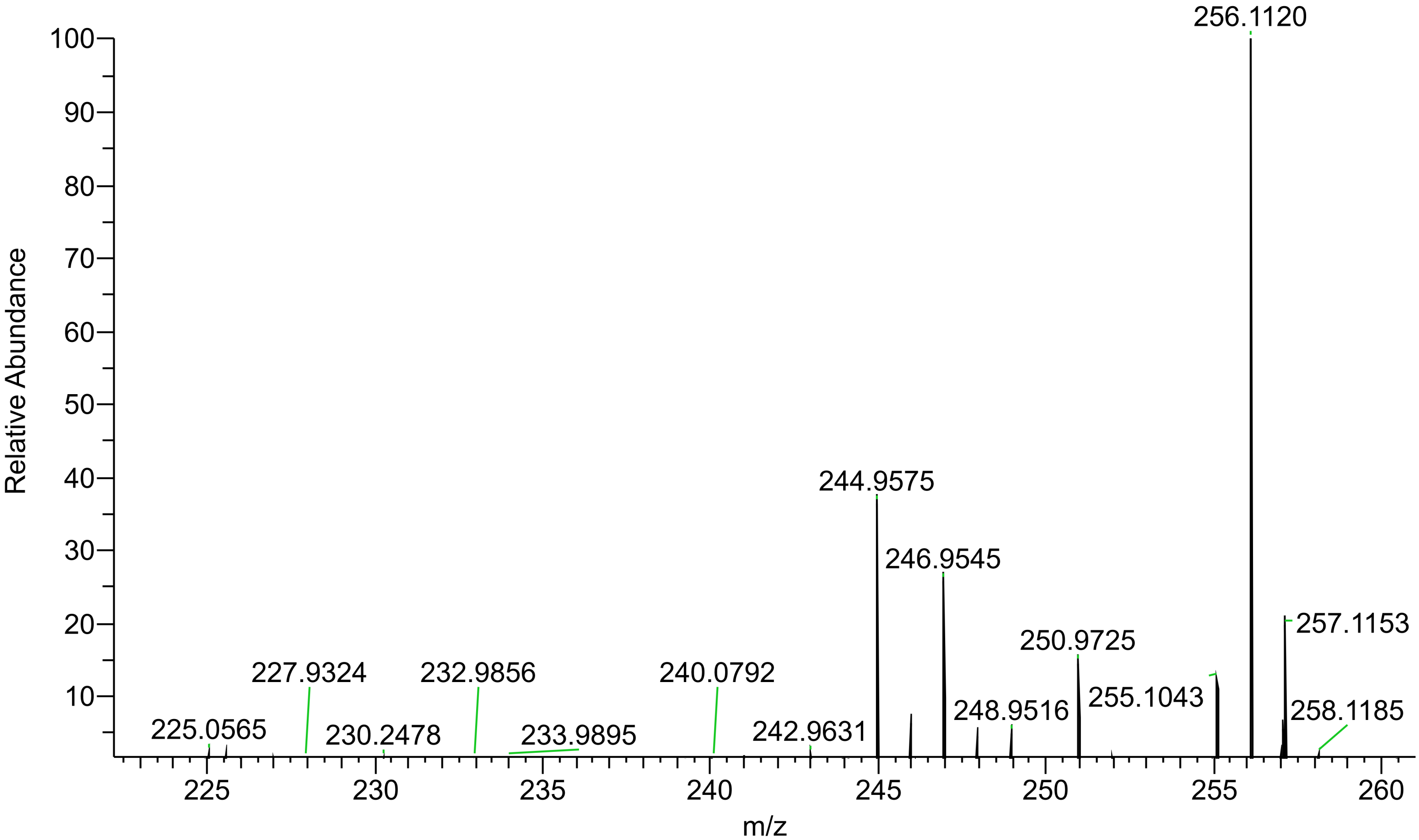

Figure S90. High resolution TOF ESI MS of *N*-methylpyrenopyrrole **20a**.

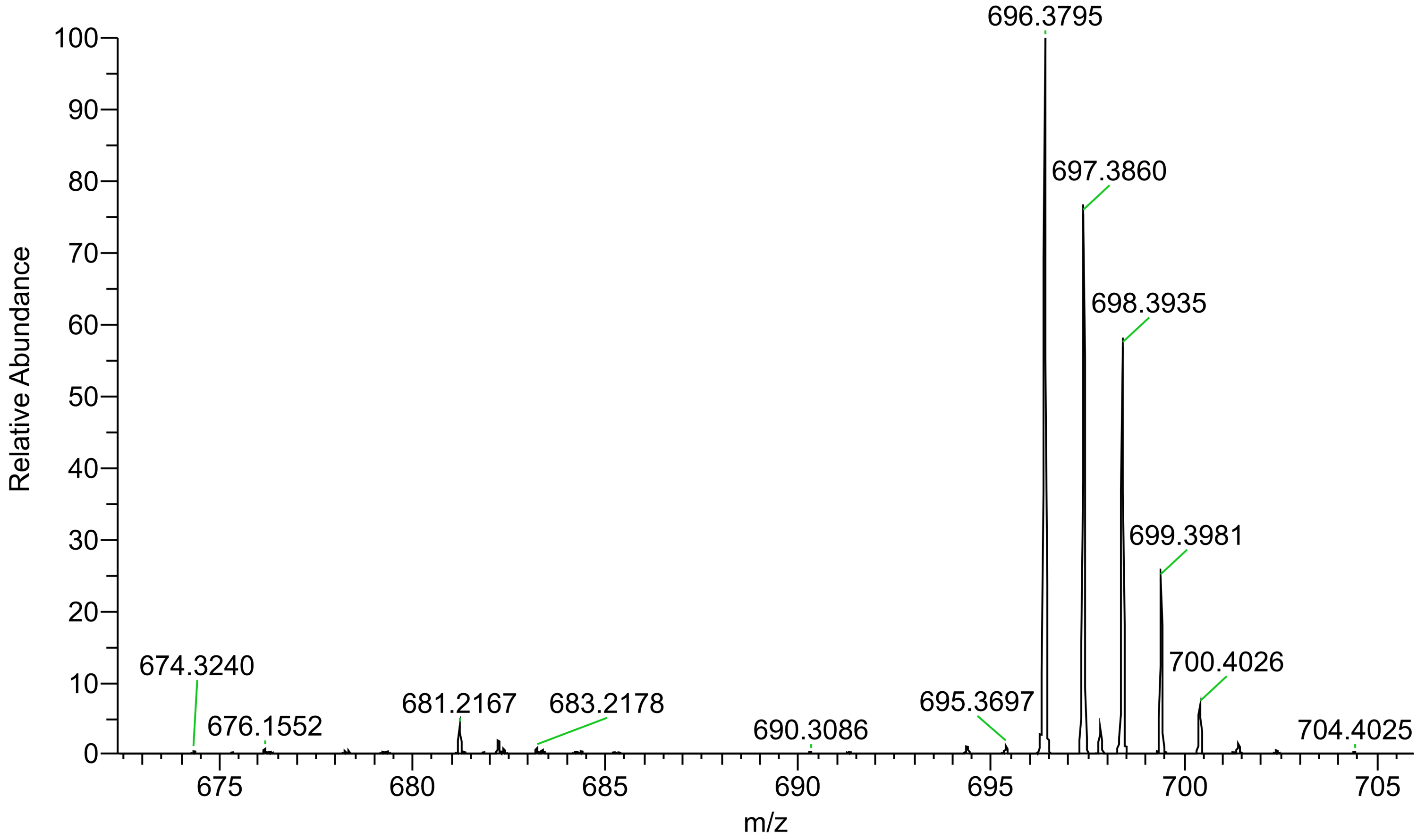

Figure S91. High resolution TOF ESI MS of pyrenotripyrrane **22**.

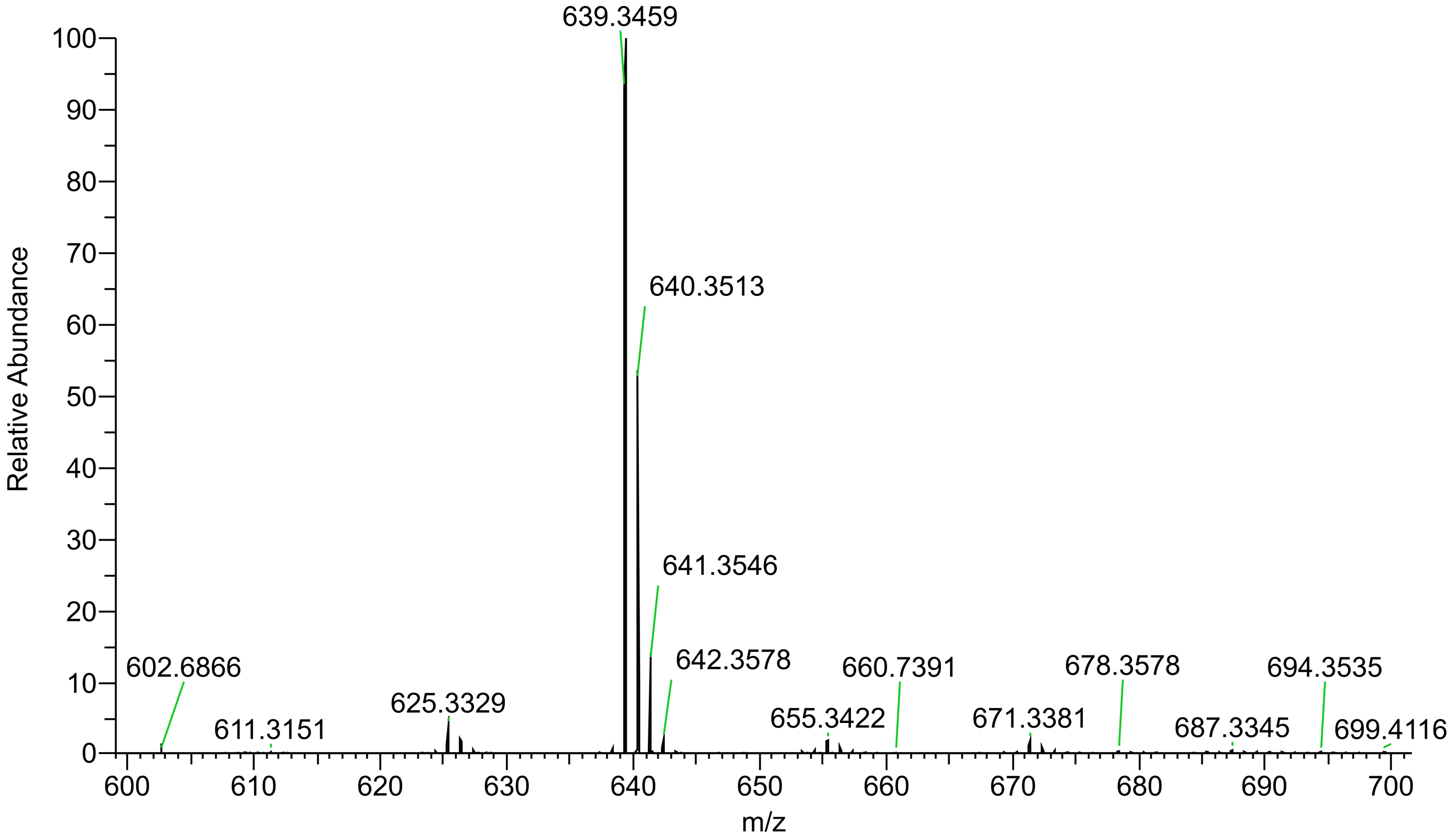

Figure S92. High resolution TOF ESI MS of *N*-methylpyrenoporphyrin **8**.

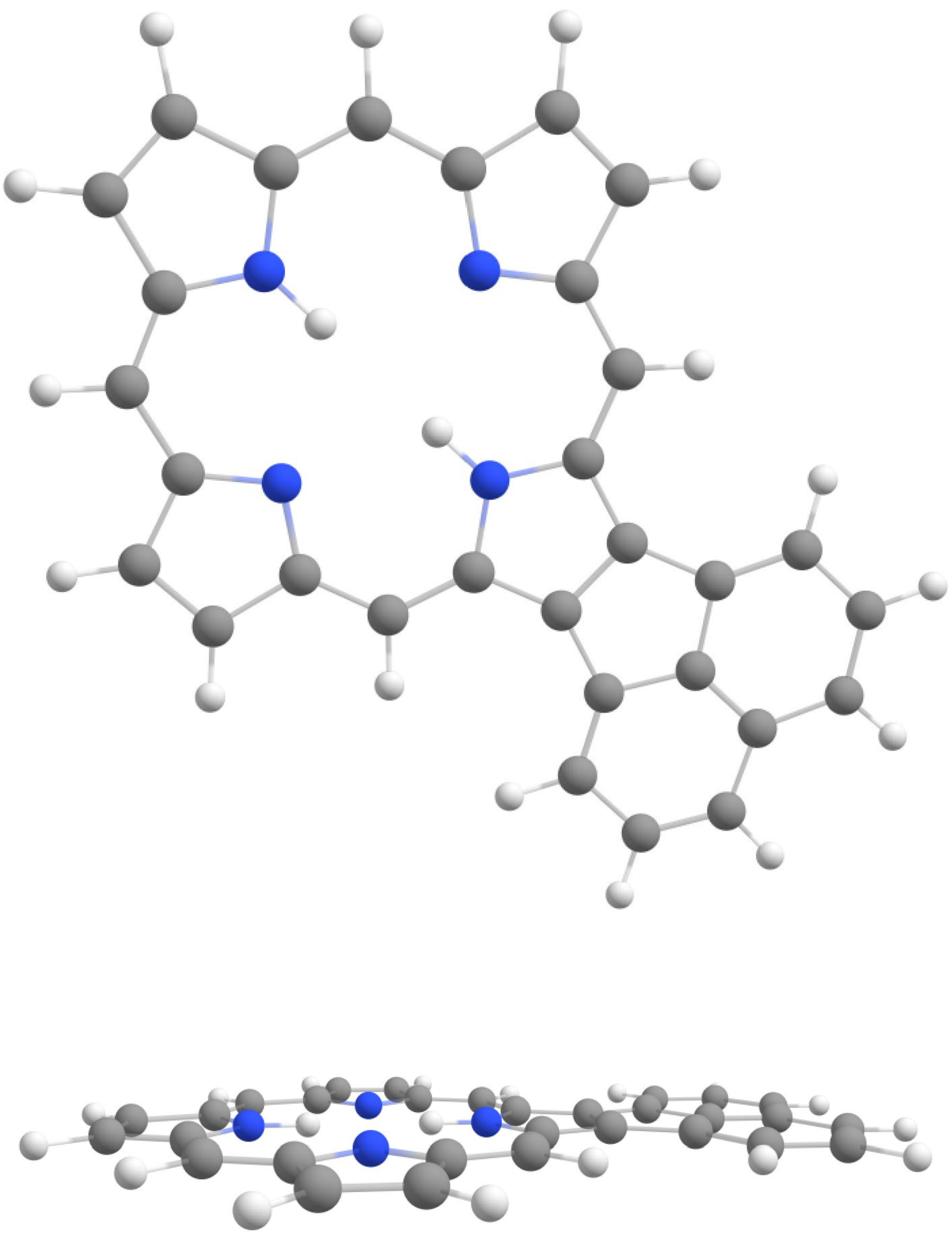

Figure S93. DFT calculated conformation (2 views) for acenaphthoporphyrin tautomer **ANPa**.

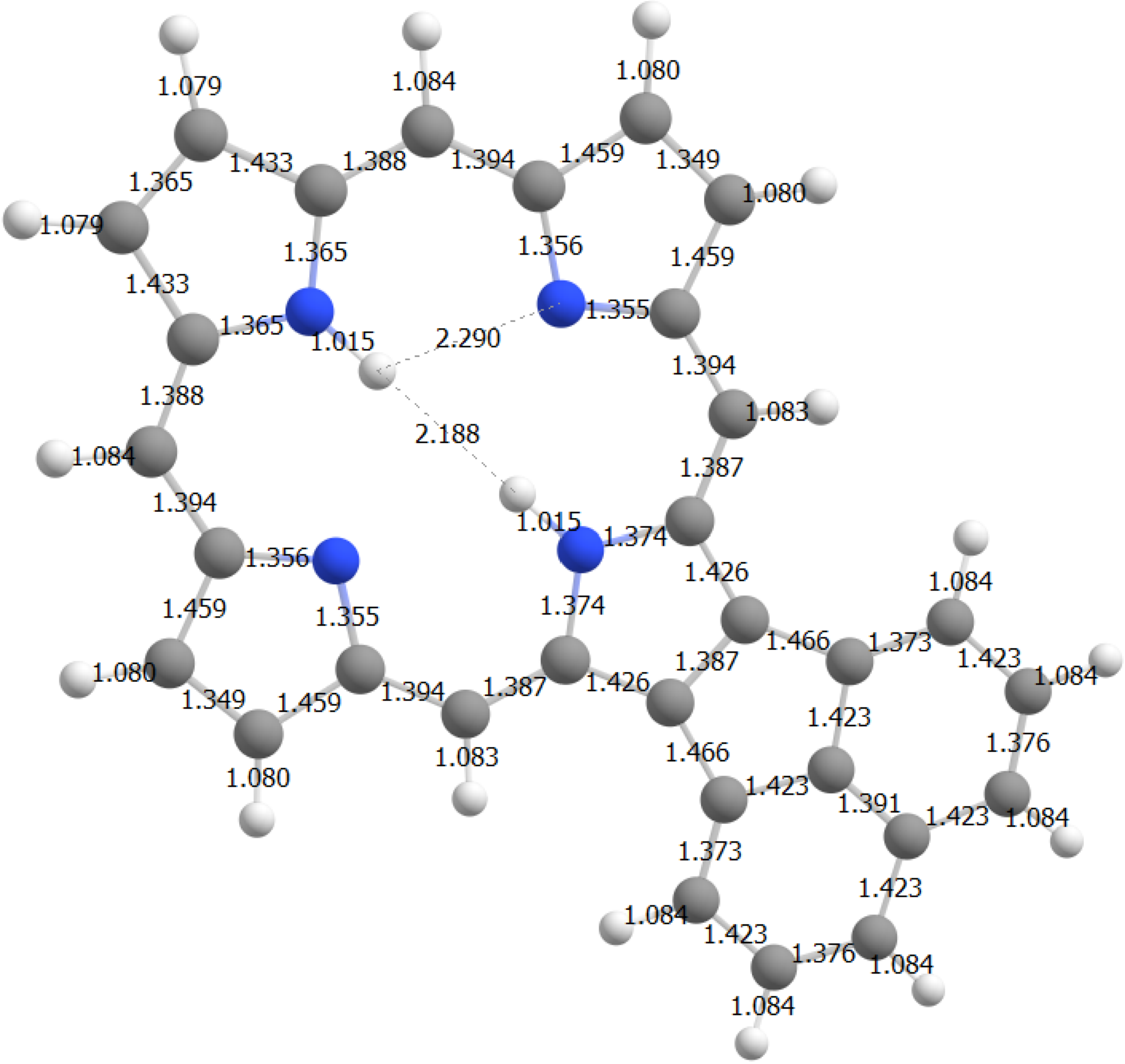

Figure S94. DFT calculated bond lengths for acenaphthoporphyrin tautomer **ANPa**.

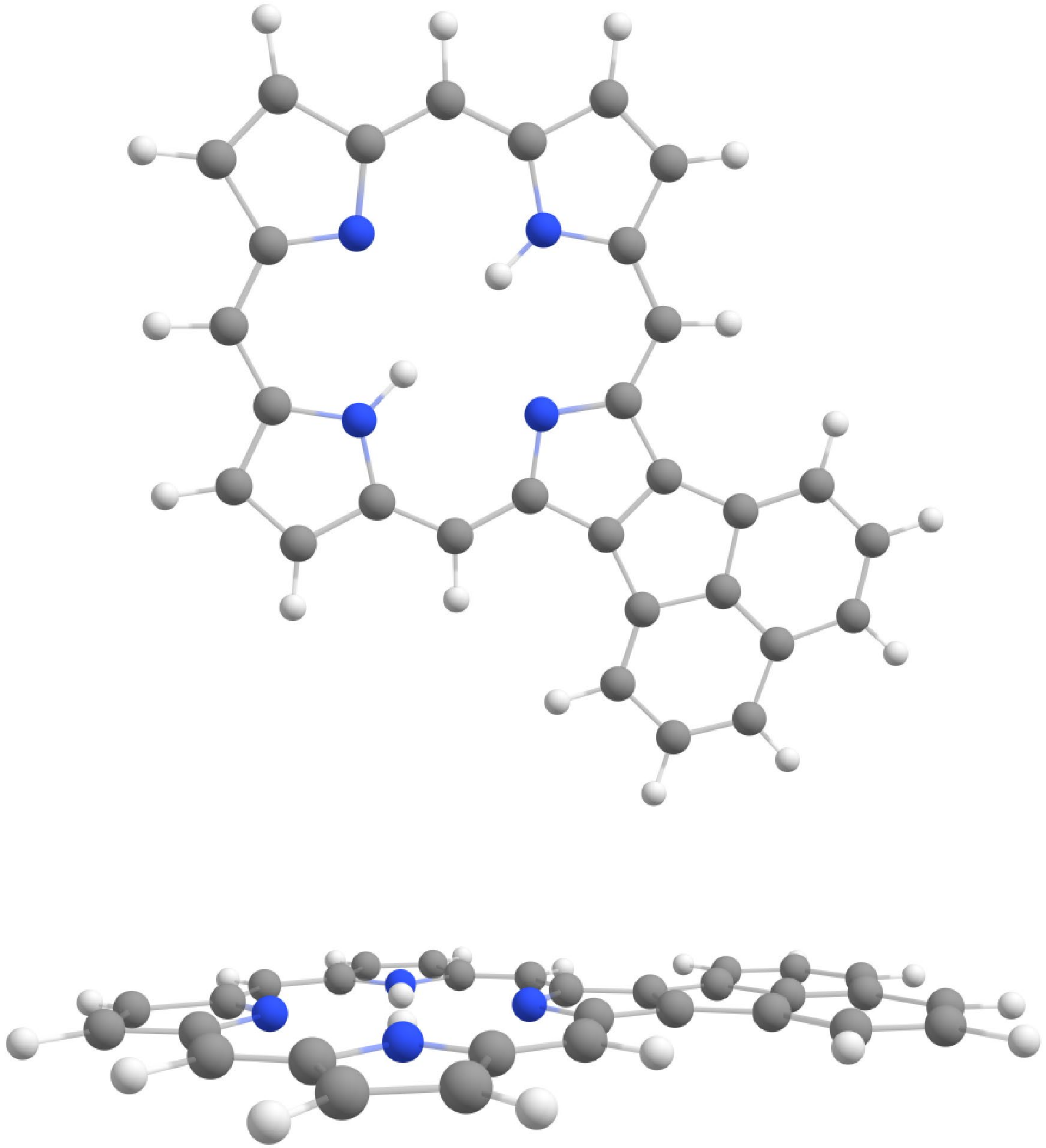

Figure S95. DFT calculated conformation (2 views) for acenaphthoporphyrin tautomer **ANPb**.

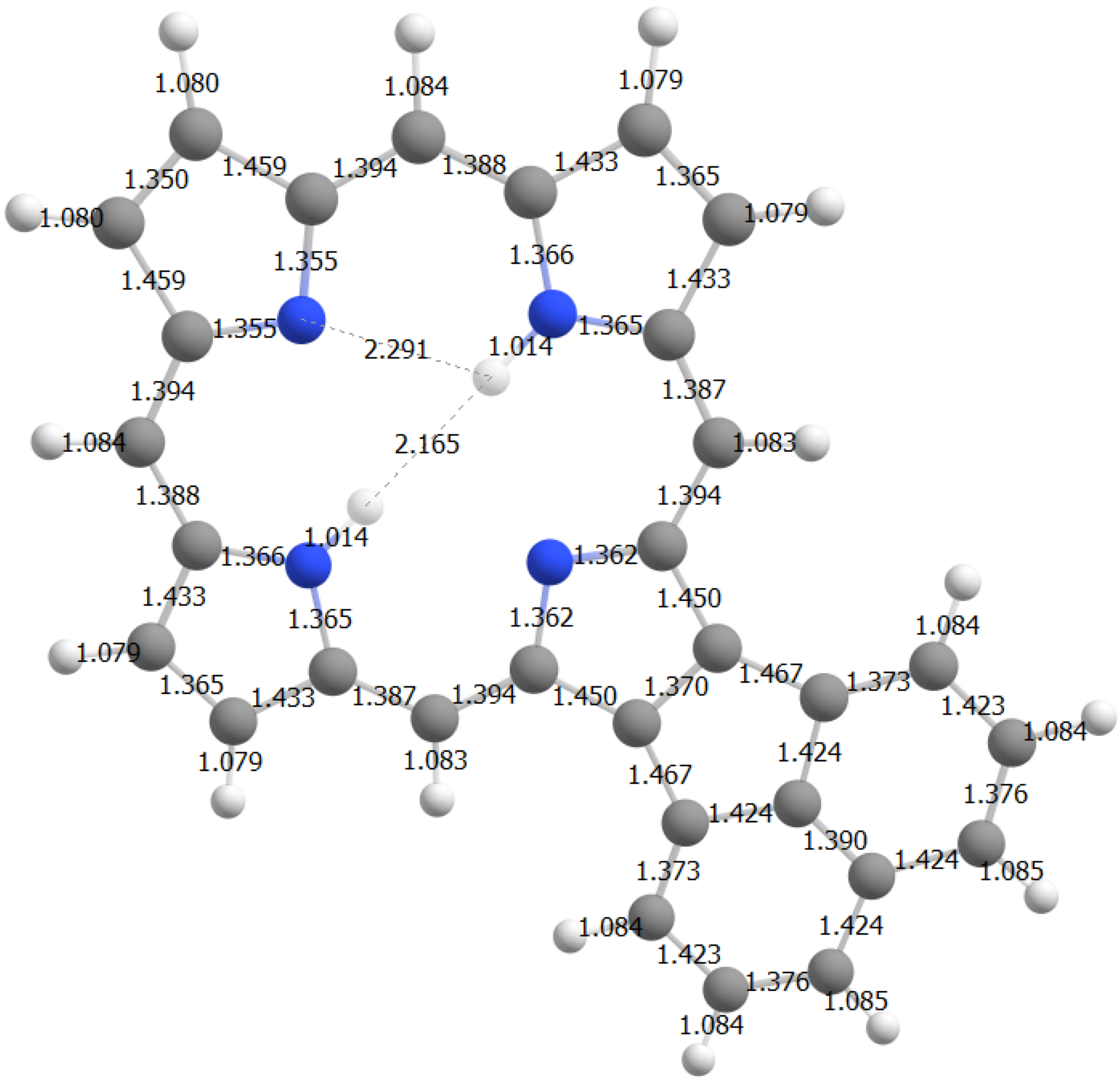

Figure S96. DFT calculated bond lengths for acenaphthoporphyrin tautomer **ANPb**.

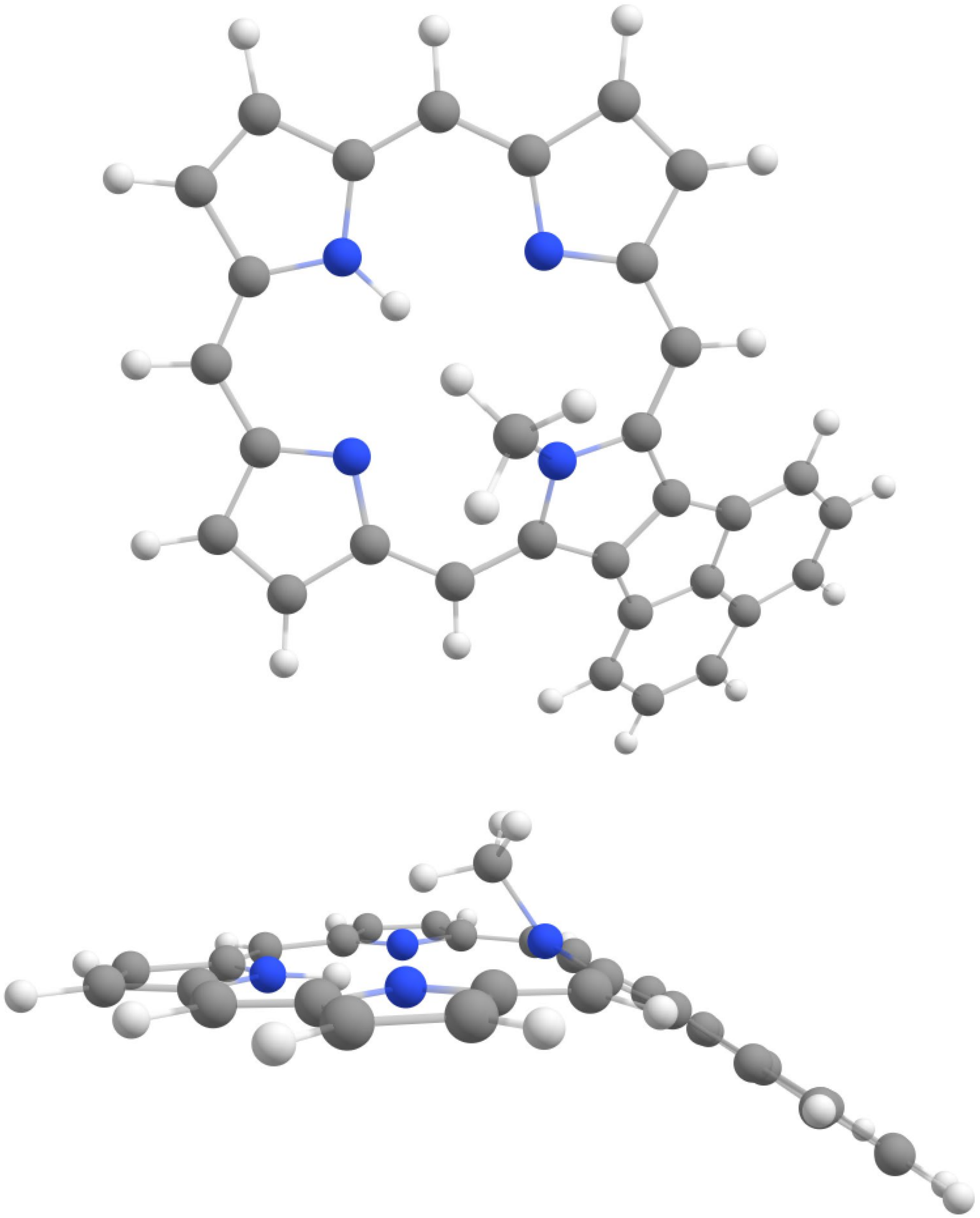

Figure S97. DFT calculated conformation (2 views) for *N*-methyl acenaphthoporphyrin tautomer **MeANPa**.

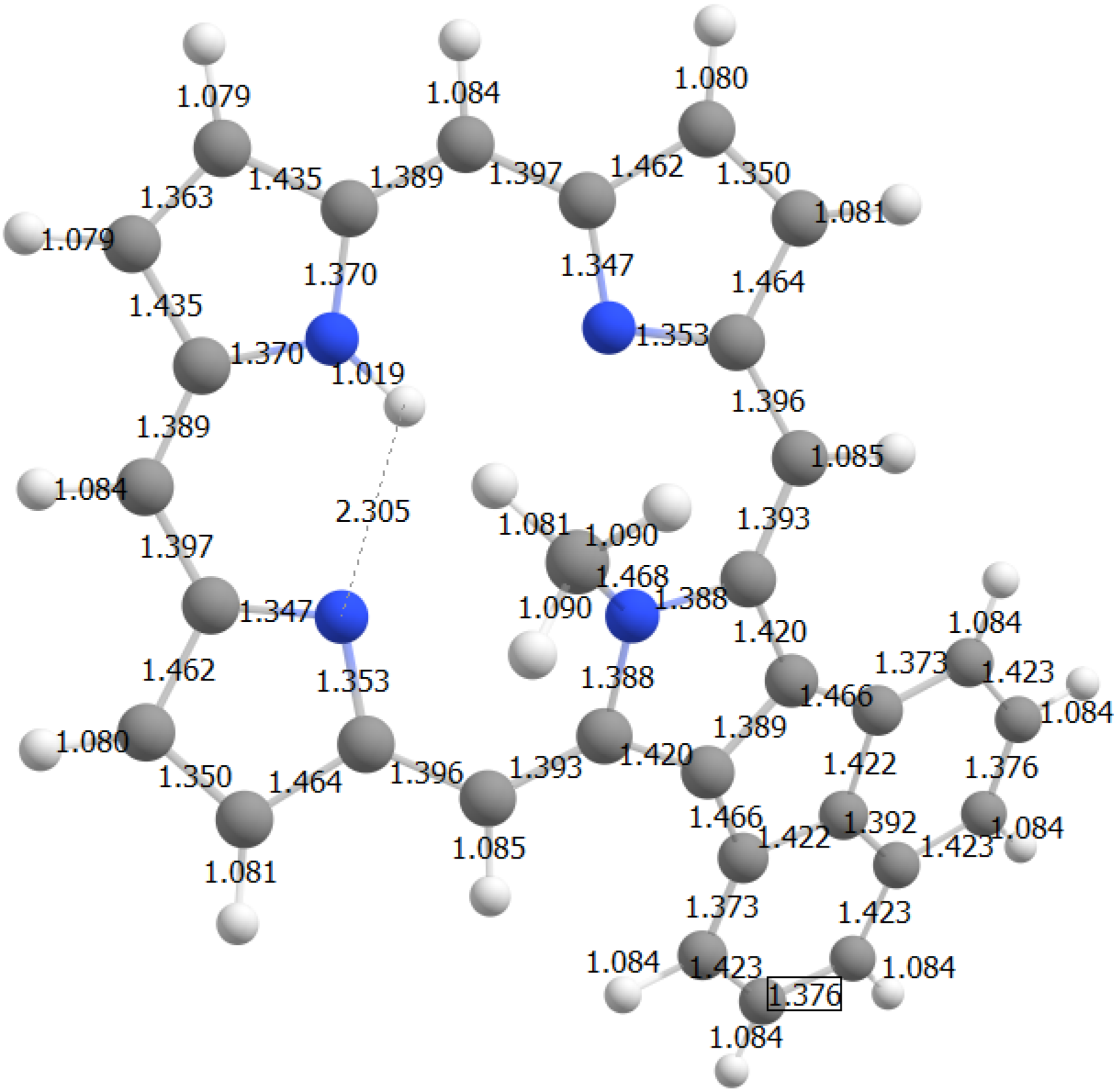

Figure S98. DFT calculated bond lengths for *N*-methyl acenaphthoporphyrin tautomer **MeANPa**.

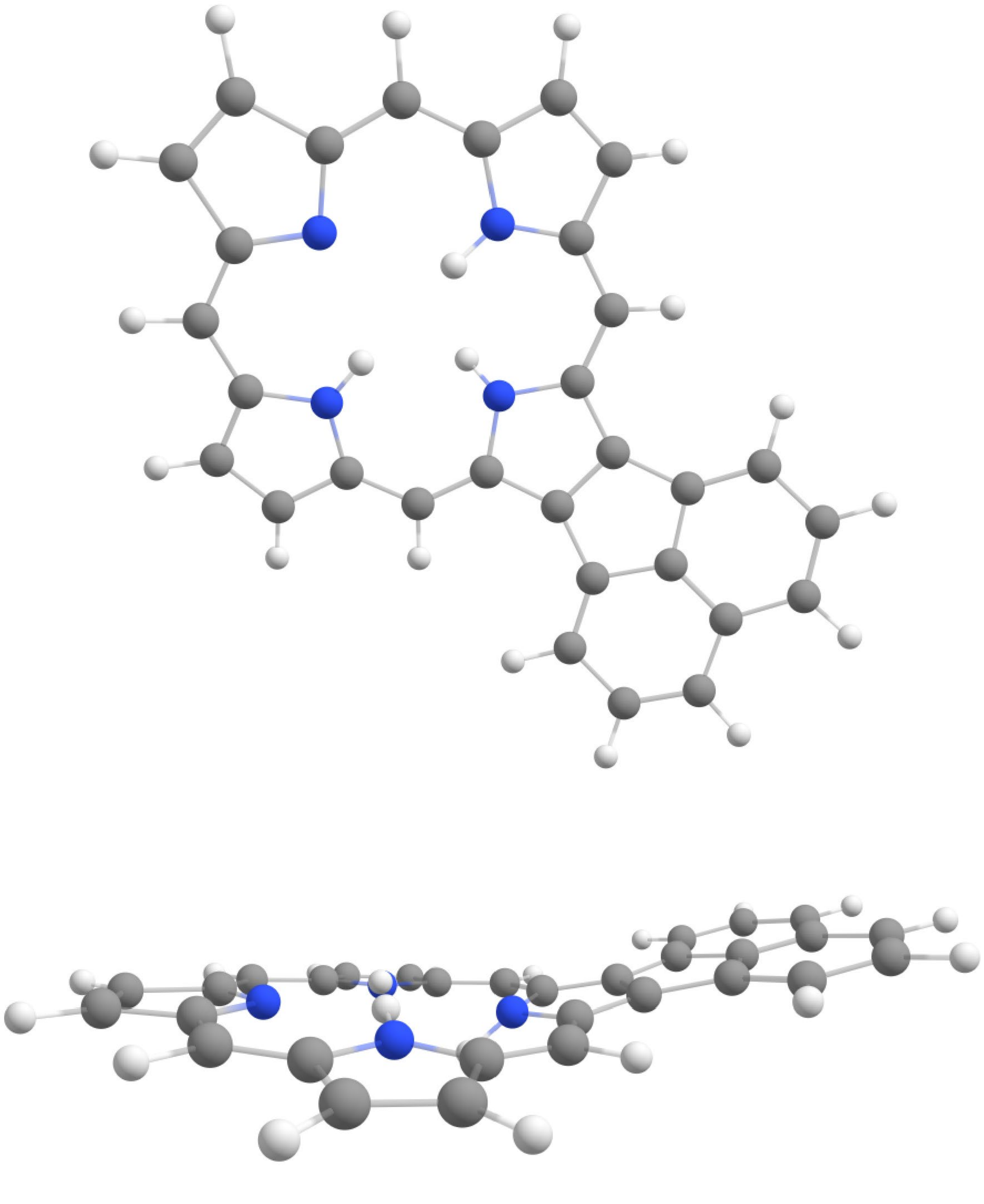

Figure S99. DFT calculated conformation (2 views) for acenaphthoporphyrin cation **ANPb**H^+**.**

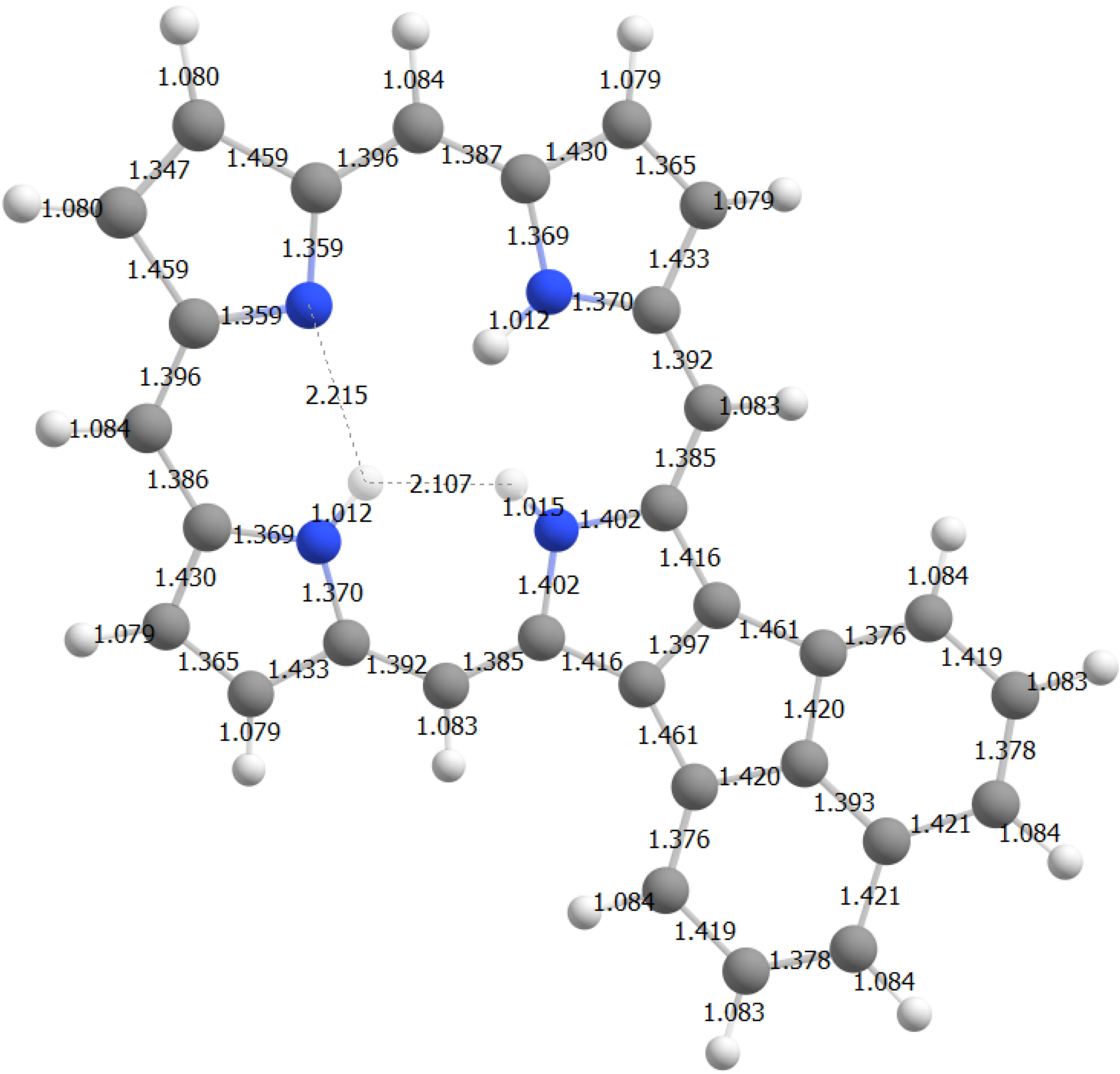

Figure S100. DFT calculated bond length for acenaphthoporphyrin cation **ANPb**H+**.**

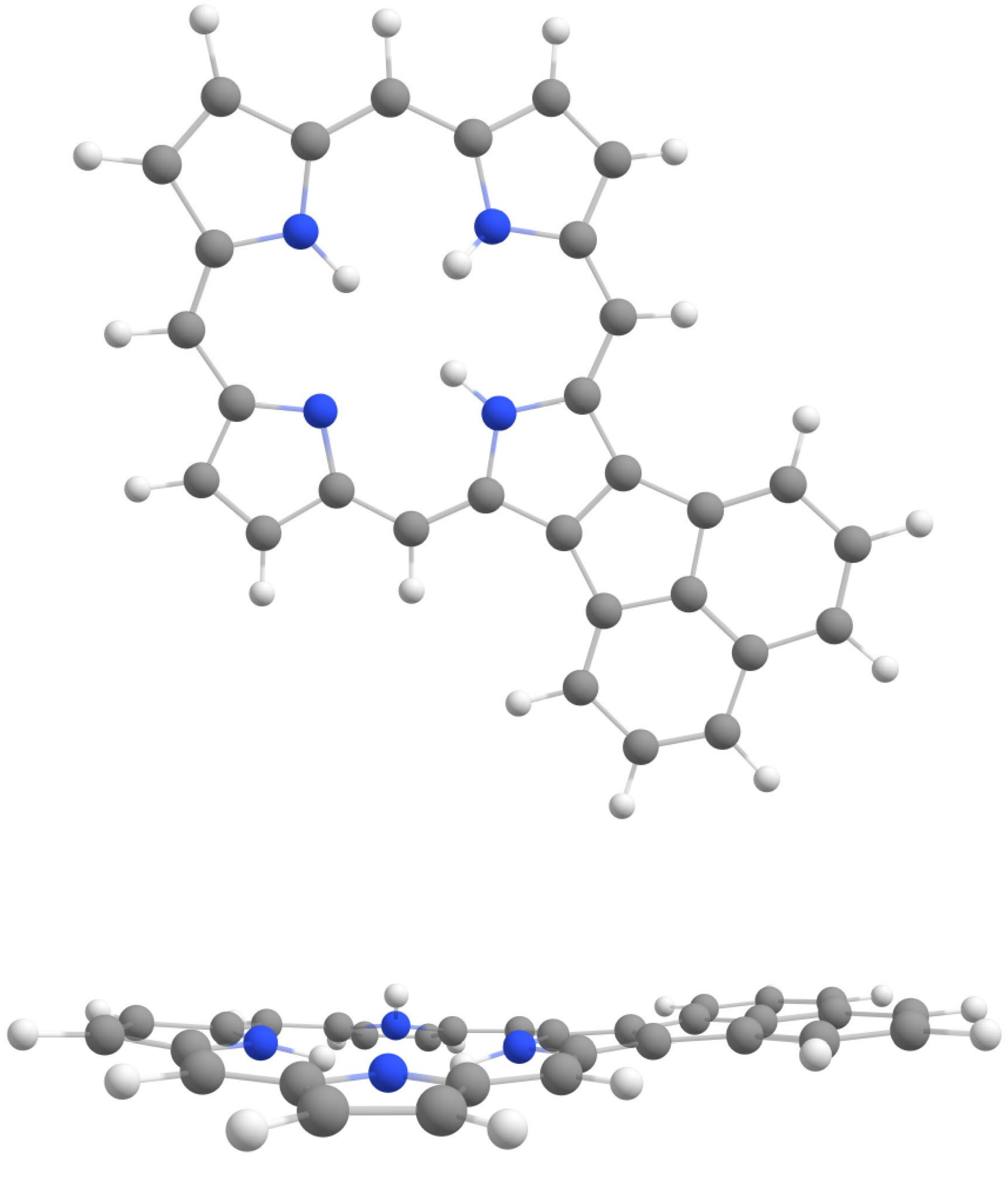

Figure S101. DFT calculated conformation (2 views) for acenaphthoporphyrin cation **ANPc**H+**.**

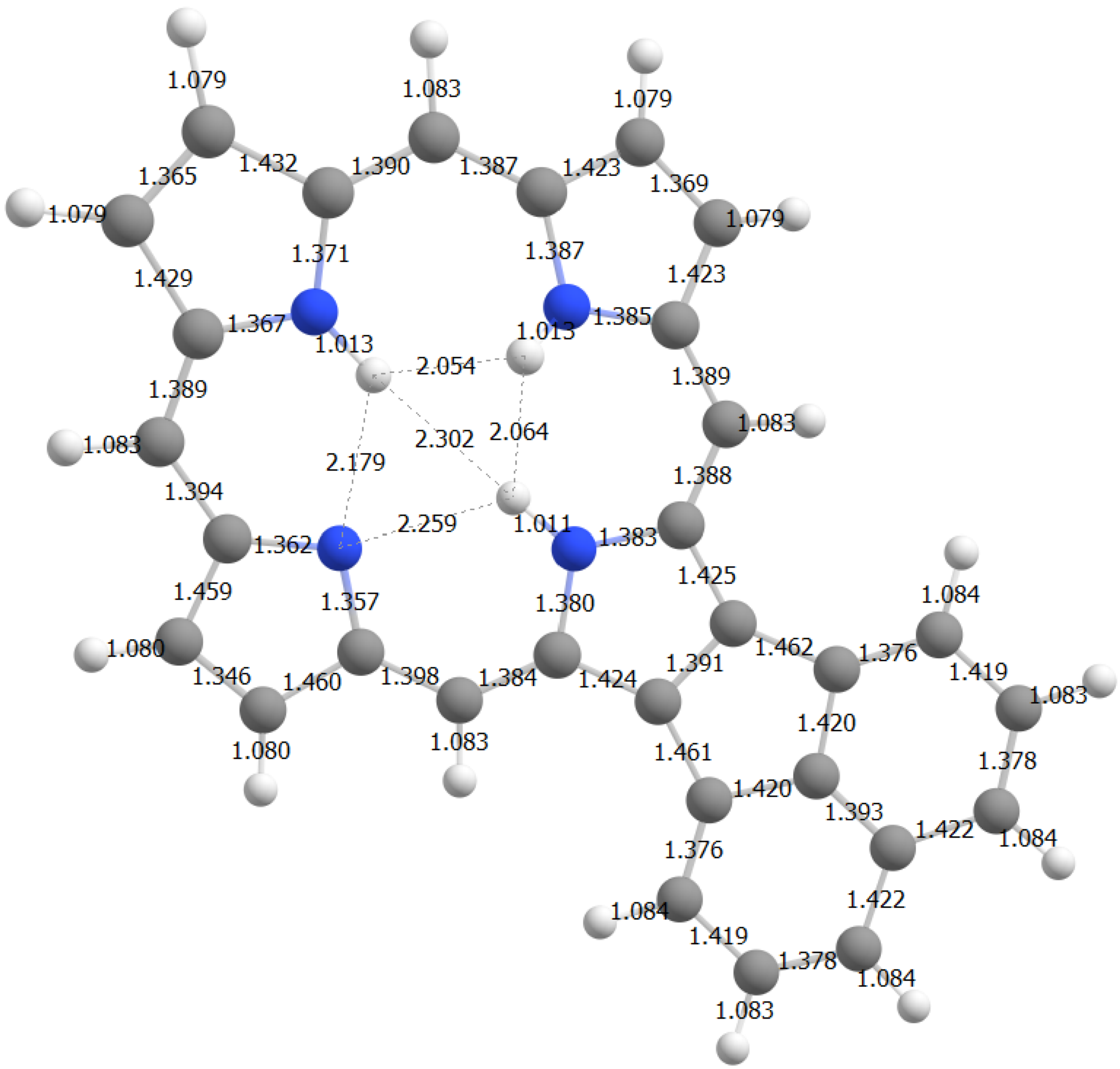

Figure S102. DFT calculated bond lengths for acenaphthoporphyrin cation **ANPc**H^{+}**.**

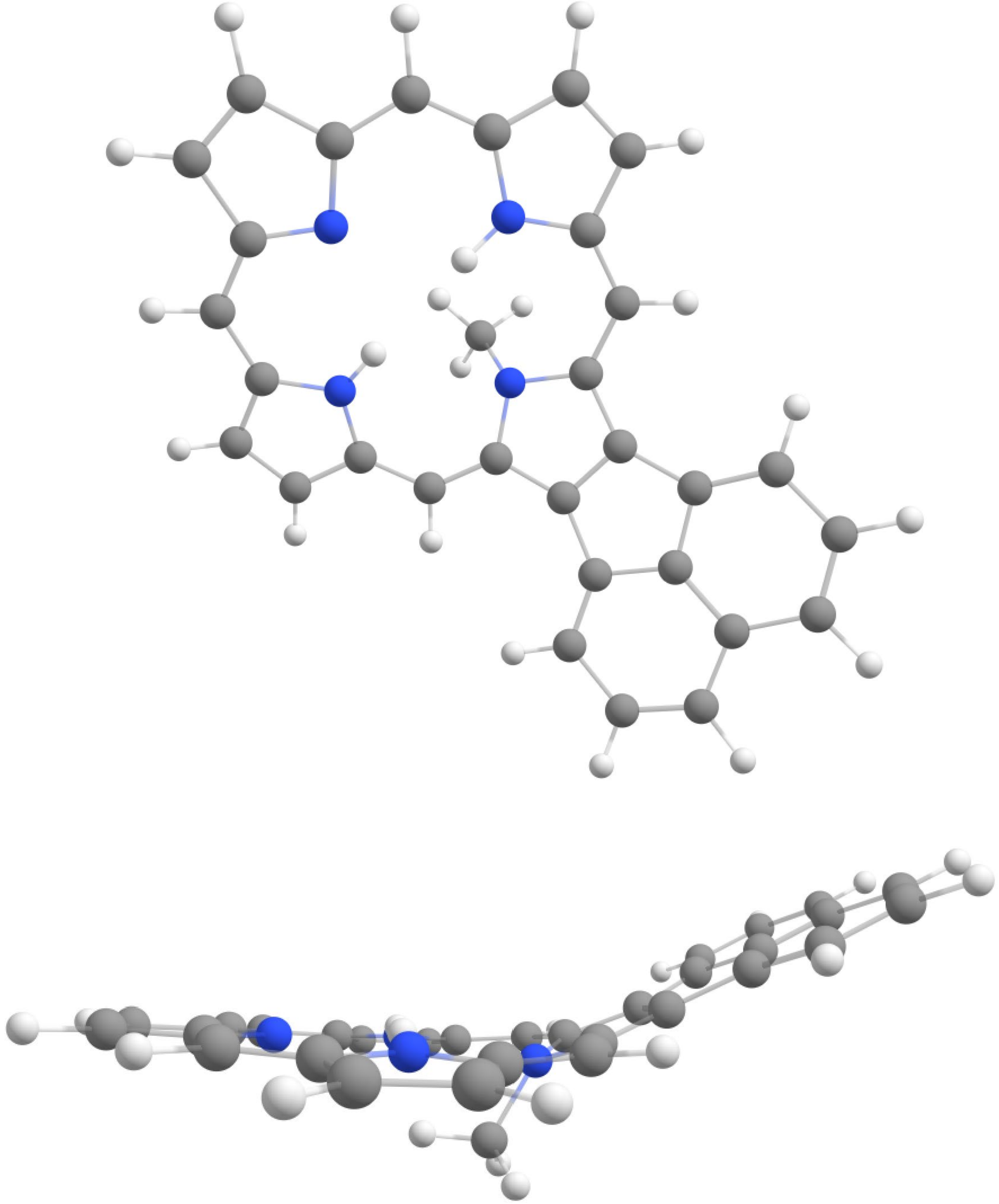

Figure S103. DFT calculated conformation (2 views) for *N*-methylacenaphthoporphyrin cation **MeANPb**H$^+$**.**

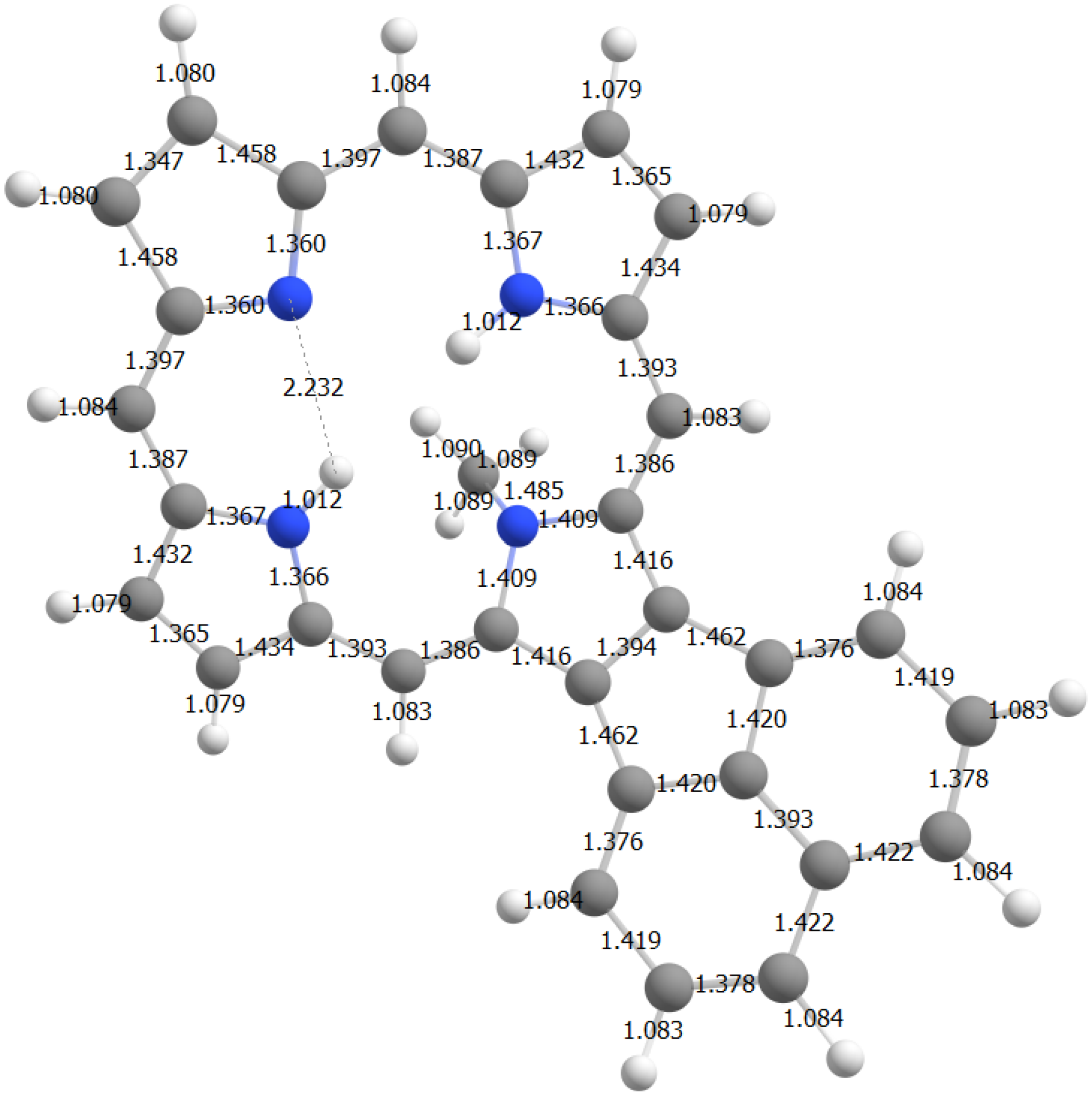

Figure S104. DFT calculated bond lengths for *N*-methylacenaphthoporphyrin cation **MeANPb**H^{+}**.**

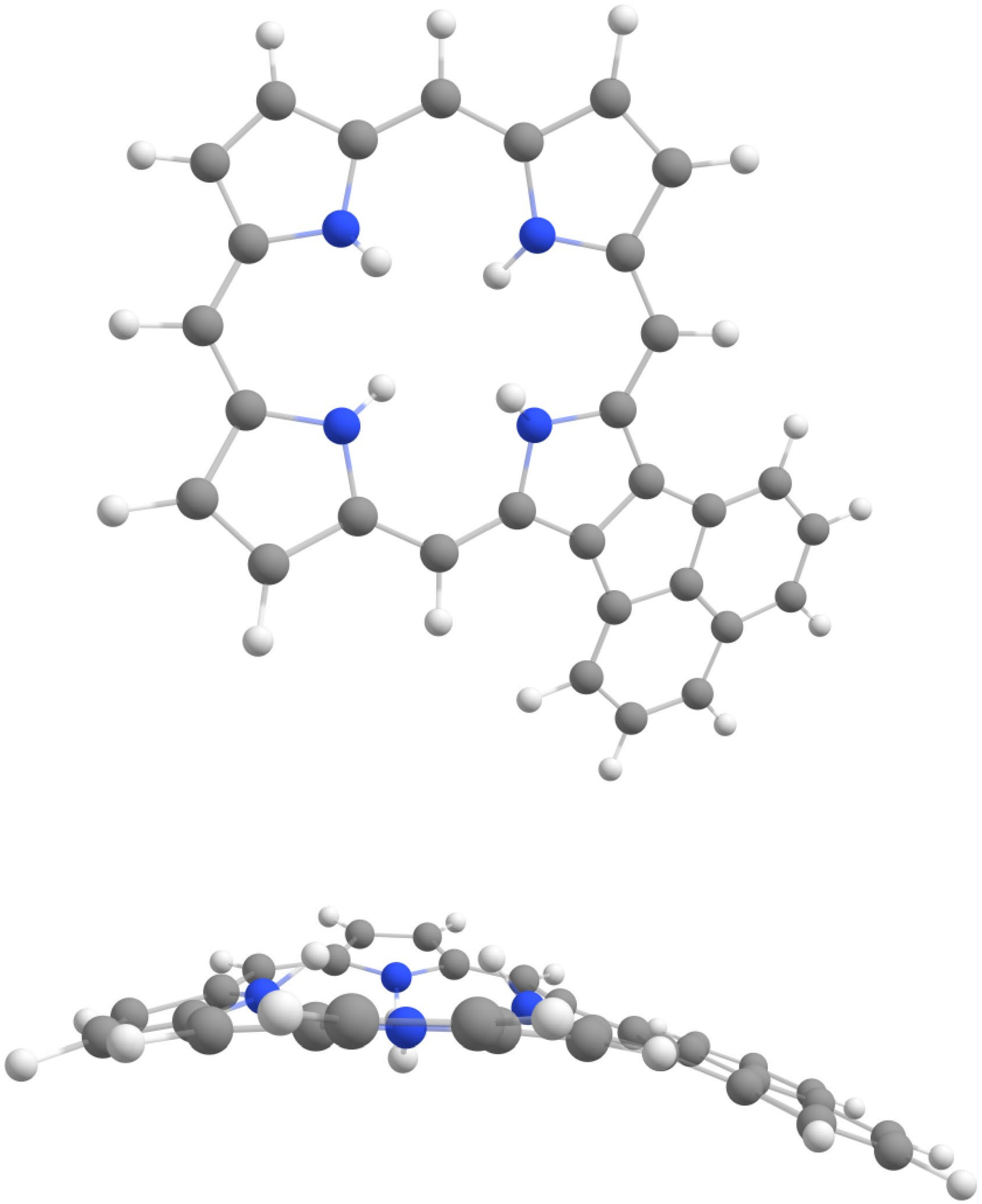

Figure S105. DFT calculated conformation (2 views) for acenaphthoporphyrin dication **ANP**H_2^{2+}**.**

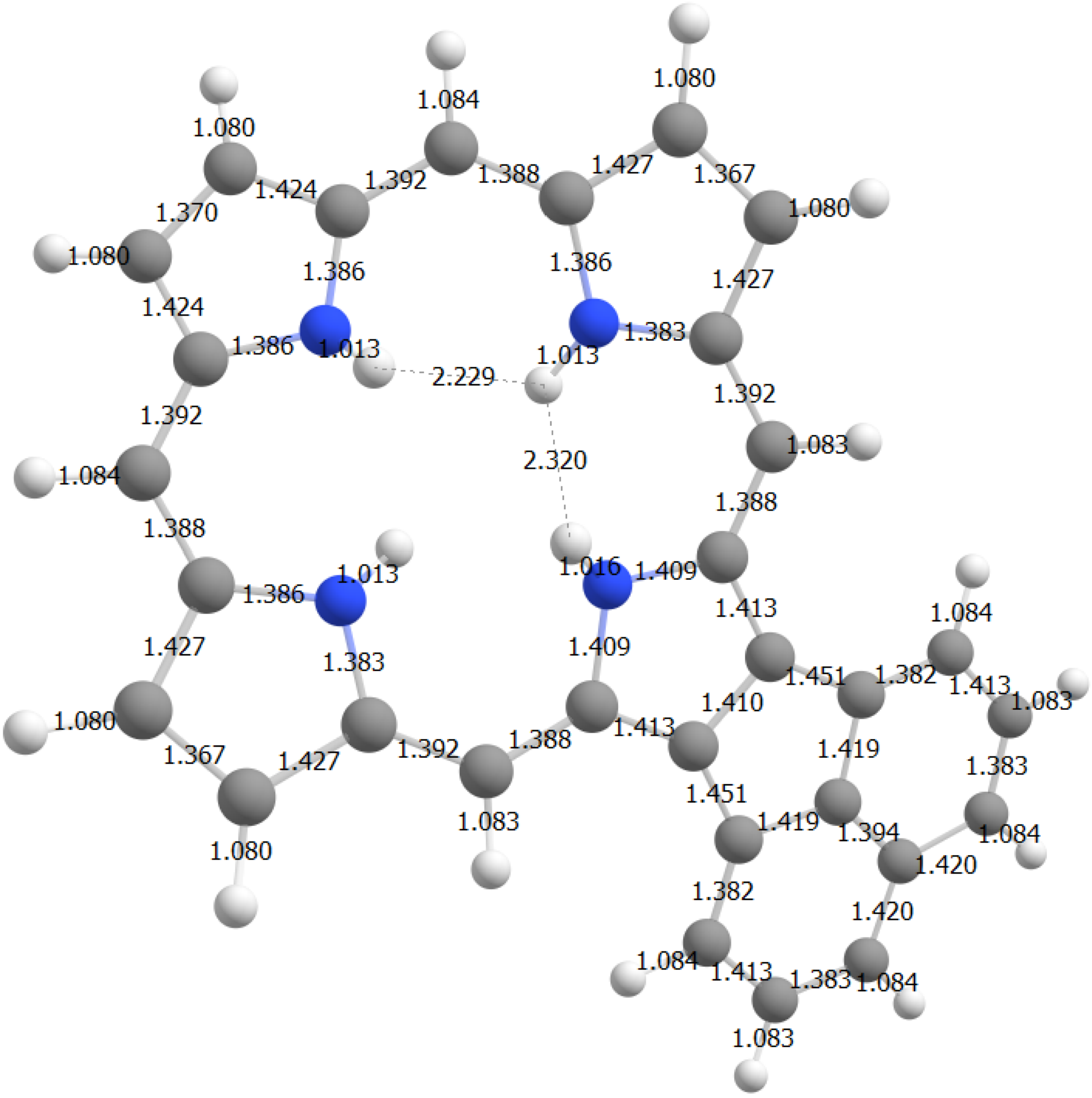

Figure S106. DFT calculated bond lengths for acenaphthoporphyrin dication **ANP**H_2^{2+}**.**

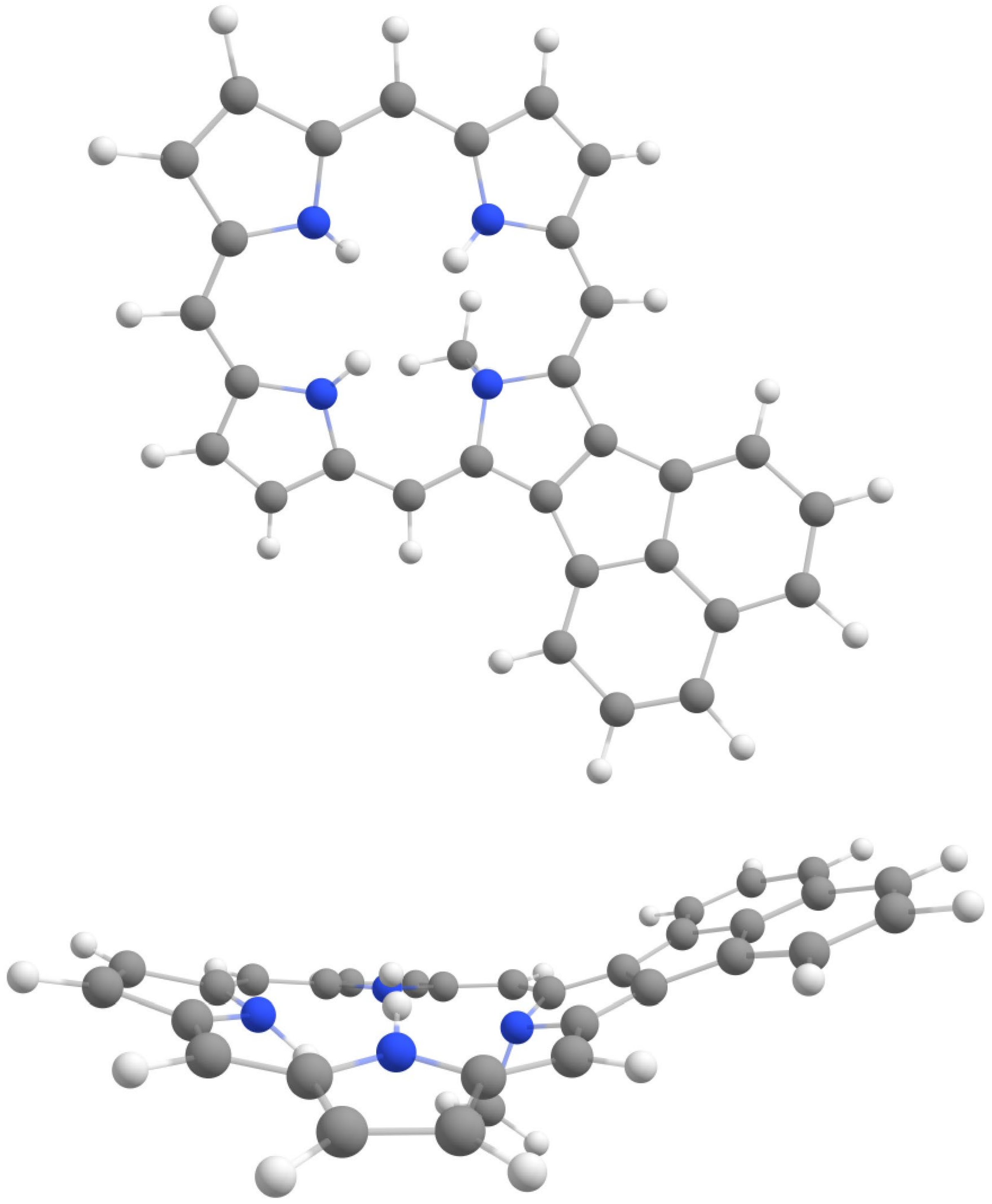

Figure S107. DFT calculated conformation (2 views) for *N*-methylacenaphthoporphyrin dication **MeANP**H_2^{2+}.

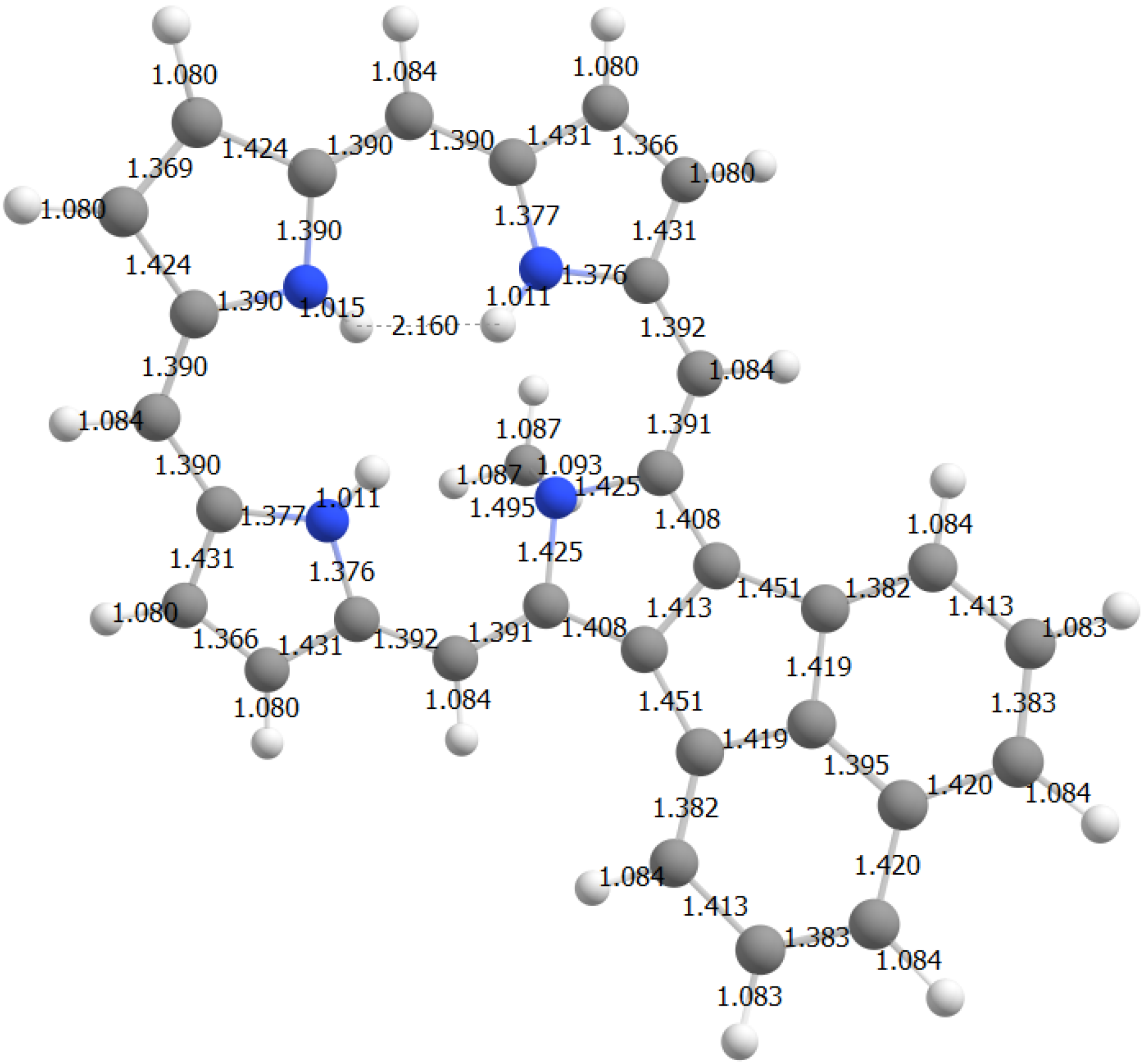

Figure S108. DFT calculated bond lengths for *N*-methylacenaphthoporphyrin dication **MeNP**H_2^{2+}**.**

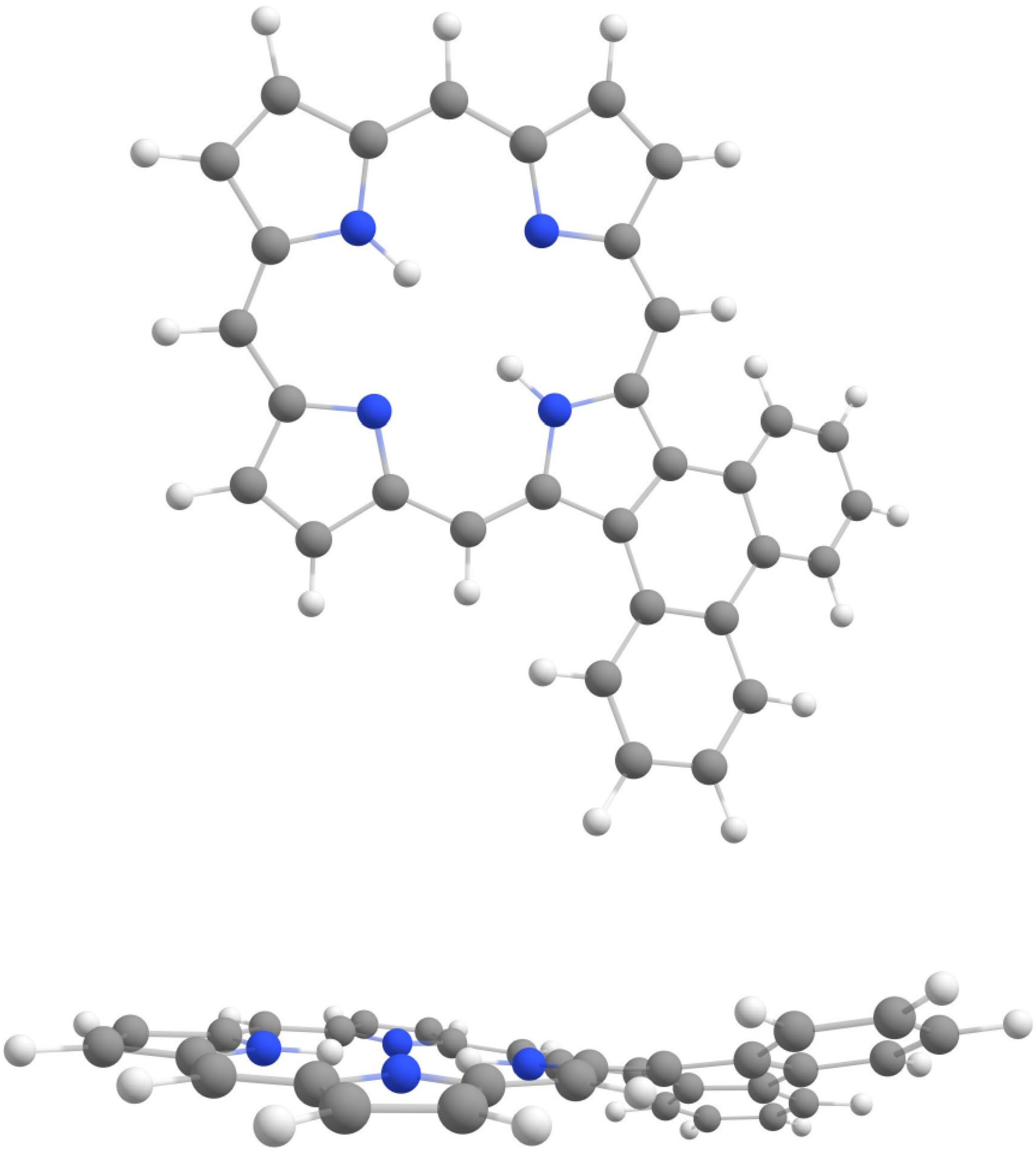

Figure S109. DFT calculated conformation (2 views) for phenanthroporphyrin tautomer **PhPa.**

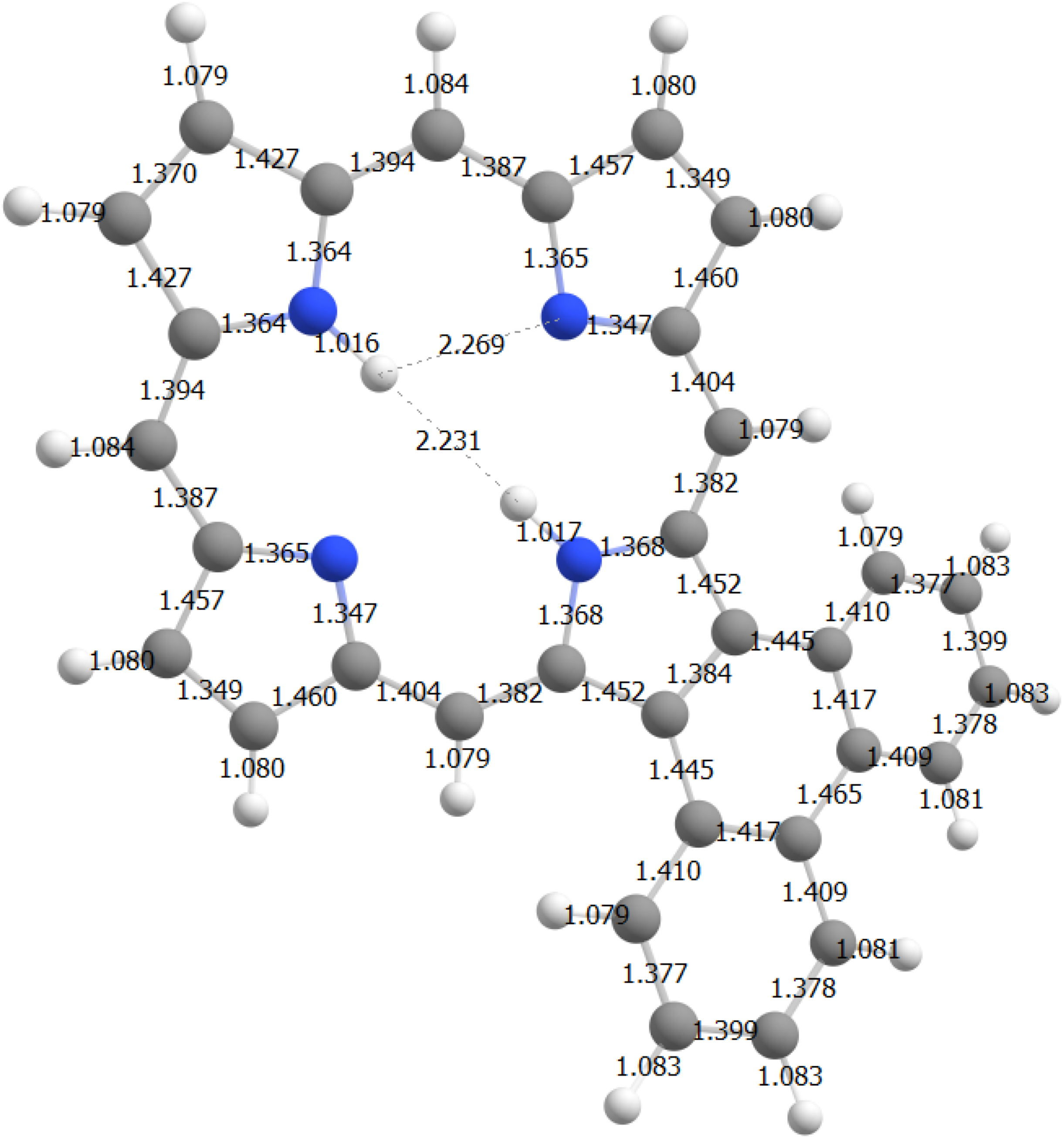

Figure S110. DFT calculated bond lengths for phenanthroporphyrin tautomer **PhPa.**

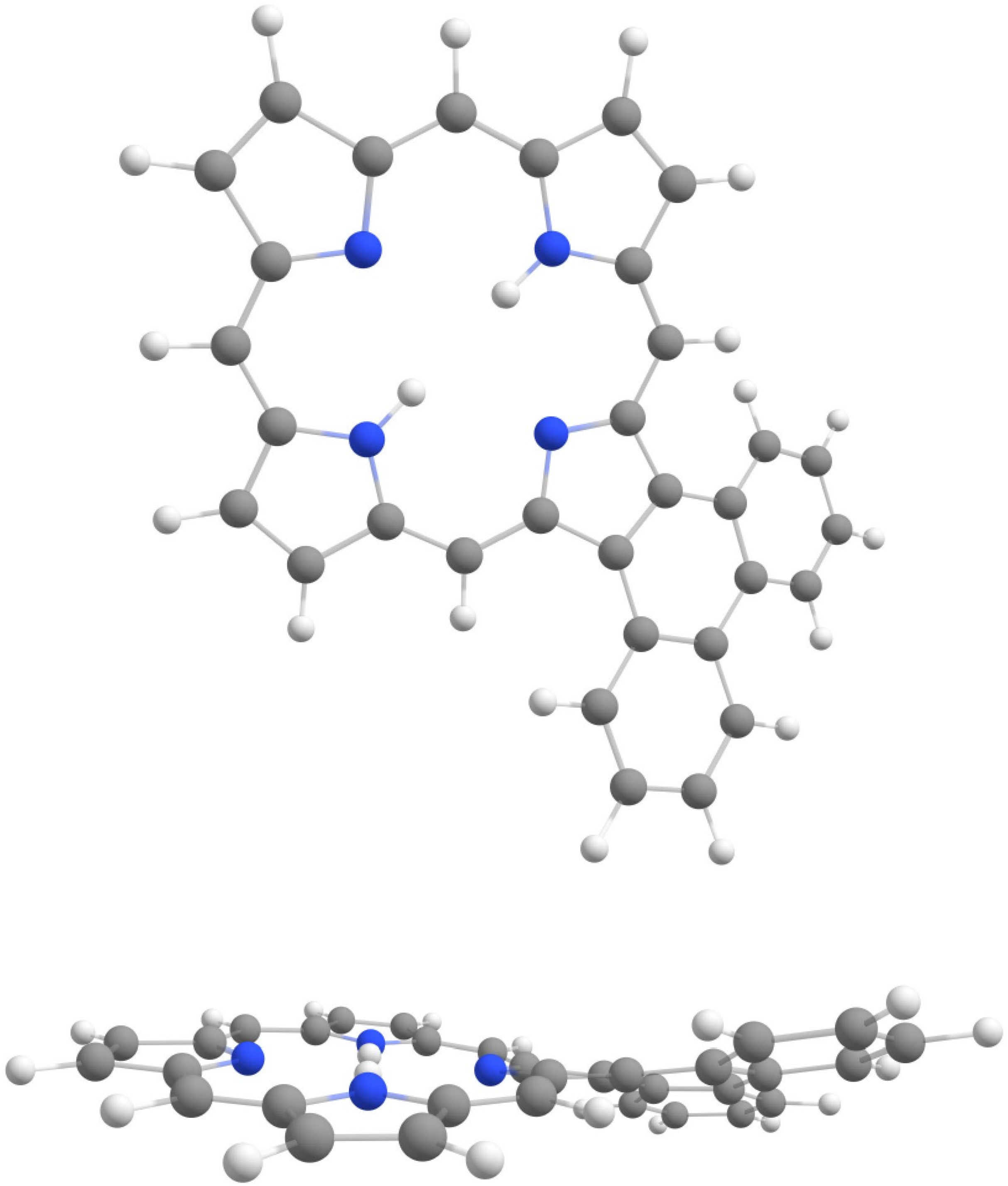

Figure S111. DFT calculated conformation (2 views) for phenanthroporphyrin tautomer **PhPb.**

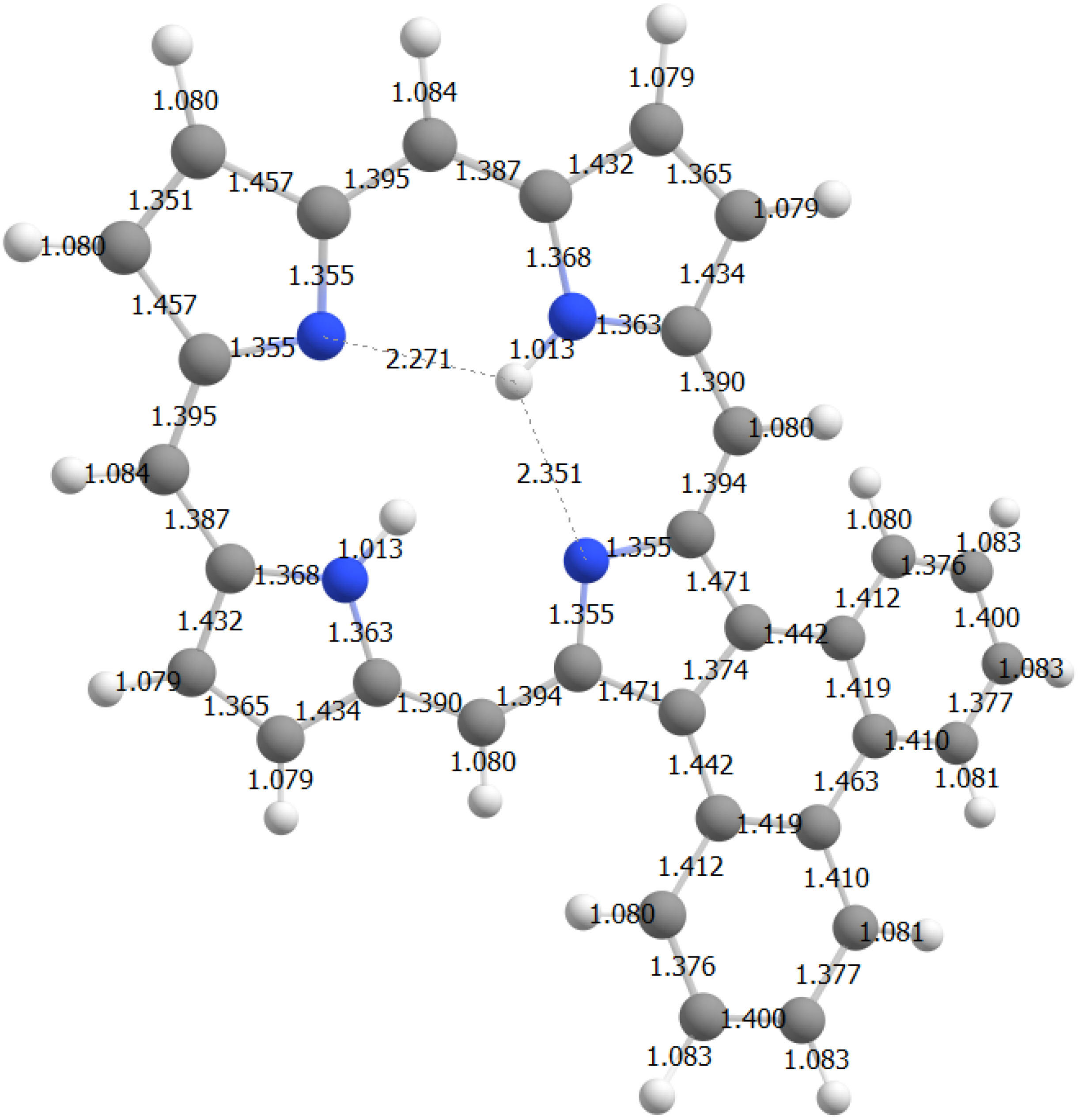

Figure S112. DFT calculated bond lengths for phenanthroporphyrin tautomer **PhPb.**

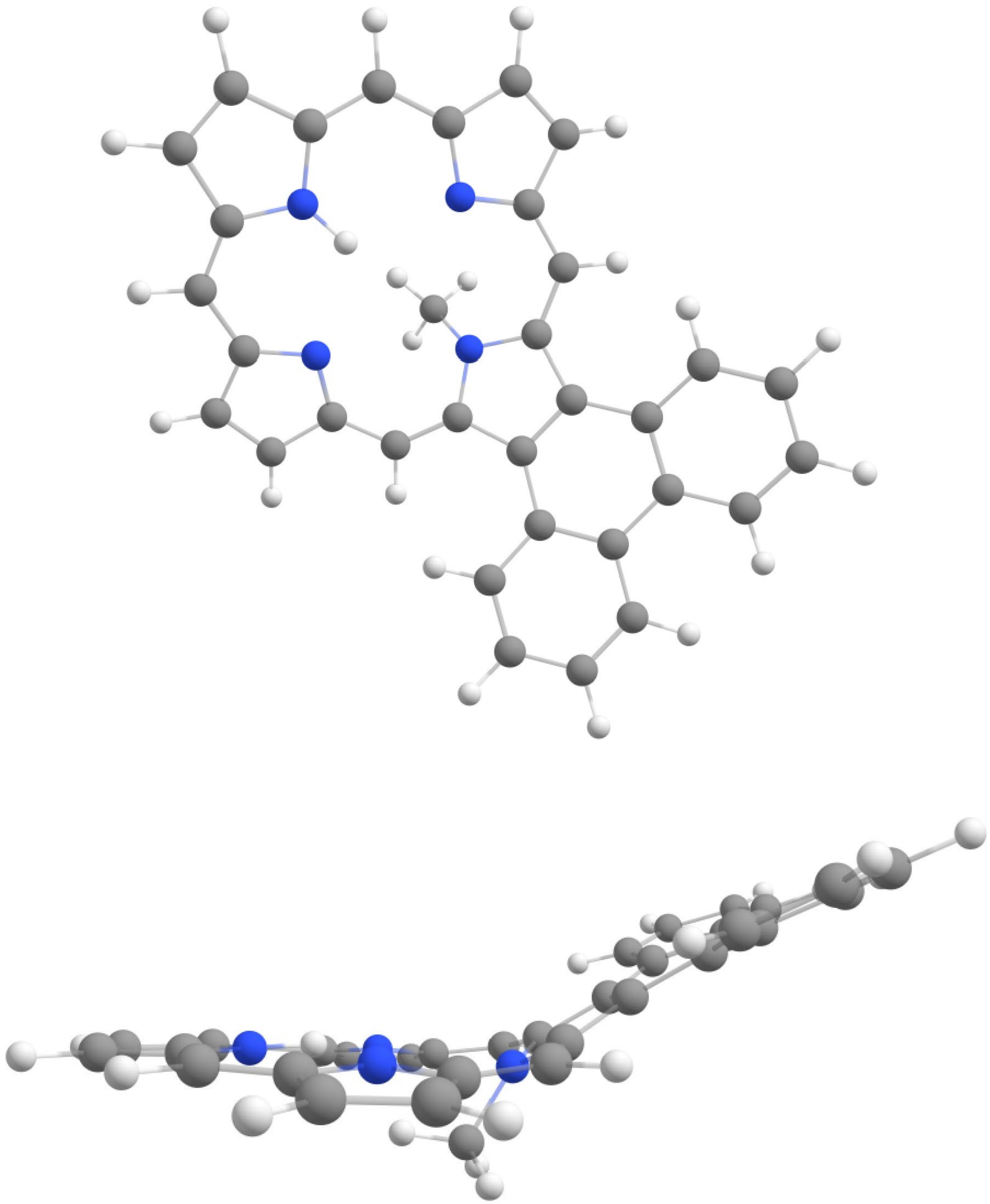

Figure S113. DFT calculated conformation (2 views) for *N*-methylphenanthroporphyrin tautomer **MePhPa.**

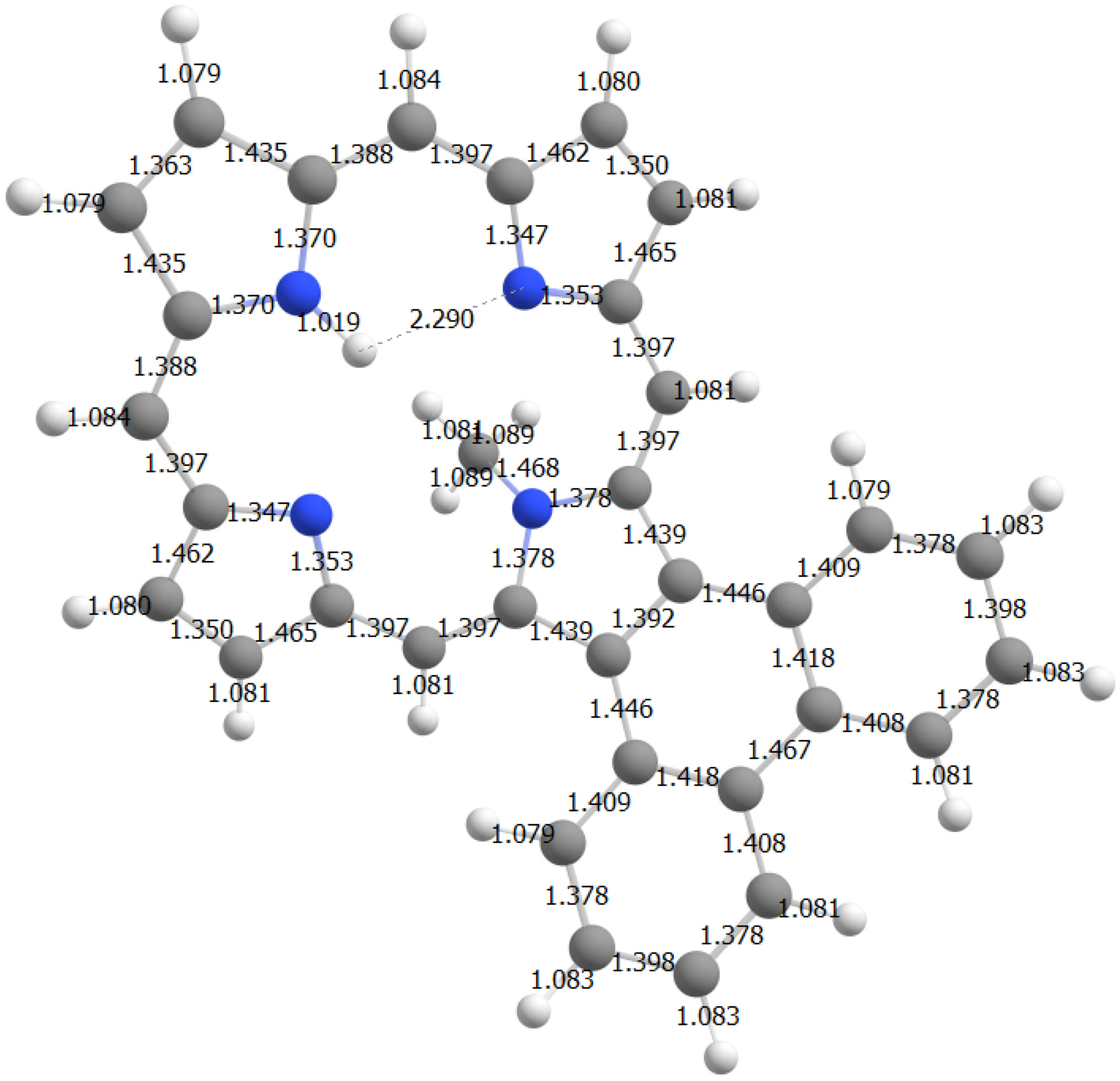

Figure S114. DFT calculated bond lengths for *N*-methylphenanthroporphyrin tautomer **MePhPa.**

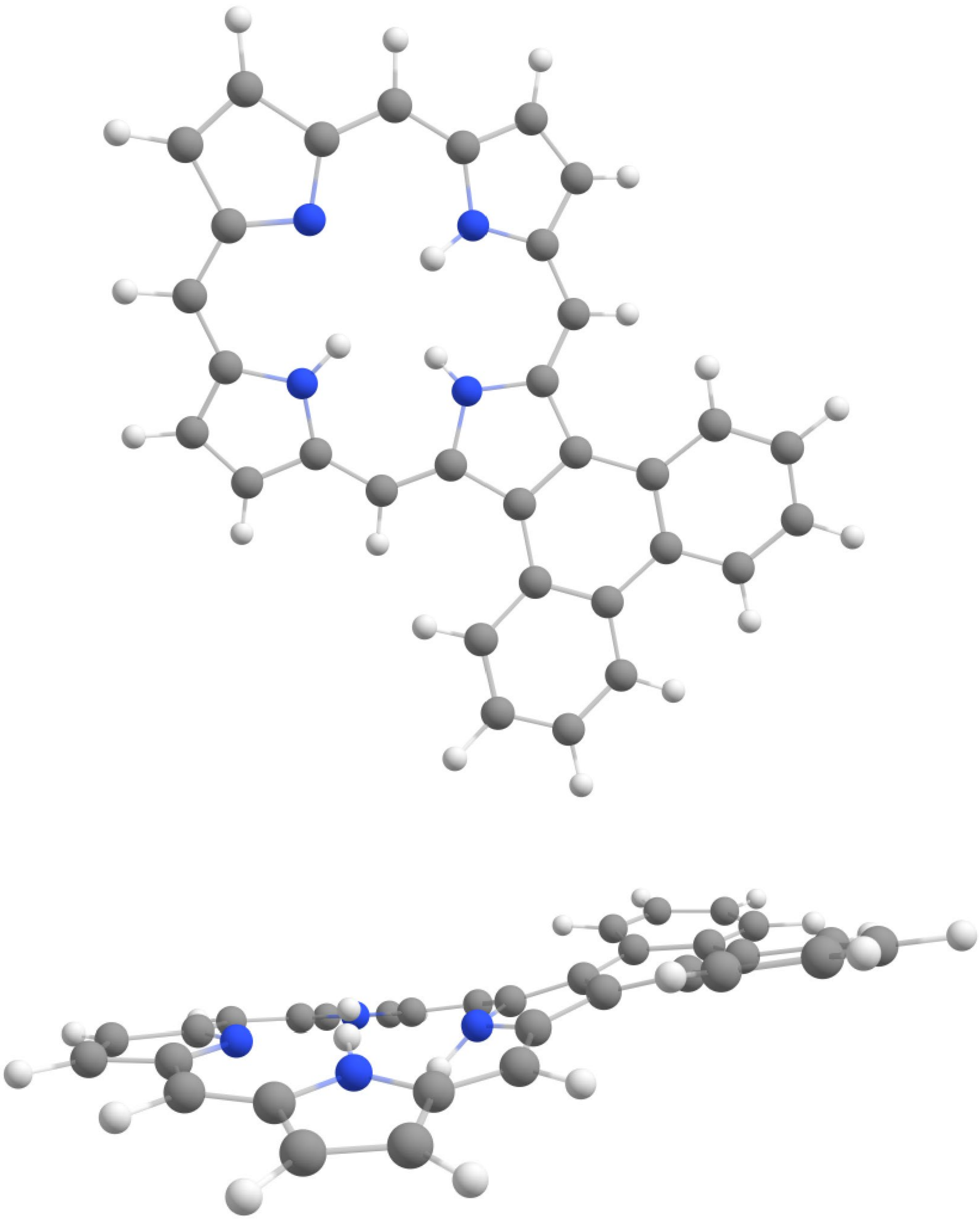

Figure S115. DFT calculated conformation (2 views) for phenanthroporphyrin cation **PhPb**H$^+$.

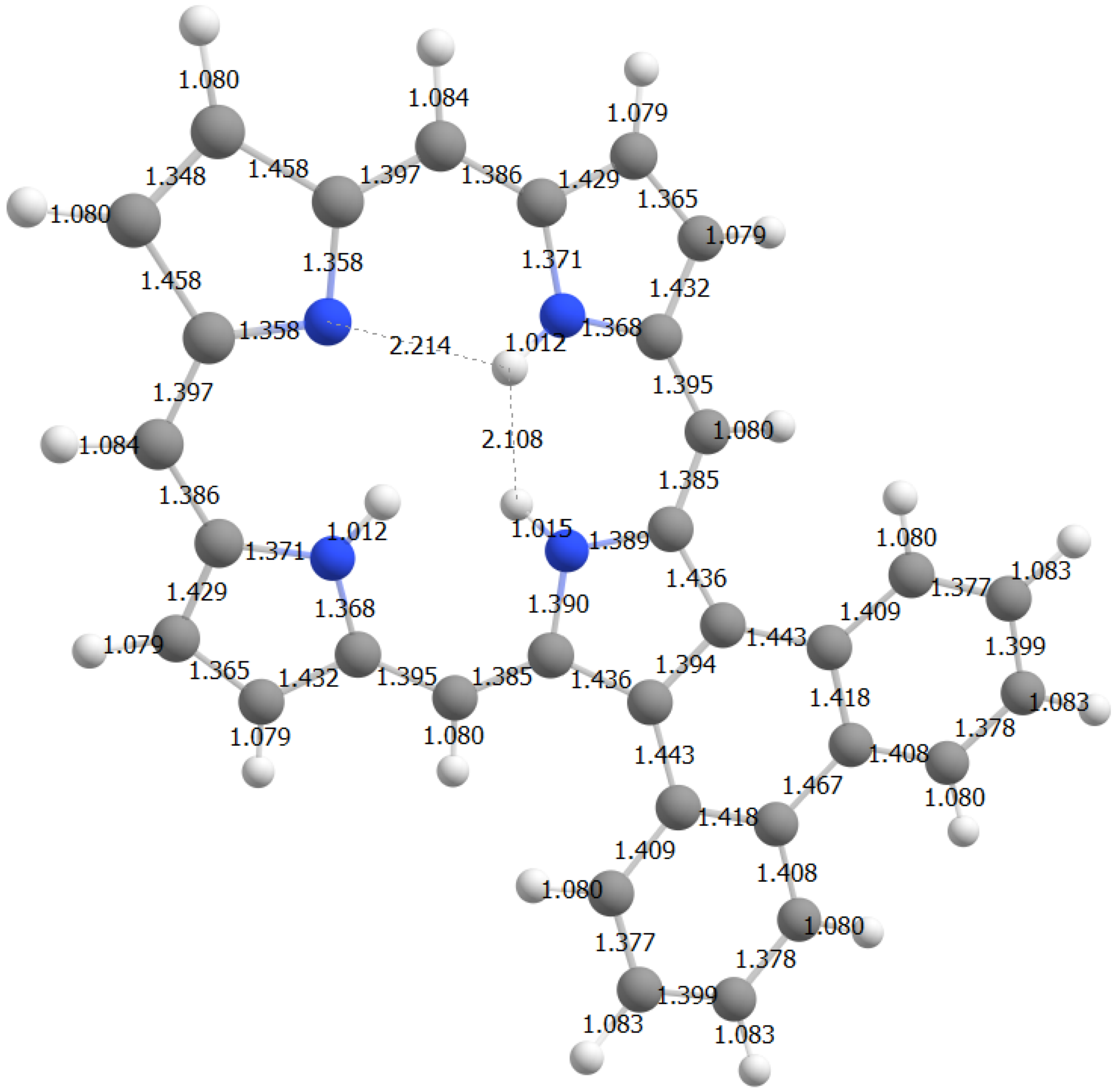

Figure S116. DFT calculated conformation (2 views) for phenanthroporphyrin cation **PhPb**H^{+}.

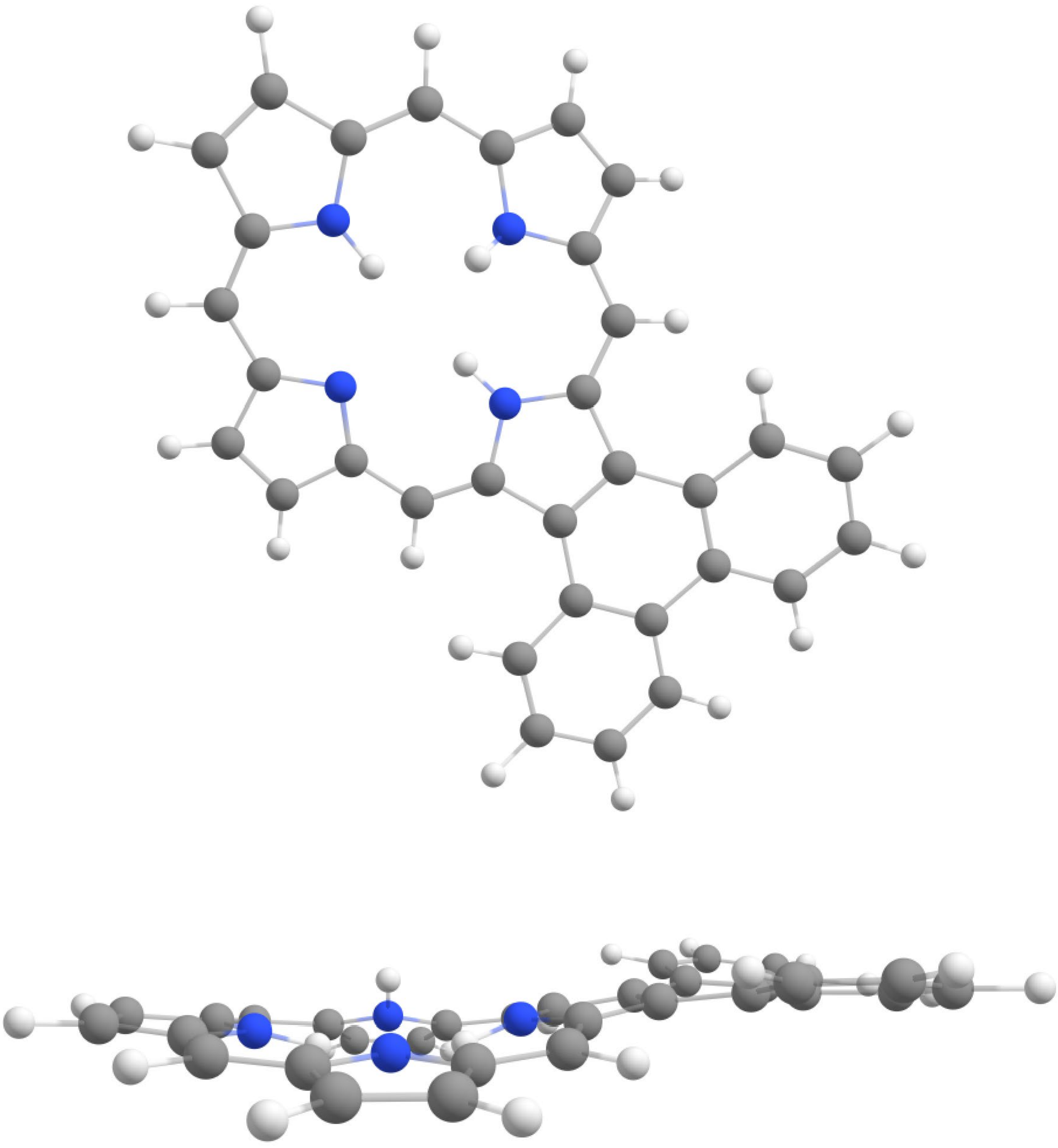

Figure S117. DFT calculated conformation (2 views) for phenanthroporphyrin cation **PhPc**H^{+}.

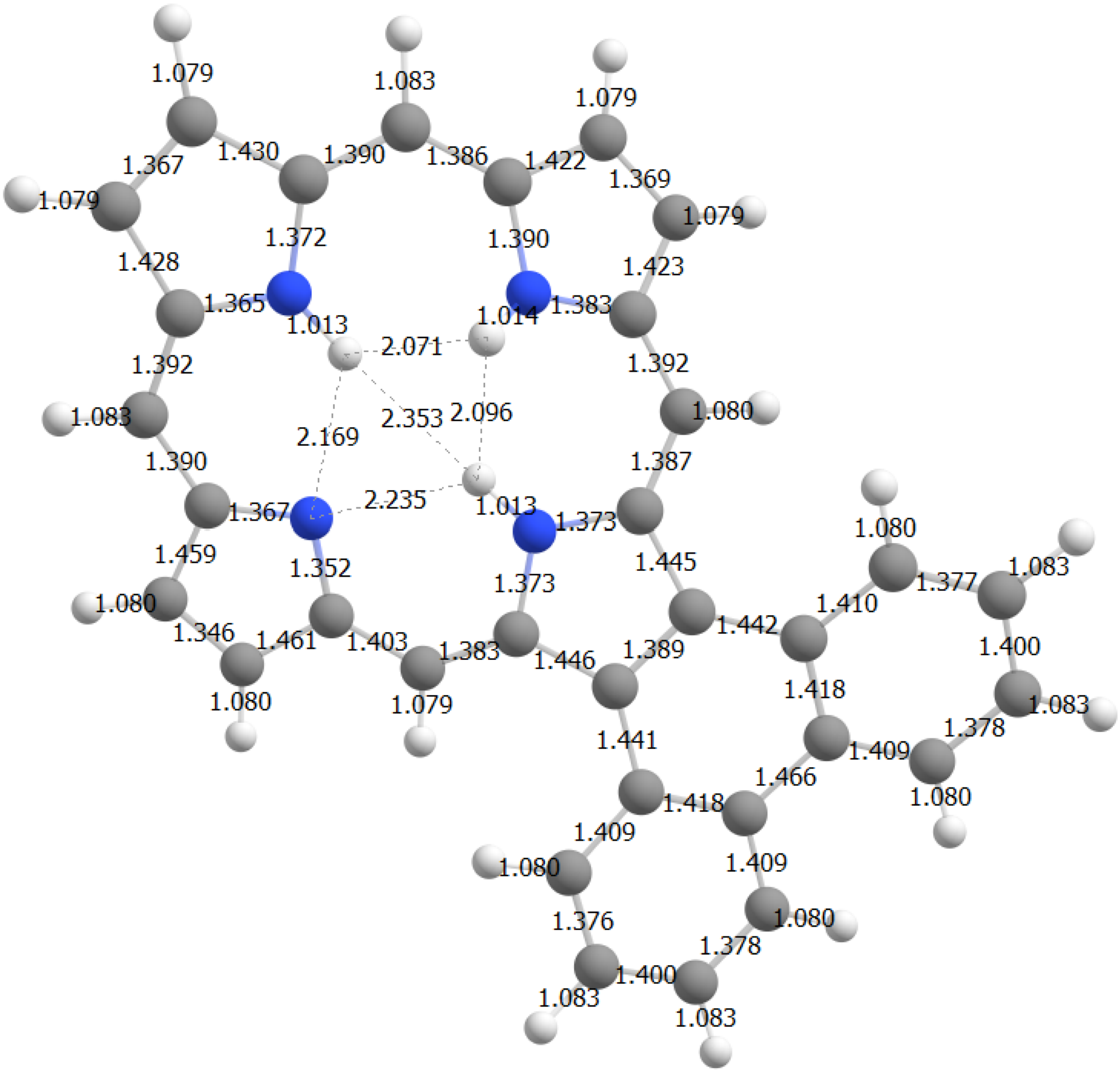

Figure S118. DFT calculated bond lengths for phenanthroporphyrin cation **PhPc**H^{+}.

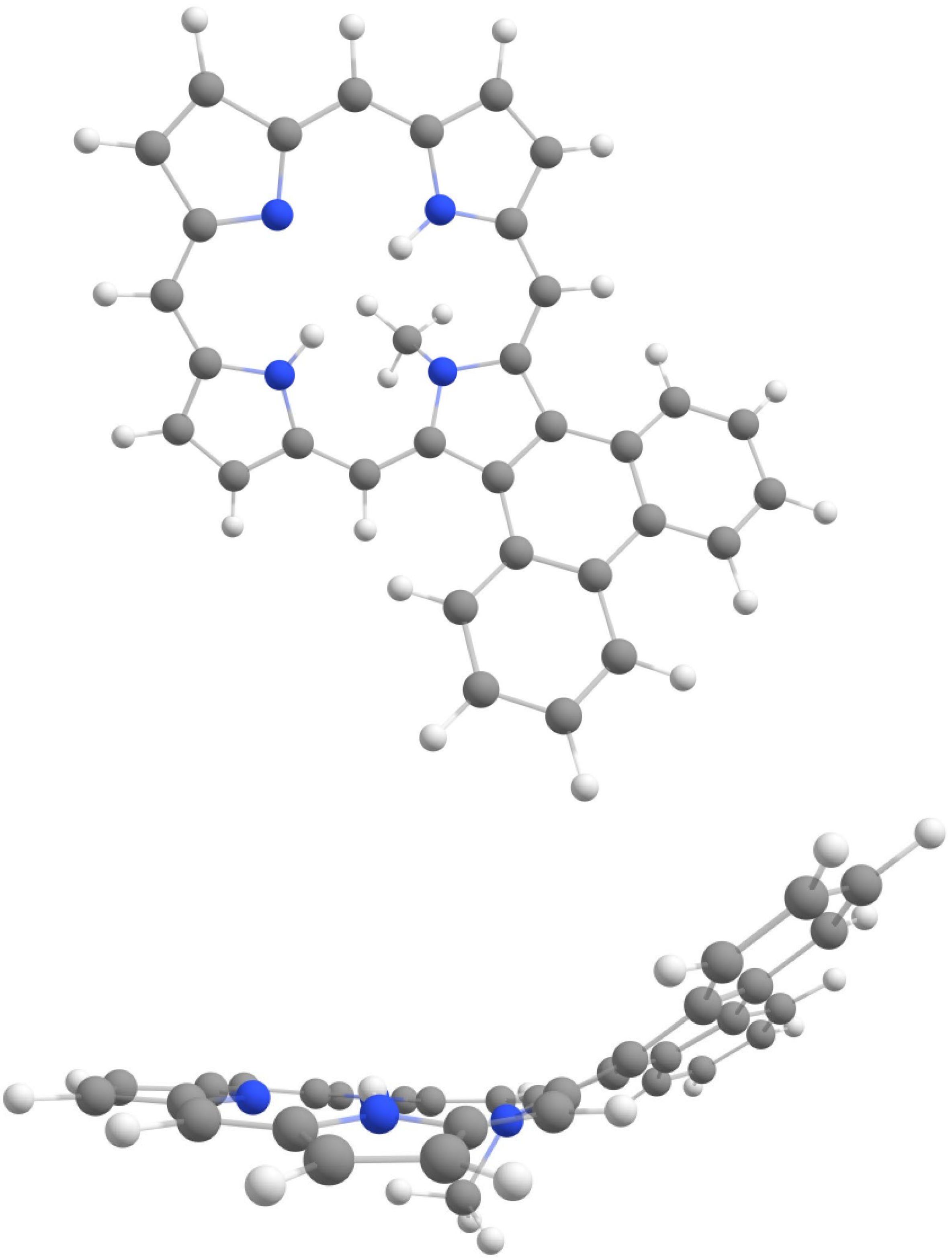

Figure S119. DFT calculated conformation (2 views) for *N*-methylphenanthroporphyrin cation **MePhPb**H^+.

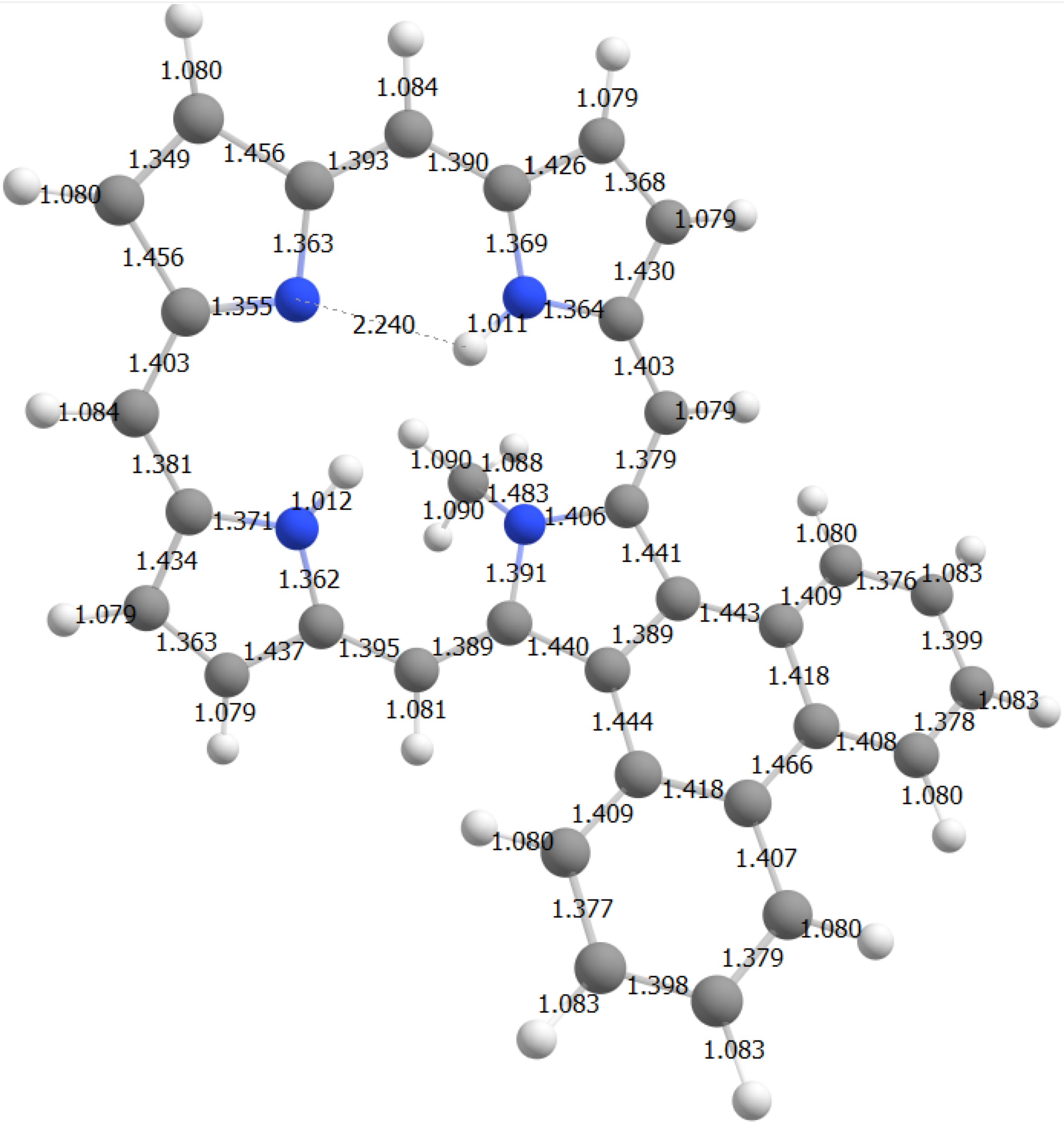

Figure S120. DFT calculated bond lengths for *N*-methylphenanthroporphyrin cation **MePhPb**H^+.

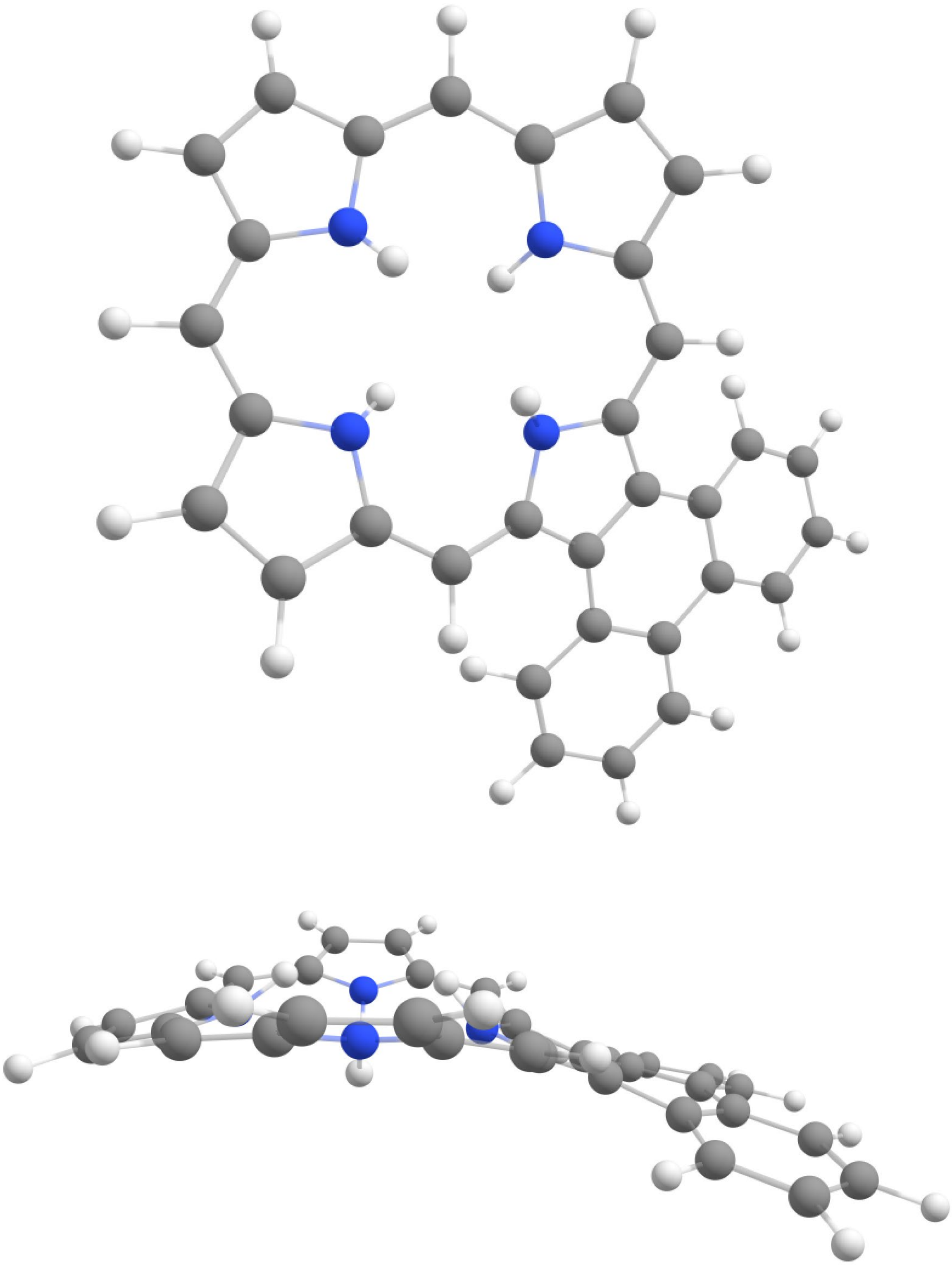

Figure S121. DFT calculated conformation (2 views) for phenanthroporphyrin dication **PhP**H_2^{2+}.

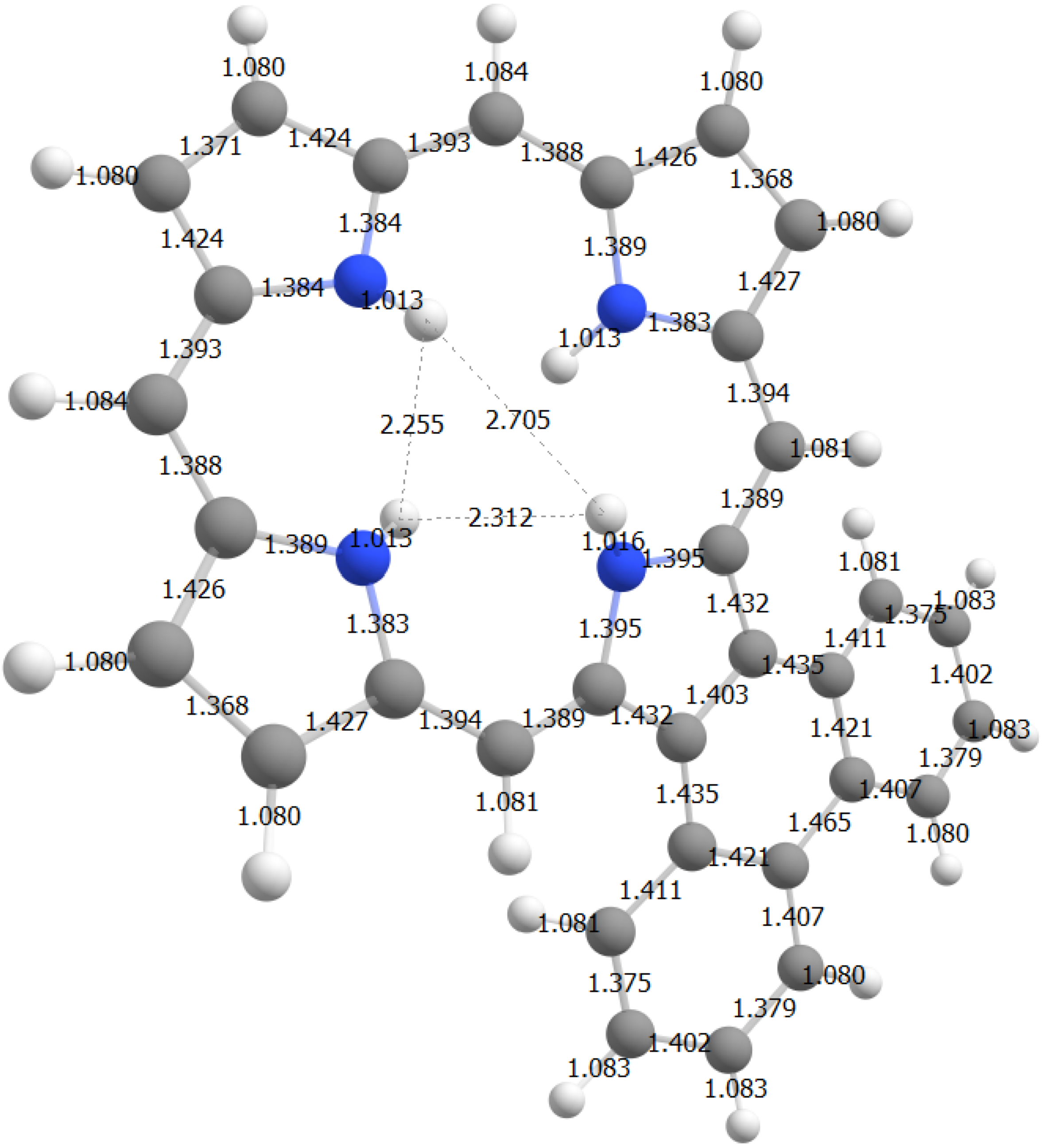

Figure S122. DFT calculated bond lengths for phenanthroporphyrin dication **PhP**H_2^{2+}.

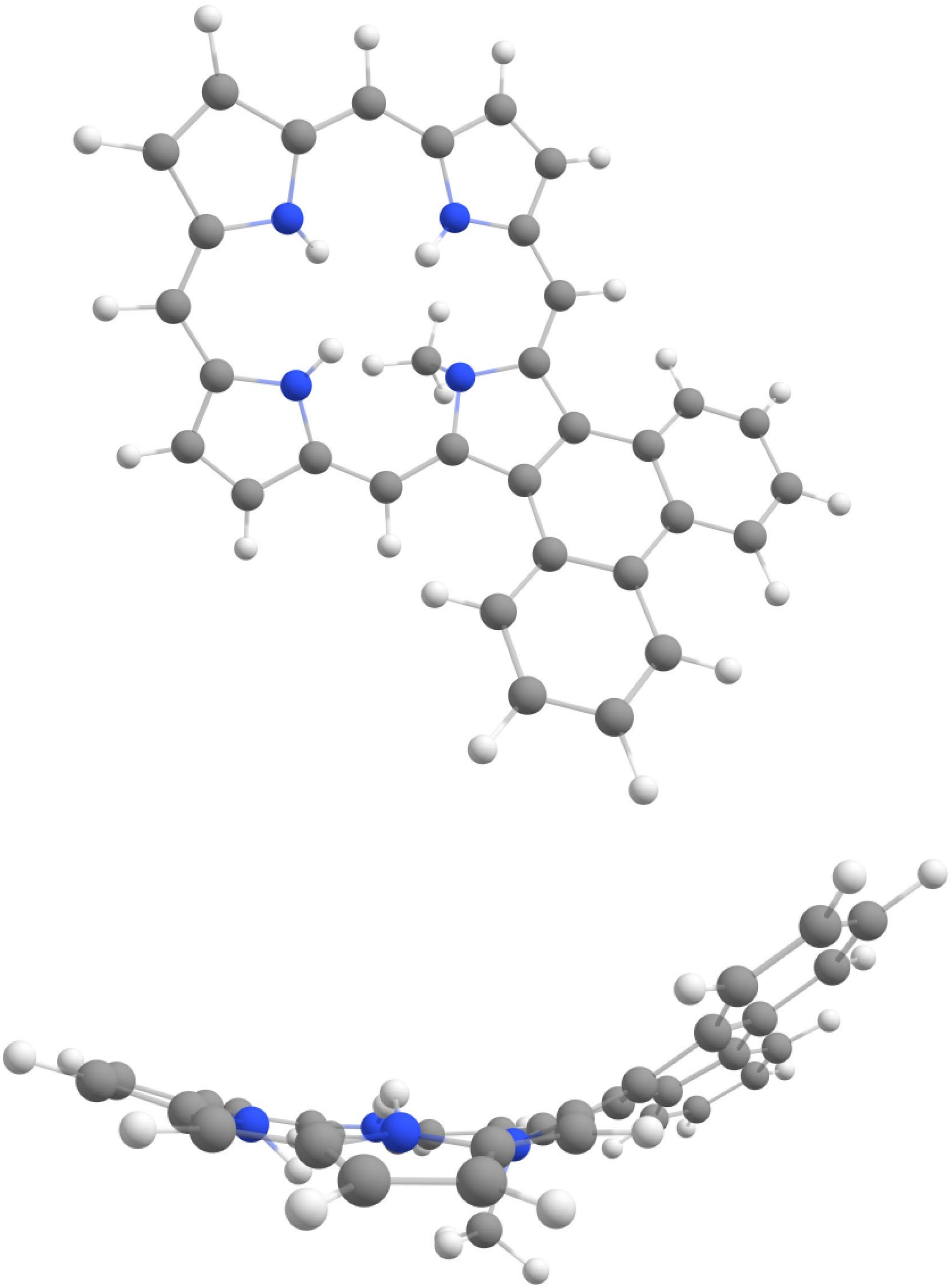

Figure S123. DFT calculated conformation (2 views) for *N*-methylphenanthroporphyrin dication **MePhP**H_2^{2+}.

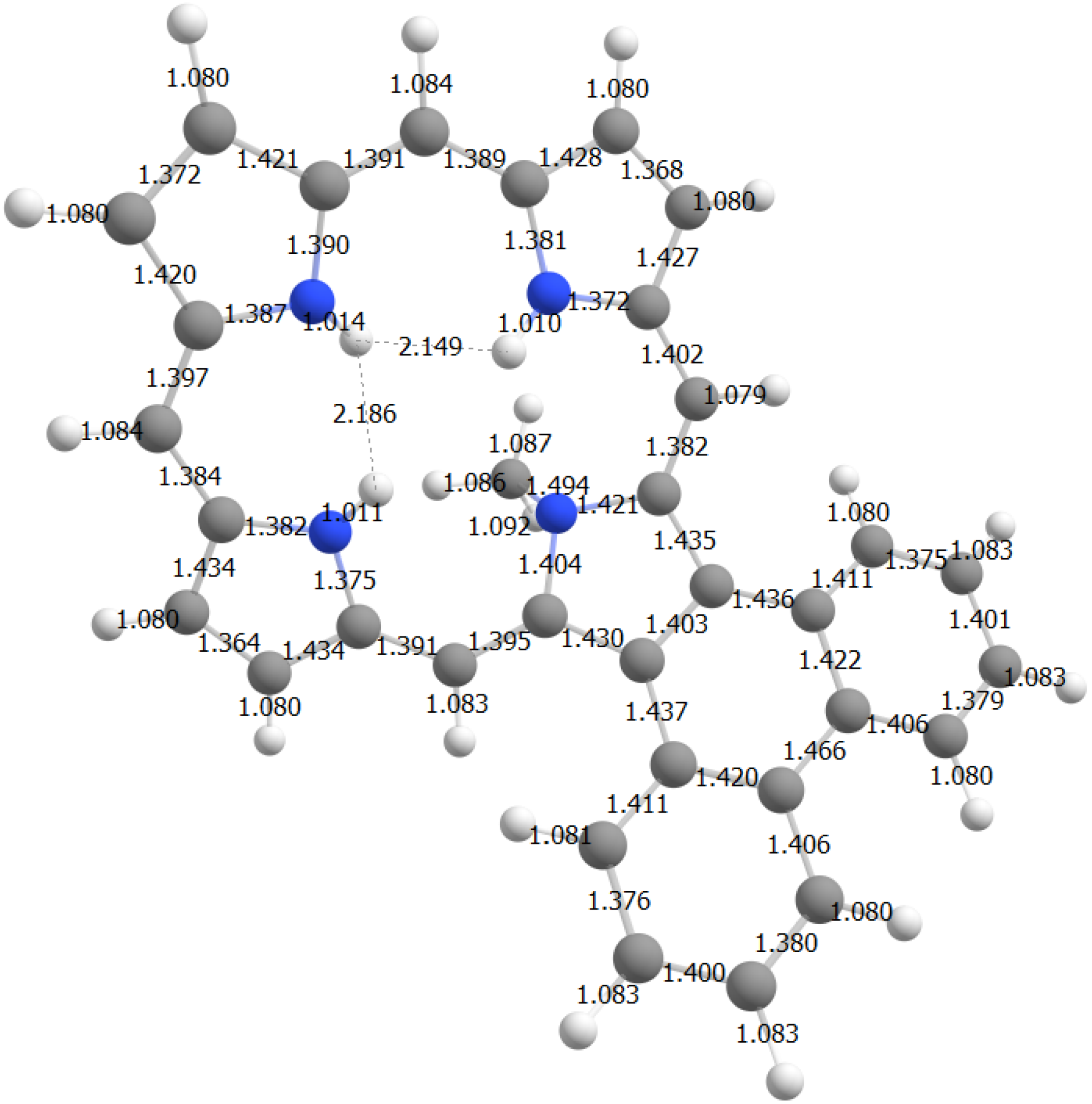

Figure S124. DFT calculated bond lengths for *N*-methylphenanthroporphyrin dication **MePhP**H_2^{2+}.

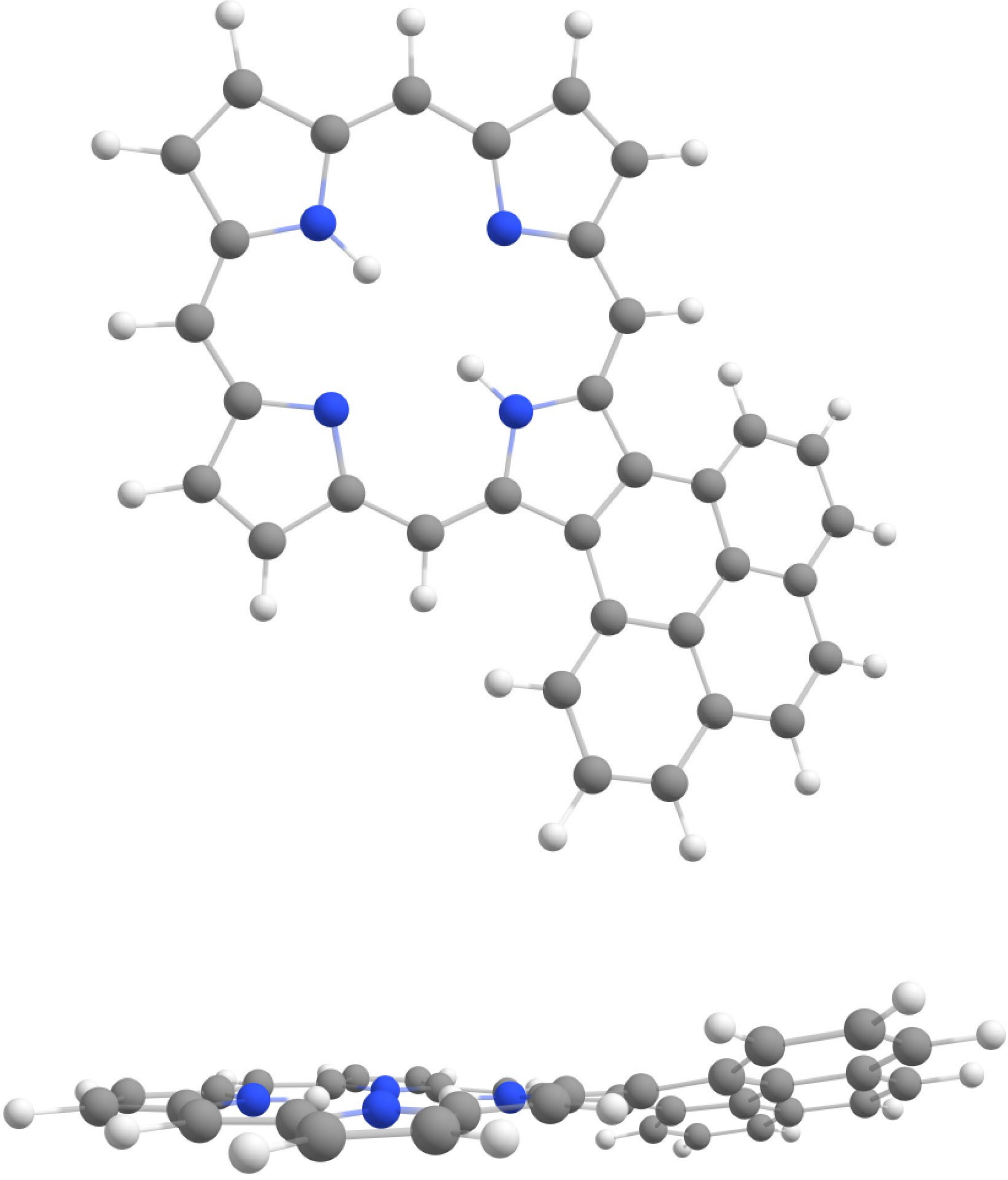

Figure S125. DFT calculated conformation (2 views) for pyrenoporphyrin tautomer **PyPa**.

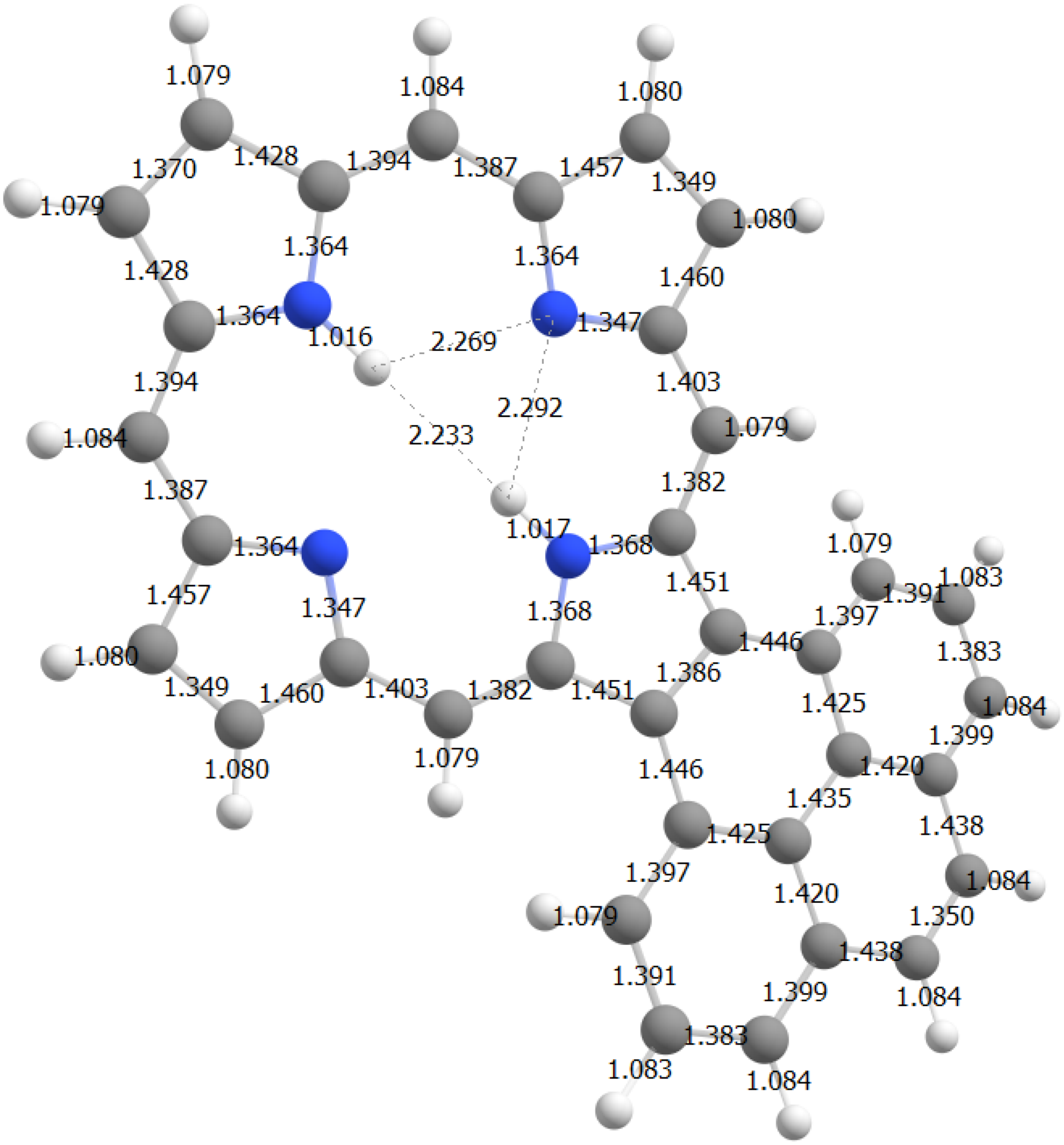

Figure S126. DFT calculated conformation (2 views) for pyrenoporphyrin tautomer **PyPa**.

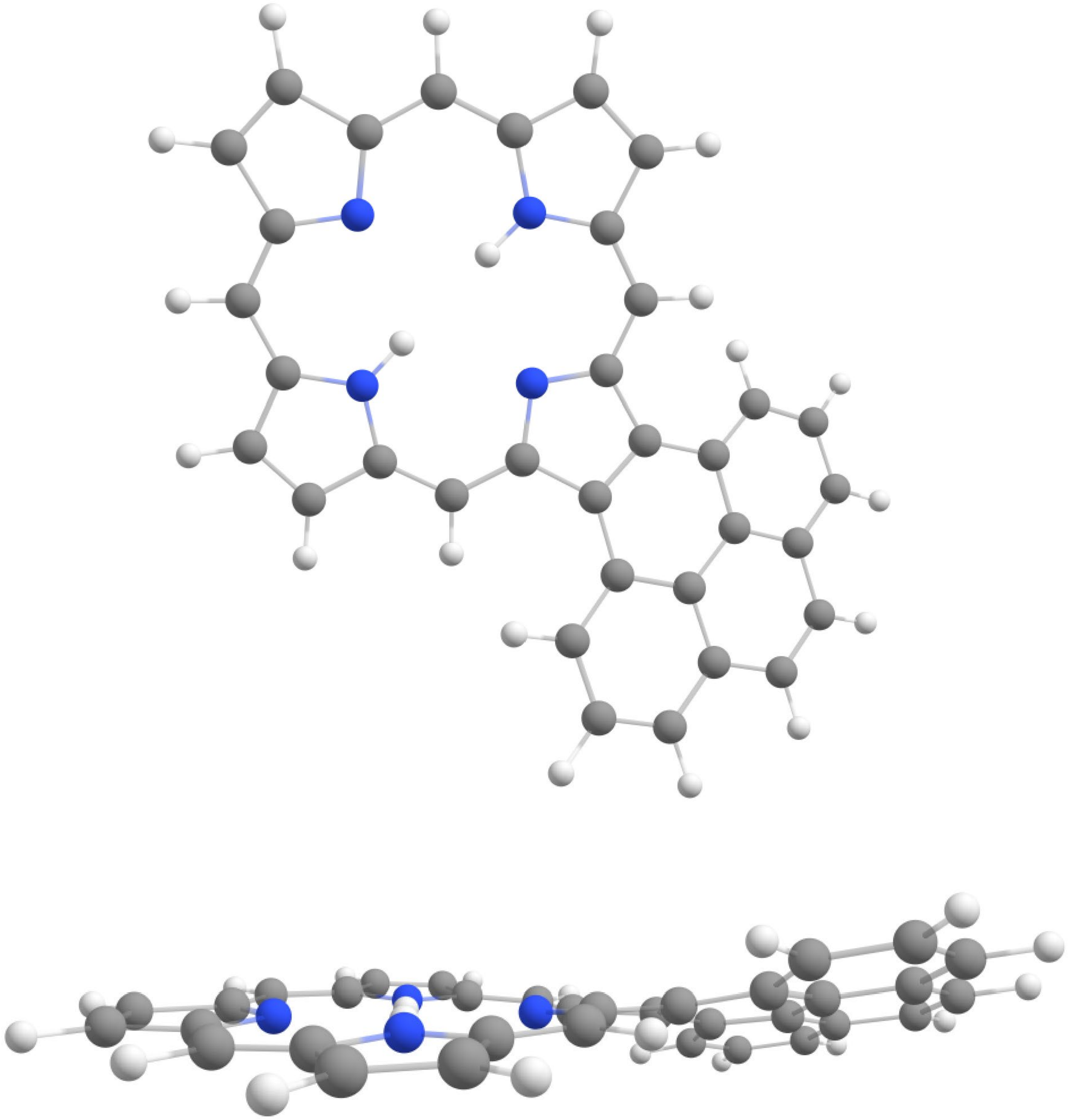

Figure S127. DFT calculated conformation (2 views) for pyrenoporphyrin tautomer **PyPb**.

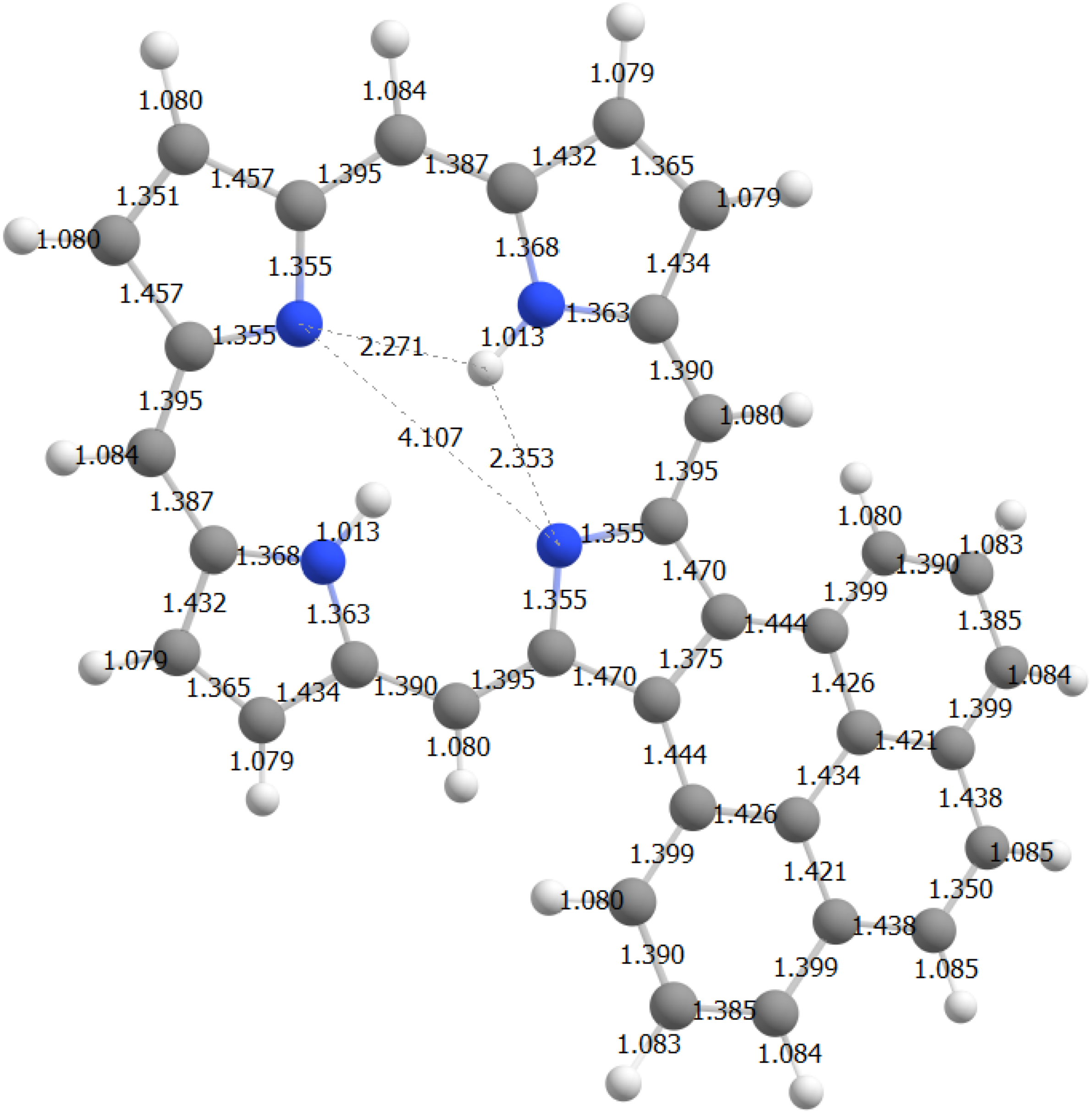

Figure S128. DFT calculated bond lengths for pyrenoporphyrin tautomer **PyPb**.

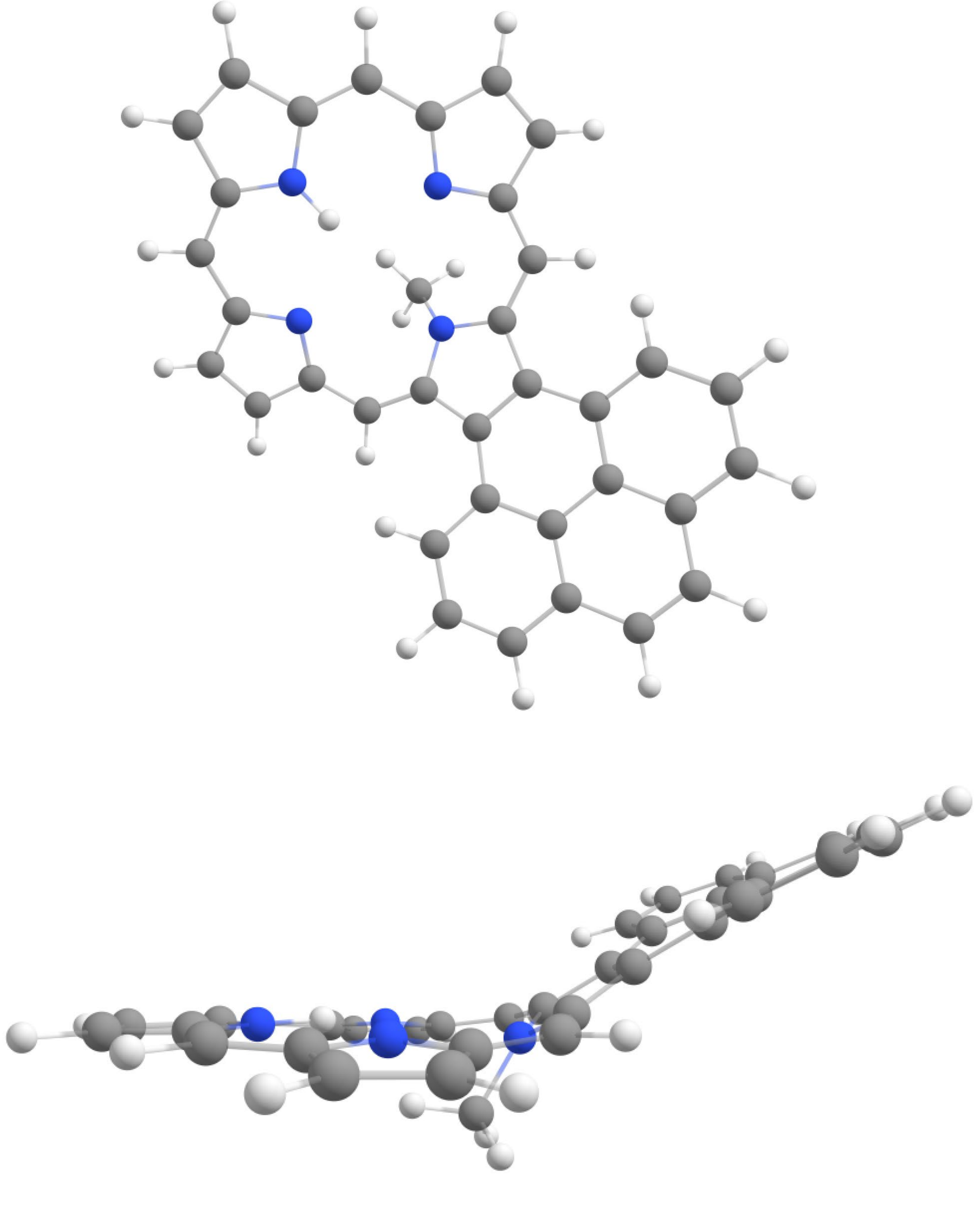

Figure S129. DFT calculated conformation (2 views) for *N*-methylpyrenoporphyrin tautomer **MePyPa**.

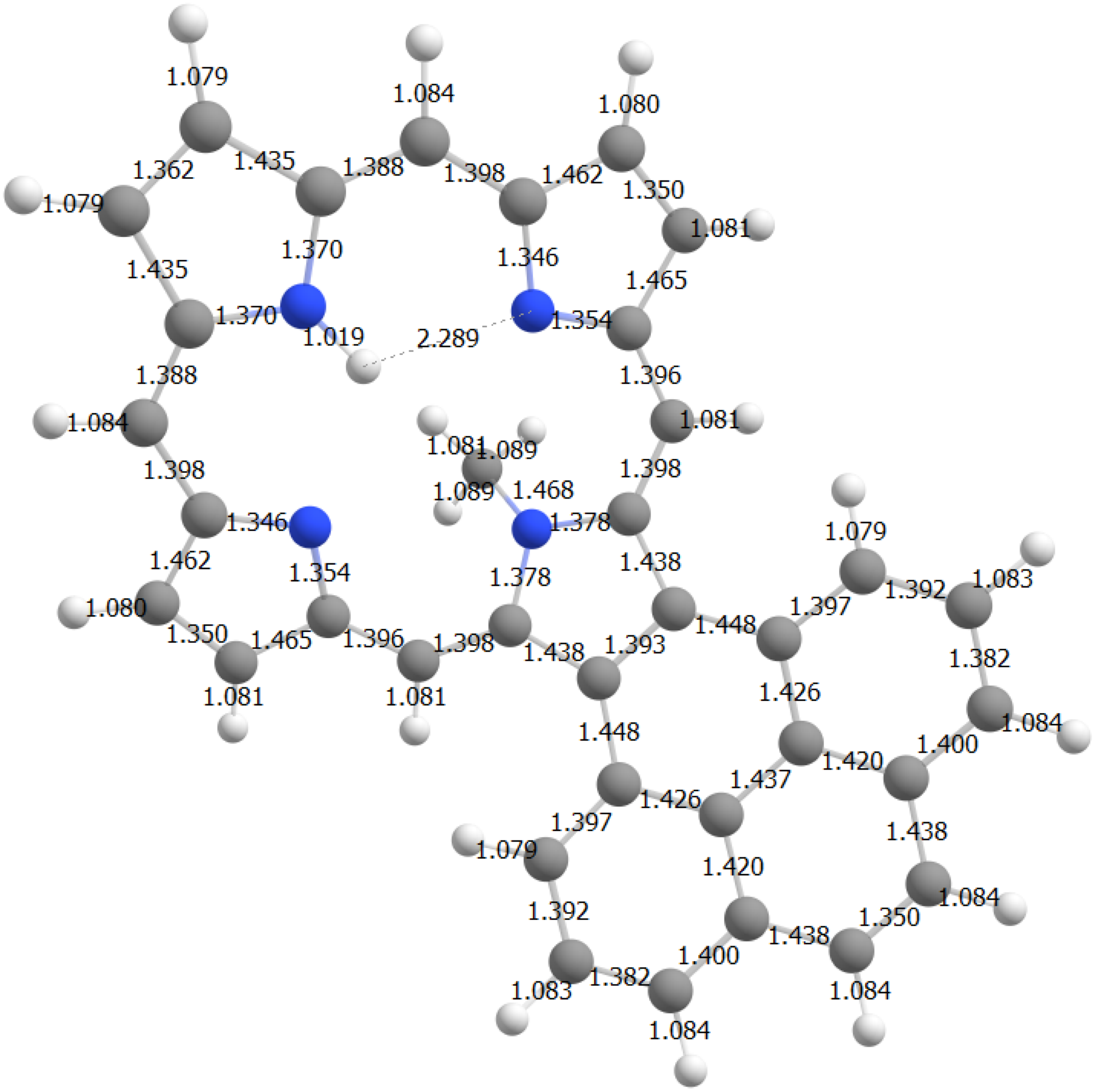

Figure S130. DFT calculated bond lengths for *N*-methylpyrenoporphyrin tautomer **MePyPa**.

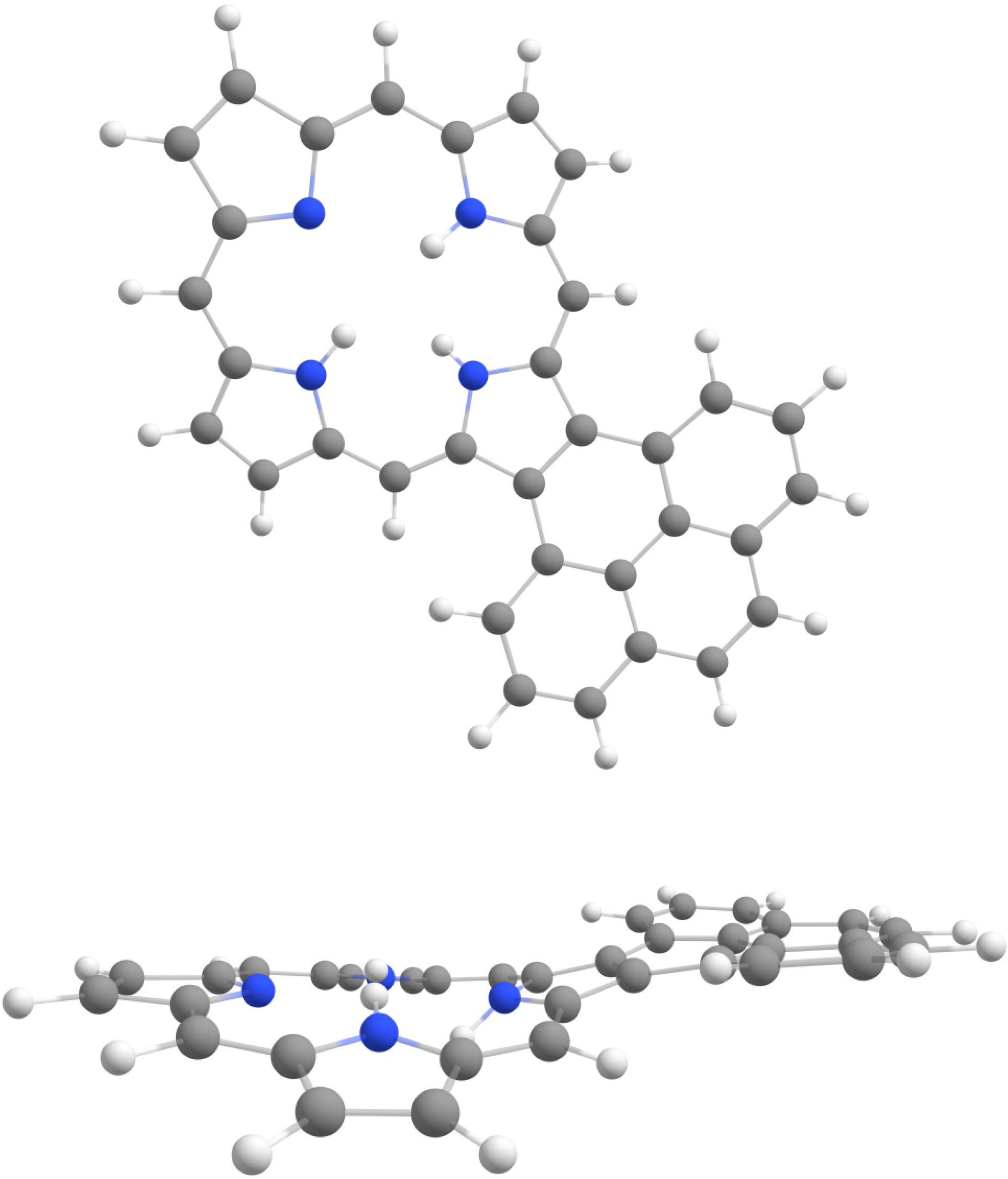

Figure S131. DFT calculated conformation (2 views) for pyrenoporphyrin cation **PyPb**H^{+}.

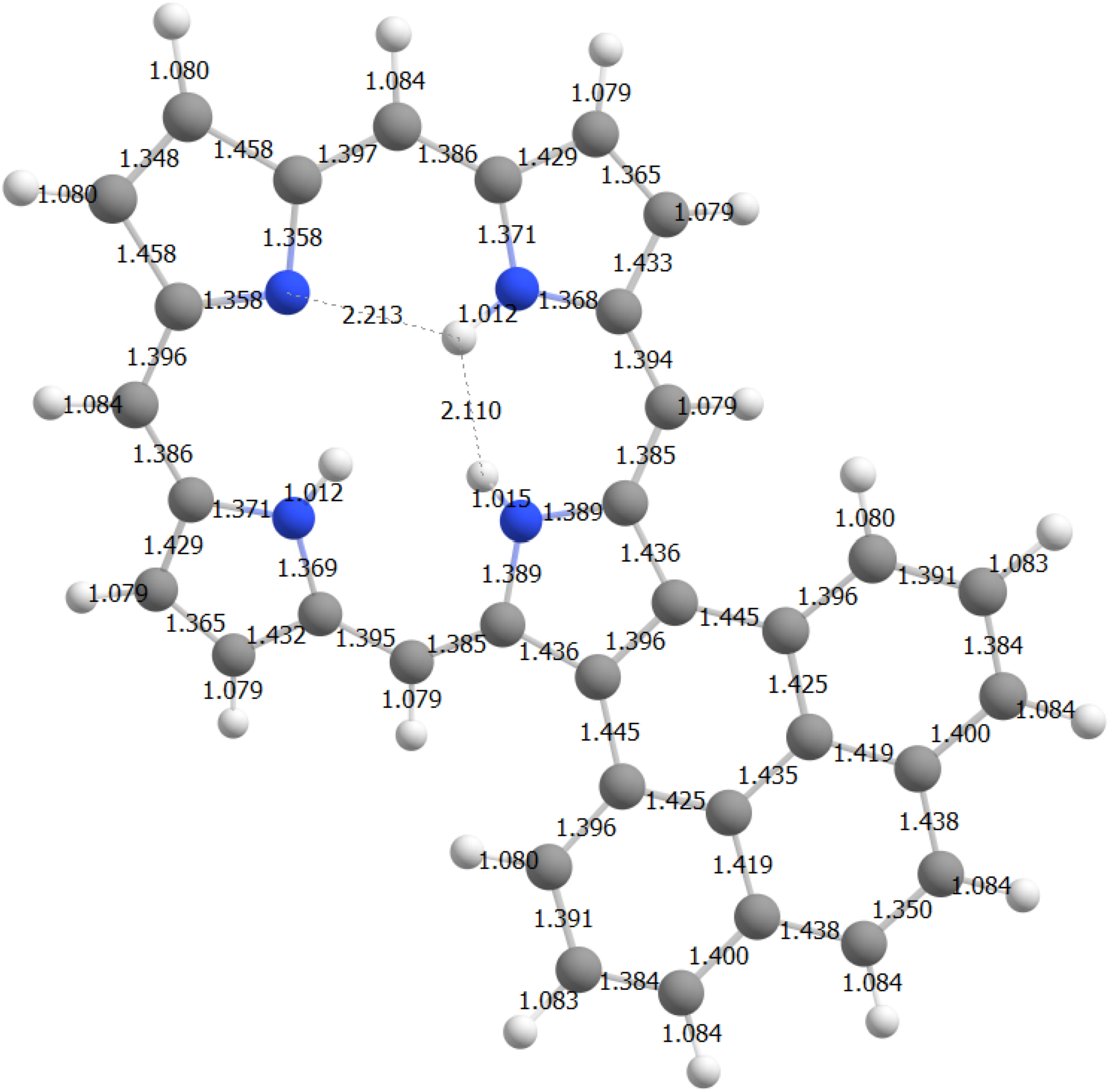

Figure S132. DFT calculated bond lengths for pyrenoporphyrin cation **PyPb**H^{+}.

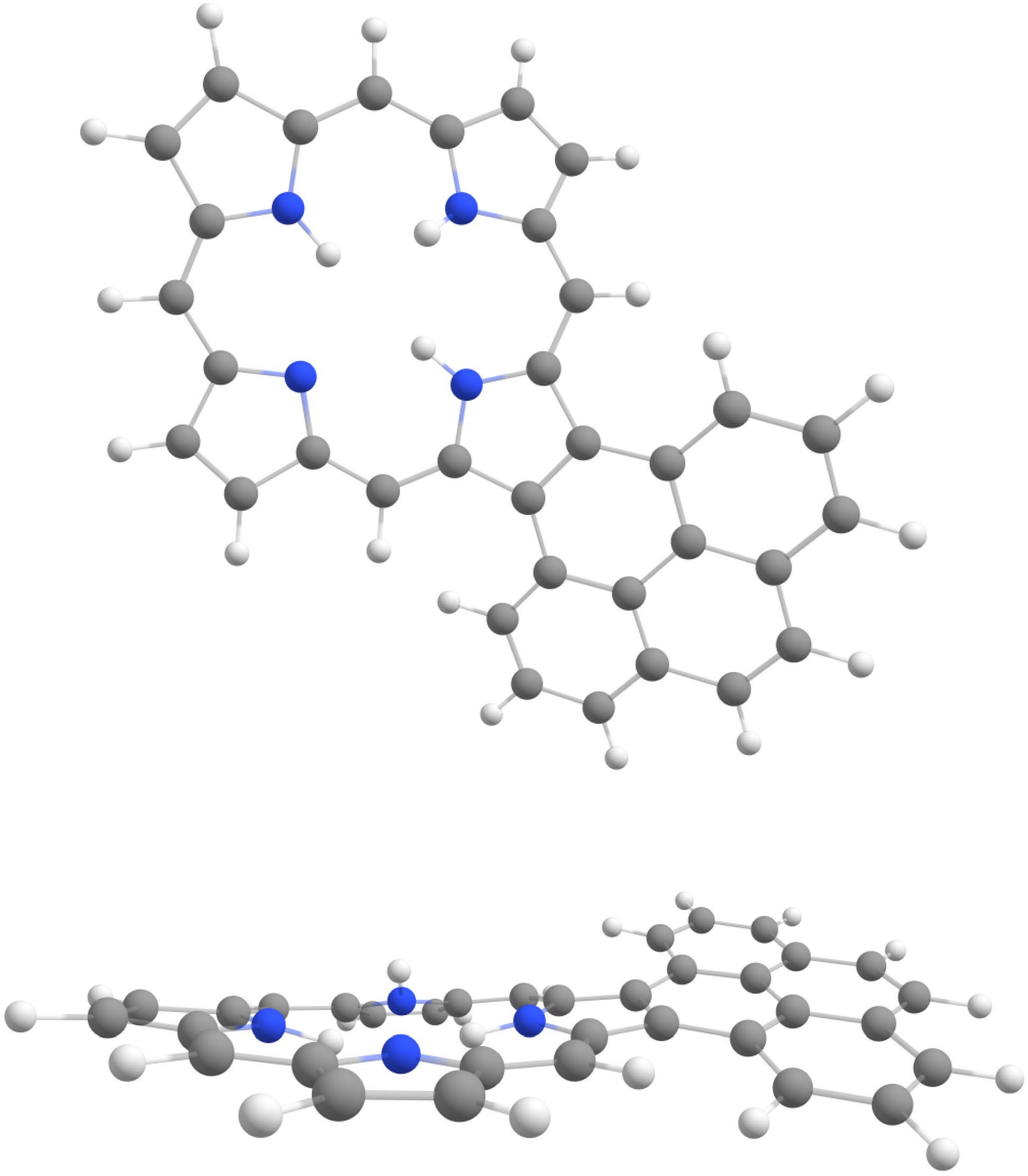

Figure S133. DFT calculated conformation (2 views) for pyrenoporphyrin cation **PyPc**H^{+}.

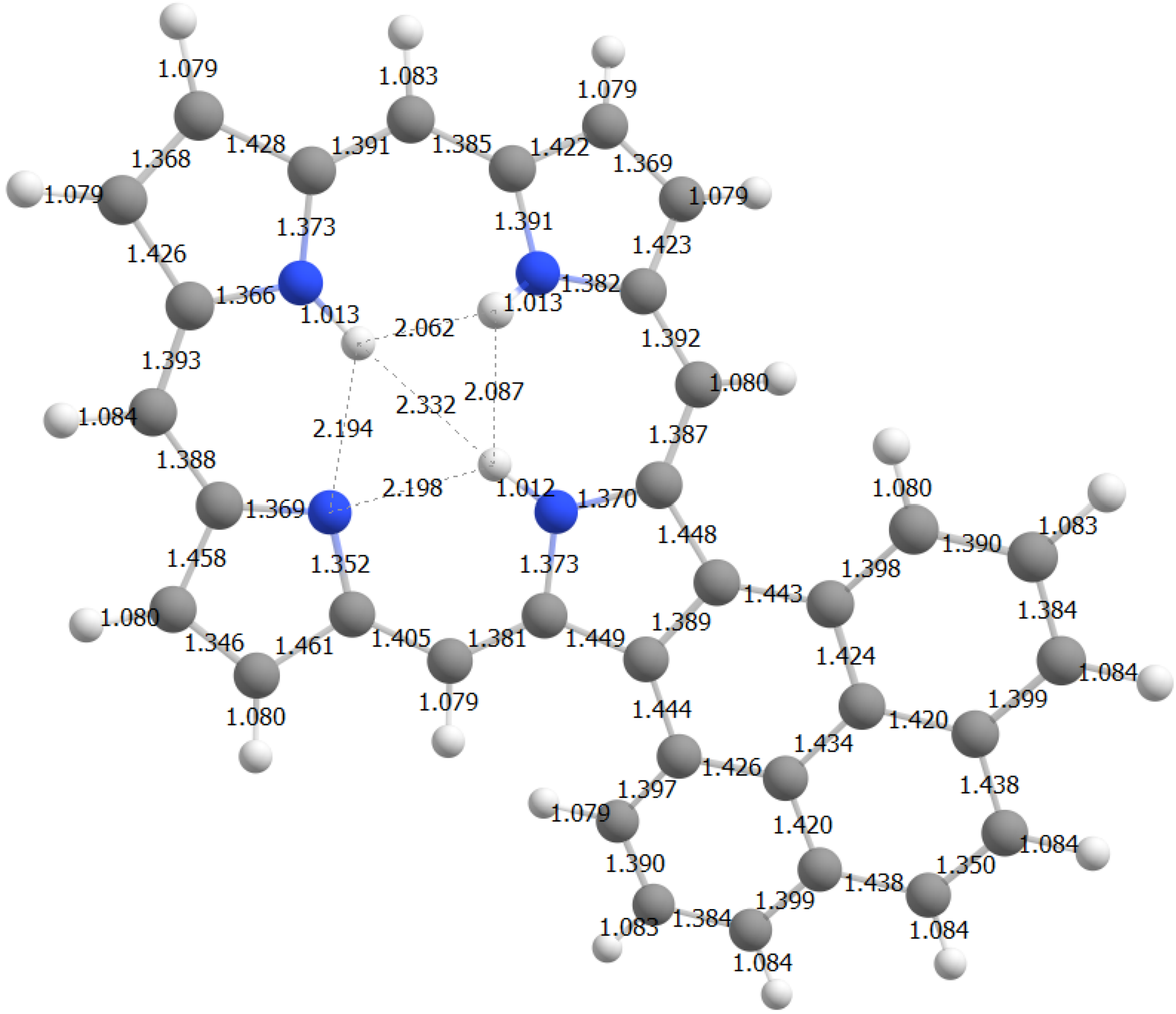

Figure S134. DFT calculated bond lengths for pyrenoporphyrin cation **PyPc**H$^+$.

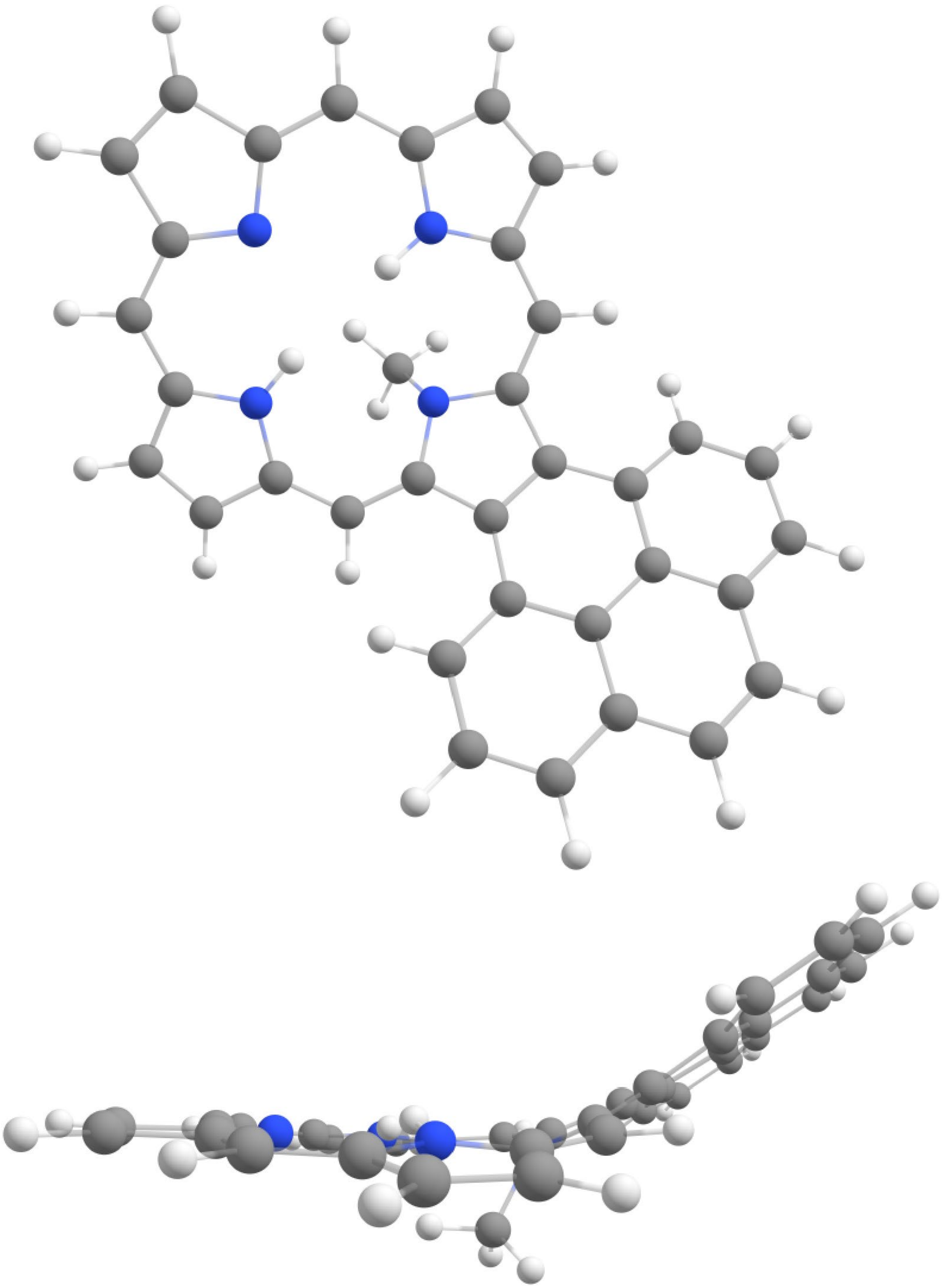

Figure S135. DFT calculated conformation (2 views) for *N*-methylpyrenoporphyrin cation **MePyPb**H^+.

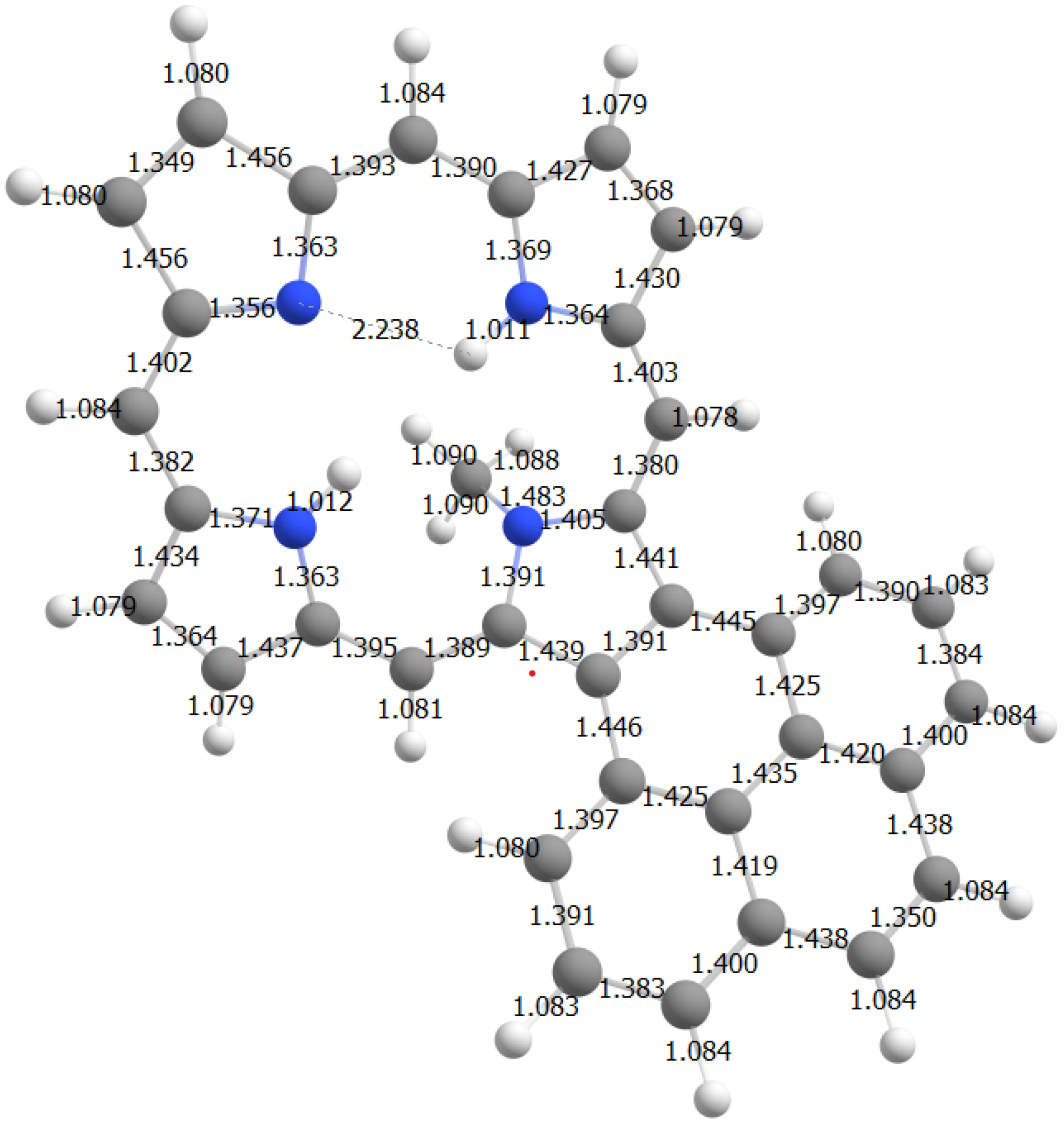

Figure S136. DFT calculated conformation (2 views) for *N*-methylpyrenoporphyrin cation **MePyPb**H^{+}.

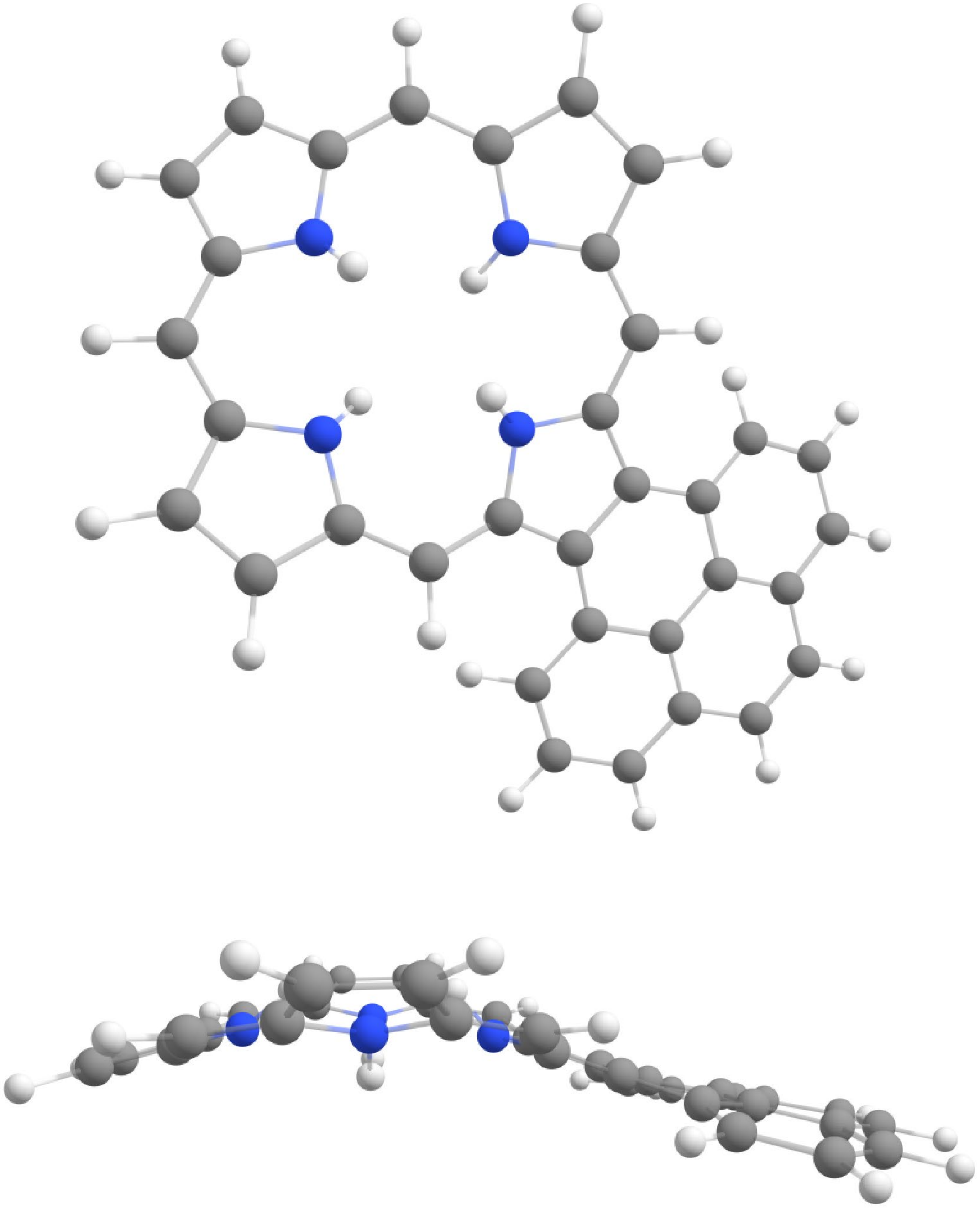

Figure S137. DFT calculated conformation (2 views) for pyrenoporphyrin dication **PyP**H_2^{2+}.

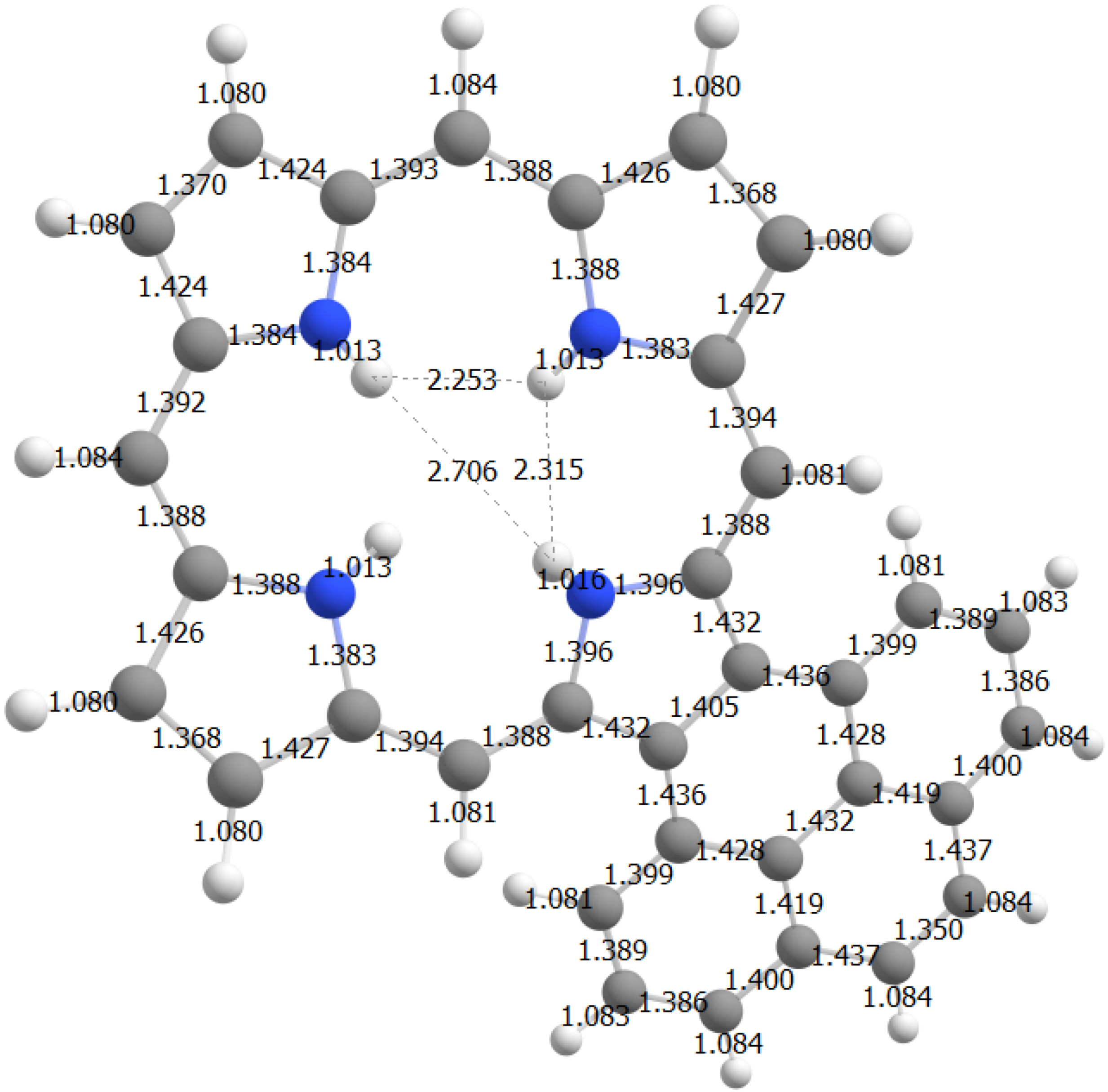

Figure S138. DFT calculated bond lengths for pyrenoporphyrin dication **PyP**H_2^{2+}.

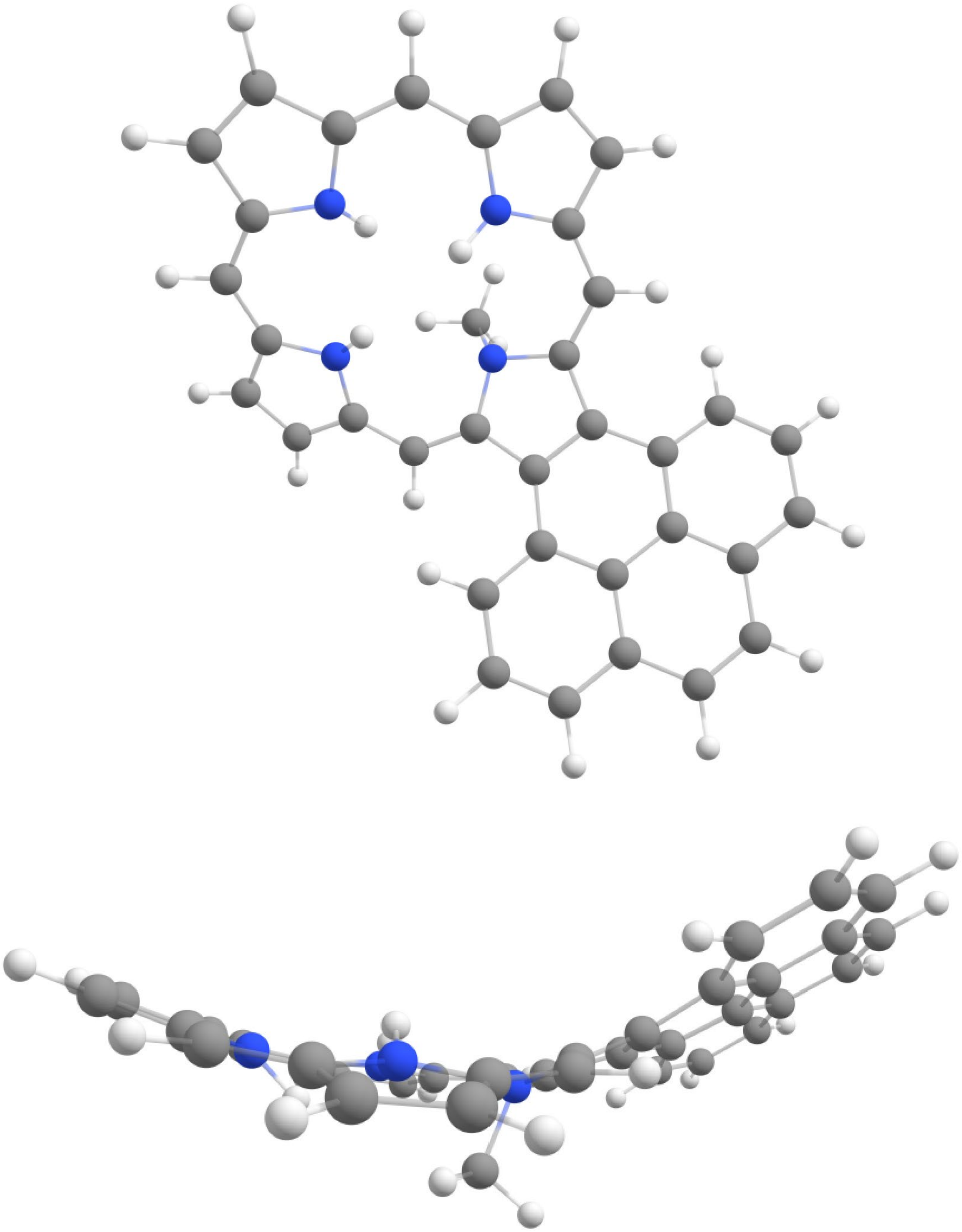

Figure S139. DFT calculated conformation (2 views) for *N*-methylpyrenoporphyrin dication **MePyP**H_2^{2+}.

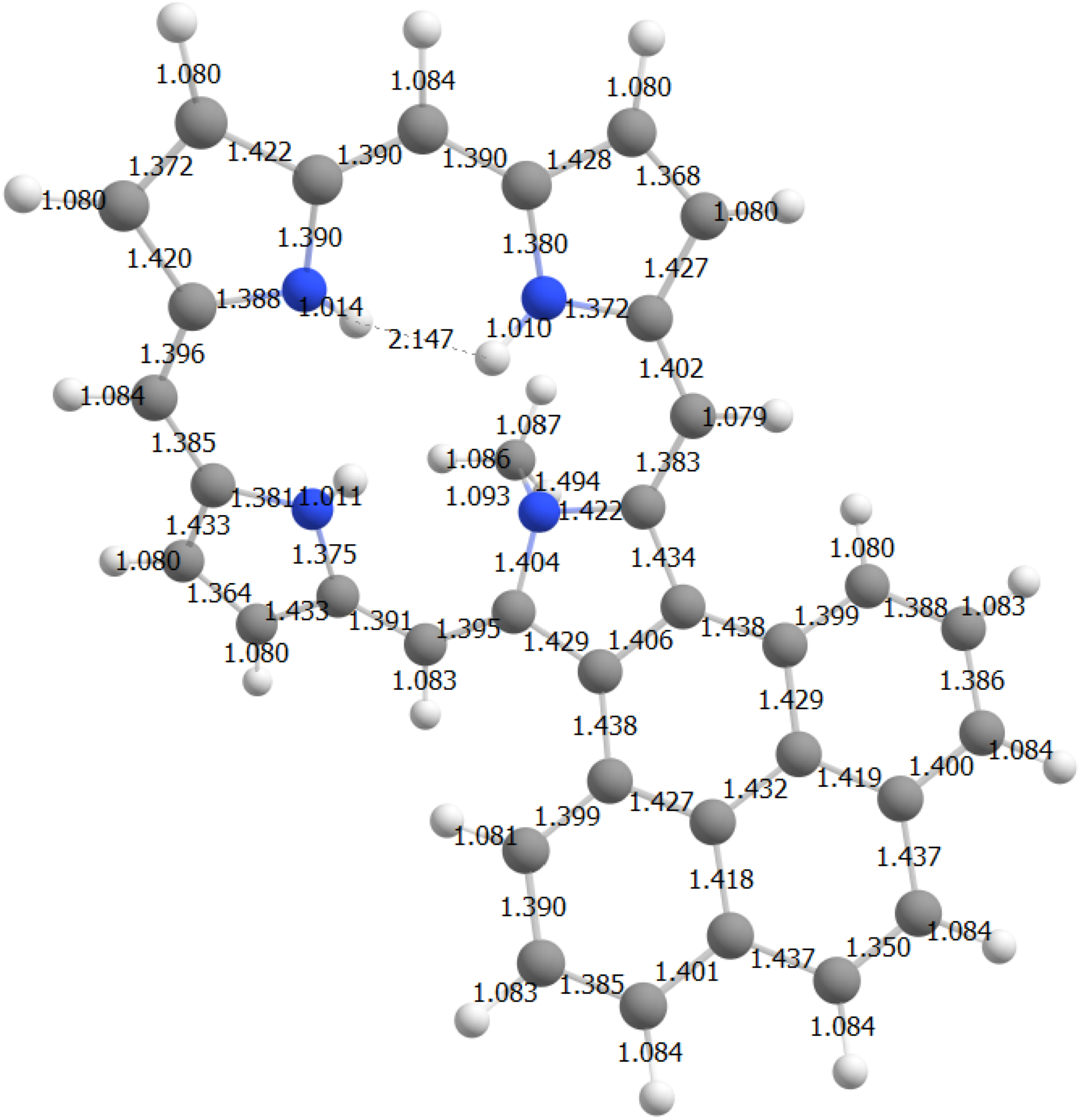

Figure S140. DFT calculated bond lengths for *N*-methylpyrenoporphyrin dication **MePyP**H_2^{2+}.

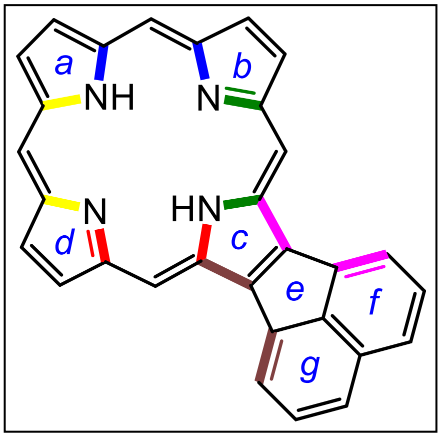

Table S1: AC Series (Dihedral Angle Analysis)

Molecule	*ab*	*bc*	*cd*	*da*	*cf*	*cg*	*Average*
**ANPa**	-0.01	0.00	-0.01	0.00	0.03	-0.05	0.02
**ANPb**	0.01	0.00	0.00	-0.01	-0.02	0.05	0.01
**ANPc**	0.00	0.01	-0.01	-0.02	-0.12	0.12	0.05
**ANPd**	-0.03	0.03	-0.05	0.02	0.13	-0.14	0.07
**MeANPa**	-0.15	-20.83	20.83	0.15	10.31	-10.32	10.43
**MeANPb**	1.47	-18.90	20.74	-2.34	10.16	-9.60	10.54
**ANPa**H^+	11.28	-3.89	3.89	-11.27	-1.84	1.84	5.67
**ANPb**H^+	-3.67	11.00	-10.99	3.64	-4.58	4.59	6.41
**ANPc**H^+	-10.93	11.69	-4.02	3.35	-1.13	1.16	5.38
**MeANPa**H^+	-0.67	17.90	-19.13	4.06	-9.23	10.55	10.26
**MeANPb**H^+	-3.76	30.77	-20.77	3.76	-8.85	8.84	12.79
**ANP**H_2^{2+}	16.54	-16.61	16.61	-16.54	2.30	-2.30	11.81
**MeANP**H_2^{2+}	-14.10	21.73	-21.73	14.10	-7.02	7.02	14.28

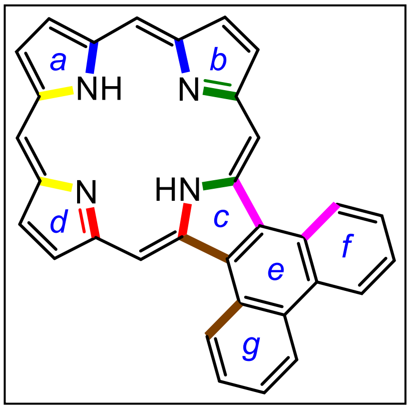

Table S2: PH Series (Dihedral Angle Analysis)

Molecule	*ab*	*bc*	*cd*	*da*	*ce*	*cg*	*Average*
**PhPa**	1.34	-5.56	-5.57	1.35	-10.83	-10.86	5.92
**PhPb**	1.37	-5.91	-5.89	1.35	-11.13	-11.18	6.14
**PhPc**	-1.38	8.05	3.58	-0.97	11.46	9.55	5.83
**PhPd**	1.27	-4.01	-7.28	1.47	-9.00	-12.74	5.96
**MePhPa**	-3.06	27.95	-27.95	3.06	9.47	-9.47	13.49
**MePhPb**	-0.34	14.94	-28.10	3.90	-14.83	-7.34	11.57
**PhPaH^{+}**	-10.30	-2.18	-10.57	12.98	-12.13	-12.17	10.05
**PhPbH^{+}**	-7.06	17.40	-17.38	7.07	13.02	-12.99	12.49
**PhPcH^{+}**	-14.02	17.17	-8.84	6.53	14.60	-13.45	12.43
**MePhPaH^{+}**	4.31	-28.59	29.40	-9.02	-11.51	7.79	15.10
**MePhPbH^{+}**	-2.67	16.51	-29.21	6.08	-16.03	-7.79	13.05
**PhPH$_2^{2+}$**	19.19	-21.98	21.99	-19.19	-12.94	12.94	18.04
**MePhPH$_2^{2+}$**	-13.07	17.33	-30.43	16.54	-16.42	-9.03	17.14

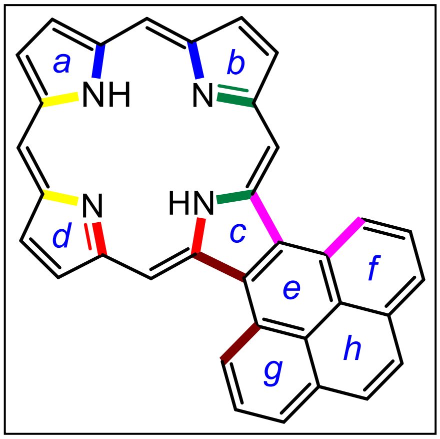

Table S3: PY Series (Dihedral Angle Analysis)							
**Molecule**	***ab***	***bc***	***cd***	***da***	***ce***	***cg***	***Average***
**PyPa**	1.30	-5.30	-5.30	1.30	-9.92	-9.92	5.51
**PyPb**	1.35	-5.66	-5.65	1.33	-10.33	-10.35	5.78
**PyPc**	-1.38	7.76	3.35	-0.92	10.61	8.74	5.46
**PyPd**	1.18	-3.74	-7.01	1.48	-8.18	-11.74	5.55
**MePyPa**	-3.01	27.96	-27.95	3.01	7.57	-7.56	12.84
**MePyPb**	-0.60	15.58	-28.05	3.92	-13.74	-6.43	11.39
**PyPaH**$^{+}$	-10.32	-1.88	-10.59	13.06	-11.10	-11.56	9.75
**PyPbH**$^{+}$	-7.00	17.40	-17.39	7.02	11.20	-11.17	11.86
**PyPcH**$^{+}$	-12.94	17.02	1.29	3.47	10.75	9.00	9.08
**MePyPaH**$^{+}$	4.26	-28.65	20.11	-8.88	-9.70	5.40	12.83
**MePyPbH**$^{+}$	-2.85	17.02	-29.14	6.17	-14.78	-7.05	12.84
**PyPH**$_2^{2+}$	19.13	-22.00	22.02	-19.13	-11.50	11.49	17.54
**MePyPH**$_2^{2+}$	-13.09	17.54	-30.23	16.51	-15.15	-8.63	16.86

Table S4: AC Series (Relative Energy Analysis)				
Molecule	E (Hartrees)	G (Hartrees)	Delta E (kcal/mol)	Delta G (kcal/mol)
	M06-2X cc-PVTZ	M06-2X 6-311++G(d,p)	M06-2X cc-PVTZ	M06-2X 6-311++G(d,p)
**ANPa**	-1372.952617	-1372.466099	0.00	0.00
**ANPb**	-1372.951104	-1372.464705	0.95	0.87
**ANPc**	-1372.937019	-1372.450697	9.79	9.66
**ANPd**	-1372.938276	-1372.451989	9.00	8.85
**MeANPa**	-1412.228303	-1411.710731	0.00	0.00
**MeANPb**	-1412.219030	-1411.701424	5.82	5.84
**ANPa**H^+	-1373.344272	-1372.841506	3.11	3.05
**ANPb**H^+	-1373.349231	-1372.846362	0.00	0.00
**ANPc**H^+	-1373.348043	-1372.845092	0.75	0.80
**MeANPa**H^+	-1412.637262	-1412.104728	5.06	4.67
**MeANPb**H^+	-1412.645327	-1412.112173	0.00	0.00
**ANP**H_2^{2+}	-1373.626423	-1373.108917	---	---
**MeANP**H_2^{2+}	-1412.922490	-1412.375629	---	---

Table S5: PH Series (Relative Energy Analysis)				
Molecule	E (Hartrees)	G (Hartrees)	Delta E (kcal/mol)	Delta G (kcal/mol)
	M06-2X cc-PVTZ	M06-2X 6-311++G(d,p)	M06-2X cc-PVTZ	M06-2X 6-311++G(d,p)
**PhPa**	-1450.386886	-1449.858747	0.70	0.73
**PhPb**	-1450.387995	-1449.859915	0.00	0.00
**PhPc**	-1450.374450	-1449.846605	8.50	8.35
**PhPd**	-1450.372132	-1449.844217	9.95	9.85
**MePhPa**	-1489.661552	-1489.102901	0.00	0.00
**MePhPb**	-1489.652397	-1489.093226	5.75	6.07
**PhPa**H^+	-1450.780424	-1450.236358	1.33	1.20
**PhPb**H^+	-1450.782257	-1450.238264	0.18	0.00
**PhPc**H^+	-1450.782536	-1450.238241	0.00	0.01
**MePhPa**H^+	-1490.069884	-1489.495718	4.66	4.24
**MePhPb**H^+	-1490.077307	-1489.502470	0.00	0.00
**PhP**H_2^{2+}	-1451.061200	-1450.502768	---	---
**MePhP**H_2^{2+}	-1490.353260	-1489.765876	---	---

Table S6: PY Series (Relative Energy Analysis)				
**Molecule**	**E (Hartrees)**	**G (Hartrees)**	**Delta E (kcal/mol)**	**Delta G (kcal/mol)**
	M06-2X cc-PVTZ	M06-2X 6-311++G(d,p)	M06-2X cc-PVTZ	M06-2X 6-311++G(d,p)
**PyPa**	-1526.618853	-1526.070655	0.66	0.74
**PyPb**	-1526.619910	-1526.071831	0.00	0.00
**PyPc**	-1526.606373	-1526.058478	8.49	8.38
**PyPd**	-1526.604101	-1526.056098	9.92	9.87
**MePyPa**	-1565.893768	-1565.315005	0.00	0.00
**MePyPb**	-1565.884390	-1565.305281	5.88	6.10
**PyPa**H^+	-1527.012257	-1526.448222	1.27	1.24
**PyPb**H^+	-1527.014233	-1526.450206	0.03	0.00
**PyPc**H^+	-1527.014282	-1526.450010	0.00	0.12
**MePyPa**H^+	-1566.301976	-1565.707885	4.62	4.15
**MePyPb**H^+	-1566.309342	-1565.714506	0.00	0.00
**PyP**H_2^{2+}	-1527.293786	-1526.715425	---	---
**MePyP**H_2^{2+}	-1566.586103	-1565.979171	---	---

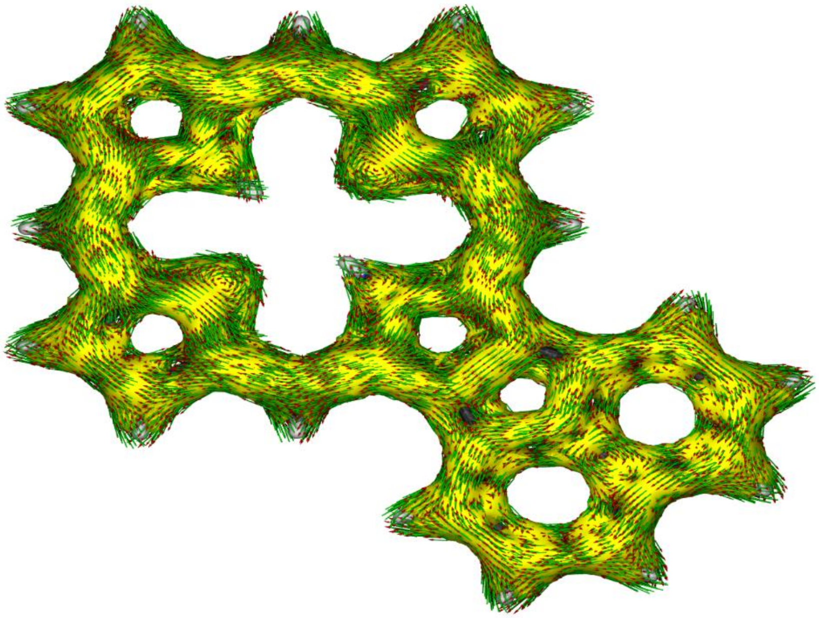

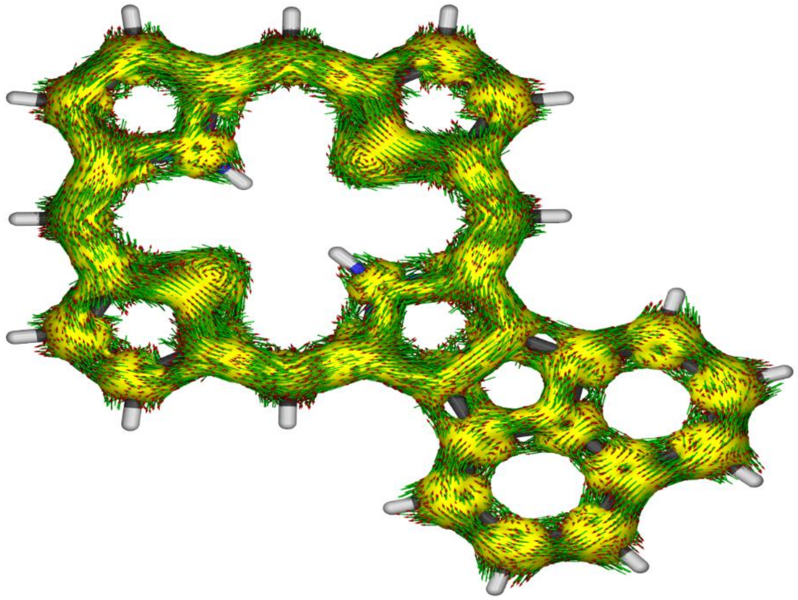

Figure S141. AICD plots for acenaphthoporphyrin tautomer **ANPa**.
Isovalues 0.05 (top) and 0.07 (bottom).

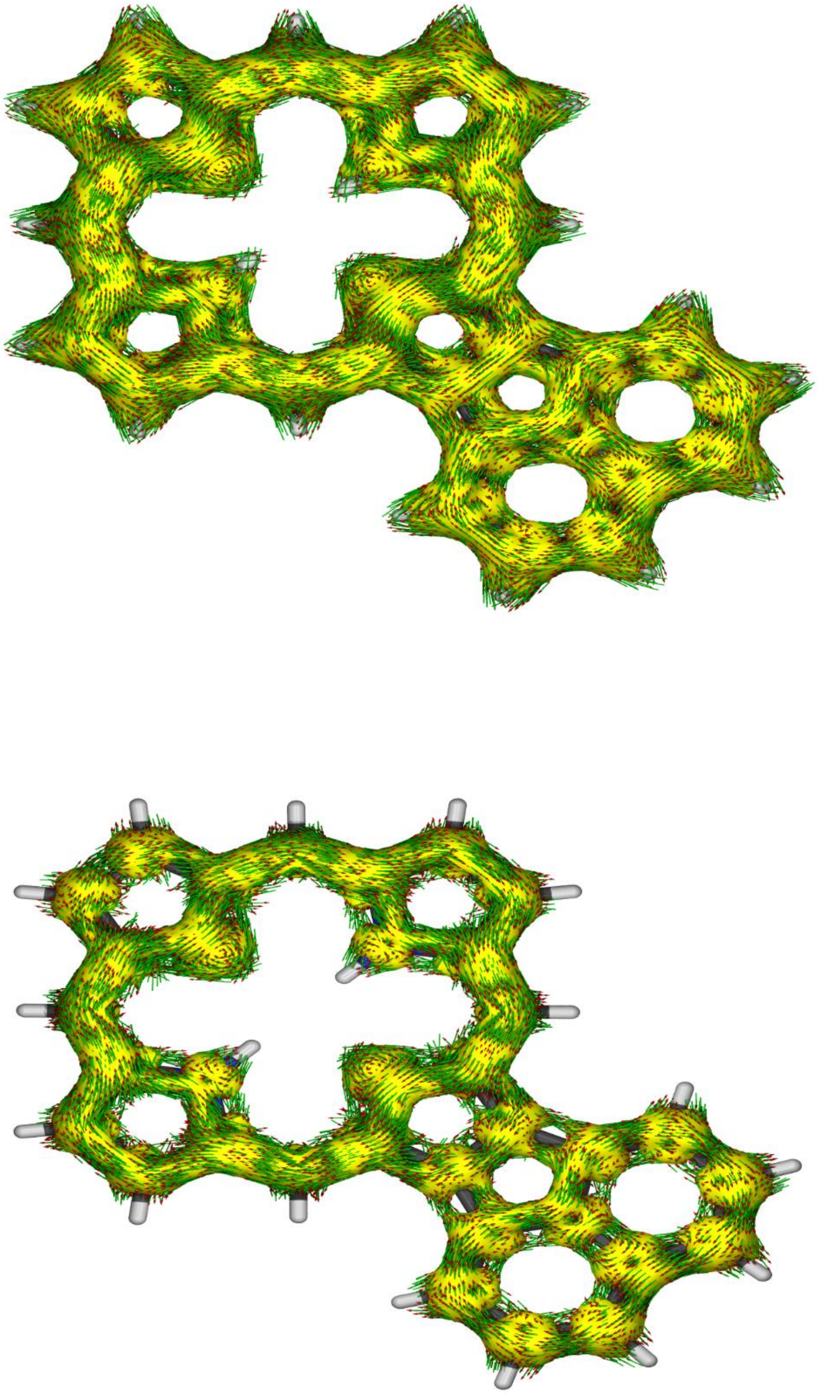

Figure S142. AICD plots for acenaphthoporphyrin tautomer **ANPb**.
Isovalues 0.05 (top) and 0.07 (bottom).

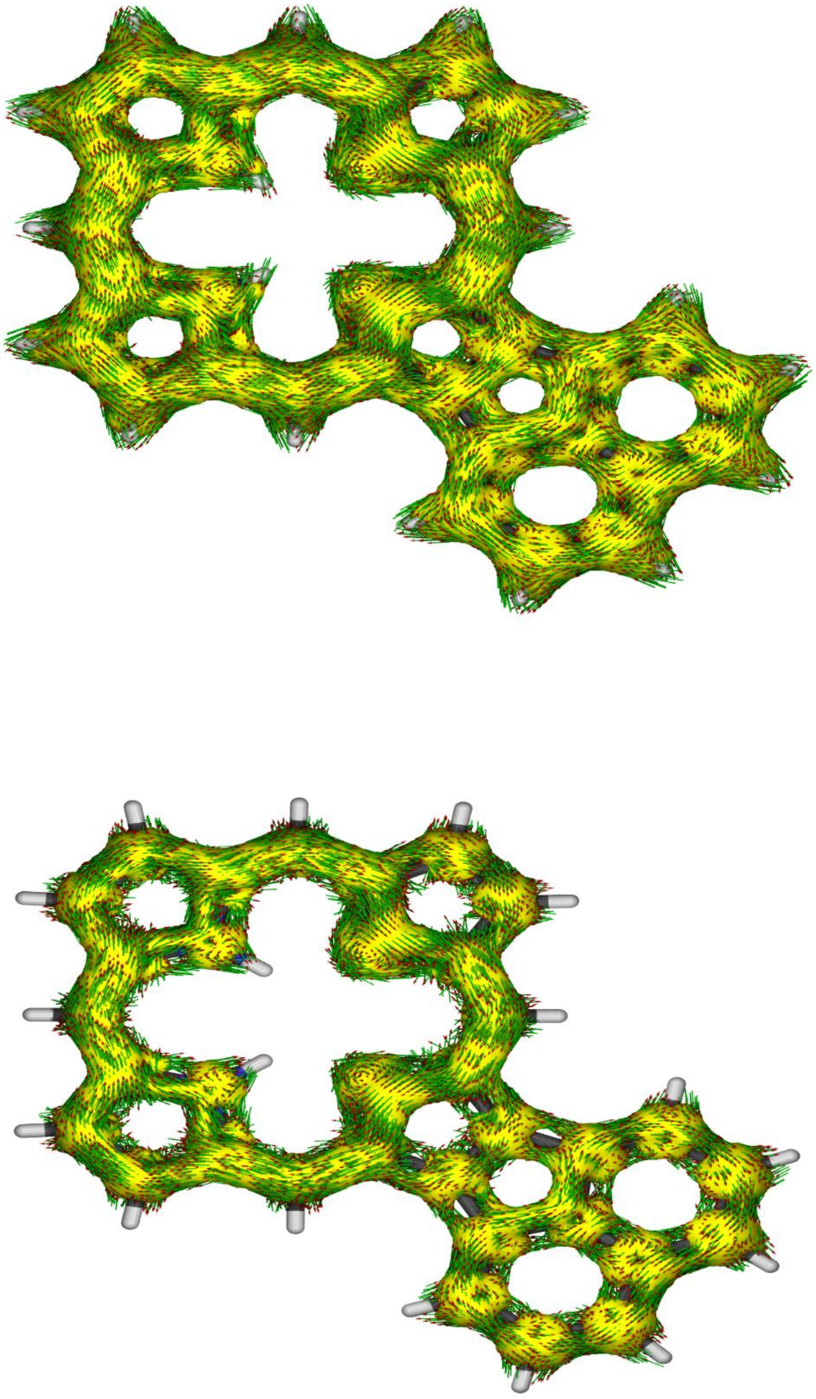

Figure S143. AICD plots for acenaphthoporphyrin tautomer **ANPc**.
Isovalues 0.05 (top) and 0.07 (bottom).

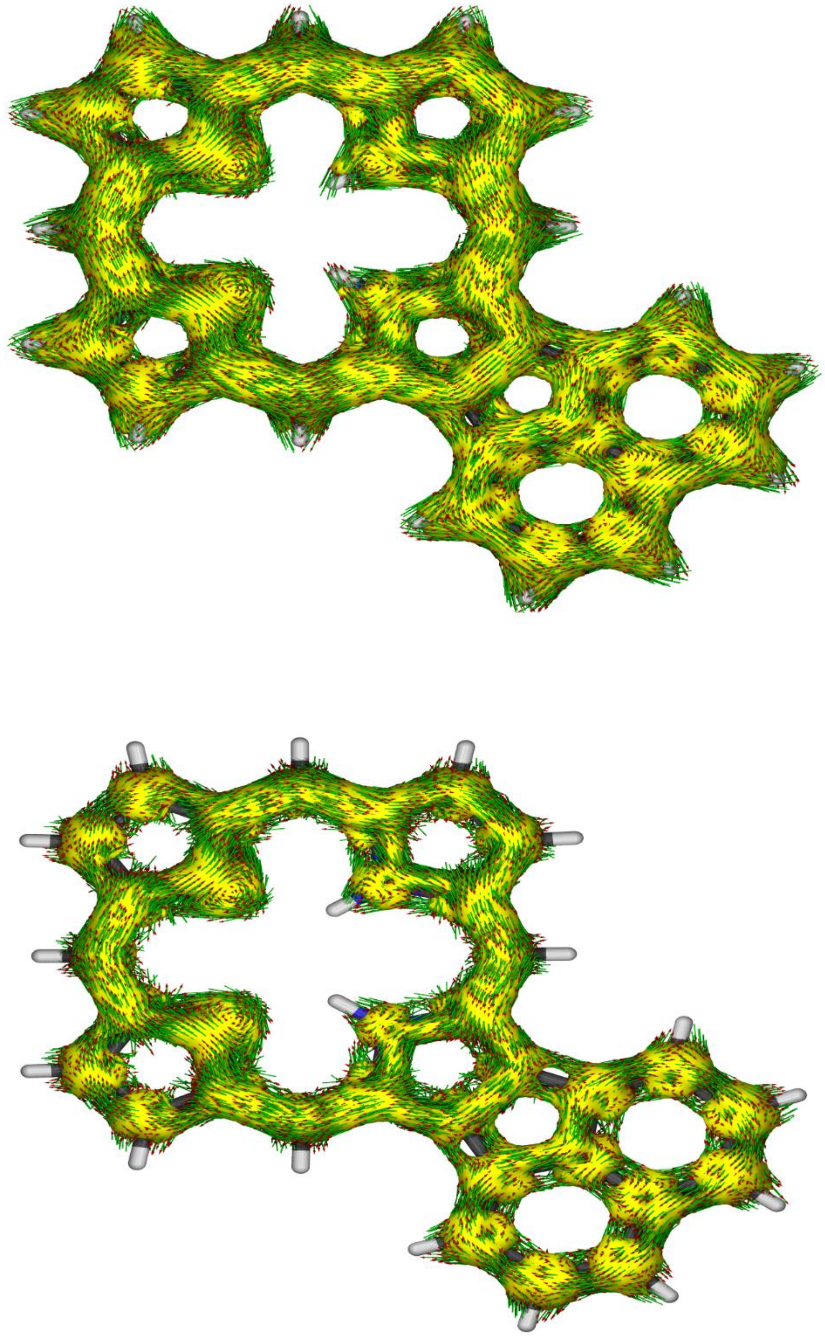

Figure S144. AICD plots for acenaphthoporphyrin tautomer **ANPd**.
Isovalues 0.05 (top) and 0.07 (bottom).

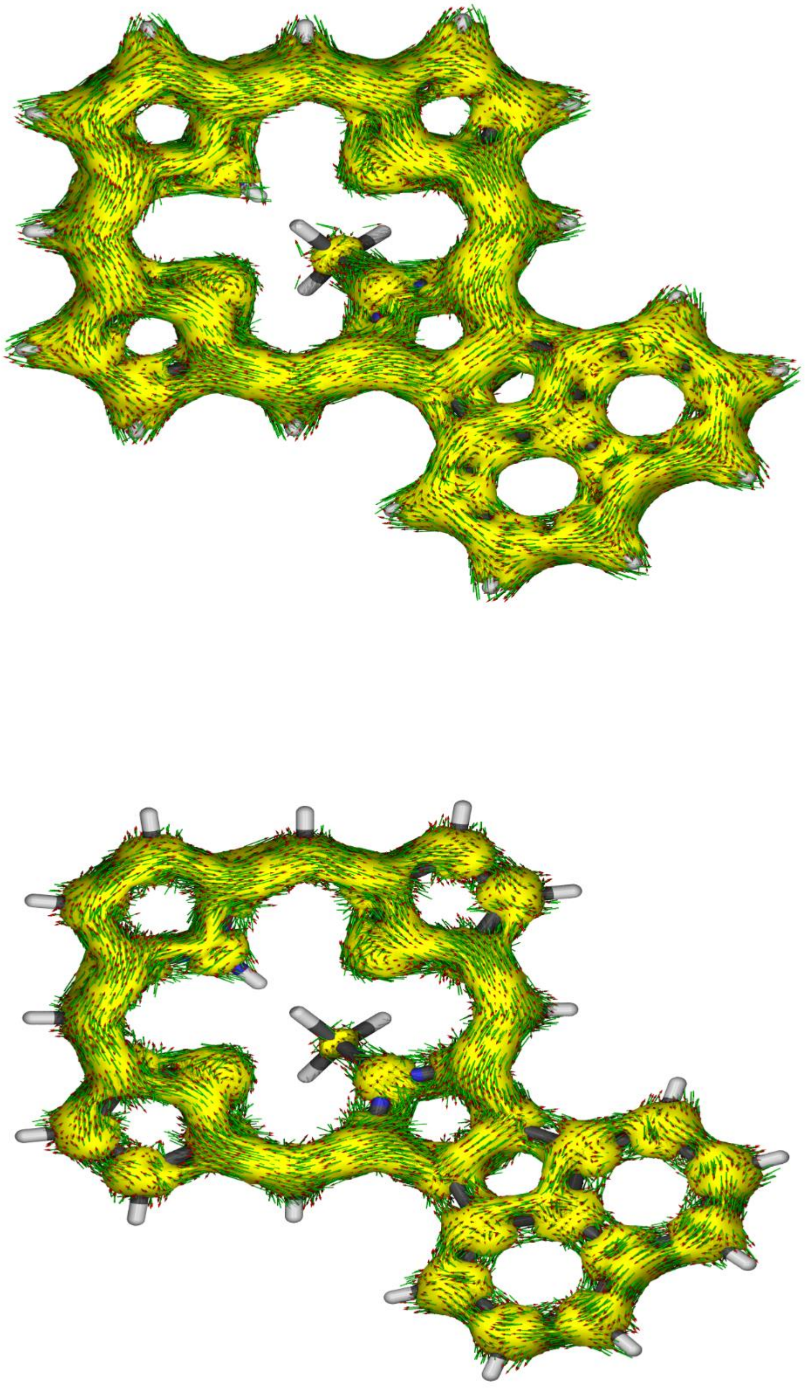

Figure S145. AICD plots for *N*-methylacenaphthoporphyrin tautomer **MeANPa**. Isovalues 0.05 (top) and 0.07 (bottom).

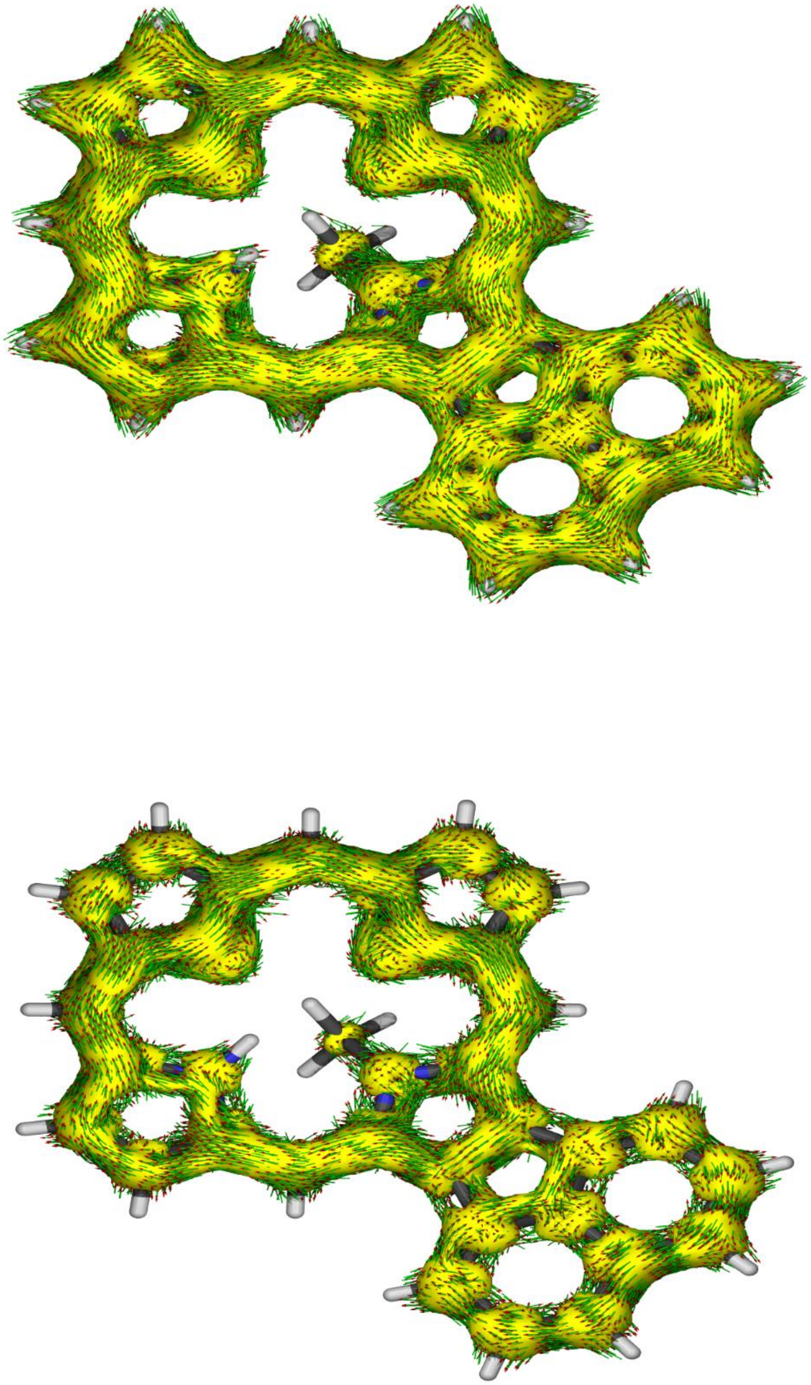

Figure S146. AICD plots for *N*-methylacenaphthoporphyrin tautomer **MeANPb**. Isovalues 0.05 (top) and 0.07 (bottom).

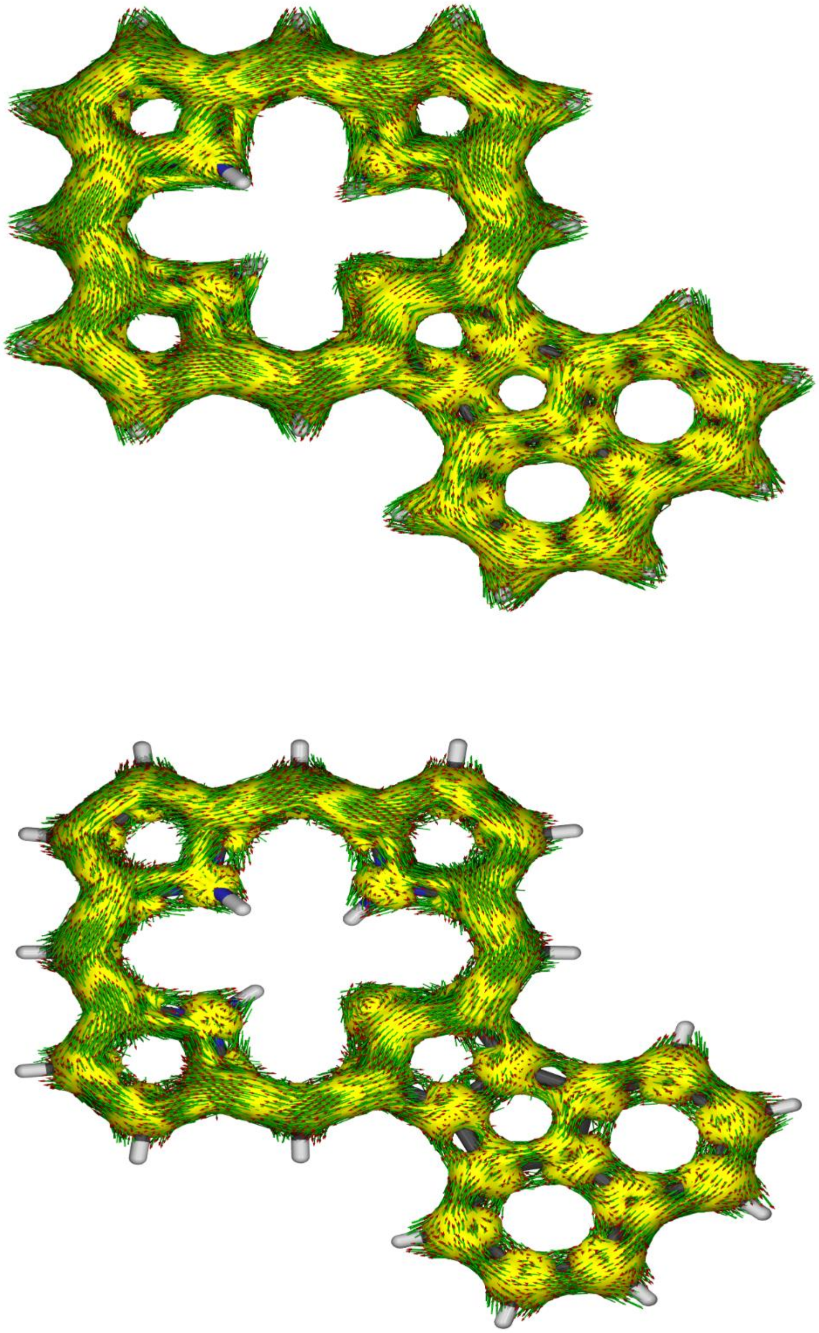

Figure S147. AICD plots for acenaphthoporphyrin cation **ANPa**H^+.
Isovalues 0.05 (top) and 0.07 (bottom).

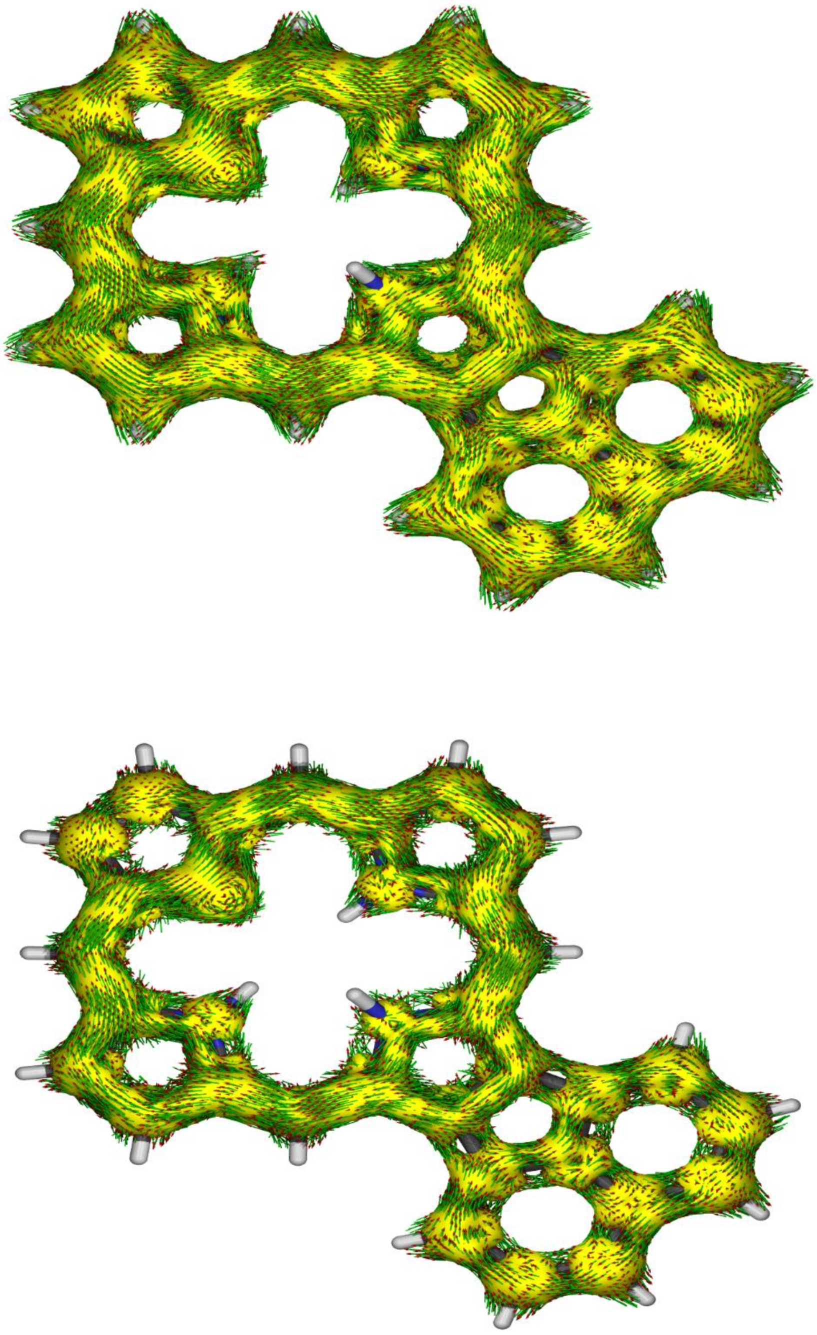

Figure S148. AICD plots for acenaphthoporphyrin cation **ANPb**H^+.
Isovalues 0.05 (top) and 0.07 (bottom).

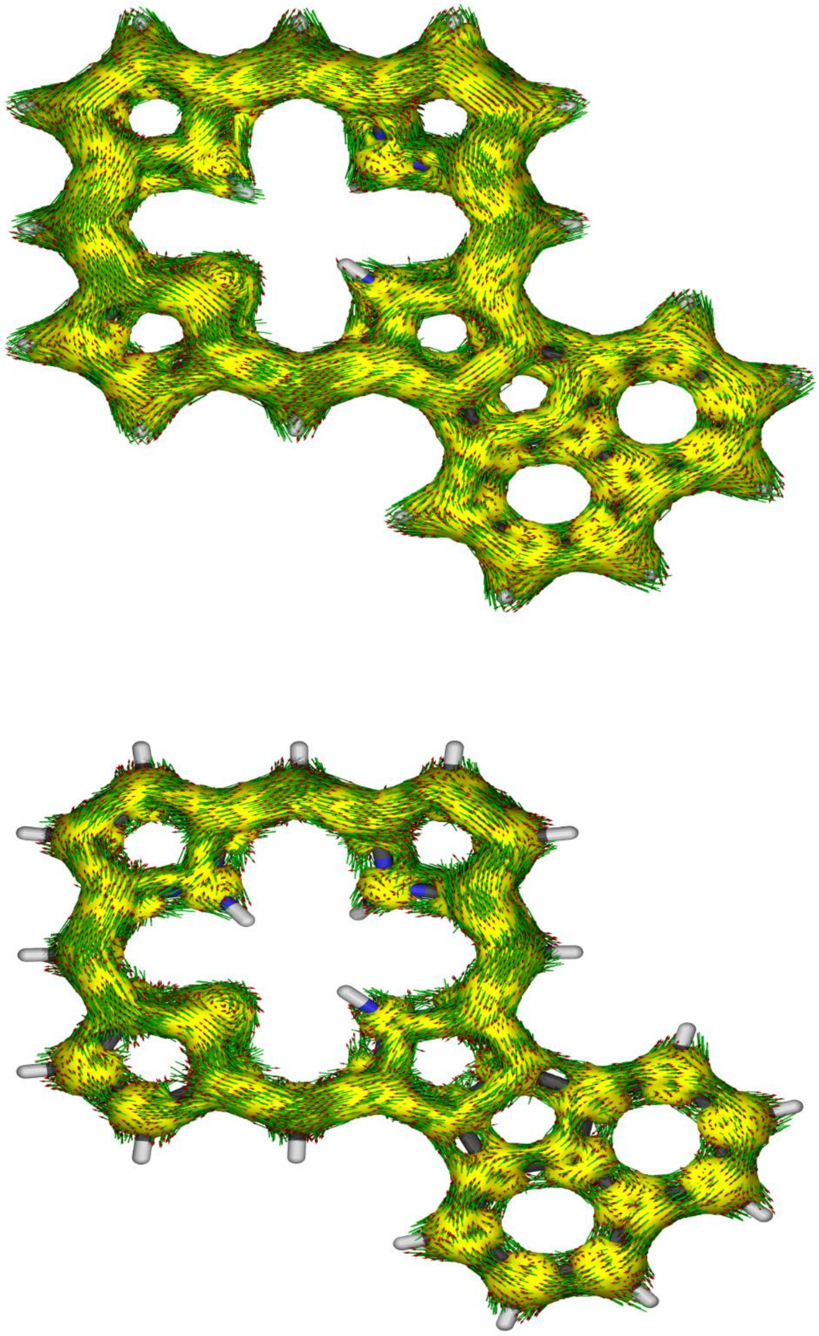

Figure S149. AICD plots for acenaphthoporphyrin cation **ANPc**H^+.
Isovalues 0.05 (top) and 0.07 (bottom).

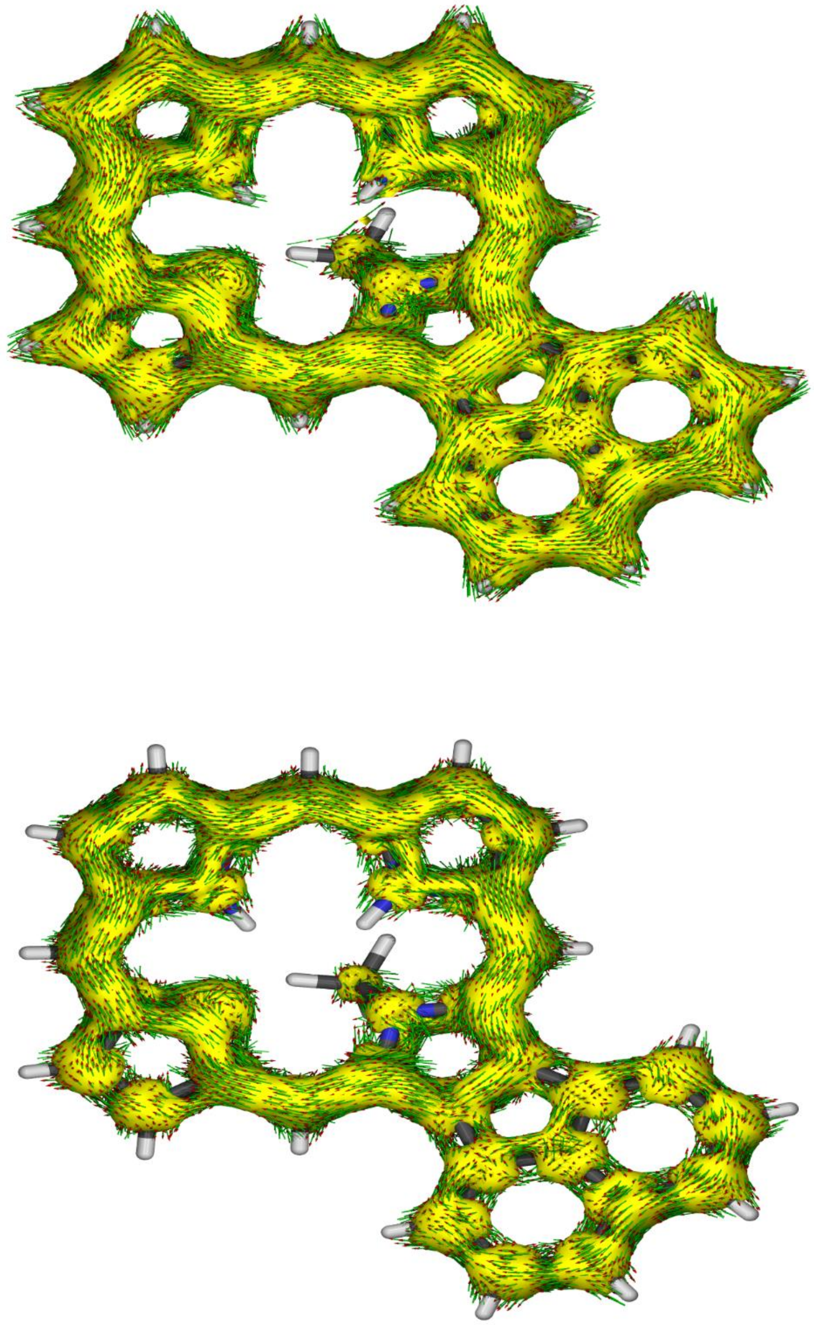

Figure S150. AICD plots for *N*-methylacenaphthoporphyrin cation **MeANPa**H^+.
Isovalues 0.05 (top) and 0.07 (bottom).

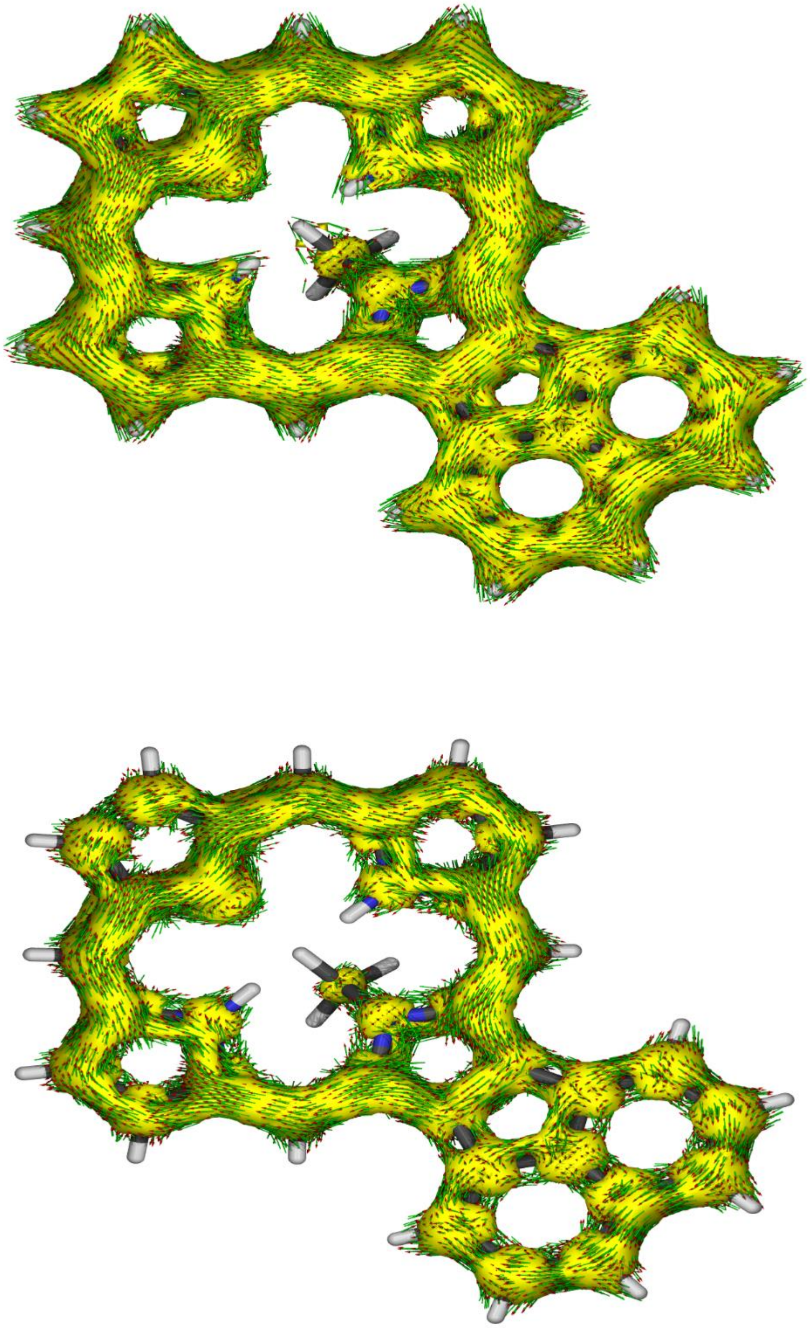

Figure S151. AICD plots for *N*-methylacenaphthoporphyrin cation **MeANPb**H$^+$. Isovalues 0.05 (top) and 0.07 (bottom).

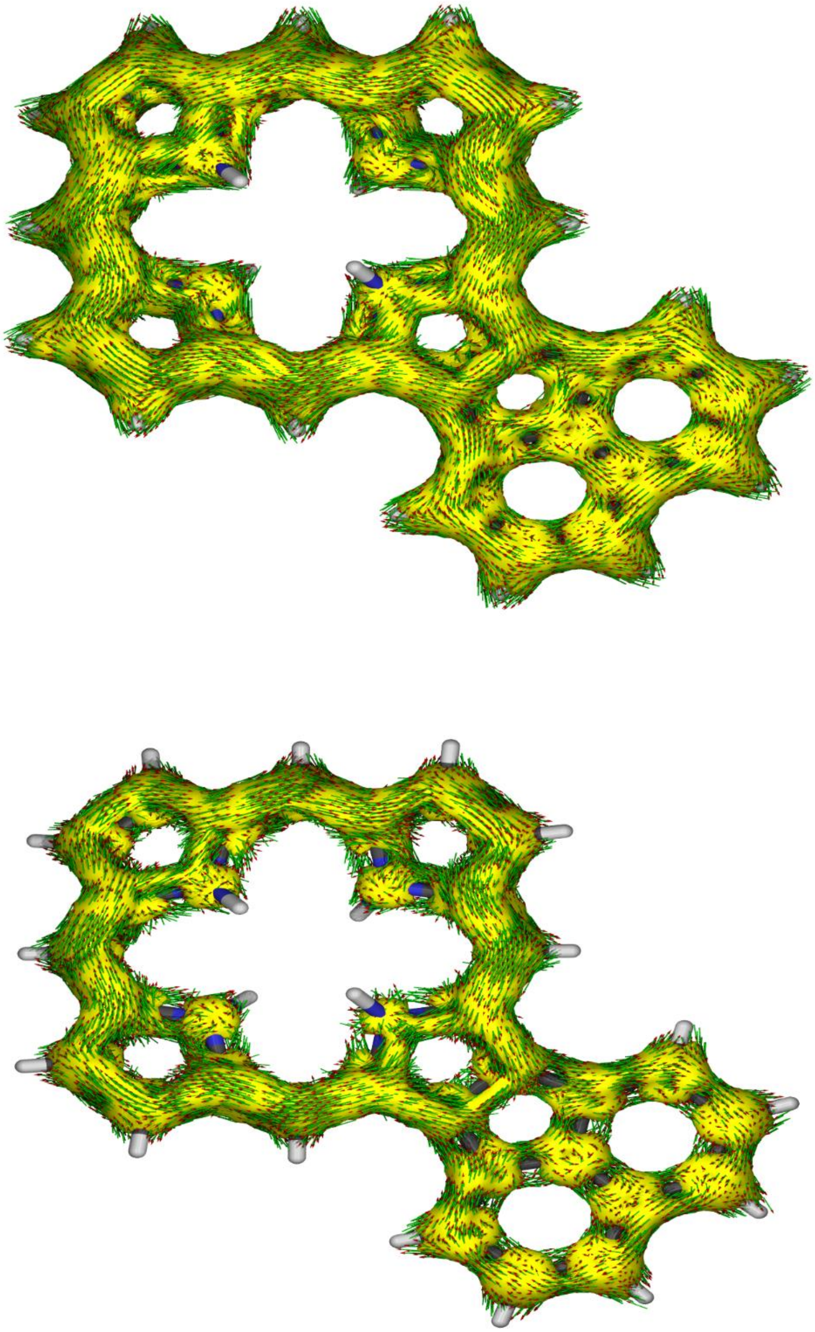

Figure S152. AICD plots for acenaphthoporphyrin dication **ANP**H_2^{2+}. Isovalues 0.05 (top) and 0.07 (bottom).

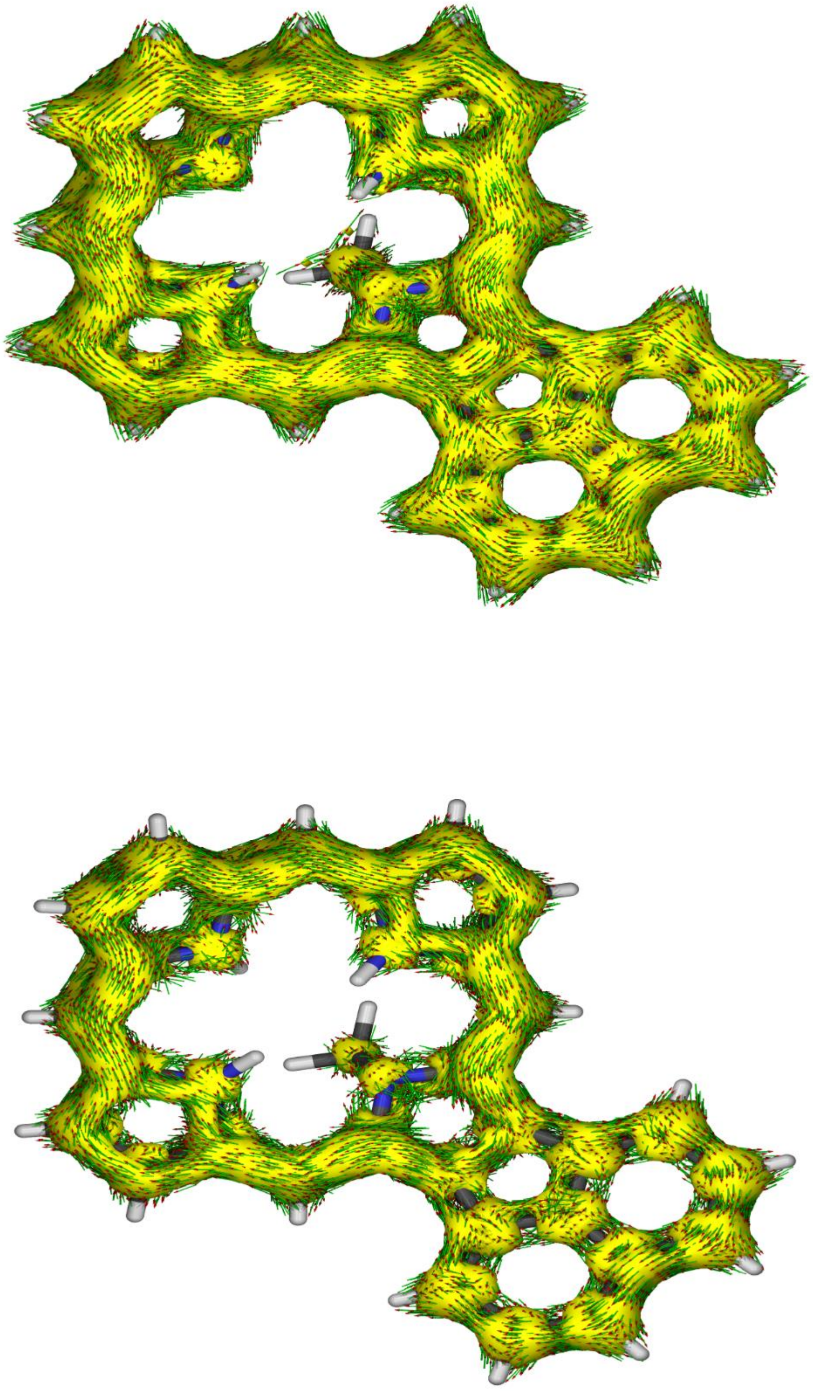

Figure S153. AICD plots for *N*-methylacenaphthoporphyrin dication **MeANP**H_2^{2+}. Isovalues 0.05 (top) and 0.07 (bottom).

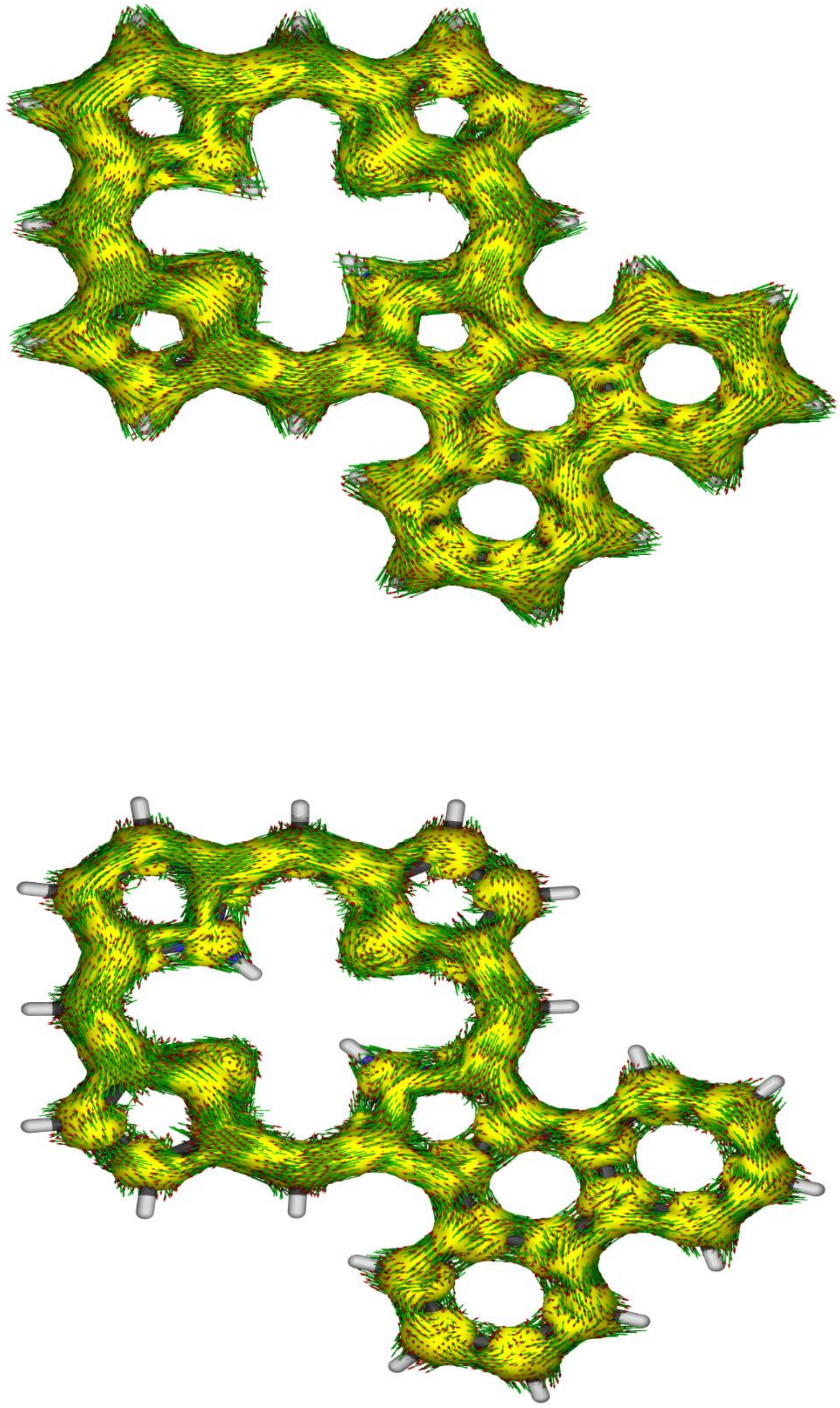

Figure S154. AICD plots for phenanthroporphyrin tautomer **PhPa**.
Isovalues 0.05 (top) and 0.07 (bottom).

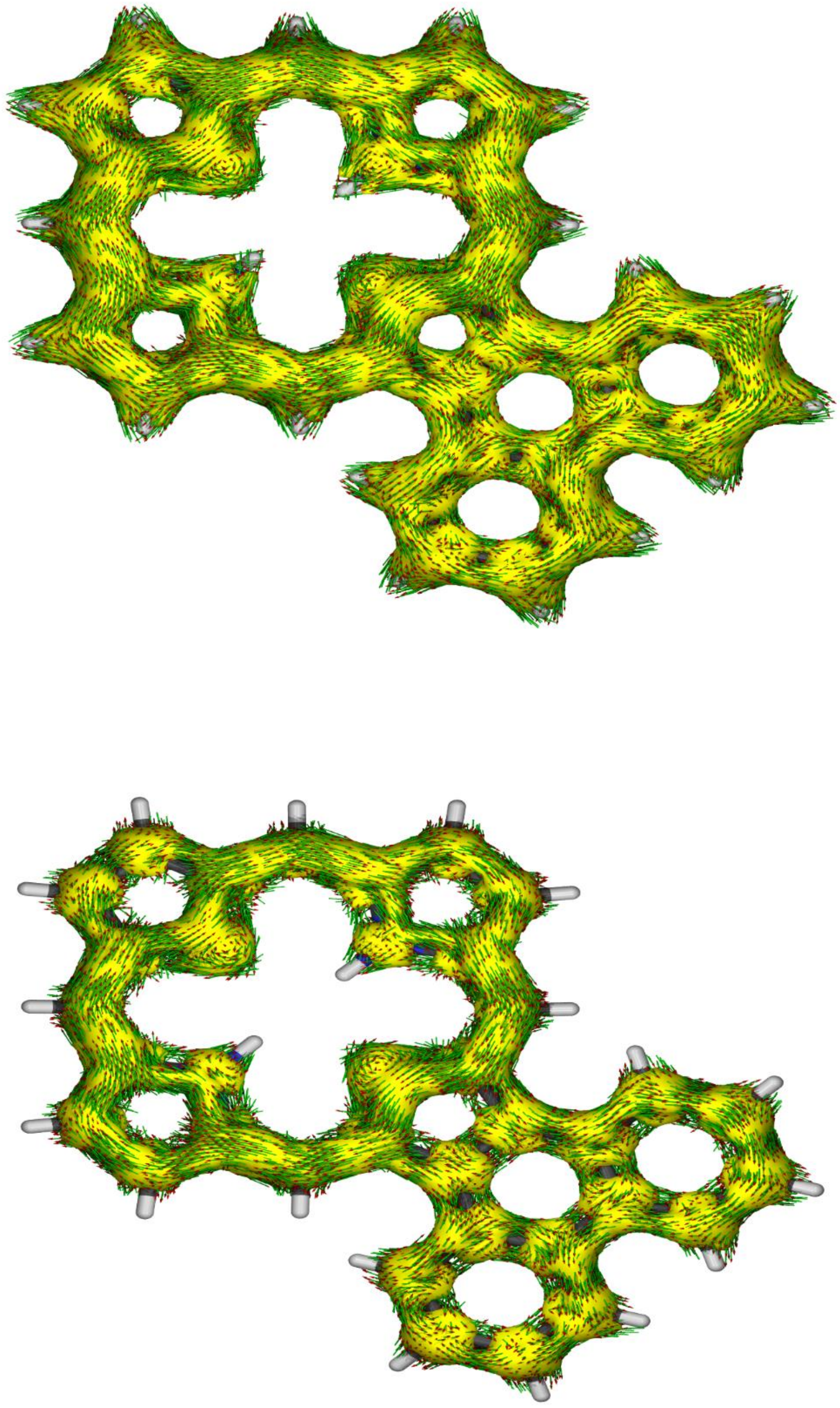

Figure S155. AICD plots for phenanthroporphyrin tautomer **PhPb**.
Isovalues 0.05 (top) and 0.07 (bottom).

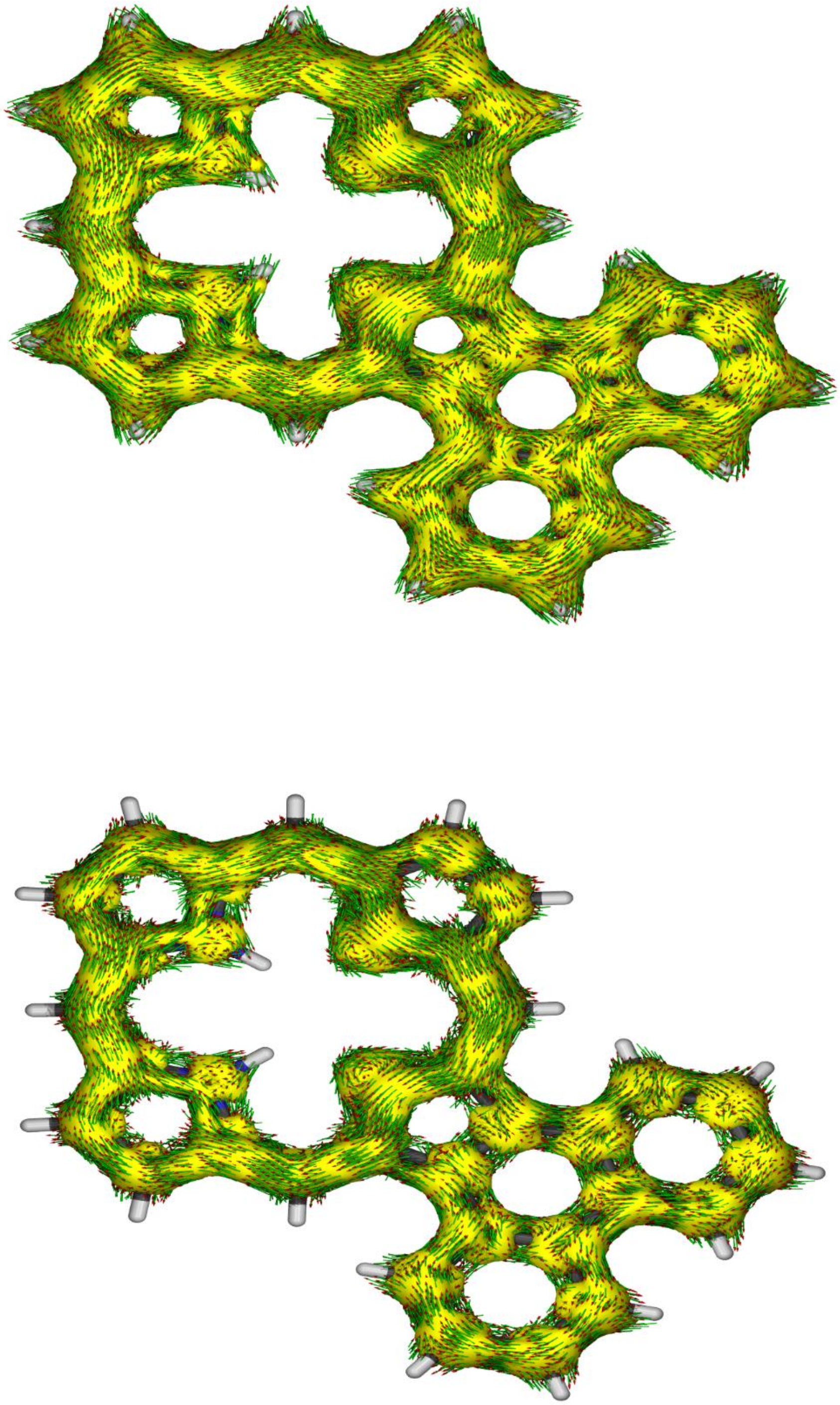

Figure S156. AICD plots for phenanthroporphyrin tautomer **PhPc**.
Isovalues 0.05 (top) and 0.07 (bottom).

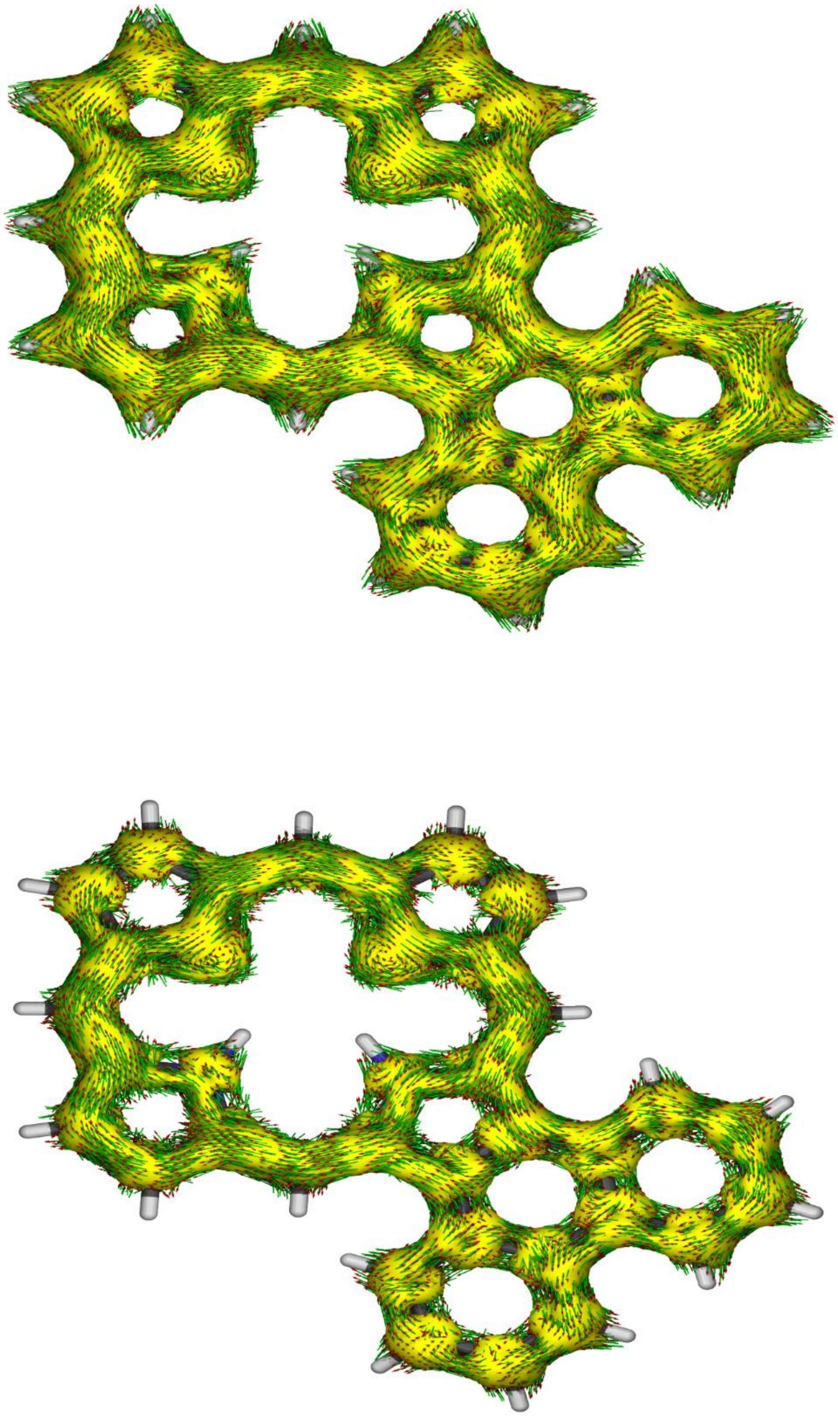

Figure S157. AICD plots for phenanthroporphyrin tautomer **PhPd**.
Isovalues 0.05 (top) and 0.07 (bottom).

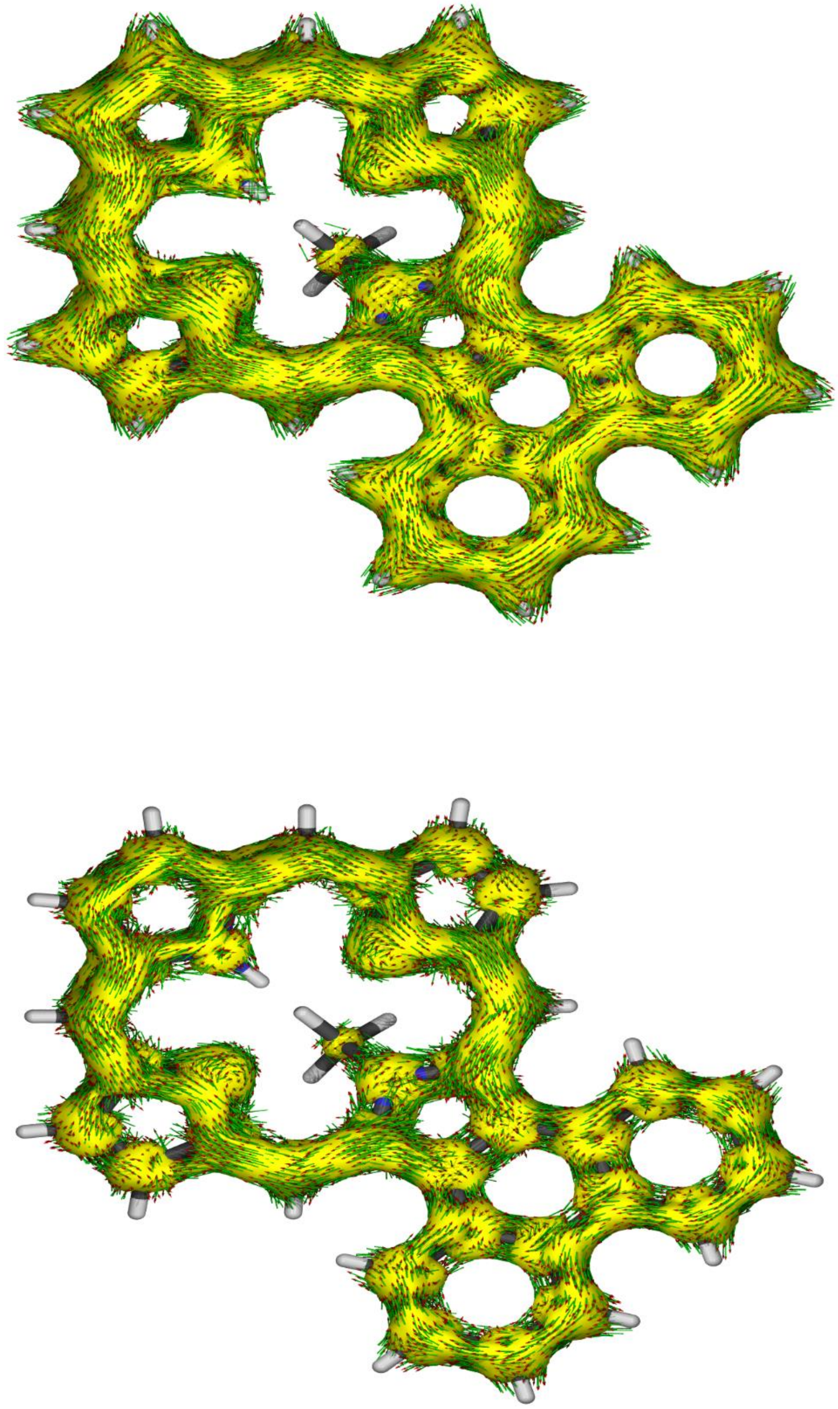

Figure S158. AICD plots for *N*-methylphenanthroporphyrin tautomer **MePhPa**. Isovalues 0.05 (top) and 0.07 (bottom).

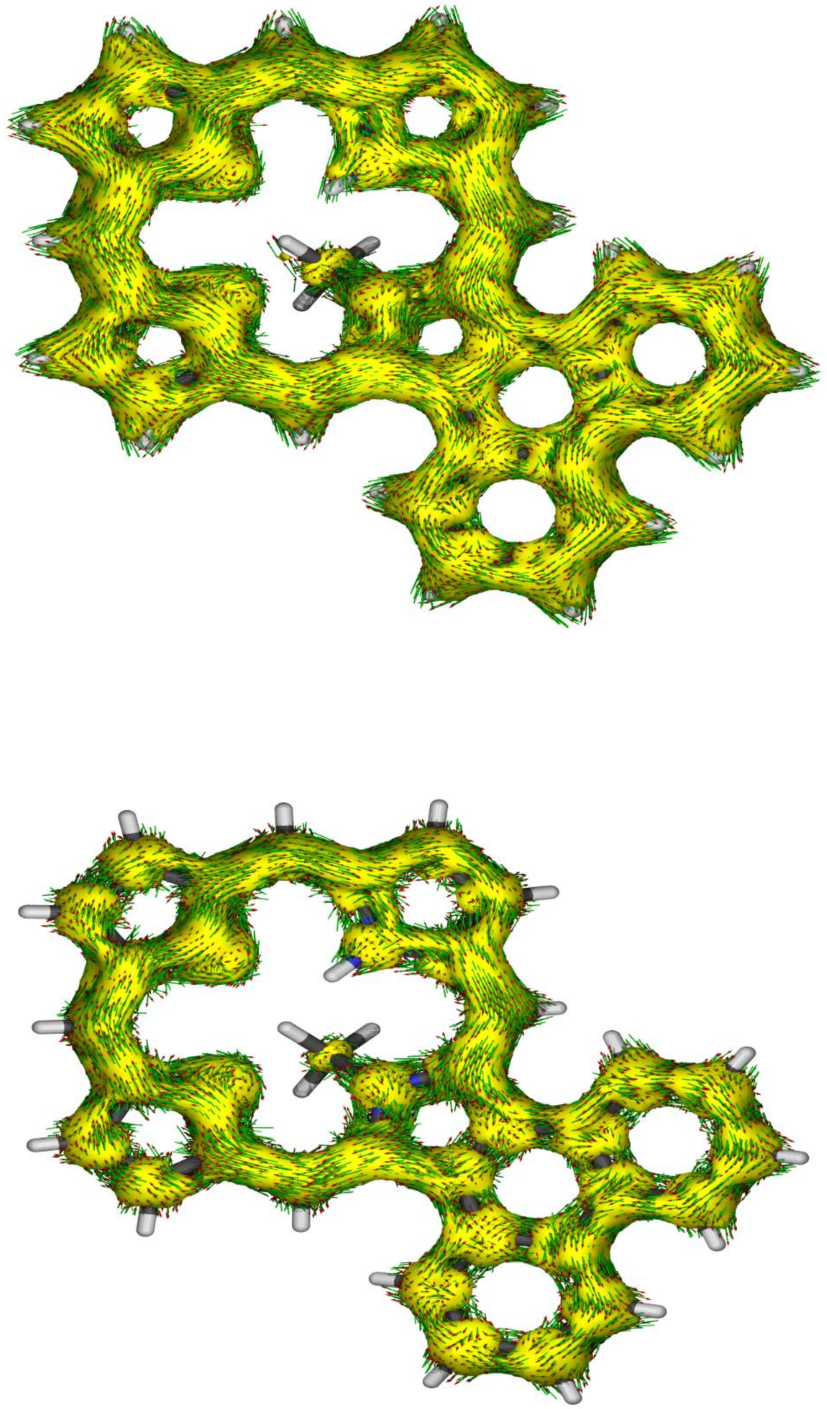

Figure S159. AICD plots for *N*-methylphenanthroporphyrin tautomer **MePhPb**.
Isovalues 0.05 (top) and 0.07 (bottom).

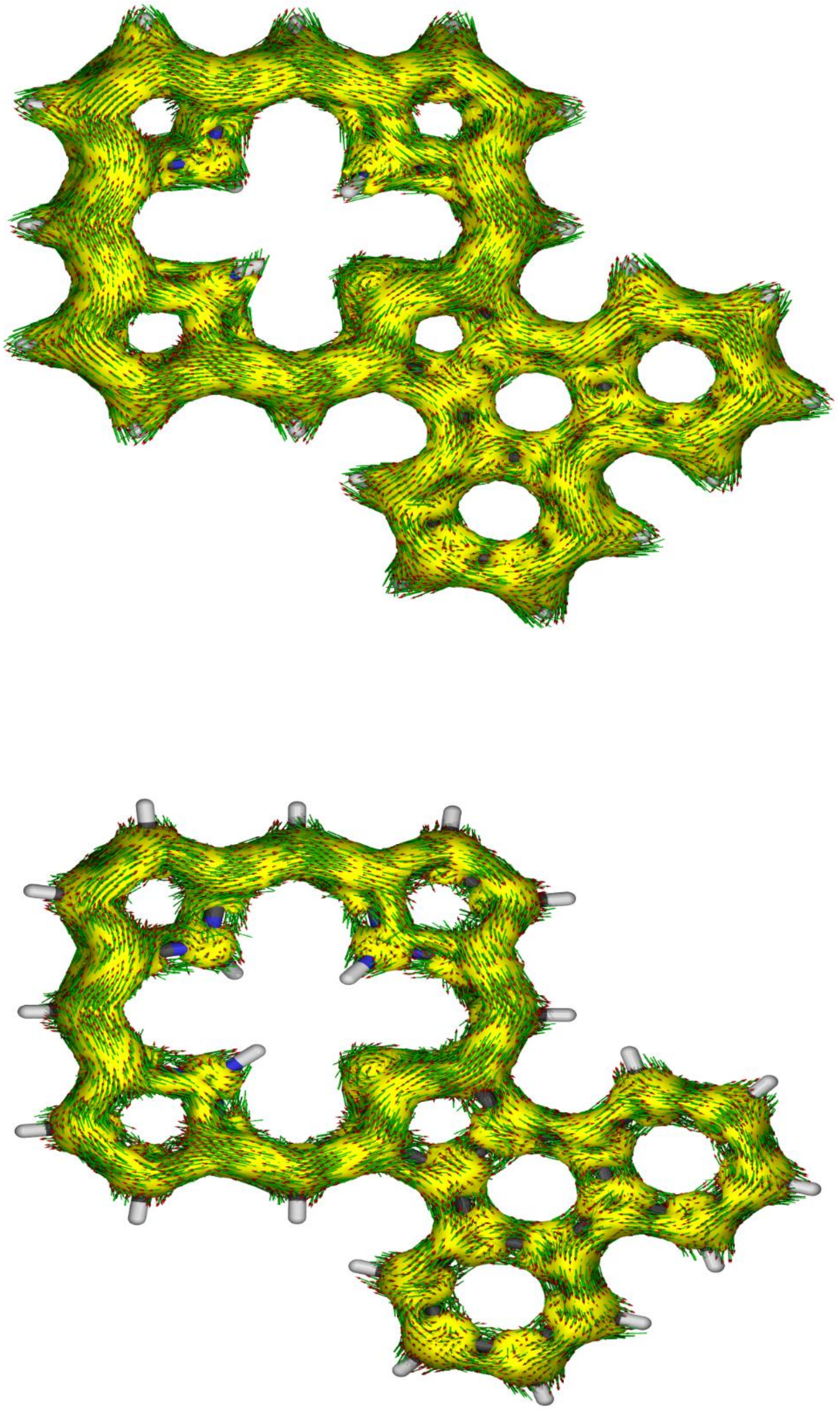

Figure S160. AICD plots for phenanthroporphyrin cation **PhPa**H^{+}. Isovalues 0.05 (top) and 0.07 (bottom).

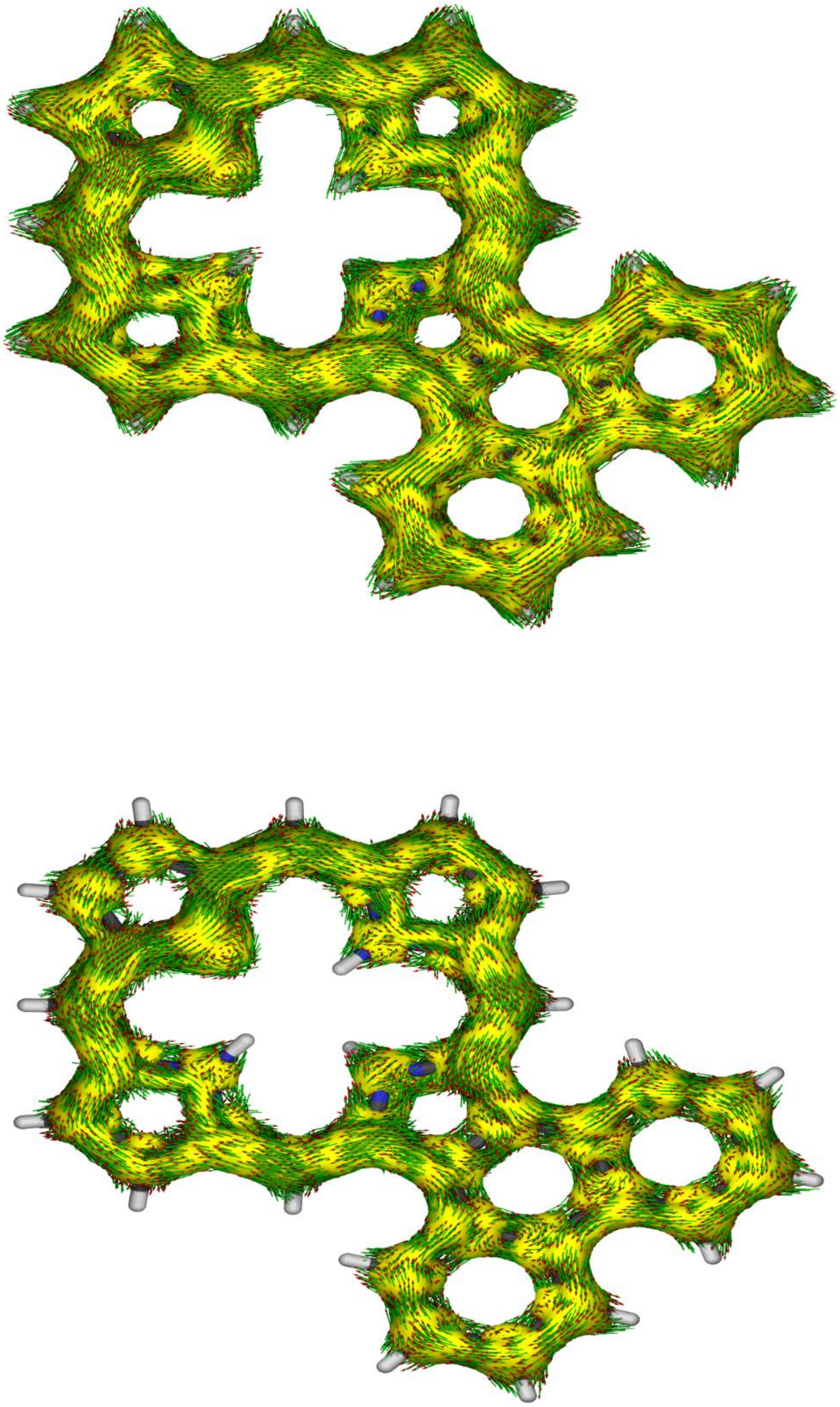

Figure S161. AICD plots for phenanthroporphyrin cation **PhPb**H
$^+$. Isovalues 0.05 (top) and 0.07 (bottom).

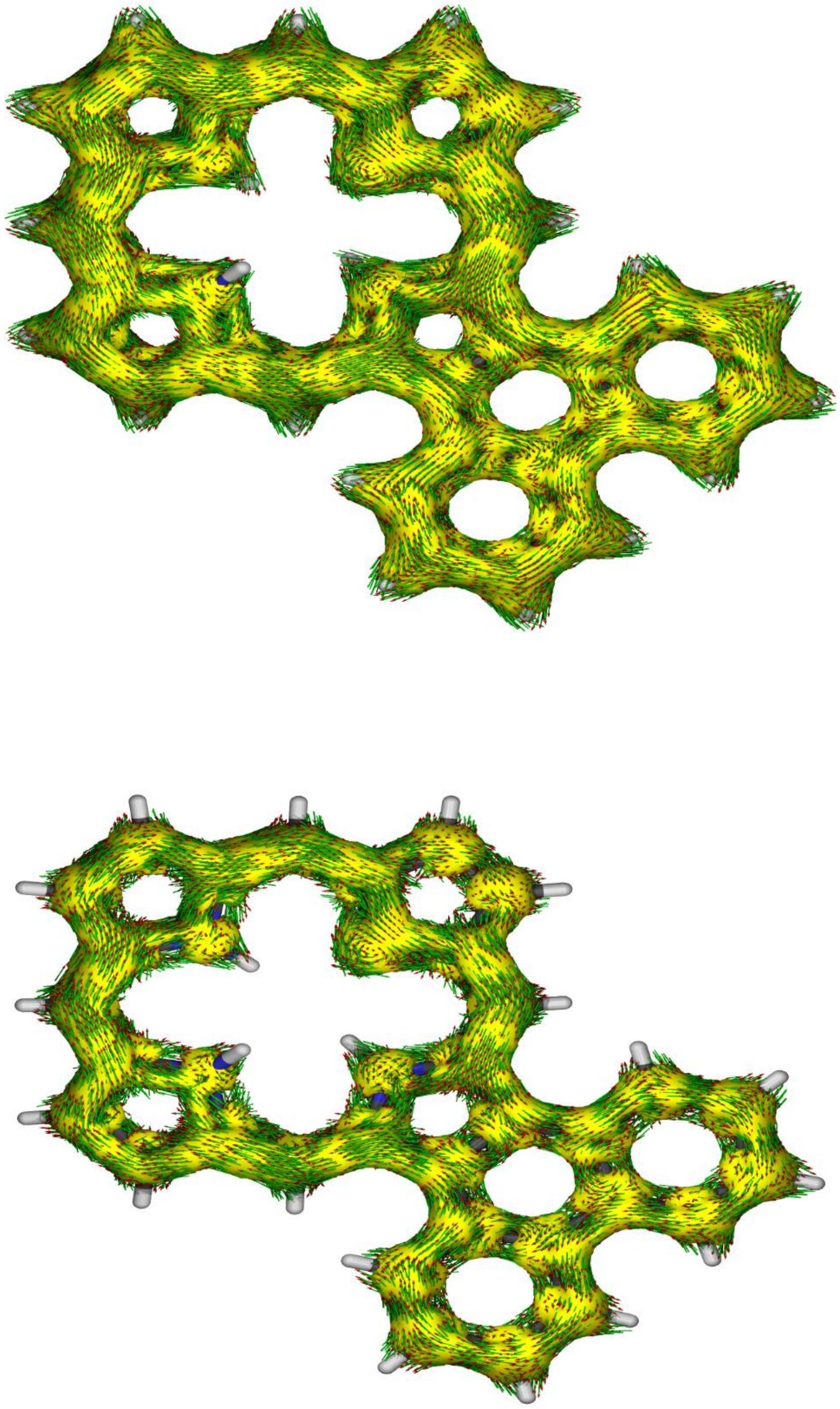

Figure S162. AICD plots for phenanthroporphyrin cation **PhPc**H $^{+}$. Isovalues 0.05 (top) and 0.07 (bottom).

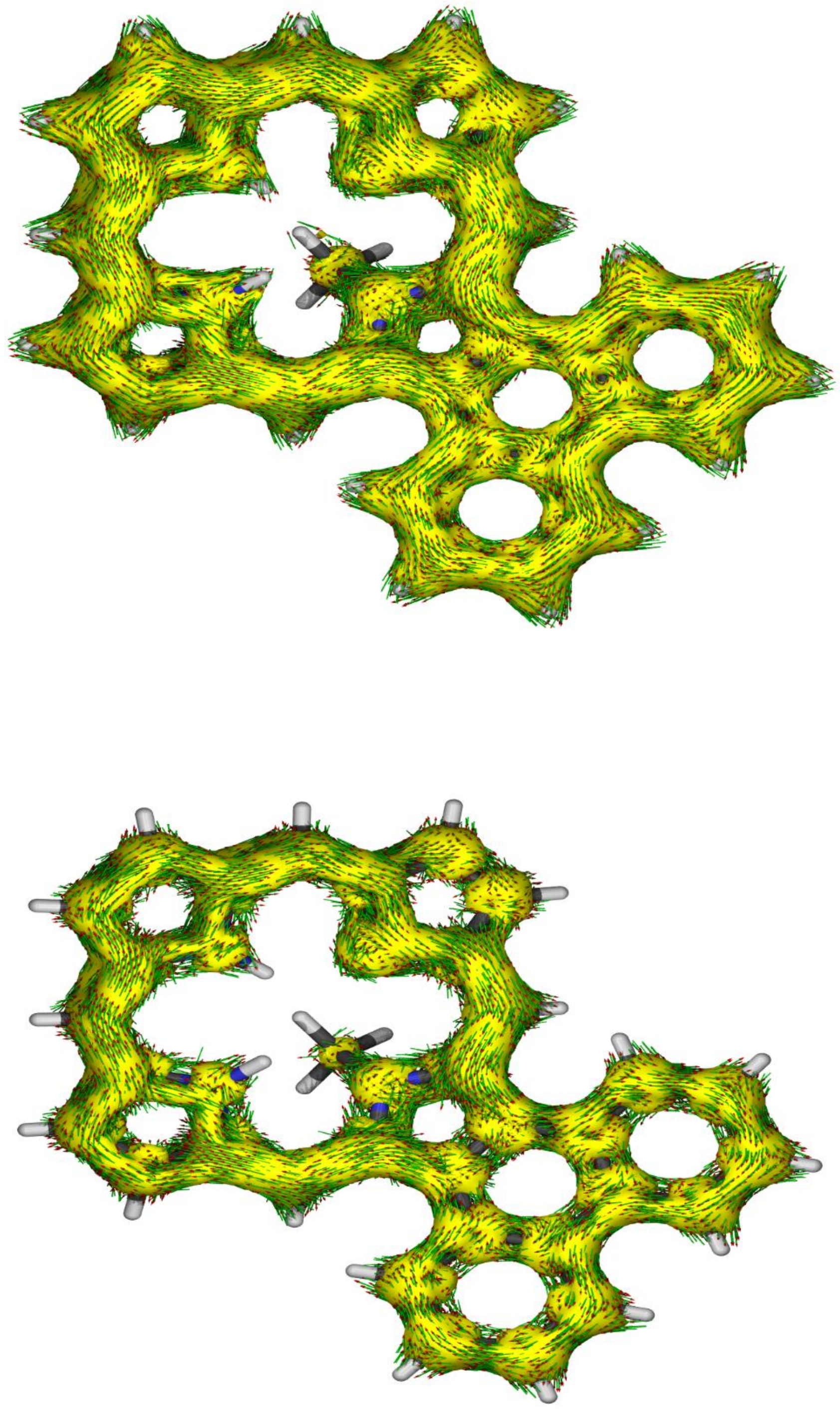

Figure S163. AICD plots for *N*-methylphenanthroporphyrin cation **MePhPa**H$^+$.
Isovalues 0.05 (top) and 0.07 (bottom).

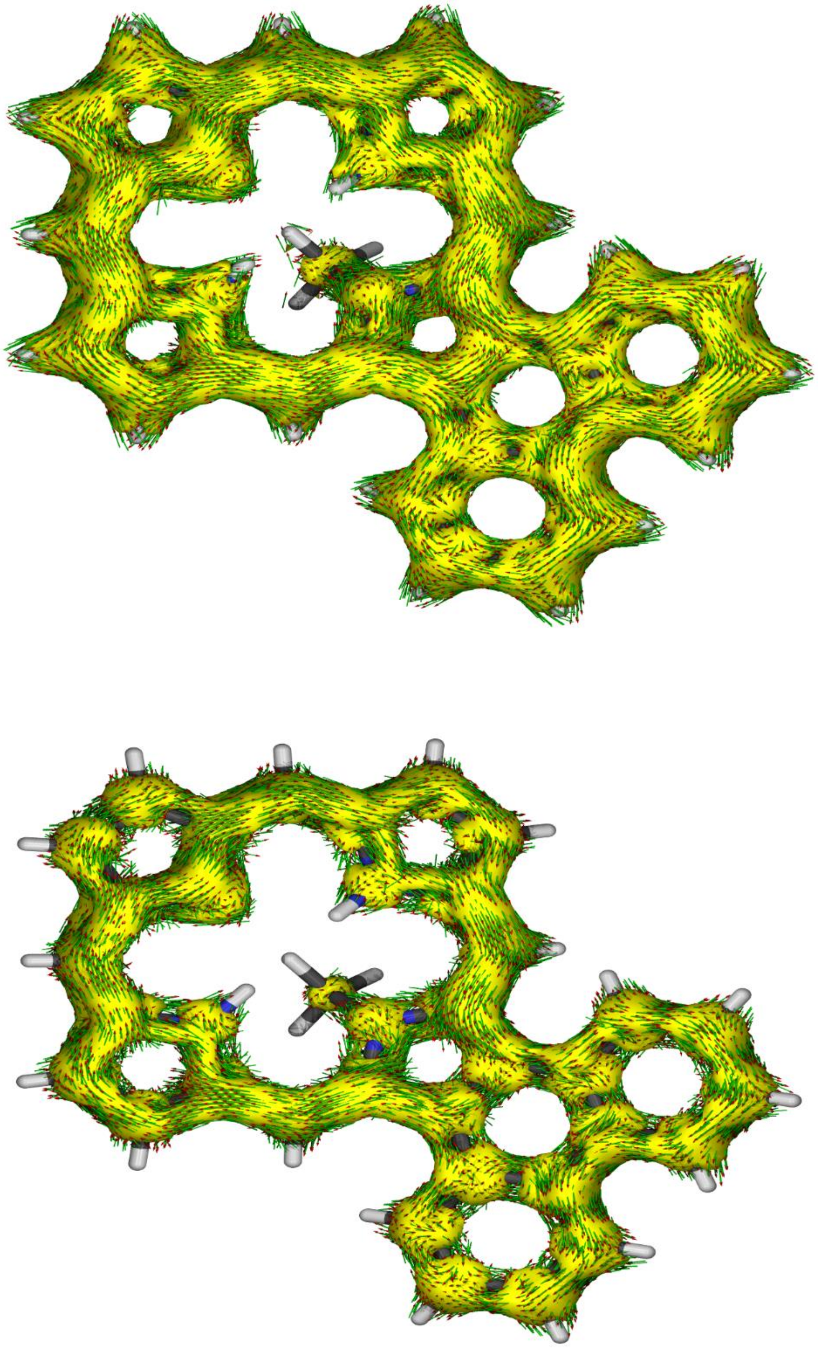

Figure S164. AICD plots for *N*-methylphenanthroporphyrin cation **MePhPb**H$^+$.
Isovalues 0.05 (top) and 0.07 (bottom).

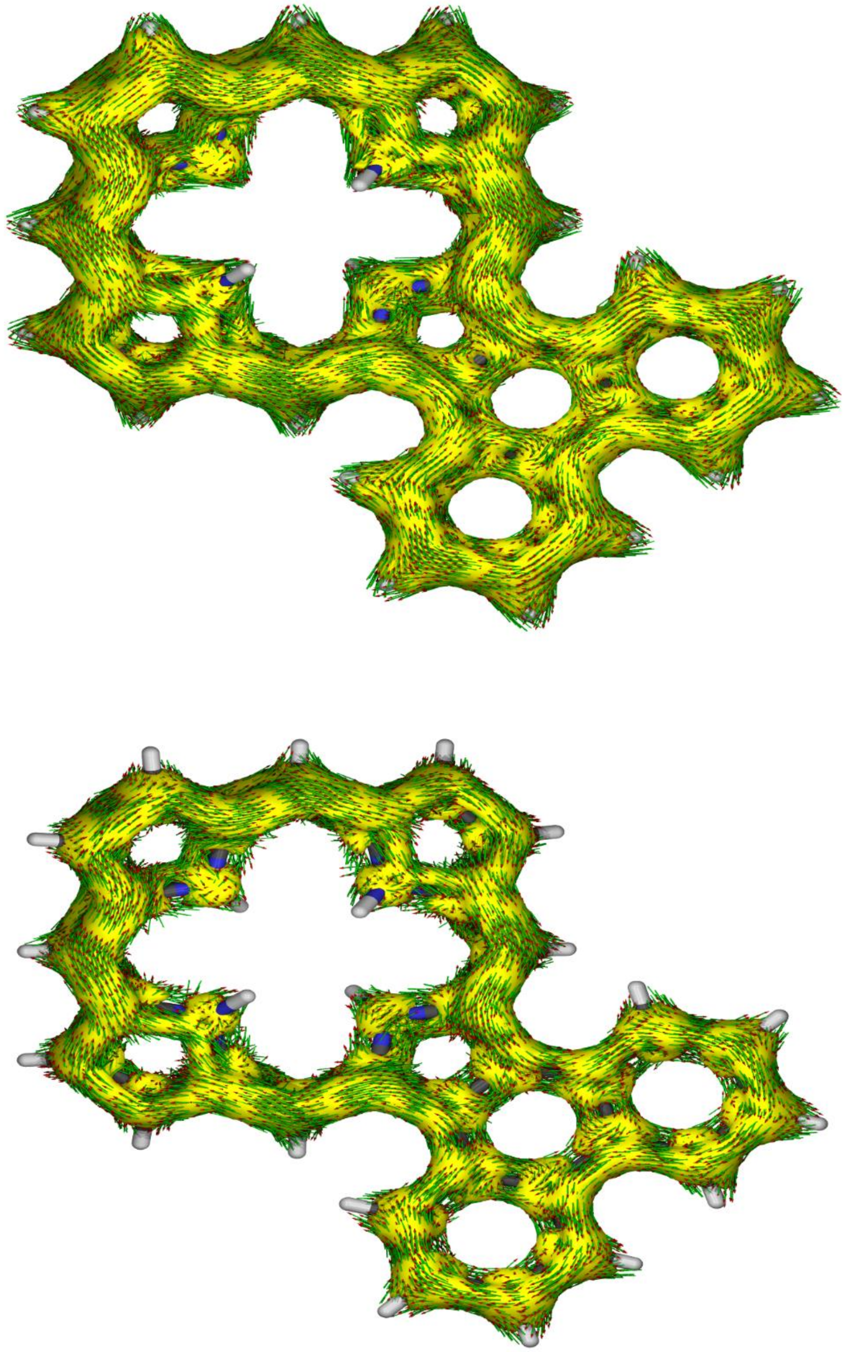

Figure S165. AICD plots for phenanthroporphyrin dication **PhP**H_2^{2+}.
Isovalues 0.05 (top) and 0.07 (bottom).

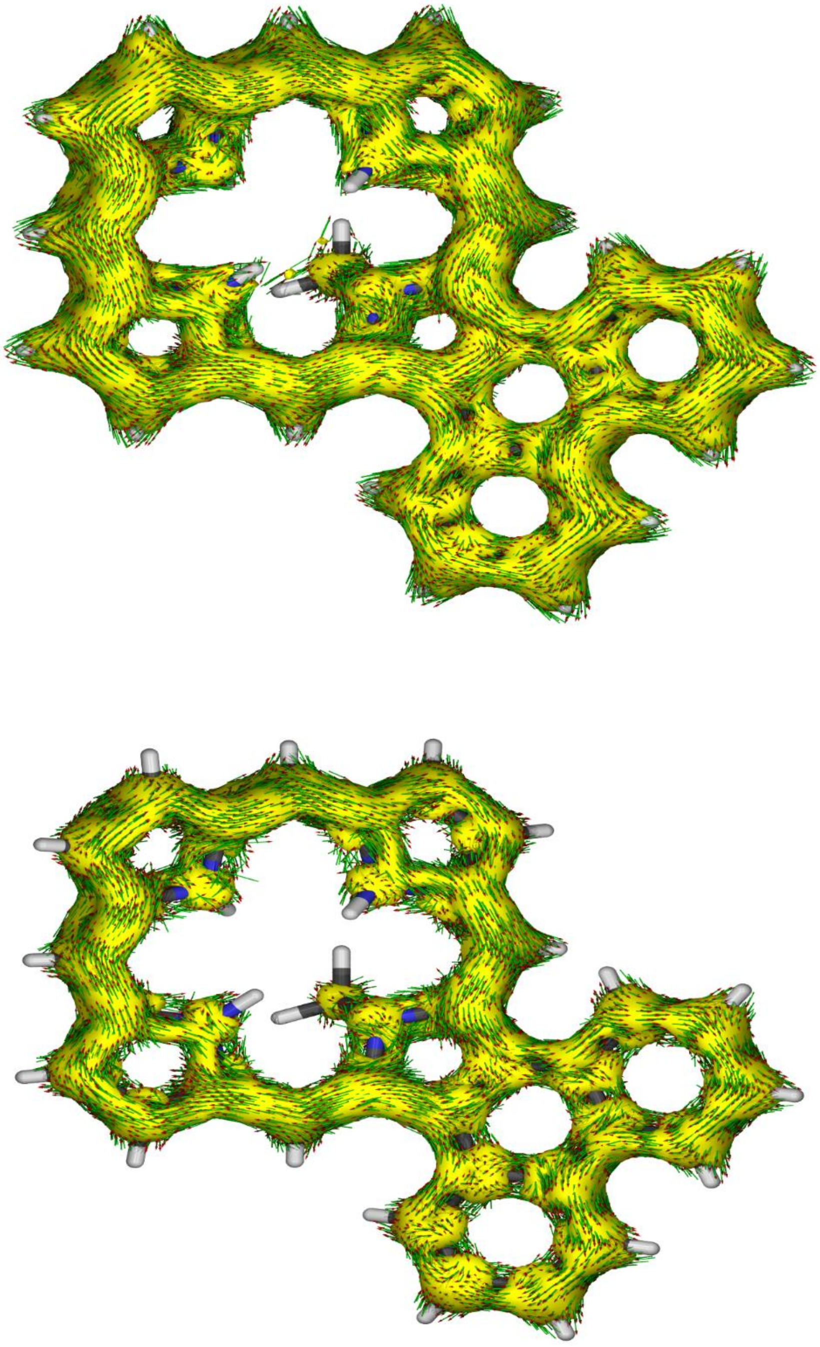

Figure S166. AICD plots for *N*-methylphenanthroporphyrin dication **MePhPa**H_2^{2+}.
Isovalues 0.05 (top) and 0.07 (bottom).

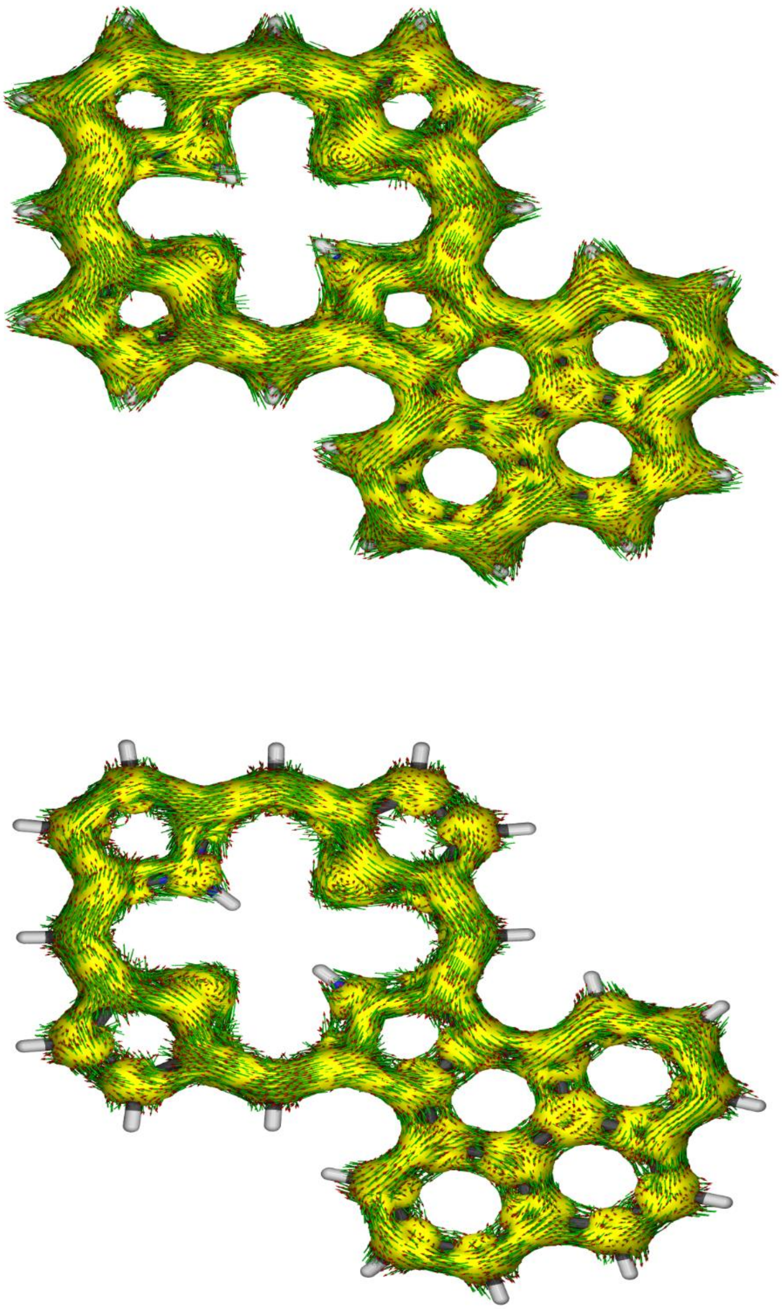

Figure S167. AICD plots for pyrenoporphyrin tautomer **PyPa**.
Isovalues 0.05 (top) and 0.07 (bottom).

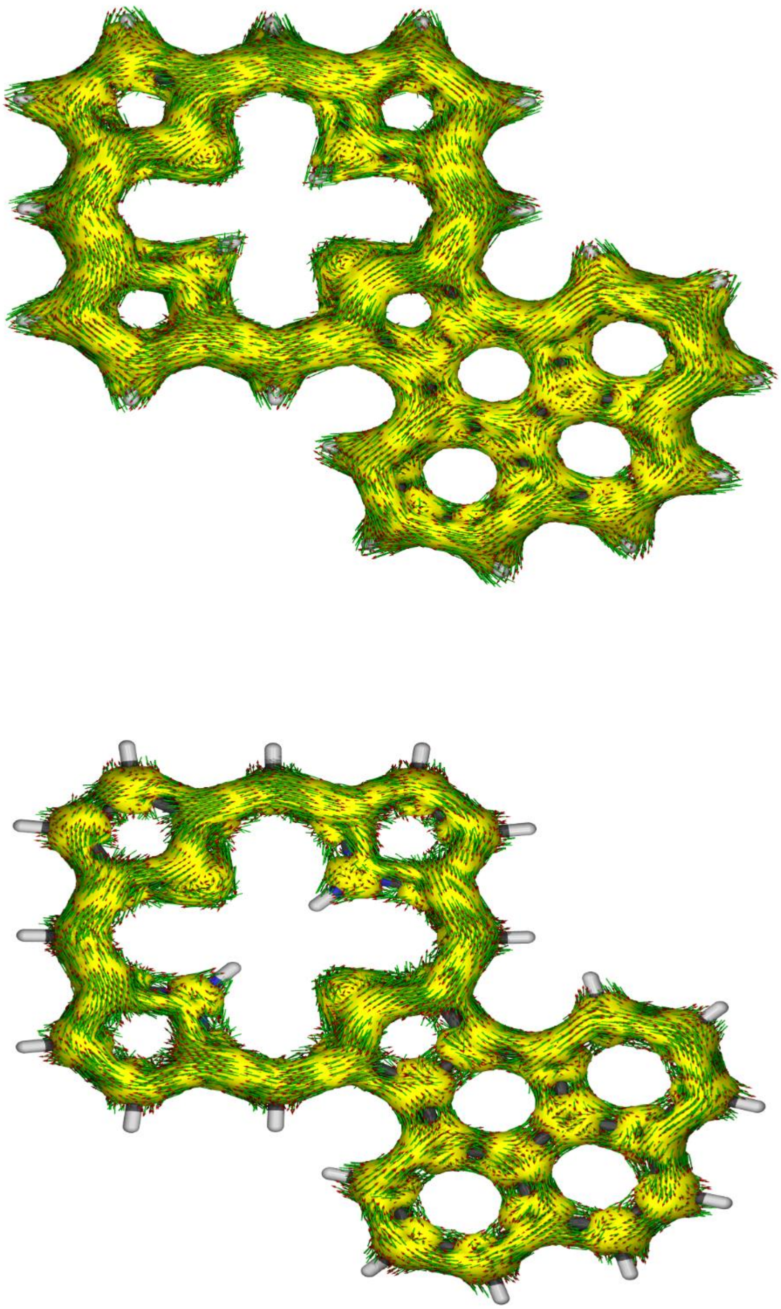

Figure S168. AICD plots for pyrenoporphyrin tautomer **PyPb**.
Isovalues 0.05 (top) and 0.07 (bottom).

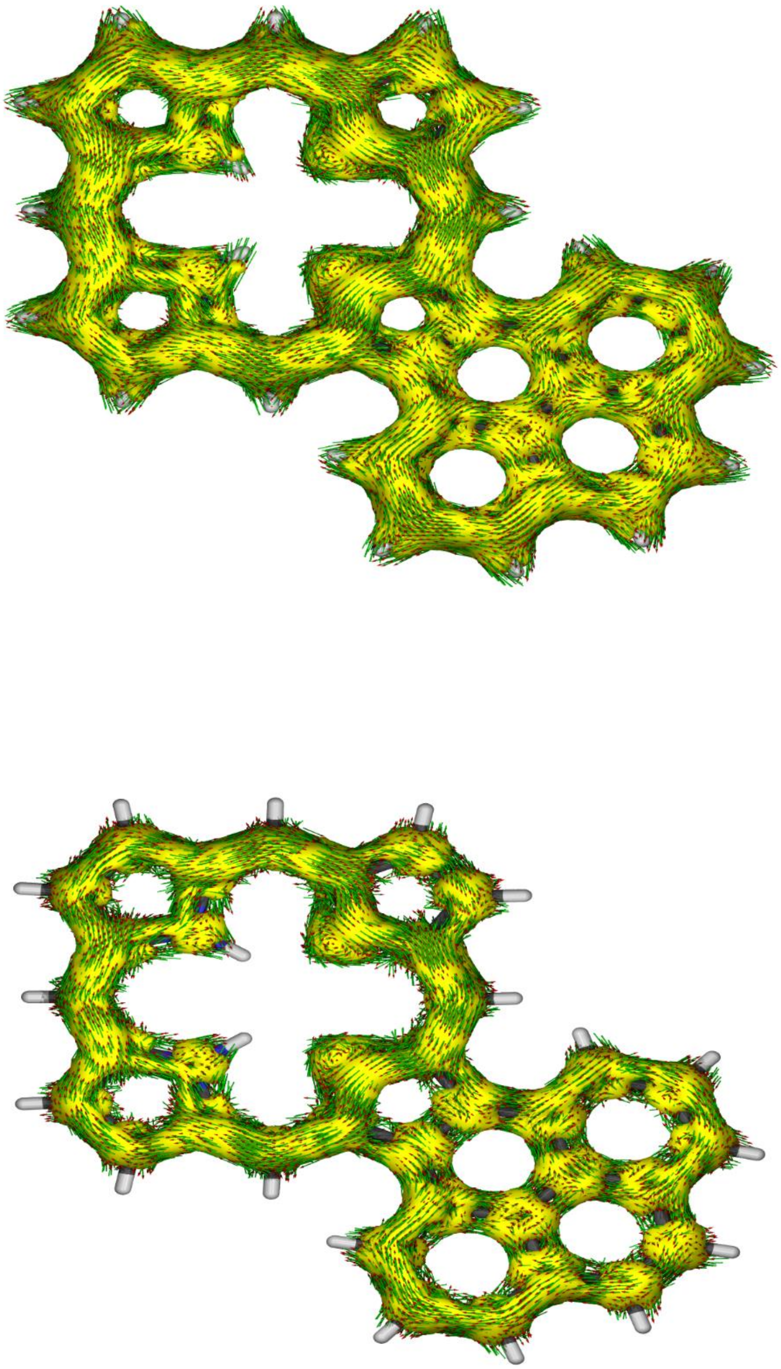

Figure S169. AICD plots for pyrenoporphyrin tautomer **PyPc**.
Isovalues 0.05 (top) and 0.07 (bottom).

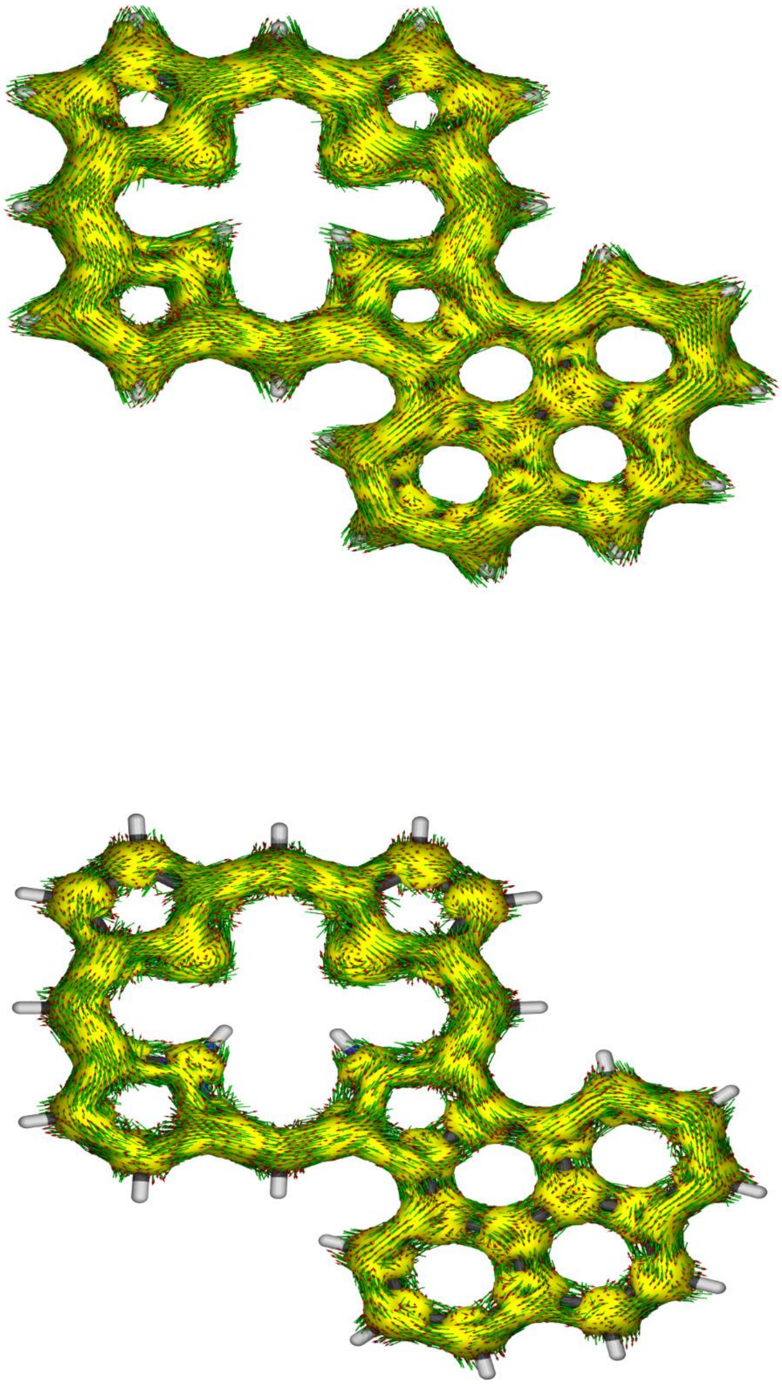

Figure S170. AICD plots for pyrenoporphyrin tautomer **PyPd**.
Isovalues 0.05 (top) and 0.07 (bottom).

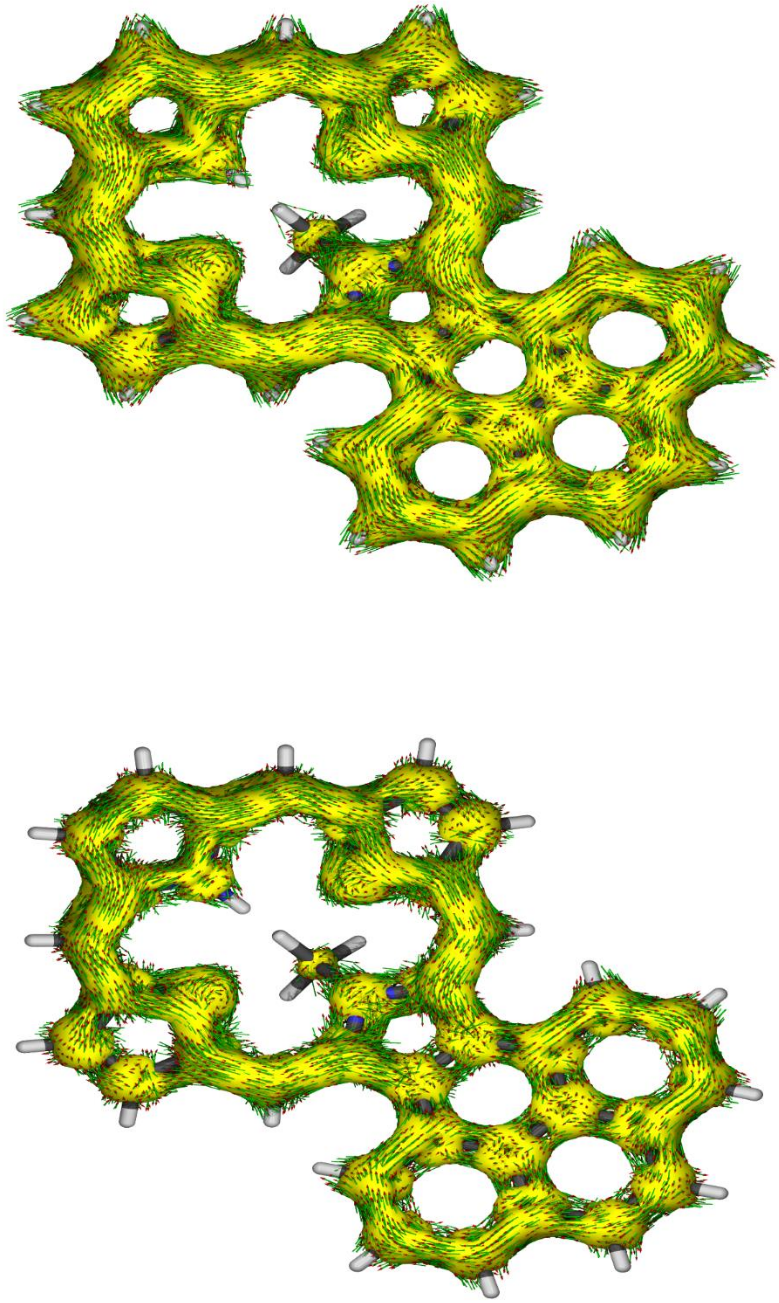

Figure S171. AICD plots for *N*-methylpyrenoporphyrin tautomer **MePyPa**. Isovalues 0.05 (top) and 0.07 (bottom).

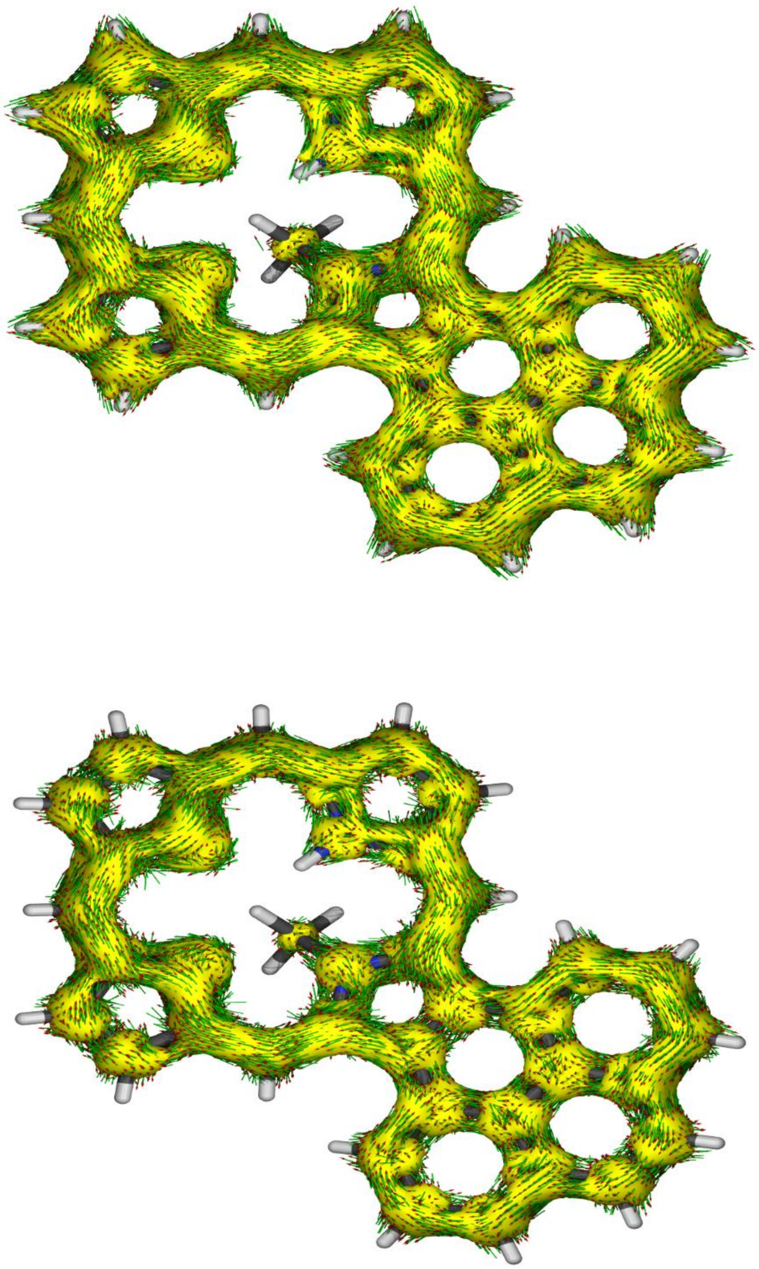

Figure S172. AICD plots for *N*-methylpyrenoporphyrin tautomer **MePyPb**.
Isovalues 0.05 (top) and 0.07 (bottom).

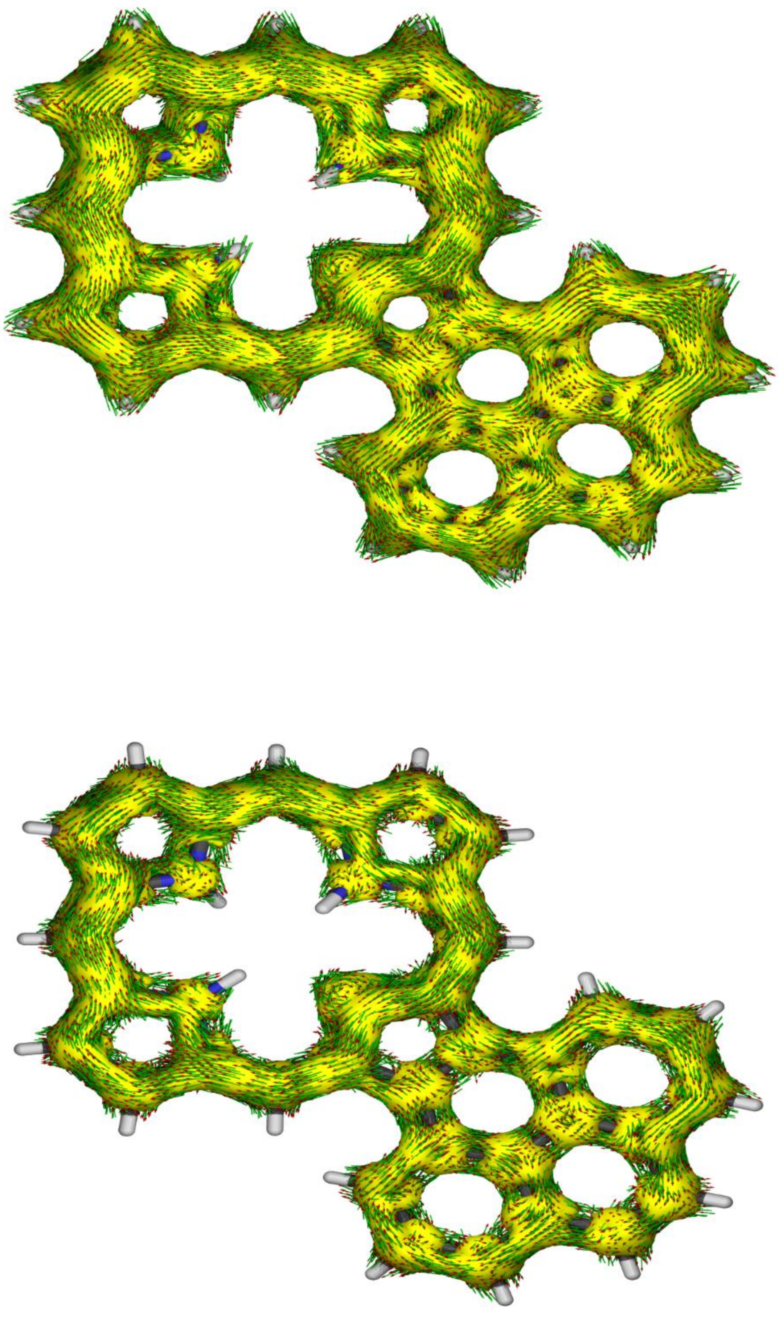

Figure S173. AICD plots for pyrenoporphyrin cation **PyPa**H$^+$.
Isovalues 0.05 (top) and 0.07 (bottom).

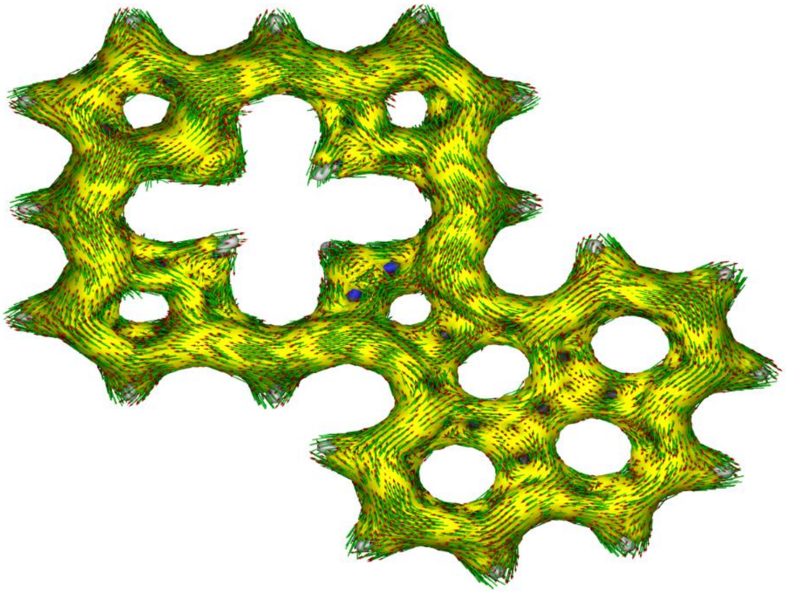

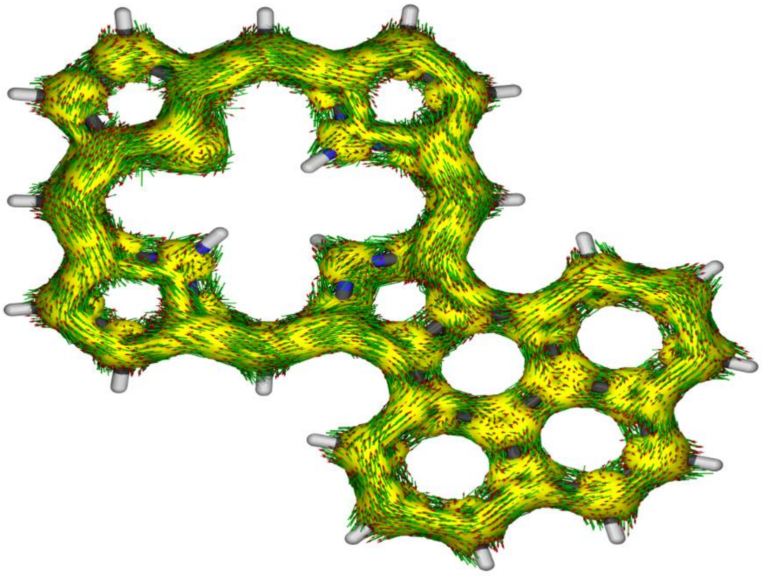

Figure S174. AICD plots for pyrenoporphyrin cation **PyPb**H$^+$.
Isovalues 0.05 (top) and 0.07 (bottom).

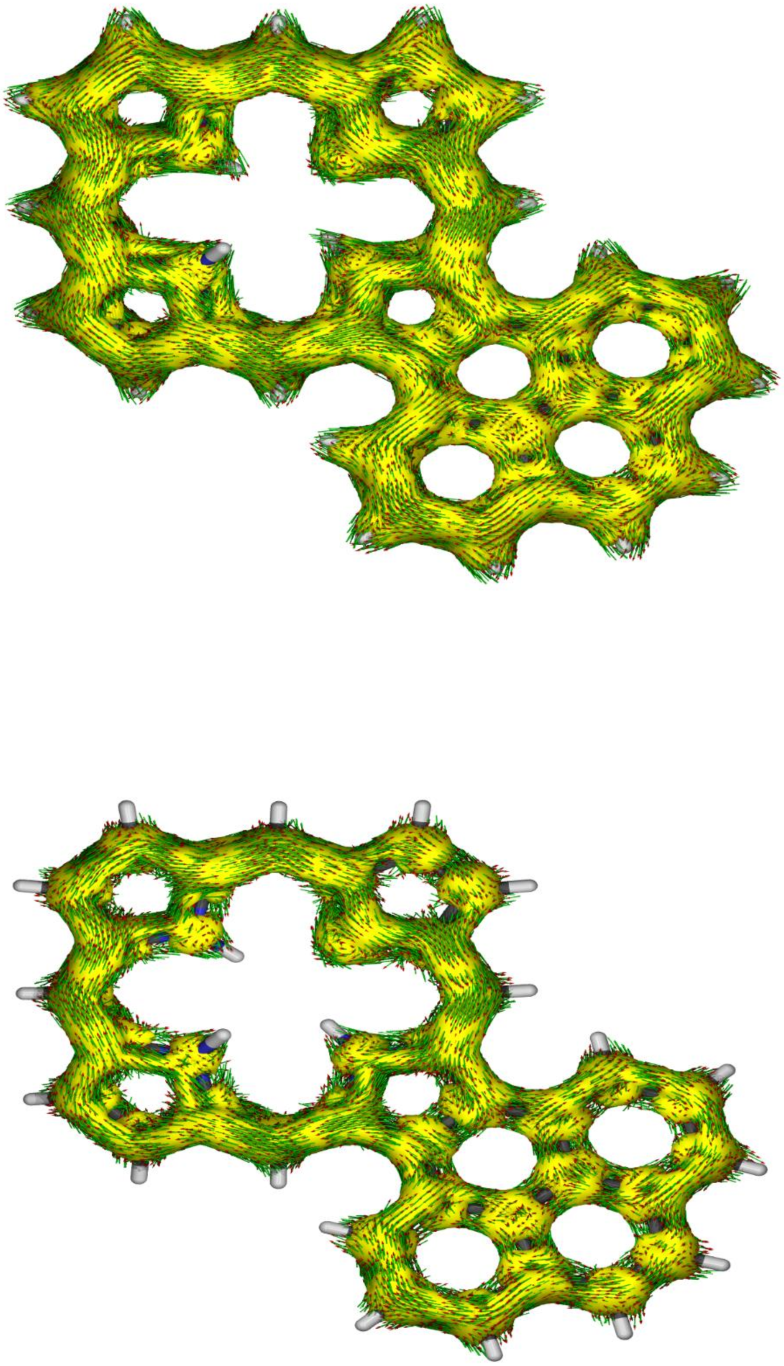

Figure S175. AICD plots for pyrenoporphyrin cation **PyPc**H$^+$.
Isovalues 0.05 (top) and 0.07 (bottom).

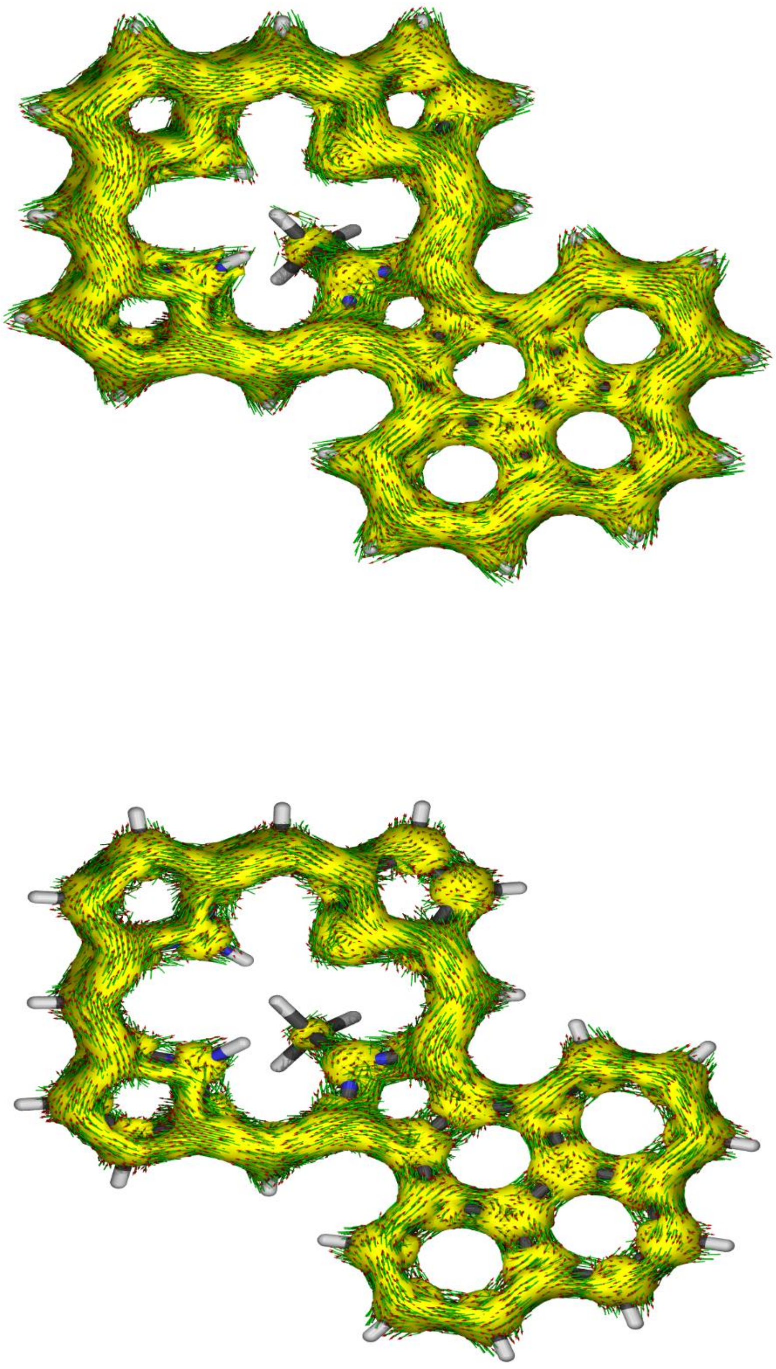

Figure S176. AICD plots for *N*-methylpyrenoporphyrin cation **MePyPa**H$^+$. Isovalues 0.05 (top) and 0.07 (bottom).

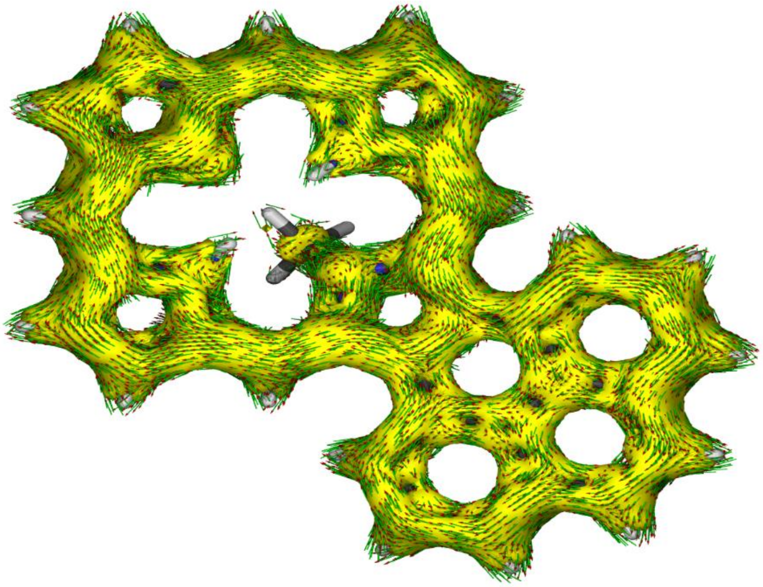

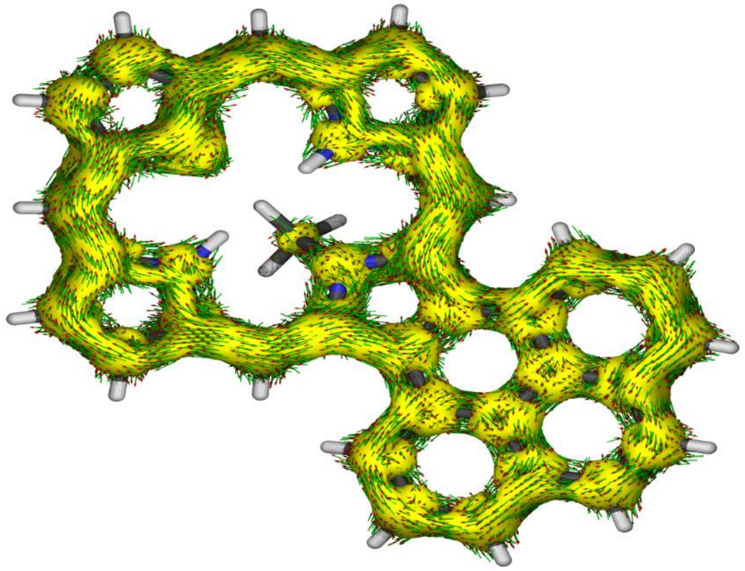

Figure S177. AICD plots for *N*-methylpyrenoporphyrin cation **MePyPb**H$^+$.
Isovalues 0.05 (top) and 0.07 (bottom).

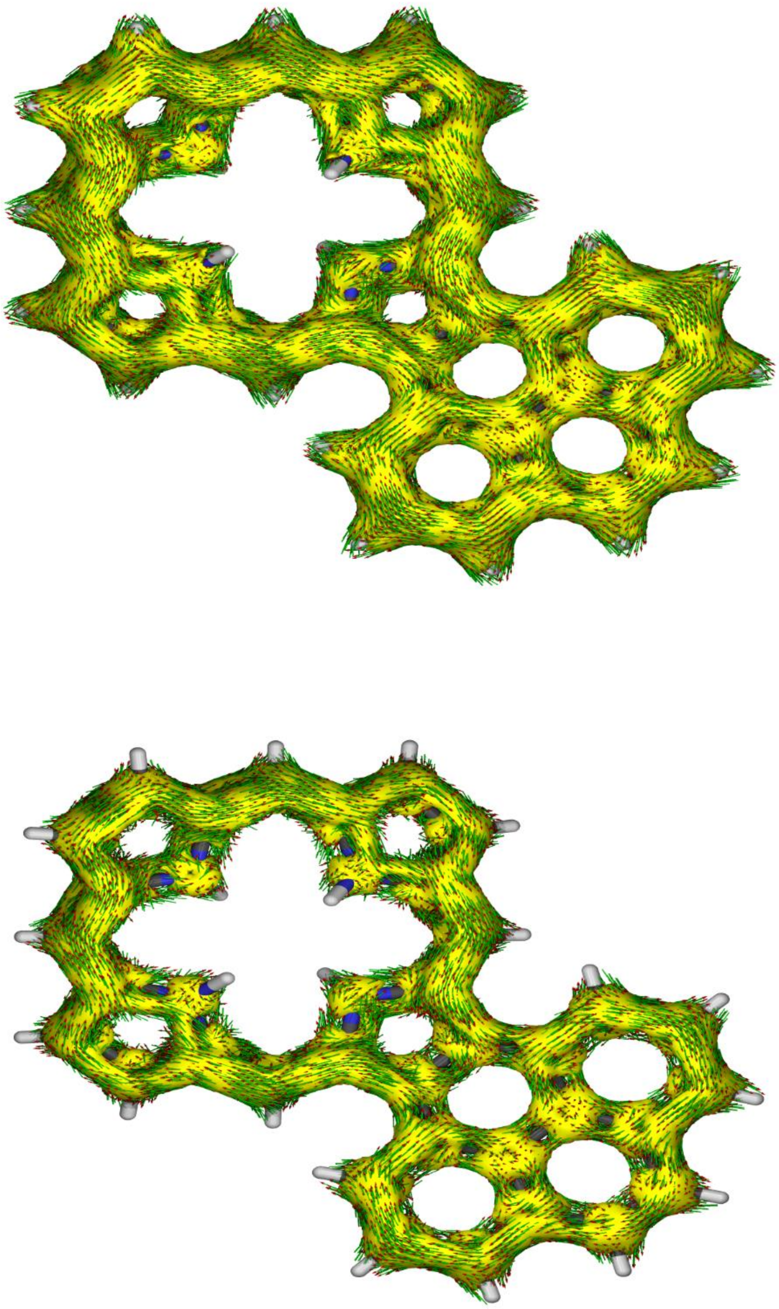

Figure S178. AICD plots for pyrenoporphyrin dication **PyP**H_2^{2+}.
Isovalues 0.05 (top) and 0.07 (bottom).

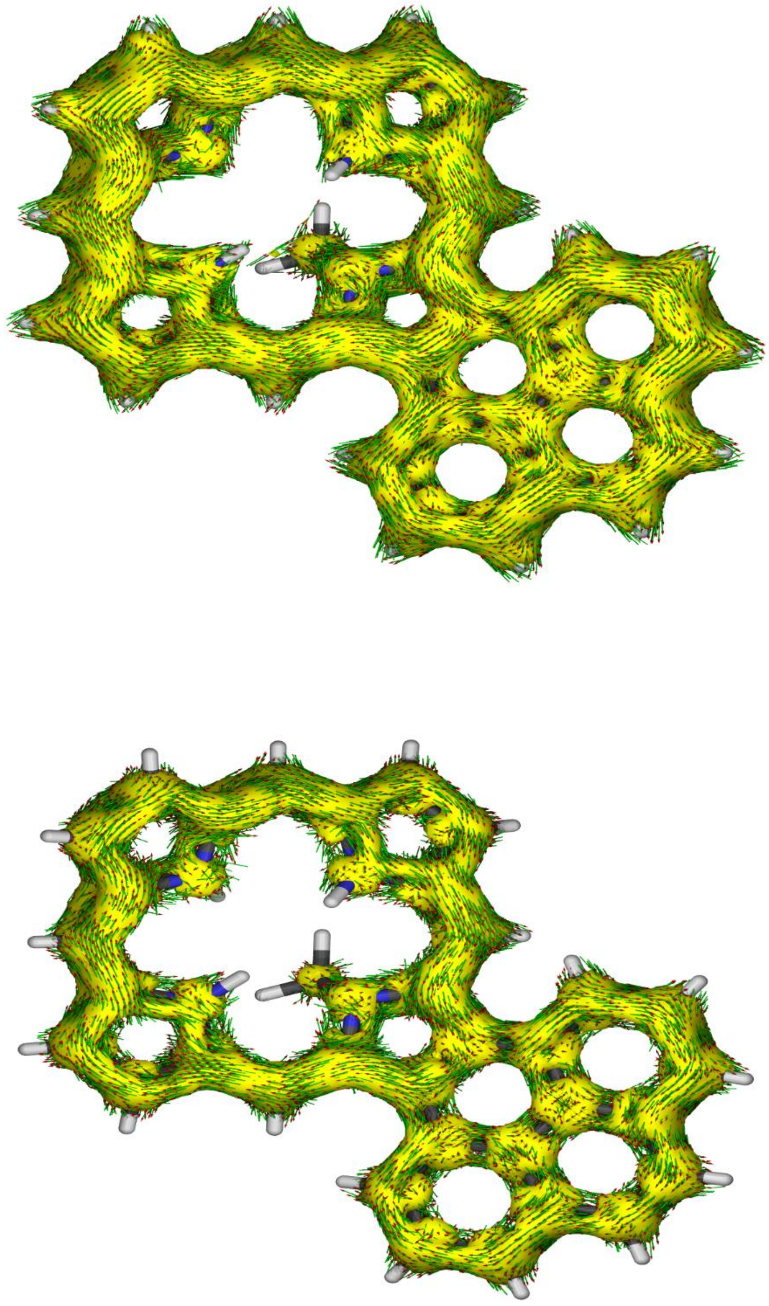

Figure S179. AICD plots for *N*-methylpyrenoporphyrin dication **MePyP**H_2^{2+}.
Isovalues 0.05 (top) and 0.07 (bottom).

Table S7. Cartesian coordinates for acenaphtho-, phenanthro- and pyrenoporphyrin tautomers and related protonated species.

ANPa

C	2.215301000	-0.693274000	-0.000465000
C	2.215294000	0.693279000	-0.000819000
C	0.860365000	1.137614000	-0.000712000
N	0.089694000	-0.000017000	-0.000426000
C	0.860394000	-1.137631000	-0.000208000
C	-2.707798000	-4.240327000	-0.000458000
C	-1.358351000	-4.241920000	-0.000264000
C	-0.953279000	-2.840015000	-0.000167000
N	-2.029890000	-2.017427000	-0.000280000
C	-3.109131000	-2.837656000	-0.000358000
C	0.382476000	-2.440158000	-0.000159000
C	-6.269171000	0.682636000	0.000584000
C	-6.269162000	-0.682649000	0.000090000
C	-4.905321000	-1.122238000	-0.000180000
N	-4.127612000	0.000005000	0.000111000
C	-4.905338000	1.122242000	0.000594000
C	-4.442720000	-2.431163000	-0.000425000
C	-1.358369000	4.241907000	0.000167000
C	-2.707815000	4.240327000	0.000525000
C	-3.109153000	2.837655000	0.000540000
N	-2.029920000	2.017420000	0.000100000
C	-0.953306000	2.840003000	-0.000073000
C	0.382449000	2.440136000	-0.000507000
C	-4.442741000	2.431165000	0.000789000
C	4.393492000	0.000006000	0.000032000
C	5.784832000	0.000011000	0.000388000
C	6.418160000	1.274657000	0.000047000
C	5.658390000	2.421977000	-0.000642000
C	4.235983000	2.395165000	-0.000986000
C	3.598074000	1.179380000	-0.000582000
C	3.598077000	-1.179367000	0.000157000
C	4.235991000	-2.395149000	0.000826000
C	5.658400000	-2.421955000	0.001311000
C	6.418166000	-1.274631000	0.001086000
H	-0.925038000	-0.000042000	-0.000321000
H	-3.384931000	-5.082085000	-0.000584000
H	-0.683438000	-5.085477000	-0.000149000
H	1.132045000	-3.222434000	-0.000071000
H	-7.119998000	1.346629000	0.000952000
H	-7.119976000	-1.346659000	-0.000112000
H	-3.112695000	0.000025000	0.000192000
H	-5.199887000	-3.206630000	-0.000706000
H	-0.683447000	5.085456000	0.000113000
H	-3.384948000	5.082085000	0.000810000
H	1.132025000	3.222406000	-0.000701000
H	-5.199911000	3.206630000	0.001151000
H	7.500789000	1.337832000	0.000303000
H	6.155428000	3.384796000	-0.000942000
H	3.688343000	3.330106000	-0.001526000
H	3.688360000	-3.330096000	0.000977000
H	6.155444000	-3.384770000	0.001861000
H	7.500795000	-1.337800000	0.001428000

ANPb

C	2.216164000	-0.685108000	-0.000457000
C	2.216129000	0.685138000	-0.000177000
C	0.824273000	1.092478000	0.000021000
N	0.011553000	-0.000072000	-0.000178000
C	0.824431000	-1.092631000	-0.000324000
C	-2.716275000	-4.237616000	0.000617000
C	-1.351412000	-4.239450000	0.000306000
C	-0.909504000	-2.875865000	-0.000036000
N	-2.030761000	-2.096682000	0.000122000
C	-3.154421000	-2.873144000	0.000416000
C	0.401086000	-2.421023000	-0.000271000
C	-6.272955000	0.674736000	-0.000219000
C	-6.272782000	-0.674984000	-0.000054000

C	-4.870716000	-1.077974000	0.000282000
N	-4.049691000	0.000107000	0.000195000
C	-4.871079000	1.078080000	-0.000238000
C	-4.463297000	-2.411466000	0.000486000
C	-1.351651000	4.239340000	-0.000042000
C	-2.716489000	4.237510000	-0.000299000
C	-3.154626000	2.873084000	-0.000291000
N	-2.030974000	2.096593000	-0.000041000
C	-0.909696000	2.875816000	0.000134000
C	0.400945000	2.420843000	0.000147000
C	-4.463612000	2.411463000	-0.000418000
C	4.397524000	0.000058000	0.000060000
C	5.787684000	0.000117000	0.000171000
C	6.421623000	1.275109000	0.000566000
C	5.660867000	2.421350000	0.000751000
C	4.237699000	2.393157000	0.000525000
C	3.598234000	1.178059000	0.000153000
C	3.598313000	-1.177983000	-0.000344000
C	4.237862000	-2.393037000	-0.000782000
C	5.661027000	-2.421124000	-0.000658000
C	6.421708000	-1.274828000	-0.000178000
H	-3.381556000	-5.087421000	0.000933000
H	-0.688482000	-5.091091000	0.000297000
H	-2.029429000	-1.082290000	-0.000129000
H	1.169068000	-3.185264000	-0.000360000
H	-7.115851000	1.350444000	-0.000440000
H	-7.115538000	-1.350879000	-0.000062000
H	-5.238002000	-3.169390000	0.000674000
H	-0.688742000	5.091004000	0.000052000
H	-3.381703000	5.087373000	-0.000513000
H	-2.030076000	1.082217000	0.000054000
H	1.169114000	3.184899000	0.000257000
H	-5.238260000	3.169462000	-0.000649000
H	7.504274000	1.338790000	0.000723000
H	6.157017000	3.384756000	0.001070000
H	3.691546000	3.329263000	0.000606000
H	3.691786000	-3.329187000	-0.001225000
H	6.157247000	-3.384494000	-0.000967000
H	7.504363000	-1.338446000	-0.000082000

ANPc

C	-2.271691000	0.749893000	-0.001377000
C	-2.183084000	-0.618776000	-0.001649000
C	-0.765051000	-0.944533000	-0.001306000
N	-0.032640000	0.196568000	-0.001002000
C	-0.907190000	1.237627000	-0.001004000
C	2.705329000	4.227210000	0.000034000
C	1.343850000	4.318729000	-0.000377000
C	0.819831000	2.988285000	-0.000845000
N	1.891656000	2.132223000	-0.000676000
C	3.063672000	2.837620000	-0.000121000
C	-0.504875000	2.574964000	-0.000944000
C	6.303072000	-0.685599000	0.001349000
C	6.226725000	0.677203000	0.001022000
C	4.840941000	1.050456000	0.000636000
N	4.123765000	-0.113106000	0.000726000
C	4.966766000	-1.194749000	0.001113000
C	4.368275000	2.357550000	0.000231000
C	1.340720000	-4.155865000	-0.000741000
C	2.687455000	-4.254201000	-0.000054000
C	3.182578000	-2.882907000	0.000313000
N	2.164070000	-1.992160000	-0.000119000
C	1.030040000	-2.725438000	-0.000854000
C	-0.282284000	-2.254228000	-0.001507000
C	4.529881000	-2.512492000	0.000893000
C	-4.405148000	-0.072141000	0.000280000
C	-5.792516000	-0.159095000	0.001130000
C	-6.344935000	-1.471477000	0.001398000
C	-5.513211000	-2.567432000	0.000703000
C	-4.094591000	-2.449920000	-0.000337000
C	-3.532503000	-1.197047000	-0.000529000
C	-3.681684000	1.153883000	-0.000173000

C	-4.395360000	2.326689000	0.000201000
C	-5.817564000	2.265714000	0.001197000
C	-6.504993000	1.073933000	0.001658000
H	3.423941000	5.032585000	0.000470000
H	0.739041000	5.212471000	-0.000515000
H	1.700560000	1.126087000	-0.000918000
H	-1.264927000	3.346385000	-0.001091000
H	7.190257000	-1.299996000	0.001817000
H	7.040265000	1.386563000	0.001141000
H	3.115391000	-0.292436000	0.000283000
H	5.136118000	3.121927000	0.000253000
H	0.608674000	-4.950621000	-0.001074000
H	3.300717000	-5.143433000	0.000266000
H	-1.046893000	-3.024654000	-0.002132000
H	5.284659000	-3.289472000	0.001139000
H	-7.421426000	-1.603372000	0.002108000
H	-5.947638000	-3.560214000	0.000894000
H	-3.490916000	-3.349924000	-0.000932000
H	-3.907571000	3.294574000	-0.000201000
H	-6.373401000	3.195994000	0.001569000
H	-7.589529000	1.069726000	0.002381000

ANPd

C	2.167658000	-0.628004000	0.001737000
C	2.254652000	0.755932000	0.001394000
C	0.931395000	1.279148000	0.000512000
N	0.086872000	0.187349000	0.000267000
C	0.788296000	-0.995982000	0.001075000
C	-2.755770000	-4.219552000	-0.000338000
C	-1.393253000	-4.143485000	-0.000369000
C	-1.019154000	-2.757529000	-0.000223000
N	-2.182331000	-2.039737000	-0.000208000
C	-3.264788000	-2.883346000	-0.000495000
C	0.291473000	-2.293639000	0.000430000
C	-6.232761000	0.736744000	-0.000420000
C	-6.330801000	-0.610506000	-0.000106000
C	-4.959395000	-1.105007000	-0.000693000
N	-4.069632000	-0.086332000	-0.000948000
C	-4.802759000	1.047206000	-0.000615000
C	-4.583751000	-2.450947000	-0.000570000
C	-1.343443000	4.343393000	0.000395000
C	-2.690774000	4.252537000	0.000343000
C	-3.008840000	2.824021000	0.000267000
N	-1.879349000	2.083968000	0.000870000
C	-0.856166000	2.969109000	0.000509000
C	0.489519000	2.594464000	0.000688000
C	-4.324317000	2.357836000	-0.000332000
C	4.385221000	-0.072040000	-0.000047000
C	5.774061000	-0.157244000	-0.000836000
C	6.483902000	1.076465000	-0.001783000
C	5.796112000	2.268442000	-0.001707000
C	4.374954000	2.328870000	-0.000651000
C	3.664665000	1.154085000	0.000148000
C	3.517712000	-1.199915000	0.000949000
C	4.080951000	-2.452286000	0.001337000
C	5.499032000	-2.566359000	0.000443000
C	6.328122000	-1.468242000	-0.000647000
H	-0.920318000	0.367152000	0.001618000
H	-3.370650000	-5.106409000	-0.000033000
H	-0.683892000	-4.956997000	-0.000177000
H	-2.361081000	-1.031110000	-0.001037000
H	1.046772000	-3.069643000	0.000742000
H	-7.027750000	1.468537000	-0.000284000
H	-7.220170000	-1.223607000	0.000229000
H	-5.357447000	-3.209141000	-0.000414000
H	-0.725322000	5.229288000	0.000387000
H	-3.418615000	5.051151000	0.000226000
H	1.243171000	3.372138000	0.000924000
H	-5.088296000	3.129507000	-0.000593000
H	7.568372000	1.073250000	-0.002557000
H	6.351574000	3.198759000	-0.002443000
H	3.884283000	3.294940000	-0.000472000

H	3.479443000	-3.353517000	0.002339000
H	5.935721000	-3.558012000	0.000632000
H	7.404769000	-1.598032000	-0.001337000

MeANPa

C	2.206410000	-0.694550000	0.477173000
C	2.206396000	0.694655000	0.477088000
C	0.886902000	1.135809000	0.762884000
N	0.122175000	0.000079000	0.993091000
C	0.886939000	-1.135705000	0.762996000
C	-2.587931000	-4.280728000	0.029055000
C	-1.288077000	-4.269920000	0.393134000
C	-0.899217000	-2.860503000	0.470259000
N	-1.941694000	-2.055772000	0.160058000
C	-2.971468000	-2.877650000	-0.119577000
C	0.410234000	-2.443845000	0.715944000
C	-5.967826000	0.681321000	-1.005175000
C	-5.967794000	-0.681479000	-1.005259000
C	-4.646858000	-1.124607000	-0.663411000
N	-3.887045000	-0.000054000	-0.475771000
C	-4.646890000	1.124449000	-0.663288000
C	-4.239006000	-2.443603000	-0.515418000
C	-1.288265000	4.269943000	0.393259000
C	-2.588158000	4.280665000	0.029330000
C	-2.971591000	2.877561000	-0.119384000
N	-1.941701000	2.055748000	0.160012000
C	-0.899274000	2.860553000	0.470225000
C	0.410212000	2.443955000	0.715823000
C	-4.239130000	2.443476000	-0.515183000
C	4.271999000	0.000030000	-0.217570000
C	5.593371000	0.000005000	-0.654109000
C	6.191327000	1.274922000	-0.861261000
C	5.465866000	2.421938000	-0.633673000
C	4.114912000	2.395115000	-0.188626000
C	3.514083000	1.179134000	0.024733000
C	3.514117000	-1.179056000	0.024931000
C	4.114995000	-2.395064000	-0.188126000
C	5.465962000	-2.421934000	-0.633134000
C	6.191376000	-1.274938000	-0.860968000
C	-0.974164000	0.000075000	1.969735000
H	-3.234924000	-5.129864000	-0.137183000
H	-0.637400000	-5.109261000	0.592425000
H	1.165452000	-3.221285000	0.756648000
H	-6.794034000	1.345945000	-1.206811000
H	-6.793986000	-1.346105000	-1.206961000
H	-2.887492000	-0.000034000	-0.280187000
H	-4.990662000	-3.198544000	-0.715458000
H	-0.637646000	5.109322000	0.592581000
H	-3.235250000	5.129757000	-0.136741000
H	1.165441000	3.221378000	0.756516000
H	-4.990861000	3.198369000	-0.715114000
H	7.218085000	1.338628000	-1.204475000
H	5.934303000	3.384707000	-0.800451000
H	3.589697000	3.329617000	-0.031051000
H	3.589797000	-3.329543000	-0.030361000
H	5.934446000	-3.384720000	-0.799686000
H	7.218141000	-1.338682000	-1.204154000
H	-0.872737000	0.891874000	2.587798000
H	-1.946915000	-0.000301000	1.498287000
H	-0.872291000	-0.891383000	2.588219000

MeANPb

C	2.199110000	-0.698281000	0.431185000
C	2.234704000	0.690515000	0.449866000
C	0.923265000	1.168762000	0.708268000
N	0.119242000	0.044839000	0.908914000
C	0.864975000	-1.107843000	0.686608000
C	-2.708768000	-4.216739000	0.087762000
C	-1.383429000	-4.195222000	0.407604000
C	-0.945661000	-2.826914000	0.419089000
N	-2.037791000	-2.066319000	0.098516000
C	-3.133781000	-2.863780000	-0.113984000

C	0.362769000	-2.406470000	0.641167000
C	-5.994264000	0.766736000	-0.959830000
C	-6.082851000	-0.580687000	-0.951241000
C	-4.752419000	-1.059404000	-0.589624000
N	-3.895972000	-0.036471000	-0.388889000
C	-4.612726000	1.093058000	-0.601391000
C	-4.398240000	-2.406980000	-0.452968000
C	-1.237485000	4.323721000	0.327788000
C	-2.542424000	4.292073000	-0.019502000
C	-2.894899000	2.872751000	-0.139997000
N	-1.836801000	2.091271000	0.128862000
C	-0.818047000	2.922807000	0.416681000
C	0.489166000	2.489340000	0.660113000
C	-4.167597000	2.411062000	-0.490977000
C	4.298925000	-0.050948000	-0.201114000
C	5.630568000	-0.080667000	-0.604331000
C	6.267203000	1.180333000	-0.776914000
C	5.567283000	2.343585000	-0.550663000
C	4.205232000	2.347174000	-0.141028000
C	3.567071000	1.144691000	0.038764000
C	3.503983000	-1.212503000	0.005431000
C	4.077667000	-2.441007000	-0.210781000
C	5.438342000	-2.498129000	-0.622634000
C	6.199694000	-1.368266000	-0.815166000
C	-0.988457000	0.057688000	1.879338000
H	-3.355104000	-5.076402000	-0.002400000
H	-0.740614000	-5.033964000	0.627573000
H	-2.095018000	-1.059078000	-0.060200000
H	1.098950000	-3.199798000	0.693068000
H	-6.766721000	1.489914000	-1.179091000
H	-6.940027000	-1.203464000	-1.162091000
H	-5.162400000	-3.158228000	-0.614014000
H	-0.608817000	5.184024000	0.507252000
H	-3.213193000	5.122766000	-0.186450000
H	1.265426000	3.245633000	0.702395000
H	-4.911930000	3.175394000	-0.693844000
H	7.303886000	1.221182000	-1.092676000
H	6.065822000	3.295398000	-0.690631000
H	3.701388000	3.293190000	0.017274000
H	3.524546000	-3.363530000	-0.080113000
H	5.884930000	-3.470808000	-0.791811000
H	7.232758000	-1.454752000	-1.133459000
H	-0.820453000	0.885798000	2.568305000
H	-1.953080000	0.212395000	1.402617000
H	-0.964541000	-0.881655000	2.431351000

ANPaH^{+}

C	-2.220799000	-0.685344000	0.049085000
C	-2.220839000	0.685379000	0.049173000
C	-0.828721000	1.091600000	0.025504000
N	-0.004814000	0.000072000	0.032743000
C	-0.828720000	-1.091528000	0.025392000
C	2.691516000	-4.236111000	-0.244797000
C	1.327995000	-4.247103000	-0.259388000
C	0.877898000	-2.895401000	-0.118206000
N	1.992775000	-2.106605000	-0.043005000
C	3.127150000	-2.878230000	-0.097219000
C	-0.424716000	-2.424769000	-0.054333000
C	6.196336000	0.685087000	0.419223000
C	6.196380000	-0.684979000	0.419395000
C	4.888127000	-1.130482000	0.085696000
N	4.105468000	-0.000068000	-0.091149000
C	4.888038000	1.130451000	0.085518000
C	4.441301000	-2.443006000	0.000396000
C	1.327949000	4.247236000	-0.259228000
C	2.691453000	4.236130000	-0.244946000
C	3.127022000	2.878201000	-0.097598000
N	1.992589000	2.106676000	-0.043221000
C	0.877759000	2.895560000	-0.118190000
C	-0.424835000	2.424888000	-0.054225000
C	4.441173000	2.442919000	-0.000146000
C	-4.392348000	-0.000042000	0.051708000

C	-5.783698000	-0.000071000	0.058281000
C	-6.417290000	1.273245000	0.061907000
C	-5.659123000	2.423325000	0.061963000
C	-4.239151000	2.396349000	0.057986000
C	-3.598703000	1.179091000	0.051051000
C	-3.598651000	-1.179134000	0.050864000
C	-4.239041000	-2.396420000	0.057595000
C	-5.659013000	-2.423462000	0.061564000
C	-6.417233000	-1.273419000	0.061701000
H	3.361328000	-5.077528000	-0.336169000
H	0.674317000	-5.099643000	-0.361454000
H	1.898388000	-1.130258000	0.206030000
H	-1.206347000	-3.173536000	-0.093108000
H	7.016495000	1.342334000	0.665119000
H	7.016610000	-1.342114000	0.665348000
H	3.334061000	-0.000182000	-0.748202000
H	5.199914000	-3.214574000	0.048055000
H	0.674342000	5.099871000	-0.360977000
H	3.361273000	5.077540000	-0.336323000
H	1.898152000	1.130196000	0.205237000
H	-1.206555000	3.173562000	-0.092968000
H	5.199833000	3.214465000	0.047182000
H	-7.499722000	1.336209000	0.065971000
H	-6.157227000	3.385010000	0.066649000
H	-3.696846000	3.334713000	0.063199000
H	-3.696696000	-3.334763000	0.062633000
H	-6.157071000	-3.385172000	0.066088000
H	-7.499663000	-1.336433000	0.065754000

ANPbH$^+$

C	2.209682000	-0.698530000	0.229304000
C	2.209525000	0.697974000	0.229982000
C	0.873728000	1.145449000	0.369556000
N	0.068410000	-0.000575000	0.419037000
C	0.874010000	-1.146448000	0.368537000
C	-2.721176000	-4.244155000	0.222780000
C	-1.368729000	-4.236585000	0.405593000
C	-0.913913000	-2.880897000	0.318234000
N	-2.023215000	-2.107373000	0.100530000
C	-3.142099000	-2.892648000	0.018175000
C	0.406376000	-2.449573000	0.403607000
C	-6.183696000	0.673747000	-0.559562000
C	-6.183706000	-0.673379000	-0.558969000
C	-4.797723000	-1.077409000	-0.350744000
N	-3.976632000	0.000370000	-0.245194000
C	-4.797714000	1.077886000	-0.351672000
C	-4.421543000	-2.415803000	-0.223136000
C	-1.368331000	4.236207000	0.404889000
C	-2.720484000	4.244507000	0.220713000
C	-3.142100000	2.893016000	0.016929000
N	-2.023713000	2.107192000	0.100690000
C	-0.914220000	2.880035000	0.319089000
C	0.405904000	2.448561000	0.405220000
C	-4.421455000	2.416458000	-0.224802000
C	4.351967000	0.000095000	-0.102216000
C	5.729266000	0.000337000	-0.311225000
C	6.353447000	1.273587000	-0.410427000
C	5.603839000	2.425141000	-0.303200000
C	4.201046000	2.399484000	-0.091731000
C	3.571482000	1.180651000	0.012470000
C	3.571727000	-1.180726000	0.011315000
C	4.201513000	-2.399340000	-0.094183000
C	5.604297000	-2.424507000	-0.305709000
C	6.353682000	-1.272688000	-0.411745000
H	-0.737296000	-0.001058000	1.036487000
H	-3.385214000	-5.094821000	0.230420000
H	-0.721785000	-5.079815000	0.593096000
H	-2.069881000	-1.126987000	-0.145874000
H	1.156000000	-3.227648000	0.473638000
H	-7.017512000	1.350212000	-0.675146000
H	-7.017547000	-1.349920000	-0.673954000
H	-5.205928000	-3.159810000	-0.296846000

H	-0.720919000	5.079121000	0.592205000
H	-3.384034000	5.095568000	0.226787000
H	-2.071619000	1.126016000	-0.142302000
H	1.155510000	3.226586000	0.475920000
H	-5.205744000	3.160496000	-0.299222000
H	7.423358000	1.336598000	-0.574400000
H	6.096746000	3.385969000	-0.384008000
H	3.661324000	3.336186000	-0.018979000
H	3.661964000	-3.336214000	-0.022377000
H	6.097398000	-3.385149000	-0.387528000
H	7.423604000	-1.335327000	-0.575789000

ANPcH^+

C	-2.238744000	0.703956000	-0.080580000
C	-2.236990000	-0.687290000	-0.068747000
C	-0.885325000	-1.133506000	-0.139143000
N	-0.105524000	0.008120000	-0.162076000
C	-0.886717000	1.145318000	-0.159318000
C	2.703903000	4.226833000	-0.186774000
C	1.360051000	4.219780000	-0.266006000
C	0.959122000	2.815976000	-0.235639000
N	2.035851000	1.993943000	-0.157842000
C	3.111327000	2.827816000	-0.107665000
C	-0.385412000	2.433893000	-0.229666000
C	6.235242000	-0.662286000	0.457285000
C	6.252099000	0.702644000	0.424026000
C	4.913866000	1.149055000	0.193549000
N	4.125679000	0.034520000	0.112514000
C	4.886954000	-1.097841000	0.249416000
C	4.441856000	2.448571000	0.059561000
C	1.369027000	-4.188820000	-0.448150000
C	2.733095000	-4.182992000	-0.338035000
C	3.148805000	-2.863694000	-0.002689000
N	2.004010000	-2.083020000	0.066626000
C	0.893882000	-2.874201000	-0.179687000
C	-0.425890000	-2.441591000	-0.195590000
C	4.450674000	-2.414858000	0.161522000
C	-4.400874000	0.006494000	0.073838000
C	-5.790247000	0.006243000	0.168622000
C	-6.420148000	-1.266762000	0.228584000
C	-5.663159000	-2.417775000	0.195002000
C	-4.247362000	-2.391698000	0.099825000
C	-3.611528000	-1.173490000	0.035564000
C	-3.614353000	1.187065000	0.010586000
C	-4.251829000	2.405588000	0.045650000
C	-5.667549000	2.430966000	0.140160000
C	-6.422214000	1.279392000	0.200117000
H	0.869986000	0.077322000	-0.420340000
H	3.376657000	5.071398000	-0.168873000
H	0.681867000	5.057860000	-0.328382000
H	-1.118910000	3.230300000	-0.261332000
H	7.067772000	-1.330403000	0.616653000
H	7.101150000	1.356978000	0.548400000
H	3.158575000	0.126234000	-0.172899000
H	5.181176000	3.238840000	0.112875000
H	0.735260000	-5.017321000	-0.725435000
H	3.410325000	-5.005570000	-0.511028000
H	1.946157000	-1.288360000	0.692689000
H	-1.180855000	-3.211611000	-0.290809000
H	5.221223000	-3.175761000	0.184830000
H	-7.499950000	-1.329743000	0.303495000
H	-6.159881000	-3.378791000	0.244554000
H	-3.703462000	-3.328779000	0.082992000
H	-3.708143000	3.341925000	0.004195000
H	-6.166277000	3.391838000	0.166872000
H	-7.502171000	1.341984000	0.273294000

MeANPaH^+

C	-2.272656000	-0.723513000	0.414694000
C	-2.191901000	0.671738000	0.374614000
C	-0.846088000	1.049247000	0.608322000
N	-0.109865000	-0.124887000	0.833951000

C	-0.975064000	-1.224949000	0.662780000
C	2.587541000	-4.237701000	-0.027566000
C	1.269866000	-4.296137000	0.324961000
C	0.763931000	-2.960075000	0.364630000
N	1.811173000	-2.130551000	0.037960000
C	2.942923000	-2.864006000	-0.217925000
C	-0.541726000	-2.544476000	0.611370000
C	6.094615000	0.663671000	-0.898217000
C	6.008902000	-0.696333000	-0.958478000
C	4.665358000	-1.078170000	-0.628374000
N	3.979706000	0.080630000	-0.381743000
C	4.805130000	1.162194000	-0.531239000
C	4.204296000	-2.388026000	-0.550661000
C	1.337481000	4.192760000	0.386073000
C	2.658657000	4.249275000	0.127151000
C	3.097848000	2.864074000	-0.011222000
N	2.069270000	1.999360000	0.184731000
C	0.985428000	2.772212000	0.419423000
C	-0.335951000	2.339531000	0.578677000
C	4.395182000	2.478231000	-0.346594000
C	0.766730000	-0.129836000	2.031900000
C	-4.312968000	0.075203000	-0.210327000
C	-5.652786000	0.140548000	-0.586940000
C	-6.185689000	1.440839000	-0.801112000
C	-5.389774000	2.553993000	-0.636147000
C	-4.027500000	2.461881000	-0.250523000
C	-3.485857000	1.215880000	-0.032772000
C	-3.620400000	-1.140308000	0.032190000
C	-4.300990000	-2.326852000	-0.112240000
C	-5.666702000	-2.285777000	-0.495395000
C	-6.331114000	-1.100783000	-0.727228000
H	3.278139000	-5.059879000	-0.137708000
H	0.685778000	-5.174128000	0.554143000
H	1.658993000	-1.152440000	-0.171590000
H	-1.294277000	-3.320777000	0.671225000
H	6.958706000	1.282078000	-1.086189000
H	6.790749000	-1.398286000	-1.205374000
H	3.013020000	0.251977000	-0.111485000
H	4.946774000	-3.150837000	-0.752326000
H	0.646868000	5.008176000	0.543306000
H	3.290142000	5.118949000	0.021979000
H	-1.084472000	3.124378000	0.586242000
H	5.139600000	3.251948000	-0.490501000
H	0.185682000	0.189384000	2.900027000
H	1.135932000	-1.136062000	2.208309000
H	1.602823000	0.547618000	1.878193000
H	-7.221928000	1.554816000	-1.099183000
H	-5.813151000	3.536072000	-0.806494000
H	-3.448431000	3.370623000	-0.137869000
H	-3.828389000	-3.287030000	0.057449000
H	-6.200140000	-3.221417000	-0.608410000
H	-7.374733000	-1.113052000	-1.020994000

MeANPbH^+

C	-2.218224000	-0.696898000	0.379016000
C	-2.218278000	0.696984000	0.379047000
C	-0.896526000	1.140241000	0.625519000
N	-0.090822000	0.000078000	0.817583000
C	-0.896456000	-1.140069000	0.625520000
C	2.650956000	-4.256661000	0.133455000
C	1.325202000	-4.250911000	0.457209000
C	0.866629000	-2.892170000	0.431166000
N	1.944726000	-2.122131000	0.097148000
C	3.048895000	-2.902039000	-0.105931000
C	-0.441209000	-2.449646000	0.617990000
C	6.010586000	0.673634000	-0.985370000
C	6.010568000	-0.673718000	-0.985340000
C	4.655994000	-1.078231000	-0.627455000
N	3.852213000	-0.000053000	-0.428156000
C	4.656041000	1.078239000	-0.627519000
C	4.295952000	-2.419021000	-0.472713000
C	1.325258000	4.250919000	0.457124000

C	2.651077000	4.256593000	0.133405000
C	3.048879000	2.902014000	-0.106025000
N	1.944686000	2.122161000	0.096959000
C	0.866626000	2.892273000	0.431005000
C	-0.441263000	2.449774000	0.617856000
C	4.295988000	2.418913000	-0.472816000
C	0.895686000	0.000104000	1.928182000
C	-4.304687000	-0.000022000	-0.214162000
C	-5.647279000	-0.000060000	-0.585401000
C	-6.254449000	1.273379000	-0.760620000
C	-5.522035000	2.424633000	-0.567174000
C	-4.154283000	2.398992000	-0.189771000
C	-3.542300000	1.180340000	-0.008994000
C	-3.542217000	-1.180342000	-0.009060000
C	-4.154092000	-2.399022000	-0.189965000
C	-5.521844000	-2.424741000	-0.567398000
C	-6.254349000	-1.273536000	-0.760741000
H	3.311714000	-5.107258000	0.065613000
H	0.700639000	-5.096124000	0.702753000
H	1.954263000	-1.133497000	-0.117408000
H	-1.197753000	-3.222588000	0.678373000
H	6.826659000	1.349807000	-1.192872000
H	6.826608000	-1.349928000	-1.192842000
H	5.069834000	-3.159978000	-0.635301000
H	0.700735000	5.096184000	0.702590000
H	3.311890000	5.107151000	0.065637000
H	1.954168000	1.133514000	-0.117536000
H	-1.197775000	3.222755000	0.678190000
H	5.069830000	3.159904000	-0.635464000
H	0.731239000	0.887109000	2.538426000
H	0.731167000	-0.886809000	2.538538000
H	1.931005000	0.000061000	1.586961000
H	-7.296928000	1.336685000	-1.051826000
H	-6.001183000	3.385517000	-0.708092000
H	-3.626663000	3.335725000	-0.055036000
H	-3.626405000	-3.335728000	-0.055305000
H	-6.000904000	-3.385655000	-0.708416000
H	-7.296822000	-1.336892000	-1.051959000

ANPH_2^{2+}

C	-2.224985000	0.704777000	-0.163832000
C	-2.224985000	-0.704777000	-0.163831000
C	-0.893129000	-1.144752000	-0.337689000
N	-0.072491000	0.000000000	-0.367895000
C	-0.893130000	1.144751000	-0.337690000
C	2.747043000	4.170834000	-0.551388000
C	1.396949000	4.168872000	-0.766530000
C	0.902169000	2.862029000	-0.477020000
N	1.990212000	2.078695000	-0.139096000
C	3.132602000	2.863675000	-0.127986000
C	-0.424936000	2.443037000	-0.485365000
C	6.034988000	-0.684959000	1.042282000
C	6.034988000	0.684959000	1.042282000
C	4.799192000	1.128719000	0.492177000
N	4.045618000	0.000000000	0.210123000
C	4.799192000	-1.128718000	0.492176000
C	4.400209000	2.439196000	0.246766000
C	1.396949000	-4.168871000	-0.766531000
C	2.747044000	-4.170834000	-0.551390000
C	3.132602000	-2.863674000	-0.127987000
N	1.990212000	-2.078695000	-0.139096000
C	0.902169000	-2.862029000	-0.477019000
C	-0.424936000	-2.443038000	-0.485363000
C	4.400210000	-2.439195000	0.246764000
C	-4.353958000	0.000000000	0.168092000
C	-5.732437000	0.000000000	0.378796000
C	-6.356750000	-1.271115000	0.479262000
C	-5.608660000	-2.429172000	0.374081000
C	-4.211853000	-2.405794000	0.163799000
C	-3.577552000	-1.182330000	0.054486000

C	-3.577552000	1.182330000	0.054485000
C	-4.211853000	2.405794000	0.163797000
C	-5.608660000	2.429172000	0.374079000
C	-6.356750000	1.271115000	0.479261000
H	0.690141000	0.000000000	-1.039852000
H	3.434029000	4.992493000	-0.690688000
H	0.785365000	4.989558000	-1.110811000
H	1.884033000	1.284010000	0.479608000
H	-1.171649000	3.216297000	-0.619116000
H	6.810534000	-1.341327000	1.408479000
H	6.810534000	1.341327000	1.408480000
H	3.383290000	0.000001000	-0.556718000
H	5.156797000	3.207019000	0.358892000
H	0.785365000	-4.989558000	-1.110812000
H	3.434029000	-4.992493000	-0.690691000
H	1.884034000	-1.284010000	0.479608000
H	-1.171649000	-3.216298000	-0.619113000
H	5.156797000	-3.207018000	0.358890000
H	-7.426781000	-1.333457000	0.643603000
H	-6.105117000	-3.387569000	0.457930000
H	-3.673896000	-3.344105000	0.097373000
H	-3.673896000	3.344105000	0.097370000
H	-6.105118000	3.387569000	0.457926000
H	-7.426781000	1.333457000	0.643602000

MeANPH_2^{2+}

C	-2.222432000	-0.706453000	0.288727000
C	-2.222497000	0.706503000	0.288745000
C	-0.906065000	1.142292000	0.535021000
N	-0.074413000	0.000105000	0.723379000
C	-0.905964000	-1.142126000	0.534994000
C	2.682876000	-4.233597000	0.313245000
C	1.347875000	-4.240435000	0.600759000
C	0.858205000	-2.903078000	0.464156000
N	1.933635000	-2.115675000	0.122526000
C	3.061494000	-2.892886000	-0.015545000
C	-0.455019000	-2.457321000	0.579474000
C	5.899805000	0.684531000	-1.270256000
C	5.899817000	-0.684492000	-1.270338000
C	4.702013000	-1.130168000	-0.643374000
N	3.969299000	-0.000055000	-0.300311000
C	4.701985000	1.130112000	-0.643248000
C	4.311729000	-2.447022000	-0.426973000
C	1.347790000	4.240602000	0.600493000
C	2.682796000	4.233645000	0.313085000
C	3.061373000	2.892837000	-0.015485000
N	1.933436000	2.115824000	0.122451000
C	0.858072000	2.903214000	0.464054000
C	-0.455170000	2.457494000	0.579439000
C	4.311637000	2.446938000	-0.426812000
C	0.638972000	0.000075000	2.036729000
C	-4.308897000	-0.000060000	-0.253293000
C	-5.662166000	-0.000109000	-0.591151000
C	-6.274080000	1.271212000	-0.749176000
C	-5.539614000	2.429138000	-0.572768000
C	-4.168855000	2.405903000	-0.231413000
C	-3.546793000	1.182634000	-0.066353000
C	-3.546689000	-1.182693000	-0.066392000
C	-4.168657000	-2.406005000	-0.231481000
C	-5.539414000	-2.429341000	-0.572842000
C	-6.273977000	-1.271473000	-0.749216000
H	3.365593000	-5.070044000	0.334131000
H	0.743164000	-5.084296000	0.897544000
H	1.807037000	-1.208318000	-0.304870000
H	-1.213898000	-3.227491000	0.652918000
H	6.645580000	1.341050000	-1.693624000
H	6.645615000	-1.340949000	-1.693763000
H	3.390214000	-0.000154000	0.532821000
H	5.056797000	-3.209522000	-0.621710000
H	0.743058000	5.084527000	0.897046000

H	3.365558000	5.070058000	0.333781000
H	1.807007000	1.207806000	-0.303540000
H	-1.214042000	3.227669000	0.652862000
H	5.056695000	3.209466000	-0.621489000
H	1.260825000	0.887612000	2.121471000
H	-0.092414000	-0.000827000	2.848705000
H	1.262209000	-0.886557000	2.120713000
H	-7.323955000	1.333735000	-1.013264000
H	-6.026739000	3.387495000	-0.700452000
H	-3.641431000	3.344492000	-0.109225000
H	-3.641164000	-3.344560000	-0.109328000
H	-6.026458000	-3.387735000	-0.700561000
H	-7.323846000	-1.334077000	-1.013309000

PhPa

C	2.070449000	-0.689551000	0.058451000
C	2.070487000	0.689676000	-0.058567000
C	0.689894000	1.133425000	0.006285000
N	-0.076118000	0.000081000	-0.000112000
C	0.689875000	-1.133272000	-0.006327000
C	-2.894758000	-4.173146000	-0.613761000
C	-1.546794000	-4.169329000	-0.555498000
C	-1.156435000	-2.783118000	-0.314476000
N	-2.230489000	-1.974907000	-0.234189000
C	-3.310015000	-2.791647000	-0.408524000
C	0.189963000	-2.409628000	-0.180366000
C	-6.476283000	0.675774000	0.112868000
C	-6.476288000	-0.675710000	-0.113343000
C	-5.116981000	-1.105115000	-0.181378000
N	-4.339120000	-0.000013000	0.000019000
C	-5.116971000	1.105122000	0.181224000
C	-4.639938000	-2.399693000	-0.382688000
C	-1.546837000	4.169361000	0.555881000
C	-2.894801000	4.173120000	0.614172000
C	-3.310022000	2.791668000	0.408572000
N	-2.230465000	1.974977000	0.234171000
C	-1.156432000	2.783181000	0.314775000
C	0.189973000	2.409733000	0.180652000
C	-4.639940000	2.399691000	0.382635000
C	3.296225000	-1.420480000	0.282012000
C	4.518461000	-0.709957000	0.180244000
C	4.518537000	0.709952000	-0.180027000
C	3.296340000	1.420526000	-0.282147000
C	3.319181000	-2.764195000	0.708480000
C	4.506550000	-3.413734000	0.962767000
C	5.717901000	-2.728611000	0.819283000
C	5.715775000	-1.400452000	0.452268000
C	5.715954000	1.400332000	-0.451888000
C	5.718235000	2.728406000	-0.819196000
C	4.506949000	3.413525000	-0.963209000
C	3.319484000	2.764114000	-0.709048000
H	-1.092665000	0.000064000	-0.000134000
H	-3.562720000	-5.006434000	-0.776501000
H	-0.863380000	-4.999654000	-0.659266000
H	0.905811000	-3.210227000	-0.286466000
H	-7.327615000	1.330087000	0.221365000
H	-7.327626000	-1.329990000	-0.221980000
H	-3.323242000	-0.000032000	0.000128000
H	-5.389754000	-3.169786000	-0.523136000
H	-0.863451000	4.999691000	0.659791000
H	-3.562789000	5.006372000	0.776991000
H	0.905816000	3.210276000	0.287111000
H	-5.389774000	3.169762000	0.523114000
H	2.391194000	-3.281837000	0.898163000
H	4.498091000	-4.443491000	1.298915000
H	6.655837000	-3.229491000	1.026363000
H	6.663280000	-0.882964000	0.399373000
H	6.663422000	0.882807000	-0.398688000
H	6.656244000	3.229200000	-1.026151000
H	4.498629000	4.443165000	-1.299719000
H	2.391595000	3.281722000	-0.899247000

PhPb

C	2.070037000	-0.683323000	0.068444000
C	2.069989000	0.683437000	-0.067696000
C	0.657743000	1.087908000	0.002767000
N	-0.149804000	-0.000030000	0.000318000
C	0.657765000	-1.087827000	-0.002004000
C	-2.897583000	-4.170045000	-0.645581000
C	-1.534003000	-4.166921000	-0.587226000
C	-1.105268000	-2.821545000	-0.338682000
N	-2.231480000	-2.057336000	-0.259323000
C	-3.348893000	-2.827436000	-0.435263000
C	0.209356000	-2.394393000	-0.192802000
C	-6.475286000	0.665004000	0.117701000
C	-6.475248000	-0.665072000	-0.119085000
C	-5.074822000	-1.062167000	-0.184855000
N	-4.253993000	-0.000117000	-0.000050000
C	-5.074899000	1.062075000	0.184220000
C	-4.659869000	-2.376684000	-0.399524000
C	-1.534162000	4.166750000	0.588058000
C	-2.897829000	4.169858000	0.645906000
C	-3.348997000	2.827328000	0.435276000
N	-2.231543000	2.057258000	0.259670000
C	-1.105412000	2.821465000	0.339493000
C	0.209288000	2.394326000	0.193749000
C	-4.660005000	2.376511000	0.399005000
C	3.290325000	-1.412859000	0.306791000
C	4.515615000	-0.705596000	0.192209000
C	4.515515000	0.705725000	-0.192756000
C	3.290164000	1.413013000	-0.306575000
C	3.313506000	-2.748857000	0.762121000
C	4.499772000	-3.393838000	1.029327000
C	5.713057000	-2.713280000	0.870438000
C	5.712671000	-1.393245000	0.477177000
C	5.712423000	1.393352000	-0.478375000
C	5.712632000	2.713440000	-0.871468000
C	4.499273000	3.394060000	-1.029488000
C	3.313136000	2.749091000	-0.761668000
H	-3.553258000	-5.010066000	-0.816609000
H	-0.862487000	-5.004213000	-0.701044000
H	-2.246744000	-1.058574000	-0.090175000
H	0.948390000	-3.173520000	-0.306806000
H	-7.318460000	1.330125000	0.234882000
H	-7.318376000	-1.330172000	-0.236691000
H	-5.428924000	-3.126504000	-0.544601000
H	-0.862671000	5.004008000	0.702243000
H	-3.553601000	5.009811000	0.816881000
H	-2.246637000	1.058535000	0.090248000
H	0.948327000	3.173430000	0.308015000
H	-5.429110000	3.126355000	0.543738000
H	2.384470000	-3.260699000	0.965083000
H	4.491018000	-4.415977000	1.388377000
H	6.650150000	-3.211558000	1.087580000
H	6.661004000	-0.878089000	0.413741000
H	6.660761000	0.878131000	-0.415552000
H	6.649615000	3.211707000	-1.089107000
H	4.490301000	4.416263000	-1.388352000
H	2.384016000	3.261048000	-0.963975000

PhPc

C	2.135915000	-0.722059000	-0.064113000
C	2.036832000	0.643478000	0.057184000
C	0.595659000	0.952417000	-0.024108000
N	-0.122552000	-0.190646000	-0.017433000
C	0.753802000	-1.219321000	-0.010058000
C	-2.852226000	-4.186406000	0.558793000
C	-1.491721000	-4.260818000	0.497148000
C	-0.992423000	-2.937280000	0.285534000
N	-2.076328000	-2.101709000	0.226888000
C	-3.236882000	-2.815490000	0.387267000
C	0.333226000	-2.541779000	0.154909000
C	-6.522951000	0.626922000	-0.063677000
C	-6.434374000	-0.719930000	0.153920000

C	-5.047147000	-1.076010000	0.200959000
N	-4.341437000	0.078712000	0.016027000
C	-5.192366000	1.138541000	-0.150216000
C	-4.548935000	-2.362451000	0.378250000
C	-1.568491000	4.061091000	-0.622091000
C	-2.914714000	4.153127000	-0.653793000
C	-3.407350000	2.802843000	-0.415775000
N	-2.382550000	1.927902000	-0.246111000
C	-1.253507000	2.654770000	-0.360171000
C	0.072242000	2.228430000	-0.230078000
C	-4.752759000	2.440939000	-0.362714000
C	3.405623000	-1.369870000	-0.271615000
C	4.577345000	-0.575465000	-0.172185000
C	4.475811000	0.838729000	0.186503000
C	3.202559000	1.457364000	0.296671000
C	3.525432000	-2.717265000	-0.674073000
C	4.755033000	-3.285358000	-0.917759000
C	5.916192000	-2.513565000	-0.785630000
C	5.821094000	-1.185166000	-0.434892000
C	5.621205000	1.614054000	0.459594000
C	5.527525000	2.933253000	0.843526000
C	4.268441000	3.524056000	1.006363000
C	3.130959000	2.793780000	0.747434000
H	-3.554810000	-4.992248000	0.707282000
H	-0.870277000	-5.138746000	0.584962000
H	-1.906925000	-1.102587000	0.097042000
H	1.065226000	-3.329083000	0.252494000
H	-7.416038000	1.225132000	-0.158660000
H	-7.242016000	-1.426931000	0.267397000
H	-3.335099000	0.269246000	0.002400000
H	-5.299273000	-3.131767000	0.516351000
H	-0.837340000	4.845411000	-0.756228000
H	-3.530131000	5.025105000	-0.820206000
H	0.797326000	3.019399000	-0.364845000
H	-5.504745000	3.209268000	-0.494886000
H	2.635862000	-3.303414000	-0.850314000
H	4.820745000	-4.319040000	-1.235504000
H	6.886949000	-2.950821000	-0.985694000
H	6.730621000	-0.602964000	-0.384867000
H	6.604165000	1.168606000	0.395780000
H	6.426905000	3.500063000	1.052024000
H	4.186719000	4.544416000	1.361293000
H	2.167939000	3.237781000	0.951941000

PhPd

C	-2.020314000	-0.644452000	-0.067690000
C	-2.119244000	0.732155000	0.047115000
C	-0.775838000	1.267915000	-0.007565000
N	0.072523000	0.188891000	0.001736000
C	-0.612565000	-0.996339000	0.007479000
C	2.976670000	-4.119984000	0.646894000
C	1.612149000	-4.049554000	0.592277000
C	1.242607000	-2.689620000	0.337579000
N	2.402313000	-1.977312000	0.250911000
C	3.484610000	-2.806397000	0.428854000
C	-0.081134000	-2.259932000	0.198968000
C	6.442140000	0.772051000	-0.140811000
C	6.552262000	-0.557592000	0.082283000
C	5.187271000	-1.056859000	0.160408000
N	4.289002000	-0.055118000	-0.008329000
C	5.010831000	1.064035000	-0.187384000
C	4.807388000	-2.379553000	0.379551000
C	1.500266000	4.282270000	-0.518148000
C	2.846494000	4.204779000	-0.577308000
C	3.188205000	2.795779000	-0.388176000
N	2.063466000	2.050494000	-0.222538000
C	1.035995000	2.917511000	-0.293742000
C	-0.317392000	2.563584000	-0.166554000
C	4.505792000	2.357478000	-0.374709000
C	-3.192578000	-1.458476000	-0.298233000
C	-4.462082000	-0.839914000	-0.171591000
C	-4.561168000	0.576908000	0.188179000

C	-3.393634000	1.376088000	0.263788000
C	-3.125661000	-2.789892000	-0.758992000
C	-4.266930000	-3.519838000	-1.007871000
C	-5.522354000	-2.930960000	-0.824996000
C	-5.610134000	-1.612450000	-0.434332000
C	-5.801745000	1.181263000	0.470494000
C	-5.896073000	2.512442000	0.814000000
C	-4.737592000	3.290076000	0.917378000
C	-3.509323000	2.724671000	0.656344000
H	1.079825000	0.374173000	-0.022207000
H	3.589540000	-4.991129000	0.820380000
H	0.901541000	-4.853015000	0.712620000
H	2.583542000	-0.983290000	0.076670000
H	-0.799603000	-3.055456000	0.322794000
H	7.231208000	1.500780000	-0.258305000
H	7.447783000	-1.152857000	0.184927000
H	5.576634000	-3.129719000	0.519674000
H	0.867716000	5.153025000	-0.611608000
H	3.560861000	5.000706000	-0.731018000
H	-1.038405000	3.360680000	-0.260207000
H	5.253626000	3.132291000	-0.514784000
H	-2.166930000	-3.232072000	-0.984867000
H	-4.188719000	-4.537323000	-1.371380000
H	-6.424701000	-3.496004000	-1.024773000
H	-6.591559000	-1.165904000	-0.358737000
H	-6.709193000	0.594679000	0.442125000
H	-6.865026000	2.946408000	1.029123000
H	-4.801313000	4.326340000	1.226412000
H	-2.619477000	3.315845000	0.809768000

MePhPa

C	2.034786000	-0.695839000	0.294066000
C	2.034803000	0.695833000	0.293959000
C	0.718644000	1.129970000	0.680739000
N	-0.028935000	0.000064000	0.932335000
C	0.718619000	-1.129889000	0.680891000
C	-2.821269000	-4.249930000	0.219189000
C	-1.534343000	-4.227431000	0.626239000
C	-1.113563000	-2.825840000	0.568912000
N	-2.130127000	-2.035594000	0.153329000
C	-3.171170000	-2.860612000	-0.071320000
C	0.207848000	-2.426257000	0.780520000
C	-6.139319000	0.681469000	-1.081798000
C	-6.139324000	-0.681467000	-1.081745000
C	-4.824185000	-1.124276000	-0.716950000
N	-4.065264000	0.000011000	-0.527464000
C	-4.824172000	1.124291000	-0.717045000
C	-4.426349000	-2.439573000	-0.517934000
C	-1.534295000	4.227527000	0.625873000
C	-2.821213000	4.250000000	0.218797000
C	-3.171110000	2.860661000	-0.071621000
N	-2.130086000	2.035653000	0.153151000
C	-1.113503000	2.825939000	0.568622000
C	0.207909000	2.426362000	0.780239000
C	-4.426305000	2.439599000	-0.518168000
C	3.209633000	-1.446630000	-0.089729000
C	4.428602000	-0.733766000	-0.216915000
C	4.428679000	0.733598000	-0.216746000
C	3.209732000	1.446539000	-0.089760000
C	3.203163000	-2.834307000	-0.336667000
C	4.370213000	-3.522572000	-0.587123000
C	5.589107000	-2.837616000	-0.606350000
C	5.608138000	-1.468885000	-0.443953000
C	5.608360000	1.468680000	-0.443149000
C	5.589472000	2.837430000	-0.605405000
C	4.370599000	3.522439000	-0.586666000
C	3.203423000	2.834229000	-0.336643000
C	-1.101187000	0.000134000	1.935653000
H	-3.481548000	-5.098891000	0.116418000
H	-0.908997000	-5.055402000	0.928339000
H	0.916968000	-3.222755000	0.957005000
H	-6.963739000	1.345806000	-1.291532000

H	-6.963748000	-1.345812000	-1.291435000
H	-3.070126000	0.000016000	-0.310140000
H	-5.180658000	-3.197824000	-0.693613000
H	-0.908956000	5.055525000	0.927914000
H	-3.481489000	5.098956000	0.115964000
H	0.917044000	3.222857000	0.956647000
H	-5.180606000	3.197848000	-0.693892000
H	2.265119000	-3.365735000	-0.376972000
H	4.338778000	-4.588169000	-0.779644000
H	6.514025000	-3.372595000	-0.784779000
H	6.559158000	-0.960640000	-0.515979000
H	6.559402000	0.960401000	-0.514663000
H	6.514500000	3.372391000	-0.783314000
H	4.339293000	4.588047000	-0.779145000
H	2.265423000	3.365713000	-0.377236000
H	-0.984091000	0.891964000	2.549455000
H	-2.083314000	0.000110000	1.483485000
H	-0.984094000	-0.891607000	2.549584000

MePhPb

C	-2.003195000	-0.711327000	0.270591000
C	-2.151948000	0.661859000	0.376106000
C	-0.836639000	1.220641000	0.600284000
N	0.019432000	0.151181000	0.852722000
C	-0.640992000	-1.037224000	0.634251000
C	3.081662000	-4.038478000	0.727649000
C	1.740132000	-4.017120000	0.985994000
C	1.257343000	-2.690161000	0.733515000
N	2.331585000	-1.953587000	0.328855000
C	3.466972000	-2.728217000	0.308412000
C	-0.084759000	-2.300027000	0.800865000
C	6.188971000	0.868264000	-0.983849000
C	6.345467000	-0.459251000	-0.773605000
C	5.032401000	-0.950814000	-0.383159000
N	4.118862000	0.047106000	-0.358690000
C	4.785243000	1.165954000	-0.710305000
C	4.727843000	-2.274346000	-0.051107000
C	1.256416000	4.326429000	-0.218880000
C	2.560539000	4.306662000	-0.571309000
C	2.977062000	2.905156000	-0.495941000
N	1.947219000	2.118201000	-0.106884000
C	0.902533000	2.934604000	0.069413000
C	-0.408542000	2.516730000	0.384625000
C	4.265094000	2.466054000	-0.783093000
C	-3.056723000	-1.536241000	-0.278066000
C	-4.325202000	-0.937472000	-0.485748000
C	-4.540731000	0.470882000	-0.142455000
C	-3.450332000	1.283808000	0.254345000
C	-2.851723000	-2.864754000	-0.704153000
C	-3.875546000	-3.606568000	-1.251912000
C	-5.141563000	-3.034785000	-1.410945000
C	-5.350234000	-1.721246000	-1.048847000
C	-5.823704000	1.050833000	-0.182371000
C	-6.032354000	2.370108000	0.157077000
C	-4.958973000	3.158464000	0.585600000
C	-3.694205000	2.616632000	0.642155000
C	1.107000000	0.273941000	1.835413000
H	3.760438000	-4.872735000	0.818623000
H	1.117030000	-4.830545000	1.325227000
H	2.366359000	-0.982728000	0.011678000
H	-0.778226000	-3.114201000	0.957965000
H	6.932413000	1.592457000	-1.284746000
H	7.240581000	-1.056784000	-0.866999000
H	5.527642000	-3.005832000	-0.059638000
H	0.587944000	5.173299000	-0.157269000
H	3.192004000	5.134701000	-0.859921000
H	-1.164371000	3.282161000	0.292140000
H	4.971209000	3.234030000	-1.085614000
H	-1.863204000	-3.295687000	-0.657676000
H	-3.689532000	-4.621343000	-1.582437000
H	-5.949440000	-3.609230000	-1.847735000
H	-6.323608000	-1.288648000	-1.232783000

H	-6.679766000	0.453340000	-0.462570000
H	-7.032311000	2.785047000	0.118943000
H	-5.123501000	4.182882000	0.896871000
H	-2.889780000	3.212833000	1.046038000
H	0.924015000	1.175207000	2.419293000
H	1.068318000	-0.598773000	2.488065000
H	2.082226000	0.376281000	1.367222000

PhPaH^{+}

C	2.082809000	-0.690524000	0.059221000
C	2.066069000	0.671798000	-0.103140000
C	0.654003000	1.064067000	0.002141000
N	-0.156012000	-0.028280000	-0.006378000
C	0.671586000	-1.107052000	0.003099000
C	-2.882545000	-4.211796000	-0.415663000
C	-1.522177000	-4.229117000	-0.338087000
C	-1.071032000	-2.874036000	-0.223765000
N	-2.183284000	-2.079972000	-0.218630000
C	-3.318163000	-2.847313000	-0.350522000
C	0.239740000	-2.428548000	-0.126812000
C	-6.393060000	0.749817000	-0.328861000
C	-6.385141000	-0.600737000	-0.572650000
C	-5.083372000	-1.094809000	-0.296433000
N	-4.309328000	-0.012027000	0.088646000
C	-5.094278000	1.130721000	0.097805000
C	-4.628449000	-2.403402000	-0.422532000
C	-1.496374000	4.106694000	0.894582000
C	-2.859353000	4.112004000	0.926903000
C	-3.320801000	2.806779000	0.556608000
N	-2.197100000	2.044109000	0.332309000
C	-1.070138000	2.797635000	0.499233000
C	0.231393000	2.363709000	0.285114000
C	-4.639744000	2.408023000	0.409846000
C	3.313341000	-1.403271000	0.287299000
C	4.523833000	-0.675430000	0.133295000
C	4.497809000	0.730349000	-0.268609000
C	3.262793000	1.422568000	-0.368273000
C	5.678331000	1.433986000	-0.582302000
C	5.647154000	2.749666000	-0.987130000
C	4.421524000	3.412854000	-1.131535000
C	3.250492000	2.753994000	-0.837261000
C	3.366852000	-2.730527000	0.764878000
C	4.569074000	-3.351537000	1.012190000
C	5.766697000	-2.654563000	0.808967000
C	5.737933000	-1.341510000	0.396746000
H	-3.551936000	-5.054377000	-0.499470000
H	-0.869363000	-5.088380000	-0.350335000
H	-2.077137000	-1.078549000	-0.312894000
H	0.989152000	-3.202039000	-0.198819000
H	-7.212373000	1.437938000	-0.471244000
H	-7.197592000	-1.205041000	-0.946558000
H	-3.554568000	-0.124409000	0.755538000
H	-5.379606000	-3.157296000	-0.623951000
H	-0.825826000	4.921506000	1.120682000
H	-3.512374000	4.930572000	1.188663000
H	-2.139460000	1.130463000	-0.094408000
H	0.994075000	3.115211000	0.431911000
H	-5.391869000	3.181055000	0.508863000
H	6.636754000	0.937316000	-0.532318000
H	6.571508000	3.260801000	-1.226383000
H	4.394031000	4.430162000	-1.501982000
H	2.309915000	3.249401000	-1.033113000
H	2.455626000	-3.256490000	1.008386000
H	4.587419000	-4.366385000	1.389902000
H	6.716026000	-3.136071000	1.008360000
H	6.676825000	-0.815144000	0.301434000

PhPbH^{+}

C	2.050243000	-0.696771000	0.064856000
C	2.050368000	0.697042000	0.064709000
C	0.701252000	1.138012000	0.284218000
N	-0.095477000	0.000279000	0.325855000

C	0.701065000	-1.137417000	0.284692000
C	-2.938635000	-4.188665000	0.433938000
C	-1.593996000	-4.161783000	0.668206000
C	-1.127417000	-2.825983000	0.445126000
N	-2.224043000	-2.078203000	0.112572000
C	-3.346181000	-2.865816000	0.078323000
C	0.204609000	-2.414653000	0.486087000
C	-6.368391000	0.673994000	-0.678377000
C	-6.368351000	-0.674167000	-0.678434000
C	-4.989168000	-1.077347000	-0.433044000
N	-4.170001000	-0.000131000	-0.318285000
C	-4.989281000	1.077287000	-0.432852000
C	-4.616797000	-2.408836000	-0.234556000
C	-1.594016000	4.162150000	0.667362000
C	-2.938878000	4.188654000	0.433674000
C	-3.346126000	2.865813000	0.078249000
N	-2.223862000	2.078465000	0.112091000
C	-1.127252000	2.826630000	0.444087000
C	0.204832000	2.415255000	0.485007000
C	-4.616911000	2.408598000	-0.234366000
C	3.267551000	-1.450165000	-0.115851000
C	4.488207000	-0.733888000	-0.033956000
C	4.488374000	0.733442000	-0.033731000
C	3.267935000	1.450056000	-0.115905000
C	5.687780000	1.469847000	-0.061469000
C	5.690012000	2.837848000	-0.230525000
C	4.485717000	3.523978000	-0.421368000
C	3.294066000	2.836176000	-0.366455000
C	3.293194000	-2.836250000	-0.366655000
C	4.484649000	-3.524344000	-0.422200000
C	5.689202000	-2.838543000	-0.231854000
C	5.687413000	-1.470577000	-0.062477000
H	-0.905690000	0.000509000	0.937150000
H	-3.606100000	-5.033631000	0.505988000
H	-0.957824000	-4.981201000	0.965940000
H	-2.264129000	-1.123292000	-0.219106000
H	0.919010000	-3.197236000	0.692766000
H	-7.200527000	1.349776000	-0.809071000
H	-7.200443000	-1.349996000	-0.809172000
H	-5.399529000	-3.156674000	-0.284184000
H	-0.958008000	4.981846000	0.964679000
H	-3.606492000	5.033452000	0.506258000
H	-2.263632000	1.123725000	-0.220129000
H	0.919215000	3.197960000	0.691314000
H	-5.399638000	3.156457000	-0.283848000
H	6.638985000	0.965602000	0.028446000
H	6.630338000	3.374573000	-0.254004000
H	4.490300000	4.587609000	-0.624322000
H	2.375727000	3.365570000	-0.573398000
H	2.374597000	-3.365339000	-0.573231000
H	4.488895000	-4.587936000	-0.625369000
H	6.629386000	-3.375487000	-0.256031000
H	6.638813000	-0.966612000	0.026943000

PhPcH^+

C	-2.087881000	0.707001000	0.047308000
C	-2.079195000	-0.681883000	0.063687000
C	-0.707745000	-1.116543000	-0.075112000
N	0.064247000	0.019428000	-0.072149000
C	-0.717794000	1.147745000	-0.091163000
C	2.905346000	4.187227000	-0.331231000
C	1.567474000	4.166266000	-0.476213000
C	1.165565000	2.767950000	-0.339594000
N	2.234482000	1.959699000	-0.158459000
C	3.311487000	2.801067000	-0.128628000
C	-0.190039000	2.407309000	-0.310055000
C	6.427317000	-0.664580000	0.574745000
C	6.436727000	0.702722000	0.563099000
C	5.106782000	1.143307000	0.288317000
N	4.327609000	0.028540000	0.171068000
C	5.091958000	-1.102033000	0.309591000
C	4.632151000	2.440084000	0.109227000

C	1.586057000	-4.087346000	-0.727225000
C	2.945085000	-4.110757000	-0.567246000
C	3.359275000	-2.836849000	-0.089704000
N	2.216089000	-2.054665000	0.024335000
C	1.110840000	-2.805653000	-0.330340000
C	-0.218538000	-2.394906000	-0.299080000
C	4.658178000	-2.412617000	0.143226000
C	-3.319939000	1.452507000	0.111897000
C	-4.526828000	0.723244000	-0.039222000
C	-4.516327000	-0.742261000	-0.007218000
C	-3.297551000	-1.445630000	0.165472000
C	-5.709441000	-1.490163000	-0.040878000
C	-5.710800000	-2.854457000	0.151876000
C	-4.514712000	-3.525807000	0.430861000
C	-3.328211000	-2.827861000	0.440192000
C	-3.374045000	2.845521000	0.318243000
C	-4.570610000	3.523554000	0.268504000
C	-5.754500000	2.821322000	0.013787000
C	-5.730922000	1.450062000	-0.116913000
H	1.030296000	0.088826000	-0.370091000
H	3.575437000	5.033905000	-0.348501000
H	0.891764000	4.993209000	-0.637203000
H	-0.888043000	3.213484000	-0.475434000
H	7.258794000	-1.329788000	0.750853000
H	7.277626000	1.359325000	0.724884000
H	3.373807000	0.114574000	-0.159415000
H	5.365022000	3.236284000	0.162644000
H	0.954310000	-4.875738000	-1.107023000
H	3.621260000	-4.919710000	-0.798820000
H	2.151785000	-1.312492000	0.711660000
H	-0.941068000	-3.163045000	-0.530506000
H	5.424646000	-3.178076000	0.140982000
H	-6.657486000	-0.996426000	-0.197766000
H	-6.646110000	-3.399771000	0.126174000
H	-4.523736000	-4.585445000	0.653887000
H	-2.420689000	-3.343562000	0.718957000
H	-2.475996000	3.389488000	0.570326000
H	-4.596562000	4.592858000	0.437209000
H	-6.697487000	3.350594000	-0.045476000
H	-6.669556000	0.935225000	-0.262184000

MePhPaH^{+}

C	2.104018000	-0.711782000	0.266219000
C	2.012204000	0.677445000	0.237581000
C	0.674263000	1.040371000	0.623271000
N	-0.030503000	-0.128326000	0.862092000
C	0.809653000	-1.217183000	0.633075000
C	-2.797909000	-4.219836000	0.165978000
C	-1.503584000	-4.251525000	0.603457000
C	-0.967352000	-2.932004000	0.497466000
N	-1.978012000	-2.132726000	0.016501000
C	-3.111844000	-2.877237000	-0.209915000
C	0.348871000	-2.527681000	0.716640000
C	-6.196255000	0.647622000	-1.107193000
C	-6.108984000	-0.711488000	-1.190924000
C	-4.786824000	-1.103316000	-0.794432000
N	-4.109452000	0.051503000	-0.503717000
C	-4.929025000	1.135850000	-0.658029000
C	-4.345674000	-2.413816000	-0.649433000
C	-1.604418000	4.110242000	0.817693000
C	-2.902550000	4.170532000	0.463630000
C	-3.283477000	2.806094000	0.107308000
N	-2.241080000	1.948667000	0.257412000
C	-1.202383000	2.708986000	0.668924000
C	0.135114000	2.310922000	0.790095000
C	-4.546094000	2.439100000	-0.356082000
C	-1.016506000	-0.173440000	1.964205000
C	3.333853000	-1.392781000	-0.065834000
C	4.499241000	-0.593727000	-0.189787000
C	4.393757000	0.867565000	-0.255482000
C	3.127149000	1.498621000	-0.167542000
C	5.517783000	1.676312000	-0.510483000

C	5.395376000	3.030409000	-0.735972000
C	4.129778000	3.626517000	-0.757695000
C	3.014336000	2.868420000	-0.480613000
C	3.435151000	-2.787355000	-0.238832000
C	4.656321000	-3.395420000	-0.424968000
C	5.820325000	-2.619810000	-0.444049000
C	5.736328000	-1.248064000	-0.345985000
H	-3.500097000	-5.038550000	0.124619000
H	-0.954486000	-5.101783000	0.977984000
H	-1.793831000	-1.210460000	-0.355530000
H	1.059338000	-3.318895000	0.902651000
H	-7.051141000	1.270487000	-1.320767000
H	-6.880067000	-1.406722000	-1.486052000
H	-3.161977000	0.212750000	-0.169310000
H	-5.085718000	-3.178813000	-0.851728000
H	-0.955543000	4.911905000	1.138848000
H	-3.555250000	5.030279000	0.431203000
H	0.842868000	3.112115000	0.953724000
H	-5.292425000	3.214415000	-0.479594000
H	-0.805478000	0.650467000	2.644262000
H	-2.037973000	-0.067615000	1.613515000
H	-0.901982000	-1.116950000	2.492773000
H	6.504479000	1.239201000	-0.559126000
H	6.278869000	3.623718000	-0.936773000
H	4.025233000	4.675341000	-1.006002000
H	2.039612000	3.325899000	-0.560489000
H	2.545044000	-3.395944000	-0.272873000
H	4.712894000	-4.467933000	-0.563278000
H	6.786748000	-3.092285000	-0.568929000
H	6.650374000	-0.675689000	-0.408911000

MePhPbH$^+$

C	-2.135798000	0.666762000	0.365285000
C	-2.011205000	-0.710784000	0.241005000
C	-0.665406000	-1.069881000	0.606112000
N	0.054053000	0.104647000	0.801842000
C	-0.809347000	1.190649000	0.573556000
C	2.648241000	4.296677000	-0.454498000
C	1.329823000	4.281012000	-0.089592000
C	0.939010000	2.919908000	0.106647000
N	2.042014000	2.155907000	-0.138588000
C	3.102708000	2.945123000	-0.494944000
C	-0.363979000	2.482980000	0.391075000
C	6.246983000	-0.566349000	-0.844070000
C	6.175228000	0.762418000	-1.065974000
C	4.800178000	1.146106000	-0.779862000
N	4.051213000	0.067547000	-0.414697000
C	4.912234000	-0.979146000	-0.434174000
C	4.368001000	2.469984000	-0.818923000
C	1.681515000	-4.057773000	1.098232000
C	3.017561000	-4.067175000	0.826276000
C	3.376054000	-2.767345000	0.338491000
N	2.229584000	-2.015727000	0.342097000
C	1.171938000	-2.751190000	0.785585000
C	-0.161944000	-2.345448000	0.824151000
C	4.611422000	-2.298388000	-0.063526000
C	-3.419169000	1.316681000	0.251068000
C	-4.520686000	0.523073000	-0.156716000
C	-4.331626000	-0.886734000	-0.511610000
C	-3.076959000	-1.515204000	-0.309495000
C	-5.371360000	-1.647837000	-1.077122000
C	-5.185251000	-2.963670000	-1.443596000
C	-3.931451000	-3.563412000	-1.289463000
C	-2.892885000	-2.844592000	-0.740170000
C	-3.638607000	2.648977000	0.654241000
C	-4.893430000	3.212007000	0.598659000
C	-5.976708000	2.445439000	0.155350000
C	-5.792228000	1.125953000	-0.196572000
C	1.037452000	0.210985000	1.906987000
H	3.263373000	5.155240000	-0.675997000
H	0.669826000	5.125105000	0.039243000
H	2.102207000	1.148077000	-0.183264000

H	-1.105170000	3.264288000	0.328065000
H	7.100816000	-1.221284000	-0.934709000
H	6.957832000	1.438488000	-1.376988000
H	5.098150000	3.218073000	-1.105881000
H	1.079335000	-4.864554000	1.487171000
H	3.712615000	-4.883035000	0.953609000
H	2.212905000	-1.090981000	-0.069159000
H	-0.883116000	-3.132358000	0.997300000
H	5.425197000	-3.013950000	-0.070980000
H	-6.337339000	-1.199031000	-1.258470000
H	-6.004086000	-3.520894000	-1.881588000
H	-3.768580000	-4.579993000	-1.625173000
H	-1.913153000	-3.296907000	-0.699762000
H	-2.831688000	3.228922000	1.077135000
H	-5.044707000	4.233965000	0.923059000
H	-6.968633000	2.878478000	0.117317000
H	-6.658562000	0.548571000	-0.485409000
H	0.847942000	-0.595071000	2.615854000
H	2.073609000	0.145102000	1.576264000
H	0.891324000	1.168313000	2.403305000

PhPH_2^{2+}

C	2.058936000	-0.701337000	-0.023374000
C	2.058920000	0.701239000	-0.023247000
C	0.715265000	1.134285000	-0.265910000
N	-0.097093000	-0.000036000	-0.283481000
C	0.715286000	-1.134341000	-0.266040000
C	-2.957324000	-4.095405000	-0.725589000
C	-1.615300000	-4.068132000	-0.987231000
C	-1.106445000	-2.798910000	-0.579671000
N	-2.182063000	-2.051213000	-0.137482000
C	-3.328341000	-2.834672000	-0.171426000
C	0.229149000	-2.401632000	-0.559006000
C	-6.205137000	0.685466000	1.116672000
C	-6.205122000	-0.685336000	1.116807000
C	-4.976591000	-1.128022000	0.550072000
N	-4.225941000	-0.000003000	0.267409000
C	-4.976614000	1.128074000	0.549860000
C	-4.586581000	-2.432132000	0.253527000
C	-1.615214000	4.068048000	-0.987533000
C	-2.957274000	4.095307000	-0.726037000
C	-3.328307000	2.834625000	-0.171784000
N	-2.182018000	2.051229000	-0.137634000
C	-1.106385000	2.798881000	-0.579801000
C	0.229214000	2.401576000	-0.558947000
C	-4.586581000	2.432126000	0.253152000
C	3.265458000	-1.454565000	0.163917000
C	4.488033000	-0.732673000	0.099677000
C	4.487998000	0.732746000	0.099768000
C	3.265369000	1.454529000	0.164232000
C	3.283325000	-2.845532000	0.397501000
C	4.473397000	-3.532214000	0.457537000
C	5.680699000	-2.840753000	0.288589000
C	5.685611000	-1.471013000	0.133246000
C	5.685516000	1.471195000	0.133262000
C	5.680479000	2.840904000	0.288833000
C	4.473139000	3.532230000	0.458123000
C	3.283123000	2.845469000	0.398096000
H	-0.884307000	-0.000030000	-0.925327000
H	-3.649042000	-4.901312000	-0.921999000
H	-1.016569000	-4.850091000	-1.430228000
H	-2.062104000	-1.324901000	0.558662000
H	0.952357000	-3.163582000	-0.815567000
H	-6.978910000	1.341627000	1.486901000
H	-6.978876000	-1.341440000	1.487175000
H	-3.554573000	-0.000089000	-0.491245000
H	-5.344658000	-3.201889000	0.339414000
H	-1.016452000	4.849993000	-1.430509000
H	-3.648993000	4.901176000	-0.922600000
H	-2.062148000	1.324795000	0.558390000
H	0.952516000	3.163481000	-0.815378000
H	-5.344648000	3.201907000	0.338918000

H	2.361244000	-3.375923000	0.590733000
H	4.479813000	-4.597673000	0.649682000
H	6.619803000	-3.379305000	0.319073000
H	6.639575000	-0.969851000	0.058799000
H	6.639524000	0.970160000	0.058519000
H	6.619530000	3.379555000	0.319206000
H	4.479520000	4.597645000	0.650515000
H	2.360992000	3.375705000	0.591518000

MePhPH_2^{2+}

C	-2.003229000	-0.705018000	0.184814000
C	-2.146542000	0.684291000	0.319847000
C	-0.826039000	1.211849000	0.510765000
N	0.070526000	0.134040000	0.744244000
C	-0.668542000	-1.046082000	0.567197000
C	3.017650000	-4.005317000	1.023441000
C	1.679149000	-3.994804000	1.285585000
C	1.152357000	-2.728832000	0.867303000
N	2.212133000	-1.992512000	0.392420000
C	3.369456000	-2.745780000	0.435384000
C	-0.177066000	-2.321423000	0.848263000
C	6.038821000	0.698641000	-1.379188000
C	6.109817000	-0.655130000	-1.166542000
C	4.941844000	-1.053607000	-0.464847000
N	4.154969000	0.075825000	-0.290966000
C	4.826385000	1.175249000	-0.811869000
C	4.608056000	-2.337631000	-0.027187000
C	1.359832000	4.308630000	0.025190000
C	2.687861000	4.301803000	-0.301345000
C	3.110255000	2.942307000	-0.415903000
N	2.014606000	2.158654000	-0.113860000
C	0.922863000	2.953807000	0.126277000
C	-0.389396000	2.513842000	0.352484000
C	4.369206000	2.488546000	-0.788584000
C	-3.054030000	-1.521036000	-0.359367000
C	-4.319865000	-0.906137000	-0.551078000
C	-4.525783000	0.499954000	-0.192020000
C	-3.433504000	1.313476000	0.215409000
C	-2.849894000	-2.849350000	-0.787788000
C	-3.883537000	-3.584036000	-1.322616000
C	-5.147245000	-2.999287000	-1.465051000
C	-5.352486000	-1.683939000	-1.103320000
C	-5.804098000	1.085075000	-0.229966000
C	-6.003332000	2.402467000	0.124233000
C	-4.931183000	3.186901000	0.568475000
C	-3.669062000	2.643982000	0.621257000
C	0.807453000	0.260095000	2.037406000
H	3.722928000	-4.796719000	1.230150000
H	1.091642000	-4.777740000	1.741544000
H	2.076621000	-1.218156000	-0.243512000
H	-0.910832000	-3.093508000	1.042723000
H	6.748478000	1.316680000	-1.909281000
H	6.886985000	-1.330515000	-1.492152000
H	3.574933000	0.170058000	0.535825000
H	5.389211000	-3.087366000	-0.070543000
H	0.728244000	5.167900000	0.193608000
H	3.335727000	5.153971000	-0.443889000
H	1.909966000	1.185749000	-0.365567000
H	-1.133214000	3.293806000	0.291873000
H	5.072120000	3.246102000	-1.116029000
H	-1.861621000	-3.285946000	-0.759609000
H	-3.713342000	-4.599068000	-1.658672000
H	-5.961891000	-3.570485000	-1.892820000
H	-6.328059000	-1.254028000	-1.277333000
H	-6.664427000	0.499117000	-0.518401000
H	-7.001092000	2.822173000	0.088013000
H	-5.100646000	4.204881000	0.895669000
H	-2.872118000	3.235799000	1.047355000
H	1.270384000	1.241393000	2.096109000
H	0.103195000	0.148094000	2.865035000
H	1.574142000	-0.505282000	2.117089000

PyPa

C	-1.719501000	-0.690203000	-0.061231000
C	-1.719502000	0.690214000	0.061218000
C	-0.339045000	1.132912000	0.004745000
N	0.426948000	0.000005000	-0.000008000
C	-0.339044000	-1.132900000	-0.004757000
C	3.248095000	-4.183907000	0.532098000
C	1.899937000	-4.178357000	0.480307000
C	1.509099000	-2.787695000	0.267521000
N	2.583882000	-1.978484000	0.197663000
C	3.663018000	-2.798785000	0.351767000
C	0.162858000	-2.411812000	0.147565000
C	6.829839000	0.677906000	-0.097879000
C	6.829837000	-0.677918000	0.097887000
C	5.470084000	-1.108907000	0.156634000
N	4.692227000	-0.000004000	-0.000003000
C	5.470087000	1.108898000	-0.156634000
C	4.993635000	-2.406928000	0.330674000
C	1.899950000	4.178361000	-0.480301000
C	3.248108000	4.183906000	-0.532089000
C	3.663026000	2.798782000	-0.351760000
N	2.583887000	1.978488000	-0.197643000
C	1.509106000	2.787699000	-0.267526000
C	0.162863000	2.411822000	-0.147574000
C	4.993641000	2.406920000	-0.330675000
C	-2.946372000	-1.429495000	-0.262639000
C	-4.166304000	-0.703479000	-0.142274000
C	-4.166306000	0.703479000	0.142276000
C	-2.946376000	1.429502000	0.262630000
C	-2.999396000	-2.771647000	-0.647816000
C	-4.216665000	-3.419191000	-0.832808000
C	-5.407669000	-2.740449000	-0.646514000
C	-5.402536000	-1.380866000	-0.315638000
C	-5.402540000	1.380859000	0.315647000
C	-5.407680000	2.740443000	0.646518000
C	-4.216679000	3.419193000	0.832799000
C	-2.999407000	2.771655000	0.647801000
C	-6.634250000	-0.657110000	-0.154333000
C	-6.634252000	0.657096000	0.154352000
H	1.443528000	0.000007000	-0.000009000
H	3.916289000	-5.020509000	0.675779000
H	1.216710000	-5.010316000	0.571351000
H	-0.551746000	-3.214261000	0.244313000
H	7.681106000	1.334576000	-0.191624000
H	7.681103000	-1.334589000	0.191635000
H	3.676341000	-0.000004000	-0.000008000
H	5.743439000	-3.180001000	0.453431000
H	1.216727000	5.010324000	-0.571344000
H	3.916305000	5.020506000	-0.675770000
H	-0.551735000	3.214275000	-0.244318000
H	5.743447000	3.179991000	-0.453438000
H	-2.089639000	-3.313478000	-0.855984000
H	-4.226445000	-4.459944000	-1.133503000
H	-6.357107000	-3.247667000	-0.777467000
H	-6.357121000	3.247655000	0.777477000
H	-4.226462000	4.459947000	1.133488000
H	-2.089654000	3.313495000	0.855960000
H	-7.565821000	-1.197042000	-0.283636000
H	-7.565825000	1.197022000	0.283664000

PyPb

C	1.719374000	0.683603000	-0.070725000
C	1.719349000	-0.683769000	0.070249000
C	0.306816000	-1.087569000	0.007571000
N	-0.500633000	-0.000011000	-0.000197000
C	0.306879000	1.087478000	-0.008053000
C	-3.250922000	4.180922000	0.568542000
C	-1.887159000	4.176041000	0.515503000
C	-1.458185000	2.826246000	0.292747000
N	-2.584679000	2.061251000	0.222705000
C	-3.702103000	2.834879000	0.381217000
C	-0.143138000	2.396531000	0.160944000

C	-6.829013000	-0.667178000	-0.104125000
C	-6.828954000	0.667416000	0.104821000
C	-5.428350000	1.065780000	0.162729000
N	-4.607555000	0.000094000	-0.000029000
C	-5.428434000	-1.065609000	-0.162333000
C	-5.013394000	2.384072000	0.351475000
C	-1.887492000	-4.175997000	-0.515886000
C	-3.251255000	-4.180822000	-0.568781000
C	-3.702363000	-2.834782000	-0.381179000
N	-2.584887000	-2.061244000	-0.222562000
C	-1.458430000	-2.826242000	-0.292966000
C	-0.143316000	-2.396595000	-0.161361000
C	-5.013620000	-2.383915000	-0.351151000
C	2.940569000	1.422319000	-0.288085000
C	4.163215000	0.699926000	-0.155669000
C	4.163192000	-0.700000000	0.155796000
C	2.940535000	-1.422468000	0.287805000
C	2.994320000	2.757307000	-0.703410000
C	4.210725000	3.400442000	-0.903108000
C	5.403513000	2.726568000	-0.702314000
C	5.399368000	1.374842000	-0.343461000
C	5.399330000	-1.374856000	0.343906000
C	5.403458000	-2.726628000	0.702584000
C	4.210654000	-3.400611000	0.902883000
C	2.994268000	-2.757521000	0.702921000
C	6.631214000	0.653866000	-0.167394000
C	6.631191000	-0.653791000	0.168303000
H	-3.906815000	5.024113000	0.722222000
H	-1.215624000	5.014928000	0.616769000
H	-2.599861000	1.059591000	0.071824000
H	0.594780000	3.177589000	0.266057000
H	-7.672200000	-1.334582000	-0.207401000
H	-7.672082000	1.334853000	0.208353000
H	-5.782329000	3.136884000	0.480758000
H	-1.216024000	-5.014917000	-0.617309000
H	-3.907173000	-5.023984000	-0.722520000
H	-2.600014000	-1.059574000	-0.071711000
H	0.594488000	-3.177732000	-0.266625000
H	-5.782610000	-3.136685000	-0.480328000
H	2.083622000	3.293368000	-0.925026000
H	4.220590000	4.434198000	-1.227474000
H	6.352341000	3.231939000	-0.844612000
H	6.352276000	-3.231951000	0.845122000
H	4.220478000	-4.434427000	1.227058000
H	2.083563000	-3.293708000	0.924176000
H	7.562925000	1.191002000	-0.307613000
H	7.562884000	-1.190864000	0.308879000

PyPc

C	1.777314000	-0.755133000	-0.066914000
C	1.692099000	0.611978000	0.059267000
C	0.253764000	0.934704000	-0.014475000
N	-0.475753000	-0.200892000	-0.018280000
C	0.390319000	-1.237874000	-0.021171000
C	-3.245817000	-4.179579000	0.486118000
C	-1.885999000	-4.265702000	0.426743000
C	-1.373661000	-2.943405000	0.239806000
N	-2.449702000	-2.096603000	0.192983000
C	-3.617080000	-2.802178000	0.338070000
C	-0.044085000	-2.558527000	0.121037000
C	-6.870160000	0.679045000	-0.048696000
C	-6.794574000	-0.672382000	0.142794000
C	-5.410615000	-1.042725000	0.182547000
N	-4.693866000	0.108319000	0.019516000
C	-5.534554000	1.179442000	-0.126070000
C	-4.925038000	-2.336886000	0.335752000
C	-1.882377000	4.073314000	-0.553142000
C	-3.227704000	4.179401000	-0.579112000
C	-3.733107000	2.830207000	-0.363216000
N	-2.717030000	1.942352000	-0.211113000
C	-1.580751000	2.659752000	-0.316271000
C	-0.258967000	2.218325000	-0.199433000

C	-5.082353000	2.480808000	-0.314660000
C	3.041179000	-1.424715000	-0.254638000
C	4.217616000	-0.627551000	-0.137743000
C	4.131903000	0.774194000	0.151645000
C	2.867165000	1.422317000	0.278042000
C	3.175783000	-2.768336000	-0.619567000
C	4.428630000	-3.342517000	-0.801899000
C	5.578395000	-2.589743000	-0.630367000
C	5.492486000	-1.231348000	-0.309504000
C	5.325147000	1.524314000	0.332600000
C	5.247817000	2.876229000	0.681393000
C	4.016366000	3.476876000	0.882230000
C	2.840957000	2.760086000	0.688776000
C	6.678324000	-0.433183000	-0.150791000
C	6.598491000	0.877309000	0.163456000
H	-3.956395000	-4.981150000	0.618764000
H	-1.273042000	-5.150823000	0.500976000
H	-2.270609000	-1.097190000	0.079470000
H	0.679480000	-3.354631000	0.206849000
H	-7.757412000	1.287625000	-0.131741000
H	-7.608918000	-1.373650000	0.243134000
H	-3.685776000	0.289299000	0.008640000
H	-5.682706000	-3.101500000	0.458873000
H	-1.143836000	4.852509000	-0.676143000
H	-3.834723000	5.060268000	-0.728690000
H	0.472533000	3.004330000	-0.325912000
H	-5.826696000	3.258897000	-0.432100000
H	2.299072000	-3.368443000	-0.811346000
H	4.501535000	-4.384963000	-1.088268000
H	6.556067000	-3.040298000	-0.761125000
H	6.164446000	3.439240000	0.818492000
H	3.963493000	4.510790000	1.201924000
H	1.899781000	3.240806000	0.910173000
H	7.640942000	-0.914897000	-0.283429000
H	7.495747000	1.472452000	0.294083000

PyPd

C	-1.676354000	-0.612642000	-0.073870000
C	-1.761367000	0.765814000	0.047908000
C	-0.412820000	1.287230000	0.004422000
N	0.424612000	0.200111000	0.002262000
C	-0.272182000	-0.977834000	-0.004478000
C	3.288654000	-4.146866000	0.571566000
C	1.925104000	-4.061383000	0.523055000
C	1.568549000	-2.693290000	0.292674000
N	2.735540000	-1.991616000	0.213778000
C	3.809581000	-2.834697000	0.374535000
C	0.248918000	-2.249044000	0.166945000
C	6.802296000	0.724249000	-0.127255000
C	6.899409000	-0.610517000	0.068718000
C	5.529369000	-1.097904000	0.137387000
N	4.641051000	-0.084603000	-0.010745000
C	5.373745000	1.031190000	-0.167391000
C	5.136527000	-2.420959000	0.331232000
C	1.894393000	4.288252000	-0.441132000
C	3.239952000	4.199045000	-0.497598000
C	3.567514000	2.783324000	-0.335081000
N	2.435866000	2.045748000	-0.186941000
C	1.416430000	2.923924000	-0.244733000
C	0.059742000	2.580941000	-0.130358000
C	4.881435000	2.332410000	-0.328515000
C	-2.857298000	-1.422766000	-0.285297000
C	-4.118924000	-0.774962000	-0.143320000
C	-4.203457000	0.629230000	0.142790000
C	-3.030549000	1.430862000	0.241446000
C	-2.833885000	-2.756312000	-0.703818000
C	-4.011598000	-3.475008000	-0.884567000
C	-5.239185000	-2.877477000	-0.663022000
C	-5.313024000	-1.525360000	-0.310158000
C	-5.476963000	1.230194000	0.326553000
C	-5.562333000	2.591316000	0.639871000
C	-4.414174000	3.348448000	0.790540000

C	-3.161502000	2.775250000	0.596706000
C	-6.585211000	-0.881118000	-0.125499000
C	-6.663219000	0.429928000	0.186442000
H	1.433797000	0.375246000	-0.015383000
H	3.893090000	-5.027073000	0.728054000
H	1.206568000	-4.859379000	0.632189000
H	2.926749000	-0.996368000	0.057683000
H	-0.475963000	-3.039522000	0.282534000
H	7.598372000	1.447519000	-0.230299000
H	7.789029000	-1.216549000	0.158900000
H	5.898184000	-3.181396000	0.456449000
H	1.270400000	5.166617000	-0.519653000
H	3.962465000	4.990803000	-0.633709000
H	-0.652504000	3.386899000	-0.211688000
H	5.636581000	3.102903000	-0.452143000
H	-1.896847000	-3.234193000	-0.946381000
H	-3.961041000	-4.506822000	-1.210928000
H	-6.157255000	-3.440607000	-0.788828000
H	-6.539779000	3.039588000	0.779538000
H	-4.485448000	4.393003000	1.068797000
H	-2.283876000	3.378679000	0.770289000
H	-7.482732000	-1.478177000	-0.243915000
H	-7.624721000	0.910537000	0.329866000

MePyPa

C	1.695746000	-0.696652000	0.361086000
C	1.695823000	0.696775000	0.361168000
C	0.375106000	1.129492000	0.729711000
N	-0.376282000	0.000112000	0.970245000
C	0.375031000	-1.129294000	0.729703000
C	-3.154953000	-4.249867000	0.199615000
C	-1.875559000	-4.227701000	0.629359000
C	-1.454190000	-2.825851000	0.581819000
N	-2.464378000	-2.035040000	0.149529000
C	-3.500062000	-2.860006000	-0.094822000
C	-0.137860000	-2.426450000	0.817558000
C	-6.450553000	0.681200000	-1.158434000
C	-6.450584000	-0.681233000	-1.158316000
C	-5.141906000	-1.124374000	-0.769078000
N	-4.386668000	-0.000012000	-0.565546000
C	-5.141847000	1.124348000	-0.769292000
C	-4.747913000	-2.439131000	-0.563866000
C	-1.875469000	4.227863000	0.628661000
C	-3.154867000	4.249962000	0.198925000
C	-3.499964000	2.860064000	-0.095325000
N	-2.464297000	2.035120000	0.149212000
C	-1.454136000	2.825994000	0.581448000
C	-0.137816000	2.426641000	0.817364000
C	-4.747811000	2.439126000	-0.564321000
C	2.874389000	-1.453131000	-0.007733000
C	4.087567000	-0.718495000	-0.152347000
C	4.087681000	0.718326000	-0.152175000
C	2.874619000	1.453135000	-0.007424000
C	2.899866000	-2.833167000	-0.220820000
C	4.096404000	-3.503341000	-0.460598000
C	5.291051000	-2.809451000	-0.509254000
C	5.302881000	-1.415094000	-0.382937000
C	5.303125000	1.414771000	-0.382558000
C	5.291553000	2.809163000	-0.508492000
C	4.097034000	3.503258000	-0.459640000
C	2.900371000	2.833230000	-0.220084000
C	6.527083000	-0.675230000	-0.525848000
C	6.527199000	0.674730000	-0.525660000
C	-1.467005000	0.000152000	1.953232000
H	-3.813027000	-5.098799000	0.083407000
H	-1.255314000	-5.055971000	0.940971000
H	0.567787000	-3.223076000	1.006264000
H	-7.270813000	1.345669000	-1.383519000
H	-7.270879000	-1.345701000	-1.383279000
H	-3.395656000	-0.000017000	-0.330060000
H	-5.498386000	-3.197656000	-0.754025000
H	-1.255219000	5.056187000	0.940123000

H	-3.812933000	5.098879000	0.082561000
H	0.567780000	3.223322000	1.006000000
H	-5.498265000	3.197633000	-0.754631000
H	1.980406000	-3.396690000	-0.239983000
H	4.084288000	-4.575360000	-0.616922000
H	6.225296000	-3.330814000	-0.685962000
H	6.225897000	3.330405000	-0.685036000
H	4.085115000	4.575329000	-0.615627000
H	1.981034000	3.396954000	-0.239051000
H	7.448574000	-1.231047000	-0.660232000
H	7.448786000	1.230425000	-0.659886000
H	-1.361518000	0.892027000	2.569081000
H	-2.440440000	-0.000029000	1.482608000
H	-1.361330000	-0.891517000	2.569357000

MePyPb

C	-1.676143000	-0.662099000	0.357250000
C	-1.795964000	0.715293000	0.468733000
C	-0.467128000	1.245914000	0.676080000
N	0.373721000	0.158952000	0.900227000
C	-0.313354000	-1.014596000	0.686750000
C	3.353301000	-4.085186000	0.638291000
C	2.020865000	-4.041761000	0.935410000
C	1.556635000	-2.701912000	0.715076000
N	2.633698000	-1.980115000	0.290364000
C	3.752394000	-2.776049000	0.226616000
C	0.225268000	-2.289220000	0.826047000
C	6.510109000	0.786003000	-1.084511000
C	6.645134000	-0.547434000	-0.900354000
C	5.332997000	-1.019373000	-0.481922000
N	4.441001000	-0.004832000	-0.416599000
C	5.120234000	1.107014000	-0.768178000
C	5.011536000	-2.342153000	-0.161538000
C	1.669301000	4.326124000	-0.129282000
C	2.963711000	4.287290000	-0.514165000
C	3.352807000	2.876200000	-0.477172000
N	2.317753000	2.102429000	-0.079269000
C	1.293572000	2.936012000	0.139230000
C	-0.017229000	2.538201000	0.475424000
C	4.625190000	2.417340000	-0.804997000
C	-2.761483000	-1.476282000	-0.147029000
C	-4.024816000	-0.837856000	-0.312773000
C	-4.187131000	0.563026000	-0.042806000
C	-3.080481000	1.370628000	0.347480000
C	-2.629010000	-2.810917000	-0.540214000
C	-3.722990000	-3.535936000	-1.003856000
C	-4.970251000	-2.945533000	-1.092722000
C	-5.136997000	-1.594602000	-0.766119000
C	-5.473111000	1.153081000	-0.164825000
C	-5.645986000	2.508288000	0.138746000
C	-4.577618000	3.268692000	0.579166000
C	-3.309842000	2.705958000	0.686396000
C	-6.420612000	-0.959414000	-0.892953000
C	-6.582716000	0.347978000	-0.598154000
C	1.486281000	0.250708000	1.858077000
H	4.017902000	-4.933584000	0.697500000
H	1.391720000	-4.847952000	1.280749000
H	2.678843000	-1.005367000	-0.013456000
H	-0.477258000	-3.092380000	0.997565000
H	7.259673000	1.500346000	-1.393650000
H	7.525009000	-1.161082000	-1.027823000
H	5.795903000	-3.088974000	-0.203306000
H	1.020304000	5.184920000	-0.033826000
H	3.605074000	5.108188000	-0.801338000
H	-0.758192000	3.320841000	0.417790000
H	5.338632000	3.176938000	-1.111372000
H	-1.659242000	-3.284379000	-0.535212000
H	-3.588687000	-4.568051000	-1.304711000
H	-5.825889000	-3.513629000	-1.440511000
H	-6.632307000	2.948386000	0.041995000
H	-4.725263000	4.307079000	0.850400000
H	-2.509083000	3.311220000	1.083107000

H	-7.257567000	-1.560428000	-1.231135000
H	-7.554040000	0.821690000	-0.688598000
H	1.333739000	1.148376000	2.456081000
H	1.446953000	-0.628525000	2.501744000
H	2.451740000	0.340788000	1.367734000

PyPaH^{+}

C	1.732935000	0.700174000	-0.069550000
C	1.718758000	-0.662870000	0.095648000
C	0.306804000	-1.057467000	0.000028000
N	-0.505255000	0.032978000	0.002051000
C	0.320288000	1.112897000	-0.018509000
C	-3.243675000	4.214051000	0.340992000
C	-1.883310000	4.233732000	0.264513000
C	-1.428414000	2.878105000	0.174294000
N	-2.538611000	2.081062000	0.181729000
C	-3.675428000	2.847386000	0.299859000
C	-0.116217000	2.434851000	0.088810000
C	-6.742164000	-0.756419000	0.360050000
C	-6.736826000	0.598798000	0.575105000
C	-5.436815000	1.090093000	0.284571000
N	-4.661497000	0.001370000	-0.079950000
C	-5.443615000	-1.143221000	-0.062828000
C	-4.984738000	2.401819000	0.382577000
C	-1.840553000	-4.117453000	-0.837962000
C	-3.203860000	-4.129350000	-0.858090000
C	-3.667742000	-2.821654000	-0.500944000
N	-2.545752000	-2.051015000	-0.295694000
C	-1.416766000	-2.801942000	-0.462582000
C	-0.115016000	-2.360950000	-0.265473000
C	-4.987347000	-2.424848000	-0.352494000
H	-3.907979000	0.100875000	-0.750269000
H	-2.489755000	-1.134326000	0.124799000
H	-2.429198000	1.081405000	0.290154000
C	2.962852000	1.424303000	-0.280482000
C	4.171039000	0.681917000	-0.115141000
C	4.148964000	-0.712455000	0.215291000
C	2.918057000	-1.420969000	0.338779000
C	5.372250000	-1.401712000	0.432331000
C	5.347786000	-2.748374000	0.807932000
C	4.143668000	-3.406518000	0.999631000
C	2.939790000	-2.750886000	0.773121000
C	3.043371000	2.751598000	-0.715543000
C	4.272790000	3.371389000	-0.902330000
C	5.450152000	2.680759000	-0.667817000
C	5.420989000	1.335288000	-0.289659000
C	6.640334000	0.601057000	-0.082811000
C	6.617510000	-0.701522000	0.269520000
H	-3.915494000	5.056046000	0.409846000
H	-1.232818000	5.094834000	0.262814000
H	0.630103000	3.211896000	0.149073000
H	-7.559417000	-1.443322000	0.519168000
H	-7.549605000	1.209021000	0.938543000
H	-5.737169000	3.157921000	0.570477000
H	-1.168337000	-4.931927000	-1.060314000
H	-3.855337000	-4.953909000	-1.104421000
H	0.648538000	-3.111634000	-0.410758000
H	-5.737633000	-3.201747000	-0.433451000
H	6.286025000	-3.265716000	0.973452000
H	4.137751000	-4.434668000	1.340096000
H	2.017906000	-3.270309000	0.993483000
H	2.150298000	3.302661000	-0.969573000
H	4.307098000	4.398647000	-1.243819000
H	6.408113000	3.170580000	-0.800595000
H	7.580787000	1.124099000	-0.213933000
H	7.538828000	-1.248837000	0.433253000

PyPbH^{+}

C	1.702410000	-0.697445000	0.079353000
C	1.702672000	0.698266000	0.079412000
C	0.353371000	1.137910000	0.298242000
N	-0.443735000	0.000624000	0.339119000

C	0.353028000	-1.136631000	0.298519000
C	-3.285862000	-4.189623000	0.427760000
C	-1.942191000	-4.162774000	0.666955000
C	-1.475244000	-2.826177000	0.448539000
N	-2.571003000	-2.078137000	0.113465000
C	-3.692562000	-2.866082000	0.073266000
C	-0.143793000	-2.414511000	0.495238000
C	-6.712096000	0.673724000	-0.694298000
C	-6.712090000	-0.674349000	-0.694043000
C	-5.333789000	-1.077555000	-0.443075000
N	-4.515157000	-0.000349000	-0.324774000
C	-5.333842000	1.077112000	-0.443382000
C	-4.962117000	-2.409113000	-0.244133000
C	-1.942735000	4.163311000	0.665955000
C	-3.286635000	4.189464000	0.427078000
C	-3.692600000	2.865953000	0.072402000
N	-2.570733000	2.078522000	0.112325000
C	-1.475214000	2.827225000	0.447212000
C	-0.143602000	2.415727000	0.494066000
C	-4.962277000	2.408482000	-0.244728000
H	-1.255659000	0.000908000	0.948102000
H	-2.608918000	1.123311000	-0.218726000
H	-2.610337000	-1.122329000	-0.215794000
C	2.918523000	-1.456558000	-0.103402000
C	4.136292000	-0.717891000	-0.055623000
C	4.136623000	0.717491000	-0.055336000
C	2.919226000	1.456808000	-0.102892000
C	5.372909000	1.412952000	-0.098992000
C	5.375861000	2.806629000	-0.232487000
C	4.189243000	3.503599000	-0.374852000
C	2.970239000	2.835358000	-0.318397000
C	2.968765000	-2.835016000	-0.319691000
C	4.187439000	-3.503844000	-0.376621000
C	5.374425000	-2.807570000	-0.233915000
C	5.372211000	-1.413958000	-0.099696000
C	6.604928000	-0.675710000	-0.050985000
C	6.605257000	0.674060000	-0.050637000
H	-3.953327000	-5.034943000	0.495482000
H	-1.306788000	-4.982605000	0.965188000
H	0.570003000	-3.197153000	0.703022000
H	-7.543629000	1.349528000	-0.828677000
H	-7.543604000	-1.350222000	-0.828178000
H	-5.744454000	-3.157074000	-0.297735000
H	-1.307865000	4.983535000	0.964241000
H	-3.954452000	5.034464000	0.495294000
H	0.569960000	3.198692000	0.701452000
H	-5.744674000	3.156389000	-0.298315000
H	6.324876000	3.329600000	-0.266505000
H	4.205765000	4.573593000	-0.540746000
H	2.065187000	3.398145000	-0.491469000
H	2.063358000	-3.397177000	-0.493029000
H	4.203397000	-4.573751000	-0.543148000
H	6.323173000	-3.331004000	-0.268237000
H	7.535580000	-1.231385000	-0.042351000
H	7.536178000	1.229278000	-0.041691000

PyPcH^+

C	-1.755568000	0.723195000	-0.123443000
C	-1.724411000	-0.656493000	0.030524000
C	-0.345528000	-1.083213000	-0.086136000
N	0.409456000	0.059141000	-0.119249000
C	-0.381053000	1.181089000	-0.136255000
C	3.237788000	4.200000000	0.357478000
C	1.899176000	4.203461000	0.220698000
C	1.503793000	2.807578000	0.048949000
N	2.574151000	1.981911000	0.065332000
C	3.649885000	2.804338000	0.265050000
C	0.149026000	2.454675000	-0.065399000
C	6.803086000	-0.703823000	0.339598000
C	6.802575000	0.651305000	0.530011000
C	5.464936000	1.113591000	0.353376000
N	4.690532000	0.021057000	0.086245000

C	5.465055000	-1.111523000	0.050717000
C	4.975201000	2.416576000	0.409029000
C	1.890530000	-4.042768000	-0.935562000
C	3.258688000	-4.060115000	-0.908515000
C	3.711066000	-2.810055000	-0.402877000
N	2.579265000	-2.041734000	-0.149476000
C	1.450043000	-2.782858000	-0.440928000
C	0.128227000	-2.368095000	-0.304668000
C	5.022515000	-2.400115000	-0.230916000
H	1.405809000	0.151210000	-0.273383000
H	2.575468000	-1.347407000	0.588665000
H	3.737178000	0.145472000	-0.232974000
C	-3.002906000	1.436347000	-0.269810000
C	-4.196995000	0.677011000	-0.095413000
C	-4.154585000	-0.724329000	0.207563000
C	-2.916566000	-1.422314000	0.301772000
C	-5.366513000	-1.428099000	0.436303000
C	-5.322205000	-2.780156000	0.793921000
C	-4.109763000	-3.427423000	0.959313000
C	-2.916003000	-2.755462000	0.721594000
C	-3.102159000	2.778691000	-0.643938000
C	-4.341093000	3.393302000	-0.782244000
C	-5.505424000	2.679740000	-0.556447000
C	-5.455927000	1.321870000	-0.224268000
C	-6.663974000	0.570802000	-0.014070000
C	-6.621606000	-0.738980000	0.308271000
H	3.905255000	5.034328000	0.514161000
H	1.219550000	5.042514000	0.240043000
H	-0.543759000	3.280206000	-0.023498000
H	7.641957000	-1.379855000	0.403899000
H	7.641802000	1.284758000	0.772603000
H	5.703292000	3.197638000	0.593043000
H	1.229217000	-4.816819000	-1.293920000
H	3.913491000	-4.850134000	-1.243808000
H	-0.613303000	-3.133295000	-0.481424000
H	5.786596000	-3.158495000	-0.351172000
H	-6.252508000	-3.308676000	0.968476000
H	-4.086974000	-4.459107000	1.287831000
H	-1.985918000	-3.267401000	0.922177000
H	-2.218560000	3.351149000	-0.880500000
H	-4.391753000	4.434026000	-1.077237000
H	-6.470899000	3.163096000	-0.653904000
H	-7.611670000	1.087156000	-0.115561000
H	-7.533975000	-1.299480000	0.476852000

MePyPaH^{+}

C	-1.761266000	0.748378000	0.334616000
C	-1.681405000	-0.643181000	0.299978000
C	-0.341767000	-1.017909000	0.666991000
N	0.377837000	0.143280000	0.895291000
C	-0.456053000	1.239949000	0.680165000
C	3.173574000	4.203022000	0.131218000
C	1.887309000	4.249732000	0.590307000
C	1.337079000	2.934640000	0.502339000
N	2.331934000	2.122689000	0.009533000
C	3.468380000	2.855127000	-0.241301000
C	0.020973000	2.545447000	0.747232000
C	6.504248000	-0.702597000	-1.174197000
C	6.428684000	0.656833000	-1.261860000
C	5.116703000	1.062954000	-0.845779000
N	4.433129000	-0.084042000	-0.539680000
C	5.239518000	-1.176971000	-0.703162000
C	4.690267000	2.378045000	-0.699604000
C	1.906212000	-4.112390000	0.829074000
C	3.198262000	-4.187262000	0.456100000
C	3.588529000	-2.827591000	0.091748000
N	2.558110000	-1.958706000	0.255881000
C	1.517209000	-2.707075000	0.684188000
C	0.186520000	-2.294258000	0.825095000
C	4.848086000	-2.475154000	-0.391673000
H	2.132518000	1.199990000	-0.353404000
H	3.489226000	-0.235112000	-0.190637000

C	-2.989566000	1.446225000	0.021302000
C	-4.153538000	0.636553000	-0.131958000
C	-4.063056000	-0.794470000	-0.194313000
C	-2.806696000	-1.459690000	-0.093393000
C	-5.234110000	-1.557055000	-0.442762000
C	-5.129343000	-2.939425000	-0.636652000
C	-3.891909000	-3.558109000	-0.639986000
C	-2.738619000	-2.825729000	-0.378094000
C	-3.110249000	2.831191000	-0.107350000
C	-4.352697000	3.429287000	-0.290684000
C	-5.497277000	2.656265000	-0.361487000
C	-5.416976000	1.260068000	-0.307652000
C	-6.593897000	0.450141000	-0.467942000
C	-6.506666000	-0.894900000	-0.534458000
C	1.382261000	0.179176000	1.980850000
H	3.882605000	5.014734000	0.072285000
H	1.352684000	5.107568000	0.968477000
H	-0.676235000	3.346347000	0.940181000
H	7.349616000	-1.334477000	-1.398900000
H	7.201569000	1.343498000	-1.571962000
H	5.433663000	3.135201000	-0.918384000
H	1.253540000	-4.906553000	1.160967000
H	3.841076000	-5.054075000	0.415409000
H	-0.527242000	-3.087401000	1.000709000
H	5.584159000	-3.258655000	-0.524612000
H	-6.028950000	-3.513325000	-0.828173000
H	-3.815589000	-4.616468000	-0.856344000
H	-1.783968000	-3.325011000	-0.445713000
H	-2.236816000	3.463699000	-0.102702000
H	-4.418817000	4.506136000	-0.384362000
H	-6.466355000	3.123095000	-0.496324000
H	-7.551132000	0.950240000	-0.560747000
H	-7.391836000	-1.502710000	-0.682977000
H	1.171975000	-0.639805000	2.667163000
H	2.396306000	0.059170000	1.613488000
H	1.288585000	1.125765000	2.508009000

$MePyPbH^+$

C	-1.783410000	0.708958000	0.455264000
C	-1.684542000	-0.671902000	0.324902000
C	-0.336566000	-1.053839000	0.654100000
N	0.405906000	0.107344000	0.842818000
C	-0.444999000	1.208247000	0.644365000
C	3.039942000	4.277952000	-0.401014000
C	1.730619000	4.276491000	-0.005626000
C	1.321223000	2.917883000	0.172830000
N	2.405588000	2.141153000	-0.112975000
C	3.470745000	2.919840000	-0.478623000
C	0.018197000	2.497132000	0.477234000
C	6.545578000	-0.635796000	-0.966319000
C	6.490764000	0.697465000	-1.163998000
C	5.129953000	1.098913000	-0.836122000
N	4.372364000	0.027192000	-0.470014000
C	5.214792000	-1.033558000	-0.528901000
C	4.719681000	2.430594000	-0.841637000
C	1.976445000	-4.082013000	1.046947000
C	3.304334000	-4.108449000	0.738071000
C	3.669726000	-2.807907000	0.257961000
N	2.536156000	-2.038878000	0.302456000
C	1.479161000	-2.763925000	0.765257000
C	0.153292000	-2.339046000	0.844974000
C	4.901930000	-2.352807000	-0.170601000
H	2.448384000	1.133507000	-0.178178000
H	2.523218000	-1.107990000	-0.094747000
C	-3.054691000	1.388258000	0.346713000
C	-4.171053000	0.596489000	-0.050638000
C	-4.031563000	-0.803414000	-0.334000000
C	-2.780411000	-1.467562000	-0.180601000
C	-5.157185000	-1.538413000	-0.788606000
C	-5.009646000	-2.889500000	-1.124626000
C	-3.772753000	-3.503770000	-1.046842000
C	-2.665464000	-2.800431000	-0.583324000

C	-3.262429000	2.722781000	0.701912000
C	-4.522167000	3.302892000	0.602117000
C	-5.599352000	2.559782000	0.152919000
C	-5.448614000	1.205240000	-0.165267000
C	-6.572510000	0.422515000	-0.602150000
C	-6.431852000	-0.884537000	-0.907456000
C	1.415827000	0.186176000	1.926268000
H	3.664157000	5.130551000	-0.620119000
H	1.088063000	5.128498000	0.155299000
H	-0.709923000	3.292106000	0.448737000
H	7.385504000	-1.303190000	-1.090010000
H	7.276508000	1.365780000	-1.483735000
H	5.455247000	3.171582000	-1.133010000
H	1.372472000	-4.884681000	1.441588000
H	3.989640000	-4.936593000	0.835278000
H	-0.574430000	-3.116723000	1.031066000
H	5.703120000	-3.081380000	-0.210125000
H	-5.874610000	-3.442228000	-1.473345000
H	-3.659334000	-4.535060000	-1.357268000
H	-1.703440000	-3.290716000	-0.590089000
H	-2.459152000	3.315286000	1.113411000
H	-4.658659000	4.338525000	0.887585000
H	-6.578998000	3.015366000	0.063312000
H	-7.536165000	0.911850000	-0.685197000
H	-7.278585000	-1.469939000	-1.247075000
H	1.229804000	-0.624647000	2.630538000
H	2.442947000	0.107994000	1.570968000
H	1.296120000	1.139979000	2.436199000

PyPH_2^{2+}

C	1.711399000	-0.702866000	-0.043676000
C	1.711251000	0.702418000	-0.043595000
C	0.366972000	1.133627000	-0.283135000
N	-0.446789000	-0.000392000	-0.296374000
C	0.367240000	-1.134260000	-0.283221000
C	-3.306073000	-4.096130000	-0.720039000
C	-1.965434000	-4.069377000	-0.989909000
C	-1.453924000	-2.800267000	-0.586259000
N	-2.526835000	-2.052382000	-0.137454000
C	-3.673099000	-2.835268000	-0.164038000
C	-0.118542000	-2.401971000	-0.573268000
C	-6.543048000	0.685274000	1.139669000
C	-6.542947000	-0.685075000	1.139830000
C	-5.316841000	-1.128141000	0.566953000
N	-4.567516000	0.000108000	0.280816000
C	-5.316990000	1.128355000	0.566707000
C	-4.928961000	-2.431988000	0.269147000
C	-1.965400000	4.068962000	-0.990476000
C	-3.306081000	4.095925000	-0.720762000
C	-3.673252000	2.835212000	-0.164562000
N	-2.527088000	2.052327000	-0.137620000
C	-1.454076000	2.799872000	-0.586570000
C	-0.118669000	2.401317000	-0.573313000
C	-4.929166000	2.432093000	0.268709000
C	2.917458000	-1.460431000	0.142359000
C	4.135469000	-0.715857000	0.105584000
C	4.135239000	0.716128000	0.105726000
C	2.916968000	1.460292000	0.142655000
C	2.962690000	-2.843050000	0.347992000
C	4.180569000	-3.508746000	0.406691000
C	5.368658000	-2.806206000	0.277250000
C	5.371514000	-1.411447000	0.152303000
C	5.371038000	1.412129000	0.152621000
C	5.367673000	2.806862000	0.277865000
C	4.179331000	3.508990000	0.407386000
C	2.961699000	2.842900000	0.348518000
H	-3.900321000	0.000011000	-0.481525000
H	-2.402883000	1.323885000	0.555530000
H	-2.402297000	-1.324533000	0.556289000
H	-1.233101000	-0.000500000	-0.939489000
C	6.605114000	-0.674521000	0.113897000
C	6.604891000	0.675644000	0.114046000

H	-3.999323000	-4.901777000	-0.911936000
H	-1.369660000	-4.851725000	-1.436167000
H	0.604366000	-3.162700000	-0.834325000
H	-7.314968000	1.341470000	1.513677000
H	-7.314791000	-1.341287000	1.513961000
H	-5.686380000	-3.201691000	0.360799000
H	-1.369504000	4.851126000	-1.436888000
H	-3.999215000	4.901631000	-0.912825000
H	0.604409000	3.162031000	-0.833973000
H	-5.686541000	3.201855000	0.360270000
H	2.054882000	-3.405441000	0.515990000
H	4.201115000	-4.579223000	0.567579000
H	6.316267000	-3.331471000	0.315836000
H	6.315084000	3.332476000	0.316655000
H	4.199541000	4.579443000	0.568472000
H	2.053686000	3.404957000	0.516579000
H	7.535813000	-1.229644000	0.110566000
H	7.535400000	1.231084000	0.110839000

MePyPH$_2^{2+}$

C	-1.679699000	-0.659096000	0.263295000
C	-1.794319000	0.735128000	0.405335000
C	-0.460612000	1.235446000	0.572589000
N	0.422996000	0.140475000	0.776056000
C	-0.342450000	-1.025105000	0.608660000
C	3.300782000	-4.051892000	0.942753000
C	1.968904000	-4.021569000	1.237452000
C	1.454526000	-2.742452000	0.846633000
N	2.515572000	-2.018992000	0.354350000
C	3.659798000	-2.791896000	0.361441000
C	0.132357000	-2.311437000	0.864493000
C	6.353189000	0.629288000	-1.463545000
C	6.403824000	-0.727747000	-1.271261000
C	5.242320000	-1.115927000	-0.551681000
N	4.479511000	0.025115000	-0.346679000
C	5.160491000	1.119801000	-0.865278000
C	4.895318000	-2.398933000	-0.125180000
C	1.769690000	4.301040000	0.089700000
C	3.090602000	4.275710000	-0.264101000
C	3.485644000	2.910945000	-0.408131000
N	2.382946000	2.142352000	-0.095696000
C	1.310251000	2.952902000	0.179040000
C	-0.004155000	2.532248000	0.425009000
C	4.728634000	2.440158000	-0.814219000
C	-2.762316000	-1.462773000	-0.236261000
C	-4.024632000	-0.810969000	-0.370397000
C	-4.179062000	0.582591000	-0.077518000
C	-3.069819000	1.393153000	0.315225000
C	-2.628907000	-2.793549000	-0.645857000
C	-3.732452000	-3.510264000	-1.093640000
C	-4.978697000	-2.909030000	-1.152062000
C	-5.145362000	-1.559671000	-0.812403000
C	-5.465940000	1.172475000	-0.178034000
C	-5.629465000	2.524399000	0.148507000
C	-4.559874000	3.285203000	0.592740000
C	-3.292231000	2.725384000	0.679161000
H	3.918212000	0.118059000	0.493128000
H	2.255630000	1.175466000	-0.359986000
H	2.378829000	-1.232141000	-0.265729000
C	-6.430719000	-0.926171000	-0.915890000
C	-6.585063000	0.378546000	-0.605220000
C	1.183633000	0.241035000	2.057987000
H	3.996933000	-4.857703000	1.122832000
H	1.379083000	-4.799904000	1.698235000
H	-0.610483000	-3.071660000	1.071051000
H	7.063358000	1.242221000	-1.998833000
H	7.162125000	-1.412214000	-1.621469000
H	5.661309000	-3.161977000	-0.197076000
H	1.157405000	5.168725000	0.284214000
H	3.750855000	5.118247000	-0.406813000
H	-0.734572000	3.326195000	0.394876000
H	5.438195000	3.189608000	-1.145785000

H	-1.657922000	-3.267600000	-0.673318000
H	-3.612855000	-4.538307000	-1.411505000
H	-5.838983000	-3.474223000	-1.492418000
H	-6.616006000	2.967405000	0.071558000
H	-4.713464000	4.316038000	0.885774000
H	-2.495924000	3.328521000	1.090088000
H	-7.273082000	-1.521952000	-1.247236000
H	-7.555733000	0.855209000	-0.675608000
H	1.673413000	1.209362000	2.114230000
H	0.490345000	0.142734000	2.896627000
H	1.931002000	-0.544600000	2.120884000
